# Combining photochemistry and catalysis: rapid access to sp^3^ – rich polyheterocycles from simple pyrroles†
†Electronic supplementary information (ESI) available: Detailed experimental procedures and spectroscopic data for all new compounds. CCDC 1441499–1441501. For ESI and crystallographic data in CIF or other electronic format see DOI: 10.1039/c5sc04062k


**DOI:** 10.1039/c5sc04062k

**Published:** 2016-01-04

**Authors:** Emma E. Blackham, Jonathan P. Knowles, Jonathan Burgess, Kevin I. Booker-Milburn

**Affiliations:** a School of Chemistry , University of Bristol , Cantock's Close , Bristol , BS8 1TS , UK . Email: k.booker-milburn@bristol.ac.uk ; http://www.chm.bris.ac.uk/org/bmilburn/index2.htm

## Abstract

Use of FEP flow reactor technology allows access to gram quantities of photochemically-generated tricyclic aziridines. These undergo a range of novel palladium-catalyzed ring-opening and cycloaddition reactions, likely driven by their inherent strain, allowing incorporation of further functionality by fusing additional heterocyclic rings onto these already complex polycyclic cores. This rapid, 2-step access to complex sp^3^ – rich heterocycles should be of interest to those in the fields of drug discovery and natural product synthesis.

## Abstract 2

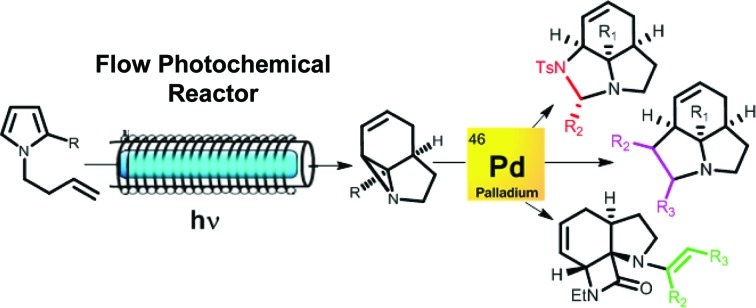
The powerful combination of photochemistry and catalysis transforms simple pyrroles into complex polycyclic structures in just two steps. Initial formation of highly reactive aziridines enables new Pd-catalysed cascade sequences, forming a range of polyheterocycles including tricyclic β-lactams.

## Introduction

**Scheme 1:**
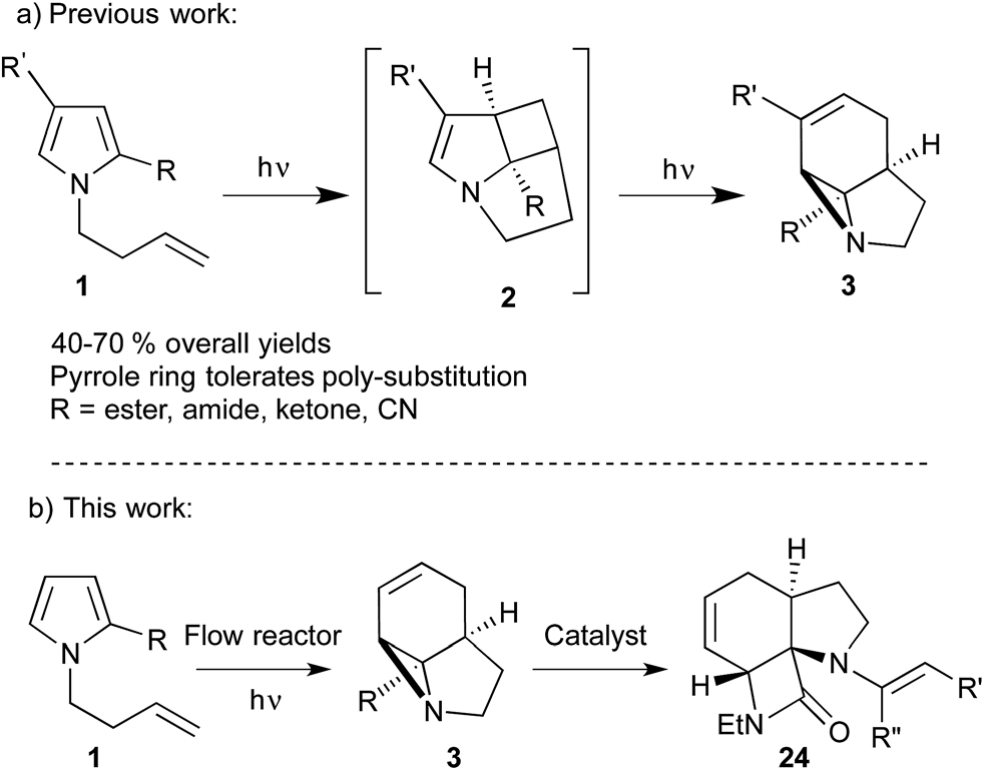
(a) Formation of aziridines by photocycloaddition/rearrangement of pyrrole derivatives. (b) Scale-up of photochemical aziridine synthesis and subsequent catalytic ring opening to generate polyheterocycles.

The strained 3-membered aziridine ring has long been exploited in a variety of ring-opening reactions, serving as a valuable strategy in the synthesis of amino compounds. Compared to epoxides, however, aziridines are generally less reactive due the lower electronegativity of N *vs.* O. Consequently the reactivity of aziridines is usually enhanced by carrying out reactions with nucleophiles in the presence of Brønsted or Lewis acids, or by the attachment of an electron withdrawing group to the nitrogen.^1^ The latter approach, however, restricts the type of aziridines that can be used and requires removal of the activating group in a subsequent step. We recently described a photochemical transformation of simple *N*-butenyl substituted pyrroles **1** into tricyclic fused aziridines **3**, *via* cyclobutane **2** (Scheme 1a).^2^ However, we found the batch scale-up of this very powerful sequence to be somewhat limited. This is due to the low overall quantum yield of this 2-step, 2-photon sequence and the fact that, due to the high extinction coefficient (*ε* = 13 000), the reaction has to be run at high dilution (<0.02 mM). This made further study of the chemistry of the resulting aziridines challenging.

Herein we describe the production of multigram quantities of aziridines using fluorinated ethylene propylene (FEP) flow reactor technology, a concept that has seen increasing use in recent years.^3^ The resulting structural complexity and close proximity of functional groups within these aziridines prompted us to explore their reactivity with various nucleophiles and ring-opening conditions. Their interesting, and sometimes unusual results are reported and include a novel β-lactam forming sequence (Scheme 1b).

## Results and discussion

**Scheme 2:**
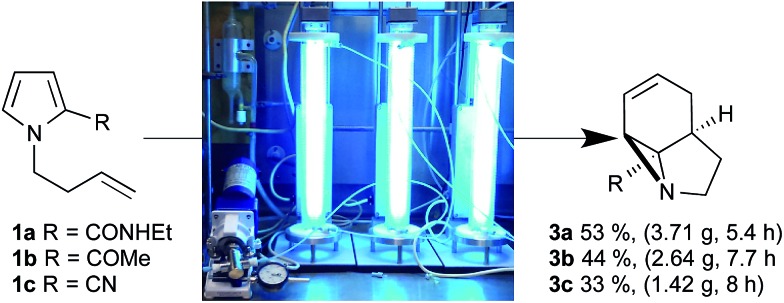
Scale up of photochemical aziridine synthesis using a trio of 1-layer FEP reactors with 36 W low-pressure Hg lamps.

Initially, we examined the scale-up irradiations of the three pyrroles **1a–c** at 254 nm. This used the previously described trio of 1-layer FEP reactors with 36 W low-pressure Hg lamps.3*b* The three reactors were connected together in series for maximum productivity. Once optimized this gave gram quantities of the three aziridines **3a–c** per 5–8 h run (Scheme 2).

**Scheme 3:**
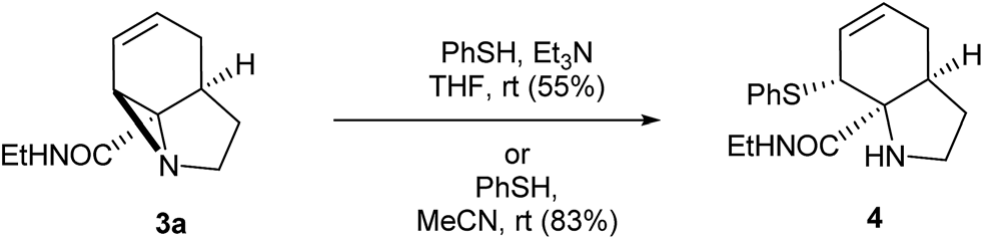
Initial ring opening reactions of aziridine **3a**.

With these quantities in hand we first explored the ring opening of the amide-aziridine **3a** with nucleophiles (Scheme 3). Using thiophenol the aziridine underwent S_N_2 ring-opening under mild conditions in THF in the presence of triethylamine. The high reactivity of the unactivated aziridine ring was highlighted by the clean ring opening with thiophenol in acetonitrile without added base to give **4**. We considered that this ring-opening of an unactivated aziridine under such mild conditions pointed towards further strain imposed by the tricyclic system. Unfortunately ring opening could not be achieved using nucleophiles such as phenol or methanol under neutral or basic conditions. When the reaction was repeated with methanol under acid catalysis (*p*-TSA) an inseparable mixture of products was obtained which appeared to be a mixture of S_N_2′ ring-opened diastereomers. A screen of other catalysts was not carried out as the aziridine was prone to decomposition under acidic conditions.

Literature reports of Pd-catalysed Tsuji–Trost ring-opening reactions of vinyl aziridines are rare.^4^ Indeed there would appear to be only two reports involving the addition of carbon-nucleophile in an S_N_2′ mode.^5^ We were intrigued therefore to see if our highly strained systems would extend the scope of these reactions to a wider range of nucleophiles. Treatment of **3a** with phenol and 5 mol% Pd(PPh_3_)_4_ in dioxane gave exclusively the *anti* product **5** (entry 1, Table 1). Despite the lack of literature precedent we then examined the reaction with a range of carbon-nucleophiles. Pleasingly the reaction of **3a** and **3b** also proceeded smoothly with a selection of soft carbon-nucleophiles (entries 3–10). It was also found that the *anti*/*syn* ratios could be switched on moving to more polar solvents.^6^ For example, reaction of **3a** in DMF at 80 °C resulted in a 1 : 4 ratio of **5** : **6** (Nu = PhO, entry 2). Similarly reaction of **3a** in MeCN with pentane-2,3-dione provided a switch in the *anti*/*syn* ratio to 1 : 9 (entry 6). This switch is likely to reflect a change from an outer sphere mechanism6a,b (direct attack of nucleophile on carbon – overall retention) to an inner sphere process6*c* (initial attack on metal – overall inversion) on moving to a more polar solvent. Such behaviour has been observed in Tsuji–Trost reactions of allylic acetates on switching from soft to hard nucleophiles,6*d* and appears to reflect the decrease in stability of the anion on moving to more polar solvents as judged from the p*K*_a_ values of phenol in this case.^7^

**Table 1:** Ring opening of aziridines with nucleophiles under Pd(0) catalysis

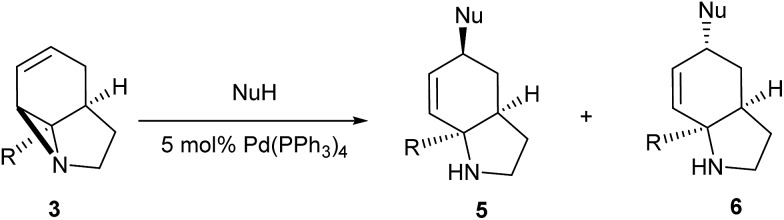						
Entry	R	Solvent	NuH	*T* (°C)	Yield (%)	**5** : **6**
1	CONHEt	Dioxane	PhOH	rt	49	1 : 0
2	CONHEt	DMF	PhOH	80	52	1 : 4
3	CONHEt	Dioxane	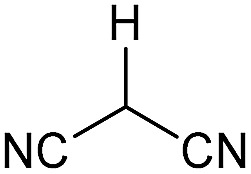	40	52	1 : 0
4	CONHEt	MeCN	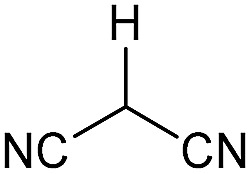	40	57	1 : 1
5	CONHEt	Dioxane	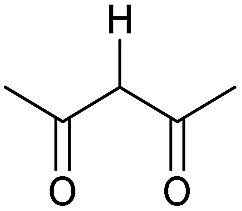	80	82	1 : 0
6	CONHEt	MeCN	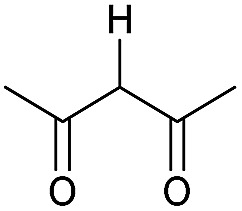	80	67	1 : 9
7	CONHEt	Dioxane	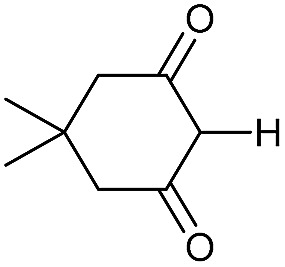	rt	75	7 : 1
8	CONHEt	Dioxane	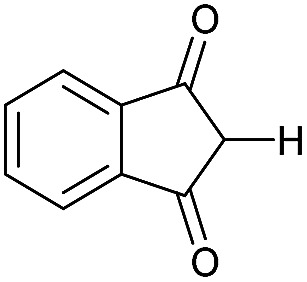	rt	64	1 : 0
9	COMe	Dioxane	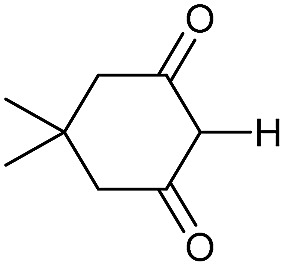	rt	52	13 : 1
10	COMe	Dioxane	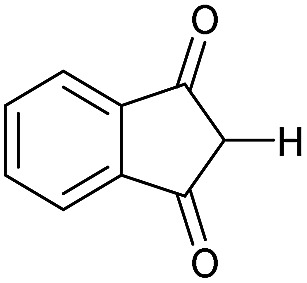	rt	43	5 : 1

It has been previously demonstrated^8^ that *N*-alkyl substituted 2-vinyl aziridines reacted with isocyanates under Pd(0) catalysis to generate vinyl substituted cyclic ureas. If this could be applied to **3** then highly functionalised tricyclic ureas such as **7** would be accessible in just two steps from simple pyrroles. Table 2 describes the reaction between a wide range of aryl and sulfonyl isocyanates with tricyclic aziridines under Pd(0) catalysis. In general, the ureas **7** were formed in good yield using a 5 mol% loading of Pd(PPh_3_)_4_. It was found that the amide-aziridine **3a** reacted more consistently than the COMe (**3b**) or CN (**3c**) substituted derivatives. A range of aryl isocyanates were tolerated, although 2-substituted examples tended to give lower yields and 2,6-substitution lead to mixed products from N/O cyclisation (entry 17). An interesting observation was noted with low (1 mol%) catalyst loadings (entries 19 & 20) and indeed no catalyst (entry 18), where exclusive formation of the cyclic imidates **8** was observed. In the case entry 19 increasing the quantity of Pd to 5 mol% results in rapid conversion of **8** to **7**. These results are in good agreement with those observed by Alper for simpler vinyl aziridines.8*a*

**Table 2:** Pd-catalysed [3 + 2] cycloadditions with isocycanates

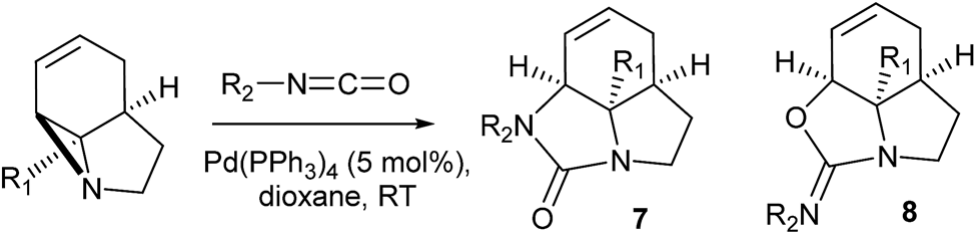				
Entry	R	R_2_	Yield (%)	**7** : **8**
1	CONHEt	4-MeC_6_H_4_SO_2_	83	1 : 0
2	CN	4-MeC_6_H_4_SO_2_	81	1 : 0
3	COMe	4-MeC_6_H_4_SO_2_	43	1 : 0
4	CONHEt	4-ClC_6_H_4_	58	1 : 0
5	CONHEt	2-ClC_6_H_4_	71	1 : 0
6	CN	2-ClC_6_H_4_	49	1 : 0
7	CONHEt	2-CF_3_C_6_H_4_	81	1 : 0
8	CONHEt	3-CF_3_C_6_H_4_	91	1 : 0
9	CONHEt	4-CF_3_C_6_H_4_	54	1 : 0
10	COMe	4-CF_3_C_6_H_4_	55	1 : 0
11	CONHEt	2-NO_2_C_6_H_4_	89	1 : 0
12	CN	2-NO_2_C_6_H_4_	54	1 : 0
13	CONHEt	4-NO_2_C_6_H_4_	89	1 : 0
14	CONHEt	4-MeCOC_6_H_4_	79	1 : 0
15	CONHEt	4-MeOC_6_H_4_	80	1 : 0
16	CONHEt	2-MeOC_6_H_4_	38	1 : 0
17	CONHEt	2,6-Cl_2_C_6_H_3_	80	10 : 1
18^*a*^	CONHEt	4-MeC_6_H_4_SO_2_	51	0 : 1
19^*b*^	CONHEt	4-MeC_6_H_4_SO_2_	85	0 : 1
20^*b*^	CONHEt	2-ClC_6_H_4_	96	0 : 1

^*a*^Uncatalysed, dioxane, rt.

^*b*^1 mol% Pd(Ph_3_P)_4_, dioxane, rt.

We then examined the potential [3 + 2] cycloaddition reactions of alkenes with the aziridines, as rapid access to the resulting tricyclic fused pyrrolidines would be attractive for alkaloid synthesis. Such Pd-catalysed [3 + 2] cycloadditions have been examined previously, but generally require activated aziridines.^9^ Initial results between **3a** and simple alkenes such as methyl acrylate, acrylonitrile and methyl vinyl ketone showed no reactions. After much optimisation, benzylidine malononitrile was found to react under the specific conditions shown in Table 3 to give the pyrrolidine **10a** as essentially one diastereomer. Higher yields were obtained by the use of the more reactive methylene diesters, which led to the formation of the tricyclic pyrrolidines **11a** and **12a**, **b**. Key to the success of these reactions was the use of triphenylphosphite as ligand;^10^ phosphine ligands gave lower yields and polymerisation of the reactive methylene diesters. However, use of nitrile aziridine **3c** gave lower yields of the corresponding products (*i.e.***12c** & **15c**), with appreciable recovery of the starting material, due to the reduced reactivity of this substrate.

**Table 3:** Pd-catalysed [3 + 2] cycloadditions of aziridines with alkenes, aldehydes and imines

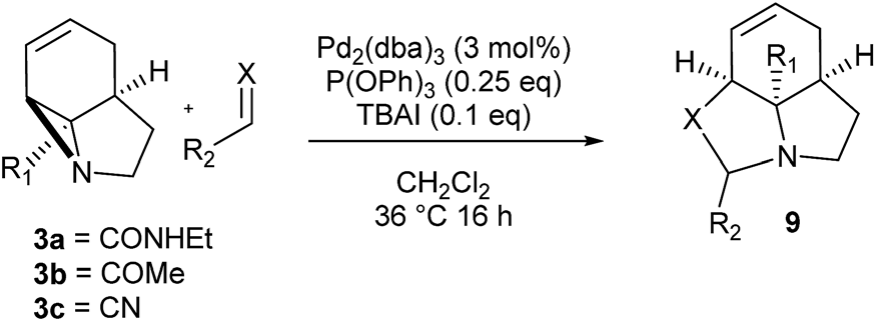
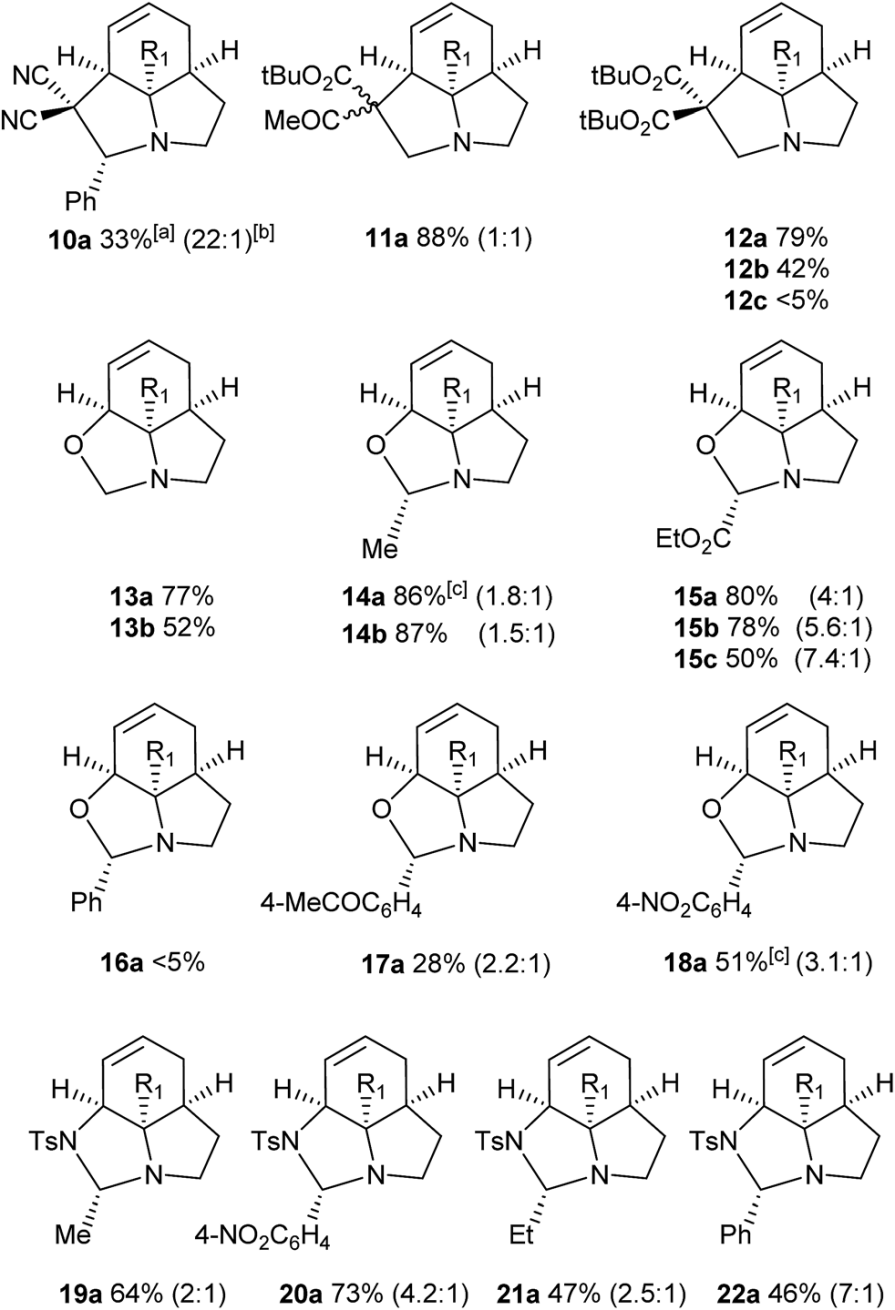

^*a*^Required 64 h.

^*b*^Diastereomeric ratio in parentheses.

^*c*^400 mol% of dipolarophile used.

There have been a number of reports of the reaction of aldehydes with aziridines in a [3 + 2] manner, but with few exceptions^11^ these all involved activated aziridines under Lewis acid catalysis.^12^ We were intrigued to discover whether our strained aziridines would undergo [3 + 2] cycloadditions with aldehydes under the aforementioned optimised Pd-catalysed conditions. With the exception of benzaldehyde, a range of aldehydes reacted well with the aziridines to give a variety of functionalised oxazolidines with good diastereoselection (**13–18**). These results highlight the exceptional reactivity of these tricyclic aziridines compared to the more conventionally activated documented examples. It should be noted that none of these reactions gave oxazolidines in the absence of palladium (*vide infra*). Following on from this success we were keen to explore the use of imines in this reaction. Gratifyingly, it was found that a selection *N*-Ts imines reacted with **3a** to give the tricyclic fused aminals **19–22** with reasonable to good levels of diastereoselection. This appears to be a previously unreported mode of addition for aziridines, allowing novel access to highly functionalised aminals.^13^

Finally, we decided to examine whether the limited scope of [3 + 2] cycloaddition reactions of alkenes (3 examples, Table 3) could be extended using alkyne based Michael acceptors. It was envisioned that the aziridines would react with alkynes under Pd-catalysis to generate tricyclic systems containing a partially unsaturated pyrrolidine ring. This proved successful in the reaction of **3c** with a range of alkynes and gave rapid access to the highly functionalised dihydropyrrole **23**. Interestingly, these cycloadditions proceeded equally well with or without catalyst (Table 4) suggesting, in this case, a purely thermal process. Moving to amide **3a** a quite different outcome was observed, and remarkably the tricyclic fused β-lactams **24** were obtained. This sequence produces a single diastereomeric product (Table 5). One notable feature here is that although Pd-catalysis generally gave the highest yields and shortest reaction times, the reactions were also found to proceed without catalyst at room temperature.

**Table 4:** [3 + 2] Cycloadditions of alkynes with cyanoaziridine **3c**

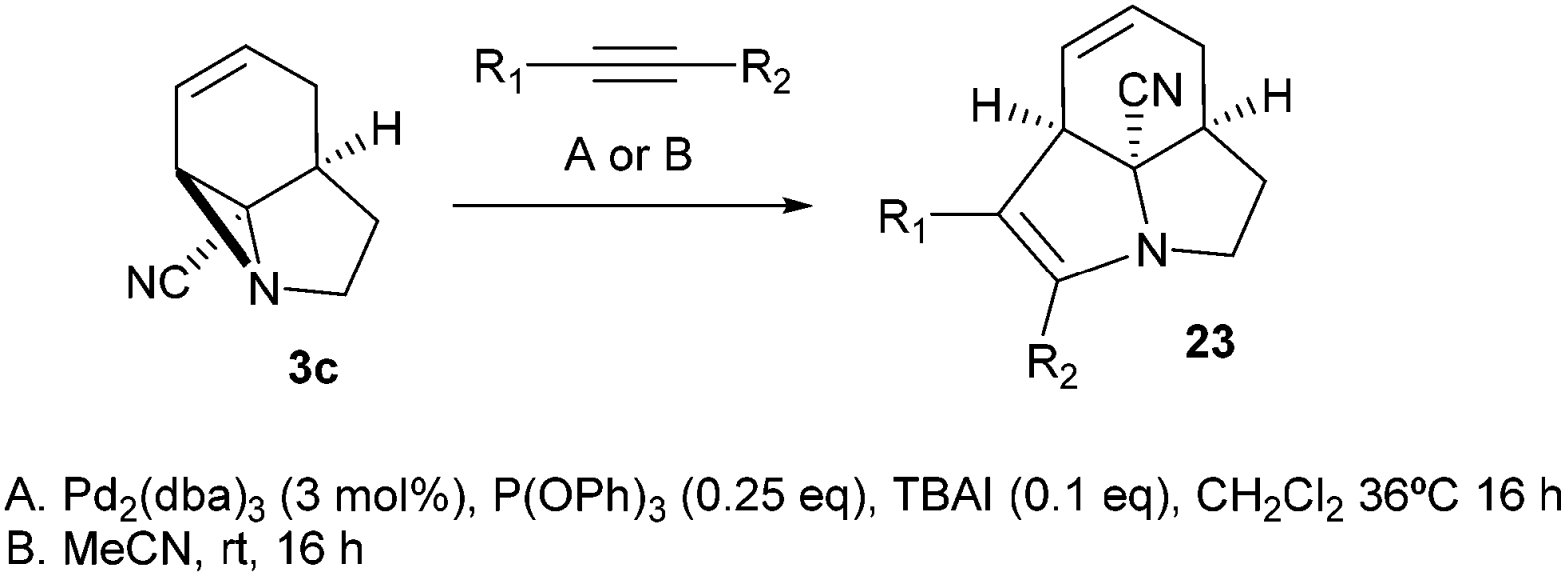				
Entry	R_1_	R_2_	Conditions **A** yield (%)	Conditions **B** yield (%)
1	COMe	H	53	56
2	CHO	TMS^*a*^	60	62
3	CO_2_Me	CO_2_Me	92	94
4	CN	H	37	18

^*a*^Desilylated product obtained on work-up (R_1_ = CHO; R_2_ = H).

**Table 5:** Formation of β-lactams by a novel Pd-catalysed addition/cyclisation sequence with alkynes

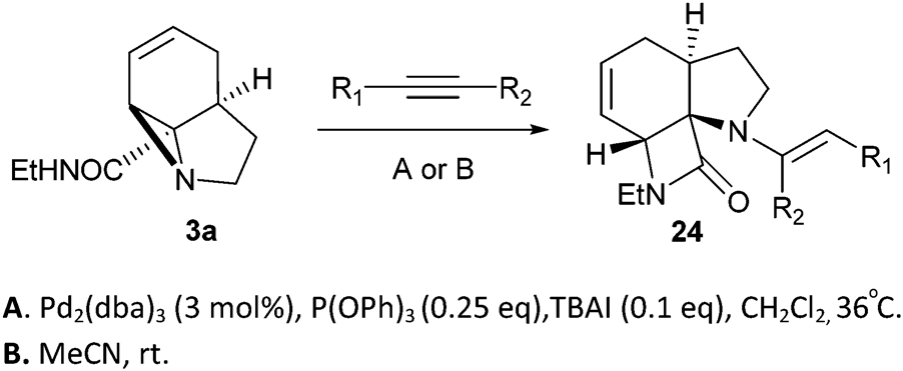						
Entry	R_1_	R_2_	*t* (h)	Conditions **A** yield (%)	*t* (h)	Conditions **B** yield (%)
1	COMe	H	16	98	16	82
2	CO_2_Me	H	16	83	24	54
3	CO_2_Et	H	16	78	47	61
4	CONH_2_	H	16	0	16	0
5	CHO	TMS^*a*^	16	46^*b*^	20	60^*c*^
6	CO_2_Me	CO_2_Me	16	32	6.5	48

^*a*^Desilylated product obtained on work-up (R_1_ = CHO; R_2_ = H).

^*b*^15% of an oxazolidinone by-product was also isolated – see ESI for details.

^*c*^Includes 9% of the imidate isomer – see ESI for details.

**Scheme 4:**
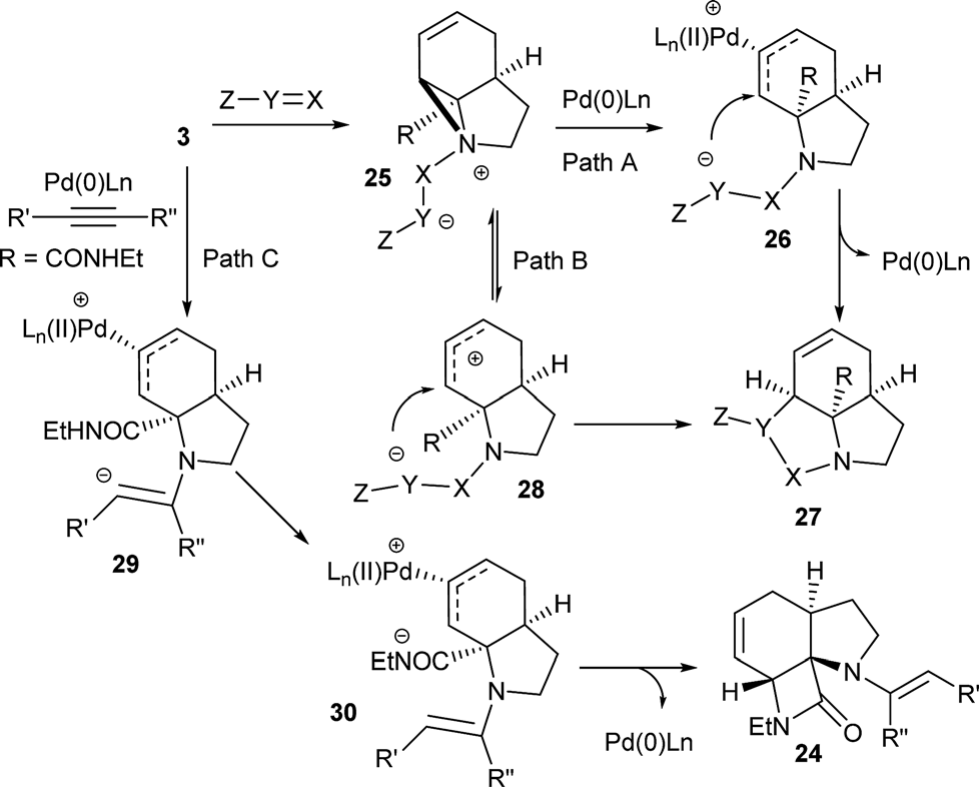
Plausible mechanistic rationale for formation of all products.

Although these reactions may seem diverse, mechanistically they are all likely to be related, with subtle differences depending on the substrates and conditions used. The unusual Tsuji–Trost type ring opening reactions described in Table 1 are likely to proceed *via* a conventional pathway where strain induces a higher reactivity compared to standard non-activated aziridines. It is possible, however, the aziridine is further activated by initial protonation from the carbon-nucleophiles used, which would not be possible for more conventionally activated aziridines (*e.g.* NTs). The reactions in Tables 2 and 3 can be described by the mechanisms in Scheme 4. Firstly the aziridine **3** reacts by nucleophilic attack on the isocyanate/ketone/aldehyde/imine to generate the zwitterion **25**. This is in contrast to initial attack of Pd to give a π-allyl Pd complex as previously described by Alper.8*a* We propose this based on the absence of any Pd-induced reactions (*e.g.* ring opening/β-hydride elimination) when the electrophile is either not present or insufficiently reactive. Palladium then reacts with the zwitterion **25** to give π-allyl Pd complex **26** (Path A), which after cyclisation forms the various heterocycles **27** and regenerates the Pd(0) catalyst. Where the reaction proceeds without catalyst (*e.g.*Table 2, entry 18) it is possible that the zwitterion **25** is in equilibrium with the ring-opened form **28**, which then undergoes cyclisation to **27** (Path B). In the case of β-lactam formation it is likely that after the aziridine adds to the alkyne and undergoes ring opening with Pd(0), the resulting vinyl anion **29** deprotonates the secondary amide to give **30** which then cyclises to the 4-membered lactam (Path C). The higher p*K*_a_ of the vinyl anion **29** compared to other species (*i.e.***26** & **28**) explains why β-lactam formation was not observed in other cases.

## Conclusions

In summary, highly strained and compact fused tricyclic aziridines have been shown to undergo a range of ring-opening and cycloaddition reactions under mild conditions. This is in contrast with the usual behaviour of aziridines, which normally require an activating electron-withdrawing group to be placed on the nitrogen. Notable features include: (a) a very rare and extremely mild Tsuji–Trost type aziridine ring-opening using carbon-nucleophiles; (b) a Pd-catalysed [3 + 2] cycloaddition of aldehydes and imines, which has hitherto not been reported; (c) a novel, mild and effective method for the stereo-controlled formation of tricyclic fused β-lactams. Together these diverse reactions highlight the power of combining an initial photochemical step with a secondary catalytic process, in this case resulting in highly complex products in just two steps from simple pyrroles. We have also shown that the photochemical step to give these complex aziridines intermediates is readily scalable using the FEP flow reactors we have previously described,3a–c allowing access to gram quantities of the substrates for these catalytic processes. As such these results may prove useful to chemists looking to exploit short routes to complex, sp^3^-rich compounds as potential scaffolds in drug discovery and alkaloid synthesis.

## Supplementary Material

Supplementary information

Supplementary information

Crystal structure data
