# Supplementary material for: Combining photochemistry and catalysis: rapid access to sp3 – rich polyheterocycles from simple pyrroles
Source: Chem Sci. 2016 Jan 4;7(3):2302–7. doi: 10.1039/c5sc04062k (PMC5977501; doi:10.1039/c5sc04062k)
Supplement: Supplementary file 1 [file SC-007-C5SC04062K-s001.pdf]

Electronic Supplementary Material (ESI) for Chemical Science.
This journal is © The Royal Society of Chemistry 2016

Supporting Information

Combining Photochemistry and Catalysis: Rapid Access to sp^3 - Rich Polyheterocycles from Simple Pyrroles

Emma E. Blackham, Jonathan P. Knowles, Jonathan Burgess and Kevin I. Booker-Milburn

General Experimental: Unless specified otherwise for all non-aqueous chemistry, glassware was flame-dried under an inert (N_2 or Ar) atmosphere. Cryogenic conditions (-78 ^oC) were achieved using solid carbon dioxide/acetone baths. Temperatures of 0 ^oC were obtained by means of an ice bath. Room temperature indicates temperatures in the range of 20-25 ^oC. For the purposes of thin layer chromatography (tlc), Merck silica-aluminium plates were used, with *uv* light (254 nm) and potassium permanganate used for visualisation. For column chromatography, Sigma Aldrich technical grade 60 Å silica gel was used. All NMR data was collected using a Jeol Eclipse 400 MHz or Varian 400-MR instruments. Data was processed directly using MestReNova (version 9.0). Reference values for residual solvents were taken as δ = 7.27 ($CDCl_3$) and 2.51 ppm (DMSO-*d*6) for ^1H NMR; δ = 77.16 ppm ($CDCl_3$) for ^{13}C NMR. Multiplicities for coupled signals were denoted as: s = singlet, d = doublet, t = triplet, q = quartet, m = multiplet, br. = broad, app. = apparent and dd = double doublet *etc.* Coupling constants (*J*) are given in Hz and are uncorrected. Where appropriate, COSY, DEPT, HMBC, HMQC and NOE experiments were carried out to aid assignment. Mass spectrometry data was collected was carried out by the University of Bristol mass spectrometry service using Fisons Autospec or Bruker Daltonics MicrOTOF II instruments. Infrared data was collected using a Perkin-Elmer Spectrum One FTIR machine. Melting points are uncorrected and were recorded on Stuart Scientific apparatus. Anhydrous solvents were obtained from a solvent tower, where degassed solvent was passed through two columns of alumina.

For all photochemical reactions, solvents were degassed by evacuating under vacuum and re-filling with N_2. The three lamp flow reactor has been described previously by us[1] and was constructed by wrapping FEP tubing (2.7 mm internal diameter, 3.1 mm external diameter) around a 360 mm length of quartz tube (44 mm internal diameter, 48 mm external diameter), which was capped at both ends with PTFE discs to act as a convenient stand and platform guides for the FEP tubing. Into this was inserted a 36 W single ended PL-L lamp at 365 nm (Philips TUV PL-L 36W UVC germicidal). Three reactors were connected together in series via FEP tubing and the reactor was wrapped in aluminium foil to reflect back light. The reaction solution was pumped through the reactor using a valveless piston pump at controlled flow rates. (Figure 1. Experimental set-up).

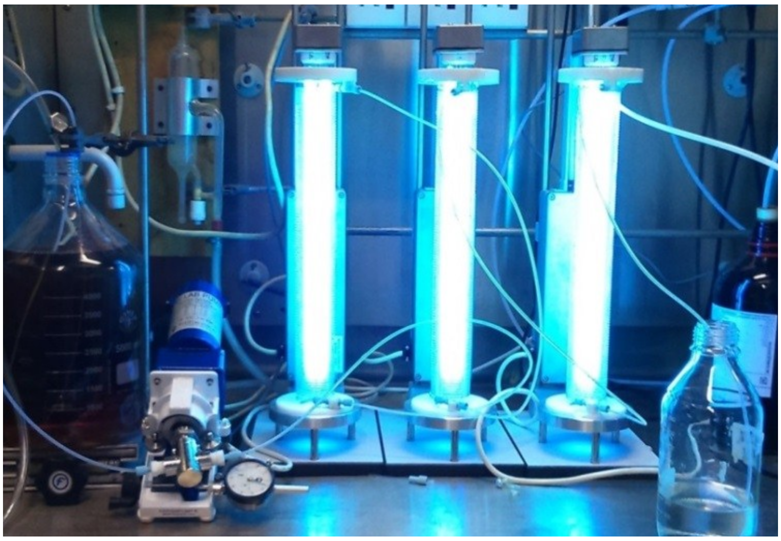

Figure 1. FEP flow photochemical reactor

Scale-up of Photochemical Aziridine Synthesis

(±)-(3*R*,3a*R*,6a*S*)-*N*-ethyl-1,3a,6,6a-tetrahydroazirino[2,3,1-hi]indole-31(2*H*)-carboxamide 3a

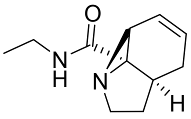

1-(But-3-en-1-yl)-*N*-ethyl-1H-pyrrole-2-carboxamide (7.00 g, 36.4 mmol) was dissolved in degassed acetonitrile (1820 mL) and the solution irradiated with 3 x 36 W UVC lamps in the FEP flow-reactor - at a flow rate of 5.6 mL/min. The reaction mixture was concentrated *in vacuo* to give crude product. Purification by column chromatography (90% EtOAc/hexane, to 100% EtOAc) afforded the title compound **3a** (3.68 g, 53%) as a yellow oil. Analytical data agrees with literature.[2]

(±)-1-((3*R*,3a*R*,6a*S*)-1,3a,6,6a-tetrahydroazirino[2,3,1-hi]indol-31(2*H*)-yl)ethan-1-one 3b

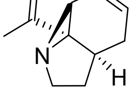

1-(1-(but-3-en-1-yl)-1*H*-pyrrol-2-yl)ethan-1-one (6.00 g, 36.8 mmol) was dissolved in degassed acetonitrile (1840 mL) and the solution irradiated with 3 x 36 W UVC lamps in the FEP flow-reactor - at a flow rate of 4.0 mL/min. The reaction mixture was concentrated *in vacuo* to give crude product. Purification by column chromatography (90% EtOAc/hexane, to 100% EtOAc) afforded the title compound **3b** (2.53 g, 44%) as a brown oil. Analytical data agrees with literature.[2]

(±)-(3*R*,3a*R*,6a*S*)-1,3a,6,6a-tetrahydroazirino[2,3,1-hi]indole-31(2*H*)-carbonitrile 3c

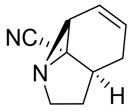

1-(but-3-en-1-yl)-1*H*-pyrrole-2-carbonitrile (4.27 g, 29.2 mmol) was dissolved in degassed acetonitrile (3023 mL) and the solution irradiated with 3 x 36 W UVC lamps in the FEP flow-reactor - at a flow rate of 7.0 mL/min. The reaction mixture was concentrated *in vacuo* to give crude product. Purification by column chromatography (90% Pet. Ether/EtOAc, to 70% Pet. Ether/EtOAc) afforded the title compound **3c** (1.40 g, 33%) as a yellow oil. Analytical data agrees with literature.[2]

Nucleophilic ring opening.

(±)-(3a*S*,7*R*,7a*S*)-*N*-ethyl-7-(phenylthio)-1,2,3,3a,4,7-hexahydro-7a*H*-indole-7a-carboxamide 4

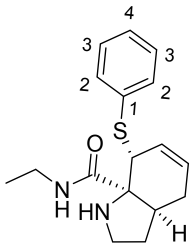

Thiophenol (45 µl, 0.44 mmol) was added to a stirred solution of **3a** (50 mg, 0.26 mmol) in anhydrous MeCN (5 ml) and stirred at room temperature for 48 h under an inert atmosphere. The solvent was removed *in vacuo* and the residue was chromatographed on silica gel eluting with a gradient of 0% to 10% MeOH/EtOAc to afford the title compound **4** (65 mg, 83%) as a yellow oil. ν_{max}/cm^{-1} (film); 3353 (w, N-H), 2877 (w, C-H), 2933 (w, C-H), 2968 (w, C-H), 1661 (s, C=O), 1582 (m, C=C); ^{1}H NMR (400 MHz, CDCl$_3$) δ_H 1.14 (t, *J* = 7.3 Hz, 3H, NHCH$_2$C*H*$_3$), 1.57 – 1.74 (m, 1H, NHCH$_2$C*H*$_2$), 1.91 – 2.11 (m, 2H, NHCH$_2$C*H*$_2$,CH=CH-C*H*$_2$), 2.39 (dtd, *J* = 10.2, 7.1, 3.2 Hz, 1H, CH$_2$C*H*CH$_2$), 2.45 – 2.57 (m, 1H, CH=CHC*H*$_2$), 2.87 – 3.14 (m, 2H, NHC*H*$_2$CH$_2$), 3.14 – 3.41 (m, 2H, NHC*H*$_2$CH$_3$), 3.62 (app p, *J* = 2.7 Hz, 1H, SC*H*CH=CH), 5.79 (dt, *J* = 10.0, 3.4 Hz, 1H, SCHCH=C*H*), 5.93 (dq, *J* = 10.0, 2.2 Hz, 1H, SCHC*H*=CH), 7.17 – 7.25 (m, 1H, Ar-*H*), 7.26 – 7.34 (m, 2H, Ar-*H*), 7.37 – 7.49 (m, 3H, 2 x Ar-*H*, NH); ^{13}C NMR (101 MHz, CDCl$_3$) δ_C 14.9 (NHCH$_2$CH$_3$), 27.0 (CH=CHCH$_2$), 31.9 (NHCH$_2$CH$_2$), 33.7 (NHCH$_2$CH$_3$), 41.6 (CH$_2$CHCH$_2$), 43.1 (NHCH$_2$CH$_2$), 51.7 (SCH), 69.8 (*C*q), 126.5 (SCHCH=*C*H), 126.8 (Ar-*C*4), 128.1 (SCH*C*H=C*H*), 129.0 (Ar-*C*3), 131.2 (Ar-*C*2), 136.9 (Ar-*C*1), 173.1 (*C*=O); HRMS (ESI$^+$) 302.1458 ($C_{17}H_{22}N_2OS^+$, M$^+$, requires 302.1453). ^{1}H NOE showed no enhancement signal between SC*H*CH=CH and CH$_2$C*H*CH$_2$ and a positive interaction between CH$_2$C*H*CH$_2$ and Ph, consistent with *syn*-stereochemistry of the title compound.

Tsuji-Trost reactions.

(±)-(3a*R*,5*S*,7a*R*)-*N*-ethyl-5-phenoxy-1,2,3,3a,4,5-hexahydro-7aH-indole-7a-carboxamide (Table 1, entry 1) and (±) -(3a*R*,5*R*,7a*R*)-*N*-ethyl-5-phenoxy-1,2,3,3a,4,5-hexahydro-7aH-indole-7a-carboxamide (Table 1, entry 2)

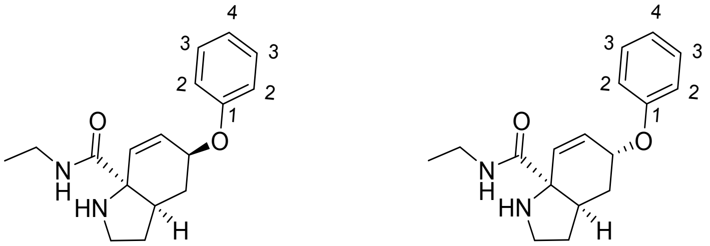

Method 1

$Pd(PPh_3)_4$ (150 mg, 0.13 mmol) was added to a stirred solution of **3a** in dioxane (15 mL). Phenol (210 mg, 2.2 mmol) was added and stirred for 15 h, evaporated, and the residue was chromatographed on silica gel eluting with a gradient of 1:9:90 NH_3/Et_2O/DCM to afford (±)-(3a*R*,5*S*,7a*R*) *N*-ethyl-5-phenoxy-1,2,3,3a,4,5-hexahydro-7a*H*-indole-7a-carboxamide (180 mg, 49%) as a clear oil. ν_{max}/cm^{-1} (film); 3323 (w, N-H), 2934 (w, C-H), 2871 (w, C-H), 1658 (s, C=O); ^1H NMR (400 MHz, $CDCl_3$) δ_H 1.14 (t, *J* = 7.3 Hz, 3H, $NHCH_2CH_3$), 1.48 – 1.52 (m, 1H, PhOCHC*H*$_2$), 1.60 (dddd, *J* = 12.7, 7.4, 4.2, 2.8 Hz, 1H, $NHCH_2CH_2$), 1.79 (s, 1H, N*H*CH_2CH_2), 1.97 (dtd, *J* = 12.7, 8.4, 7.4 Hz, 1H, $NHCH_2CH_2$), 2.17 (dtd, *J* = 12.7, 4.9, 1.2 Hz, 1H, PhOCHC*H*$_2$), 2.79 – 2.96 (m, 2H, NHC*H*$_2$CH$_2$, CH$_2$C*H*CH$_2$), 3.18 (dt, *J* = 10.8, 7.9 Hz, 1H, NHC*H*$_2$CH$_2$), 3.22 – 3.31 (m, 2H, NHC*H*$_2$CH$_3$), 5.02 (ddt, *J* = 10.0, 5.0, 1.9 Hz, 1H, PhOC*H*), 5.63 (dd, *J* = 10.0, 1.9 Hz, 1H, CH-CH=C*H*), 6.10 (dt, *J* = 10.0, 1.4 Hz, 1H, CH-C*H*=CH), 6.76 – 6.80 (m, 3H, Ar-*H*), 7.23 – 7.30 (m, 2H, Ar-*H*), 7.82 – 8.33 (m, 1H, N*H*CH_2CH_3); ^{13}C NMR (101 MHz, $CDCl_3$) δ_C 14.9 (NHCH$_2$*C*H$_3$), 31.9 (PhOCH*C*H$_2$), 32.1 (HNCH$_2$*C*H$_2$), 34.2 (NH*C*H$_2$CH$_3$), 38.3 (COCq*C*H), 45.2 (HN*C*H$_2$CH$_2$), 69.1 (*C*q), 71.8 (PhO*C*HCH$_2$), 115.7 (Ar *C*-2), 121.0 (Ar *C*-4), 129.1 (CH-*C*H=CH), 129.6 (Ar *C*-3), 132.7 (CH-CH=*C*H), 157.3 (Ar *C*-1), 174.7 (*C*=O); HRMS (ESI$^+$) 287.1753 ($C_{17}H_{23}N_2O_2^+$, $[M+H]^+$, requires 287.1754). ^1H NOE enhancement signal between PhOC*H* and CH$_2$C*H*CH$_2$ confirmed the *anti*-stereochemistry conformation.

Method 2

$Pd(PPh_3)_4$ (75 mg, 0.07 mmol) was added to a stirred solution of **3a** (250 mg, 1.3 mmol) in DMF (15 mL). Phenol (210 mg, 2.2 mmol) was added and stirred for 15 h (crude ^1H NMR showed conversion a 21:79 ratio in favour of (±)-(3a*R*,5*R*,7a*R*) *N*-ethyl-5-phenoxy-1,2,3,3a,4,5-hexahydro-7a*H*-indole-7a-carboxamide). The solution was diluted with water (150 ml), extracted into Et_2O (3 x 50 ml), washed with brine (50 ml) and dried over $MgSO_4$. The solvent was removed *in vacuo* and the residue was

chromatographed on silica gel eluting with a gradient of 1:9:90 NH_3/Et_2O/DCM to afford (±)-(3a*R*,5*S*,7a*R*) *N*-ethyl-5-phenoxy-1,2,3,3a,4,5-hexahydro-7a*H*-indole-7a-carboxamide (32 mg, 9%) and (±)-(3a*R*,5*R*,7a*R*) *N*-ethyl-5-phenoxy-1,2,3,3a,4,5-hexahydro-7a*H*-indole-7a-carboxamide (160 mg, 43%) as clear oils. ν_{max}/cm^{-1} (film): 3323 (w, N-H), 2930 (w, C-H) 2871 (w, C-H), 1659 (s, C=O); ^{1}H NMR (400 MHz, $CDCl_3$) δ_H 1.12 (t, *J* = 7.3 Hz, 3H, $NHCH_2CH_3$), 1.57 (dq, *J* = 12.3, 7.0 Hz, 1H, $HNCH_2CH_2$), 1.89 – 2.02 (m, 2H, $HNCH_2CH_2$, $PhOCHCH_2$), 2.20 (ddd, *J* = 13.5, 7.3, 5.0 Hz, 1H, $PhOCHCH_2$), 2.65 (qd, *J* = 7.3, 5.0 Hz, 1H, CH_2CHCH_2), 2.84 – 3.02 (m, 2H, $HNCH_2CH_2$), 3.27 (m, 2H, $NHCH_2CH_3$), 4.77 – 4.86 (m, 1H, $PhOCHCH_2$), 5.67 (dd, *J* = 10.0, 1.3 Hz, 1H, PhOCHCH=C*H*), 6.15 (dd, *J* = 10.0, 3.3 Hz, 1H, PhOCHC*H*=CH), 6.85 – 6.98 (m, 3H, Ar-*H* (C-2 and C-4), 7.22 – 7.29 (m, 2H, Ar-*H* (C3)), 7.29 – 7.36 (m, 1H, $NHCH_2CH_3$); ^{13}C NMR (101 MHz, $CDCl_3$) δ_C 14.9 ($NHCH_2CH_3$), 30.5 ($PhOCHCH_2$), 31.8 ($HNCH_2CH_2$), 34.3 ($NHCH_2CH_3$), 39.4 (CH_2CHCH_2), 45.3 ($HNCH_2CH_2$), 67.0 (*C*q), 68.8 ($PhOCHCH_2$), 115.9 (Ar *C*-2), 121.1 (Ar *C*-4), 129.4 (CH*C*H=CH), 129.5 (Ar *C*-3), 132.1 (CHCH=*C*H), 157.5 (Ar *C*-1), 174.5 (*C*=O); HRMS (ESI$^+$) 287.1762 ($C_{17}H_{23}N_2O_2^+$, $[M+H]^+$, requires 287.1754). No ^{1}H NOE enhancement signal between PhOC*H* and CH_2CHCH_2 consistent with *syn*- stereochemistry conformation.

(±)-(3a*S*,5*S*,7a*R*)-5-(dicyanomethyl)-N-ethyl-1,2,3,3a,4,5-hexahydro-7aH-indole-7a-carboxamide (Table 1, entry 3)

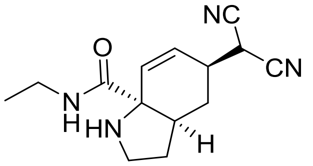

To a stirred solution of **3a** (50 mg, 0.26 mmol) and malononitrile (32 mg, 0.48 mmol) in anhydrous, degassed dioxane (3 mL) was added $Pd(PPh_3)_4$ (15 mg, 0.013 mmol) and the stirred mixture heated to 40 °C under nitrogen. After 14 h the reaction was evaporated to give an orange oil. Purification by silica gel chromatography (EtOAc/petrol, 3:7 to 1:0 as eluent) afforded a yellow solid which was triturated in Et_2O/petrol (4:1, 2 × 2.5 mL) at 0 °C and dried to afford the title compound (35mg, 52%) as a yellow solid. Mp. 145 – 146 °C. ν_{max} /cm^{-1} (film) 3330 (br), 2936, 2256, 1655, 1514 and 1450; δ_H (400 MHz, $CDCl_3$) 1.14 (3H, t, *J* = 7.3, Me), 1.18 – 1.31 (1H, m, homoallylic C*H*H), 1.57 (1H, dddd, *J* = 13.1, 7.5, 3.8, 1.9, NCH_2CHH), 1.96 – 2.08 (2H, m, NCH_2CHH and homoallylic CH*H*), 2.86 (1H, dddd, *J* = 14.0, 7.0, 4.8, 2.0, CH_2CHCH_2), 2.92 (1H, ddd, *J* = 11.0, 8.9, 4.0, $NCHHCH_2$), 3.04 (1H, dt, *J* = 11.8, 4.8, C*H*CH(CN)$_2$), 3.19 (dt, *J* = 11.1, 8.2, $NCHHCH_2$), 3.22 – 3.30 (2H, m, NCH_2Me), 3.90 (1H, d, J 5.3, C*H*(CN)$_2$), 5.85 – 5.91 (2H, m, C*H*=C*H*) and 7.98 (1H, brs, NH); δ_C (101 MHz, $CDCl_3$) 14.9 (Me), 28.1 (*C*H(CN)$_2$), 29.9 (homoallylic CH2), 31.7 (NCH_2CH_2), 34.4 (NCH_2Me), 37.2 (*C*HCH(CN)$_2$), 38.8

(CH_2CHCH_2), 44.6 (NCH_2CH_2), 69.0 (*C*q-CONHEt), 111.4 (CN), 111.5 (CN), 128.5 (alkene), 132.3 (alkene) and 173.8 (*C*ONHEt); HRMS (ESI$^+$) 259.1555 ($C_{14}H_{19}N_4O$, $[M+H]^+$, requires 259.1553). ^1H NOE showed enhancement between allylic CH and CH_2CHCH_2, proving *anti*- stereochemistry of the title compound.

(±)-(3a*S*,5*R*,7a*R*)-5-(dicyanomethyl)-N-ethyl-1,2,3,3a,4,5-hexahydro-7aH-indole-7a-carboxamide (Table 1, entry 4)

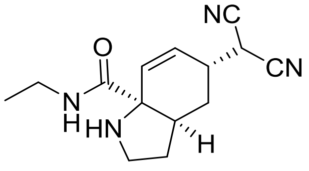

To a stirred solution of **3a** (50 mg, 0.26 mmol) and malononitrile (32 mg, 0.48 mmol) in anhydrous, degassed MeCN (3 mL) was added $Pd(PPh_3)_4$ (15 mg, 0.013 mmol) and the stirred mixture heated to 40 °C under nitrogen. After 14 h the reaction was evaporated to give a brown oil. Purification by silica gel chromatography (EtOAc/petrol, 3:7 to 1:0 followed by EtOAc/EtOH, 95:5 to 9:1 as eluent) afforded (±)-(3a*S*,5*S*,7a*R*)-5-(dicyanomethyl)-N-ethyl-1,2,3,3a,4,5-hexahydro-7aH-indole-7a-carboxamide (18 mg, 27%) as a yellow solid and (±)-(3a*S*,5*R*,7a*R*)-5-(dicyanomethyl)-N-ethyl-1,2,3,3a,4,5-hexahydro-7aH-indole-7a-carboxamide (20 mg, 30%) as a yellow oil. (±)-(3a*S*,5*R*,7a*R*)-5-(dicyanomethyl)-N-ethyl-1,2,3,3a,4,5-hexahydro-7aH-indole-7a-carboxamide: ν_{max} /cm^{-1} (film) 3322 (br), 2933, 2254, 1646, 1516 and 1449; δ_H (400 MHz, $CDCl_3$) 1.14 (1H, t, *J* 7.3, Me), 1.43 – 1.52 (1H, m, NCH_2CHH), 1.68 (1H, ddd, *J* 14.3, 10.0, 4.6, homoallylic CH*H*), 1.90 (1H, brs, NH), 2.08 (1H, dtd, *J* 12.8, 7.3, 5.2, NCH_2CHH), 2.18 (1H, dt, *J* 14.0, 5.4, homoallylic CHH), 2.63 (1H, p, *J* 7.0, CH_2CHCH_2), 2.78 – 2.89 (2H, m, $NCHHCH_2$ and $CHCH(CN)_2$), 3.08 (1H, ddd, *J* = 11.7, 7.4, 5.3, $NCHHCH_2$)3.21 – 3.29 (2H, m, NCH_2Me), 4.64 (1H, d, J 10.1, $CH(CN)_2$), 5.79 (1H, d, J 10.0, C*H*=CH-CH), 6.12 (1H, dd, J 9.9, 5.1, CH=C*H*-CH) and 7.84 (1H, brs, NH); δ_C (101 MHz, $CDCl_3$) 14.8 (Me), 27.1 ($CH(CN)_2$), 28.8 (homoallylic CH_2), 32.8 (NCH_2CH_2), 34.4 (NCH_2Me), 35.1 ($CHCH(CN)_2$), 37.6 (CH_2CHCH_2), 45.1 (NCH_2CH_2), 68.1 (*C*q-CONHEt), 112.3 (CN), 112.6 (CN), 127.7 (CH=*C*H-CH), 133.2 (*C*H=CH-CH) and 174.2 (*C*ONHEt); HRMS (ESI$^+$) 259.1553 ($C_{14}H_{19}N_4O$, $[M+H]^+$, requires 259.1553). ^1H NOE showed no enhancement signal between allylic CH and CH_2CHCH_2 (^1H TOCSY used to identify peak shapes of overlapping muliplet), consistent with *syn*- stereochemistry of the title compound.

(±)-(3a*S*,5*S*,7a*R*)-5-(2,4-dioxopentan-3-yl)-N-ethyl-1,2,3,3a,4,5-hexahydro-7aH-indole-7a-carboxamide (Table 1, entry 5)

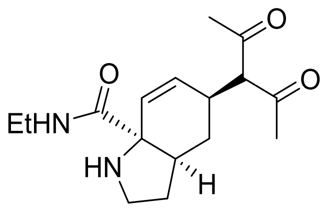

To a solution of **3a** (50 mg, 0.26 mmol) in anhydrous dioxane (3 mL) was added acetylacetone (0.10 mL, 0.97 mmol) and the mixture degassed using the freeze-pump-thaw method. $Pd(PPh_3)_4$ (10 mg, 8.7 µmol) was added and the stirred mixture was heated to 80 °C, stirred for 18 h, cooled to rt and evaporated. Purification by silica gel chromatography (EtOAc/MeOH, 1:0 to 4:1 as eluent) afforded the title compound (62 mg, 82%) as a yellow oil. ν_{max}/cm^{-1} (film) 3328, 2934, 1722, 1697, 1656, 1586, 1509 and 1357; δ_H (400 MHz, $CDCl_3$) 0.87 (1H, app q, J 12.7, homoallylic CH*H*), 1.09 (3H, t, J 7.3, CH_2*Me*), 1.43 (1H, dddd, J 13.0, 7.7, 3.5, 1.5, NCH_2CH*H*), 1.57 (1H, dt, J 12.6, 4.5, homoallylic C*H*H), 1.84 – 1.95 (1H, m, NCH2C*H*H), 2.156 (3H, s, CO*Me*), 2.163 (3H, s, CO*Me*), 2.71 – 2.79 (1H, m, CH_2C*H*CH_2), 2.84 (1H, ddd, J 10.9, 9.1, 3.7, NCH*H*), 3.05 (1H, dt, J 10.9, 8.3, NC*H*H), 3.13 (1H, app td, J 10.7, 4.2, C*H*-CH=), 3.18 – 3.26 (2H, m, NC*H*$_2$Me), 3.52 (1H, d, J 9.9, C*H*$(COMe)_2$), 5.57 – 5.63 (2H, m, C*H*=C*H*), 7.87 (1H, brm, NH); δ_C (101 MHz, $CDCl_3$) 15.0 (NCH_2*Me*), 29.5 (CO*Me*), 30.1 (CO*Me*), 30.7 (homoallylic *C*H_2), 31.9 (NCH_2*C*H_2), 34.3 (N*C*H_2Me), 35.7 (=CH-*C*H), 39.4 (CH_2*C*HCH_2), 44.6 (N*C*H_2), 69.3 (*C*q-CONHEt), 73.9 (*C*H$(COMe)_2$), 129.1 (*C*H=CH-CH), 132.4 (CH=*C*H-CH), 174.7 (*C*ONHEt), 203.6 (*C*OMe) and 203.7 (*C*OMe); HRMS (CI^+) 293.1860 ($C_{16}H_{25}N_2O_3$, $[M+H]^+$, requires 293.1865). ^{1}H NOESY analysis showed no interaction between CH2CHCH2 and allylic CH, consistent with assigned stereochemistry.

(±)-(3a*S*,5*R*,7a*R*)-5-(2,4-dioxopentan-3-yl)-N-ethyl-1,2,3,3a,4,5-hexahydro-7aH-indole-7a-carboxamide (Table 1, entry 6)

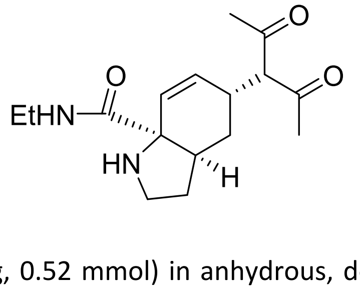

To a stirred solution of **3a** (100 mg, 0.52 mmol) in anhydrous, degassed MeCN (5 mL) were added acetylacetone (0.20 mL, 1.9 mmol) and $Pd(PPh_3)_4$ (18 mg, 16 µmol) and the mixture heated to 80 °C under nitrogen. After 15 h the mixture was cooled to rt and evaporated to give a yellow oil.

Purification by silica gel chromatography (EtOAc/petrol, 3:7 to 1:0 then EtOAc/MeOH, 4:1 as eluent) afforded the title compound (102 mg, 67%) as a yellow wax. ν_{max} /cm^{-1} (film) 3334, 2934, 1722, 1697, 1657, 1511, 1449, 1419, 1357 and 1267; δ_H (400 MHz, $CDCl_3$) 1.11 (3H, t, J 7.3, NCH_2*Me*), 1.43 – 1.55 (2H, m, homoallylic CH*H* and NCH_2CH*H*), 1.65 (1H, ddd, J 13.5, 6.5, 5.0, homoallylic C*H*H), 1.83 – 1.93 (1H, m, NCH_2C*H*H), 2.20 (3H, s, CO*Me*), 2.22 (3H, s, CO*Me*), 2.33 (1H, brs, NH), 2.45 – 2.53 (1H, m, CH_2C*H*CH_2), 2.82 (1H, dt, J 10.6, 7.3, NCH*H*), 2.93 – 3.00 (1H, m, =CH-C*H*), 3.02 (1H, ddd, J 13.5, 7.7, 5.6, NC*H*H), 3.18 – 3.26 (2H, m, NCH_2Me), 4.09 (1H, d, J 9.9, C*H*$(COMe)_2$), 5.50 (1H, dd, J 10.0, 1.8, C*H*=CH-CH), 5.73 (1H, dd, J 10.0, 4.1, CH=C*H*-CH) and 7.85 (1H, brs, NH); δ_C (101 MHz, $CDCl_3$) 14.8 (NCH_2*Me*), 28.1 (homoallylic *C*H2), 30.2 (Me), 31.3 (Me), 31.4 (NCH_2*C*H_2), 32.6 (=CH-*C*H), 34.2 (N*C*H_2Me), 38.6 (CH_2*C*HCH_2), 44.7 (N*C*H_2CH_2), 67.6 (*C*q-CONHEt), 71.6 (*C*H$(COMe)_2$), 129.8 (*C*H=CH-CH), 130.7 (CH=*C*H-CH), 175.8 (*C*ONHEt), 203.0 (*C*=O) and 203.8 (*C*=O); HRMS (CI^+) 293.1859 ($C_{16}H_{25}N_2O_3$, $[M+H]^+$, requires 293.1865). ^{1}H NOESY analysis showed an interaction between CH2CHCH2 and allylic CH, confirming stereochemistry shown.

(±)-(3a*S*,5*S*,7a*R*)-5-(4,4-dimethyl-2,6-dioxocyclohexyl)-N-ethyl-1,2,3,3a,4,5-hexahydro-7aH-indole-7a-carboxamide (Table 1, entry 7)

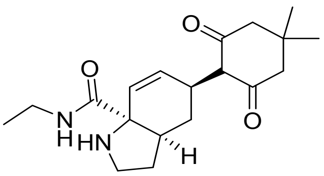

$Pd(PPh_3)_4$ (15 mg, 0.01 mmol, 0.05 eq.) was added to a stirred solution of **3a** (50 mg, 0.26 mmol) in degassed anhydrous dioxane (5 mL). 5,5-dimethylcyclohexane-1,3-dione (62 mg, 44 mmol) was added and the solution was stirred for 15 h at rt. The reaction was evaporated and the residue was chromatographed on silica gel eluting with a gradient of 0% to 20% MeOH/EtOAc to afford the title compound (65 mg, 75%) as a yellow oil. ν_{max}/cm^{-1} (film); 3313 (w, N-H), 2956 (m, C-H), 2871 (m, C-H), 1651 (s, C=O), 1600 (s, C=O) 1215 (s); ^{1}H NMR (400 MHz, $CDCl_3$) δ_H 1.05 (s, 6H, C(C*H*$_3)_2$), 1.11 (t, *J* = 7.3 Hz, 3H, $NHCH_2$C*H*$_3$), 1.46 (td, *J* = 12.4, 9.3 Hz, 1H, CHC*H*$_2$CH), 1.52 – 1.64 (m, 1H, $HNCH_2$C*H*$_2$), 1.95 (dd, *J* = 12.9, 7.8 Hz, 1H, $HNCH_2$C*H*$_2$), 2.13 – 2.19 (m, 1H, CHC*H*$_2$CH), 2.19 (s, 2H, C*H*$_2C(CH_3)_2$), 2.23 (s, 2H, C*H*$_2C(CH_3)_2$), 2.69 – 2.80 (m, 1H, CH_2C*H*CH_2), 2.88 (ddd, *J* = 10.8, 8.3, 4.6 Hz, 1H, NHC*H*$_2CH_2$), 3.14 (dt, *J* = 10.8, 7.8 Hz, 1H, NHC*H*$_2CH_2$), 3.24 (m, 2H, NHC*H*$_2CH_3$), 4.87 (ddt, *J* = 9.2, 4.6, 1.9 Hz, 1H, HC=CHC*H*CH_2), 5.40 (s, CHC*H*$(C=O)_2$, 1H), 5.63 (dd, *J* = 10.0, 1.9 Hz, 1H, *H*C=CHCH), 5.96 (ddd, *J* = 10.0, 1.9, 1.0 Hz, 1H, HC=C*H*-CH), 7.86 (s, 1H, N*H*CH_2CH_3); ^{13}C NMR (101 MHz, $CDCl_3$) δ_C 15.0 ($NHCH_2$*C*H_3), 28.35 (C(*C*$H_3)_2$), 28.37 (C(*C*$H_3)_2$), 30.8 (CH*C*H_2CH), 32.1 ($NHCH_2$*C*H_2), 32.6 (CH_2*C*$(CH_3)_2$), 34.3 (NH*C*H_2CH_3), 38.3 (CH_2*C*HCH_2), 43.3 (*C*$H_2C(CH_3)_2$), 45.3 (HN*C*H_2CH_2), 50.7 (*C*$H_2C(CH_3)_2$), 68.7 (*C*q),

72.5 (HC=CHCHCH$_2$), 102.5 (CHCH(CO)$_2$), 130.3 (HC=CHCHCH$_2$), 130.6 (HC=CHCHCH$_2$), 174.3 (CO), 174.6 (Amide CO), 199.5 (CO); HRMS (CI$^+$) 333.2178 ($C_{19}H_{29}N_2O_3^+$, [M+H]$^+$, requires 333.2183). ^{1}H NOE enhancement signal between CH$_2$C*H*CH$_2$ and HC=CH-C*H* confirmed the *anti*- stereochemistry conformation.

(±)-(3a*S*,5*S*,7a*R*)-5-(1,3-dioxo-2,3-dihydro-1H-inden-2-yl)-N-ethyl-1,2,3,3a,4,5-hexahydro-7aH-indole-7a-carboxamide (Table 1, entry 8)

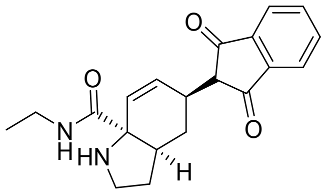

Pd(PPh$_3$)$_4$ (30 mg, 0.026 mmol) was added to a solution of **3a** (100 mg, 0.52 mmol) in degassed anhydrous dioxane (5 mL). 1*H*-indene-1,3(2*H*)-dione (130 mg, 0.88 mmol) was added and stirred for 15 h at room temperature. The reaction was evaporated and the residue chromatographed on silica gel (8:1 EtOH/NH3 in DCM, 2% to 12% as eluent) to afford the title compound (112 mg, 64%) as a red solid. Mp. 192-193°C; ν_{max}/cm^{-1} (solid); 3287 (m, N-H), 2967 (w, C-H), 1676 (m, C=O), 1512 (s) 1488 (s); ^{1}H NMR (400 MHz, CDCl$_3$) δ_H 1.11 (3H, t, J 7.3, NCH$_2$*Me*), 1.30 (1H, app. q, J 12.6, CHCH*H*CH), 1.38 – 1.45 (1H, m, NCH$_2$CH*H*), 1.58 (1H, dtd, J 12.5, 4.6, 1.2, CHC*H*HCH), 1.87 (1H, dtd, J 13.0, 9.0, 7.2, NCH$_2$C*H*H), 1.95 (1H, brs, NH), 2.74 – 2.80 (1H, m, CH$_2$C*H*CH$_2$), 2.82 (1H, ddd, J 11.0, 9.3, 3.6, NCH*H*), 3.08 (1H, td, J 11.0, 8.3, NC*H*H), 3.09 (1H, d, J 4.2, C*H*(C=O)$_2$), 3.21 – 3.28 (3H, NC*H*$_2$Me and allylic C*H*), 5.64 (1H, dd, J 9.9, 2.8, =C*H*-CH), 5.86 (1H, app. d, J 9.9, C*H*=CH-CH), 7.83 – 7.87 (2H, m, 2 × CH$_{Ar}$) and 7.93 – 7.99 (3H, m, 2 × CH$_{Ar}$ and NH); ^{13}C NMR (101 MHz, CDCl$_3$) δ_C 14.9 (CH$_2$*Me*), 29.4 (CH*C*H$_2$CH), 31.6 (NCH$_2$*C*H$_2$), 34.2 (N*C*H$_2$Me), 35.9 (allylic CH), 39.3 (CH$_2$*C*HCH$_2$), 44.4 (N*C*H$_2$CH$_2$), 56.6 (*C*H(C=O)$_2$), 69.2 (*C*q-CONHEt), 123.07 (CH$_{Ar}$), 123.09 (CH$_{Ar}$), 128.7 (HC=*C*H-CH), 132.7 (H*C*=CH-CH), 135.8 (CH$_{Ar}$), 135.8 (CH$_{Ar}$), 142.7 (Cq$_{Ar}$), 142.8 (Cq$_{Ar}$), 174.8 (amide), 199.8 (ketone) and 199.9 (ketone); HRMS (ESI$^+$) 339.1709 ($C_{20}H_{23}N_2O_3^+$, [M+H]$^+$, requires 339.1703). ^{1}H NOE enhancement signal between CH$_2$C*H*CH$_2$ and HC=CH-C*H* confirmed the *anti*- stereochemistry conformation.

(±)-2-((3a*S*,5*S*,7a*R*)-7a-acetyl-2,3,3a,4,5,7a-hexahydro-1H-indol-5-yl)-5,5-dimethylcyclohexane-1,3-dione (Table 1, entry 9)

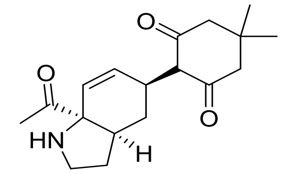

$Pd(PPh_3)_4$ (18 mg, 0.02 mmol, 0.05 eq.) was added to a solution of **3b** (50 mg, 0.31 mmol) in dioxane (5 mL). 5,5-dimethylcyclohexane-1,3-dione (73 mg, 0.52 mmol) was added and stirred for 15 h at room temperature. The reaction was evaporated and the residue was chromatographed on silica gel eluting with a gradient of 0% to 20% MeOH/EtOAc to afford the title compound (48 mg, 52%) as a yellow oil. ν_{max}/cm^{-1} (film); 2941 (w, N-H) 2870 (w, C-H) 1707 (m, C=O) 1649 (m, C=O) 1598 (m); ^1H NMR (400 MHz, $CDCl_3$) δ_H 1.05 (s, 6H, (C(C*H*$_3$)$_2$), 1.53 – 1.66 (m, 2H, CHC*H*$_2$CH, HNCH$_2$C*H*$_2$), 1.84 (dd, *J* = 12.6, 8.1 Hz, 1H, HNCH$_2$C*H*$_2$), 2.11 – 2.17 (m, 1H, CHC*H*$_2$CH), 2.17 (s, 3H, C*H*$_3$CO), 2.19 (s, 2H, C*H*$_2$C(CH$_3$)$_2$), 2.25 (s, 2H, C*H*$_2$C(CH$_3$)$_2$), 2.51 (dddd, *J* = 11.5, 8.0, 5.1, 3.0 Hz, 1H, CH$_2$C*H*CH$_2$), 2.76 (brs, 1H, *H*NCH$_2$CH$_2$), 2.89 – 3.11 (m, 2H, HNC*H*$_2$CH$_2$), 4.68 – 4.75 (m, 1H, HC=CHC*H*), 5.36 (s, 1H, CHC*H*(CO)$_2$), 5.66 (dd, *J* = 10.1, 2.0 Hz, 1H, *H*C=CHCH), 6.00 (ddd, *J* = 10.1, 2.0, 1.1 Hz, 1H, HC=C*H*-CH); ^{13}C NMR (101 MHz, $CDCl_3$) δ_C 25.2 (*C*H$_3$CO), 28.29 (C(*C*H$_3$)$_2$), 28.34 (C(*C*H$_3$)$_2$), 31.2 (CH*C*H$_2$CH), 32.6 (C(*C*H$_3$)$_2$), 32.8 (HNCH$_2$*C*H$_2$), 37.1 (CH$_2$*C*HCH$_2$), 43.2 (*C*H$_2$CO), 45.7 (HN*C*H$_2$CH$_2$), 50.7 (*C*H$_2$CO), 72.1 (HC=CH-C*H*), 73.6 (*C*q), 102.5 (CH*C*H(CO)$_2$), 130.5 (HC=*C*H-CH), 130.6 (H*C*=CH-CH), 174.7 (CH$_2$*C*O), 199.4 (CH$_2$*C*O), 209.1 (CH$_3$*C*O); HRMS (ESI$^+$) 304.1911 ($C_{18}H_{26}NO_3^+$, $[M+H]^+$, requires 304.1907). ^1H NOE enhancement signal between CH$_2$C*H*CH$_2$ and HC=CH-C*H* confirmed the *anti*- stereochemistry conformation.

(±)-2-((3a*S*,5*S*,7a*R*)-7a-acetyl-2,3,3a,4,5,7a-hexahydro-1H-indol-5-yl)-1H-indene-1,3(2H)-dione (Table 1, entry 10)

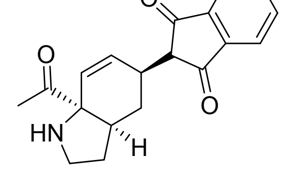

$Pd(PPh_3)_4$ (18 mg, 0.02 mmol) was added to a solution of **3a** (50 mg, 0.31 mmol) in dioxane (5 mL). 1*H*-indene-1,3(2*H*)-dione (76 mg, 0.52 mmol) was added and the solution was stirred for 15 h at room temperature. The reaction was evaporated and the residue was chromatographed on silica gel eluting with a gradient of 1:2:97 to 1:10:89 NH_3/MeOH/DCM to afford the title compound (30 mg, 32%) as an orange oil (5:1 mix of inseparable diastereomers). ν_{max}/cm^{-1} (film); 3356 (b w, N-H), 1713

(m, C=O), 1608 (w, C=O), 1513 (br s); ^{1}H NMR (400 MHz, $CDCl_3$) δ_H 1.39 – 1.44 (m, 2H, NCH_2CH_2, $CHCH_2CH$), 1.68 (dt, *J* = 12.1, 4.4 Hz, 1H, $CHCH_2CH$), 1.78 (dq, *J* = 12.5, 8.4 Hz, 1H, NCH_2CH_2), 2.19 (s, 3H, CH_3CO), 2.49 (app q, *J* = 7.0 Hz, 1H, CH_2CHCH_2), 2.67 (s, 1H, N*H*), 2.81 – 3.02 (m, 2H, NCH_2CH_2), 3.02 – 3.14 (m, 2H, $(CO)_2CHCH$, CH=CHC*H*), 5.70 (dd, *J* = 10.0, 2.4 Hz, 1H, CH=C*H*CH), 5.99 (d, *J* = 10.0 Hz, 1H, C*H*=CHCH), 7.87 (ddd, *J* = 7.3, 5.7, 3.2 Hz, 2H, Ar-*H* (C-2)), 7.97 (td, *J* = 5.4, 2.8 Hz, 2H, Ar-*H* (C-1)); ^{13}C NMR (101 MHz, $CDCl_3$) δ_C 24.9 (CH_3CO), 30.0 ($CHCH_2CH$), 32.5 (NCH_2CH_2), 35.9 ($(CO)_2CHCH$), 39.1 (CH=CHC*H*), 44.9 (NCH_2CH_2), 56.3 ($(CO)_2CHCH$), 74.0 (*C*q), 123.2 (Ar *C*-1), 128.8 (CH=*C*HCH), 132.8 (*C*H=CHCH), 135.9 (Ar *C*-2), 142.7 (Ar *C*), 142.8 (Ar *C*), 199.6 (*C*=O), 199.7 (*C*=O), 209.9 (CH_3CO); HRMS (ESI$^+$) 310.1434 ($C_{19}H_{20}NO_3^+$, $[M+H]^+$, requires 310.1438). ^{1}H NOE enhancement between CH_2CHCH_2 and HC=CH-C*H* confirmed the *anti*- stereochemistry conformation.

Isocyanate reactions.

(±)-(31*R*,5a*S*,8a*S*)-N-ethyl-2-oxo-1-tosyl-1,4,5,5a,6,8a-hexahydroimidazo[4,5,1-hi]indole-31(2H)-carboxamide (Table 2, entry 1)

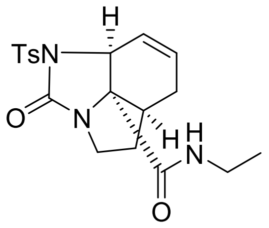

To a stirred solution of **3a** (50 mg, 0.26 mmol) in degassed, anhydrous dioxane (3 mL) was added $Pd(PPh_3)_4$ (15 mg, 0.013 mmol) and tosyl isocyanate (55 µL, 0.36 mmol) and the reaction stirred at rt under nitrogen. After 20 h the reaction was evaporated to give a brown oil. Purification by silica gel chromatography (EtOAc/petrol, 3:7 to 1:0 as eluent) afforded the title compound (84 mg, 83%) as a light yellow solid. Mpt = 169 – 170 °C. ν_{max} /cm^{-1} (film) 3346 (br), 2973, 1731, 1664, 1525, 1356 and 1166; δ_H (500 MHz, $CDCl_3$) 1.09 (3H, t, J 7.3, NCH_2*Me*), 1.64 – 1.74 (2H, m, allylic CH*H* and NCH_2CHH), 1.93 – 2.01 (1H, m, NCH_2CHH), 2.29 (1H, dt, J 16.5, 5.6, allylic C*H*H), 2.43 (3H, s, Me), 2.63 – 2.69 (1H, m, CH_2CHCH_2), 3.13 – 3.31 (3H, m, $NCHHCH_2$ and NCH_2Me), 3.63 (1H, dt, J 11.5, 7.3, $NCHHCH_2$), 4.79 (1H, d, J 3.6, NCH), 6.10 – 6.17 (2H, m, C*H*=C*H*), 6.46 (1H, brt, J 4.8, NH), 7.31 (2H, d, J 8.4, 2 × CH_{Ar}) and 7.90 (2H, app. d, J 8.4, 2 × CH_{Ar}); δ_C (126 MHz, $CDCl_3$) 14.7 (NCH_2*Me*), 21.6 (Me), 24.7 (allylic CH_2), 31.7 (NCH_2CH_2), 34.5 (NCH_2Me), 39.5 (CH_2CHCH_2), 45.7 (NCH_2CH_2), 56.2 (N*C*H), 70.0 (*C*q-CONHEt), 125.4 (*C*H=CH), 128.1 (2 × CH_{Ar}), 129.6 (2 × CH_{Ar}), 131.4 (CH=*C*H), 136.2 (Cq_{Ar}), 144.8 (Cq_{Ar}), 155.7 (urea) and 172.2 (CONHEt); HRMS (CI$^+$) 390.1490 ($C_{19}H_{24}N_3O_4S$, $[M+H]^+$, requires 390.1488)

(±)-(31*R*,8a*S*)-2-oxo-1-tosyl-1,4,5,5a,6,8a-hexahydroimidazo[4,5,1-hi]indole-31(2H)-carbonitrile (Table 2, entry 2)

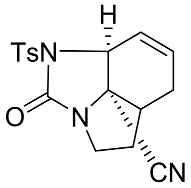

Tosyl isocyanate (0.065 mL, 0.42 mmol) was added dropwise to a stirred solution of **3c** (50 mg, 0.34 mmol) and $Pd(PPh_3)_4$ (15 mg, 0.013 mmol) in anhydrous, degassed dioxane (3 mL) at rt under nitrogen. After 15 h the reaction was evaporated and purified by silica gel chromatography (EtOAc/petrol, 1:4 to 2:3 as eluent) afforded the title compound (94 mg, 81%) as a white solid. Mp 194 – 195 °C. ν_{max} /cm^{-1} (film) 2970, 1735, 1596, 1353 and 1166; δ_H (400 MHz, $CDCl_3$) 1.57 (1H, dd, J 16.1, 9.8, allylic CH*H*), 1.86 – 1.94 (1H, m, NCH_2CH*H*), 2.17 – 2.26 (1H, m, allylic C*H*H), 2.34 – 2.44 (4H, m, Me and NCH_2C*H*H), 2.53 – 2.60 (1H, m, CH_2C*H*CH_2), 3.32 (1H, ddd, J 12.4, 9.0, 3.5, NCH*H*), 3.55 (1H, dt, J 11.4, 8.1, NC*H*H), 5.07 (1H, t, J 2.1, C*H*-CH=), 6.14 – 6.21 (2H, m, C*H*=C*H*), 7.30 (2H, d, J 8.2, ArH) and 7.87 (2H, d, J 8.4, ArH); δ_C (101 MHz, $CDCl_3$) 21.7 (Me), 24.0 (allylic CH2), 31.7 (NCH_2*C*H_2), 42.3 (CH_2*C*HCH_2), 43.8 (NCH_2), 55.6 (NCH), 61.0 (*C*q-CN), 119.4 (CN), 124.4 (=CH), 128.1 (2 × CH_{Ar}), 129.7 (2 × CH_{Ar}), 132.5 (=CH), 135.5 (Cq), 145.2 (Cq) and 152.7 (urea); HRMS (ESI$^+$) 344.1067 ($C_{17}H_{18}N_3O_3S$, $[M+H]^+$, requires 344.1063).

(±)-(31*R*,5a*S*,8a*S*)-31-acetyl-1-tosyl-31,4,5,5a,6,8a-hexahydroimidazo[4,5,1-hi]indol-2(1H)-one (Table 2, entry 3)

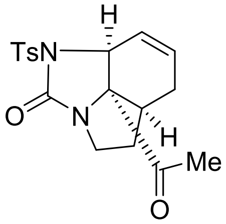

To a stirred solution of **3b** (50 mg, 0.31 mmol) in anhydrous dioxane (3 mL) was added $Pd(PPh_3)_4$ (12 mg, 0.010 mmol) and tosyl isocyanate (60 µL, 0.39 mmol) and the reaction stirred at rt under nitrogen. After 15 h the reaction was warmed to 50 °C for 3 h, cooled to rt and evaporated to give an orange oil. Purification by silica gel chromatography (EtOAc/petrol, 1:4 to 4:1 as eluent) afforded the title compound (48 mg, 43%) as a yellow solid. Mp 154 – 156 °C. ν_{max} /cm^{-1} (film) 2958, 1728, 1713, 1596 and 1351; δ_H (400 MHz, $CDCl_3$) 1.62 – 1.76 (2H, m, allylic CH*H* and NCH_2CH*H*), 1.87 – 1.97 (1H,

m, NCH_2CHH), 2.13 (3H, s, CO*Me*), 2.16 (1H, dt, J 15.6, allylic C*H*H), 2.40 (3H, Me), 2.47 – 2.56 (1H, m, CH_2CHCH_2), 3.17 (1H, ddd, J 11.7, 8.1, 5.9, NCH*H*), 3.66 (1H, ddd, J 11.7, 8.0, 6.4, NC*H*H), 4.72 (1H, d, J 3.1, NCH), 6.05 – 6.13 (2H, m, C*H*=C*H*), 7.29 (2H, d, J 8.3, 2 × CH_{Ar}) and 7.87 (2H, d, J 8.4, CH_{Ar}); δ_C (101 MHz, $CDCl_3$) 21.6 (Me), 24.5 (allylic CH_2), 25.1 (CO*Me*), 31.7 (NCH_2CH_2), 38.0 (CH_2CHCH_2), 45.2 (NCH_2), 54.0 (NCH), 74.3 (*C*q-COMe), 125.3 (CH-*C*H=), 128.0 (2 × CH_{Ar}), 129.6 (2 × CH_{Ar}), 131.4 (CH_2-*C*H=), 136.1 (Cq), 144.8 (Cq), 154.9 (urea) and 207.9 (*C*OMe); HRMS (ESI$^+$) 361.1221 ($C_{18}H_{21}N_2O_4S$, $[M+H]^+$, requires 361.1217).

(±)-(31*R*,5a*S*,8a*S*)-1-(4-chlorophenyl)-N-ethyl-2-oxo-1,4,5,5a,6,8a-hexahydroimidazo[4,5,1-hi]indole-31(2H)-carboxamide (Table 2, entry 4)

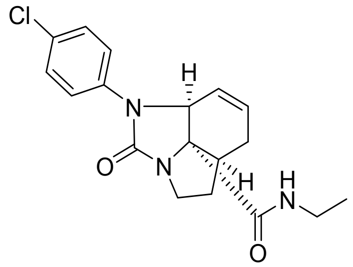

PPh_3 (20 mg, 0.08 mmol) was added to a stirred solution of $Pd(OAc)_2$ (12 mg, 0.05 mmol) in degassed anhydrous THF (10 mL). This solution was stirred for 10 minutes at room temperature under nitrogen and transferred into a stirred solution of **3a** (100 mg, 0.52 mmol) and 4-chlorophenyl isocyanate (80 mg, 0.52 mmol) in THF (10 mL). The reaction was stirred at room temperature for 48 h, the solvent removed *in vacuo* and the residue was chromatographed on silica gel eluting with a gradient of 0.25:1:98.75 to 1:1:98 MeOH/NH_3/DCM to afford the title compound (104 mg, 58%) as a white solid. Mp. 195-196 °C; ν_{max}/cm^{-1} (solid); 3275 (m, N-H), 2975 (w, C-H), 1689 (s, Urea C=O), 1651 (s, Amide C=O) 1491 (s); ^1H NMR (400 MHz, $CDCl_3$) δ_H 1.14 (t, *J* = 7.3 Hz, 3H, $NHCH_2CH_3$), 1.65 (dq, *J* = 12.7, 7.7 Hz, 1H, $HNCH_2CH_2$), 1.87 – 2.07 (m, 2H, HC=CHC*H*$_2$CH, $HNCH_2CH_2$), 2.42 (dddt, *J* = 16.6, 6.5, 4.5, 1.9 Hz, 1H, HC=CH-C*H*$_2$CH), 2.66 (app. p, *J* = 6.5 Hz, 1H, CH_2CHCH_2), 3.14 – 3.40 (m, 3H, $NHCH_2CH_3$, $HNCH_2CH_2$), 3.83 (ddd, *J* = 12.0, 7.6, 4.7 Hz, 1H, $HNCH_2CH_2$), 4.71 (dt, *J* = 3.0, 1.2 Hz, 1H, C*H*-CH=CH), 5.86 (ddt, *J* = 10.0, 3.4, 1.8 Hz, 1H, CH-C*H*=CH), 6.05 (dddd, *J* = 10.0, 5.5, 4.5, 1.2 Hz, 1H, CHCH=C*H*), 6.88 (app. t, *J* = 5.8 Hz, 1H, N*H*CH_2CH_3), 7.22 – 7.32 (m, 2H, Ar-*H* (C-3)), 7.45 – 7.54 (m, 2H, Ar-*H* (C-2)); ^{13}C NMR (101 MHz,$CDCl_3$) δ_C 14.84 ($NHCH_2CH_3$), 25.0 (HC=CH-*C*H_2CH), 32.1 ($HNCH_2CH_2$), 34.4 ($NHCH_2CH_3$), 40.7 (CH_2CHCH_2), 47.4 ($HNCH_2CH_2$), 55.9 (*C*H-CH=CH), 69.3 (*C*q), 121.7 (Ar-*C*2), 123.5 (CH*C*H=CH), 129.1 (Ar-*C*3), 129.3 (Ar-*C*4), 130.7 (CHCH=*C*H), 136.7 (Ar-*C*1), 158.4 (Urea *C*=O), 173.9 (Amide *C*=O); HRMS (ESI$^+$) 346.1310 ($C_{18}H_{21}ClN_3O_2^+$, $[M+H]^+$, requires 346.1317).

(±)-(31*R*,5a*S*,8a*S*)-1-(2-chlorophenyl)-N-ethyl-2-oxo-1,4,5,5a,6,8a-hexahydroimidazo[4,5,1-hi]indole-31(2H)-carboxamide (Table 2, entry 5)

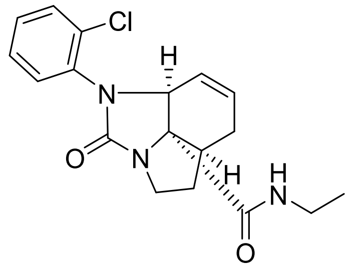

To a stirred solution of **3a** (50 mg, 0.26 mmol) in anhydrous, degassed dioxane were added $Pd(PPh_3)_4$ (18 mg, 0.016 mmol) and 2-chlorophenyl isocyanate (0.040 mL, 0.33 mmol) and the stirred mixture heated to 40 °C under nitrogen. After 16 h the reaction was evaporated to give an orange oil. Purification by silica gel chromatography (EtOAc/petrol, 3:7 to 1:0 as eluent) afforded the title compound (64 mg, 71%) as a white solid. Mp. 167 – 168 °C. ν_{max} /cm^{-1} (film) 3316 (br), 2969, 1706, 1662, 1524, 1482 and 1400; δ_H (400 MHz, $CDCl_3$) 1.17 (3H, t, *J* 7.3, Me), 1.74 (1H, h, *J* 6.5, NCH_2CHH), 1.96 – 2.07 (2H, m, NCH_2CHH and allylic CH*H*), 2.44 (1H, dt, J 16.5, 5.6, allylic C*H*H), 2.72 (1H, p, *J* 6.5, CH_2CHCH_2), 3.22 – 3.34 (2H, m, $NCHHCH_2$ and NCH*H*Me), 3.36 – 3.48 (1H, m, NC*H*HMe), 3.82 (1H, ddd, *J* 11.5, 7.5, 5.7, $NCHHCH_2$), 4.55 (1H, d, J 3.7, C*H*N), 5.63 (1H, ddt, *J* 10.0, 3.7, 1.8, CH=C*H*-CH), 6.11 (1H, dt, *J* 10.0, 4.8, C*H*=CH-CH), 7.09 (1H, brs, NH), 7.24 – 7.31 (3H, m, CH_{Ar}) and 7.42 – 7.47 (1H, m, CH_{Ar}); δ_C (101 MHz, $CDCl_3$) 14.8 (Me), 25.2 (allylic CH_2), 32.0 (NCH_2CH_2), 34.3 (NCH_2Me), 39.9 (CH_2CHCH_2), 47.0 (NCH_2CH_2), 56.5 (NCH), 70.7 (*C*q-CONHEt), 124.2 (CH=*C*H-CH), 127.5 (CHAr), 129.4 (CHAr), 130.4 (CHAr), 130.6 (*C*H=CH-CH), 131.9 (CHAr), 133.4 (CqAr), 134.0 (CqAr), 160.6 (urea) and 173.7 (*C*ONHEt); HRMS (ESI$^+$) 346.1322 ($C_{18}H_{21}ClN_3O_2$, $[M+H]^+$, requires 346.1317).

(±)-(31*R*,5a*S*,8a*S*)-1-(2-chlorophenyl)-2-oxo-1,4,5,5a,6,8a-hexahydroimidazo[4,5,1-hi]indole-31(2H)-carbonitrile (Table 2, entry 6)

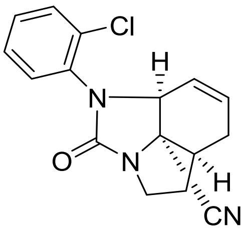

To a stirred solution of **3c** (45 mg, 0.31 mmol) in degassed, anhydrous dioxane (3 mL) was added $Pd(PPh_3)_4$ (12 mg, 0.010 mmol) and 2-chlorophenyl isocyanate (50 µL, 0.41 mmol) and the reaction stirred at 50 °C under nitrogen. After 13 h the reaction was evaporated to give a brown oil.

Purification by silica gel chromatography (EtOAc/petrol, 1:9 to 7:3 as eluent) afforded the title compound (45 mg, 49%) as a yellow solid. Mp 147 – 148 °C. ν_{max}/cm^{-1} (film) 2968, 2237, 1758, 1683, 1585 and 1474; δ_H (400 MHz, $CDCl_3$) 1.85 – 1.96 (2H, m, $NCH_2CH\mathit{H}$ and allylic CH*H*), 2.32 (1H, dt, J 16.7, 6.3, allylic CH*H*), 2.36 – 2.47 (1H, m, $NCH_2CH\mathit{H}$), 2.62 – 2.70 (1H, m, $CH_2C\mathit{H}CH_2$), 3.45 (1H, ddd, J 11.6, 8.7, 4.6, NCH*H*), 3.80 (1H, dt, J 11.6, 7.7, NC*H*H), 4.96 (1H, d, J 4.7, NCH), 5.64 (1H, ddd, J 9.9, 4.6, 2.8, CH-C*H*=), 6.19 (1H, ddd, J 9.9, 6.5, 3.7, =C*H*-CH_2), 7.19 – 7.30 (3H, m, CH_{Ar}) and 7.43 – 7.47 (1H, m, CH_{Ar}); δ_C (101 MHz, $CDCl_3$) 24.8 (allylic CH_2), 31.5 ($NCH_2\mathit{C}H_2$), 43.1 ($CH_2\mathit{C}HCH_2$), 45.6 (NCH_2), 56.1 (NCH), 62.0 (*C*q-CN), 120.8 (CN), 123.8 (Cq), 127.5 (CH-*C*H=), 129.6 (CH_{Ar}), 130.5 (CH_{Ar}), 131.5 (CH_{Ar}), 132.8 (=*C*H-CH_2), 133.5 (Cq), 133.7 (Cq) and 158.1 (urea); HRMS (ESI$^+$) 300.0884 ($C_{16}H_{15}N_3OCl$, $[M+H]^+$, requires 300.0898)

(±)-(31*R*,5a*S*,8a*S*)-N-ethyl-2-oxo-1-(2-(trifluoromethyl)phenyl)-1,4,5,5a,6,8a-hexahydroimidazo[4,5,1-hi]indole-31(2H)-carboxamide (Table 2, entry 7)

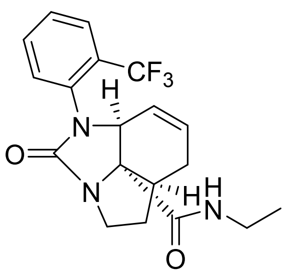

To a stirred solution of **3a** (50 mg, 0.26 mmol) in anhydrous dioxane (3 mL) was added $Pd(PPh_3)_4$ (9 mg, 0.008 mmol) and 2-trifluoromethylphenyl isocyanate (0.050 mL, 0.33 mmol) and the reaction stirred at rt under nitrogen. After 21 h the reaction was quenched by the addition of ethanol (0.10 mL) and evaporated to give an orange oil. Purification by silica gel chromatography (EtOAc/petrol, 3:7 to 1:0 as eluent) afforded the title compound (80 mg, 81%) as a white solid. Mp 155 – 157 °C. ν_{max}/cm^{-1} (film) 3324 (br), 2971, 1706, 1660, 1605, 1522, 1497, 1455 and 1397; δ_H (400 MHz, $CDCl_3$) 1.16 (3H, t, J 7.2, $NCH_2\mathit{Me}$), 1.66 – 1.76 (1H, m, $NCH_2CH\mathit{H}$), 1.92 – 2.04 (2H, m, $NCH_2C\mathit{H}H$ and allylic CH*H*), 2.41 (1H, app. dt, J 16.7, 5.7, allylic C*H*H), 2.72 (1H, quint., J 6.5, $CH_2C\mathit{H}CH_2$), 3.17 – 3.28 (2H, m, NCH*H*Me and NCH*H*CH_2), 3.38 – 3.50 (1H, m, NC*H*HMe), 3.76 (1H, dt, J 11.3, 6.9, NC*H*HCH_2), 4.38 (1H, d, J 3.5, NC*H*), 5.62 – 5.68 (1H, m, CH-C*H*=), 6.12 (1H, dt, J 10.1, 4.9, =C*H*-CH_2), 7.02 (1H, br s, NH), 7.22 (1H, d, J 7.9, CH_{Ar}), 7.46 (1H, t, J 7.6, CH_{Ar}), 7.57 (1H, app. t, J 7.7, CH_{Ar}) and 7.70 (1H, d, J 8.0, CH_{Ar}); δ_C (101 MHz, $CDCl_3$) 14.6 ($NCH_2\mathit{Me}$), 25.1 (allylic CH_2), 32.0 ($NCH_2\mathit{C}H_2$), 34.3 ($N\mathit{C}H_2Me$), 39.6 ($CH_2\mathit{C}HCH_2$), 46.3 ($N\mathit{C}H_2CH_2$), 58.2 (q, J 2, NCH), 70.7 (Cq-CONHEt), 123.4 (q, J 272, CF_3), 124.8 (CH-*C*H=), 127.4 (q, J 5.3, CH_{Ar}), 128.8 (CH_{Ar}), 129.7 (q, J 30, *C*CF_3), 130.7 (=*C*H-CH_2), 132.7 (CH_{Ar}), 133.8 (CHAr), 134.6 (q, J 1.7, Cq_{Ar}), 160.9 (urea) and 173.5 (amide); HRMS (ESI$^+$) 380.1587 ($C_{19}H_{21}F_3N_3O_2$, $[M+H]^+$, requires 380.1580)

(±)-(31*R*,5a*S*,8a*S*)-N-ethyl-2-oxo-1-(3-(trifluoromethyl)phenyl)-1,4,5,5a,6,8a-hexahydroimidazo[4,5,1-hi]indole-31(2H)-carboxamide (Table 2, entry 8)

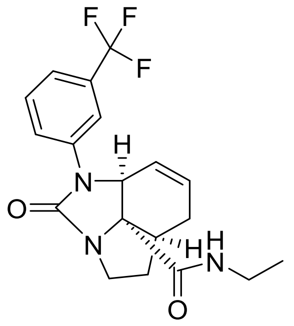

To a stirred solution of **3a** (50 mg, 0.26 mmol) in anhydrous dioxane (3 mL) was added $Pd(PPh_3)_4$ (12 mg, 0.010 mmol) and 3-(trifluoromethyl)phenyl isocyanate (0.050 mL, 0.36 mmol) and the reaction stirred at rt under nitrogen. After 14 h the reaction was quenched by the addition of ethanol (0.10 mL) and evaporated to give an orange oil. Purification by silica gel chromatography (EtOAc/petrol, 3:7 to 1:0 as eluent) afforded the title compound (90 mg, 91%) as a yellow solid. Mp 163 – 165 °C. ν_{max}/cm^{-1} (film) 3324 (br), 2973, 1702, 1658, 1612, 1496 and 1321; δ_H (400 MHz, $CDCl_3$) 1.14 (3H, t, J 7.2, NCH$_2$*Me*), 1.67 (1H, dtd, J 12.7, 7.8, 7.6, NCH$_2$CH*H*), 1.92 – 2.05 (2H, m, NCH$_2$C*H*H and allylic CH*H*), 2.39 – 2.48 (1H, m, allylic C*H*H), 2.68 (1H, quint., J 6.5, CH$_2$C*H*CH$_2$), 3.17 – 3.49 (3H, m, NC*H*$_2$Me and NC*H*HCH$_2$), 3.85 (1H, ddd, J 11.6, 7.4, 4.6, NC*H*HCH$_2$), 4.80 (1H, t, J 1.3, NC*H*), 5.90 (1H, ddt, J 10.1, 3.1, 1.6, CH-C*H*=), 6.09 (1H, dt, J 10.1, 4.7, =C*H*-CH$_2$), 6.91 (1H, br t, J 4.7, NH), 7.32 (1H, d, J 7.8, CH$_{Ar}$), 7.43 (1H, t, J 7.9, CH$_{Ar}$), 7.74 (1H, d, J 8.2, CH$_{Ar}$) and 7.90 (1H, s, CH$_{Ar}$); δ_C (101 MHz, $CDCl_3$) 14.7 (NCH$_2$*Me*), 24.8 (allylic CH$_2$), 32.0 (NCH$_2$*C*H$_2$), 34.3 (N*C*H$_2$Me), 40.6 (CH$_2$*C*HCH$_2$), 47.2 (N*C*H$_2$CH$_2$), 55.7 (NCH), 69.2 (Cq-CONHEt), 116.4 (q, J 4.0, CHAr), 120.2 (q, J 3.9, CHAr), 122.6 (CHAr), 123.2 (CH-*C*H=) 123.7 (q, J 272, CF$_3$), 129.5 (CH$_{Ar}$), 130.9 (=CH-CH$_2$), 131.4 (q, J 33, *C*CF$_3$), 139.7 (Cq$_{Ar}$), 159.2 (urea) and 173.7 (amide); HRMS (ESI$^+$) 380.1575 ($C_{19}H_{21}F_3N_3O_2$, [M+H]$^+$, requires 380.1580).

(±)-(31*R*,5a*S*,8a*S*)-N-ethyl-2-oxo-1-(4-(trifluoromethyl)phenyl)-1,4,5,5a,6,8a-hexahydroimidazo[4,5,1-hi]indole-31(2H)-carboxamide (Table 2, entry 9)

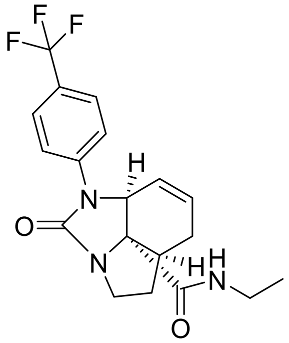

To a stirred solution of **3a** (50 mg, 0.26 mmol) in anhydrous dioxane (3 mL) was added $Pd(PPh_3)_4$ (12 mg, 0.010 mmol) and 4-(trifluoromethyl)phenyl isocyanate (0.050 mL, 0.35 mmol) and the reaction stirred at rt under nitrogen. After 15 h the reaction was quenched by the addition of ethanol (0.10 mL) and evaporated to give a red solid. Purification by silica gel chromatography (EtOAc/petrol, 3:7 to 1:0 as eluent) afforded the title compound (53 mg, 54%) as a yellow solid. Mp 192 – 194 °C. ν_{max} /cm^{-1} (film) 3323, 2973, 1704, 1658, 1614, 1520 and 1320; δ_H (400 MHz, $CDCl_3$) 1.14 (3H, t, J 7.3, NCH_2*Me*), 1.67 (1H, ddt, J 12.7, 7.8, 7.7, NCH_2CH*H*), 1.92 – 2.05 (2H, m, NCH_2C*H*H and allylic CH*H*), 2.39 – 2.48 (1H, m, allylic C*H*H), 2.69 (1H, quint., J 6.5, CH_2C*H*CH_2), 3.17 – 3.40 (3H, m, NC*H*$_2$Me and NCH*H*CH_2), 3.85 (1H, ddd, J 11.7, 7.7, 4.6, NC*H*HCH_2), 4.81 (1H, t, J 1.1, NC*H*), 5.92 (1H, ddt, J 10.1, 3.0, 1.7, CH-C*H*=), 6.09 (1H, dt, J 10.1, 4.8, =C*H*-CH_2), 6.87 (1H, br t, J 4.8, NH), 7.56 (2H, d, J 8.8, CH_{Ar}) and 7.72 (2H, d, J 8.8, CH_{Ar}); δ_C (101 MHz, $CDCl_3$) 14.7 (NCH_2*Me*), 24.8 (allylic CH_2), 32.1 (NCH_2*C*H_2), 34.3 (N*C*H_2Me), 40.6 (CH_2*C*HCH_2), 47.1 (N*C*H_2CH_2), 55.6 (NCH), 69.1 (Cq-CONHEt), 118.8 (2 × CHAr), 123.2 (CH-*C*H=), 124.0 (q, J 272, CF_3), 125.2 (q, J 32, *C*CF_3), 126.2 (q, J 3.8, 2 × CH_{Ar}), 130.8 (=*C*H-CH_2), 141.3 (q, J 1.4, Cq_{Ar}), 159.1 (urea) and 173.6 (amide); HRMS (ESI$^+$) 380.1588 ($C_{19}H_{21}F_3N_3O_2$, $[M+H]^+$, requires 380.1580).

(±)-(31*R*,5a*S*,8a*S*)-31-acetyl-1-(4-(trifluoromethyl)phenyl)-31,4,5,5a,6,8a-hexahydroimidazo[4,5,1-hi]indol-2(1H)-one (Table 2, entry 10)

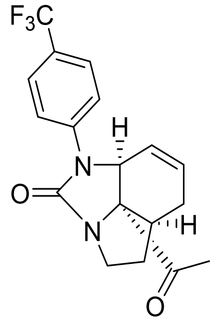

To a stirred solution of **3b** (50 mg, 0.31 mmol) in anhydrous dioxane (3 mL) was added $Pd(PPh_3)_4$ (12 mg, 0.010 mmol) and 4-(trifluoromethyl)phenyl isocyanate (50 µL, 0.35 mmol) and the reaction stirred at rt under nitrogen. After 16 h the reaction was quenched by the addition of EtOH (0.2 mL) and evaporated to give an orange oil. Purification by silica gel chromatography (EtOAc/petrol, 1:9 to 3:2 as eluent) the title compound (59 mg, 55%) as a yellow solid. Mp 126 – 128 °C. ν_{max} /cm^{-1} (film) 2935, 1704, 1614, 1522, 1428 and 1320; δ_H (400 MHz, $CDCl_3$) 1.68 (1H, dq, J 12.5, 8.5, NCH_2CH_2), 1.93 – 2.02 (1H, m, NCH_2C*H*H), 2.06 (1H, dt, J 16.8, 5.2, allylic CH*H*), 2.19 – 2.28 (1H, m, allylic C*H*H), 2.32 (3H, s, Me), 2.54 – 2.61 (1H, m, CH_2C*H*CH_2), 3.27 (1H, ddd, J 11.9, 9.1, 6.7, NCH*H*), 3.95 (1H, ddd, J 11.8, 7.9, 3.5, NC*H*H), 4.87 (1H, d, J 1.5, NCH), 5.93 (1H, app. d, J 10.1, CH-C*H*=), 6.03 (1H, dddd, J 10.1, 5.6, 3.9, 1.5, =C*H*-CH_2), 7.56 (2H, d, J 8.9, 2 × CH_{Ar}) and 7.73 (2H, d, 8.7, 2 × CH_{Ar}); δ_C (101 MHz, $CDCl_3$) 24.2 (allylic CH_2), 25.0 (CO*Me*), 32.2 (NCH_2CH_2), 39.1 (CH_2*C*HCH_2), 47.2 (NCH_2), 54.0 (NCH), 73.6 (Cq-N), 118.4 (2 × CH_{Ar}), 123.5 (CH-*C*H=), 124.0 (q, J 124, *C*F_3), 124.8 (q, J 32, *C*CF_3) 126.2 (q, J 3.7, 2 × *C*HCCF_3), 129.8 (=CH-CH_2), 141.5 (Cq), 158.7 (urea) and 210.1 (*C*OMe); HRMS (ESI^+) 351.1314 ($C_{18}H_{17}F_3N_2O_2$, $[M+H]^+$, requires 351.1315).

(±)-(31*R*,5a*S*,8a*S*)-N-ethyl-2-oxo-1-(2-(nitro)phenyl)-1,4,5,5a,6,8a-hexahydroimidazo[4,5,1-hi]indole-31(2H)-carboxamide (Table 2, entry 11)

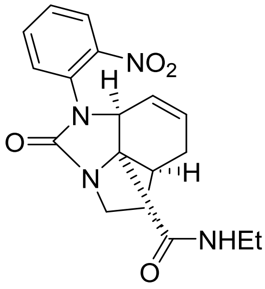

To a stirred solution of **3a** (50 mg, 0.26 mmol) in degassed, anhydrous dioxane (3 mL) was added $Pd(PPh_3)_4$ (12 mg, 0.010 mmol) and 2-nitrophenyl isocyanate (47 mg, 0.34 mmol) and the reaction stirred at rt under nitrogen. After 14 h the reaction was evaporated to give a brown oil. Purification by silica gel chromatography (EtOAc/petrol, 3:7 to 1:0 as eluent) afforded the title compound (82 mg, 89%) as a yellow solid. Mp 170 – 171 °C. ν_{max} /cm^{-1} (film) 3324, 1705, 1658, 1528 and 1354; δ_H (400 MHz, $CDCl_3$) 1.16 (3H, t, J 7.3, NCH_2*Me*), 1.75 (1H, ddt, J 13.0, 7.6, 5.2, NCH_2CH*H*), 1.95 – 2.07 (2H, m, allylic CH*H* and NCH_2*C*HH), 2.38 (1H, dt, J 16.4, 5.9, allylic *C*HH), 2.68 – 2.76 (1H, m, CH_2*CH*CH_2), 3.23 – 3.42 (3H, m, NCH*H*CH_2 and NC*H*2Me), 3.76 (1H, dt, J 11.3, 7.2, NC*H*HCH_2), 4.51 (1H, d, J 4.4, NCH), 5.80 – 5.86 (1H, m, =CH), 6.23 (1H, ddd, J 9.9, 5.6, 4.2, =CH), 7.05 (1H, br t, J 5.0, NH), 7.32 (1H, d, J 8.0, CH_{Ar}), 7.40 (1H, t, J 8.0, CH_{Ar}), 7.58 (1H, t, J 7.9, CH_{Ar}) and 7.95 (1H, dd, J 8.1, 1.1, CH_{Ar}); δ_C (101 MHz, $CDCl_3$) 14.7 (NCH2*Me*), 25.4 (allylic CH_2), 31.4 (NCH_2CH_2), 39.5 (N*C*H2Me), 40.2 (CH2*C*HCH2), 46.8 (N*C*H_2CH_2), 57.3 (NCH), 71.6 (*C*q-CONHEt), 124.5 (=CH), 125.8 (CH_{Ar}), 127.8 (CH_{Ar}), 129.6 (CH_{Ar}), 131.1 (Cq), 132.8 (=CH), 133.6 (CH_{Ar}), 146.3 (Cq), 159.9 (urea) and 173.2 (*C*ONHEt); HRMS (ESI$^+$) 357.1574 ($C_{18}H_{21}N_4O_4$, $[M+H]^+$, requires 357.1557).

(±)-(31*R*,5a*S*,8a*S*)-1-(2-nitrophenyl)-2-oxo-1,4,5,5a,6,8a-hexahydroimidazo[4,5,1-hi]indole-31(2H)-carbonitrile (Table 2, entry 12)

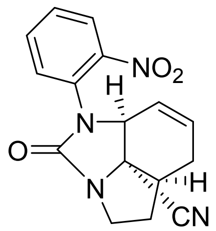

To a stirred solution of **3c** (50 mg, 0.34 mmol) in degassed, anhydrous dioxane (3 mL) was added $Pd(PPh_3)_4$ (13 mg, 0.011 mmol) and 2-nitrophenyl isocyanate (62 mg, 0.38 mmol) and the reaction stirred at 50 °C under nitrogen. After 18 h the reaction was evaporated to give a yellow solid. Purification by silica gel chromatography (EtOAc/petrol, 3:7 to 7:3 as eluent) afforded the title

compound (57 mg, 54%) as a yellow solid. Mp 182 – 183 °C. ν_{max}/cm^{-1} (film) 2953, 2243, 1712, 1605, 1528, 1438 and 1398; δ_H (400 MHz, $CDCl_3$) 1.91 – 2.08 (2H, m, NCH_2CH*H* and allylic CH*H*), 2.35 (1H, dt, J 16.5, 6.4, allylic C*H*H), 2.39 – 2.49 (1H, m, NCH_2C*H*H), 2.63 – 2.70 (1H, m, CH_2C*H*CH_2), 3.47 (1H, ddd, J 11.4, 8.9, 4.2, NCH*H*), 3.80 (1H, dt, J 11.4, 7.8, NC*H*H), 5.05 (1H, d, J 4.9, NCH), 5.84 (1H, ddd, J 9.9, 4.9, 2.9, =CH), 6.30 (1H, ddd, J 9.9, 6.8, 3.4, =CH), 7.36 (1H, dd, J 8.0, 1.3, CH_{Ar}), 7.43 (1H, td, J 7.9, 1.3, CH_{Ar}), 7.62 (1H, td, J 7.7, 1.4, CH_{Ar}) and 7.95 (1H, dd, J 8.1, 1.5, CH_{Ar}); δ_C (101 MHz, $CDCl_3$) 24.9 (allylic CH2), 31.3 (NCH_2*C*H_2), 43.5 (CH_2*C*HCH_2), 45.8 (NCH_2), 56.5 (NCH), 62.4 (*C*q-CN), 120.5 (CN), 123.1 (=CH), 125.9 (CH_{Ar}), 128.0 (CH_{Ar}), 128.5 (CH_{Ar}), 130.3 (Cq), 133.5 (CH_{Ar}), 134.7 (=CH), 146.4 (Cq) and 157.3 (urea); HRMS (ESI$^+$) 311.1131 ($C_{16}H_{15}N_4O_3$, $[M+H]^+$, requires 311.1139).

(±)-(31*R*,5a*S*,8a*S*)-N-ethyl-1-(4-nitrophenyl)-2-oxo-1,4,5,5a,6,8a-hexahydroimidazo[4,5,1-hi]indole-31(2H)-carboxamide (Table 2, entry 13)

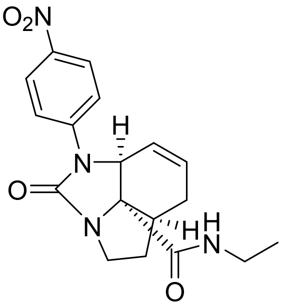

To a stirred solution of **3a** (50 mg, 0.26 mmol) in anhydrous dioxane (3 mL) was added $Pd(PPh_3)_4$ (12 mg, 0.010 mmol) and 4-nitrophenyl isocyanate (55 mg, 0.34 mmol) and the reaction stirred at rt under nitrogen. After 18 h the reaction was quenched by the addition of ethanol (0.10 mL) and evaporated to give an orange oil. Purification by silica gel chromatography (EtOAc/petrol, 3:7 to 4:1 as eluent) afforded the title compound (82 mg, 89%) as a yellow solid. Mp 196 °C (dec.). ν_{max}/cm^{-1} (film) 3332, 2972, 1707, 1660, 1594, 1501 and 1319; δ_H (400 MHz, $CDCl_3$) 1.14 (3H, t, J 7.3, NCH_2*Me*), 1.63 – 1.74 (1H, m, NCH_2CH*H*), 1.95 – 2.08 (2H, m, NCH_2CH*H* and allylic CH*H*), 2.41 – 2.51 (1H, m, allylic C*H*H), 2.71 (1H, quint., J 6.4, CH_2C*H*CH_2), 3.18 – 3.41 (3H, m, NC*H*$_2$Me and NCH*H*CH_2), 3.87 (1H, ddd, J 11.7, 7.7, 4.6, NC*H*HCH_2), 4.85 (1H, s NC*H*), 5.92 – 5.98 (1H, m, CH-C*H*=), 6.13 (1H, dt, J 10.2, 4.8, =C*H*-CH_2), 6.76 (1H, br t, J 5.1, NH), 7.77 (2H, dt, J 9.4, 3.1, 2 × CH_{Ar}) and 8.19 (2H, dt, J 9.4, 3.1, 2 × CH_{Ar}); δ_C (101 MHz, $CDCl_3$) 14.7 (NCH_2*Me*), 24.8 (allylic CH_2), 32.2 (NCH_2*C*H_2), 34.4 (N*C*H_2Me), 40.6 (CH_2*C*HCH_2), 47.1 (N*C*H_2CH_2), 55.8 (NCH), 69.0 (*C*q-CONHEt), 117.9 (2 × CHAr), 122.7 (CH-*C*H=), 125.0 (2 × CHAr), 131.4 (=*C*H-CH_2), 142.5 (Cq), 144.2 (Cq), 158.6 (urea) and 173.3 (amide); HRMS (ESI$^+$) 357.1553 ($C_{18}H_{21}N_4O_4$, $[M+H]^+$, requires 357.1557).

(±)-(31*R*,5a*S*,8a*S*)-1-(4-acetylphenyl)-N-ethyl-2-oxo-1,4,5,5a,6,8a-hexahydroimidazo[4,5,1-hi]indole-31(2H)-carboxamide (Table 2, entry 14)

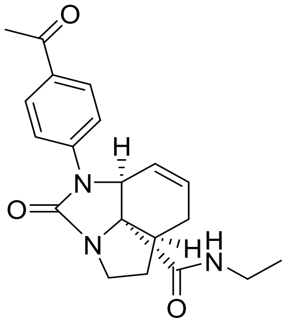

To a stirred solution of **3a** (50 mg, 0.26 mmol) in anhydrous dioxane (3 mL) was added $Pd(PPh_3)_4$ (12 mg, 0.010 mmol) and 4-acetylphenyl isocyanate (45 mg, 0.28 mmol) and the reaction stirred at rt under nitrogen. After 16 h the reaction was quenched by the addition of ethanol (0.10 mL) and evaporated to give a red solid. Purification by silica gel chromatography (EtOAc/petrol, 3:7 to 1:0 as eluent) afforded the title compound (73 mg, 79%) as a yellow solid. Mp 200 – 201 °C. ν_{max} /cm^{-1} (film) 3330, 2971, 1705, 1661, 1599, 1512 and 1354;δ_H (400 MHz, $CDCl_3$) 1.14 (3H, t, J 7.3, NCH_2*Me*), 1.67 (1H, ddt, J 12.7, 7.9, 7.8, NCH_2CH*H*), 1.92 – 2.07 (2H, m, NCH_2CH*H* and allylic CH*H*), 2.40 – 2.49 (1H, m, allylic C*H*H), 2.55 (3H, s, Me), 2.69 (1H, quint., J 6.5, CH_2*C*HCH_2), 3.17 – 3.41 (3H, m, NC*H*$_2$Me and NCH*H*CH_2), 3.86 (1H, ddd, J 11.7, 7.6, 4.4, NC*H*HCH_2), 4.83 (1H, t, J 1.3, NC*H*), 5.95 (1H, ddt, J 10.1, 3.0, 1.5, CH-C*H*=), 6.09 (1H, dt, J 10.1, 4.8, =C*H*-CH_2), 6.83 (1H, d, J 5.4, CH_{Ar}), 7.70 (2H, dt, J 8.9, 2.0, 2 × CH_{Ar}) and 7.93 (1H, dt, J 8.9, 2.0, 2 × CH_{Ar}); δ_C (101 MHz, $CDCl_3$) 14.7 (NCH_2*Me*), 24.8 (allylic CH_2), 26.4 (CO*Me*) 32.1 (NCH_2*C*H_2), 34.4 (N*C*H_2Me), 40.6 (CH_2*C*HCH_2), 47.1 (N*C*H_2CH_2), 55.6 (NCH), 69.0 (*C*q-CONHEt), 118.1 (2 × CH_{Ar}), 123.2 (CH-*C*H=), 129.6 (2 × CH_{Ar}), 130.7 (=*C*H-CH_2), 131.9 (Cq), 142.6 (Cq), 159.0 (urea), 173.6 (amide) and 196.9 (ketone); HRMS (ESI$^+$) 354.1800 ($C_{20}H_{24}N_3O_3$, $[M+H]^+$, requires 354.1812).

(±)-(31*R*,5a*S*,8a*S*)-N-ethyl-1-(4-methoxyphenyl)-2-oxo-1,4,5,5a,6,8a-hexahydroimidazo[4,5,1-hi]indole-31(2H)-carboxamide (Table 2, entry 15)

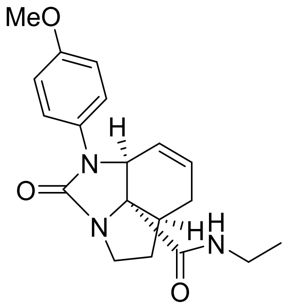

To a stirred solution of **3a** (50 mg, 0.26 mmol) in anhydrous dioxane (3 mL) was added $Pd(PPh_3)_4$ (12 mg, 0.010 mmol) and 4-methoxyphenyl isocyanate (0.050 mL, 0.39 mmol) and the reaction stirred at rt under nitrogen. After 16 h the reaction was quenched by the addition of ethanol (0.10 mL) and evaporated to give a brown oil. Purification by silica gel chromatography (EtOAc/petrol, 3:7 to 1:0 as eluent) afforded the title compound (71 mg, 80%) as a yellow solid. Mp 167 – 168 °C. ν_{max} /cm^{-1} (film) 3316, 2968, 1698, 1661, 1511, 1396 and 1246; δ_H (400 MHz, $CDCl_3$) 1.15 (3H, t, J 7.3, NCH_2*Me*), 1.66 (1H, ddt, J 12.8, 7.7, 7.6, NCH_2CH*H*), 1.89 – 2.04 (2H, m, NCH_2CH*H* and allylic CH*H*), 2.37 – 2.46 (1H, m, allylic C*H*H), 2.65 (1H, quint., J 6.4, CH_2C*H*CH_2), 3.16 – 3.40 (3H, m, NC*H*$_2$Me and NCH*H*CH_2), 3.78 (3H, s, OMe), 3.84 (1H, ddd, J 11.3, 7.6, 4.7, NC*H*HCH_2), 4.63 (1H, t, J 1.7, NC*H*), 5.80 (1H, ddt, J 10.1, 3.4, 1.7, CH-C*H*=), 6.03 (1H, dt, J 10.1, 4.7, =C*H*-CH_2), 6.87 (2H, dt, J 9.1, 2.3, 2 × CH_{Ar}), 6.96 (1H, br t, J 4.9, NH) and 7.37 (2H, dt, J 9.1, 2.3, 2 × CH_{Ar}); δ_C (101 MHz, $CDCl_3$) 14.8 (NCH_2*Me*), 25.0 (allylic CH_2), 32.0 (NCH_2CH_2), 34.2 (NCH_2Me), 40.6 (CH_2*C*HCH_2), 47.5 (N*C*H$_2CH_2$), 55.5 (OMe), 56.5 (NCH), 69.5 (*C*q-CONHEt), 114.3 (2 × CHAr), 123.88 (2 × CHAr), 124.93 (CH-*C*H=), 130.0 (=*C*H-CH_2), 130.6 (Cq), 156.8 (Cq), 160.0 (urea) and 174.1 (amide); HRMS (ESI$^+$) 342.1815 ($C_{19}H_{24}N_3O_3$, $[M+H]^+$, requires 342.1812).

(±)-(31*R*,5a*S*,8a*S*)-N-ethyl-1-(2-methoxyphenyl)-2-oxo-1,4,5,5a,6,8a-hexahydroimidazo[4,5,1-hi]indole-31(2H)-carboxamide (Table 2, entry 16)

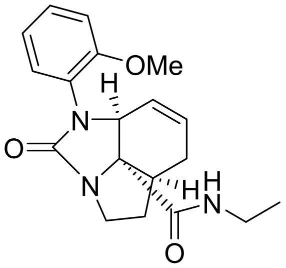

To a stirred solution of **3a** (50 mg, 0.26 mmol) in anhydrous dioxane (3 mL) was added $Pd(PPh_3)_4$ (12 mg, 0.010 mmol) and 2-methoxyphenyl isocyanate (0.050 mL, 0.38 mmol) and the reaction stirred at rt under nitrogen. After 40 h the reaction was quenched by the addition of ethanol (0.10 mL) and evaporated to give a brown oil. Purification by silica gel chromatography (EtOAc/petrol, 3:7 to 1:0 as eluent) followed by a second column (MeCN/DCM, 1:9 to 2:3 as eluent) afforded the title compound (34 mg, 38%) as a yellow solid. Mp 154 – 155 °C. ν_{max}/cm^{-1} (film) 3315, 2967, 1700, 1659, 1502 and 1397; δ_H (400 MHz, $CDCl_3$) 1.16 (3H, t, J 7.3, NCH_2*Me*), 1.62 – 1.73 (1H, m, NCH_2CH*H*), 1.89 – 2.03 (2H, m, NCH_2CH*H* and allylic CH*H*), 2.39 (1H, app dt, J 16.6, 5.4, allylic C*H*H), 2.66 (1H, quint., J 6.5, CH_2C*H*CH_2), 3.15 – 3.43 (3H, m, NC*H*$_2$Me and NCH*H*CH_2), 3.76 (3H, s, OMe), 3.80 (1H, ddd, J 11.7, 7.6, 5.6, NC*H*HCH_2), 4.53 (1H, d, J 3.6, NC*H*), 5.59 (1H, ddt, J 10.0, 3.8, 1.7, CH-C*H*=), 6.01 (1H, dt, J 10.0, 4.9, =C*H*-CH_2), 6.87 – 6.95 (2H, m, 2 × CH_{Ar}), 7.09 (1H, brs, NH) and 7.18 - 7.28 (2H, m, 2 × CH_{Ar}); δ_C (101 MHz, $CDCl_3$) 14.9 (NCH_2*Me*), 25.3 (allylic CH_2), 31.9 (NCH_2*C*H_2), 34.2 (N*C*H_2Me), 40.0 (CH_2*C*HCH_2), 47.2 (N*C*$H_2$$CH_2$), 55.5 (OMe), 56.2 (NCH), 70.5 (*C*q-CONHEt), 111.7 (CH_{Ar}), 120.6 (CH_{Ar}), 124.8 (CH-*C*H=), 125.2 (Cq), 128.9 (CH_{Ar}), 129.7 (=*C*H-CH_2), 130.7 (CH_{Ar}), 155.4 (Cq), 161.2 (urea) and 174.2 (amide); HRMS (ESI$^+$) 342.1807 ($C_{19}H_{24}N_3O_3$, $[M+H]^+$, requires 342.1812).

(±)-(31*R*,5a*S*,8a*S*)-1-(2,6-dichlorophenyl)-N-ethyl-2-oxo-1,4,5,5a,6,8a-hexahydroimidazo[4,5,1-hi]indole-31(2H)-carboxamide and (±)-(31*R*,5a*S*,8a*S*,*Z*)-2-((2,6-dichlorophenyl)imino)-N-ethyl-8a-methyl-5,5a,6,8a-tetrahydro-2H-oxazolo[5,4,3-hi]indole-31(4H)-carboxamide (Table 2, entry 17)

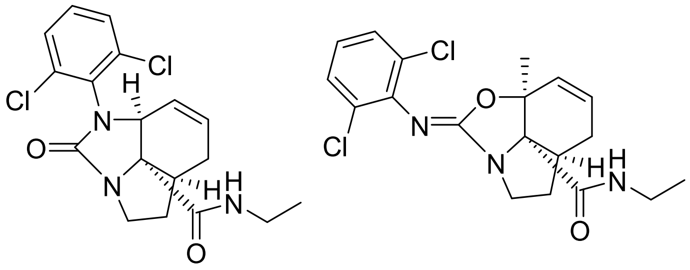

To a stirred solution of **3a** (50 mg, 0.26 mmol) in anhydrous dioxane (3 mL) was added $Pd(PPh_3)_4$ (12 mg, 0.010 mmol) and 2,6-dichlorophenyl isocyanate (60 mg, 0.32 mmol) and the reaction stirred at rt under nitrogen. After 14 h the reaction was quenched by the addition of ethanol (0.10 mL) and evaporated to give an orange oil. Purification by silica gel chromatography (EtOAc/petrol, 1:4 to 4:1 as eluent) afforded *O*-cyclised product (40 mg, 40%) and *N*-cyclised product (29 mg, 29%), both white solids. Mp 217 – 219 °C. ν_{max}/cm^{-1} (film) 3301, 2934, 1658, 1519, 1433 and 1245; δ_H (400 MHz, $CDCl_3$) 1.16 (3H, t, J 7.3, NCH_2*Me*), 1.69 – 1.81 (1H, m, NCH_2CH*H*), 1.93 – 2.04 (1H, m, NCH_2CH*H*), 2.11 – 2.21 (1H, m, allylic CH*H*), 2.43 - 2.53 (1H, m, allylic C*H*H), 2.62 – 2.71 (1H, m, CH_2C*H*CH_2), 3.24 – 3.40 (3H, m, NC*H*$_2$Me and NCH*H*CH_2), 4.10 (1H, app q, J 7.1, NC*H*HCH_2), 4.93 (1H, s, NC*H*), 5.80 (1H, d, J 10.1, CH-C*H*=), 6.00 – 6.07 (1H, m, =C*H*-CH_2), 6.87 (1H, t, J 8.0, 1 × CH_{Ar}), 6.97 (1H, brs, NH) and 7.24 (2H, d, J 8.0, 2 × CH_{Ar}); ^{13}C NMR (101 MHz, DMSO) δ_C 15.2 (NCH_2*Me*), 24.9 (allylic CH_2), 32.8 (NCH_2*C*H_2), 34.1 (N*C*H_2Me), 40.7 (CH_2*C*HCH_2), 50.2 (N*C*H_2CH_2), 73.7 (*C*q-CONHEt), 76.3 (*C*HO), 124.0 (1 × CH_{Ar}), 124.8 (*C*H=CH-CH_2), 128.1 (*C*Cl), 128.4 (2 × CH_{Ar}), 128.9 (CCl), 131.7 (CH=*C*H-CH_2), 143.6 (Cq-N), 157.5 (imidate) and 173.0 (amide). HRMS (ESI$^+$) 380.0944 ($C_{18}H_{20}Cl_2N_3O_3$, $[M+H]^+$, requires 380.0927). (31*R*,5a*S*,8a*S*)-1-(2,6-dichlorophenyl)-N-ethyl-2-oxo-1,4,5,5a,6,8a-hexahydroimidazo[4,5,1-hi]indole-31(2H)-carboxamide: Mp 197 – 199 °C. ν_{max} /cm^{-1} (film) 3317, 2970, 1710, 1659, 1526, 1463 and 1394; δ_H (400 MHz, $CDCl_3$) 1.14 (3H, t, J 7.3, NCH_2*Me*), 1.74 (1H, dq, J 12.8, 7.3, NCH_2CH*H*), 1.97 (1H, app quint., NCH_2CH*H*, 6.3), 2.11 (1H, dt, J 16.6, 5.6, allylic CH*H*), 2.40 – 2.49 (1H, m, allylic C*H*H), 2.68 (1H, quint., J 6.5, CH_2C*H*CH_2), 3.18 – 3.29 (2H, m, NCH*H*Me and NCH*H*CH_2), 3.35 – 3.48 (1H, m, NC*H*HMe), 3.85 (1H, ddd, J 11.3, 7.4, 5.4, NC*H*HCH_2), 4.60 (1H, d, J 3.6, NC*H*), 5.59 (1H, ddt, J 10.1, 1.9, 1.7, CH-C*H*=), 6.06 (1H, dt, J 10.1, 4.6, =C*H*-CH_2), 7.10 (1H, brs, NH), 7.22 (1H, t, J 8.1, CH_{Ar}), 7.35 (1H, dd, J 8.1, 1.3, CH_{Ar}) and 7.38 (1H, dd, J 8.1, 1.4, CH_{Ar}); δ_C (101 MHz, $CDCl_3$) 14.8 (NCH_2*Me*), 25.1 (allylic CH_2), 32.1 (NCH_2*C*H_2), 34.2 (N*C*H_2Me), 40.1 (CH_2*C*HCH_2), 47.5 (N*C*H_2CH_2), 56.0 (NCH), 70.7 (Cq-CONHEt), 123.4 (CH-*C*H=), 128.8 (CH_{Ar}), 129.1 (CH_{Ar}), 130.0 (CH_{Ar}),

130.4 (=CH-CH_2), 131.9 (Cq), 135.6 (Cq), 137.6 (Cq), 159.7 (urea) and 173.9 (amide); HRMS (ESI$^+$) 380.0941 ($C_{18}H_{20}Cl_2N_3O_3$, $[M+H]^+$, requires 380.0927).

(±)-(31*R*,5a*S*,8a*S*,*Z*)-N-ethyl-2-(tosylimino)-5,5a,6,8a-tetrahydro-2H-oxazolo[5,4,3-hi]indole-31(4H)-carboxamide (Table 2, entry 18)

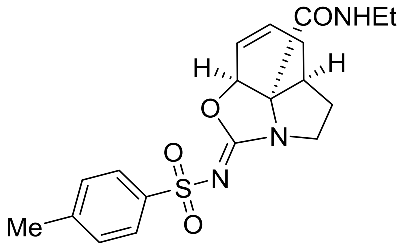

Uncatalysed procedure

To a stirred solution of **3a** (50 mg, 0.26 mmol) in anhydrous dioxane (3 mL) was added tosyl isocyanate 50 µL, 0.32 mmol) and the reaction stirred at rt under nitrogen. After 16 h the reaction was evaporated to give a yellow oil. Purification by silica gel chromatography (EtOAc/petrol, 3:7 to 1:0 as eluent) afforded the title compound (52 mg, 51%) as a white solid.

Catalysed procedure

To a solution of **3a** (50 mg, 0.26 mmol) in anhydrous dioxane (3 mL) under nitrogen were added tosyl isocyanate (0.050 mL, 0.33 mmol) and $Pd(PPh_3)_4$ (9 mg, 8 µmol) and the reaction stirred at rt. After 16 h the reaction was evaporated to give a yellow oil. Purification by silica gel chromatography (EtOAc/petrol, 1:1 to 1:0 as eluent) afforded the title compound (81 mg, 80%) as a white solid. Mp. 190 – 192 °C. ν_{max}/cm^{-1} (film) 3341 (br), 2974, 2251, 1660, 1592, 1525, 1405 and 1300; δ_H (500 MHz, $CDCl_3$) 1.12 (3H, t, J 7.3, CH_2*Me*), 1.58 (1H, dq, *J* 12.8, 7.3, NCH_2CH*H*), 1.77 (1H, dtd, J 17.0, 5.4, 1.9, allylic CH*H*), 1.98 – 2.05 (1H, m, NCH_2C*H*H), 2.31 (1H, dddt, *J* 17.2, 6.4, 4.1, 1.9, allylic C*H*H), 2.39 (3H, s, Me), 2.65 (1H, p, *J* 6.4, CH_2C*H*CH_2), 3.20 – 3.36 (3H, m, NC*H*$_2$Me and NCH*H*CH_2), 3.78 (1H, ddd, *J* 12.2, 7.5, 5.1, NC*H*HCH_2), 5.14 (1H, d, *J* 3.7, CHO), 5.60 (1H, ddt, *J* 10.1, 3.7, 1.9, CH=C*H*-CH), 5.99 (1H, dtd, *J* 10.1, 4.8, 1.1, C*H*-CH=CH), 6.84 (1H, brt, *J* 5.8, NH), 7.24 – 7.20 (2H, m, CH_{Ar}) and 7.81 – 7.78 (2H, m, CH_{Ar}); δ_C (126 MHz, $CDCl_3$) 14.6 (*Me*CH_2), 21.5 (Me), 24.6 (allylic CH_2), 32.9 (NCH_2*C*H_2), 34.6 (NCH2Me), 38.9 (CH_2*C*HCH_2), 48.7 (N*C*H$_2$$CH_2$), 73.1 (*C*q-CONHEt), 79.6 (CHO), 122.4 (CH=*C*H-CH), 127.1 (CHAr), 127.1 (CHAr), 132.5 (*C*H=CH-CH), 139.3 (CqAr), 142.8 (CqAr), 161.6 (imidate) and 171.4 (*C*ONHEt); HRMS (ESI$^+$) 390.1475 ($C_{19}H_{24}N_3O_4S$, $[M+H]^+$, requires 390.1482).

(±)-(31*R*,5a*S*,8a*S*,*Z*)-2-((2-chlorophenyl)imino)-N-ethyl-5,5a,6,8a-tetrahydro-2H-oxazolo[5,4,3-hi]indole-31(4H)-carboxamide (Table 2, entry 20)

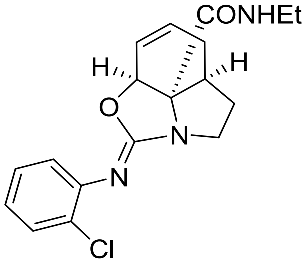

To a solution of **3a** (50 mg, 0.26 mmol) in anhydrous dioxane (3 mL) under nitrogen were added $Pd(PPh_3)_4$ (9 mg, 8 µmol) and 2-chlorophenyl isocyanate (0.050 mL, 0.41 mmol) and the reaction stirred at rt. After 16 h the reaction was quenched by the addition of ethanol (0.2 mL) and evaporated to give a yellow oil. Purification by silica gel chromatography (EtOAc/petrol, 1:1 to 1:0 as eluent) afforded the title compound (86 mg, 96%) as a clear oil. Mp 134 – 136 °C. ν_{max} /cm^{-1} (film) 3314, 2971, 1660, 1586, 1524, 1375 and 1236; δ_H (500 MHz, $CDCl_3$) 1.20 (3H, t, J 7.3, NCH_2*Me*), 1.72 – 1.82 (1H, m, NCH_2C*H*H), 1.96 – 2.04 (1H, m, NCH_2CH*H*), 2.19 (1H, app. dt, J 17.1, 3.8, allylic CH*H*), 2.52 (1H, app. dp, J 17.1, 2.9, allyic CH*H*), 2.67 – 2.74 (1H, m, CH_2C*H*CH_2), 3.28 – 3.42 (3H, m, NCH*H*CH_2 and NC*H*$_2$Me), 4.08 (1H, app. t, J 8.2, NC*H*HCH_2), 4.96 – 4.98 (1H, m, =CH-C*H*N), 5.86 (1H, d, J 10.1, =C*H*-CHN), 6.09 (1H, ddt, J 10.1, 4.0, 1.2, =CH-CH_2), 6.97 (1H, td, J 7.7, 1.5, CH_{Ar}), 7.00 (1H, brs, NH), 7.06 (1H, dd, J 8.0, 1.5, CH_{Ar}), 7.18 (1H, td, J 7.8, 1.5, CH_{Ar}) and 7.36 (1H, dd, J 8.0, 1.4, CH_{Ar}); δ_C (126 MHz, $CDCl_3$) 14.8 (Me), 25.0 (allylic CH_2), 33.2 (NCH_2*C*H$_2$), 34.3 (N*C*H$_2$Me), 40.0 (CH_2*C*HCH_2), 50.9 (N*C*H$_2$$CH_2$), 73.0 (*C*q-CONHEt), 123.8 (CH_{Ar}), 124.1 (=*C*H-CHN), 124.2 (CH_{Ar}), 127.08 (CH_{Ar}), 127.11 Cq_{Ar}), 129.5 (CH_{Ar}), 130.1 (=*C*H-CH_2), 144.4 Cq_{Ar}), 157.6 (urea) and 173.4 (CONHEt); HRMS (ESI$^+$) 346.1321 ($C_{18}H_{21}ClN_3O_2$, $[M+H]^+$, requires 346.1317).

[3+2] Cycloadditions

General Experimental Procedure A: Palladium Catalysed [3+2] Annulations

To a solution of $Pd_2(dba)_3$ (3 mol%), in anhydrous, degassed, dichloromethane (0.2 M w.r.t aziridine) was charged $P(OPh)_3$ (0.25 eq). The resultant pale green solution was stirred under a N_2 atmosphere for 20 mins before addition of tetra-butyl ammonium iodide (0.1 eq) and dipolarophile (2-4 eq). After stirring at ambient temperature for a further 20 mins, aziridine (1 eq) was added and the reaction heated to 36 °C for 16 h. After concentration to dryness, purification was carried out by column chromatography (100% CH_2Cl_2 then gradient of 5% EtOAc/CH_2Cl_2 to 50% EtOAc/ CH_2Cl_2 unless otherwise stated), to give cyclised products.

General Experimental Procedure B: Catalyst Free Reactions Annulations

To a solution of aziridine (100 mol%) in anhydrous MeCN (0.25 M) was added dipolarophile (120 mol%), the reaction mixture was stirred at room temperature until no starting material was observed by TLC. The reaction mixture was then concentrated to dryness and purified by column chromatography.

(±)-(3R,5a*S*)-1,1-Dicyano-*N*-ethyl-2-phenyl-1,4,5,5a,6,8a-hexahydropyrrolo[3,2,1-hi]indole-3(2*H*)-carboxamide 10a

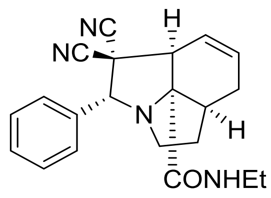

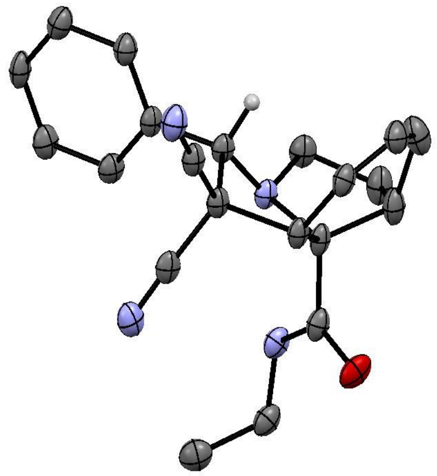

General procedure A for Pd catalysed [3+2] annulation reactions was followed, using benzylidene malononitrile (114 mg, 0.50 mmol) as the dipolarophile and amide aziridine **3a** (48.0 mg, 0.25 mmol) in anhydrous dichloromethane (1.25 mL) to yield the title compound **10a** (45.0 mg, 53%, 22:1 d.r.) as a colourless crystalline solid. M.p 152-154 °C (EtOAc/Pet). υ_{max}/cm^{-1} (film) 3341 m (N-H) 2973 w (C-H), 2255 w (CN), 1647 s (C=O). ^{1}H NMR (400 MHz, $CDCl_3$) δ_H 7.66 (1H, t, *J* = 5.0 Hz, CON*H*Et), 7.58 –

7.54 (2H, m, ArC*H*), 7.49-7.46 (3H, m, ArC*H*), 6.34 (1H, dtd, *J* = 10.0, 4.5, 2.5 Hz, CH=C*H*-CH_2), 6.03 (1H, ddt, *J* = 10.0, 4.0, 2.0 Hz, C*H*=CH-CH_2), 4.11 (1H, s, N-C*H*-Ph), 3.65-3.51 (1H, m, C*H*-CH=CH), 3.45 (1H, dq, *J* = 13.0, 7.0 Hz, CONHCH_{2a}), 3.32 (1H, dq, *J* = 13.0, 7.0 Hz, CONHCH_{2b}), 3.01 (1H, dt, *J* = 12.0, 6.5 Hz, N-CH_{2a}-CH_2), 2.73– 2.64 (2H, m, N-CH_{2b}-CH_2, CH_2-C*H*-CH_2), 2.49 (1H, dddt, *J* = 16.5, 7.0, 4.5, 2.0 Hz, CH=CH-CH_{2a}), 2.04- 1.93 (2H, m, N-CH_2-CH_{2a}, CH=CH-CH_{2b}), 1.78 (1H, dt, *J* = 13.0, 6.5 Hz, N-CH_2-CH_{2b}), 1.25 (3H, t, *J* = 7.0 Hz, CONHCH_2CH_3). ^{13}C NMR (101 MHz, $CDCl_3$) δc 174.1 (C=O), 133.1 (Ar*C*), 133.0 (CH=*C*H-CH_2), 130.1 (Ar*C*H), 129.1 (Ar*C*H), 127.7 (Ar*C*H), 122.1 (CH-*C*H=CH), 113.6 (*C*N), 112.1 (*C*N), 76.3 (N-*C*-CONHEt), 74.1 (N-*C*H-Ph), 49.9 (N-*C*H_2-CH_2), 48.2 (N-CH-*C*-CN), 47.1 (*C*H-CH=CH), 40.6 (CH_2-*C*H-CH_2), 34.6 (CONH*C*H_2), 32.6 (N-CH_2-*C*H_2), 25.9 (CH=CH-*C*H_2), 14.5 (CONHCH_2*C*H_3). m/z (CI+) 347.2 ([M+H+]). HRMS: (ESI+) calculated for $C_{21}H_{23}N_4O$: 347.1871. Found (M+H+) 347.1866.

The stereochemistry of the major diastereomer of this compound was confirmed by single crystal X-ray diffraction (see above) of crystals grown from EtOAc/hexane.

(±)-1-(*Tert*-butyl) 1-methyl (3*R*,8a*R*)-3-(ethylcarbamoyl)-31,4,5,5a,6,8a-hexahydropyrrolo[3,2,1-hi]indole-1,1(2*H*)-dicarboxylate 11a

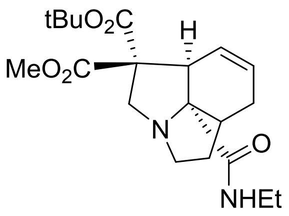

To a solution of paraformaldehyde (1.77 g, 59.1 mmol), copper acetate monohydrate (0.29 g, 1.48 mmol) and potassium acetate (0.29 g, 2.96 mmol) in acetic acid (10.0 mL) was added *tert*-butyl methyl malonate (5.00 mL, 29.6 mmol). The reaction was heated at 120 °C for 2 h under nitrogen before cooling to ambient temperature. Acetic acid was removed under reduced pressure and the resultant blue residue diluted with water (30 mL) before extraction using ethyl acetate (2 x 30 mL). The combined organic extracts were washed with saturated $NaHCO_3$ solution (5 x 75 mL), brine, dried ($MgSO_4$) and concentrated to dryness. Purification by vacuum distillation (b.p 65 °C – 70 °C at 3.5 mmHg) yielded the title compound as a mixture with polymeric material (0.91 g) which was used without further purification. To a solution of $Pd_2(dba)_3$ (7.00 mg, 7.60 µmol) in anhydrous, degassed dichloromethane (1.25 mL) was charged $P(OPh)_3$ (0.02 mL, 6.00 µmol). The resultant pale green solution was stirred under a N_2 atmosphere for 20 mins before addition of tetra-butyl ammonium iodide (9.00 mg, 0.025 mmol) and crude *tert*-butyl methyl 2-methylenemalonate (186 mg). After stirring at ambient temperature for a further 20 mins, amide aziridine **3a** (48.0 mg, 0.25 mmol) was

added and the reaction heated to 36 °C for 16 h. After concentration to dryness, purification was carried out by column chromatography (100% CH_2Cl_2 then a gradient of 5% EtOAc/CH_2Cl_2 to 10% EtOAc/CH_2Cl_2), to give the title compound **11a** (83.0 mg, 88%) as an inseparable mixture of diastereomers (1:1.1 dr). υ_{max}/cm^{-1} (film) 2952 w (C-H), 1732 s (C=O), 1695s (C=O). ^{1}H NMR (400 MHz, $CDCl_3$) δ_H 7.38 (1H, brs, NH, both diastereomers), 6.02 – 5.96 (1H, m, CH=C*H*-CH_2, both diastereomers), 5.57 (1H, ddt, *J* = 10.0, 3.5, 2.0 Hz, CH-C*H*=CH, minor diastereomer), 5.42 (1H, ddt, *J* = 10.0, 3.5, 2.0 Hz, CH-C*H*=CH, major diastereomer), 3.71-3.63 (4H, m, C*H*-CH=CH, CO_2-CH_3, both diastereomers), 3.45 (1H, d, *J* = 9.5 Hz, N-CH_{2a}-C, both diastereomers), 3.36 -3.10 (2H, m, CONH-CH_2-CH_3, both diastereomers), 3.05 (1H, d, *J* = 9.5 Hz, N-CH_{2b}-C, major diastereomer), 2.99 (1H, d, *J* = 9.5 Hz, N-CH_{2b}-C, minor diastereomer), 2.97- 2.88 (1H, m, N-CH_{2a}-CH_2, both diastereomers), 2.78-2.70 (1H, m, N-CH_{2b}-CH_2, both diastereomers), 2.44-2.20 (2H, m, CH=CH-CH_{2a}, CH=CH-CH_2-C*H*, both diastereomers), 1.88- 1.64 (3H, m, CH=CH-CH_{2b}, N-CH_2-CH_2, both diastereomers), 1.40 (9H, s, C-(CH_3)$_3$, major diastereomer), 1.39 (9H, s, C-(CH_3)$_3$, minor diastereomer), 1.09 (3H, t, *J* = 7.5 Hz, CONH-CH_2-CH_3, both diastereomers).^{13}C NMR (101 MHz, $CDCl_3$) δ 176.4 (*C*ONHEt, minor diastereomer), 176.3 (*C*ONHEt diastereomer), 170.7 (*C*O_2Me, minor diastereomer), 168.7 (*C*O_2Me, major diastereomer), 168.5 (*C*O_2tBu, major diastereomer), 166.6 (*C*O_2tBu, minor diastereomer), 129.4 (CH=C*H*-CH_2, both diastereomers), 124.7 (C*H*=CH-CH_2, major diastereomer), 124.4 (C*H*=CH-CH_2, minor diastereomer), 82.3 (*C*-(CH_3)$_3$ minor diastereomer), 81.8 (*C*-(CH_3)$_3$, major diastereomer), 78.0 (N-*C*-CO, major diastereomer), 77.9 (N-*C*-CO, minor diastereomer), 66.3 (N-CH_2-*C*, major diastereomer), 66.3 (N-CH_2-*C*, minor diastereomer), 59.7 (N-*C*H_2-C, major diastereomer), 59.2 (N-*C*H_2-C, minor diastereomer), 52.7 (CO_2-*C*H_3, minor diastereomer), 52.2 (CO_2-*C*H_3, major diastereomer), 51.8 (N-*C*H_2-CH_2, major diastereomer), 51.6 (N-*C*H_2-CH_2, minor diastereomer), 43.6 (N-C-*C*H-CH=CH, minor diastereomer), 43.4 (N-C-*C*H-CH=CH, major diastereomer), 40.9 (CH_2-*C*H-CH_2, major diastereomer), 40.8 (CH_2-*C*H-CH_2-, minor diastereomer), 33.9 (CONH-*C*H_2, both diastereomers), 32.4 (N-CH_2-*C*H_2, both diastereomers), 27.8 (C-(*C*H_3)$_3$ minor diastereomer), 27.7 (C-(*C*H_3)$_3$, major diastereomer), 25.8 (CH=CH-*C*H_2, both diastereomers), 14.8 (CONH-CH_2-*C*H_3, minor diastereomer).14.7 (CONH-CH_2-*C*H_3, major diastereomer). m/z (ESI+) 379.222 (M+H+), HRMS: (ESI+) calculated for $C_{20}H_{31}N_2O_5$: 379.2227. Found (M+H+) 379.2220.

Di-*tert*-butyl 2-methylenemalonate

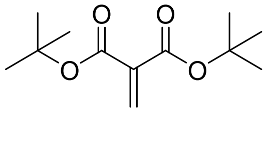

This compound was synthesized following an adapted literature procedure.[3] To a solution of paraformaldehyde (1.39 g, 46.2 mmol), copper acetate monohydrate (0.23 g, 1.16 mmol) and potassium acetate (0.23 g, 2.31 mmol) in acetic acid (10.0 mL) was added di-*tert*-butyl malonate (5.00 g, 23.1 mmol). The reaction was heated at 100 °C for 2 h under nitrogen before cooling to ambient temperature. Acetic acid was removed under reduced pressure and the resultant blue residue diluted with water (50 mL) before extraction using ethyl acetate (2 x 50 mL). The combined organic extracts were washed with saturated $NaHCO_3$ solution (4 x 75 mL), brine, dried ($MgSO_4$) and concentrated to dryness. Purification by vacuum distillation (b.p 78 °C – 80 °C at 5 mmHg) yielded the title compound (1.06 g, 20%) as a colourless oil. The spectroscopic properties of this compound were consistent with those available in the literature.[1] υ_{max}/cm^{-1} (film) 2979 w (C-H), 1718 s (C=O). ^1H NMR (400 MHz, $CDCl_3$) δ_H 6.23 (2H, s, C=C*H*$_2$), 1.50 (18H, s, C*H*$_3$). ^{13}C NMR (101 MHz, $CDCl_3$) δ_C 163.7 (*C*=O), 138.3 (*C*H$_2$), 130.8 (*C*=CH$_2$), 81.9 (*C*-CH$_3$), 28.0 (CH$_3$).

(±)-Di-*tert*-butyl (3*R*,8a*R*)-3-(ethylcarbamoyl)-31,4,5,5a,6,8a-hexahydropyrrolo[3,2,1-hi]indole-1,1 (2*H*)-dicarboxylate 12a

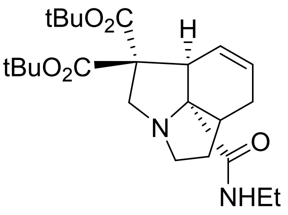

General procedure A for Pd catalysed [3+2] annulation reactions was followed, using di-*tert*-butyl 2-methylenemalonate (114 mg, 0.50 mmol) as the dipolarophile and amide aziridine **3a** (48.0 mg, 0.25 mmol) in anhydrous dichloromethane (1.25 mL) to yield the title compound **12a** (83.0 mg, 79%) as a pale yellow, crystalline solid. Mp. 85 – 86 °C (EtOAc/hex). υ_{max}/cm^{-1} (film) 2984 w (C-H), 1726 s (C=O). ^1H NMR (400 MHz, $CDCl_3$) δ_H 7.35 (1H, t, *J* = 5.5 Hz, N*H*), 5.97 (1H, dtd, *J* = 10.5, 4.5, 2.5 Hz, CH=C*H*-CH$_2$), 5.56 (1H, ddt, *J* = 10.5, 3.5, 2.0 Hz, CH-C*H*=CH), 3.64 (1H, d, *J* = 2.0 Hz, C*H*-CH=CH), 3.40 (1H, d, *J* = 9.5 Hz, N-C*H*$_{2a}$-C), 3.28 (1H, dq, *J* = 14.0, 7.0, CONH-C*H*$_{2a}$-CH$_3$), 3.17 (1H, dq, *J* = 14.0, 7.0, CONH-C*H*$_{2b}$-CH$_3$), 3.01 (1H, d, *J* = 9.5 Hz, N-C*H*$_{2b}$-C), 2.92 (1H, dt, *J* = 11.5, 7.0 Hz, N-C*H*$_{2a}$-CH$_2$), 2.73 (1H, dt, *J* = 11.5, 6.5 Hz, N-C*H*$_{2b}$-CH$_2$), 2.35 (2H, m, CH=CH-C*H*$_{2a,}$ CH=CH-CH$_2$-C*H*), 1.90 – 1.81 (1H, m, CH=CH-C*H*$_{2b}$), 1.79 - 1.65 (2H, m, N-CH$_2$-C*H*$_2$), 1.44 (9H, s, C-(C*H*$_3$)$_3$), 1.43 (9H, s, C-(C*H*$_3$)$_3$), 1.11 (3H, t, *J* = 7.0 Hz, CONH-CH$_2$-C*H*$_3$). ^{13}C NMR (101 MHz, $CDCl_3$) δ 176.7 (N*C*=O), 168.9 (*C*OOtBu), 167.1 (*C*OOtBu), 129.1 (CH=*C*H-CH$_2$), 124.7 (*C*H=CH-CH$_2$), 81.8 (*C*-(CH$_3$)$_3$), 81.3 (*C*-(CH$_3$)$_3$), 78.0 (N-*C*-C=O), 66.5 (N-CH$_2$-*C*), 59.8 (N-*C*H$_2$-C), 51.8 (N-*C*H$_2$-CH$_2$), 43.2 (*C*H-CH=CH), 41.3 (CH=CH-CH$_2$-*C*H), 33.9 (CONH-*C*H$_2$), 32.2 (N-CH$_2$-*C*H$_2$), 27.7 (C-(*C*H$_3$)$_3$), 25.7 (CH=CH-*C*H$_2$), 14.7 (CONH-CH$_2$-CH$_3$). m/z (CI+) 421.268 (M+H+),

HRMS: (CI+) calculated for $C_{23}H_{37}N_2O_5$: 421.2697. Found (M+H+) 421.2682. Anal. Calcd. for C, 65.69; H, 8.63; N, 6.66. Found C, 65.42; H, 8.43; N, 6.99.

(±)-Di-*tert*-butyl (3*R*,8a*R*)-3-acetyl-31,4,5,5a,6,8a-hexahydropyrrolo[3,2,1-hi]indole-1,1(2H)-dicarboxylate 12b

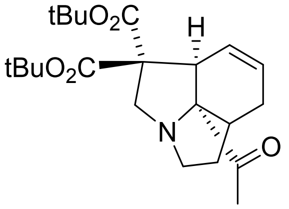

General procedure A for Pd catalysed [3+2] annulation reactions was followed, using Di-*tert*-butyl 2-methylenemalonate (456 mg, 2.00 mmol) as the dipolarophile and ketone aziridine **3b** (163 mg, 1.00 mmol) in anhydrous dichloromethane (5.00 mL) to yield the title compound **12b** (163 mg, 42%) as a colourless oil. υ_{max}/cm^{-1} (film) 2979 w (C-H), 1723 s (C=O). ^{1}H NMR (400 MHz, $CDCl_3$) δ_H 5.94 (1H, ddt, *J* = 10.0, 6.0, 3.0 Hz, CH=C*H*-CH_2), 5.51(1H, dtd, *J* = 10.0, 2.5, 1.0 Hz, CH-C*H*=CH), 3.54 (1H, d, *J* = 3.0 Hz, C-C*H*-CH=CH), 3.35 (1H, dd, *J* = 9.0, 1.0 Hz, N-CH_{2a}-C), 3.01 (1H, d, *J* = 9.0Hz, N-CH_{2b}-C), 2.96 – 2.76 (2H, m, N-C*H*$_2$-CH_2), 2.25 – 2.16 (4H, m, COC*H*$_3$, CH=CH-C*H*$_{2a,}$), 2.13- 2.06 (1H, dddd, *J* = 12.5, 10.0, 6.0, 3.5 Hz, CH=CH-CH_2-C*H*), 1.97 (1H, dtt, *J* = 16.0, 4.5, 1.0 Hz, CH=CH-C*H*$_{2b}$), 1.76 – 1.59 (2H, m, N-CH_2-C*H*$_2$), 1.43 (18H, s, C-(C*H*$_3$)$_3$). ^{13}C NMR (101 MHz, $CDCl_3$) δ 215.0 (*C*OMe), 169.6 (*C*OOtBu), 167.4 (*C*OOtBu), 128.7 (CH=*C*H-CH_2), 124.9 (*C*H=CH-CH_2), 82.4 (N-*C*-C=O), 81.8 (*C*-(CH_3)$_3$), 81.6 (*C*-(CH_3)$_3$), 65.4 (N-CH_2-*C*), 59.8 (N-*C*H$_2$-C), 52.4 (N-*C*H$_2$-CH_2), 43.7 (*C*H-CH=CH), 41.87 (CH=CH-CH_2-*C*H), 30.9 (N-CH_2-*C*H$_2$), 27.8 (C-(*C*H$_3$)$_3$), 27.7 (C-(*C*H$_3$)$_3$), 25.1 (CO-*C*H$_3$), 24.6 (CH=CH-*C*H$_2$). m/z (ESI+) 392.244 (M+H+), HRMS: (ESI+) calculated for $C_{22}H_{34}NO_5$: 392.2432. Found (M+H+) 392.2442.

(±)- (3*R*,8a*S*)-*N*-ethyl-5,5a,6,8a-tetrahydro-2*H*-oxazolo[5,4,3-hi]indole-31(4*H*)-carboxamide 13a

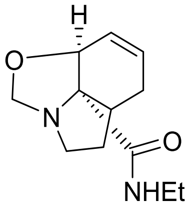

General procedure A for Pd catalysed [3+2] annulation reactions was followed, using paraformaldehyde (0.016 g, 0.5 mmol) as the dipolarophile and amide aziridine **3a** (96.0 mg, 0.25 mmol) in anhydrous dichloromethane (1.25 mL). Purification by column chromatography (20%

EtOAc/ CH_2Cl_2) gave the title compound **13a** (43.0 mg, 77%) as a yellow solid. Mp. 95- 97 °C (CH_2Cl_2). υ_{max}/cm^{-1} (film) 3328 w (N-H), 2961 w (C-H), 2890 w (C-H), 1641 s (C=O) 1517 m (C=C).^{1}H NMR (400 MHz, $CDCl_3$) δ_H 7.59 (1H, brs, N-*H*), 6.00 (1H, dtd, *J* = 10.5, 2.5, 1.0 Hz, O-CH-CH=C*H*), 5.91 (1H, ddt, *J* = 10.5, 6.0, 1.5 Hz, CH-C*H*=CH), 4.51 (1H, d, *J* = 7.0 Hz, N-CH_{2a}-O), 4.18 (1H, d, *J* = 7.0 Hz, N-CH_{2b}-O), 3.95 (1H, s, O-C*H*-CH=CH), 3.32 -3.21 (3H, m, CONH-CH_2, N-CH_{2a}-CH_2), 2.89 (1H, ddd, *J* = 11.0, 8.5, 2.0 Hz, N-CH_{2b}-CH_2), 2.59 (1H, dddd, *J* = 17.5, 6.0, 5.0, 2.5 Hz, CH=CH-CH_{2a}), 2.42 (1H, ddt, *J* = 11.5, 6.0, 2.0 Hz, CH_2-C*H*-CH_2), 2.18 (2H, ddt, *J* = 17.5, 6.0, 2.0 Hz, CH=CH-CH_{2b}), 1.81-1.59 (2H, m, N-CH_2-CH_2), 1.13 (3H, t, *J* = 7.0 Hz, CONHCH_2-CH_3). ^{13}C NMR (101 MHz, $CDCl_3$) δ_c 174.2 (*C*=O), 128.1 (O-CH-*C*H=CH), 123.7 (O-CH-CH=*C*H), 87.7 (N-*C*H_2-O), 75.5 (N-*C*-CONHEt), 73.6 (O-*C*H-CH=CH), 56.0 (N-*C*H_2-CH_2), 40.1 (CH_2-*C*H-CH_2), 33.5 (CONH-*C*H_2-CH_3), 32.4 (N-CH_2-*C*H_2), 26.3 (CH=CH-*C*H_2), 14.9 (CONHCH_2-*C*H_3). m/z (ESI+) 245.146 (M+Na+), HRMS: (ESI+) calculated for $C_{12}H_{19}N_2O_2$: 223.1441. Found (M+H+) 223.1440. Anal. Calcd. for C, 64.84; H, 8.16; N, 12.60. Found C, 64.38; H, 8.29; N, 11.96.

(±)-1-((31R,8aS)-5,5a,6,8a-Tetrahydro-2H-oxazolo[5,4,3-hi]indol-31(4H)-yl)ethan-1-one 13b

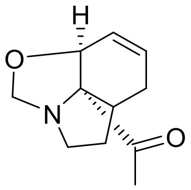

General procedure A for Pd catalysed [3+2] annulation reactions was followed, using paraformaldehyde (16.0 mg, 0.50 mmol) as the dipolarophile and ketone aziridine **3b** (41.0 mg, 0.25 mmol) in anhydrous dichloromethane (1.25 mL) to yield the title compound **13b** (25.0 mg, 52%) as a yellow oil. υ_{max}/cm^{-1} (film) 2958 w (C-H), 2870 w (C-H) 1700 s (C=O). ^{1}H NMR (400 MHz, $CDCl_3$) δ_H 6.01 (1H, ddt, *J* = 10.0, 4.0, 2.0 Hz, O-CH-C*H*=CH), 5.83 (1H, dt, *J* = 10.0, 5.0 Hz, O-CH-CH=C*H*), 4.53 (1H, d, *J* = 7.5 Hz, N-CH_{2a}), 4.19 (1H, d, *J* = 7.5 Hz, N-CH_{2b}), 4.04 (1H, s, O-C*H*-CH=CH), 3.38 (1H, td, *J* = 11.0, 6.0 Hz, N-CH_{2a}-CH_2), 2.95 (1H, ddd, *J* = 11.0, 6.0, 2.0 Hz, N-CH_{2b}), 2.45 (1H, ddt, *J* = 15.0, 7.5, 4.0 Hz, CH_2-C*H*-CH_2), 2.31 (3H, s, CO-CH_3), 2.22 – 2.18 (2H, m, CH=CH-CH_2), 1.81- 1.63 (2H, m, N-CH_2-CH_2). ^{13}C NMR (101 MHz, $CDCl_3$) δ_c 212.9 (*C*=O), 127.0 (O-CH-CH=*C*H), 124.1 (O-CH-*C*H=CH), 88.3 (N-*C*H_2-O), 80.3 (N-*C*-C=O), 71.8 (O-*C*H-CH=CH), 57.0 (N-*C*H_2-CH_2), 38.8 (CH_2-*C*H-CH_2), 31.9 (N-CH_2-*C*H_2), 25.9 (CO-*C*H_3), 25.21 (CH=CH-*C*H_2).m/z (ESI+) 194.118 (M+H+), HRMS: (ESI+) calculated for $C_{11}H_{16}NO_2$: 194.1176. Found (M+H+) 194.1179.

(±)-(3*R*,8a*S*)-*N*-Ethyl-2-methyl-5,5a,6,8a-tetrahydro-2*H*-oxazolo[5,4,3-hi]indole-31(4*H*)-carboxamide 14a

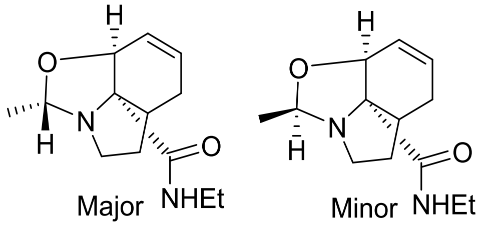

General procedure A for Pd catalysed [3+2] annulation reactions was followed, using freshly distilled acetaldehyde (0.06 mL, 0.5 mmol) as the dipolarophile and amide aziridine **3a** (48.0 mg, 0.25 mmol) in anhydrous dichloromethane (1.25 mL) to yield the title compound **14a** (51.0 mg, 86%) as a partially separable mixture of diastereomers (d.r. 1.8:1), as a brown oil.

Major Diastereomer

υ_{max}/cm^{-1} (film) 3348 w (N-H), 2971 w (C-H), 2873 w (C-H), 1657 s (C=O) 1510 m (C=C). ^{1}H NMR (400 MHz, $CDCl_3$) δ_H 7.70 (1H, brs, NH), 5.96 (1H, td, *J* = 9.5, 3.0 Hz, CH=C*H*-CH_2), 5.80 (1H, dt, *J* = 9.5, 2.0 Hz, C*H*=CH-CH_2), 4.48 (1H, d, *J* = 2.0 Hz O-C*H*-CH=CH), 4.42 (1H, q, *J* = 5.5 Hz, N-C*H*-O), 3.34- 3.18 (2H, m, CO NHCH_2,), 2.93 (1H, td, *J* = 12.5, 8.4 Hz, N-CH_{2a}), 2.77 (1H, ddd, *J* = 12.5, 6.0, 3.5 Hz, N-CH_{2b}), 2.55 – 2.43 (2H, m, CH=CH-C$H_{2a,}$ CH_2-C*H*-CH_2), 2.15 (1H, dd, *J* = 15.5, 6.0 Hz, CH=CH-CH_{2b}), 1.74 – 1.67 (2H, m, N-CH_2-CH_2), 1.27 (3H, d, *J* = 5.5 Hz, N-CH-CH_3),1.14 (3H, t, *J* = 7.0 Hz, CONHCH_2CH_3). ^{13}C NMR (101 MHz, $CDCl_3$) δ 175.9 (*C*ONHEt), 129.0 (CH=*C*H-CH_2), 126.3 (*C*H=CH-CH_2), 97.5 (N-*C*-CONHEt), 93.4 (N-*C*H-O), 74.4 (O-*C*H-CH=CH), 51.2 (N-*C*H_2-CH_2), 40.7 (CH_2-*C*H-CH_2), 33.7 (CONH-*C*H_2-CH_3), 32.5 (N-CH_2-*C*H_2), 25.8 (CH=CH-*C*H_2), 21.0 (N-CH-*C*H_3), 14.9 (CONHCH_2-*C*H_3). m/z (ESI+) 259.142 (M+Na+), HRMS: (ESI+) calculated for $C_{13}H_{21}N_2O_2$: 237.1598. Found (M+H+) 237.1600. ^{1}H NOE showed no enhancement signal between N-C*H*-CH_3 and O-C*H*-CH=CH, consistent with *anti*-stereochemistry of the major diastereomer of the title compound.

Minor Diastereomer

υ_{max}/cm^{-1} (film) 3348 w (N-H), 2971 w (C-H), 2873 w (C-H), 1657 s (C=O) 1510 m (C=C). ^{1}H NMR (400 MHz, $CDCl_3$) δ_H 7.60 (1H, brs, NH), 6.01 – 5.88 (2H, m, C*H*=C*H*-CH_2), 4.41 (1H, q, *J* = 6.0 Hz, N-C*H*-O), 4.08 (1H, s, O-C*H*-CH=CH), 3.30 – 3.15 (3H, m, CONHCH_2, N-CH_{2a}), 2.84 (1H, td, *J* = 9.5, 6.5 Hz, N-CH_{2b}), 2.55 – 2.38 (2H, m, CH=CH-C$H_{2a,}$ CH_2-C*H*-CH_2), 2.13 (1H, ddd, *J* = 16.5, 4.0, 3.0 Hz, CH=CH-

CH_{2b}), 1.77 – 1.54 (2H, m, N-CH_2-CH_2), 1.42 (3H, d, *J* = 6.0 Hz, N-CH-CH_3), 1.14 (3H, t, *J* = 7.5 Hz, CONHCH_2CH_3). ^{13}C NMR (101 MHz, $CDCl_3$) δ 174.6 (*C*ONHEt), 128.2 (CH=*C*H-CH_2), 124.5 (*C*H=CH-CH_2), 88.9 (N-*C*H-O), 76.9 (N-*C*-CONHEt), 73.5 (N-C-*C*H-CH=CH), 45.4 (N-*C*H_2-CH_2), 40.2 (CH_2-*C*H-CH_2), 33.5 (CONH-*C*H_2-CH_3), 32.2 (N-CH_2-*C*H_2), 26.4 (CH=CH-*C*H_2), 15.9 (N-CH-*C*H_3), 14.9 (CONHCH_2-*C*H_3). m/z (ESI+) 259.142 (M+Na+), HRMS: (ESI+) calculated for $C_{13}H_{21}N_2O_2$: 237.1598. Found (M+H+) 237.1600. ^{1}H NOE enhancement signal between N-C*H*-CH_3 and O-C*H*-CH=CH, confirmed the *syn*-stereochemistry conformation of the minor diastereomer of the title compound.

(±)-1-((3*R*,8a*S*)-2-Methyl-5,5a,6,8a-tetrahydro-2*H*-oxazolo[5,4,3-hi]indol-31(4*H*)-yl)ethan-1-one 14b

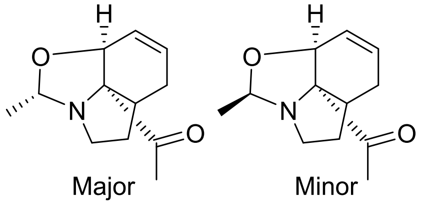

General procedure A for Pd catalysed [3+2] annulation reactions was followed, using freshly distilled acetaldehyde (0.06 mL, 1.00 mmol) as the dipolarophile and ketone aziridine **3b** (41.0 mg, 0.25 mmol) in anhydrous dichloromethane (1.25 mL). Purification by column chromatography (10% EtOAc/CH_2Cl_2 to 20 % EtOAc/CH_2Cl_2) yielded the title compound **14b** (45.0 mg, 87%) as separable mixture of diastereomers (1:1.5 dr) and as a yellow oil.

Major Diastereomer

υ_{max}/cm^{-1} (film) 2929 w (C-H), 2873 w (C-H) 1703 s (C=O). ^{1}H NMR (400 MHz, $CDCl_3$) δ_H 5.89-5.80 (2H, m, C*H*=C*H*), 4.54 (1H, q, *J* = 5.5 Hz, N-C*H*-O), 4.47 (1H, s, O-C*H*-CH=CH), 3.10 (1H, td, *J* = 12.0, 6.0 Hz, N-CH_{2a}-CH_2), 2.86 (1H, ddd, *J* = 12.0, 7.5, 2.0 Hz, N-CH_{2b}-CH_2), 2.46 (1H, ddd, *J* = 14.0, 7.5, 4.0 Hz, CH_2-C*H*-CH_2), 2.34 (3H, s, CO-CH_3), 2.16-2.11 (2H, m, CH=CH-CH_2), 1.80 – 1.60 (2H, m, N-CH_2-CH_2), 1.21 (3H, d, *J* = 5.5 Hz, N-CH-CH_3). ^{13}C NMR (101 MHz, $CDCl_3$) δ_c 213.5 (*C*=O), 127.2 (O-CH-*C*H=CH), 126.5 (O-CH-CH=*C*H), 94.7 (N-*C*H-O), 81.2 (N-*C*-COMe), 72.0 (O-*C*H-CH=CH), 53.5 (N-*C*H_2-CH_2), 38.9 (CH_2-*C*H-CH_2), 31.5 (N-CH_2-*C*H_2), 25.5 (CO-*C*H_3), 24.7 (CH=CH-*C*H_2), 21.1 (N-CH-*C*H_3). m/z (ESI+) 208.133 (M+H+), HRMS: (ESI+) calculated for $C_{12}H_{18}NO_2$: 208.1332. Found (M+H+) 208.1333. ^{1}H NOE showed no enhancement signal between N-C*H*-CH_3 and O-C*H*-CH=CH, consistent with *anti*-stereochemistry of the major diastereomer of the title compound.

Minor Diastereomer

υ_{max}/cm^{-1} (film) 2929 w (C-H), 2873 w (C-H) 1703 s (C=O). ^{1}H NMR (400 MHz, $CDCl_3$) δ_H 5.98 (1H, ddt, *J* = 10.0, 3.5, 2.0 Hz, O-CH-C*H*=CH), 5.86 -5.79 (1H, m, O-CH-CH=C*H*), 4.40 (1H, q, *J* = 6.0 Hz, N-C*H*-O), 4.18 (1H, ddd, *J* = 5.0, 3.5, 1.5 Hz, O-C*H*-CH=CH), 3.10 (1H, ddd, *J* = 12.0, 8.0, 4.0 Hz, N-CH_{2a}-CH_2), 2.92 (1H, ddd, *J* = 12.0, 9.0, 6.5 Hz, N-CH_{2b}-CH_2), 2.43 (1H, ddd, *J* = 14.5, 8.5, 4.0 Hz, CH_2-C*H*-CH_2), 2.30 (3H, s, CO-CH_3), 2.17-2.13 (2H, m, CH=CH-CH_2), 1.80 (1H, dtd, *J* = 11.5, 6.5, 4.0, N-CH_2-CH_{2a}), 1.65-1.55 (1H, m, N-CH_2-CH_{2b}), 1.41 (3H, d, *J* = 6.0 Hz, N-CH-CH_3). ^{13}C NMR (101 MHz, $CDCl_3$) δ_c 213.1 (*C*=O), 126.7 (O-CH-*C*H=CH), 125.5 (O-CH-CH=*C*H), 89.6 (N-*C*H-O), 82.0 (N-*C*-COMe), 71.6 (O-*C*H-CH=CH), 46.1 (N-CH_2-CH_2), 38.3 (CH_2-*C*H-CH_2), 31.8 (N-CH_2-CH_2), 25.6 (CO-CH_3), 25.4 (CH=CH-CH_2), 16.1 (N-CH-CH_3). m/z (ESI+) 208.133 (M+H+), HRMS: (ESI+) calculated for $C_{12}H_{18}NO_2$: 208.1332. Found (M+H+) 208.1333. ^{1}H NOE enhancement signal between N-C*H*-CH_3 and O-C*H*-CH=CH, confirmed the *syn*- stereochemistry conformation of the minor diastereomer of the title compound.

(±)-Ethyl (3*R*,8a*S*)-3-(ethylcarbamoyl)-3,4,5,5a,6,8a-hexahydro-2*H*-oxazolo[5,4,3-hi]indole-2-carboxylate 15a

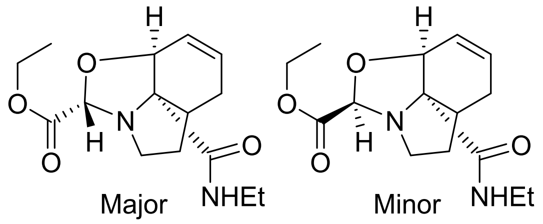

General procedure A for Pd catalysed [3+2] annulation reactions was followed, using ethyl glyoxalate (50% in toluene, 0.10 mL) as the dipolarophile and amide aziridine **3a** (48.0 mg, 0.25 mmol) in anhydrous dichloromethane (1.25 mL) to yield the title oxazolidine **15a** (58.0 mg, 80%) as a partially separable mixture of diastereomers (d.r. 4:1), and as colourless solids.

Major Diastereomer

Mp 100 – 101 °C (EtOH). υ_{max}/cm^{-1} (film) 3365 w (N-H), 2975 w (C-H), 1744 s (C=O, ketone), 1665 s (C=O, amide), 1518 (C=C). ^{1}H NMR (400 MHz, $CDCl_3$) δ_H 7.45 (1H, brs, NH), 5.97- 5.90 (2H, m, C*H*=C*H*), 4.87 (1H, s, N-C*H*-O), 4.59 (1H, dd, *J* = 4.0, 2.0 Hz, O-C*H*-CH=CH), 4.16 (2H, qd, *J* = 7.0, 3.0 Hz, COOCH_2), 3.39 – 3.09 (3H, m, CONHCH_2, N-CH_{2a}), 3.00 (1H, ddd, *J* = 11.5, 7.5, 3.0 Hz, N-CH_{2b}), 2.54 (1H, ddt, *J* = 16.5, 6.5, 1.5 Hz, CH=CH-CH_{2a}), 2.43 (1H, ddd, *J* = 10.5, 6.5, 4.5 Hz, CH_2-C*H*-CH_2), 2.17 (1H, ddd, *J* = 16.5, 4.5, 2.5 Hz, CH=CH-CH_{2b}), 1.78 – 1.63 (2H, m, N-CH_2-CH_2), 1.26 (3H, t, *J* = 7.0 Hz, COCH_2-CH_3), 1.10 (3H, t, *J* = 7.0 Hz, CONHCH_2CH_3). ^{13}C NMR (101 MHz, $CDCl_3$) δ_c 174.2 (*C*ONHEt), 169.8 (*C*OOCH_2CH_3), 128.2 (CH=*C*H-CH_2), 124.0 (*C*H=CH-CH_2), 95.4 (N-*C*H-O), 75.9 (N-*C*-CONHEt), 74.7

(O-*C*H-CH=CH), 61.4 (COO*C*H_2), 56.5 (N-*C*H_2), 40.7 (CH_2-*C*H-CH_2), 33.7 (CONH*C*H_2), 32.0 (N-CH_2-*C*H_2), 25.9 (CH=CH-*C*H_2), 14.7 (CONHCH_2*C*H_3), 14.1 (COCH_2-*C*H_3). m/z (ESI+) 317.15 (M+Na+), HRMS: (ESI+) calculated for $C_{15}H_{23}N_2O_4$: 295.1652. Found (M+H+) 295.1647. [1]H NOE showed no enhancement signal between N-C*H*-CO, and O-C*H*-CH=CH, consistent with *anti*-stereochemistry of the major diastereomer of the title compound.

Minor Diastereomer

υ_{max}/cm^{-1} (film) 3365 w (N-H), 2975 w (C-H), 1744 s (C=O, ketone), 1665 s (C=O, amide), 1518 (C=C). ^{1}H NMR (400 MHz, $CDCl_3$) δ_H 7.59 (1H, brs, NH), 6.07 (2H, dddd, *J* = 10.5, 3.5, 2.5, 1.5 Hz, C*H*=CH-CH_2), 6.00 – 5.90 (1H, m, CH=C*H*-CH_2), 4.74 (1H, s, N-C*H*-O), 4.38- 4.29 (2H, m, COOC*H_2*), 4.16 (1H, s, O-C*H*-CH=CH), 3.33 – 3.19 (2H, m, CONHC*H_2*), 3.11 (1H, q, *J* = 10.0, 8.0 Hz, N-C*H_{2a}*), 2.96 – 2.87 (1H, m, N-C*H_{2b}*), 2.61– 2.39 (2H, m, CH=CH-C*H_{2a}*, CH_2-C*H*-CH_2), 2.17 (1H, dd, *J* = 16.5, 7.0 Hz, CH=CH-C*H_{2b}*), 1.77 – 1.69 (2H, m, N-CH_2-C*H_2*), 1.35 (3H, t, *J* = 7.0 Hz, COCH_2-C*H_3*), 1.15 (3H, t, *J* = 7.0 Hz, CONHCH_2C*H_3*). ^{13}C NMR (101 MHz, $CDCl_3$) δ_c 173.5 (*C*ONHEt), 166.4 (*C*OOCH_2CH_3), 128.4 (CH=*C*H-CH_2), 123.7 (*C*H=CH-CH_2), 90.9 (N-*C*H-O), 76.1 (N-*C*-CH), 73.6 (O-*C*H-CH=CH), 61.9 (COO*C*H_2), 50.1 (N-*C*H_2), 38.9 (CH_2-*C*H-CH_2), 33.6 (CONH*C*H_2), 31.9 (N-CH_2-*C*H_2), 26.1 (CH=CH-*C*H_2), 15.0 (CONHCH_2*C*H_3), 14.2 (COCH_2-*C*H_3). m/z (ESI+) 317.15 (M+Na+), HRMS: (ESI+) calculated for $C_{15}H_{23}N_2O_4$: 295.1652. Found (M+H+) 295.1647. [1]H NOE enhancement signal between N-C*H*-CO and O-C*H*-CH=CH, confirmed the *syn-* stereochemistry conformation of the minor diastereomer of the title compound.

(±)-Ethyl (3*R*,8a*S*)-3-acetyl-3,4,5,5a,6,8a-hexahydro-2*H*-oxazolo[5,4,3-hi]indole-2-carboxylate 15b

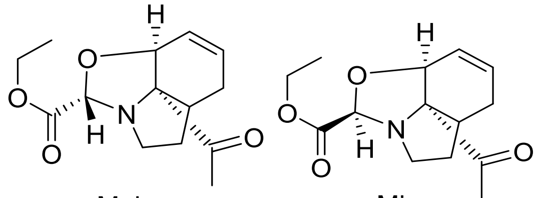

General procedure A for Pd catalysed [3+2] annulation reactions was followed, using ethyl glyoxalate (50 % in toluene, 0.10 mL) as the dipolarophile and ketone aziridine **3b** (41.0 mg, 0.25 mmol) in anhydrous dichloromethane (1.25 mL) to yield the title compound **15b** (51.0 mg, 77%) as a separable mixture of diastereomers (d.r. 5.6:1), and as colourless solids.

Major Diastereomer

Mp. 62-63 °C (CH_2Cl_2). υ_{max}/cm^{-1} (film) 2984 w (C-H), 2899 w (C-H) 1735 s (C=O, ester), 1702 s (C=O, ketone). ^{1}H NMR (400 MHz, $CDCl_3$) δ_H 5.97 (1H, ddt, *J* = 10.5, 3.5, 2.0 Hz, CH-CH=C*H*), 5.79 (1H, ddt, *J* = 10.5, 5.0, 2.0 Hz CH-C*H*=CH), 4.94 (1H, s, N-C*H*-O), 4.64 (1H, brs, O-C*H*-CH=CH), 4.13 (2H, qd, *J* = 7.0,

3.0 Hz, COOC*H*$_2$), 3.43 (1H, td, *J* = 12.0, 6.0 Hz, N-C*H*$_{2a}$-CH$_2$), 3.09 (1H, dd, *J* = 12.0, 8.0 Hz, N-C*H*$_{2b}$-CH$_2$), 2.37 (1H, ddt, *J* = 12.0, 6.5, 3.0 Hz, CH$_2$-C*H*-CH$_2$), 2.29 (3H, s, CO-C*H*$_3$), 2.18 -2.14 (2H, m, CH=CH-C*H*$_2$), 1.80 – 1.60 (2H, m, N-CH$_2$-C*H*$_2$), 1.24 (3H, t, *J* = 7.0 Hz, COCH$_2$-C*H*$_3$). ^{13}C NMR (101 MHz, CDCl$_3$) δ_c 211.5 (*C*OMe), 169.4 (*C*OOCH$_2$CH$_3$), 125.9 (CH=*C*H-CH$_2$), 124.7 (*C*H=CH-CH$_2$), 95.8 (N-*C*H-O), 80.6 (N-*C*-COMe), 72.8 (O-*C*H-CH=CH), 61.2 (COO*C*H$_2$), 57.5 (N-*C*H$_2$), 39.2 (CH$_2$-*C*H-CH$_2$), 30.8 (N-CH$_2$-*C*H$_2$), 24.9 (CO-*C*H$_3$), 24.4 (CH=CH-*C*H$_2$), 14.1 (COCH$_2$-*C*H$_3$). m/z (ESI+) 288.121 (M+Na+), HRMS: (ESI+) calculated for $C_{14}H_{19}NO_4$: 266.1387. Found (M+H+) 266.1387. ^{1}H NOE showed no enhancement signal between N-C*H*-CO, and O-C*H*-CH=CH, consistent with *anti*-stereochemistry of the major diastereomer of the title compound.

Minor Diastereomer

υ_{max}/cm^{-1} (film) 2984 w (C-H), 2899 w (C-H) 1735 s (C=O, ester), 1702 s (C=O, ketone). ^{1}H NMR (400 MHz, CDCl$_3$) δ_H 6.08 (1H, ddt, *J* = 10.5, 3.0, 1.5 Hz, CH-CH=C*H*), 5.89- 5.82 (1H, m, CH-C*H*=CH), 4.72 (1H, s, N-C*H*-O), 4.33 (2H, q, *J* = 7.0 Hz, COOC*H*$_2$), 4.27 (1H, dt, *J* = 3.5, 1.5 Hz, O-C*H*-CH=CH), 3.19 (1H, dt, *J* = 11.5, 3.0 Hz, N-C*H*$_{2a}$-CH$_2$), 2.97 (1H, ddd, *J* = 11.5, 8.0, 3.4 Hz, N-C*H*$_{2b}$-CH$_2$), 2.49 (1H, ddt, *J* = 11.0, 7.5, 4.0 Hz, CH$_2$-C*H*-CH$_2$), 2.37 (3H, s, CO-C*H*$_3$), 2.18 (2H, m, CH=CH-C*H*$_2$), 1.84 – 1.66 (2H, m, N-CH$_2$-C*H*$_2$), 1.34 (3H, t, *J* = 7.0 Hz, COCH$_2$-C*H*$_3$). ^{13}C NMR (101 MHz, CDCl$_3$) δ_c 212.0 (*C*OMe), 166.4 (*C*OOCH$_2$CH$_3$), 127.0 (CH=*C*H-CH$_2$), 124.4 (*C*H=CH-CH$_2$), 91.3 (N-*C*H-O), 81.0 (N-*C*-COMe), 71.7 (O-*C*H-CH=CH), 61.8 (COO*C*H$_2$), 50.8 (N-*C*H$_2$), 38.3 (CH$_2$-*C*H-CH$_2$), 31.6 (N-CH$_2$-*C*H$_2$), 25.8 (CO-*C*H$_3$), 25.1 (CH=CH-*C*H$_2$), 14.2 (COCH$_2$-*C*H$_3$). m/z (ESI+) 288.121 (M+Na+), HRMS: (ESI+) calculated for $C_{14}H_{19}NO_4$: 266.1387. Found (M+H+) 266.1387. ^{1}H NOE enhancement signal between N-C*H*-CO and O-C*H*-CH=CH, confirmed the *syn*- stereochemistry conformation of the minor diastereomer of the title compound.

(±)-Ethyl (3*S*,8a*S*)-3-cyano-3,4,5,5a,6,8a-hexahydro-2*H*-oxazolo[5,4,3-hi]indole-2-carboxylate 15c

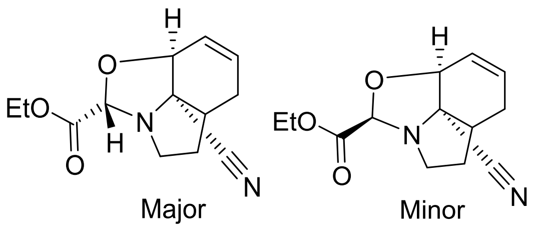

General procedure A for Pd catalysed [3+2] annulation reactions was followed, using ethyl glyoxalate (0.1 mL, 50 % solution in toluene) as the dipolarophile and nitrile aziridine **3c** (40.0 mg, 0.25 mmol) in anhydrous dichloromethane (1.25 mL) to yield the title compound **15c** (31.0 mg, 50%) as an inseparable mixture of diastereomers (d.r. 1:5.5) as a colourless solid. υ_{max}/cm^{-1} (film) 2977 w (C-H),

2900 w (C-H), 2234 s (CN) 1743 s (C=O). ^{1}H NMR (400 MHz, $CDCl_3$) δ_H 6.04 (1H, dt, *J* = 10.0, 2.5 Hz, CH=C*H*-$CH_{2,}$ minor diastereomer) 6.00 – 5.92 (3H, m, C*H*=C*H,* both diastereomers), 5.01 (1H, s, N-C*H*-O, minor diastereomer), 4.98 (1H, s, N-C*H*-O, major diastereomer), 4.89 (1H, dd, *J* = 4.0, 2.0 Hz, O-C*H*-CH=CH, major diastereomer), 4.38-4.25 (3H, m, O-C*H*-CH=CH, CO_2-CH_2-$CH_{3,}$ minor diastereomer), 4.26 (1H, dq, *J* = 7.5, 1.0 Hz, CO_2-CH_2-$CH_{3,}$ major diastereomer), 3.49 (1H, td, *J* = 11.5, 6.5 Hz, N-CH_{2a} - $CH_{2,}$ major diastereomer), 3.21 (1H, td, *J* = 11.0, 7.0 Hz, N-CH_{2a}-CH_2, minor diastereomer), 3.05 (1H, ddd, *J* = 11.5, 7.0, 2.5 Hz, N-CH_{2b}-$CH_{2,}$ major diastereomer), 2.92 (1H, ddd, *J* = 11.0, 8.0, 3.0 Hz, N-CH_{2b}-$CH_{2,}$ minor diastereomer), 2.84 (2H, dtd, *J* = 13.0, 6.5, 2.5 Hz, CH_2-C*H*-CH_2, both diastereomers), 2.52- 2.43 (2H, m, CH=CH-CH_{2a}, both diastereomers), 2.28 (2H, ddd, *J* =18.0, 4.0, 2.5 Hz, CH=CH-C$H_{2b,}$ both diastereomers), 1.85 – 1.76 (2H, m, N-CH_2-CH_2, minor diastereomer), 1.75 – 1.62 (2H, m, N-CH_2-C$H_{2,}$ major diastereomer), 1.34 (6H, t, *J* = 7.0 Hz, COCH_2-C$H_{3,}$ both diastereomers). ^{13}C NMR (101 MHz, $CDCl_3$) δ_c 168.6 (*C*=O, major diastereomer), 165.4 (*C*=O, minor diastereomer), 128.5 (CH-*C*H=CH, minor diastereomer), 127.6 (CH-*C*H=CH, major diastereomer), 122.8 (CH-CH=*C*H, major diastereomer), 122.5 (CH-CH=*C*H, minor diastereomer), 121.9 (*C*N, both diastereomers), 96.5 (N-*C*H-O, major diastereomer), 91.5 (N-*C*H-O, minor diastereomer), 75.9 (O-*C*H-CH=CH, major diastereomer), 74.7 (O-*C*H-CH=CH, minor diastereomer), 66.7 (N-*C*-CN, minor diastereomer), 66.0 (N-*C*-CN, major diastereomer), 62.1 (CO_2-*C*H_2-$CH_{3,}$ minor diastereomer), 61.8 (CO_2-*C*H_2-$CH_{3,}$ major diastereomer), 57.7 (N-*C*H_2-$CH_{2,}$ major diastereomer), 50.8 (N-*C*H_2-$CH_{2,}$ minor diastereomer), 42.1 (CH_2-*C*H-CH_2, major diastereomer), 40.5 (CH_2-*C*H-CH_2 minor diastereomer), 30.8 (N-CH_2-*C*$H_{2,}$ minor diastereomer), 30.5 (N-CH_2-*C*$H_{2,}$ major diastereomer), 24.4 (CH=CH-*C*$H_{2,}$ minor), 24.0 (CH=CH-*C*H_2, major diastereomer), 14.1 (CO_2-CH_2-*C*$H_{3,}$ both diastereomers). m/z (ESI+) 271.155 (M+Na+), HRMS: (ESI+) calculated for $C_{13}H_{17}N_2O_3$: 249.1234. Found (M+H+) 249.1231. ^{1}H NOE enhancement signal between CH_2-C*H*-CH_2 and N-CH_{2b}-CH_2 (3.05 ppm) confirms the *syn*-stereochemistry of these protons in the major diastereomer. ^{1}H NOE enhancement signal between N-C*H*-CO and N-CH_{2a}-CH_2 (3.49 ppm) confirms the *syn*-sterechemistry of these protons in the major diastereomer, and thus the *anti*-stereochemical relationship between N-C*H*-CO and CH_2-C*H*-CH_2 in the major diastereomer of the title compound.

(±)-(3*R*,8a*S*)-2-(4-acetylphenyl)-*N*-ethyl-5,5a,6,8a-tetrahydro-2*H*-oxazolo[5,4,3-hi]indole-31(4*H*)-carboxamide 17a

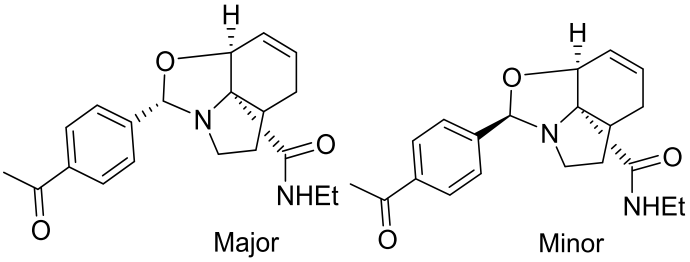

General procedure A for Pd catalysed [3+2] annulation reactions was followed, using 4-acetylbenzaldehyde (74.0 mg, 0.50 mmol) as the dipolarophile and amide aziridine **3a** (48.0 mg, 0.25 mmol) in anhydrous dichloromethane (1.25 mL) to yield the title compound **17a** (24.0 mg, 28%) as an inseparable mixture of diastereomers (d.r. 2.2:1), and as off white oil. υ_{max}/cm^{-1} (film) 3355 w (N-H), 2965 w (C-H), 2924 w (C-H), 1681 s (C=O, ketone), 1659 s (C=O, amide) 1513 m (C=C). ^{1}H NMR (400 MHz, $CDCl_3$) δ_H 7.99 (2H, dt, *J* = 8.5, 2.0 Hz, N-CH-C-C*H,* minor diastereomer), 7.97 (2H, dt, *J* = 8.5, 2.0 Hz, N-CHC-C*H*, major diastereomer), 7.74 (1H, brs, N*H*, minor diastereomer), 7.61 – 7.58 (2H, m, CO-C-C*H* aromatic, minor diastereomer), 7.58 – 7.55 (2H, m, CO-C-C*H* aromatic, major diastereomer), 7.43 (1H, brs, N-*H*, major diastereomer), 6.13 (1H, ddd, *J* = 10.0, 3.5, 2.5, 1.5 Hz, C*H*=CH-$CH_{2,}$ minor diastereomer), 6.06 (1H, ddtd, *J* = 10.5, 6.0, 2.0, 1.0 Hz, CH=C*H*-$CH_{2,}$ major diastereomer), 6.03 – 5.94 (1H, m, CH-CH=C*H,* minor diastereomer), 5.95 (1H, dtd, *J* = 10.5, 2.5, 1.5 Hz, CH-C*H*=CH, major diastereomer), 5.42 (1H, s, N-C*H*-O, minor diastereomer), 5.34 (1H, s, N-C*H*-O, major diastereomer), 4.63 (1H, dd, *J* = 2.5, 2.0 Hz, C*H*-CH=CH, major diastereomer), 4.30 (1H, dt, *J* = 3.5, 2.0 Hz, C*H*-CH=CH, minor diastereomer), 3.35 (2H, qd, *J* = 7.0, 1.5 Hz, CONH-$CH_{2a,}$ minor diastereomer), 3.21 (1H, dqd, *J* = 13.5, 7.5, 6.5 Hz, CONH-$CH_{2a,}$ major diastereomer), 3.13- 3.05 (2H, m, CONH-$CH_{2b,}$ N-CH_{2a}-$CH_{2,}$ major diastereomer), 2.99 (1H, ddd, *J* = 12.0, 7.0, 3.0 Hz, N-CH_{2b}-$CH_{2,}$ major diastereomer), 2.71 – 2.66 (2H, m, N-CH_2-$CH_{2,}$ minor diastereomer), 2.63 (3H, s, CO-$CH_{3,}$ minor diastereomer), 2.61 (3H, s, CO-$CH_{3,}$ major diastereomer), 2.60- 2.46 (4H, m, CH_2-C*H*-$CH_{2,}$ CH=CH-CH_{2a}, both diastereomers), 2.26- 2.20 (1H, m, CH=CH-CH_{2b}, minor diastereomer), 2.23 (1H, dd, *J* = 12.5, 5.5 Hz, CH=CH-CH_{2b}, major diastereomer), 1.91 – 1.78 (2H, m N-CH_2-CH_{2b}, major diastereomer), 1.65 – 1.59 (2H, m N-CH_2-$CH_{2b,}$ minor diastereomer), 1.22 (3H, t, *J* = 7.5 Hz, CONHCH_2-CH_3, minor diastereomer), 0.98 (3H, t, *J* = 7.5 Hz, CONHCH_2-CH_3, major diastereomer). ^{13}C NMR (101 MHz, $CDCl_3$) δ_C 197.7 (*C*OMe, both diastereomers), 175.0 (*C*ONHEt, major diastereomer), 174.2 (*C*ONHEt, minor diastereomer), 145.2 (N-CH-*C* aromatic, major diastereomer), 136.7 (CO-*C* aromatic, minor diastereomer), 141.3 (N-CH-*C* aromatic, minor diastereomer), 137.2 (CO-*C* aromatic, major diastereomer), 129.3 (CH=*C*H-$CH_{2,}$ major diastereomer), 128.5 (N-CH-C-*C*H aromatic, major

diastereomer), 128.3 (N-CH-C-*C*H aromatic, minor diastereomer), 128.1 (CH-CH=*C*H, minor diastereomer), 127.1 (CO-C-*C*H aromatic, minor diastereomer) 126.9 (CO-C-*C*H aromatic, major diastereomer) 125.8 (*C*H=CH-$CH_{2,}$ major diastereomer), 124.1 (*C*H=CH-$CH_{2,}$ minor), 97.0 (N-*C*H-O, major diastereomer), 93.2 (N-*C*H-O, minor diastereomer), 77.2 (N-*C*-CONHEt, minor diastereomer), 76.9 (N-*C*-CONHEt, major diastereomer), 75.3 (O-*C*H-CH=CH, major diastereomer), 73.6 (O-*C*H-CH=CH, minor diastereomer), 52.4 (N-*C*H_2-$CH_{2,}$ major diastereomer), 47.9 (N-*C*H_2-CH_2, minor diastereomer), 40.9 (CH_2-*C*H-$CH_{2,}$ major diastereomer), 40.17 (CH_2-*C*H-CH_2 minor diastereomer), 33.6 (CONH-*C*$H_{2,}$ both diastereomers), 32.5 (N-CH_2-*C*H_2, major diastereomer), 31.9 (N-CH_2-*C*$H_{2,}$ minor diastereomer), 26.7 (CO-*C*H_3, both diastereomers), 26.2 (CH=CH-*C*$H_{2,}$ minor diastereomer), 25.9 (CH=CH-*C*$H_{2,}$ major diastereomer), 15.1 (CONHCH_2-*C*$H_{3,}$ minor diastereomer), 14.7 (CONHCH_2-*C*$H_{3,}$ major diastereomer). m/z (ESI+) 363.1681 (M+Na+), HRMS: (ESI+) calculated for $C_{20}H_{25}N_2O_3$: 341.1860. Found (M+H+) 341.1861. ^{1}H NOE showed no enhancement signal between N-C*H*-C, and O-C*H*-CH=CH, consistent with *anti*-stereochemistry of the major diastereomer of the title compound. ^{1}H NOE enhancement signal between N-C*H*-C and O-C*H*-CH=CH, confirmed the *syn*- stereochemistry conformation of the minor diastereomer of the title compound.

(±)-(3*R*,8a*S*)-*N*-Ethyl-2-(4-nitrophenyl)-5,5a,6,8a-tetrahydro-2H-oxazolo[5,4,3-hi]indole-3(4*H*)-carboxamide 18a

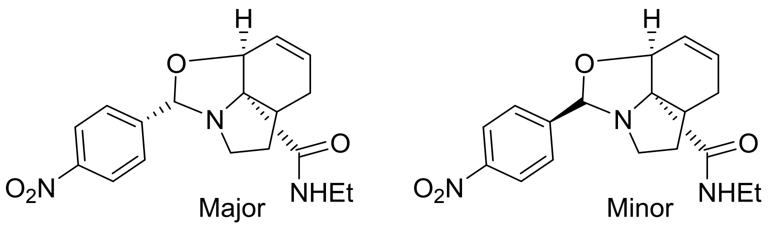

General procedure A for Pd catalysed [3+2] annulation reactions was followed, using 4-nitrobenzaldehyde (152 mg, 1.00 mmol) as the dipolarophile and amide aziridine **3a** (48.0 mg, 0.25 mmol) in anhydrous dichloromethane (1.25 mL) to yield the title compound **18a** (42.0 mg, 71%) as an inseparable mixture of diastereomers (d.r. 3.1:1), and as a yellow oil. υ_{max}/cm^{-1} (film) 3362 w (N-H), 2969 w (C-H), 2891 w (C-H), 1659 s (C=O), 1517 m (C=C). ^{1}H NMR (400 MHz, $CDCl_3$) δ_H 8.24 (2H, d, *J* = 9.0 Hz, ArC*H*, minor diastereomer), 8.21 (2H, d, *J* = 9.0 Hz, ArC*H*, major diastereomer), 7.67 (2H, d, *J* = 9.0 Hz, ArC*H*, minor diastereomer), 7.63 (2H, d, *J* = 9.0 Hz, ArC*H*, major diastereomer), 7.33 (1H, t, *J* = 5.5 Hz, N*H*, minor diastereomer), 7.30 (1H, t, *J* = 5.5 Hz, N*H*, major diastereomer), 6.10 (1H, dt, *J* = 10.0, 2.0 Hz, CH-CH=C*H*, minor diastereomer), 6.08 – 6.00 (1H, m, CH-CH=C*H*, major diastereomer), 5.98 (1H, dt, *J* = 11.0, 2.0 Hz, CH-C*H*=CH, minor diastereomer), 5.92 (1H, dt, *J* = 11.0, 2.0 Hz, CH-C*H*=CH, major diastereomer), 5.41 (1H, s, N-C*H*-O, minor diastereomer), 5.36 (1H, s, N-

C*H*-O, major diastereomer), 4.60 (1H, dd, *J* = 5.0, 2.0 Hz, O-C*H*-CH=CH, major diastereomer), 4.30 (1H, dt, *J* = 4.0, 2.0 Hz, O-C*H*-CH=CH, minor diastereomer), 3.38 – 3.29 (2H, m, CONH-CH_2 , minor diastereomer), 3.25 – 3.04 (3H, m, CONH-CH_2, N-CH_{2a}-CH_2, major diastereomer), 3.14 – 3.03 (2H, m, N-CH_2-CH_2, minor diastereomer), 2.99 (1H, ddd, *J* = 12.5, 6.4, 3.0 Hz, N-CH_{2b}-CH_2, major diastereomer), 2.69 (1H, td, *J* = 11.0, 6.5 Hz, CH_2-C*H*-CH_2, minor diastereomer), 2.62 -2.45 (3H, m, CH=CH-CH_{2a} both diastereomers, CH_2-C*H*-CH_2, major diastereomer), 2.22 (1H, dd, *J* = 14.5, 6.0 Hz, CH=CH-CH_{2b}, major diastereomer), 2.20 – 2.16 (1H, m, CH=CH-CH_{2b}, minor diastereomer), 1.91 – 1.78 (2H, m, N-CH_2-CH_2, major diastereomer), 1.67 – 1.55 (2H, m, N-CH_2-CH_2, minor diastereomer), 1.20 (3H, t, *J* = 7.0 Hz, CONHCH_2-CH_3, minor diastereomer), 0.96 (3H, t, *J* = 7.5 Hz, CONHCH_2-CH_3, major diastereomer). ^{13}C NMR (101 MHz, $CDCl_3$) δ 174.7 (*C*O, both diastereomers), 148.0 (*C*-NO_2, minor diastereomer), 147.3 (*C*-NO_2, major diastereomer), 130.5 (N-CH-*C*Ar, minor diastereomer), 129.4 (CH-CH=*C*H, major diastereomer), 128.2 (CH-CH=*C*H, minor diastereomer), 127.9 (N-CH-*C*Ar, major diastereomer), 127.6 (Ar*C*H, major diastereomer), 125.4 (CH-*C*H=CH, major diastereomer), 124.3 (Ar*C*H, minor diastereomer), 123.9 (CH-*C*H=CH, minor diastereomer), 123.7 (Ar*C*H, major diastereomer), 123.5 (Ar*C*H minor diastereomer), 96.7 (N-*C*H-O, major diastereomer), 92.6 (N-*C*H-O, minor diastereomer), 77.0 (N-*C*-CONHEt, major diastereomer), 76.8 (N-*C*-CONHEt, minor diastereomer), 75.2 (O-*C*H-CH=CH, major diastereomer), 73.7 (O-*C*H-CH=CH, minor diastereomer), 52.8 (N-*C*H_2-CH_2, major diastereomer), 48.1 (N-*C*H_2-CH_2, minor diastereomer), 40.9 (CH_2-*C*H-CH_2, major diastereomer), 40.1 (CH_2-*C*H-CH_2, minor diastereomer), 33.7 (CONH-*C*H_2, both diastereomers), 32.5 (N-CH_2-*C*H_2, major diastereomer), 31.9 (N-CH_2-*C*H_2, minor diastereomer), 26.2 (CH=CH-*C*H_2, minor diastereomer), 25.8 (CH=CH-*C*H_2, major diastereomer), 15.1 (CONHCH_2-*C*H_3, minor diastereomer), 14.8 (CONHCH_2-*C*H_3, major diastereomer). m/z (ESI+) 344.161 (M+H+), HRMS: (ESI+) calculated for $C_{18}H_{22}N_3O_4$: 344.1605. Found (M+H+) 344.1608. ^{1}H NOE showed no enhancement signal between N-C*H*-C, and O-C*H*-CH=CH, consistent with *anti*-stereochemistry of the major diastereomer of the title compound. ^{1}H NOE enhancement signal between N-C*H*-C and O-C*H*-CH=CH, confirmed the *syn*- stereochemistry conformation of the minor diastereomer of the title compound.

(±)-(3*R*,8a*S*)-*N*-Ethyl-2-methyl-1-tosyl-1,4,5,5a,6,8a-hexahydroimidazo[4,5,1-hi]indole-3(2*H*)-carboxamide 19a

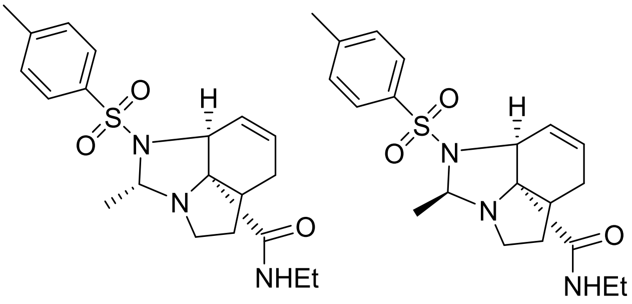

General procedure A for Pd catalysed [3+2] annulation reactions was followed, using *N*-ethylidene-4-methylbenzenesulfonamide (98.0 mg, 0.50 mmol) as the dipolarophile and amide aziridine **3a** (48.0 mg, 0.25 mmol) in anhydrous dichloromethane (1.25 mL). Purification by column chromatography (40% hex/EtOAc) yielded the title compound **19a** (62.0 mg, 64%) as a partially separable mixture of diastereomers (d.r. 2:1), and as a pale yellow solids.

Major Diastereomer

M.p 79 – 80 °C (EtOAc). υ_{max}/cm^{-1} (film) 3346 w (N-H), 2970 w (C-H), 2932 w (C-H), 1657 s (C=O). ^{1}H NMR (400 MHz, $CDCl_3$) δ_H 7.70 (2H, d, *J* = 8.0 Hz, ArC*H*), 7.42 (1H, t, *J* = 5.5 Hz, CON*H*), 7.25 (2H, d, *J* = 8.0 Hz, ArC*H*), 6.07 (1H, ddt, *J* = 10.0, 3.5, 1.5 Hz, N-CH-C*H*=CH), 5.87 (1H, dddd, *J* = 10.0, 5.5, 3.5, 1.5 Hz, CH=C*H*-CH_2), 4.60 (1H, q, *J* = 6.0 Hz, N-C*H*-NTs), 4.36 – 4.34 (1H, m, N-C*H*-CH=CH), 3.29 – 3.10 (3H, m, CONH-CH_2, N-CH_{2a}-CH_2), 2.86 (1H, ddd, *J* = 11.0, 7.5, 5.5 Hz, N-CH_{2b}-CH_2), 2.45 – 2.40 (4H, CH_2-C*H*-CH_2, ArC-CH_3), 2.32 (1H, dddd, *J* = 17.0, 6.0, 3.5, 1.5, CH=CH-CH_{2a}), 1.94 (1H, dtd, *J* = 17.0, 5.0, 1.5 Hz, CH=CH-CH_{2b}), 1.80 – 1.63 (2H, m, N-CH_2-CH_2), 1.29 (3H, d, *J* = 6.0 Hz, N-CH-CH_3), 1.07 (3H, t, *J* = 7.0 Hz, CONHCH_2-CH_3). ^{13}C NMR (101 MHz, $CDCl_3$) δ_c 175.1 (*C*=O), 143.2 (CH_3-*C*Ar), 138.9 (SO_2-*C*Ar), 129.4 (Ar*C*H), 128.9 (CH=*C*H-CH_2), 127.2 (Ar*C*H), 124.7 (N-CH-*C*H=CH), 79.7 (N-*C*H-NTs), 77.2 (N-*C*-CONHEt), 55.7 (N-*C*H-CH=CH), 54.3 (N-*C*H_2-CH_2), 42.3 (CH_2-*C*H-CH_2), 33.8 (CONH-*C*H_2), 31.8 (N-CH_2-*C*H_2), 25.8 (CH=CH-*C*H_2), 21.5 (Ar-*C*H_3), 20.1 (N-CH-*C*H_3), 14.8 (CONHCH_2-*C*H_3). m/z (ESI+) 412.168 (M+Na+), HRMS: (ESI+) calculated for $C_{20}H_{28}N_3O_3S$: 390.1846 Found (M+H+) 390.1857. ^{1}H NOE showed no enhancement signal between N-C*H*-CH_3, and N-C*H*-CH=CH, consistent with *anti*-stereochemistry of the major diastereomer of the title compound.

Minor Diastereomer

M.p 138 – 139 °C (EtOAc/Pet). υ_{max}/cm^{-1} (film) 3346 w (N-H), 2970 w (C-H), 2932 w (C-H), 1657 s (C=O). ^{1}H NMR (400 MHz, $CDCl_3$) δ_H 7.67 (2H, d, *J* = 8.0 Hz, ArC*H*), 7.31 (2H, d, *J* = 8.0 Hz, ArC*H*), 6.84

(1H, t, *J* = 5.0 Hz, CON*H*), 6.16 (1H, dt, *J* = 10.0, 2.5 Hz, N-CH-C*H*=CH), 5.88 (1H, ddd, *J* = 10.0, 7.0, 3.0 Hz, CH=C*H*-CH_2), 4.41 (1H, dd, *J* = 5.0, 3.0 Hz, N-C*H*-CH=CH), 3.95 (1H, q, *J* = 6.0 Hz, N-C*H*-CH_3), 2.94 – 2.77 (4H, m, CONH-CH_2, N-CH_2-CH_2), 2.44 – 2.39 (4H, m, CH_2-C*H*-CH_2, ArC-CH_3), 2.22 (1H, ddd, *J* = 16.0, 5.5, 3.0 Hz, CH=CH-CH_{2a}), 2.14 – 2.00 (2H, m, CH=CH-CH_{2b}, N-CH_2-CH_{2a}), 1.61 (3H, d, *J* = 6.0 Hz, N-CH-CH_3), 1.48 – 1.42 (1H, m, N-CH_2-CH_{2b}), 0.82 (3H, t, *J* = 7.0 Hz, CONHCH$_2$-CH_3). ^{13}C NMR (101 MHz, $CDCl_3$) δ_c 172.4 (*C*=O), 143.6 (CH_3-*C*Ar), 133.0 (SO_2-*C*Ar), 132.6 (N-CH-*C*H=CH), 129.5 (Ar*C*H), 128.1 (Ar*C*H), 125.7 (CH=*C*H-CH_2), 77.9 (N-*C*-CONHEt), 74.9 (N-*C*H-NTs), 57.2 (N-*C*H-CH=CH), 48.7 (N-*C*H$_2$-CH_2), 40.4 (CH_2-*C*H-CH_2), 33.6 (CONH*C*H$_2$), 31.9 (N-CH_2-*C*H$_2$), 27.2 (CH=CH-*C*H$_2$), 21.5 (Ar-*C*H$_3$), 18.5 (N-CH-*C*H$_3$), 14.5 (CONHCH$_2$-*C*H$_3$). m/z (ESI+) 412.168 (M+Na+), HRMS: (ESI+) calculated for $C_{20}H_{28}N_3O_3S$: 390.1846 Found (M+H+) 390.1857. ^{1}H NOE enhancement signal between N-C*H*-CH_3 and N-C*H*-CH=CH, confirmed the *syn-* stereochemistry conformation of the minor diastereomer of the title compound.

(±)-(3*R*,8a*S*)-*N*-Ethyl-2-(4-nitrophenyl)-1-tosyl-1,4,5,5a,6,8a-hexahydroimidazo[4,5,1-hi]indole-3(2*H*)-carboxamide 20a

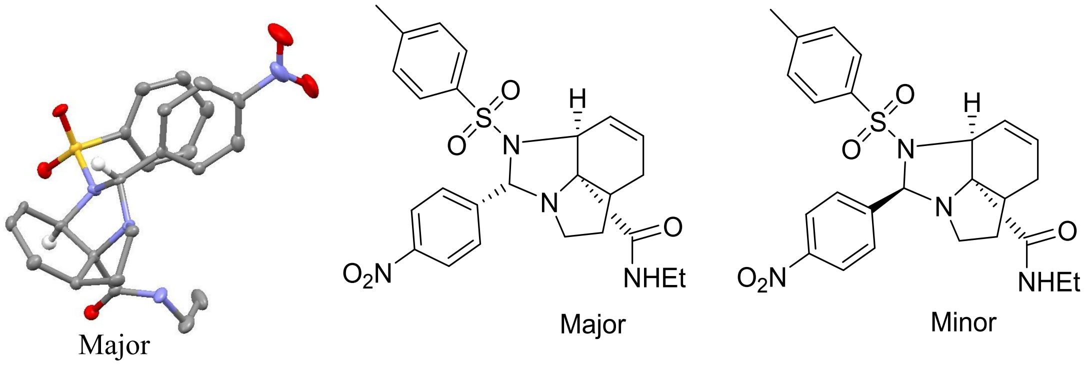

General procedure A for Pd catalysed [3+2] annulation reactions was followed, using (4-methyl-N-(4-nitrobenzylidene)benzenesulfonamide (152.0 mg, 0.50 mmol) as the dipolarophile and amide aziridine **3a** (48.0 mg, 0.25 mmol) in anhydrous dichloromethane (1.25 mL). Purification by column chromatography (10% hex/EtOAc) yielded the title compound **20a** (47.0 mg, 47%) as an inseparable mixture of diastereomers (d.r. 4.2:1), and as a colourless solid. Mp. 190 – 191 °C (EtOAc). υ_{max}/cm^{-1} (film) 3359 w (N-H), 2972 w (C-H), 2901 w (C-H), 1656 s (C=O). ^{1}H NMR (400 MHz, $CDCl_3$) δ_H 8.21 (2H, d, *J* = 9.0 Hz, NO_2-C-C*H*, minor diastereomer), 7.93 (2H, d, *J* = 9.0 Hz, NO_2-C-C*H*, major diastereomer), 7.59 (1H, d, *J* = 9.0 Hz, NO_2-CCH-C*H*, minor diastereomer), 7.35 – 7.28 (6H, m, NO_2-CCH-C*H*, major diastereomer, SO_2-C-*C*H, both diastereomers), 7.10 (1H, t, *J* = 5.0 Hz, CON*H*, major diastereomer), 7.03 (2H, d, *J* = 8.0 Hz, SO_2-CCH-*C*H, both diastereomers), 6.91 (1H, t, *J* = 5.0 Hz, CON*H*, minor

diastereomer), 6.30 (1H, dd, *J* = 10.0, 3.5 Hz, N-CH-C*H*=CH, major diastereomer), 6.24 (1H, dd, *J* = 10.0, 2.5 Hz, N-CH-C*H*=CH, minor diastereomer), 6.02 (1H, dddd, *J* = 10.0, 8.0, 4.0, 2.0 Hz, CH=C*H*-CH_2, major diastereomer), 5.94 (1H, tdd, *J* = 10.5, 5.5, 3.0, Hz, CH=C*H*-CH_2, minor diastereomer), 5.28 (1H, s, N-C*H*-NTs, major diastereomer), 5.19 (1H, s, N-C*H*-NTs, minor diastereomer), 4.81 (1H, dd, *J* = 4.0, 2.5 Hz, N-C*H*-CH=CH, major diastereomer), 4.65 (1H, dd, *J* = 5.5, 2.5 Hz, N-C*H*-CH=CH, minor diastereomer), 3.17 (1H, dq, *J* = 14.0, 7.5 Hz, CONHCH_{2a}, major diastereomer), 3.09 – 2.98 (3H, CONHCH_{2b}, major diastereomer, CONHCH_{2a}, major diastereomer, N-CH_{2a}-CH_2, major diastereomer), 2.90 (1H, dq, *J* = 13.6, 7.0 Hz, CONHCH_{2b}, minor diastereomer), 2.85 – 2.75 (2H, m, N-CH_{2b}-CH_2, both diastereomers), 2.54 – 2.44 (2H, m, CH_2-C*H*-CH_2, both diastereomers), 2.42 – 2.34 (4H, m, Ar-CH_3, minor diastereomer, CH=CH-CH_{2a}, major diastereomer), 2.34 (3H, s, Ar-CH_3, major diastereomer), 2.12 (1H, td *J* = 8.5, 2.5 Hz, CH=CH-CH_{2a}, minor diastereomer), 2.03 (1H, ddd, *J* = 16.0, 7.0, 2.5 Hz, CH=CH-CH_{2b}, minor diastereomer), 1.95 (1H, dt, *J* = 16.5, 4.0 Hz, CH=CH-C$H_{2b,}$ major diastereomer), 1.84 -1.74 (2H, m, N-CH_2-CH_{2a}, both diastereomers), 1.70 – 1.61 (2H, m, N-CH_2-CH_{2b}, both diastereomers), 0.91 (3H, t, *J* = 7.0 Hz, CONHCH_2-CH_3 major diastereomer), 0.90 (3H, t, *J* = 7.0 Hz, CONHCH_2-CH_3 minor diastereomer). ^{13}C NMR (101 MHz, $CDCl_3$) δ_c 174.4 (*C*=O, major diastereomer), 171.9 (*C*=O, minor diastereomer), 148.1 (*C*Ar, minor diastereomer), 147.8 (*C*Ar, major diastereomer), 145.4 (*C*Ar, major diastereomer), 144.4 (*C*Ar, minor diastereomer), 143.8 (CAr, minor diastereomer), 143.7 (*C*Ar, major diastereomer), 138.5 (*C*Ar, major diastereomer), 132.3 (*C*Ar, minor diastereomer), 131.8 (CH-*C*H=CH, minor diastereomer), 130.2 (CH=*C*H-CH_2, major diastereomer), 129.8 (CH=*C*H-CH_2, minor diastereomer), 129.4 (*C*HAr, minor diastereomer), 129.3 (*C*HAr, major diastereomer), 129.1 (*C*HAr, major diastereomer), 128.5 (*C*HAr, minor diastereomer), 127.3 (*C*HAr, major diastereomer), 126.9 (*C*HAr, minor diastereomer), 125.1 (CH-*C*H=CH, major diastereomer), 123.4 (*C*HAr, minor diastereomer), 123.1 (*C*HAr, major diastereomer), 83.0 (N-*C*H-NTs, major diastereomer), 80.4 (N-*C*H-NTs, minor diastereomer), 79.0 (N-*C*-CONHEt, minor diastereomer), 7.24 (N-*C*-CONEt, major diastereomer), 59.3 (N-*C*H-CH=CH, major diastereomer), 57.5 (N-*C*H-CH=CH, minor diastereomer), 53.0, (N-*C*H_2-$CH_{2,}$ major diastereomer), 51.0 (N-*C*H_2-$CH_{2,}$ minor diastereomer), 42.1 (CH_2-*C*H-CH_2, major diastereomer), 40.6 (CH_2-*C*H-CH_2, minor diastereomer), 33.9 (CCONH-*C*H_2, both diastereomers), 32.3 (N-CH_2-*C*H_2, major diastereomer), 30.4 (N-CH_2-*C*H_2, minor diastereomer), 27.4 (CH=CH-*C*$H_{2,}$ minor diastereomer), 25.8 (CH=CH-*C*$H_{2,}$ major diastereomer), 21.6 (Ar-*C*H_3, minor diastereomer), 21.5 (Ar-*C*H_3, major diastereomer), 14.9 (CONHCH_2-*C*H_3, minor diastereomer), 14.6 (CONHCH_2-*C*H_3, major diastereomer). m/z (ESI+) 519.166 (M+Na+), HRMS: (ESI+) calculated for $C_{25}H_{29}N_4O_5S$: 497.1853 Found (M+H+) 497.1842. The major diastereomer was isolated by crystallisation (EtOAc/Pet) and the stereochemistry of the major diastereomer was confirmed by single crystal X-ray diffraction (see above).

(±)-(3*R*,8a*S*)-*N*,2-Diethyl-1-tosyl-1,4,5,5a,6,8a-hexahydroimidazo[4,5,1-hi]indole-3(2*H*)-carboxamide 21a

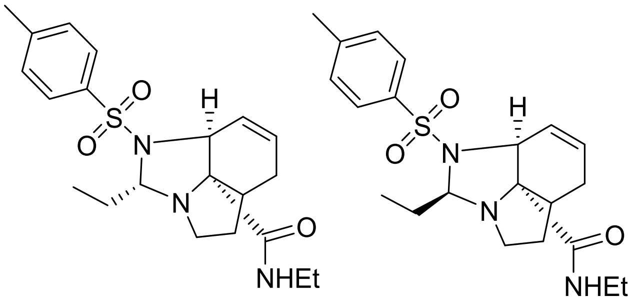

Major Minor

General procedure A for Pd catalysed [3+2] annulation reactions was followed, using 4-methyl-*N*-propylidenebenzenesulfonamide (106.0 mg, 0.50 mmol) as the dipolarophile and amide aziridine **3a** (48.0 mg, 0.25 mmol) in anhydrous dichloromethane (1.25 mL). Purification by column chromatography (30% hex/EtOAc) yielded the title compound **21a** (47.0 mg, 47%) as a partially separable mixture of diastereomers (d.r. 2.5:1), and as a colourless solid

Major Diastereomer

M.p 131 – 133 °C (EtOAc/Pet). υ_{max}/cm^{-1} (film) 3364 w (N-H), 2968 w (C-H), 2875 w (C-H), 1663 s (C=O). ^{1}H NMR (400 MHz, $CDCl_3$) δ_H 7.70 (2H, d, *J* = 8.0 Hz, ArC*H*), 7.34 (1H, t, *J* = 5.0 Hz, N-*H*), 7.25 (2H, d, *J* = 8.0 Hz, ArC*H*), 6.08 (1H, ddt, *J* = 10.0, 4.0, 1.5 Hz, N-CH-C*H*=CH), 5.81 (1H, dt, *J* = 10.0, 5.0 Hz, CH=C*H*-CH_2), 4.47 (1H, dd, *J* = 11.0, 3.0 Hz, N-C*H*-NTs), 4.27 (1H, d, *J* = 4.0 Hz, N-C*H*-CH=CH), 3.39 – 3.28 (2H, m, CONH-CH_{2a}, N-CH_{2a}-CH_2), 3.13 (1H, dqd, *J* = 14.5, 7.5, 5.0 Hz, CONH-CH_{2b}), 2.92 (1H, dt, *J* = 10.0, 7.0 Hz, N-CH_{2b}-CH_2), 2.42 – 2.34 (4H, m, CH_2-C*H*-CH_2, Ar-CH_3), 2.23 (1H, dtd, *J* = 16.0, 4.0, 2.0 Hz, CH=CH-CH_{2a}), 1.96 – 1.74 (3H, m, N-CH_2-CH_{2a}, CH=CH-C$H_{2b,}$ N-CH-CH_{2a}-CH_3), 1.68 – 1.57 (1H, m, N-CH_2-CH_{2b}), 1.40 (1H, ddq, *J* = 14.5, 11.0, 7.0 Hz, N-CH-CH_{2b}-CH_3), 1.10 (3H, t, *J* = 7.0 Hz, CONHCH_2-CH_3), 0.96 (3H, t, *J* = 7.5 Hz, N-CH-CH_2-CH_3). ^{13}C NMR (101 MHz, $CDCl_3$) δ_c 175.1 (*C*=O), 143.1 (CH_3-*C*Ar), 139.0 (SO_2-*C*Ar), 129.3 (Ar*C*H), 129.1 (CH-*C*H=CH), 127.2 (Ar*C*H), 124.1 (CH=*C*H-CH_2), 86.4 (N-*C*H-NTs), 78.2 (N-*C*-CONHEt), 56.6 (N-*C*H_2-CH_2), 55.1 (N-*C*H-CH=CH), 43.1 (CH_2-*C*H-CH_2), 33.8 (CONH-*C*H_2), 31.6 (N-CH_2-*C*H_2), 26.0 (N-CH-*C*H_2-CH_3), 25.9 (CH=CH-*C*H_2), 21.5 (Ar-*C*H_3), 14.7 (CONH-CH_2-*C*H_3), 10.5 (N-CH-CH_2-*C*H_3). m/z (ESI+) 426.183 (M+Na+), HRMS: (ESI+) calculated for $C_{21}H_{30}N_3O_3S$: 404.2002 Found (M+H+) 404.2015. ^{1}H NOE showed no enhancement signal between N-C*H*-$CH_{2,}$ and N-C*H*-CH=CH, consistent with *anti*-stereochemistry of the major diastereomer of the title compound.

Minor Diastereomer

M.p 144 – 146 °C (EtOAc). υ_{max}/cm^{-1} (film) 3364 w (N-H), 2968 w (C-H), 2875 w (C-H), 1663 s (C=O). ^{1}H NMR (400 MHz, $CDCl_3$) δ_H 7.66 (2H, d, *J* = 8.0 Hz, ArC*H*), 7.32 (2H, d, *J* = 8.0 Hz, ArC*H*), 6.80 (1H, t, *J* = 5.0 Hz, N-*H*), 6.16 (1H, dt, *J* = 10.0, 2.5 Hz, N-CH-C*H*=CH), 5.86 (1H, ddt, *J* = 10.0, 7.0, 3.0 Hz, CH=C*H*-CH_2), 4.46 (1H, dd, *J* = 5.0, 3.0 Hz, N-C*H*-CH=CH), 3.65 (1H, dd, *J* = 10.5, 3.5 Hz, N-C*H*-NTs), 2.97 – 2.74 (4H, m, CONH-CH_2, N-CH_2-CH_2), 2.54 – 2.36 (6H, m, N-CH-CH_{2a}-CH_3, CH_2-C*H*-CH_2, Ar-CH_3), 2.23 (1H, ddd, *J* = 16.0, 5.0, 2.5 Hz, CH=CH-CH_{2a}), 2.13 – 2.01 (2H, m, N-CH_2-CH_{2a}, CH=CH-CH_{2b}), 1.72 (1H, dqd, *J* = 14.5, 7.0, 3.5 Hz, N-CH-CH_{2b}-CH_3), 1.45 (1H, dddd, *J* = 13.5, 8.5, 4.0, 1.5, N-CH_2-CH_{2b}), 1.01 (3H, t, *J* = 7.5 Hz, N-CH-CH_2-CH_3), 0.82 (3H, t, *J* = 7.0 Hz, CONHCH_2-CH_3). ^{13}C NMR (101 MHz, $CDCl_3$) δ_c 172.4 (*C*=O), 143.6 (CH_3-*C*Ar), 132.8 (SO_2-*C*Ar), 132.7 (N-CH-*C*H=CH), 129.5 (Ar*C*H), 128.2 (Ar*C*H), 125.6 (CH=*C*H-CH_2), 81.0 (N-*C*H-NTs), 77.8 (N-*C*-CONHEt), 57.5 (N-*C*H-CH=CH), 47.7 (N-*C*H_2-CH_2), 40.1 (CH_2-*C*H-CH_2), 33.6 (CONH-*C*H_2), 31.7 (N-CH_2-*C*H_2), 27.1 (CH=CH-*C*H_2), 24.5 (N-CH-*C*H_2-CH_3), 21.5 (Ar-*C*H_3), 14.4 (CONH-CH_2-*C*H_3), 10.5 (N-CHCH_2-*C*H_3). m/z (ESI+) 426.183 (M+Na+), HRMS: (ESI+) calculated for $C_{21}H_{30}N_3O_3S$: 404.2002 Found (M+H+) 404.2015. ^{1}H NOE enhancement signal between N-C*H*-CH_2 and N-C*H*-CH=CH, confirmed the *syn*- stereochemistry conformation of the minor diastereomer of the title compound.

(±)-(3*R*,8a*S*)-*N*-Ethyl-2-phenyl-1-tosyl-1,4,5,5a,6,8a-hexahydroimidazo[4,5,1-hi]indole-3(2*H*)-carboxamide 22a

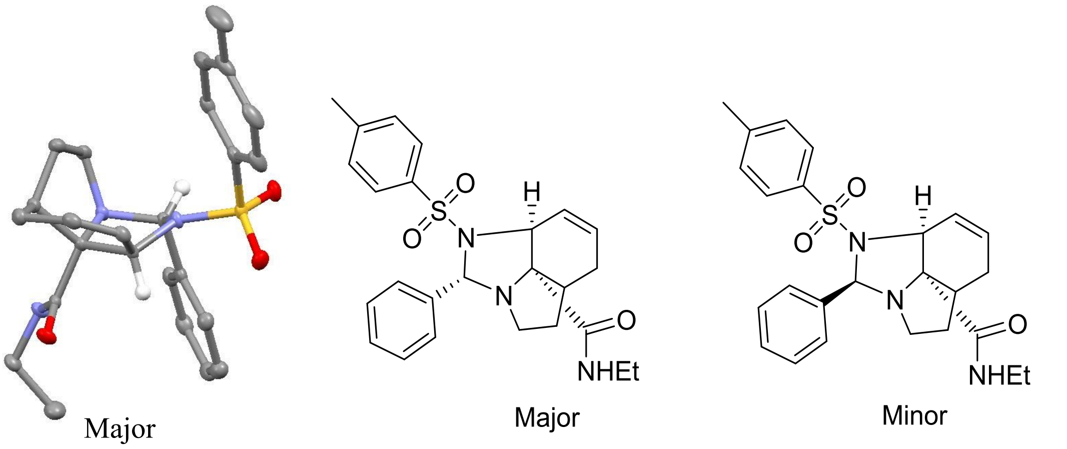

Major Major Minor

General procedure A for Pd catalysed [3+2] annulation reactions was followed, using *N*-benzylidene-4-methylbenzenesulfonamide (129.0 mg, 0.50 mmol) as the dipolarophile and amide aziridine **3a** (48.0 mg, 0.25 mmol) in anhydrous dichloromethane (1.25 mL). Purification by column chromatography (30% hex/EtOAc to 10% hex/EtOAc) yielded the title compound **22a** (43.0 mg, 46%) as an inseparable mixture of diastereomers (d.r. 7:1), and as a colourless solid. υ_{max}/cm^{-1} (film) 3361 w (N-H), 2975 w (C-H), 2929 w (C-H), 1665 s (C=O). ^{1}H NMR (400 MHz, $CDCl_3$) δ_H 7.62 (1H, d, *J* = 7.0

Hz, ArC*H*, minor diastereomer), 7.39 – 7.05 (14H, m, ArC*H*, both diastereomers), 7.04 – 6.59 (3H, m, ArC*H*, both diasteromers), 6.34 (1H, d, *J* = 10.0 Hz, N-CH-C*H*=CH, major diastereomer), 6.31 – 6.23 (1H, m, N-CH-C*H*=CH, minor diastereomer), 6.08 – 5.98 (1H, m, CH=C*H*-CH_2, major diastereomer), 5.97 – 5.87 (1H, m, CH=C*H*-CH_2, major diastereomer), 5.20 (1H, s, N-C*H*-NTs, major diastereomer), 5.12 (1H, s, N-C*H*-NTs, minor diastereomer), 4.78 (1H, s, N-C*H*-CH=CH, major diastereomer), 4.65 (1H, s, N-C*H*-CH=CH, minor diastereomer), 3.46 (1H, dq, *J* = 14.0, 7.0 Hz, CONH-CH_{2a}, minor diastereomer), 3.27 – 3.10 (2H, m, CONH-CH_{2b}, minor diastereomer, CONH-CH_{2a}, major diastereomer), 3.10 – 2.94 (2H, m, CONH-CH_{2b}, major diastereomer, N-CH_{2a}-CH_2, major diastereomer), 2.91 – 2.74 (4H, m, N-CH_{2b}-CH_2, major diastereomer, N-CH_2-CH_2, minor diastereomer), 2.62 – 2.27 (10H, m, CH_2-C*H*-CH_2, Ar-CH_3, CH=CH-CH_{2a} both diastereomers), 1.96 (1H, dt, *J* = 16.0, 5.0 Hz, CH=CH-CH_{2b}, major diastereomer), 1.84 – 1.58 (5H, m, N-CH_2-CH_2 both diastereomers, CH=CH-CH_{2b} minor diastereomer), 0.93 (3H, t, *J* = 7.0 Hz, CONHCH_2-CH_3, major diastereomer), 0.87 (3H, t, *J* = 7.0 Hz, CONHCH_2-CH_3, minor diastereomer). ^{13}C NMR (101 MHz, $CDCl_3$) δ_c 174.9 (*C*=O), 142.7 (Ar*C*), 138.6 (Ar*C*), 137.6 (Ar*C*), 129.8 (CH=*C*H-CH_2), 128.8 (Ar*C*H), 128.4 (Ar*C*H), 128.3 (Ar*C*H), 127.9 (Ar*C*H), 127.2 (Ar*C*H), 125.6 (N-CH-*C*H=CH), 83.9 (N-*C*H-NTs), 60.4 (N-*C*H-CH=CH, minor diastereomer), 59.2 (N-*C*H-CH=CH, major diastereomer), 52.3 (N-CH_2-CH_2), 42.0 (CH_2-*C*H-CH_2), 33.8 (CONH-CH_2), 32.3 (N-CH_2-CH_2), 25.8 (CH=CH-CH_2), 21.4 (Ar-CH_3), 21.0 (Ar-CH_3, minor diasteromer), 14.7 (CONHCH_2-CH_3 major diastereomer), 14.2 ((CONHCH_2-CH_3 minor diastereomer).m/z (ESI+) 452.199 (M+H+), HRMS: (ESI+) calculated for $C_{25}H_{29}N_3O_3S$: 452.2002 Found (M+H+) 452.1995. The major diastereomers were isolated by crystallisation (Ethanol) and the stereochemistry of the major diastereomer was confirmed by single crystal X-ray diffraction (see above).

(±)-(3*R*,5a*R*)-5-acetyl-1,5a,8,8a-tetrahydropyrrolo[3,2,1-hi]indole-31(2*H*)-carbonitrile (Table 4, entry 1)

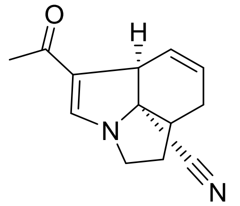

General procedure A for Pd catalysed [3+2] annulation reactions was followed, using 3-butyn-2-one (0.04 mL, 0.50 mmol) as the dipolarophile and nitrile aziridine **3c** (40.0 mg, 0.25 mmol) in anhydrous dichloromethane (1.25 mL) to yield the title compound (28.0 mg, 53%) as a bright yellow solid.

(±)-(3*R*,5a*R*)-5-acetyl-1,5a,8,8a-tetrahydropyrrolo[3,2,1-hi]indole-31(2*H*)-carbonitrile was also obtained by following general procedure B: stirring nitirle aziridine **3c** (40.0 mg, 0.25 mmol) and 3-butyn-2-one (0.03 mL, 0.30 mmol) in MeCN (1.00 mL) at room temperature for 16 h. After concentrating to dryness, purification by column chromatography (5% EtOAc/CH_2Cl_2 then a gradient of 5% EtOAc/CH_2Cl_2 to 20% EtOAc/CH_2Cl_2) gave the title lactam (30.0 mg, 56%) as a bright yellow solid. Mp. 138 – 140 °C (CH_2Cl_2) υ_{max}/cm^{-1} (film) 2925 w (C-H), 2194 s (CN) 1666 (C=O) 1585 m (C=C). ^{1}H NMR (400 MHz, $CDCl_3$) δ_H 8.20 (1H, s, N-C*H*=C), 5.98 (1H, ddd, *J* = 9.0, 6.0, 3.0 Hz, CH-C*H*=CH), 5.69- 5.64 (2H, m, CH-CH=C*H*, C*H*-CH=CH), 4.48 (1H, dd, *J* = 12.0, 9.0 Hz, N-CH_{2a}-CH_2), 3.84 (1H, dt, *J* = 12.0, 7.0 Hz, N-CH_{2b}-CH_2), 2.90 (1H, dtd, *J* = 18.5, 9.0, 2.5 Hz, CH_2-C*H*-CH_2), 2.50 (1H, ddd, *J* = 16.5, 9.0, 6.5 Hz, CH=CH-CH_{2a}), 2.42- 2.36 (4H, m, N-CH_2-C$H_{2a,}$ CO-CH_3), 2.02 (1H, tt, *J* = 16.5, 3.0 Hz, CH=CH-CH_{2b}), 1.71 (1H, qd, *J*= 12.0, 9.0 Hz, N-CH_2-CH_{2b}). ^{13}C NMR (101 MHz, $CDCl_3$) δ_c 192.3 (*C*=O), 145.6 (N-*C*H=C), 143.3 (N-*C*-CN), 124.1 (CH-*C*H=CH), 122.1 (CH-CH=*C*H), 119.7 (N-CH=*C*), 99.7 (N-C-*C*H-CH=CH), 85.4 (*C*N), 50.3 (N-*C*H_2-CH_2), 36.9 (CH_2-*C*-CH_2), 30.3 (N-CH_2-*C*H_2), 29.4 (CH=CH-*C*H_2), 27.9 (CO-*C*H_3). m/z (ESI+) 237.100 (M+Na+), HRMS: (ESI+) calculated for $C_{13}H_{15}N_2O$: 215.1179. Found (M+H+) 215.1183.

(±)-(31*R*,5a*R*)-5-formyl-1,5a,8,8a-tetrahydropyrrolo[3,2,1-hi]indole-31(2*H*)-carbonitrile (Table 4, entry 2)

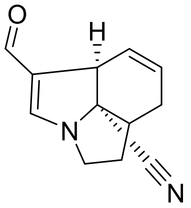

General procedure A for Pd catalysed [3+2] annulation reactions was followed, using TMS-propynal (0.07 mL, 0.50 mmol) as the dipolarophile and nitrile aziridine **3c** (40.0 mg, 0.25 mmol) in anhydrous dichloromethane (1.25 mL) to yield the title compound (30.0 mg, 60%) as a yellow powder.

(±)-(31*R*,5a*R*)-5-formyl-1,5a,8,8a-tetrahydropyrrolo[3,2,1-hi]indole-31(2*H*)-carbonitrile was also obtained following general procedure B: nitrile aziridine **3c** (40.0 mg, 0.25 mmol) and TMS-propynal (0.05 mL, 0.30 mmol) were stirred in MeCN (1.00 mL) at room temperature for 16 h. After concentrating to dryness the product was obtained by column chromatography (100% $CH_2Cl_{2,}$ then 5% EtOAc/CH_2Cl_2). The title compound (31.0 mg, 62%) was obtained as an yellow solid. M.p, 195-196 °C (EtOAc). υ_{max}/cm^{-1} (film) 2977 w (C-H), 2936 w (C-H) 2207 s (CN) 1600 s (C=O) 1588 s (C=C), 1574 s (C=C). ^{1}H NMR (400 MHz, $CDCl_3$) δ_H 9.29 (1H, s, CO*H*), 7.84 (1H, s, N-C*H*=C), 6.00 (1H, ddd, *J* = 9.0, 5.5, 3.5 Hz, CH-C*H*=CH), 5.71 (1H, ddd, *J* = 9.0, 6.5, 2.0 Hz, CH-CH=C*H*), 5.66 (1H, dd, *J* = 5.5, 2.0

Hz, C*H*-CH=CH), 4.52 (1H, dd, *J* = 12.0, 9.5, N-CH_{2a}-CH_2), 3.88 (1H, td, *J* = 12.0, 7.0 Hz, N-*C*H_{2b}-CH_2), 3.01 – 2.87 (1H, m, CH_2-C*H*-CH_2), 2.53 (1H, ddd, *J* = 17.0, 8.0, 6.5 Hz, CH=CH-CH_{2a}), 2.45 (1H, dt, *J* = 12.0, 7.0 Hz, N-CH_2-CH_{2a}), 2.05 (1H, tt, *J* = 17.0, 2.5 Hz, CH=CH-CH_{2b}), 1.76 (1H, qd, *J* = 12.0, 9.5 Hz, N-CH_2-CH_{2b}). ^{13}C NMR (101 MHz, DMSO-d6) δ_c 188.0 (*C*=O), 151.9 (N-CH=*C*-CO), 144.0 (N-*C*H=C), 124.5 (CH-*C*H=CH), 122.5 (CH-CH=*C*H), 116.3 (*C*N), 99.9 (*C*H-CH=CH), 89.7 (N-*C*-CN), 50.5 (N-*C*H_2-CH_2), 36.6 (CH_2-*C*H-CH_2), 30.2 (N-CH_2-*C*H_2), 29.3 (CH=CH-*C*H_2). m/z (ESI+) 223.085 (M+Na+), HRMS: (ESI+) calculated for $C_{12}H_{13}N_2O$: 201.1022. Found (M+H+) 201.1031

(±)-Dimethyl (3*R*,5a*R*)-3-cyano-1,2,3,5a,8,8a-hexahydropyrrolo[3,2,1-hi]indole-4,5-dicarboxylate (Table 4, entry 3)

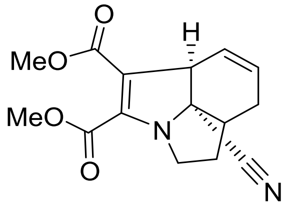

General procedure A for Pd catalysed [3+2] annulation reactions was followed, using dimethyl acetylenedicarboxlyate (0.06 mL, 0.50 mmol) as the dipolarophile and nitrile aziridine **3c** (40.0 mg, 0.25 mmol) in anhydrous dichloromethane (1.25 mL) to yield the title compound (67.0 mg, 92%) as an orange powder.

(±)-Dimethyl (3*R*,5a*R*)-3-cyano-1,2,3,5a,8,8a-hexahydropyrrolo[3,2,1-hi]indole-4,5-dicarboxylate was also obtained following general procedure B: nitrile aziridine **3c** (40.0 mg, 0.25 mmol) and dimethyl acetylenedicarboxlyate (0.04 mL, 0.30 mmol) were stirred in MeCN (1.00 mL) at room temperature for 16 h. After concentrating to dryness the product was obtained by column chromatography (100% CH_2Cl_2, then a gradient of 5% EtOAc/CH_2Cl_2 to 20 % EtOAc). The title diester (68.0 mg, 94%) was obtained as a bright red solid. Mp.165-166°C (EtOAc). υ_{max}/cm^{-1} (film) 2929 w (C-H), 2213 s (CN) 1741 s (C=O) 1703 s (C=O), 1525 s (C=C). ^{1}H NMR (400 MHz, $CDCl_3$) δ_H 5.92 (1H, ddd, *J* = 9.5, 6.0, 3.0 Hz, CH-CH=C*H*), 5.68 (1H, dddd, *J* = 9.5, 6.5, 2.5, 1.5 Hz, CH-C*H*=CH), 5.50 (1H, dd, *J* = 6.0, 2.5 Hz, C*H*-CH=CH), 4.12 (1H, ddd, *J* = 11.0, 9.0, 1.5 Hz, N-CH_{2a}-CH_2), 3.89 (1H, td, *J* = 11.0, 7.0 Hz, N-CH_{2b}-CH_2), 3.86 (3H, s, CO_2CH_3), 3.77 (3H, s, CO_2CH_3), 2.93- 2.80 (1H, m, CH_2-C*H*-CH_2), 2.46 (1H, ddd, *J* = 17.0, 8.0, 6.5 Hz, CH=CH-CH_{2a}), 2.36 (1H, dt, *J* = 12.0, 7.0 Hz, N-CH_2-CH_{2a}), 2.01 (1H, tt, *J* = 17.0, 3.0 Hz, CH=CH-CH_{2b}), 1.69 (1H, qd, *J* = 12.0, 9.0 Hz, N-CH_2-CH_{2b}). ^{13}C NMR (101 MHz, $CDCl_3$) δ_c 164.2 (N-C=C-*C*OMe), 162.7 (N-C-*C*OMe), 154.9 (N-*C*=CCOMe), 141.5 (N-C=*C*-COMe), 124.6 (CH-CH=*C*H), 123.1 (CH-*C*H=CH), 116.4 (*C*N), 104.8 (*C*H-CH=CH), 81.1 (N-*C*-CN), 55.2 (N-*C*H_2-CH_2), 53.4 (N-C-CO-O*C*H_3), 52.5 (N-C=C-CO-O*C*H_3), 36.9 (CH_2-*C*H-CH_2), 30.0 (N-CH_2-*C*H_2), 29.4 (CH=CH-*C*H_2). m/z (ESI+) 311.101

(M+Na+), HRMS: (ESI+) calculated for $C_{15}H_{17}N_2O_4$: 289.1183. Found (M+H+) 289.1192. Anal. Calcd. for C, 62.49; H, 5.59; N, 9.72. Found C, 62.359; H, 5.715; N, 9.485.

(±)-(3*R*,5a*R*)-1,5a,8,8a-tetrahydropyrrolo[3,2,1-*hi*]indole-3,5(2*H*)-dicarbonitrile (Table 4, entry 4)

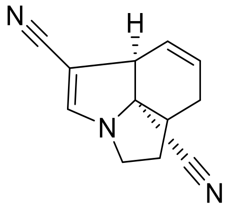

General procedure A for Pd catalysed [3+2] annulation reactions was followed, using propiolonitirile (0.06 mL, 0.50 mmol) as the dipolarophile and nitrile aziridine **3c** (40.0 mg, 0.25 mmol) in anhydrous dichloromethane (1.25 mL) to yield the title compound (18.0 mg, 37%) as a yellow solid.

(±)-(3*R*,5a*R*)-1,5a,8,8a-tetrahydropyrrolo[3,2,1-*hi*]indole-3,5(2*H*)-dicarbonitrile was also obtained following general procedure B: nitrile aziridine **3c** (40.0 mg, 0.25 mmol) and propiolnitirile (0.02 mL, 0.50 mmol) were stirred in MeCN (1.00 mL) at room temperature for 16 h. After concentrating to dryness the product was obtained by column chromatography (70/30 Pet/Et_2O). The title compound (9.00 mg, 18%) was obtained as a yellow solid. Mp. 164-165°C (EtOAc). υ_{max}/cm^{-1} (film) 2934 w (C-H), 2213 s (CN), 1598 s (C=C). ^{1}H NMR (400 MHz, $CDCl_3$) δ_H 7.48 (1H, s, N-C*H*=C), 5.99 (1H, ddd, *J* = 9.0, 6.0, 3.5 Hz, CH-C*H*=CH), 5.71 (1H, dddd, *J* = 1.5, 3.0, 6.5, 9.0 Hz, CH-CH=C*H*), 5.54 (1H, dd, *J* = 6.0, 3.0 Hz, C*H*-CH=CH), 4.41 (1H, dd, *J* = 12.0, 9.0 Hz, N-CH_{2a}-CH_2), 3.82 (1H, app td, *J* = 12.0, 7.0 Hz, N-CH_{2b}-CH_2), 2.97 – 2.84 (1H, m, CH_2-C*H*-CH_2), 2.59 – 2.40 (2H, m, CH=CH-CH_{2a}, N-CH_2-CH_{2a}), 2.03 (1H, app tt, *J* = 17.5, 3.5 Hz, CH=CH-CH_{2b}), 1.74 (1H, app qd, *J* = 12.0, 9.0 Hz, N-CH_2-CH_{2b}). ^{13}C NMR (101 MHz, $CDCl_3$) δ_c 147.5 (N-*C*H=C), 142.6 (NCH=*C*-CN), 123.7 (CH-*C*H=CH), 122.6 (CH-CH=*C*H), 115.9 (*C*N), 114.6 (*C*N), 99.4 (*C*H-CH=CH), 55.9 (N-*C*-CN), 50.1 (N-*C*H_2-CH_2), 36.7 (CH_2-*C*-CH_2), 30.3 (N-CH_2-*C*H_2), 29.3 (CH=CH-*C*H_2). m/z (ESI+) 220.083 (M+Na+), HRMS: (ESI+) calculated for $C_{12}H_{12}N_3$: 198.1026. Found (M+H+) 198.1017

(±)-(6a*R*,8a*R*)-7-Ethyl-1-((*E*)-3-oxobut-1-en-1-yl)-2,3,3a,4,6a,7-hexahydroazeto[2,3-h]indol-8(1*H*)-one (Table 5, entry 1)

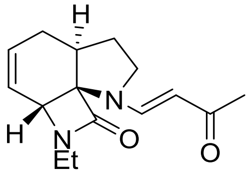

General procedure A was followed, using 3-butyn-2-one (0.04 mL, 0.50 mmol) as the dipolarophile and amide aziridine **3a** (48.0 mg, 0.25 mmol) in anhydrous dichloromethane (1.25 mL). Purification by column chromatography (100% EtOAc, then 5% MeOH/EtOAc), yielded the title compound (101 mg, 97%) as a yellow crystalline solid.

(±)-(6a*R*,8a*R*)-7-Ethyl-1-((*E*)-3-oxobut-1-en-1-yl)-2,3,3a,4,6a,7-hexahydroazeto[2,3-h]indol-8(1*H*)-one was also obtained following general procedure B stirring aziridine **3a** (48.0 mg) and 3-butyn-2-one (0.03 mL, 0.30 mmol) in MeCN (1.00 mL) at room temperature for 20 h. After concentration to dryness, purification by column chromatography (100% EtOAc, then 5% MeOH/EtOAc), gave the title lactam (53.0 mg, 82%) as a yellow crystalline solid. Mp. 88 – 89 °C (EtOAc). υ_{max}/cm^{-1} (film) 2977 w (C-H), 1746 s (C=O, lactam) 1603 s (C=O, ketone), 1558 (C=C). ^{1}H NMR (400 MHz, $CDCl_3$) δ_H 7.47 (1H, d, *J* = 13.0 Hz, N-C*H*=CH), 6.06 (1H, ddd, *J* = 10.0, 6.0, 3.5 Hz, CH=C*H*-CH_2), 5.89 (1H, dt, *J* = 10.0, 3.0 Hz C*H*=CH-CH_2), 5.14 (1H, d, *J* = 13.0 N-CH=C*H*), 3.86 (1H, brs, N-C*H*-CH=CH), 3.39 (1H, dq, *J* = 14.0, 7.0 Hz, CON-CH_{2a}-CH_3), 3.33 – 3.23 (2H, m, N-CH_2-CH_2), 3.18 (1H, dq, *J* = 14.0, 7.0 Hz, CON-CH_{2b}-CH_3), 2.74 – 2.64 (1H, m, CH_2-C*H*-CH_2), 2.33 (1H, ddt, *J* = 17.5, 6.0, 3.0 Hz, CH=CH-CH_{2a}), 2.16 – 1.97 (5H, m, CH=CH-C$H_{2b,}$ CO-CH_3, N-CH_2-CH_{2a}), 1.78 (1H, ddt, *J* = 12.0, 9.5, 9.0 Hz, N-CH_2-CH_{2b}), 1.21 (3H, t , *J* = 7.0 Hz, CH_2-CH_3). ^{13}C NMR (101 MHz, $CDCl_3$) δ_c 195.4 (*C*OCH_3), 167.6 (*C*ONHEt), 144.6 (N-*C*H=CH), 132.1 (CH=*C*H-CH_2), 122.9 (*C*H=CH-CH_2), 100.0 (N-CH=*C*H), 77.9 (CO-*C*-N), 57.4 (N-*C*H-CH=CH), 47.1 (N-*C*H_2-CH_2), 37.9 (CH_2-*C*H-CH_2), 35.7 (N-*C*H_2-CH_3), 27.9 (CO*C*H_3), 27.3 (N-CH_2-*C*H_2), 24.9 (CH=CH-*C*H_2), 13.5 (N-CH_2-*C*H_3). m/z (ESI+) 261.159 (M+H+), HRMS: (ESI+) calculated for $C_{15}H_{21}N_2O_2$: 261.1598. Found (M+H+) 261.1594. Anal. Calcd. for C, 69.20; H, 7.74; N, 10.76. Found C, 68.66; H, 7.53; N, 10.71.

(±)-Methyl (*E*)-3-((6a*R*,8a*R*)-7-ethyl-8-oxo-3,3a,4,6a,7,8-hexahydroazeto[2,3-h]indol-1(2*H*)-yl) acrylate (Table 5, entry 2)

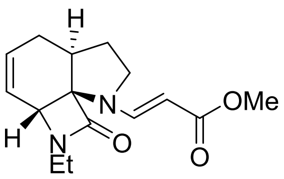

General procedure A was followed, using methyl propiolate (0.03 mL, 0.50 mmol) as the dipolarophile and aziridine **3a** (48.0 mg, 0.25 mmol) in anhydrous dichloromethane (1.25 mL) to yield the title lactam (58.0 mg, 83%) as an off white oil.

(±)-Methyl (*E*)-3-((6a*R*,8a*R*)-7-ethyl-8-oxo-3,3a,4,6a,7,8-hexahydroazeto[2,3-h]indol-1(2*H*)-yl) acrylate was also obtained following general procedure B stirring aziridine **3a** (48.0 mg) and methyl propiolate (0.03 mL, 0.30 mmol) in MeCN (1.00 mL) at room temperature for 24 h. After concentration to dryness, purification by column chromatography (10% EtOAc/CH_2Cl_2, then 20% EtOAc/CH_2Cl_2), gave the title lactam (38.0 mg, 54%) as an off white oil. υ_{max}/cm^{-1} (film) 2975 w (C-H), 1748 s (C=O, lactam) 1688 s (C=O, ester), 1607 (C=C). ^{1}H NMR (400 MHz, $CDCl_3$) δ_H 7.49 (1H, d, *J* = 13.0 Hz, N-C*H*=CH), 6.06 (1H, ddd, CH=C*H*-CH_2), 5.88 (1H, dt, *J* = 9.5, 3.0 Hz C*H*=CH-CH_2), 4.64 (1H, d, *J* = 13.0 N-CH=C*H*), 3.85 (1H, brs, N-C*H*-CH=CH), 3.63 (3H, s, O-CH_3), 3.40 (1H, dq, *J* = 14.5, 7.0 Hz, CON-CH_{2a}-CH_3), 3.29 – 3.11 (4H, m, N-CH_2-CH_2, CON-CH_{2b}-CH_3), 2.71 – 2.62 (1H, m, CH_2-C*H*-CH_2), 2.32 (1H, ddt, *J* = 17.5, 6.0, 3.0 Hz, CH=CH-CH_{2a}), 2.11 (1H, dt, *J* = 17.5, 4.5 Hz, CH=CH-CH_{2b}), 2.00 (1H, dtd, *J* = 10.5, 7.0, 4.5 Hz, N-CH_2-CH_{2a}), 1.78 (1H, ddt, *J* = 12.0, 10.5, 8.5 Hz, N-CH_2-CH_{2b}), 1.19 (3H, t, *J* = 7.0 Hz, CONH-CH_2-CH_3). ^{13}C NMR (101 MHz, $CDCl_3$) δ_C 169.0 (*C*OOMe), 167.9 (*C*ONHEt), 144.9 (N-*C*H=CH), 132.0 (CH=*C*H-CH_2), 123.0 (*C*H=CH-CH_2), 88.6 (N-CH=*C*H), 77.7 (CO-*C*-N), 57.6 (N-*C*H-CH=CH), 50.6 (CO_2-*C*H_3), 46.9 (N-*C*H_2-CH_2), 38.0 (CH_2-*C*H-CH_2), 35.6 (CON-*C*H_2-CH_3), 27.4 (N-CH_2-*C*H_2), 24.9 (CH=CH-*C*H_2), 13.4 (CON-CH_2-*C*H_3). M/z (ESI+) 277.156 (M+H+), HRMS: (ESI+) calculated for $C_{15}H_{21}N_2O_3$: 277.1547. Found (M+H+) 277.1555

(±)-Ethyl (*E*)-3-((6a*R*,8a*R*)-7-ethyl-8-oxo-3,3a,4,6a,7,8-hexahydroazeto[2,3-h]indol-1(2*H*)-yl) acrylate (Table 5, entry 3)

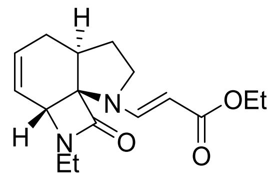

General procedure A was followed, using ethyl propiolate (0.05 mL, 0.50 mmol) as the dipolarophile and amide aziridine **3a** (48.0 mg, 0.25 mmol) in anhydrous dichloromethane (1.25 mL) to yield the title compound (57.0 mg, 78%) as a yellow oil.

(±)-Ethyl (*E*)-3-((6a*R*,8a*R*)-7-ethyl-8-oxo-3,3a,4,6a,7,8-hexahydroazeto[2,3-h]indol-1(2*H*)-yl) acrylate was also obtained following general procedure B, stirring amide aziridine **3a** (48.0 mg) and ethyl propiolate (0.03 mL, 0.30 mmol) in MeCN (1.00 mL) at room temperature for 47 h. After concentration to dryness, purification by column chromatography (30% EtOAc/CH_2Cl_2), gave the title lactam (44.0 mg, 61%) as a yellow oil. $υ_{max}$/cm^{-1} (film) 2985 w (C-H), 1746 s (C=O, lactam) 1687 s (C=O, ester), 1600 (C=C). ^{1}H NMR (400 MHz, $CDCl_3$) $δ_H$ 7.48 (1H, d, *J* = 13.0 Hz, N-C*H*=CH), 6.05 (1H, ddd, *J* = 10.0, 5.5, 3.5 Hz, CH=C*H*-CH_2), 5.89 (1H, dt, *J* = 10.0, 3.5 Hz C*H*=CH-CH_2), 4.63 (1H, d, *J* = 13.0 N-CH=C*H*), 4.08 (2H, q, *J* = 7.0 Hz, COO-CH_2-CH_3), 3.84 (1H, brs, N-C*H*-CH=CH), 3.39 (1H, dq, *J* = 14.5, 7.0 Hz, CON-CH_{2a}-CH_3), 3.28 – 3.19 (2H, m, N-CH_2-CH_2), 3.13 (1H, dq, *J* = 14.5, 7.2 Hz, CON-CH_{2b}-CH_3), 2.66 (1H, dtd, *J* = 10.0, 6.0, 4.5, Hz, CH_2-C*H*-CH_2), 2.31 (1H, ddt, *J* = 17.0, 6.0, 3.5 CH=CH-CH_{2a}), 2.09 (1H, dt, *J* = 17.0 Hz, CH=CH-CH_{2b}), 2.01 (1H, dtd, *J* = 12.5, 6.5, 4.5 Hz, N-CH_2-CH_{2a}), 1.88 – 1.67 (1H, m, N-CH_2-CH_{2b}), 1.23 (3H, t, *J* = 7.0 Hz, CO-CH_2-CH_3), 1.19 (3H, t, *J* = 7.0 Hz, CONH-CH_2-CH_3). ^{13}C NMR (101 MHz, $CDCl_3$) $δ_c$ 168.7 (*C*OOEt), 167.9 (*C*ONHEt), 144.7 (N-*C*H=CH), 132.0 (CH=*C*H-CH_2), 123.0 (*C*H=CH-CH_2), 89.0 (N-CH=*C*H), 77.7 (CO-*C*-N), 59.0 (CO-*C*H_2-CH_3), 56.6 (N-*C*H-CH=CH), 47.1 (N-*C*H_2-CH_2), 37.9 (CH_2-*C*H-CH_2), 35.6 (CON-*C*H_2-CH_3), 27.5 (N-CH_2-*C*H_2), 24.9 (CH=CH-*C*H_2), 14.5 (COO-CH_2-*C*H_3), 13.5 (CON-CH_2-*C*H_3). m/z (ESI+) 291.170 (M+H+), HRMS: (ESI+) calculated for $C_{16}H_{23}N_2O_3$: 291.1703. Found (M+H+) 291.1702.

(±)-(3*R*,8a*S*)-*N*-ethyl-2-((trimethylsilyl)ethynyl)-5,5a,6,8a-tetrahydro-2*H*-oxazolo[5,4,3-hi]indole-3(4*H*)-carboxamide and (±)-(*E*)-3-((6aR,8aR)-7-ethyl-8-oxo-3,3a,4,6a,7,8-hexahydroazeto[2,3-h]indol-1(2*H*)-yl)acrylaldehyde (Table 5, entry 5)

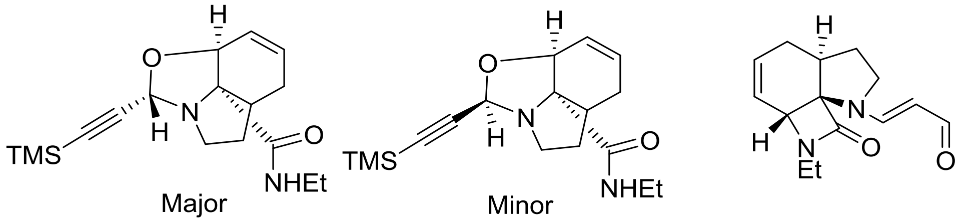

General procedure A was followed, using trimethylsilyl propynal (0.14 mL, 1.00 mmol) as the dipolarophile and aziridine **3a** (96.0 mg, 0.50 mmol) in anhydrous dichloromethane (2.50 mL) to yield the title oxaxolidine (24.0 mg, 15%) as an inseperable mixture of diastereomers (d.r. 1.5:1), as a yellow oil. υ_{max}/cm^{-1} (film) 3366 w (N-H), 2962 w (C-H), 2891 w (C-H), 1667 s (C=O), 1517 m (C=C). ^{1}H NMR (400 MHz, $CDCl_3$) δ_H 7.55 (2H, brs, N-*H*, both diastereomers), 6.05 – 5.90 (4H, m, C*H*=C*H*, both diastereomers), 5.23 (1H, s, N-C*H*-O, major diastereomer), 4.86 (1H, s, N-C*H*-O, minor diastereomer), 4.48 (1H, s, C*H*-CH=CH, major diastereomer), 4.03 (1H, s, C*H*-CH=CH, minor diastereomer), 3.68 (1H, ddd, *J* = 11.5, 7.5, 3.0 Hz, N-CH_{2a}-CH_2, minor diastereomer), 3.40-3.15 (5H, m, N-CH_{2a}-CH_2 major diastereomer, CO_2NHCH_2, both diastereomers), 3.05 (1H, app dt, *J* = 11.5, 7.5 Hz, N-CH_{2b}-CH_2, minor diastereomer), 2.95 (1H, ddd, *J* = 11.5, 7.5, 2.0 Hz, N-CH_{2b}-CH_2, major diastereomer), 2.68 – 2.52 (2H, m, CH=CH-CH_{2a}, both diastereomers), 2.48 -2.34 (2H, m, CH_2-C*H*-CH_2, both diastereomers), 2.22 – 2.14 (2H, m, CH=CH-CH_{2b}, both diastereomers), 1.79 – 1.59 (4H, m, N-CH_2-CH_2, both diastereomers), 1.16 (3H, t, *J* = 7.5 Hz, $CO_2NHCH_2CH_3$, major diastereomer), 1.14 (3H, t, *J* = 7.0 Hz, $CO_2NHCH_2CH_3$, minor diastereomer), 0.20 (9H, s, Si-Me_3, major diastereomer), 0.17 (9H, s, Si-Me_3, minor diastereomer). ^{13}C NMR (101 MHz, $CDCl_3$) δ_c 174.3 (*C*ONHEt, major diastereomer), 173.7 (*C*ONHEt, minor diastereomer) 128.7 (CH=*C*H-CH_2, major diastereomer), 128.2 (CH=*C*H-CH_2, minor diasteromer), 123.2 (*C*H=CH-CH_2, major diastereomer), 123.1 (*C*H=CH-CH_2, minor diastereomer), 101.7 (Si-*C*, major diastereomer), 97.9 (Si-*C*, minor diastereomer), 94.7 (Si-C-*C*, minor diastereomer), 90.0 (Si-C-*C*, major diastereomer), 88.3 (N-*C*H-O, major diastereomer), 83.9 (N-*C*H-O, minor diastereomer), 75.6 (N-*C*-CONHEt, minor diastereomer), 75.4 (N-*C*-CONHEt, major diastereomer), 73.5 (O-*C*H-CH=CH, minor diastereomer), 72.5 (O-*C*H-CH=CH, major diastereomer), 56.2 (N-CH_2-CH_2, major diastereomer), 49.5 (N-CH_2CH_2, minor diastereomer), 40.8 (CH_2-*C*H-CH_2, major diasteromer), 40.4 (CH_2-*C*H-CH_2, minor diasteromer), 33.7 (CONHCH_2, major diasteromer), 33.6 (CONHCH_2, minor diastereomer), 32.1 (N-CH_2-CH_2, major diastereomer), 31.9 (N-CH_2-CH_2, minor diastereomer), 26.1 (CH=CH-CH_2, major diastereomer), 26.0 (CH=CH-CH_2, minor diastereomer), 14.9 ($CONHCH_2CH_3$, minor diastereomer), 14.8 ($CONHCH_2CH_3$, major diasteromer), -0.28 (Si-$(CH_3)_3$, major diastereomer), -0.43(Si-$(CH_3)_3$, minor diastereomer). m/z (ESI+) 319.184 (M+H+), HRMS: (ESI+) calculated for $C_{17}H_{27}N_2O_2Si$: 319.1836. Found (M+H+) 319.1839

Further elution gave (±)-(*E*)-3-((6aR,8aR)-7-ethyl-8-oxo-3,3a,4,6a,7,8-hexahydroazeto[2,3-h]indol-1(2*H*)-yl)acrylaldehyde (29.0 mg, 46%) as a yellow oil. υ_{max}/cm^{-1} (film) 2933 w (C-H), 1744 s (C=O, lactam) 1650 s (C=O, aldehyde), 1597 (C=C). ^{1}H NMR (400 MHz, $CDCl_3$) δ_H 9.08 (1H, d, *J* = 8.0 Hz, CO*H*), 7.13 (1H, d, *J* = 14.0 Hz, N-C*H*=CH-CHO), 6.09 (1H, ddd, *J* = 9.5, 6.0, 3.0 Hz, CH-CH=C*H*), 5.89 (1H, ddd, *J* = 9.5, 4.5, 3.0 Hz, CH-C*H*=CH), 5.17 (1H, dd, *J* = 14.0, 8.0 Hz, N-CH=C*H*-CHO), 3.83 (1H, s, C*H*-CH=CH), 3.39 (1H, dq, *J* = 14.0, 7.0 Hz, CON-CH_{2a}), 3.34 – 3.24 (2H, m, N-CH_2-CH_2), 3.16 (1H, dq, *J*

= 14.0, 7.0 Hz, CONH-CH_{2b}), 2.71 (1H, dtd, *J* = 10.0, 6.5, 3.5 Hz, CH_2-C*H*-CH_2), 2.35 (1H, ddt, *J* = 17.0, 6.5, 3.0 Hz, CH=CH-CH_{2a}), 2.17 (1H, ddd, *J* = 17.0, 6.0, 3.5 Hz, CH=CH-CH_{2b}), 2.06 – 1.96 (1H, m, N-CH_2-CH_{2a}), 1.82 (1H, dt, *J*= 19.0, 5.5 Hz, N-CH_2-CH_{2b}), 1.18 (3H, t, *J* = 7.0 Hz, $CONCH_2$-CH_3). ^{13}C NMR (101 MHz, $CDCl_3$) δ_c 189.5 (*C*HO), 166.9 (*C*ONEt), 151.9 (N-*C*H=CH-CHO), 132.2 (CH-CH=*C*H), 122.7 (CH-*C*H=CH), 104.8 (N-CH=*C*H-CHO), 78.2 (N-*C*-CO), 57.4 (N-*C*H-CH=CH), 47.2 (N-*C*H_2-CH_2), 37.7 (CH_2-*C*H-CH_2), 35.8 (CON-*C*H_2-CH_3), 27.2 (N-CH_2-*C*H_2), 24.7 (CH=CH-*CH$_2$*), 13.4 (CONCH-*C*H_3). m/z (ESI+) 269.126 (M+Na+), HRMS: (ESI+) calculated for $C_{14}H_{19}N_2O_2$: 247.1441. Found (M+H+) 247.1440.

(±)-(*E*)-3-((6aR,8aR)-7-ethyl-8-oxo-3,3a,4,6a,7,8-hexahydroazeto[2,3-h]indol-1(2*H*)-yl)acrylaldehyde and (±)-(*E*)-3-((3a*S*,6a*R*,8a*R*,*E*)-8-(ethylimino)-3,3a,4,6a-tetrahydro-8*H*-oxeto[2,3-h]indol-1(2*H*)-yl)acrylaldehyde (Table 5, entry 5)

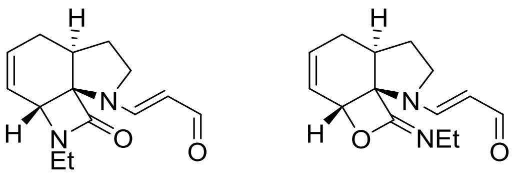

General procedure B was followed stirring aziridine **3a** (48.0 mg) and trimethylsilyl propynal (0.05 mL, 0.30 mmol) in MeCN (1.00 mL) at room temperature for 20 h. After concentrating to dryness, purification by column chromatography (100% EtOAc, then 5% MeOH/EtOAc), gave a mixture an inseparable mixture of the title lactam (31.5 mg, 51%) and the tentatively assigned *O*-cyclised imine (5.5 mg, 9%) as a yellow oil. Spectroscopic properties of the lactam compound are consistent with those reported above.

Tentatively assigned *O*-cyclised imine

υ_{max}/cm^{-1} (film) 2933 w (C-H), 1744 s (C=O, lactam) 1650 s (C=O, aldehyde), 1597 (C=C). ^{1}H NMR (400 MHz, $CDCl_3$) δ_H 9.02 (1H, d, *J* = 8.5 Hz, CO*H*), 7.21 (1H, d, *J* = 12.5 Hz, N-C*H*=CH-CHO), 5.73 (1H, ddd, *J* = 10.0, 6.5, 3.5 Hz, CH-CH=C*H*), 5.59 (1H, ddd, *J* = 10.0, 4.0, 2.0 Hz, CH-C*H*=CH), 5.09 (1H, dd, *J* = 12.5, 8.5 Hz, N-CH=C*H*-CHO), 4.29 (1H, ddd, *J* = 4.0, 3.5, 2.0 Hz, C*H*-CH=CH), 3.45 -3.05 (5H, m, CON-CH_2, N-CH_2-CH_2, CH_2-C*H*-CH_2), 2.44 (1H, dddd, *J* = 17.0, 8.0, 4.0, 2.0 Hz, CH=CH-CH_{2a}), 2.10- 2.01 (1H, m, CH=CH-CH_{2b}), 2.06 – 1.96 (1H, m, N-CH_2-CH_{2a}), 1.81- 1.67 (1H, m, N-CH_2-CH_{2b}), 1.10 (3H, t, *J* = 7.0 Hz, $CONCH_2$-CH_3). ^{13}C NMR (101 MHz, $CDCl_3$) δ_c 189.5 (*C*HO), 168.2 (O*C*=NEt), 155.4 (N-*C*H=CH-CHO), 128.5 (CH-*C*H=CH), 128.1 (CH-CH=*C*H), 103.3 (N-CH=*C*H-CHO), 72.5 (N-*C*-CO), 70.9 (N-*C*H-CH=CH), 46.9 (N-*C*H_2-CH_2), 40.6 (CH_2-*C*H-CH_2), 34.6 (CON-*C*H_2-CH_3), 29.6 (N-CH_2-*C*H_2), 26.1 (CH=CH-*CH$_2$*), 14.7 (CONCH-*C*H_3). m/z (ESI+) 269.126 (M+Na+), HRMS: (ESI+) calculated for $C_{14}H_{19}N_2O_2$: 247.1441. Found (M+H+) 247.1440.

(±)-Dimethyl 2-((6a*R*,8a*R*)-7-ethyl-8-oxo-3,3a,4,6a,7,8-hexahydroazeto[2,3-h]indol-1(2*H*)-yl) maleate (Table 5, entry 6)

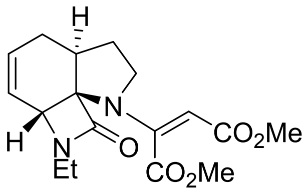

General procedure A was followed, using dimethyl acetylenedicarboxylate (0.06 mL, 0.50 mmol) as the dipolarophile and amide aziridine **3a** (48.0 mg, 0.25 mmol) in anhydrous dichloromethane (1.25 mL) to yield the title compound (27.0 mg, 32%) as a yellow oil.

(±)-Dimethyl 2-((6a*R*,8a*R*)-7-ethyl-8-oxo-3,3a,4,6a,7,8-hexahydroazeto[2,3-h]indol-1(2*H*)-yl) maleate was also obtained following general procedure B stirring aziridine **3a** (48.0 mg) and dimethyl acetylenedicarboxylate (0.04 mL, 0.30 mmol) in MeCN (1.00 mL) at room temperature for 6.5 h. After concentration to dryness, purification by column chromatography (100% CH_2Cl_2, then a 5% gradient of EtOAc up to 20% EtOAc/CH_2Cl_2), gave the title lactam (40.0 mg, 48%) as a yellow oil. υ_{max}/cm^{-1} (film) 2952 w (C-H), 1748 s (C=O, lactam), 1697 s (C=O, ester), 1570 m (C=C). ^{1}H NMR (400 MHz, $CDCl_3$) 6.08 (1H, ddd, CH=C*H*-CH_2), 5.88 (1H, ddd, *J* = 10.0, 5.0, 3.0 Hz C*H*=CH-CH_2), 4.73 (1H, s, N-C=C*H*), 4.32 (1H, d, *J* = 5.0 Hz, N-C*H*-CH=CH), 3.86 (3H, s, O-CH_3), 3.60 (3H, s, O-CH_3), 3.46-3.27 (3H, m, CON-CH_{2a}-CH_3, N-CH_2-CH_2), 3.07 (1H, dq, *J* = 14.0, 7.0 Hz, CON-CH_{2b}-CH_3), 2.73 (1H, dtd, *J* = 11.0, 6.0, 3.0 Hz, CH_2-C*H*-CH_2), 2.32 (1H, ddt, *J* = 17.0, 6.0, 2.5 Hz, CH=CH-CH_{2a}), 2.16 (1H, ddd, *J* = 17.0, 6.0, 3.5 Hz, CH=CH-CH_{2b}), 1.89 (1H, dtd, *J* = 12.0, 6.0, 3.5 Hz, N-CH_2-CH_{2a}), 1.81 – 1.70 (1H, m, N-CH_2-CH_{2b}), 1.19 (3H, t, *J* = 7.0 Hz, CONH-CH_2-CH_3). ^{13}C NMR (101 MHz, $CDCl_3$) δ_c 167.6 (*C*ONHEt), 167.3 (*C*OMe), 165.2 (*C*OMe), 150.5 (N-*C*=CH), 132.2 (CH=*C*H-CH_2), 123.1 (*C*H=CH-CH_2), 96.7 (N-*C*H-CH=CH), 87.7 (N-C=*C*H), 77.2 (CO-*C*-N), 52.9 (CO_2-*C*H_3), 50.9 (CO_2-*C*H_3), 49.0 (N-*C*H_2-CH_2), 40.0 (CH_2-*C*H-CH_2), 35.3 (CON-*C*H_2-CH_3), 26.4 (N-CH_2-*C*H_2), 24.7 (CH=CH-*C*H_2), 12.7 (CONH-CH_2-*C*H_3). m/z (ESI+) 357.142 (M+Na+), HRMS: (ESI+) calculated for $C_{17}H_{23}N_2O_5$: 335.1601. Found (M+H+) 335.1595.

Bibliography

1. L. D. Elliott, J. P. Knowles, P. J. Koovits, K. G. Maskill, M. J. Ralph, G. Lejeune, L. J. Edwards, R. I. Robinson, I. R. Clemens, B. Cox, D. D. Pascoe, G. Koch, M. Eberle, M. B. Berry and K. I. Booker-Milburn, *Chem. Eur. J.*, 2014, **20**, 15226–15232

2. K. G. Maskill, J. P. Knowles, L. D. Elliott, R. W. Alder and K. I. Booker-Milburn, *Angew. Chem. Int. Ed.*, 2013, **52**, 1499–1502.

3. P. Ballesteros, B. W. Roberts, J. Wong, *J. Org. Chem.* 1983, **48**, 3603- 3605.

X-ray diffraction experiments on **10a**, **20a** and **22a** were carried out at 100K on a Bruker APEX II diffractometer using Mo-K_{α} radiation (λ = 0.71073 Å). Data collections were performed using a CCD area detector from a single crystal mounted on a glass fibre. Intensities were integrated [A] using SAINT and absorption corrections were based on equivalent reflections using SADABS.[B] The structures were solved using Superflip [C], all of the structures were refined against F^2 in SHELXL [D] using Olex2.[E] All of the non-hydrogen atoms were refined anisotropically. While all of the hydrogen atoms were located geometrically and refined using a riding model. Crystal structure and refinement data are given in Table 1.

[A] Bruker-AXS SAINT V8.27B Madison, Wisconsin.

[B] Sheldrick, G. M. SADABS V2012/1, University of Göttingen, Germany.

[C] Palatinus, L. & Chapuis, G. (2007). J. Appl. Cryst., 40, 786-790; Palatinus, L. & van der Lee, A. (2008). *J. Appl. Crystallogr.* **41**, 975-984; Palatinus, L., Prathapa, S. J. & van Smaalen, S. (2012). *J. Appl. Crystallogr.* **45**, 575-580.

[D] Sheldrick, G. M. (2008) *Acta Crystallogr. A*, **64**, 112.

[E] Dolomanov, O. V.; Bourhis, L. J.; Gildea, R. J.; Howard, J. A. K.; Puschmann, H. (2009) *J. Appl. Crystallogr.*, **42**, 339-341.

Table 1 Crystal data and structure refinement for **10a, 20a** and **22a**.

Identification code	**10a**	**20a**	**22a**
Empirical formula	$C_{21}H_{23}N_4O$	$C_{25}H_{28}N_4O_5S$	$C_{25}H_{29}N_3O_3S$
Formula weight	347.43	496.57	451.57
Temperature/K	100(2)	100(2)	100(2)
Crystal system	orthorhombic	monoclinic	monoclinic
Space group	*Pccn*	*Pc*	$P2_1/n$
a/Å	20.862(3)	7.4546(4)	13.2995(5)
b/Å	10.4975(17)	18.7124(10)	10.5425(4)
c/Å	16.690(3)	34.4314(19)	16.0040(6)
β/°	90	91.776(3)	93.024(2)
Volume/Å^3	3655.2(10)	4800.6(4)	2240.79(15)
Z	8	8	4
ρ_{calc}g/cm^3	1.259	1.374	1.339
μ/mm^{-1}	0.080	0.180	0.177
F(000)	1472.0	2096.0	960.0
Crystal size/mm^3	0.39 × 0.36 × 0.29	0.35 × 0.2 × 0.18	0.39 × 0.31 × 0.14
Radiation	MoKα (λ = 0.71073)	MoKα (λ = 0.71073)	MoKα (λ = 0.71073)
2Θ range for data collection/°	3.904 to 55.178	2.176 to 52.742	3.882 to 56.168
Index ranges	$-27 \le h \le 26$,	$-9 \le h \le 9$,	$-17 \le h \le 17$,

	$-13 \le k \le 10$, $-21 \le l \le 21$	$-23 \le k \le 23$, $-43 \le l \le 43$	$-13 \le k \le 13$, $-21 \le l \le 21$
Reflections collected	27920	113630	40020
R_{int}	0.0710	0.0971	0.0913
Data/restraints/parameters	4220/0/236	19637/2/1269	5414/0/295
Goodness-of-fit on F^2	1.041	1.043	1.051
Final R indexes [I>=2σ (I)]	$R_1 = 0.0503$, $wR_2 = 0.1068$	$R_1 = 0.0585$, $wR_2 = 0.1154$	$R_1 = 0.0428$, $wR_2 = 0.0953$
Final R indexes [all data]	$R_1 = 0.0867$, $wR_2 = 0.1232$	$R_1 = 0.0796$, $wR_2 = 0.1245$	$R_1 = 0.0527$, $wR_2 = 0.1009$
Largest diff. peak/hole / e $Å^{-3}$	0.22/-0.23	0.32/-0.40	0.35/-0.45
Flack parameter	-	0.08(4)	-

[A] Bruker-AXS SAINT V8.27B Madison, Wisconsin.

[B] Sheldrick, G. M. SADABS V2012/1, University of Göttingen, Germany.

[C] Palatinus, L. & Chapuis, G. (2007). J. Appl. Cryst., 40, 786-790; Palatinus, L. & van der Lee, A. (2008). *J. Appl. Crystallogr.* **41**, 975-984; Palatinus, L., Prathapa, S. J. & van Smaalen, S. (2012). *J. Appl. Crystallogr.* **45**, 575-580.

[D] Sheldrick, G. M. (2008) *Acta Crystallogr. A*, **64**, 112.

[E] Dolomanov, O. V.; Bourhis, L. J.; Gildea, R. J.; Howard, J. A. K.; Puschmann, H. (2009) *J. Appl. Crystallogr.*, **42**, 339-341.
