# Supplementary material for: Combining photochemistry and catalysis: rapid access to sp3 – rich polyheterocycles from simple pyrroles
Source: Chem Sci. 2016 Jan 4;7(3):2302–7. doi: 10.1039/c5sc04062k (PMC5977501; doi:10.1039/c5sc04062k)

Electronic Supplementary Material (ESI) for Chemical Science.
This journal is © The Royal Society of Chemistry 2016

(±)-(3a*S*,7*R*,7a*S*)-N-ethyl-7-(phenylthio)-1,2,3,3a,4,7-hexahydro-7aH-indole-7a-carboxamide

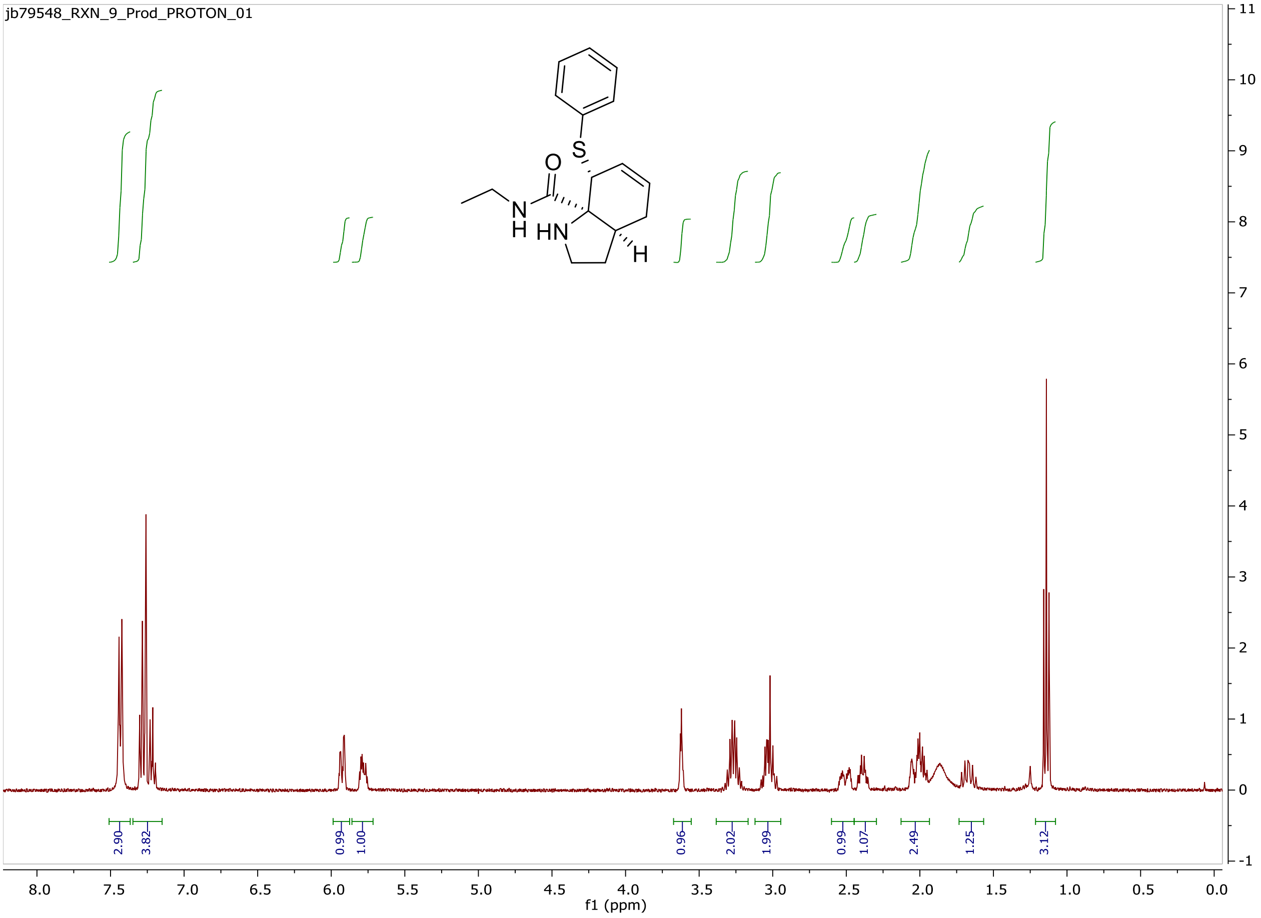

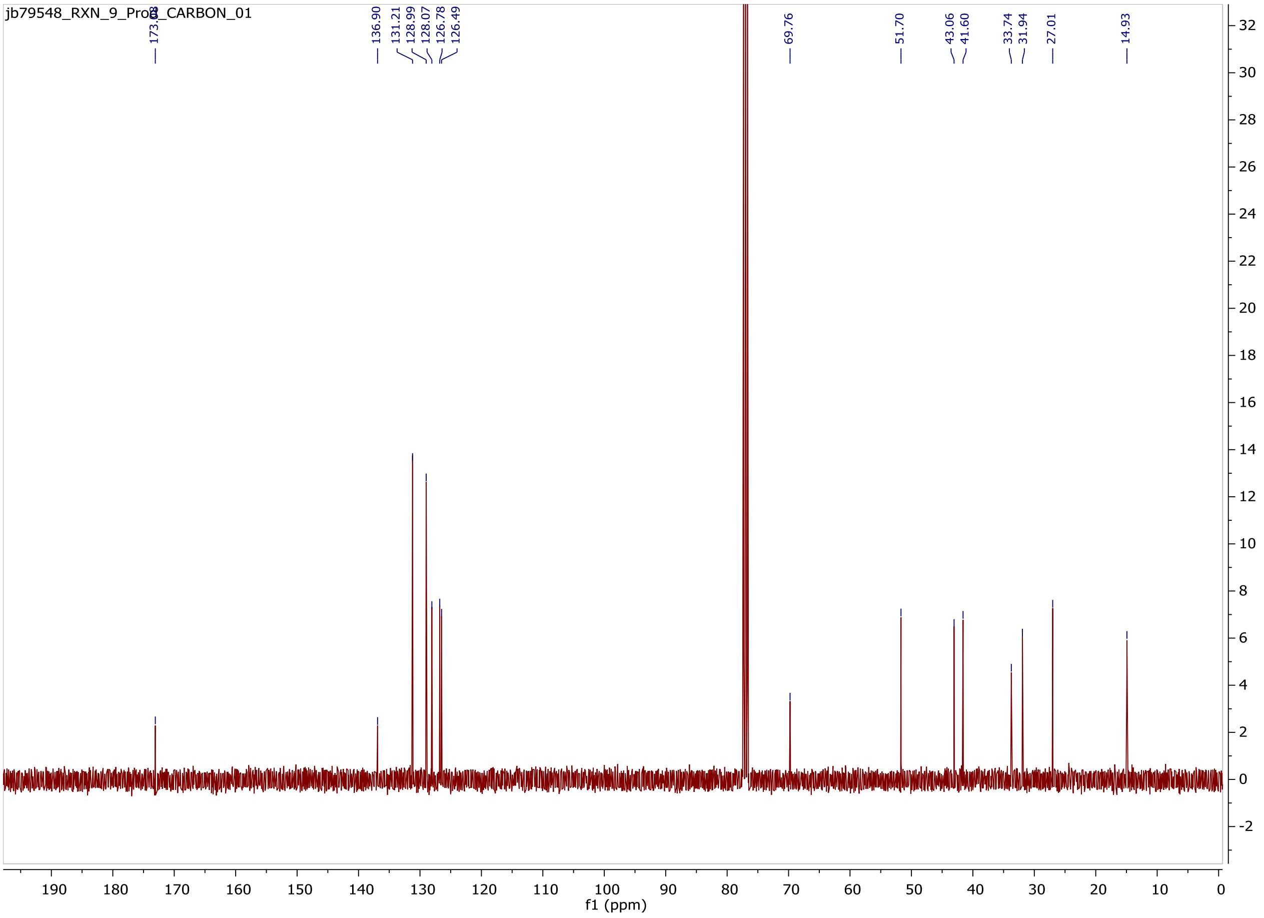

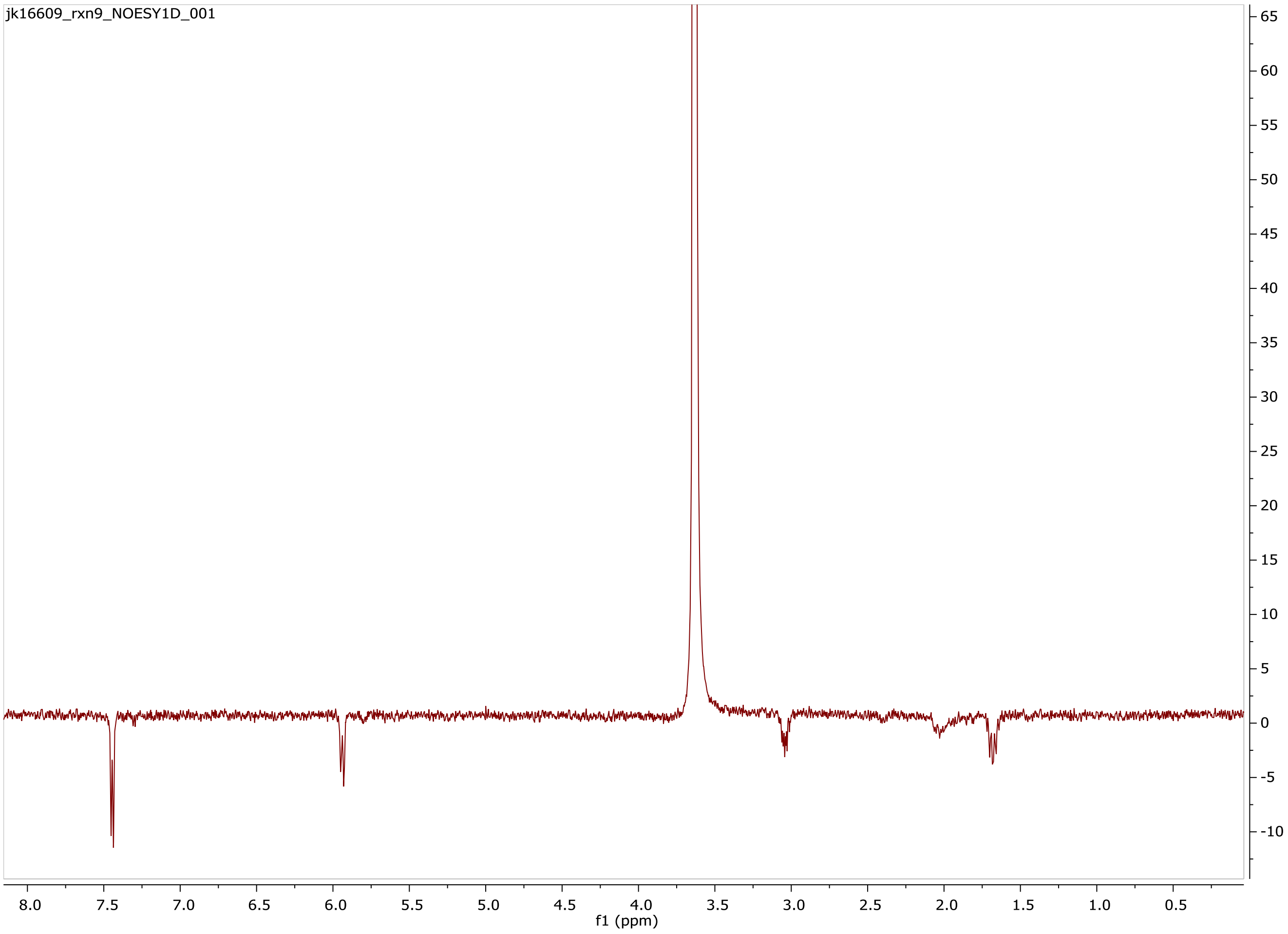
jk16609_rxn9_NOESY1D_001
65
60
55
50
45
40
35
30
25
20
15
10
5
0
-5
-10
8.0
7.5
7.0
6.5
6.0
5.5
5.0
4.5
4.0
3.5
3.0
2.5
2.0
1.5
1.0
0.5
f1 (ppm)

(±)-(3a*R*,5*S*,7a*R*)-*N*-ethyl-5-phenoxy-1,2,3,3a,4,5-hexahydro-7aH-indole-7a-carboxamide

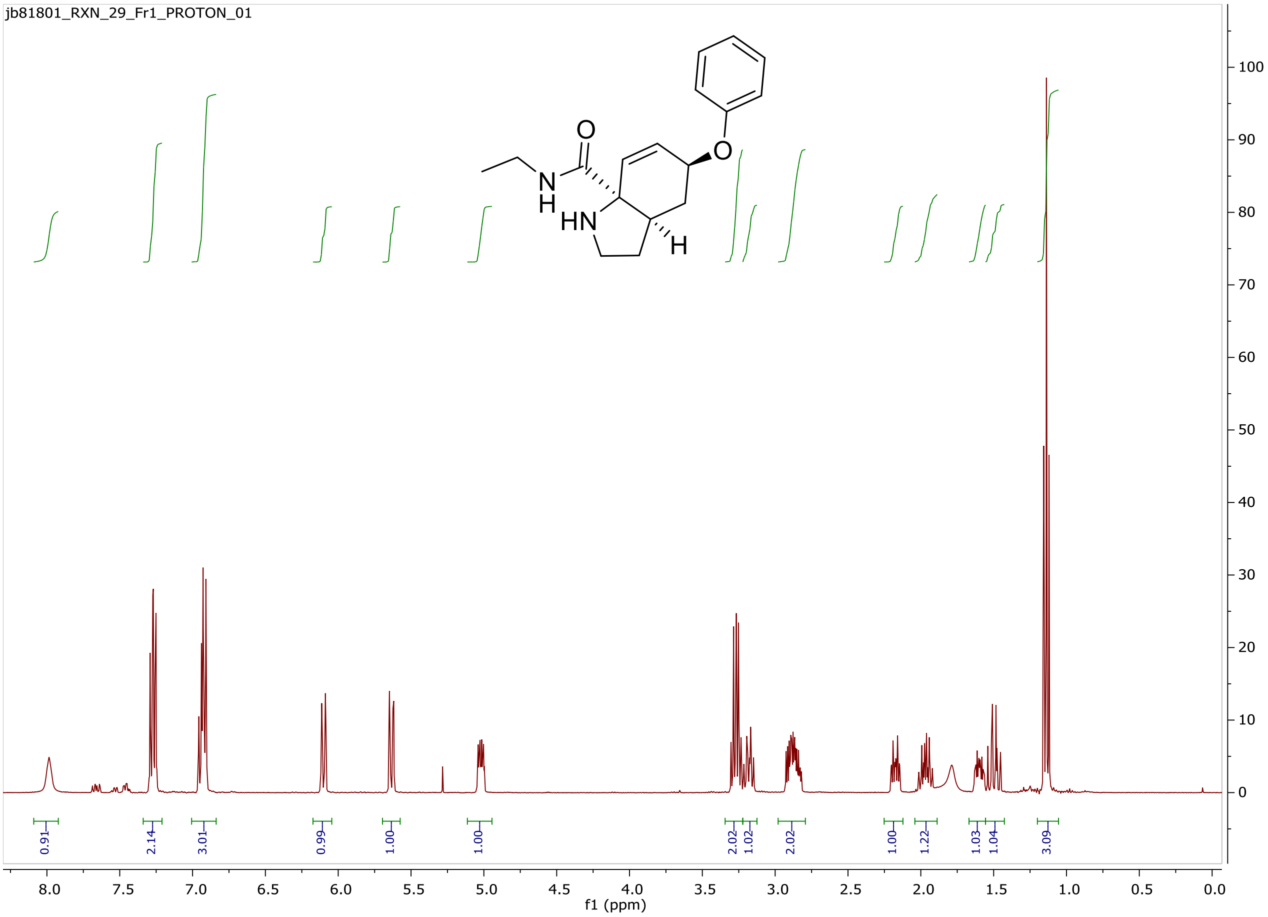

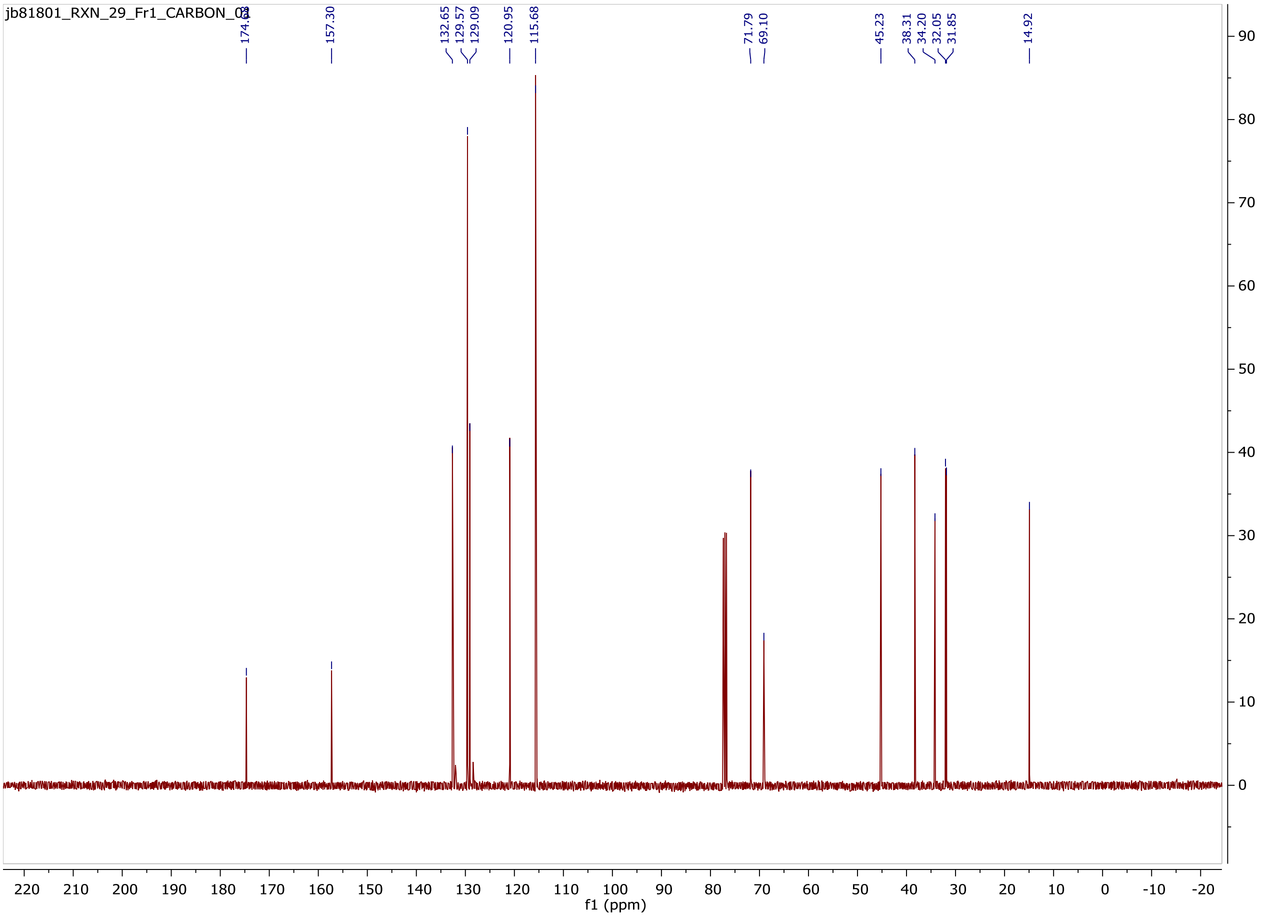

(±)-(3a*R*,5*S*,7a*R*)-*N*-ethyl-5-phenoxy-1,2,3,3a,4,5-hexahydro-7aH-indole-7a-carboxamide

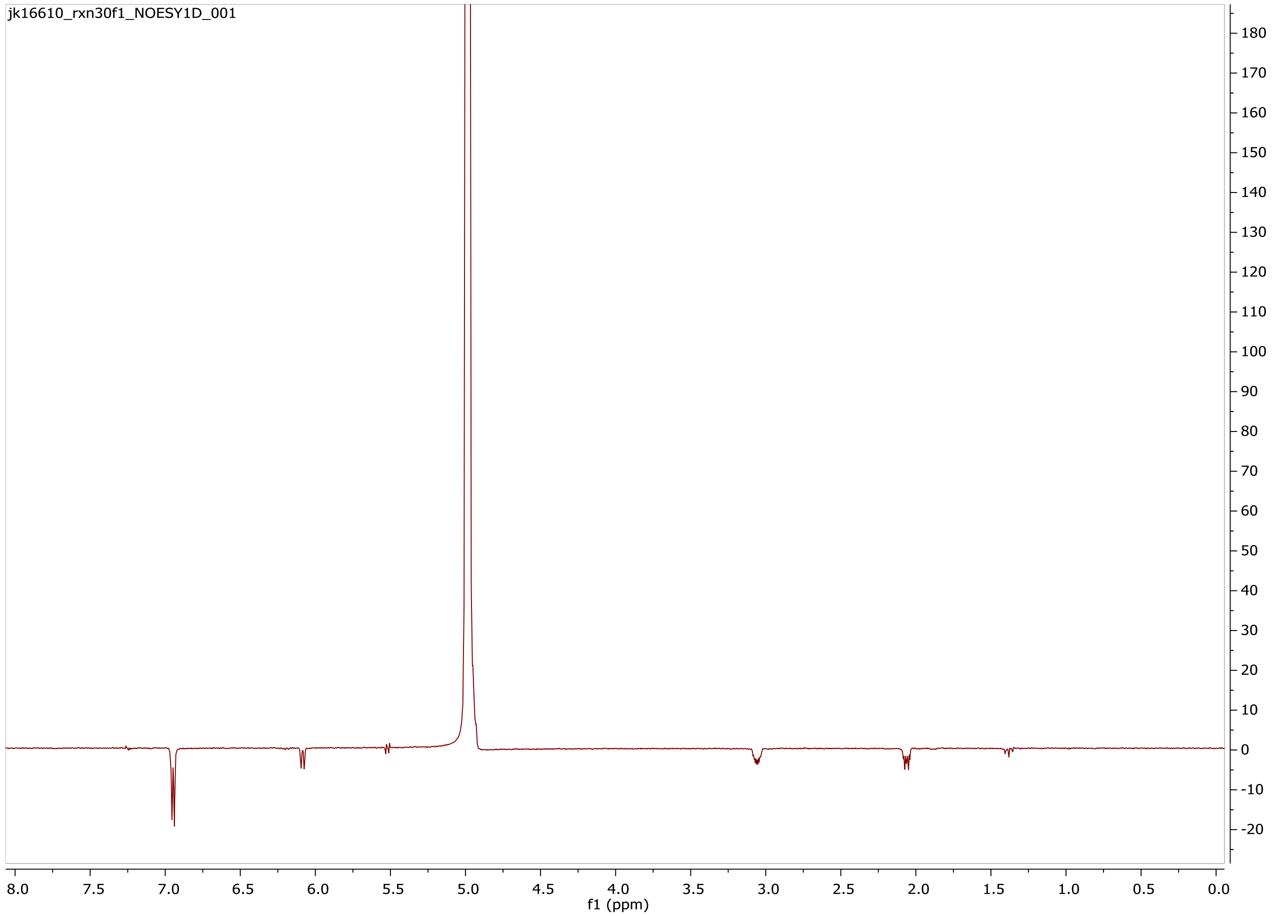

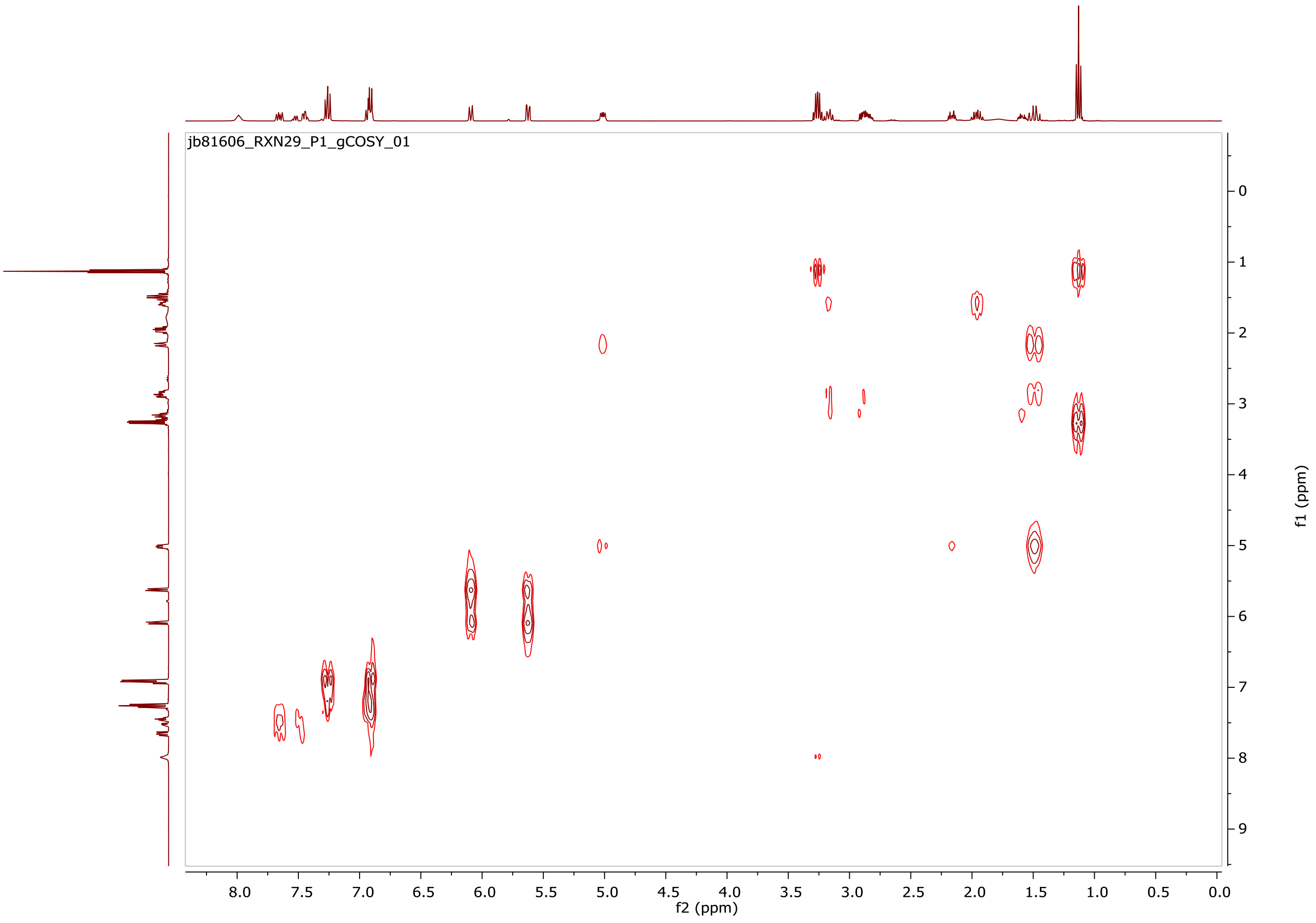

(±) -(3a*R*,5*R*,7a*R*)-*N*-ethyl-5-phenoxy-1,2,3,3a,4,5-hexahydro-7aH-indole-7a-carboxamide

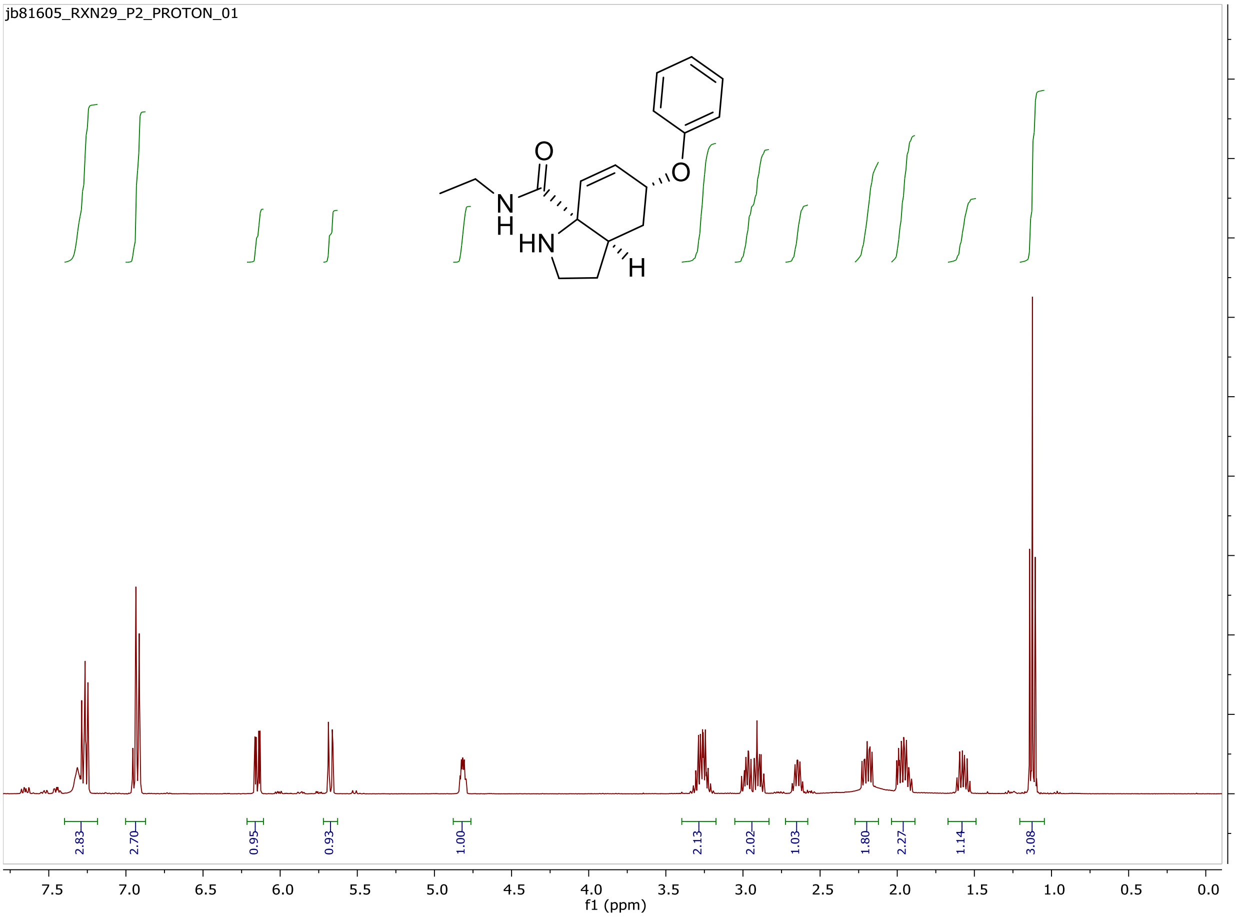

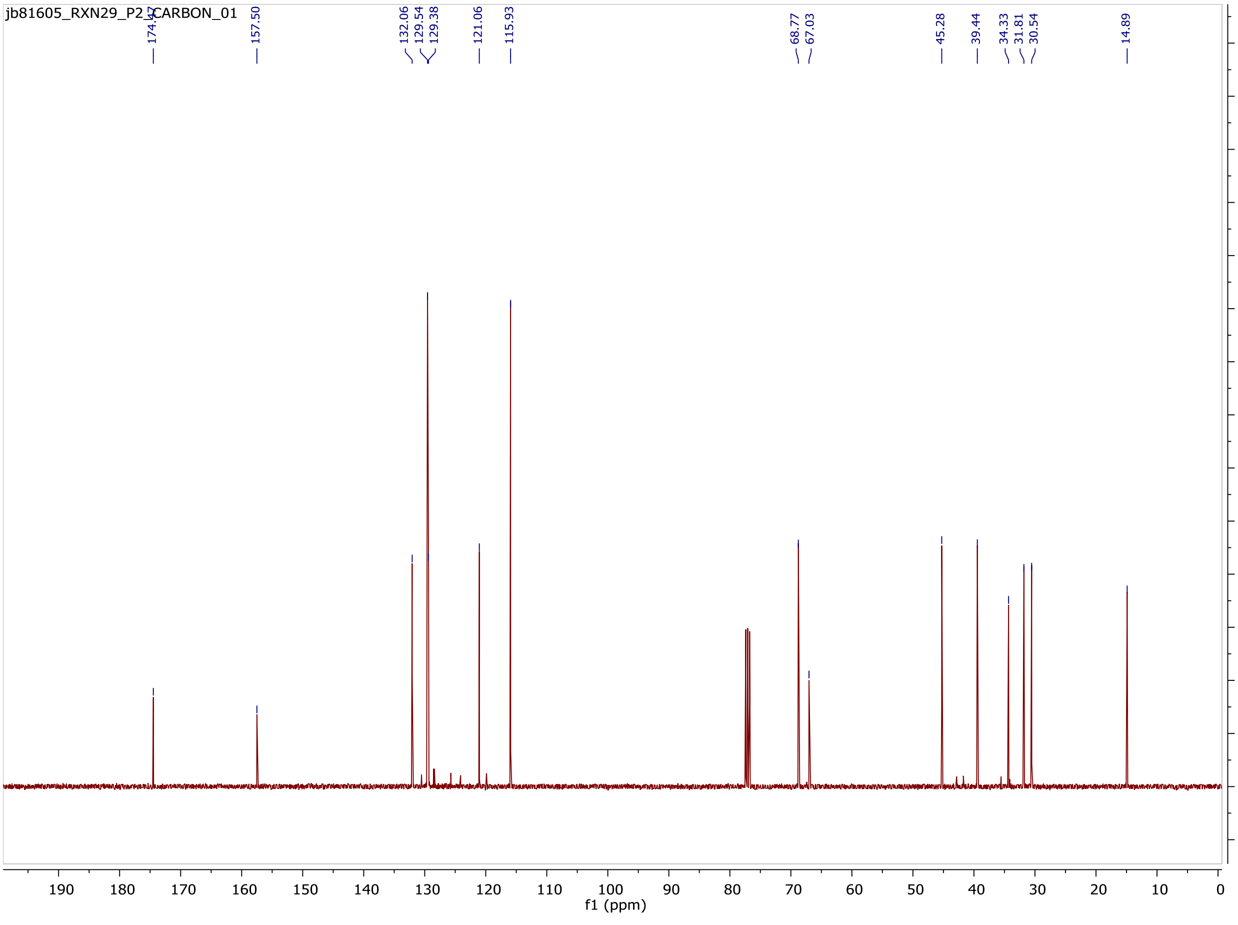

(±) -(3a*R*,5*R*,7a*R*)-*N*-ethyl-5-phenoxy-1,2,3,3a,4,5-hexahydro-7aH-indole-7a-carboxamide

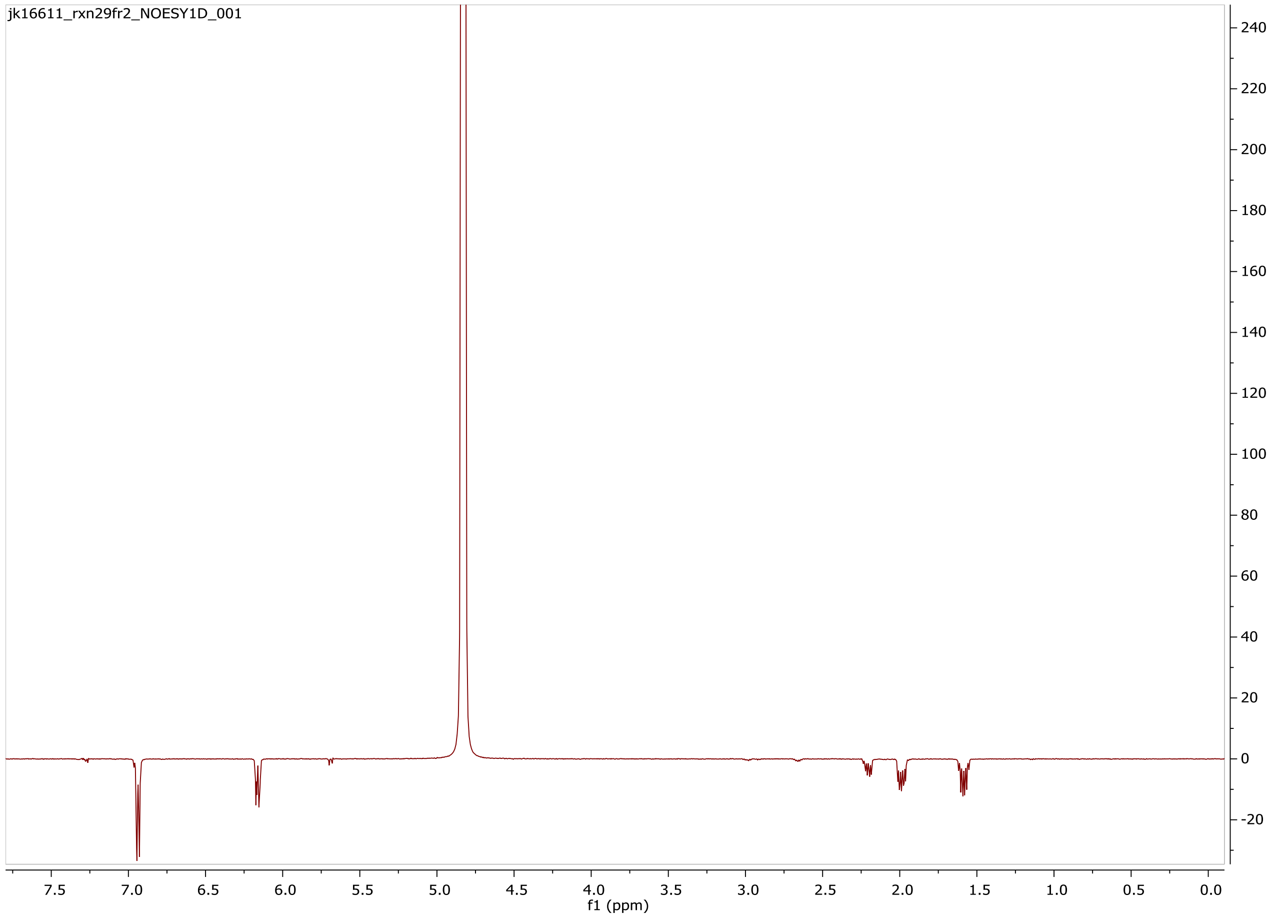

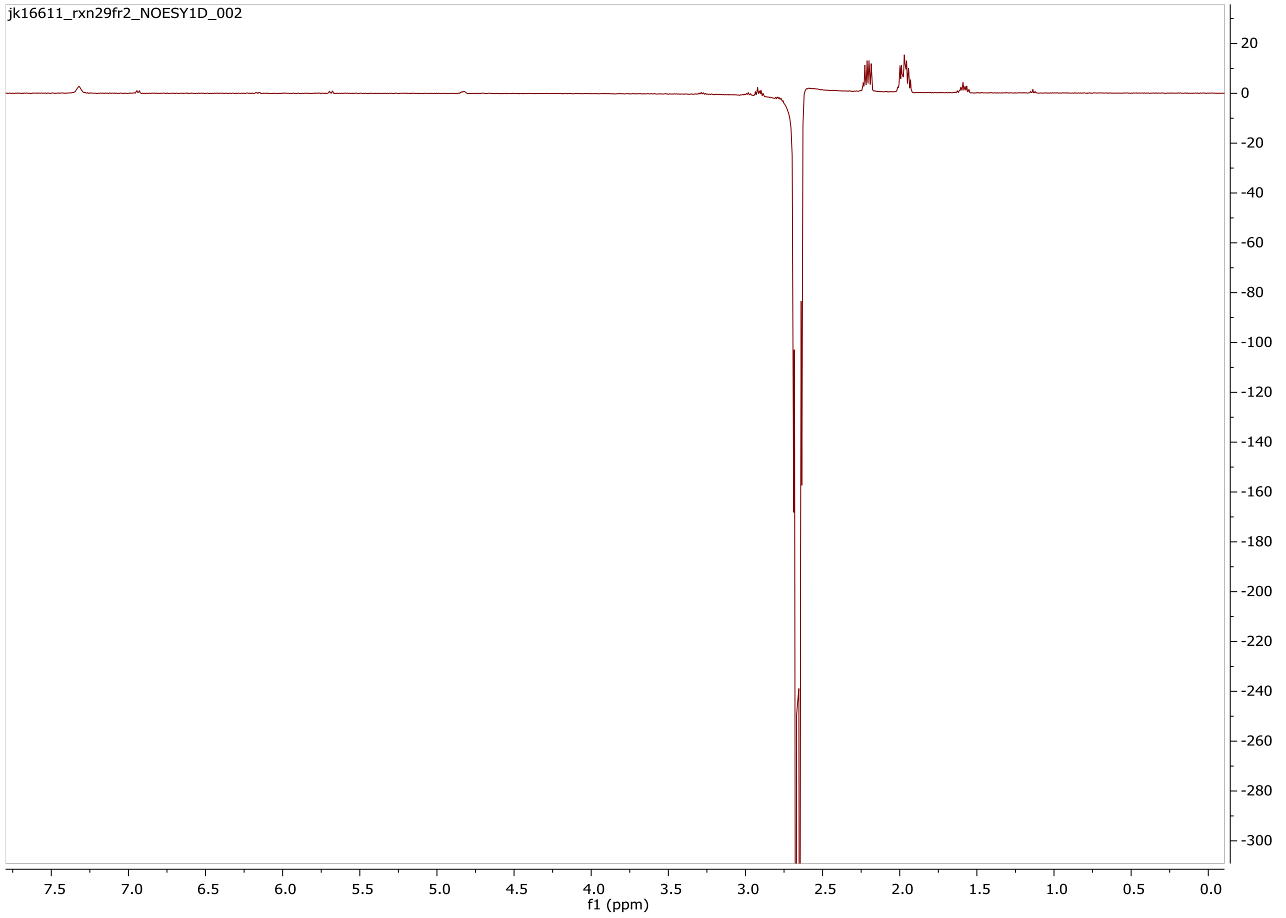

(±) -(3a*R*,5*R*,7a*R*)-*N*-ethyl-5-phenoxy-1,2,3,3a,4,5-hexahydro-7aH-indole-7a-carboxamide

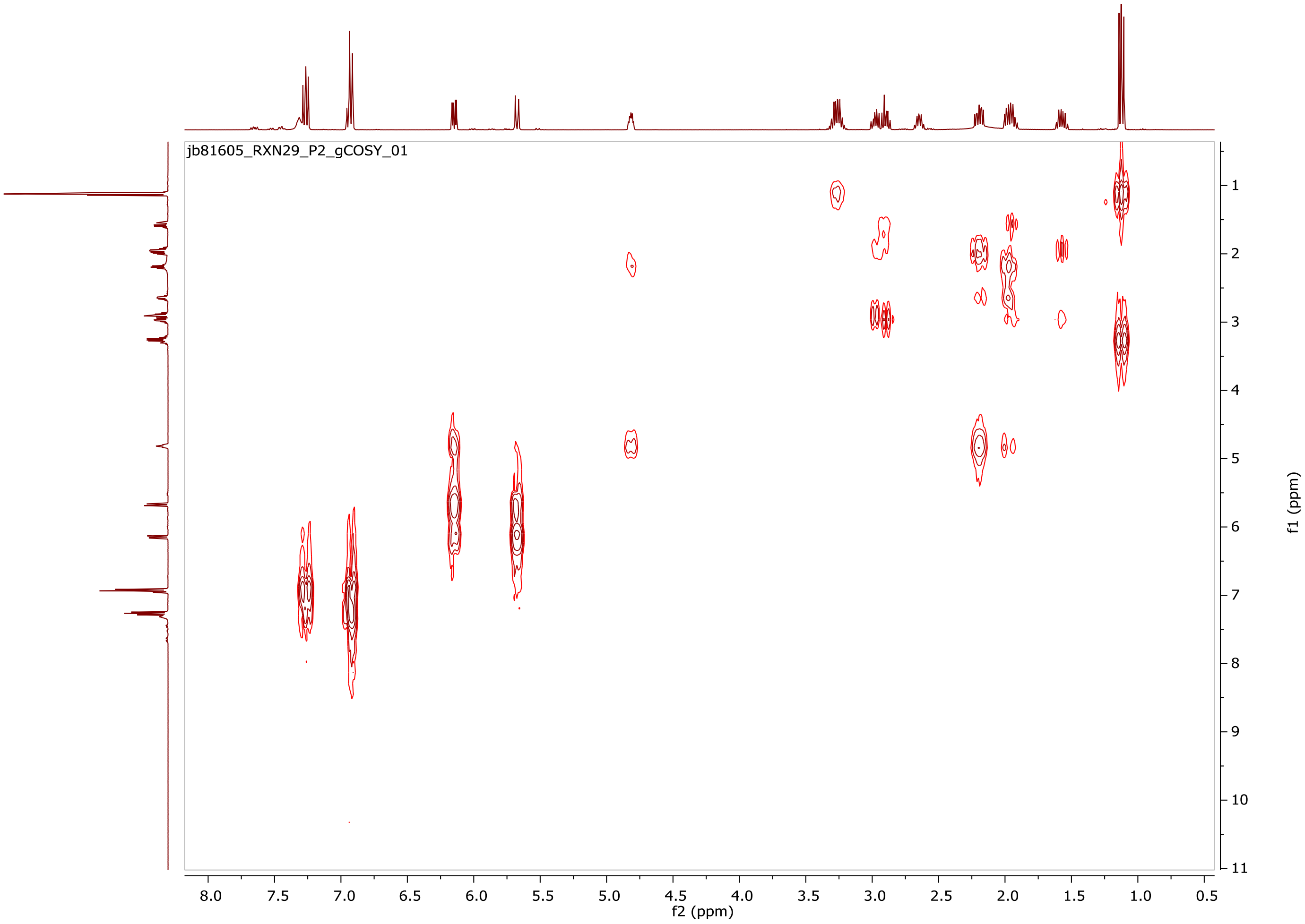

(±)-(3a*S*,5*S*,7a*R*)-5-(dicyanomethyl)-N-ethyl-1,2,3,3a,4,5-hexahydro-7aH-indole-7a-carboxamide

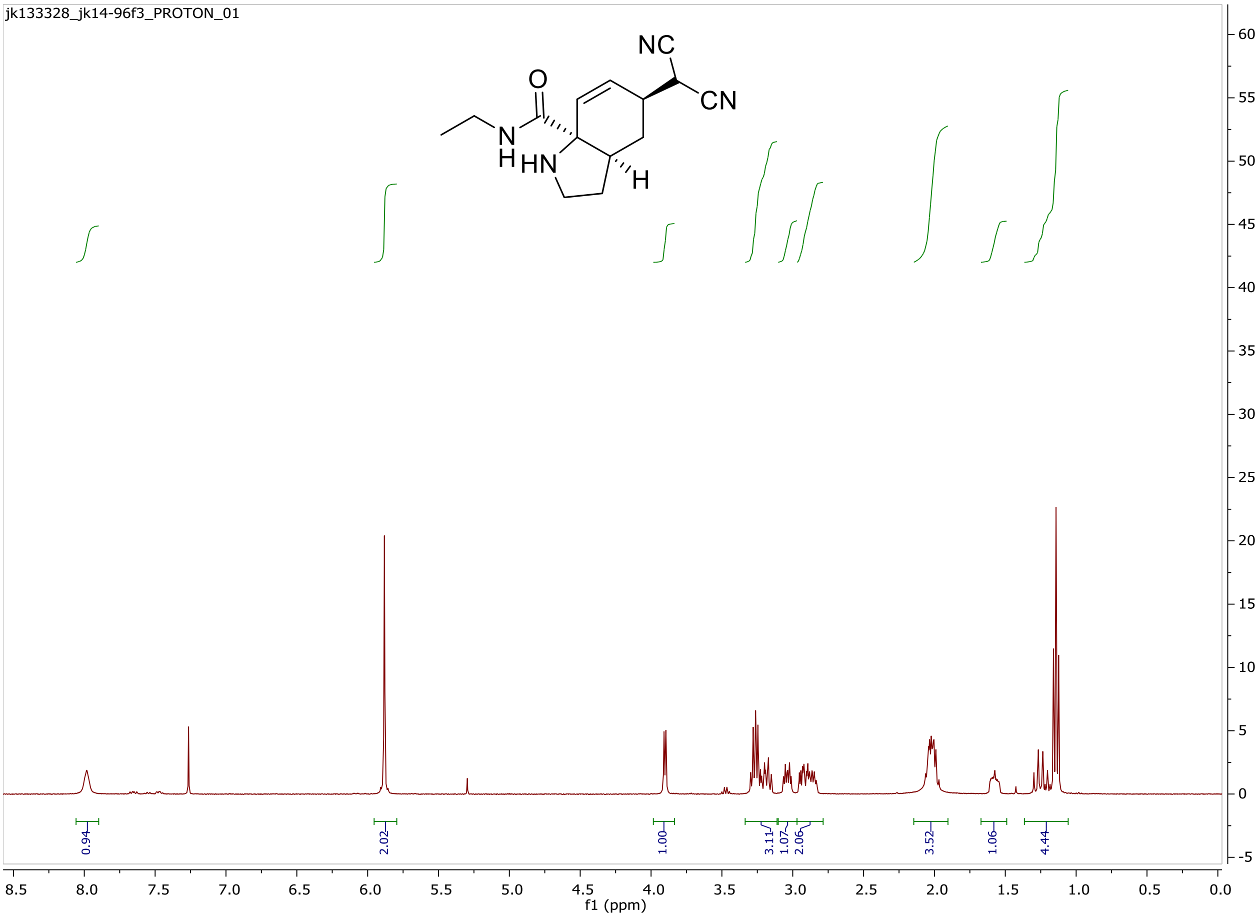

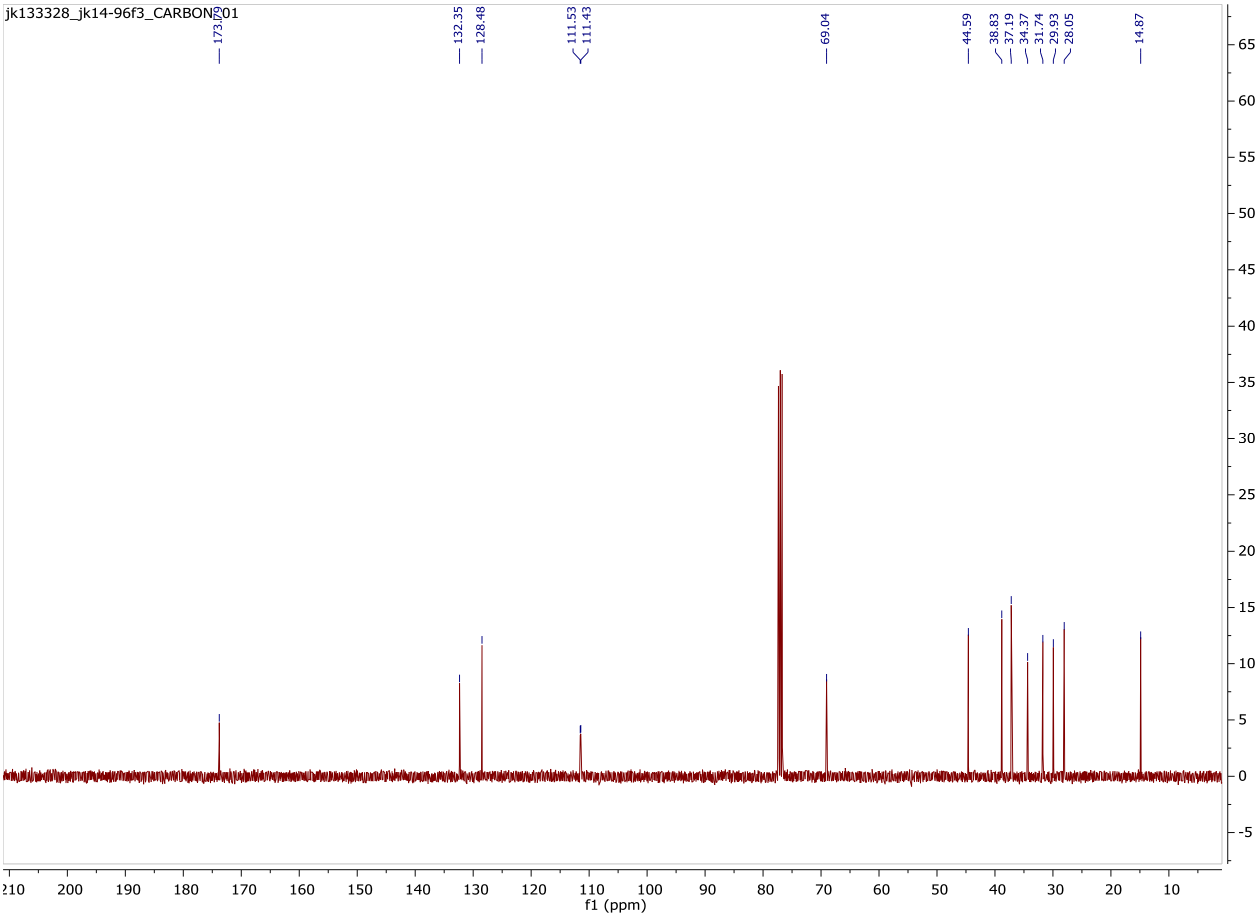

(±)-(3a*S*,5*S*,7a*R*)-5-(dicyanomethyl)-N-ethyl-1,2,3,3a,4,5-hexahydro-7aH-indole-7a-carboxamide

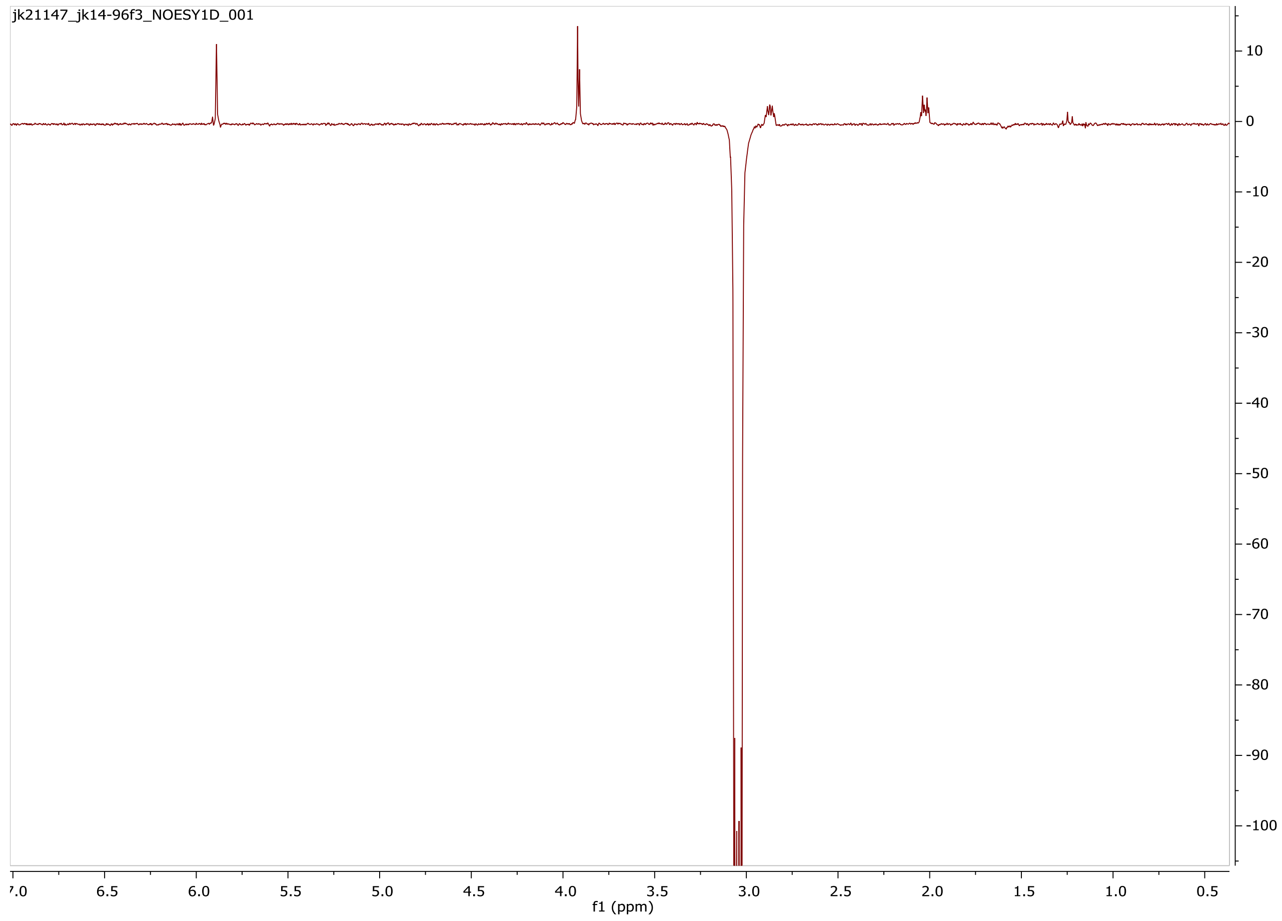

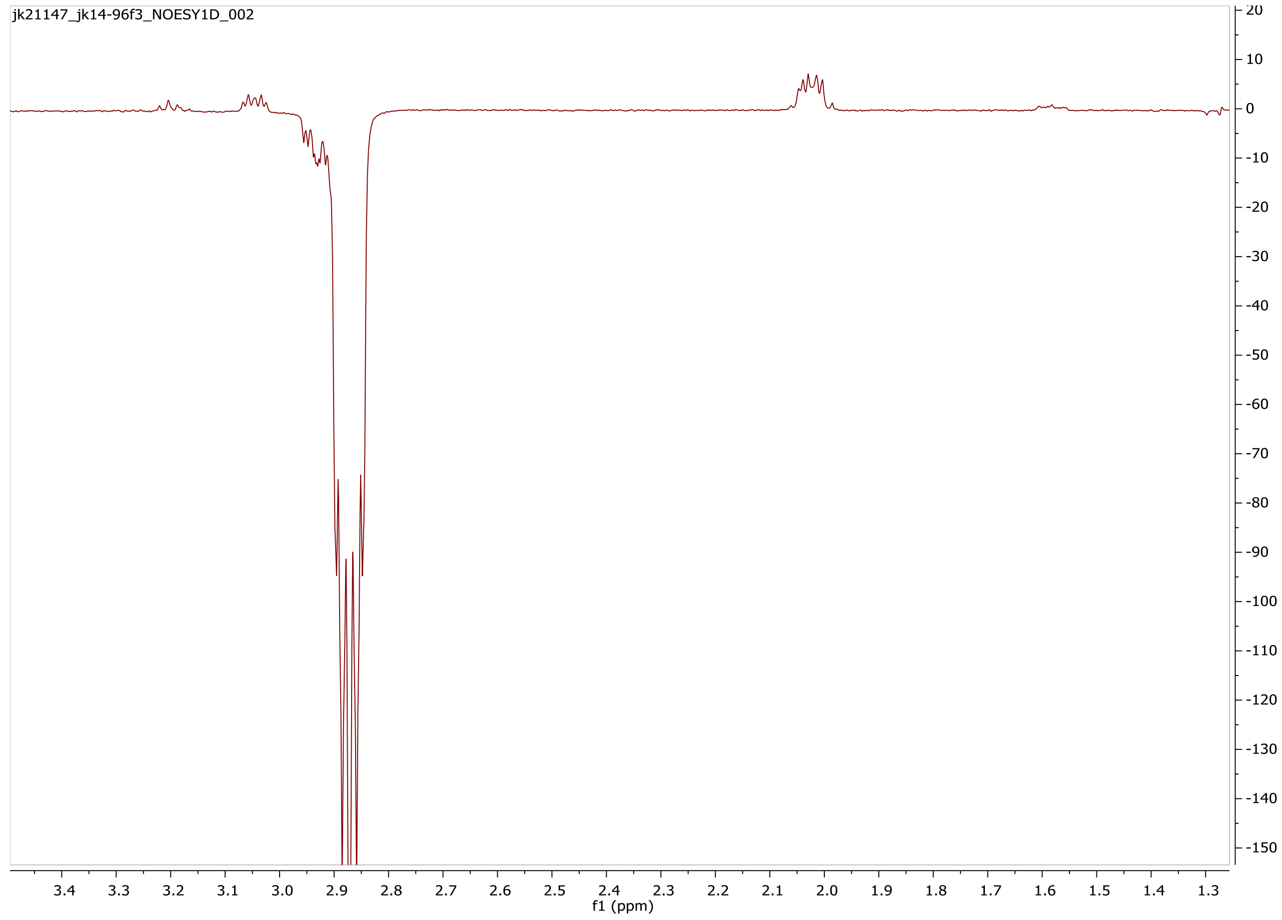

(±)-(3a*S*,5*R*,7a*R*)-5-(dicyanomethyl)-N-ethyl-1,2,3,3a,4,5-hexahydro-7aH-indole-7a-carboxamide

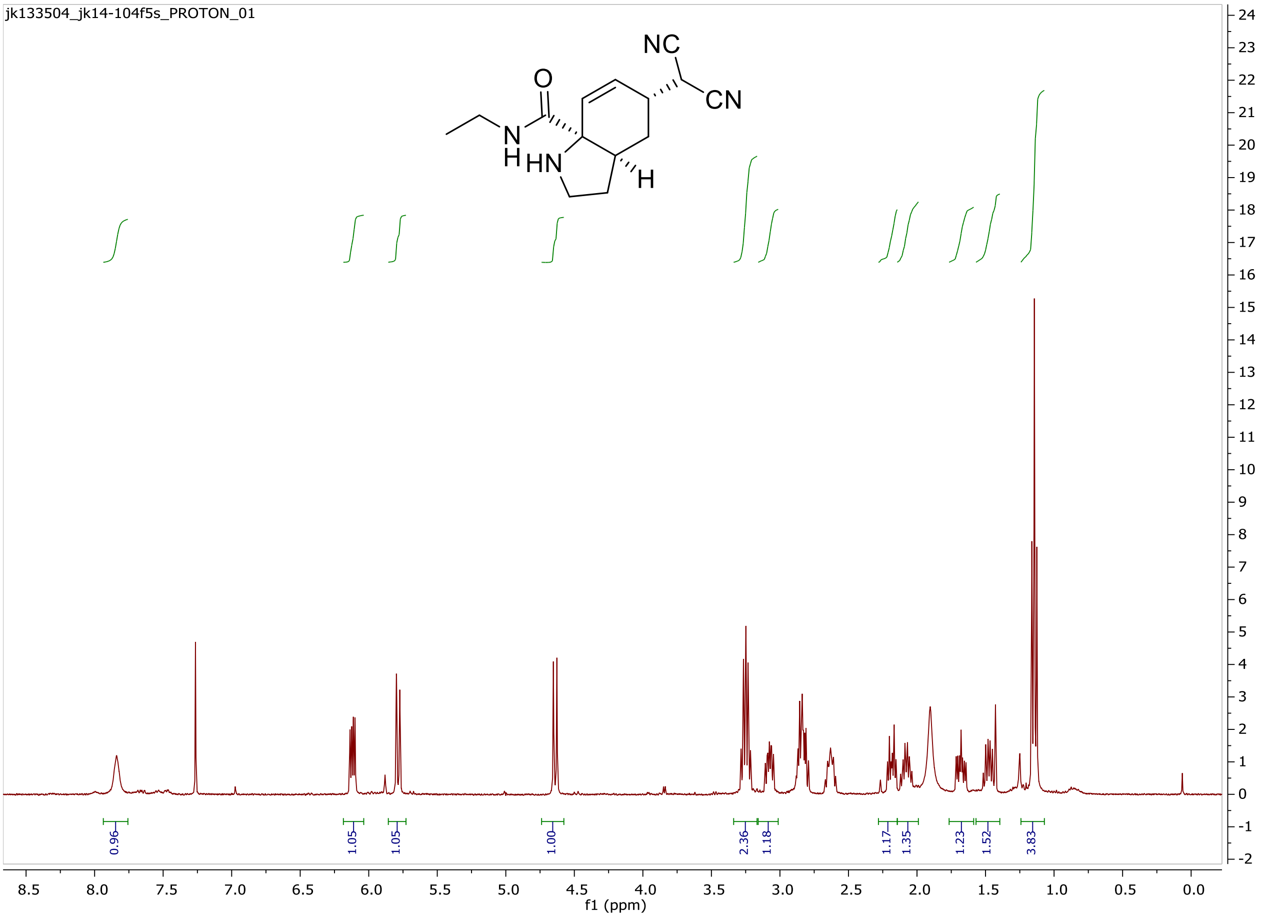

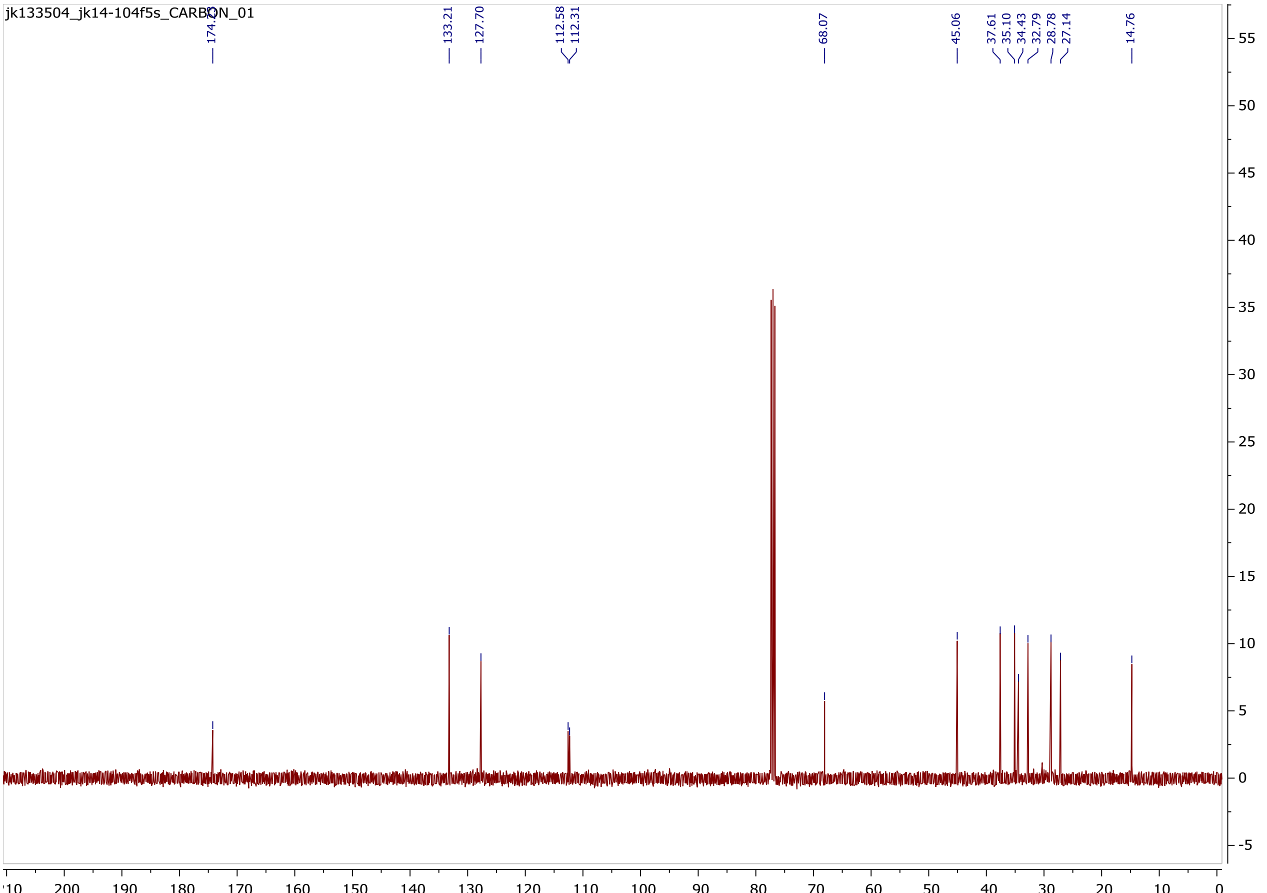

(±)-(3a*S*,5*S*,7a*R*)-5-(2,4-dioxopentan-3-yl)-N-ethyl-1,2,3,3a,4,5-hexahydro-7aH-indole-7a-carboxamide

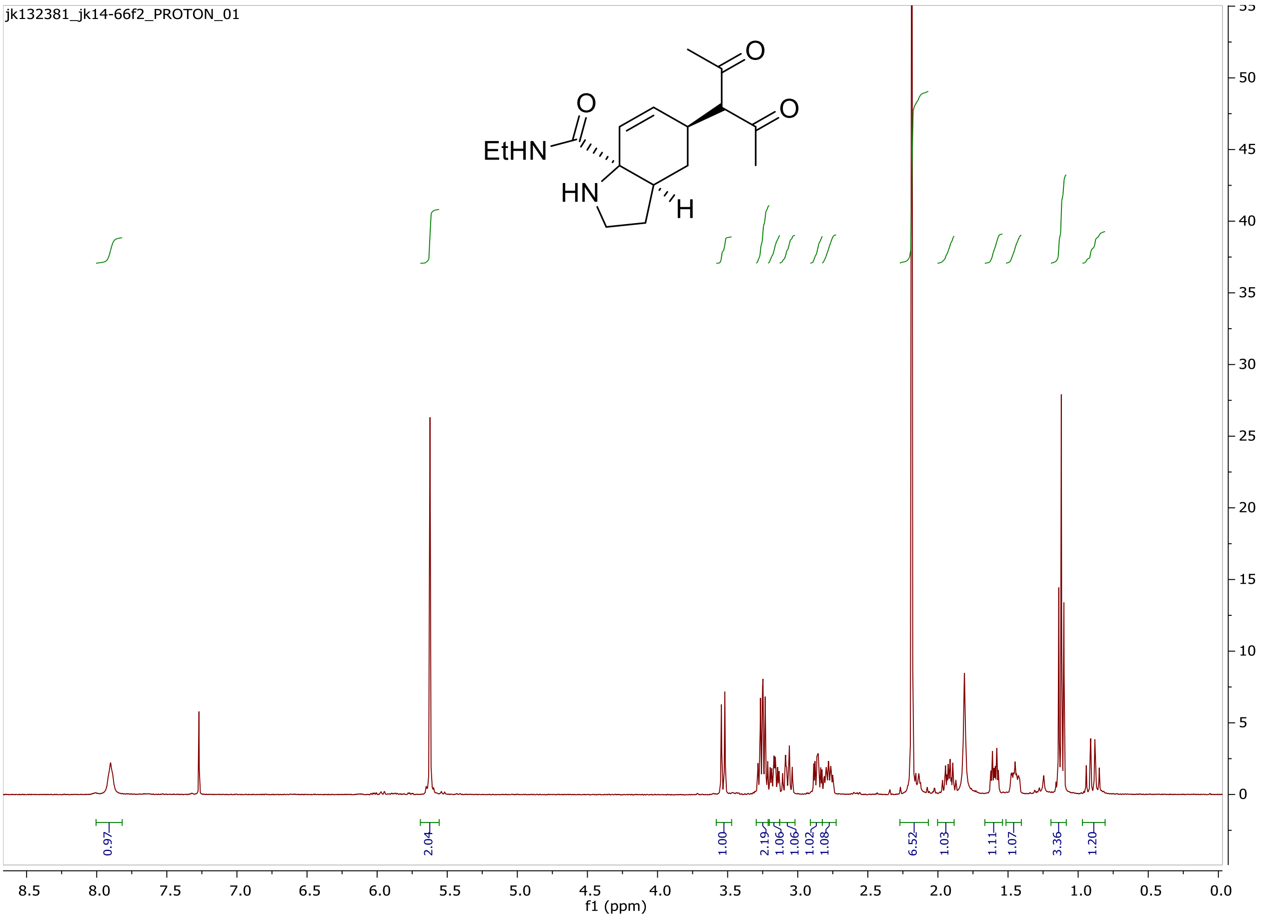

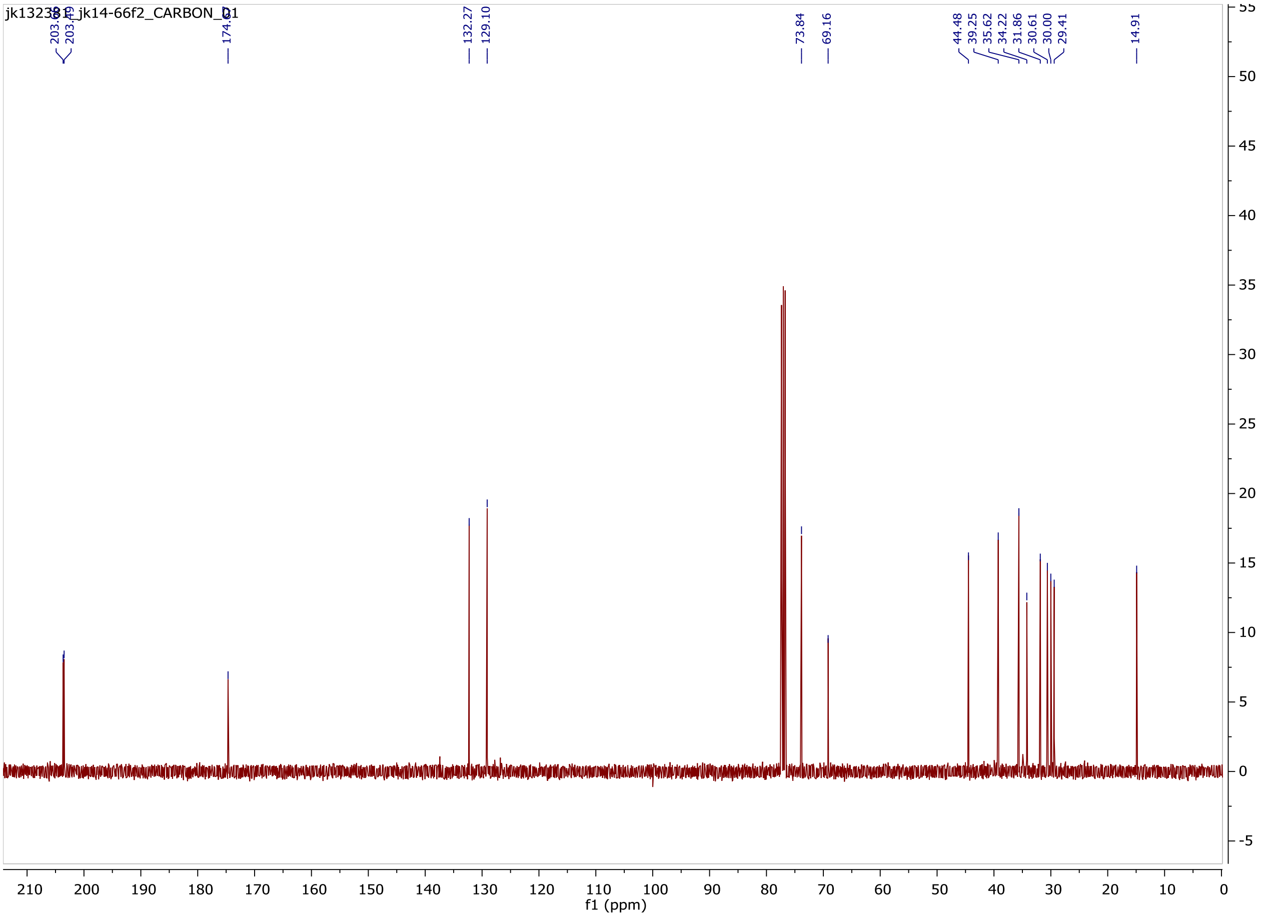

(±)-(3a*S*,5*S*,7a*R*)-5-(2,4-dioxopentan-3-yl)-N-ethyl-1,2,3,3a,4,5-hexahydro-7aH-indole-7a-carboxamide

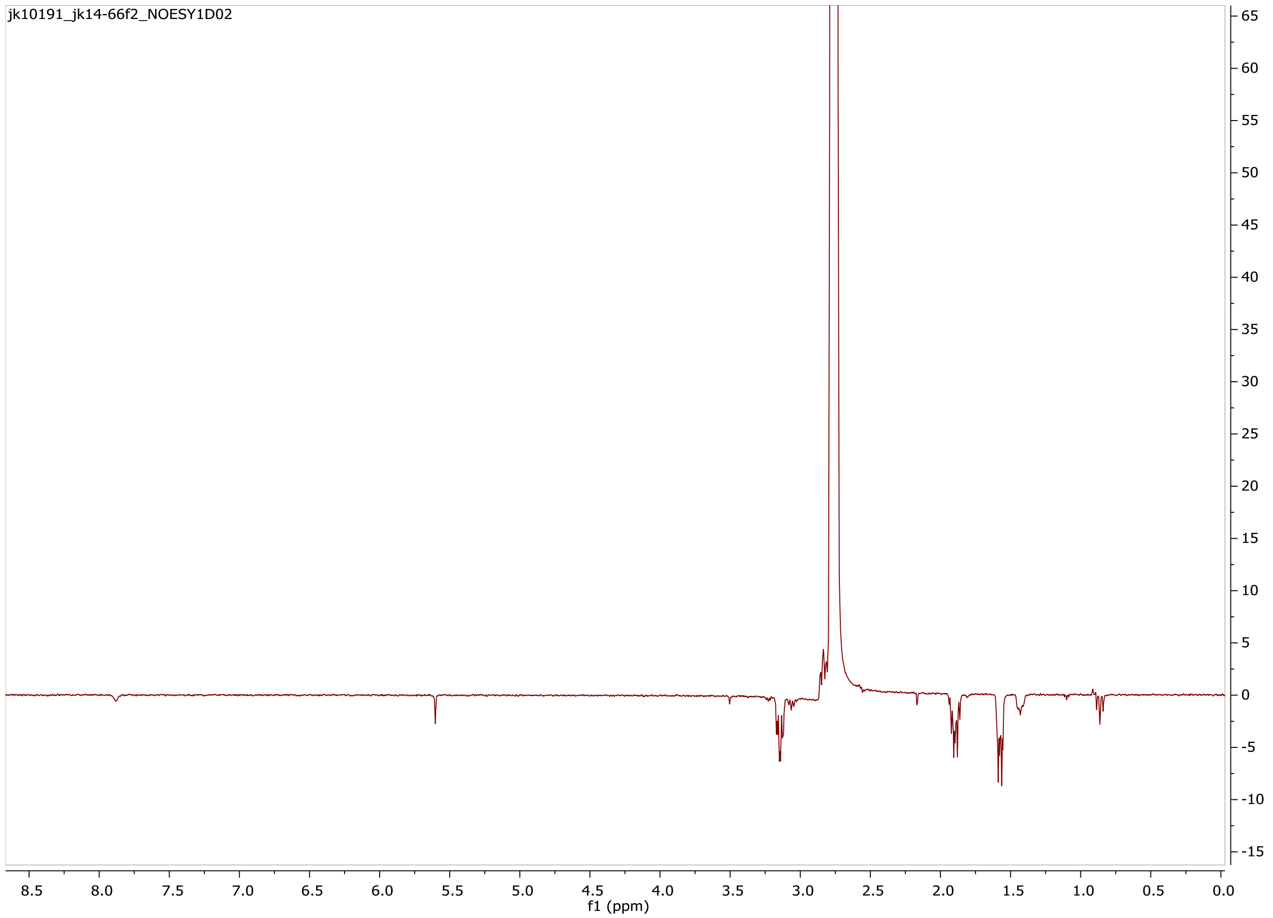

(±)-(3a*S*,5*R*,7a*R*)-5-(2,4-dioxopentan-3-yl)-N-ethyl-1,2,3,3a,4,5-hexahydro-7aH-indole-7a-carboxamide

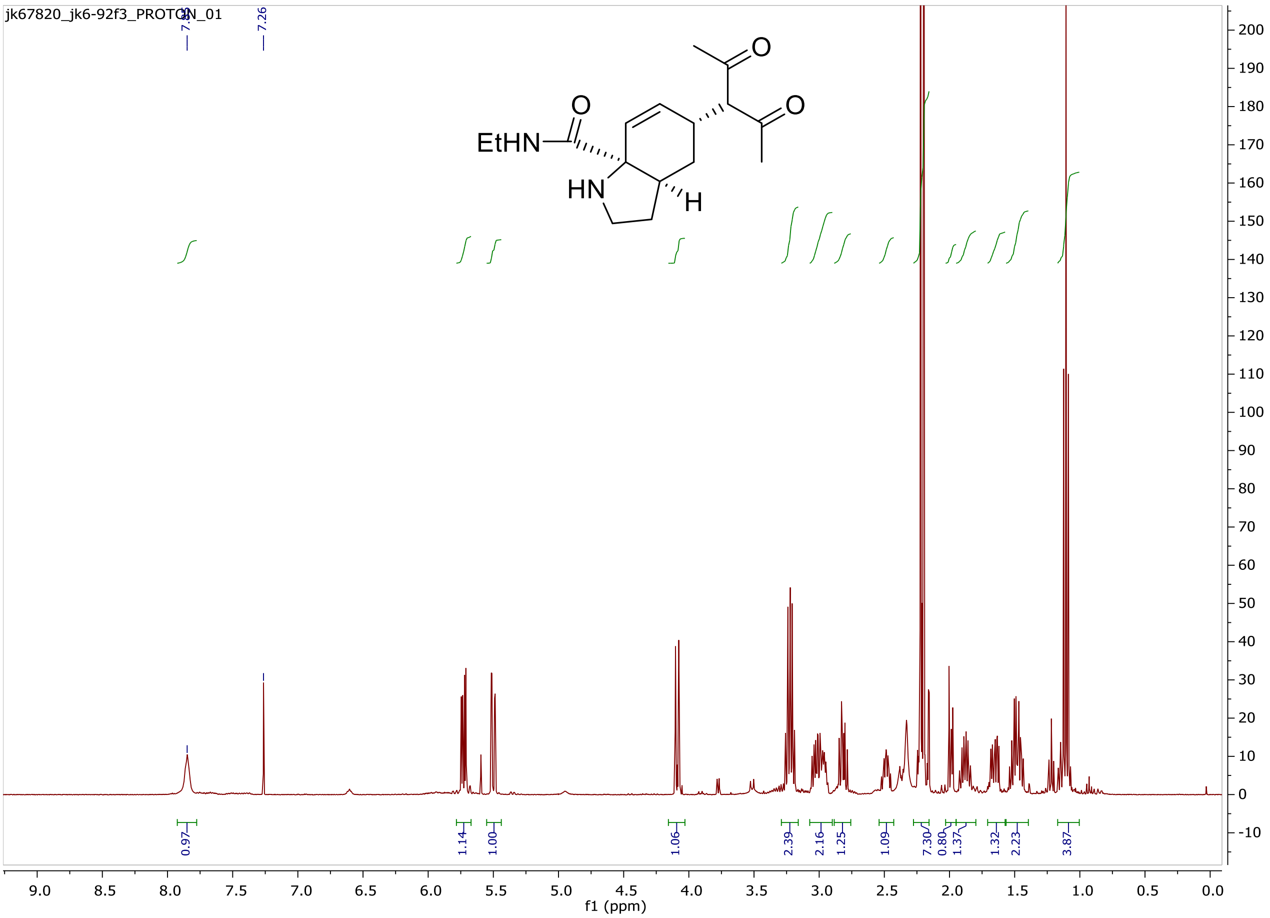

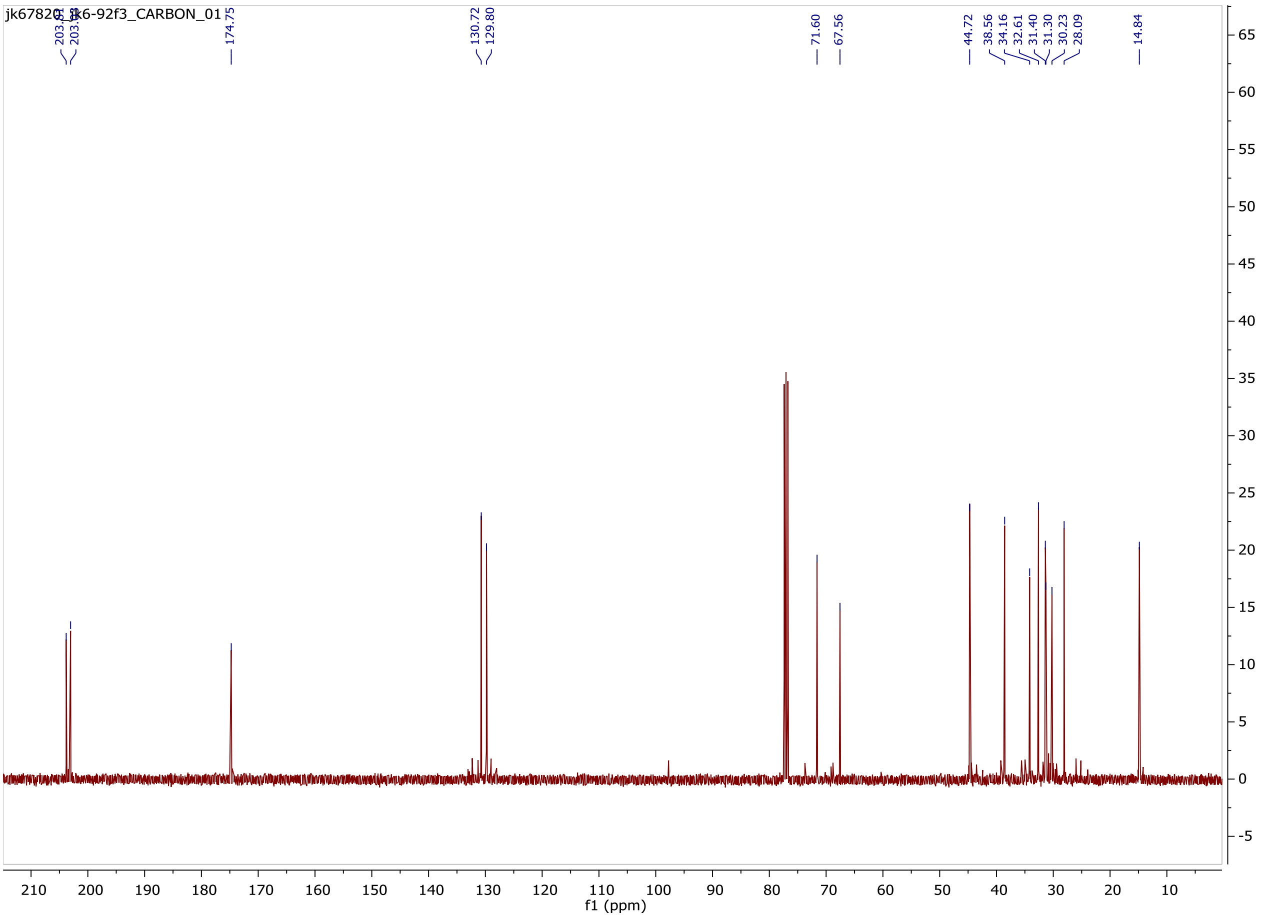

(±)-(3a*S*,5*S*,7a*R*)-5-(1,3-dioxo-2,3-dihydro-1H-inden-2-yl)-N-ethyl-1,2,3,3a,4,5-hexahydro-7aH-indole-7a-carboxamide

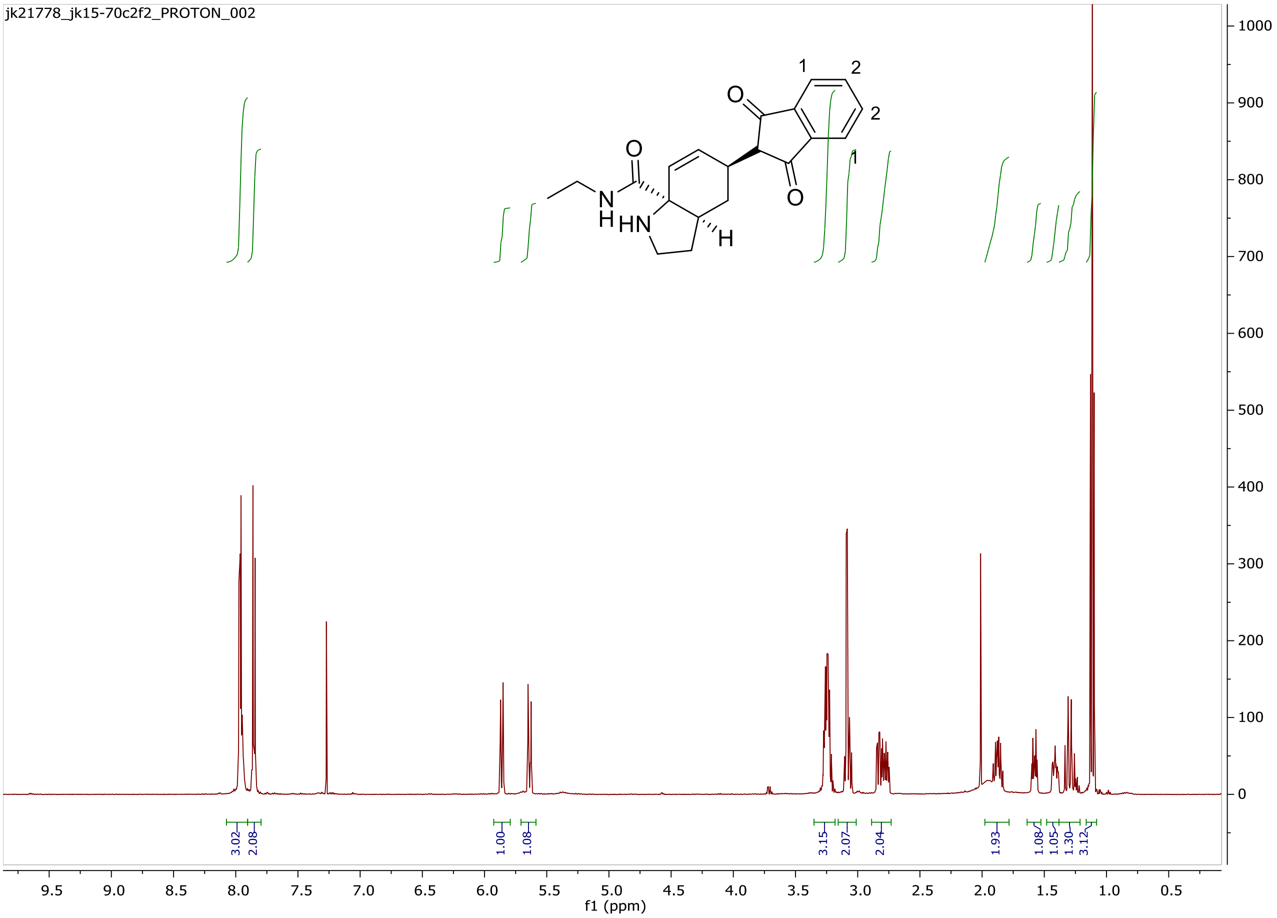

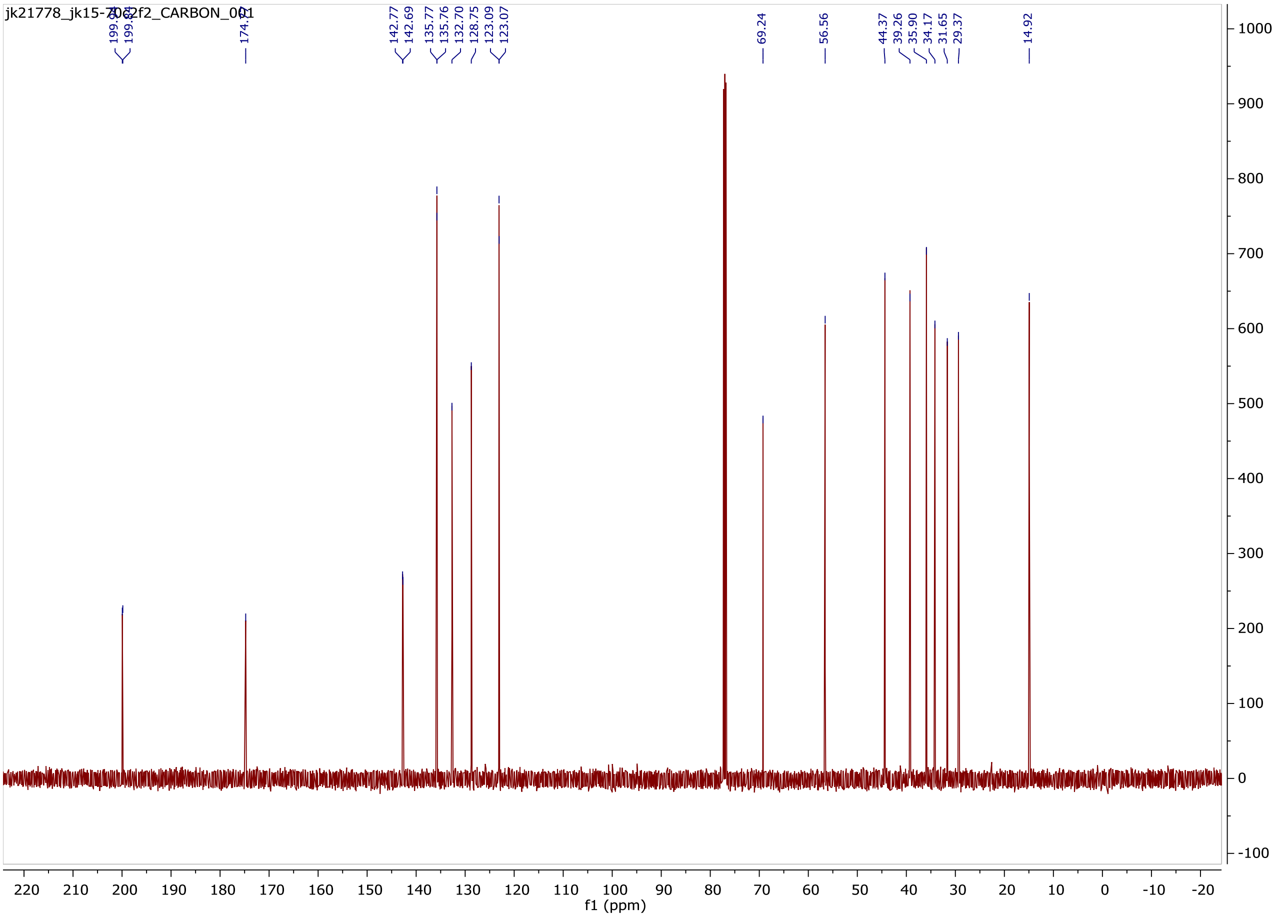

(±)-(3a*S*,5*S*,7a*R*)-5-(1,3-dioxo-2,3-dihydro-1H-inden-2-yl)-N-ethyl-1,2,3,3a,4,5-hexahydro-7aH-indole-7a-carboxamide

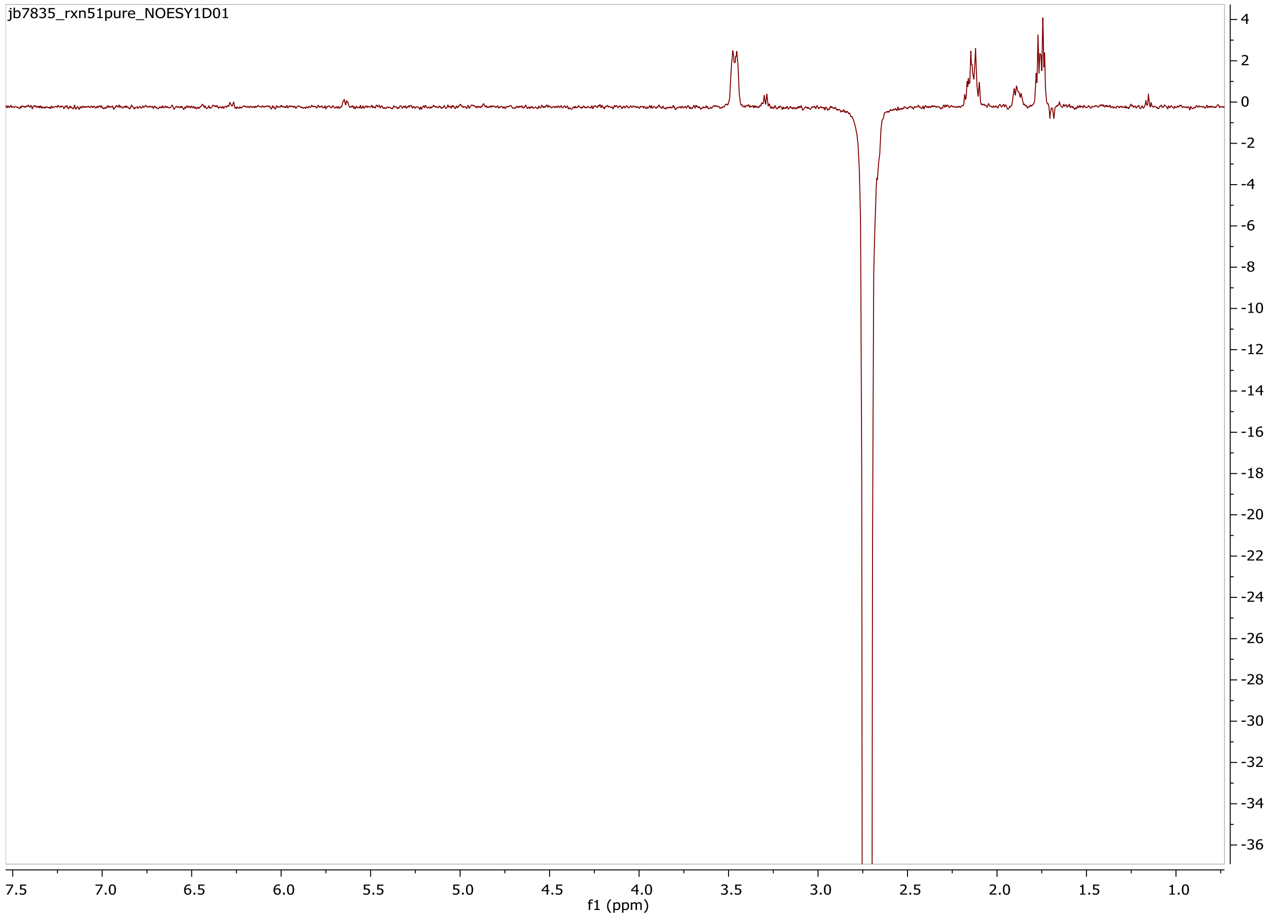

(±)-(3a*S*,5*S*,7a*R*)-5-(4,4-dimethyl-2,6-dioxocyclohexyl)-N-ethyl-1,2,3,3a,4,5-hexahydro-7aH-indole-7a-carboxamide

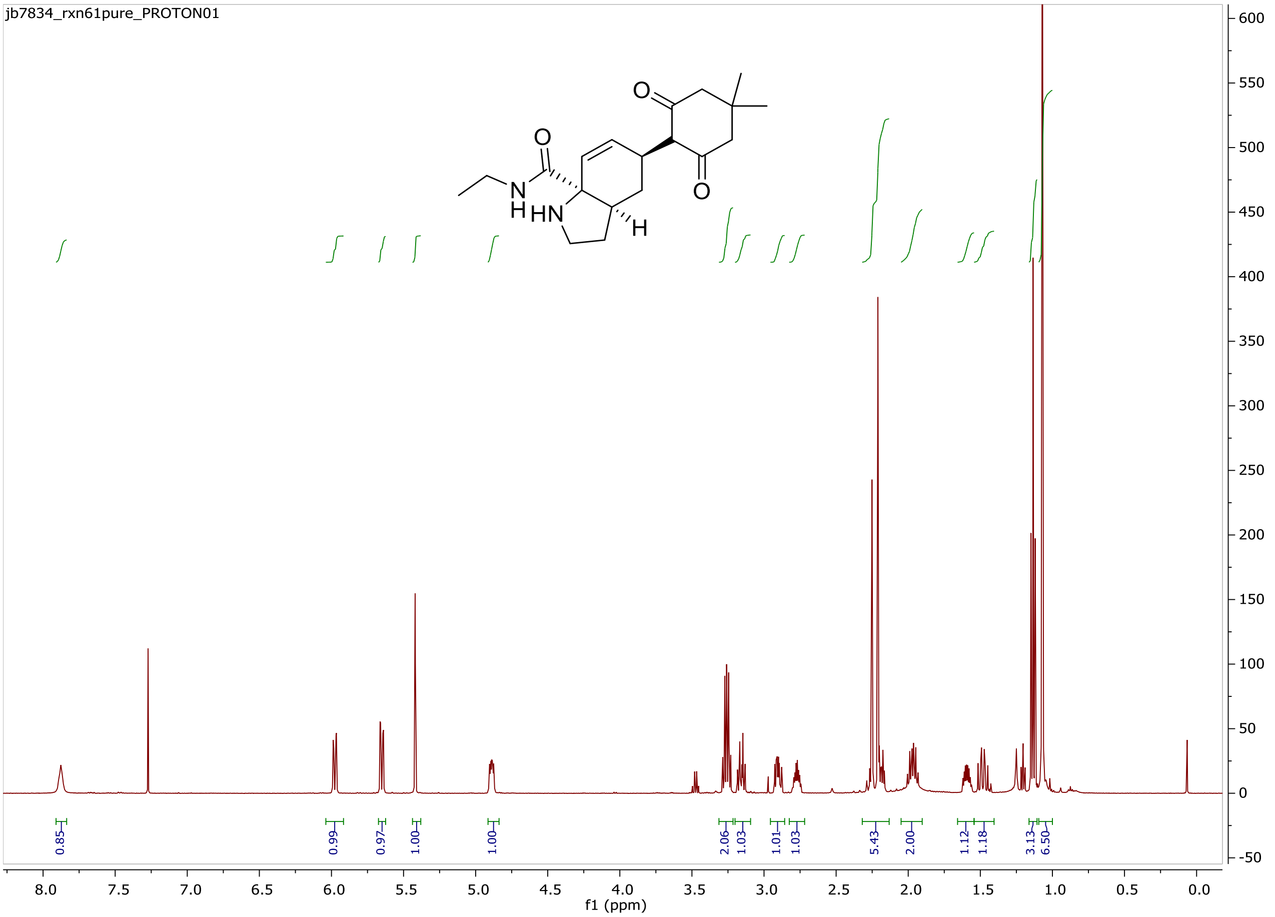

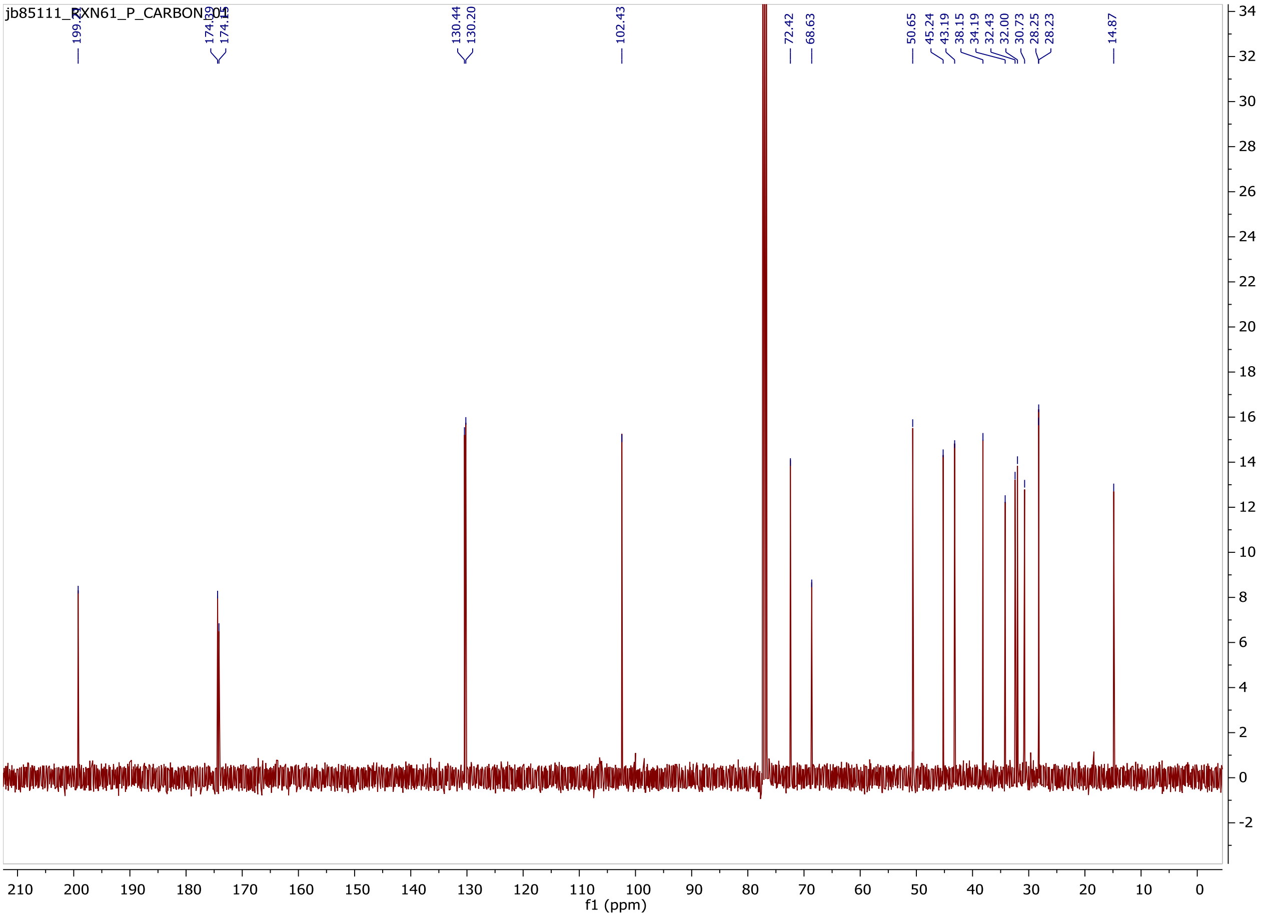

(±)-(3a*S*,5*S*,7a*R*)-5-(4,4-dimethyl-2,6-dioxocyclohexyl)-N-ethyl-1,2,3,3a,4,5-hexahydro-7aH-indole-7a-carboxamide

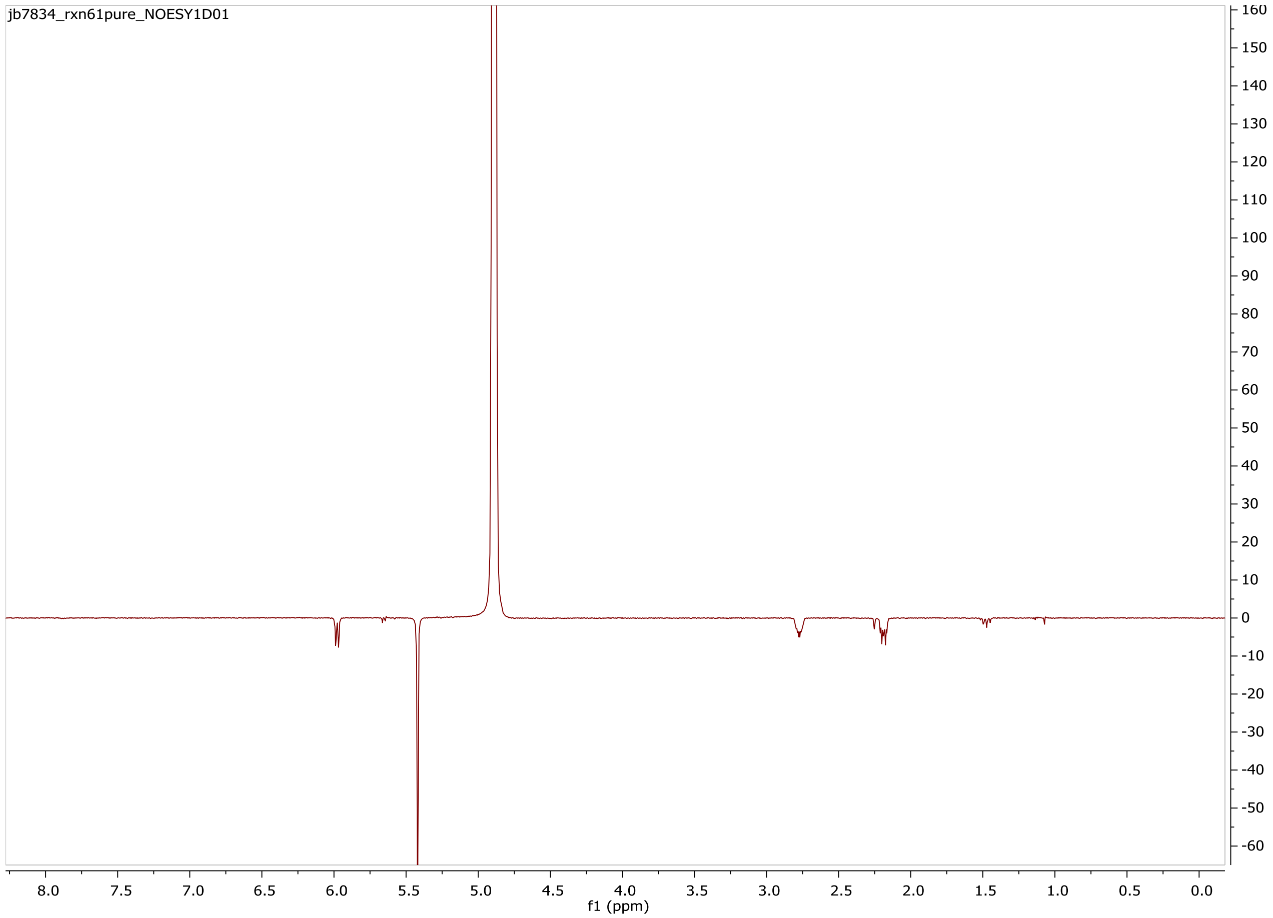

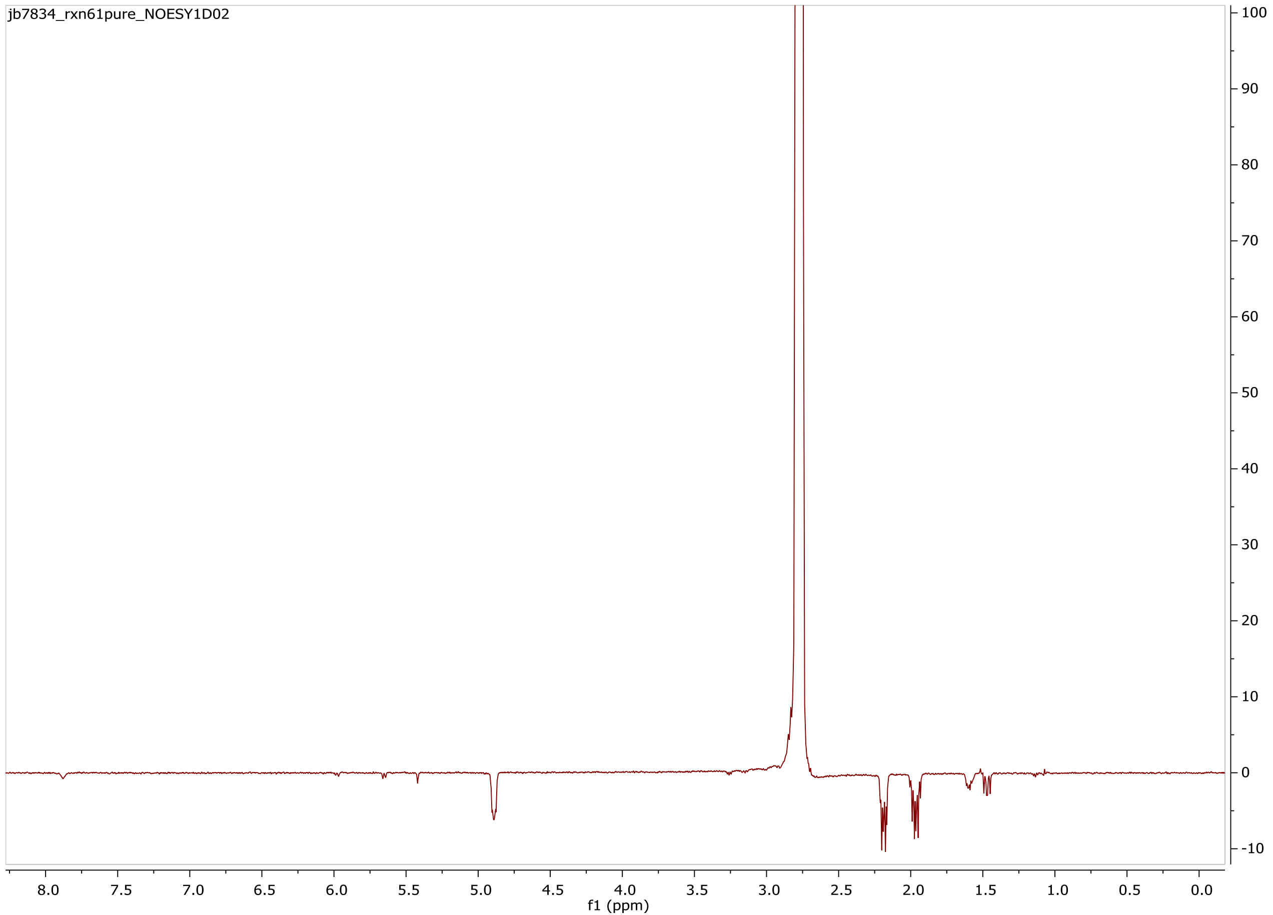

(±)-2-((3a*S*,5*S*,7a*R*)-7a-acetyl-2,3,3a,4,5,7a-hexahydro-1H-indol-5-yl)-5,5-dimethylcyclohexane-1,3-dione

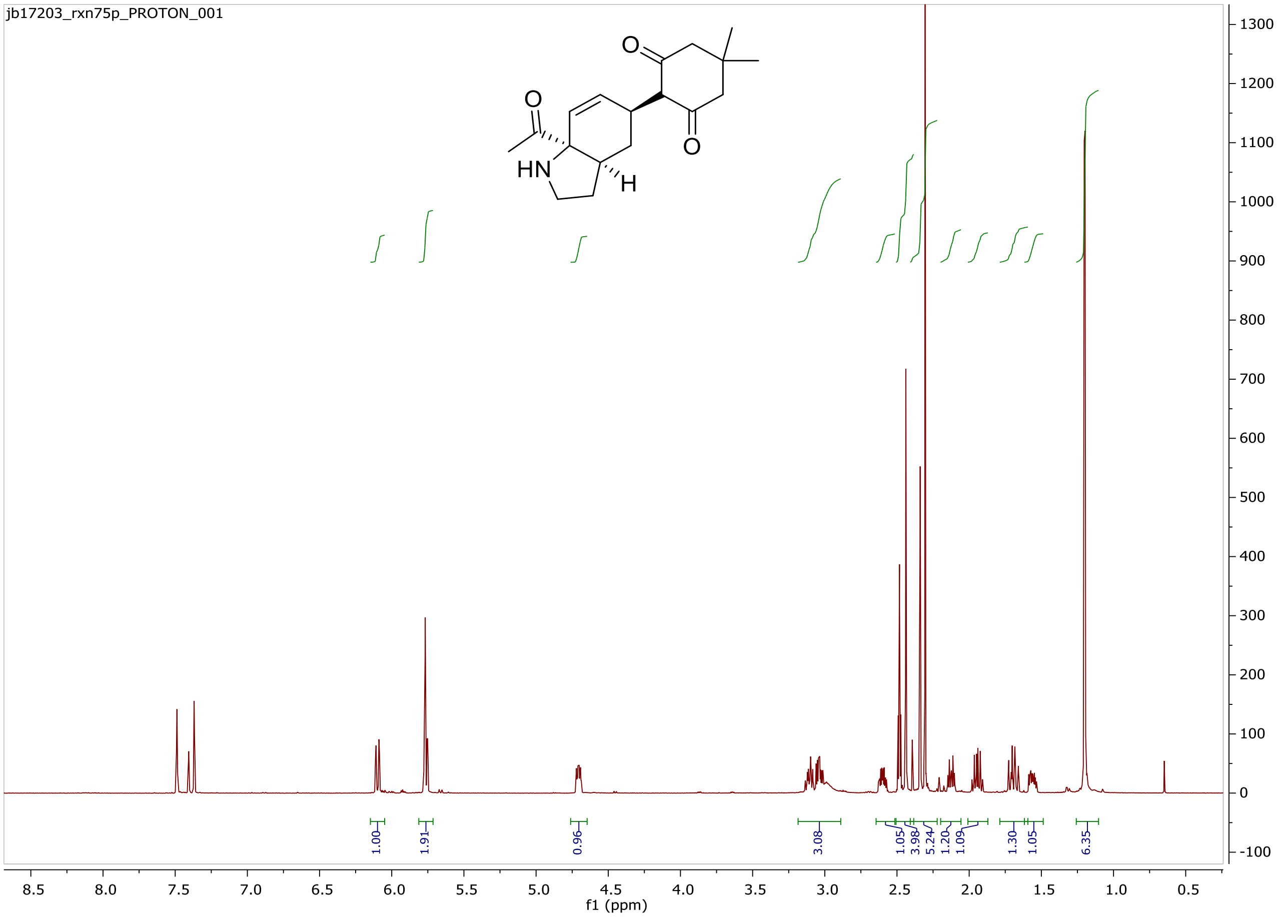

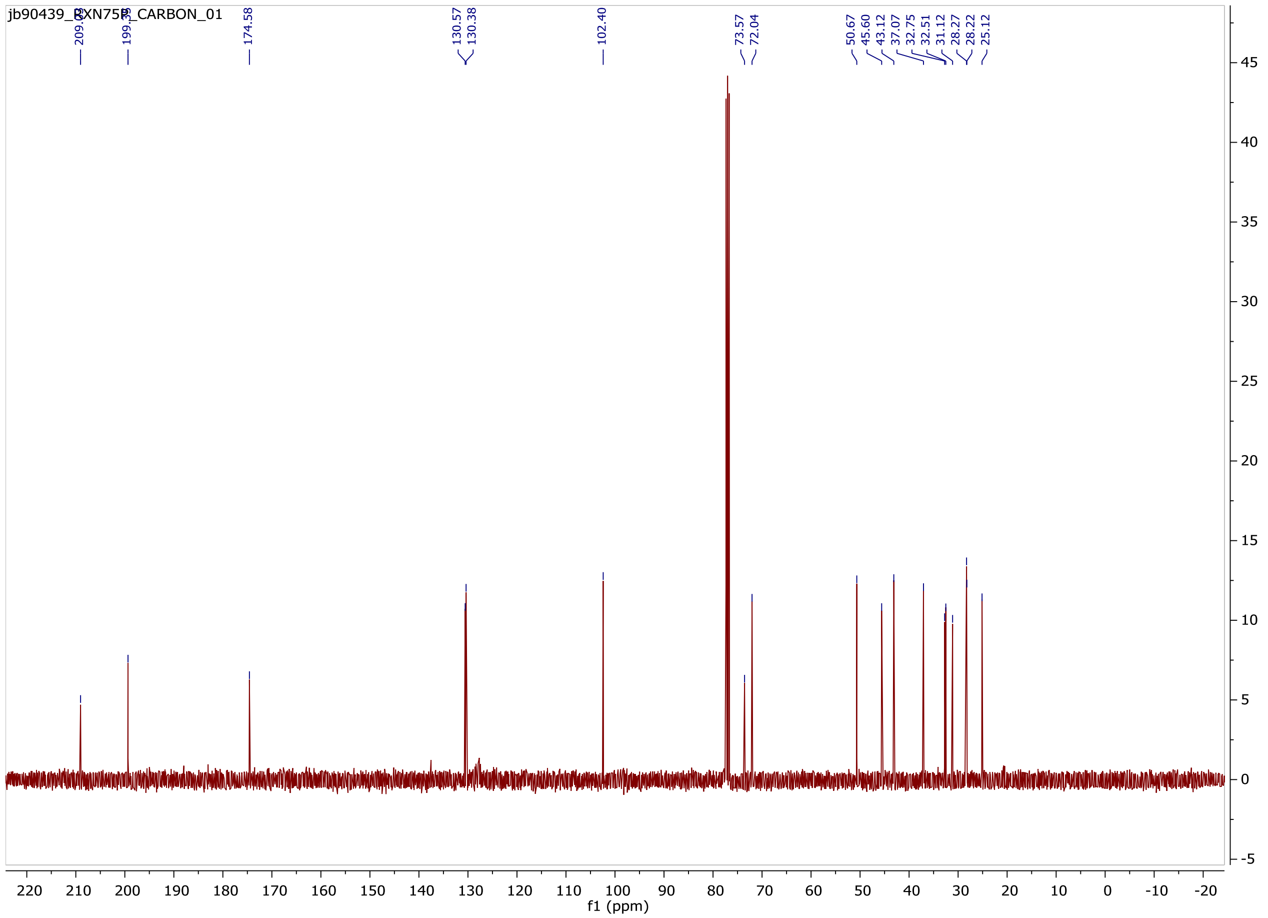

(±)-2-((3a*S*,5*S*,7a*R*)-7a-acetyl-2,3,3a,4,5,7a-hexahydro-1H-indol-5-yl)-5,5-dimethylcyclohexane-1,3-dione

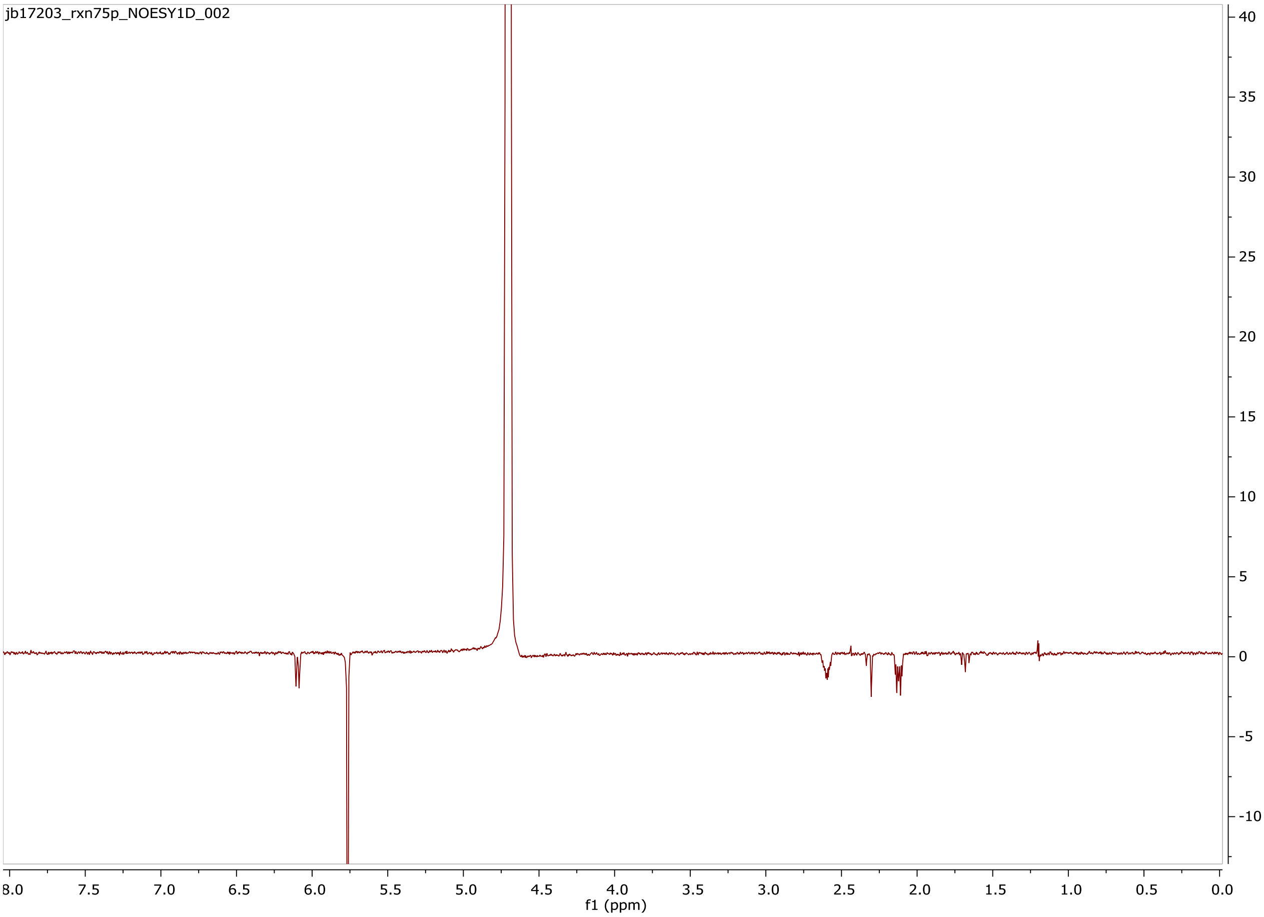

(±)-2-((3a*S*,5*S*,7a*R*)-7a-acetyl-2,3,3a,4,5,7a-hexahydro-1H-indol-5-yl)-1H-indene-1,3(2H)-dione

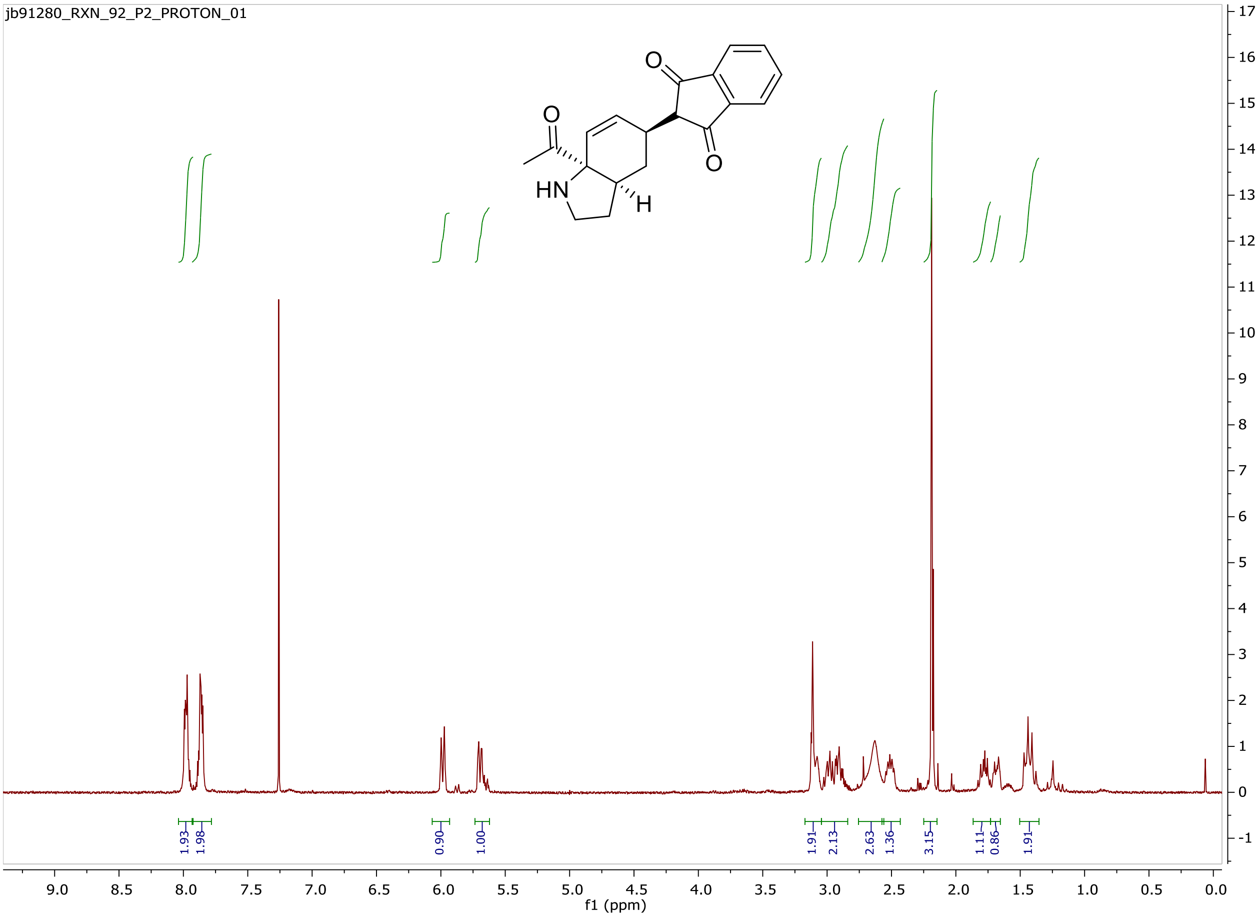

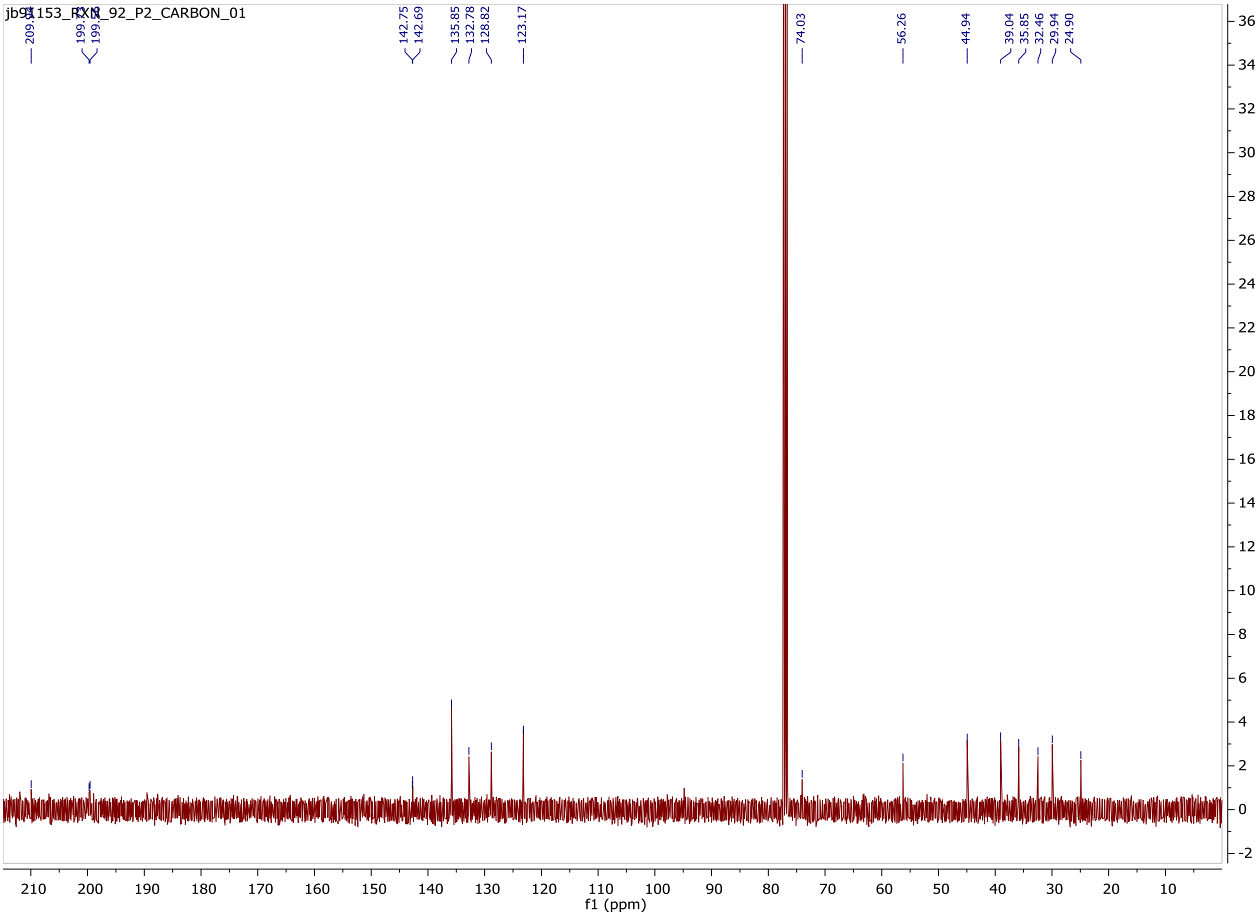

(±)-2-((3a*S*,5*S*,7a*R*)-7a-acetyl-2,3,3a,4,5,7a-hexahydro-1H-indol-5-yl)-1H-indene-1,3(2H)-dione

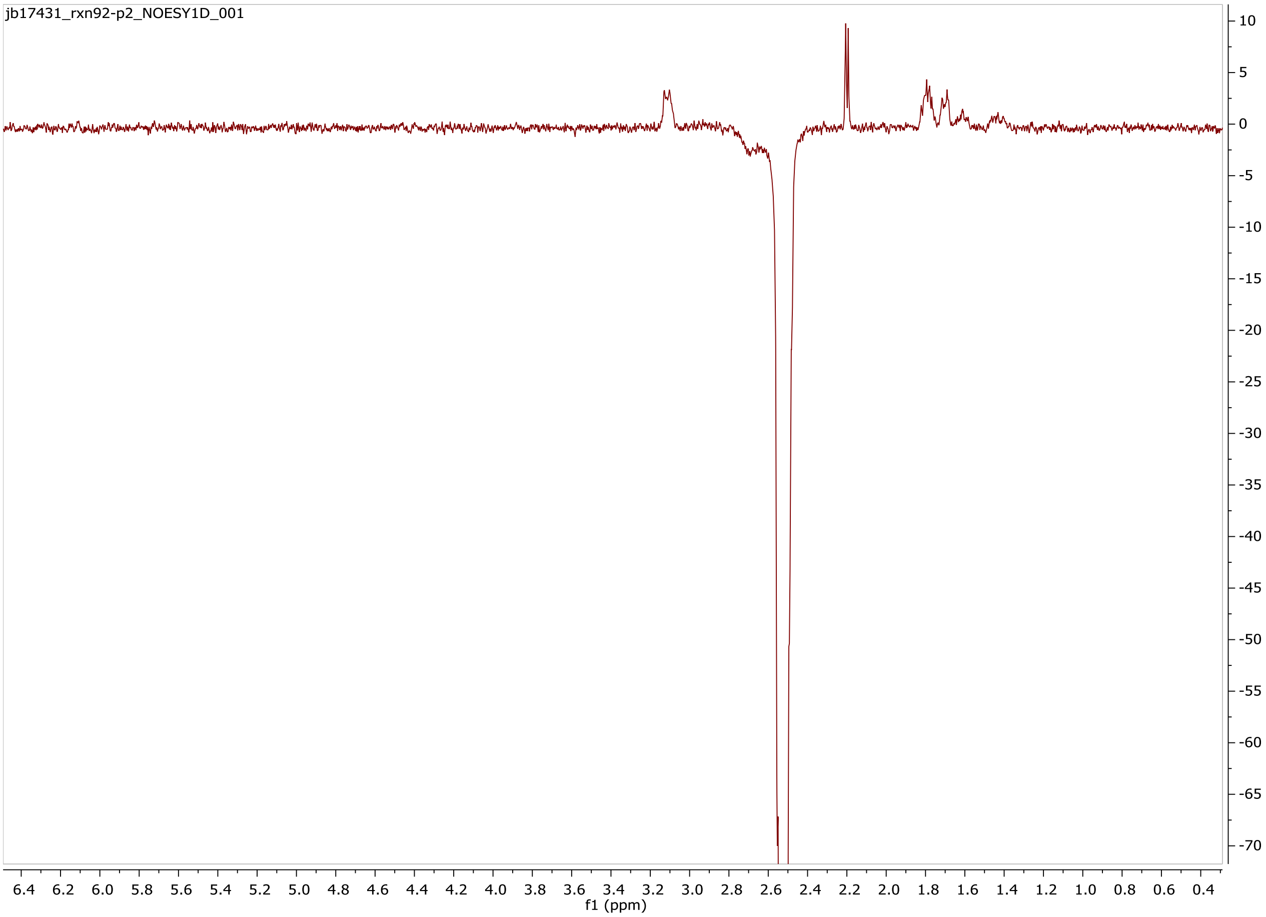

(±)-(31*R*,5a*S*,8a*S*)-N-ethyl-2-oxo-1-tosyl-1,4,5,5a,6,8a-hexahydroimidazo[4,5,1-hi]indole-31(2H)-carboxamide

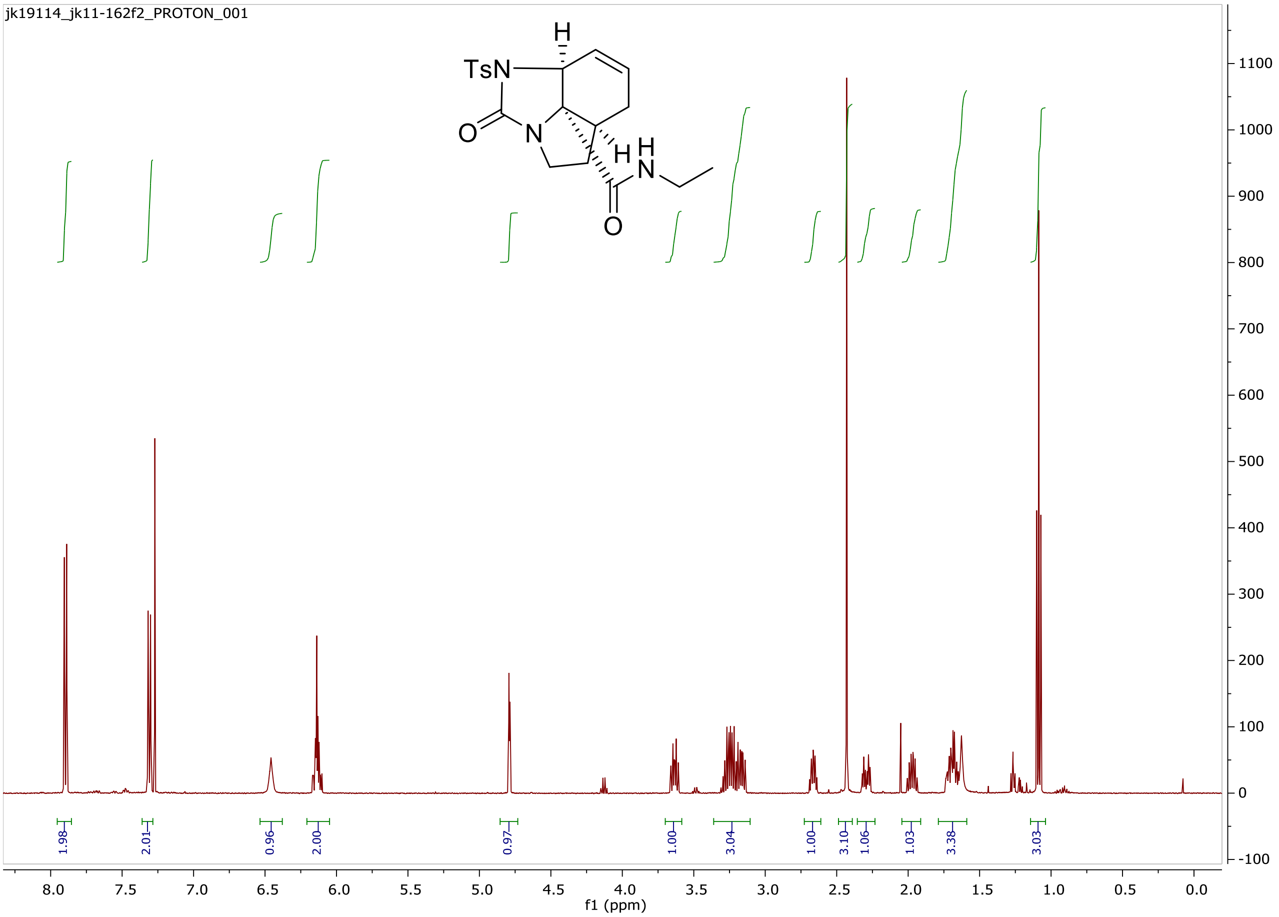

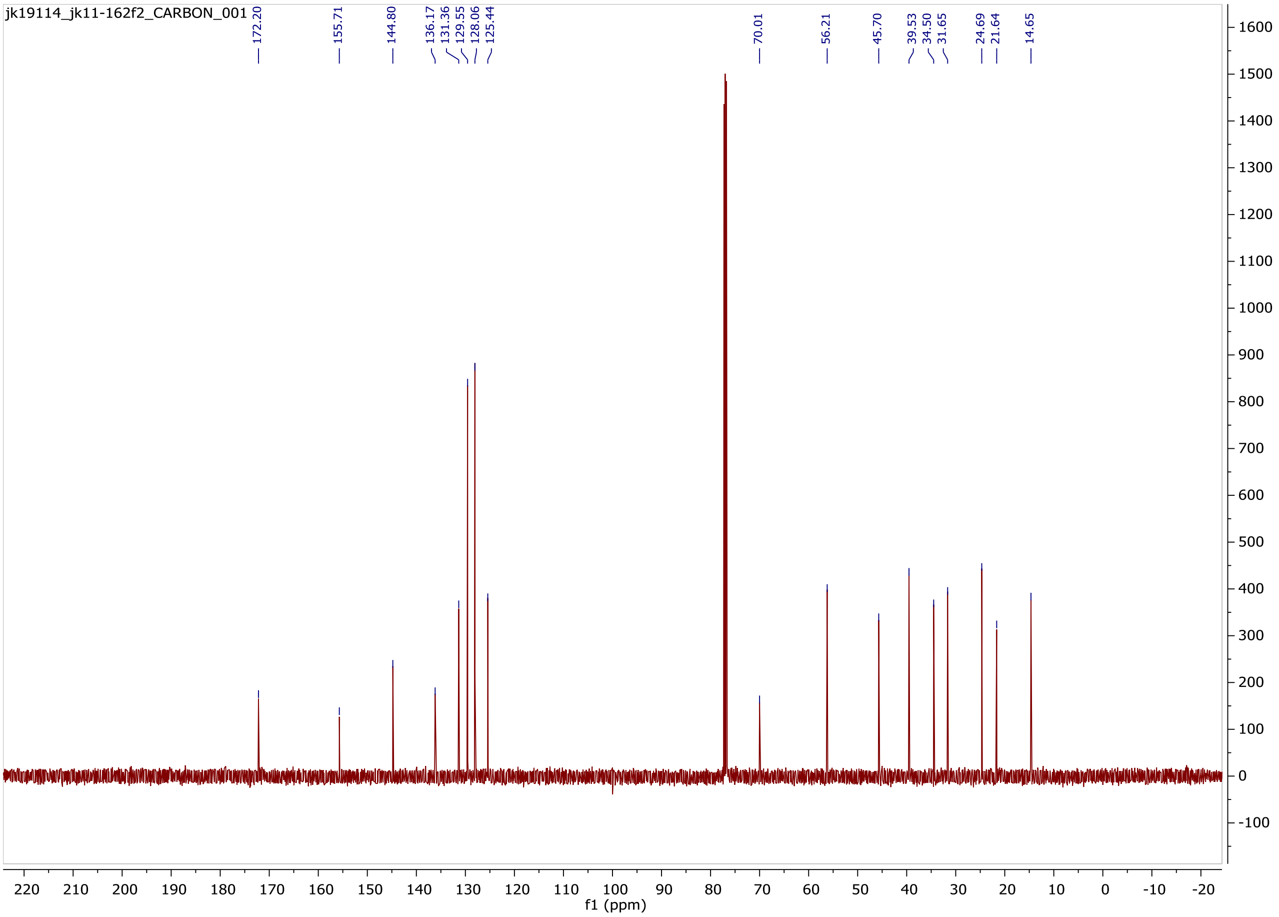

(±)-(31*R*,8a*S*)-2-oxo-1-tosyl-1,4,5,5a,6,8a-hexahydroimidazo[4,5,1-hi]indole-31(2H)-carbonitrile

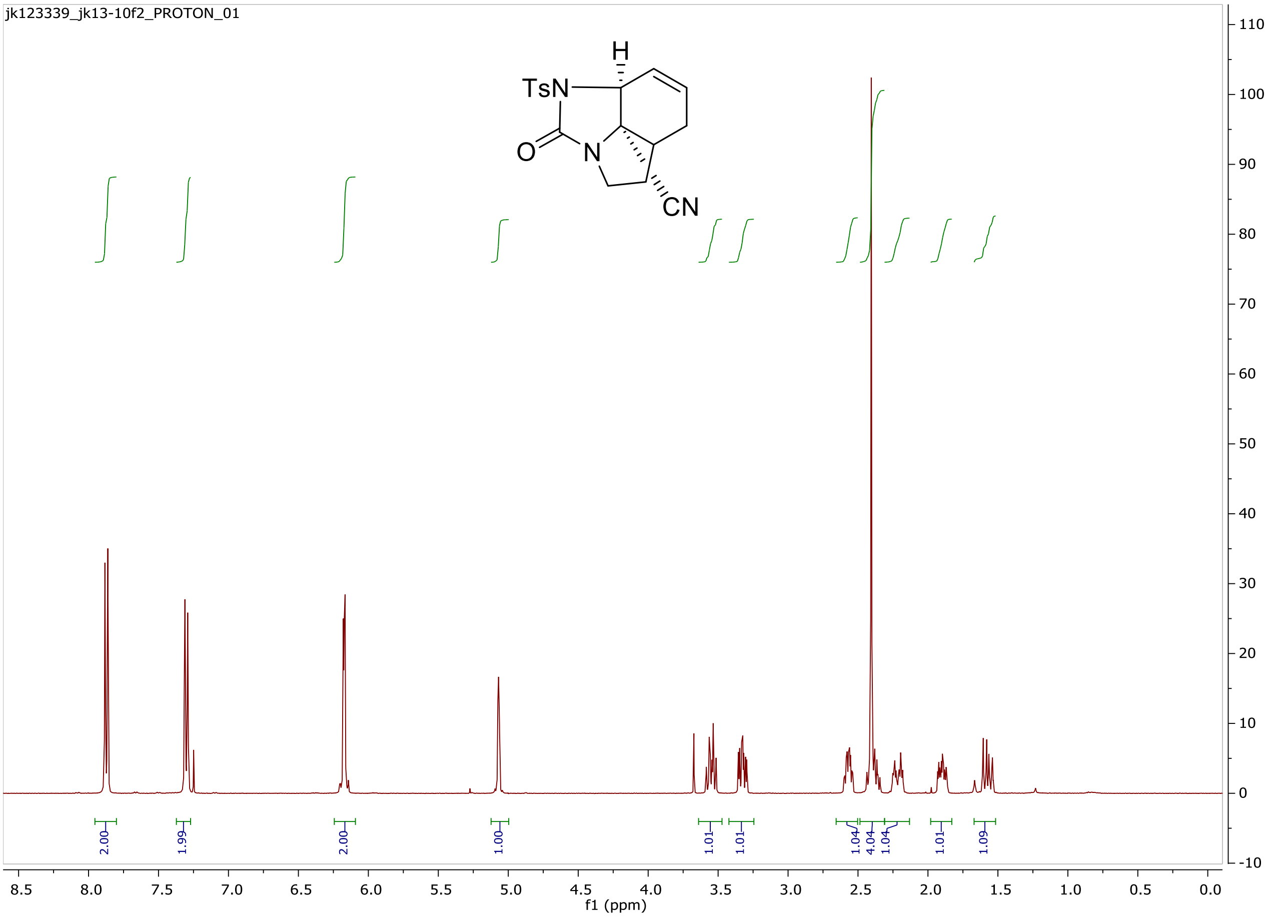

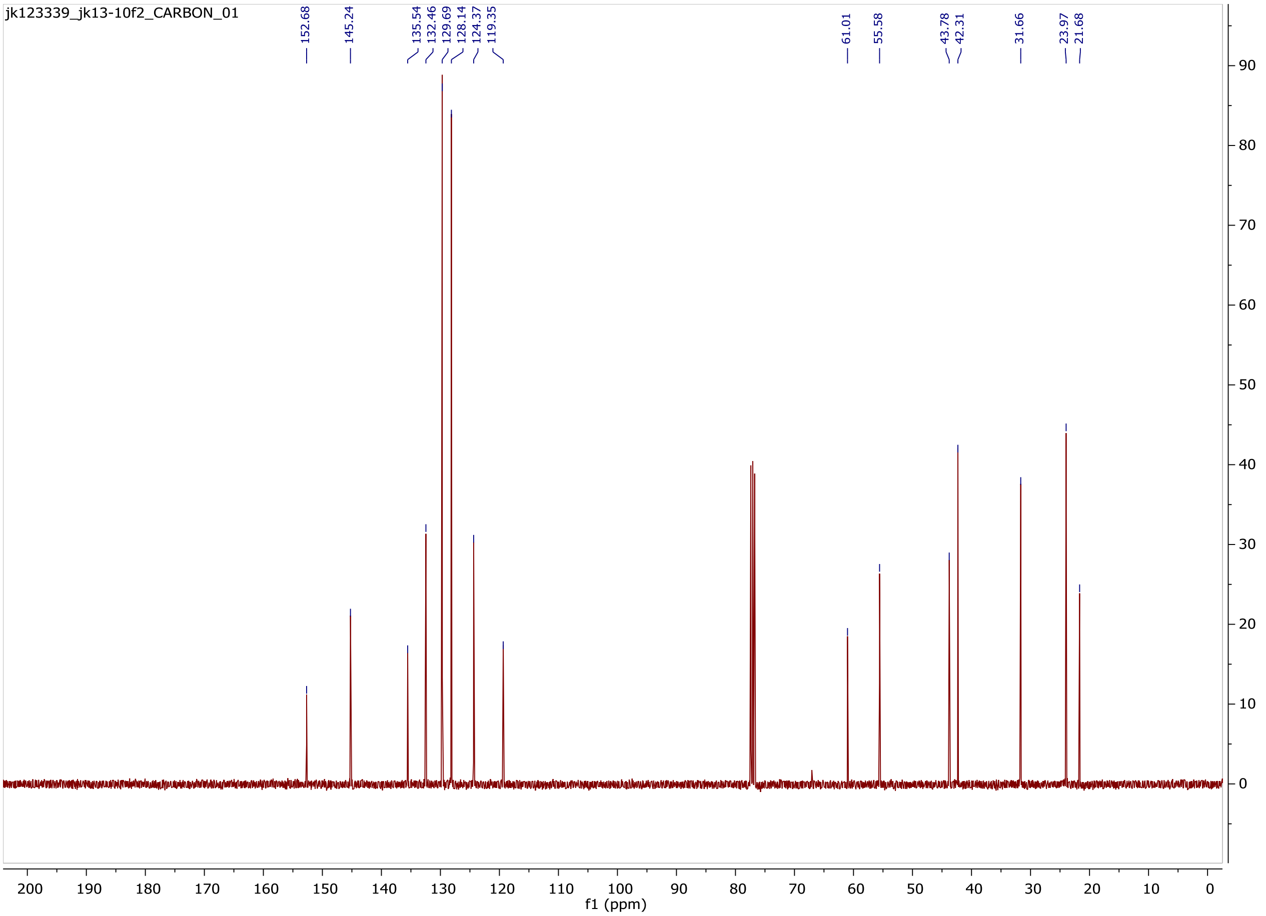

(±)-(31*R*,5a*S*,8a*S*)-31-acetyl-1-tosyl-31,4,5,5a,6,8a-hexahydroimidazo[4,5,1-hi]indol-2(1H)-one

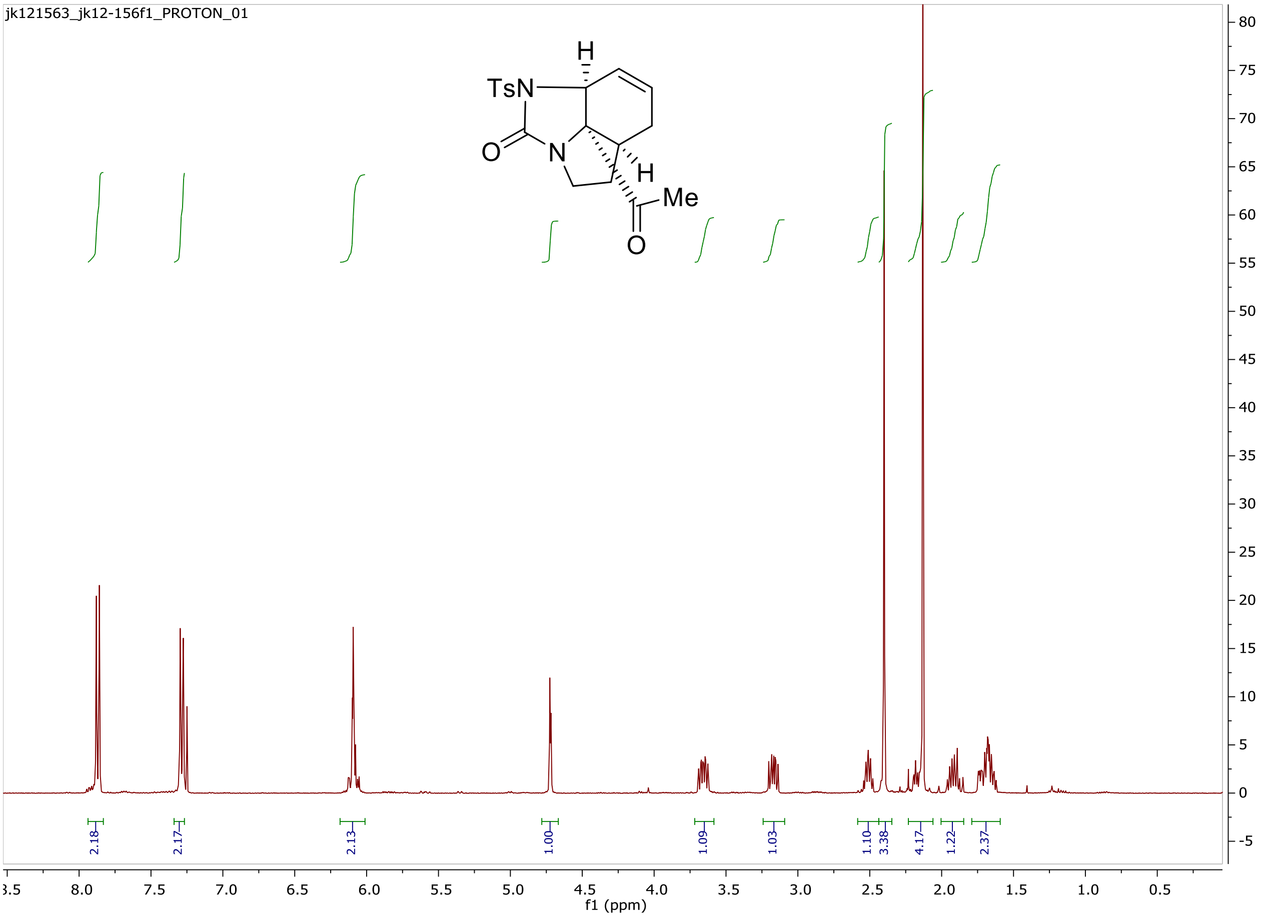

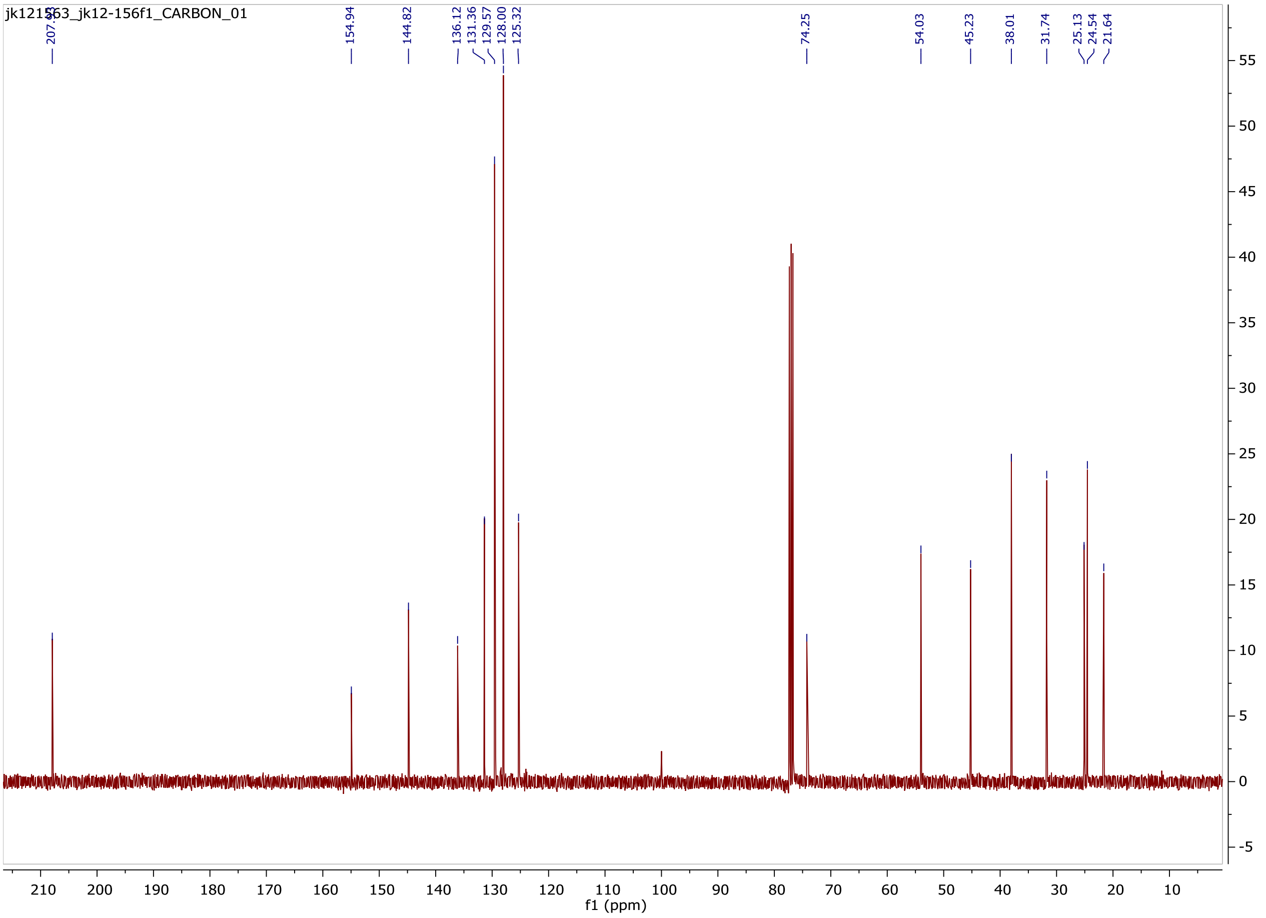

(±)-(31*R*,5a*S*,8a*S*)-1-(4-chlorophenyl)-N-ethyl-2-oxo-1,4,5,5a,6,8a-hexahydroimidazo[4,5,1-hi]indole-31(2H)-carboxamide

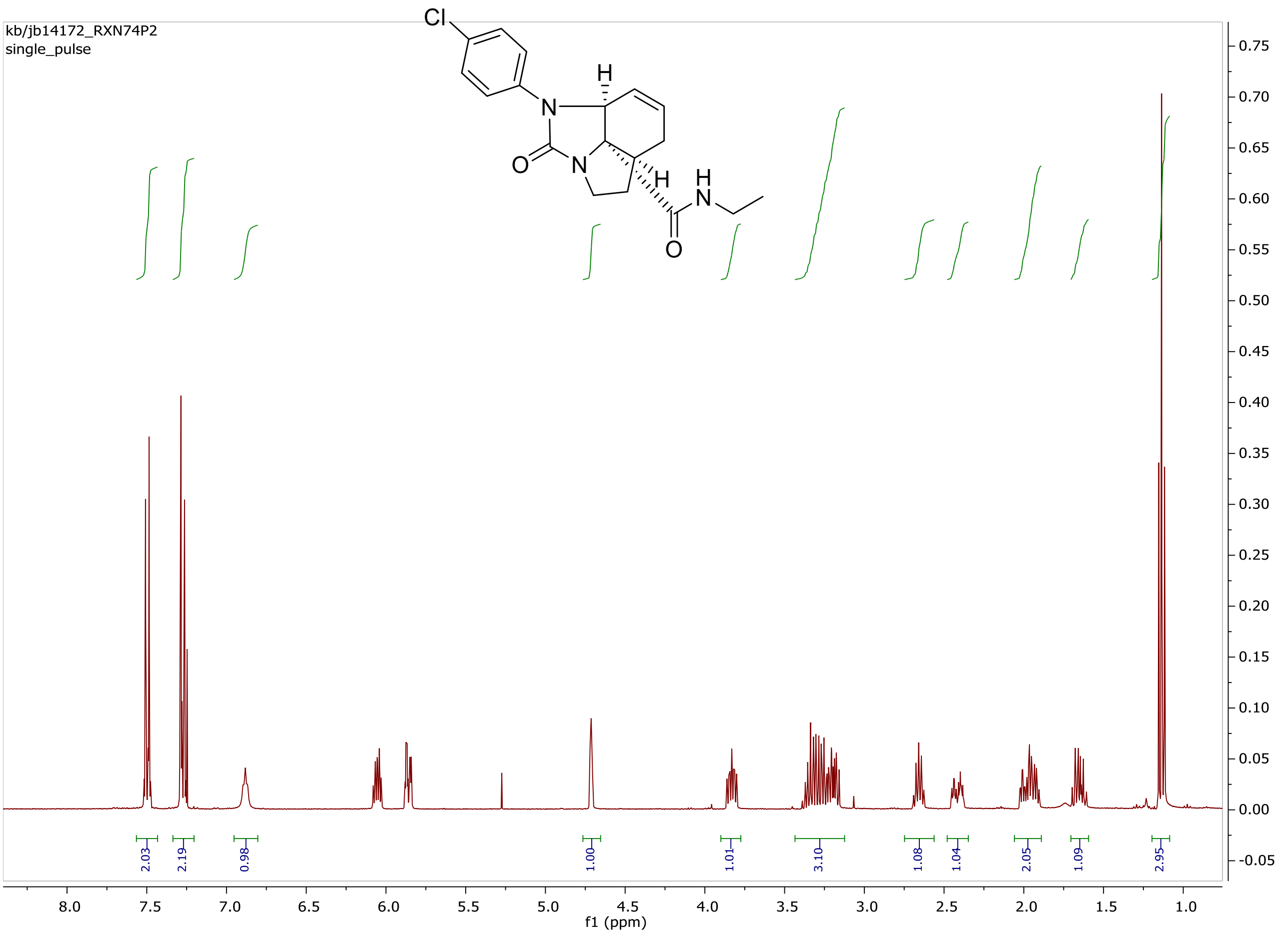

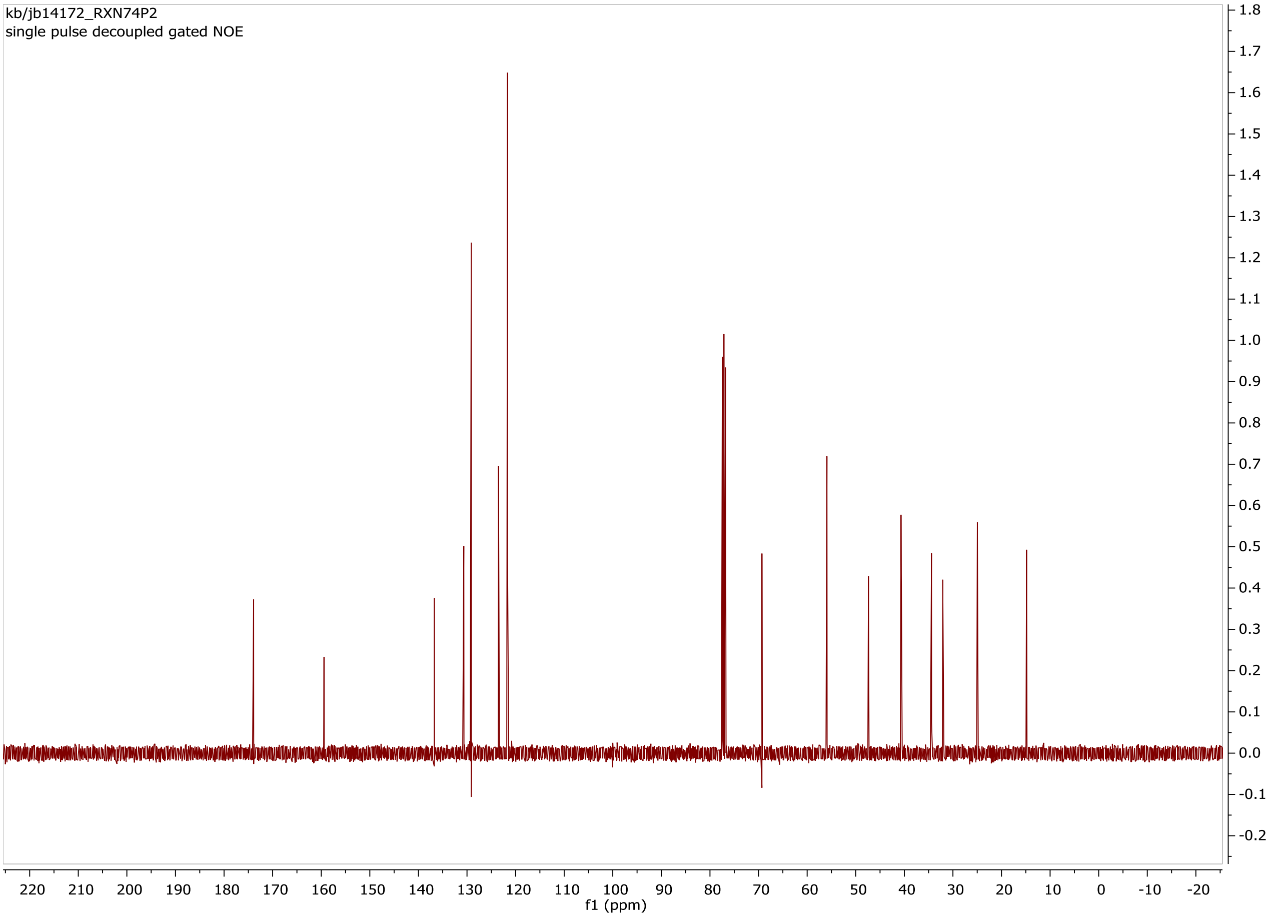

(±)-(31*R*,5a*S*,8a*S*)-1-(2-chlorophenyl)-N-ethyl-2-oxo-1,4,5,5a,6,8a-hexahydroimidazo[4,5,1-hi]indole-31(2H)-carboxamide

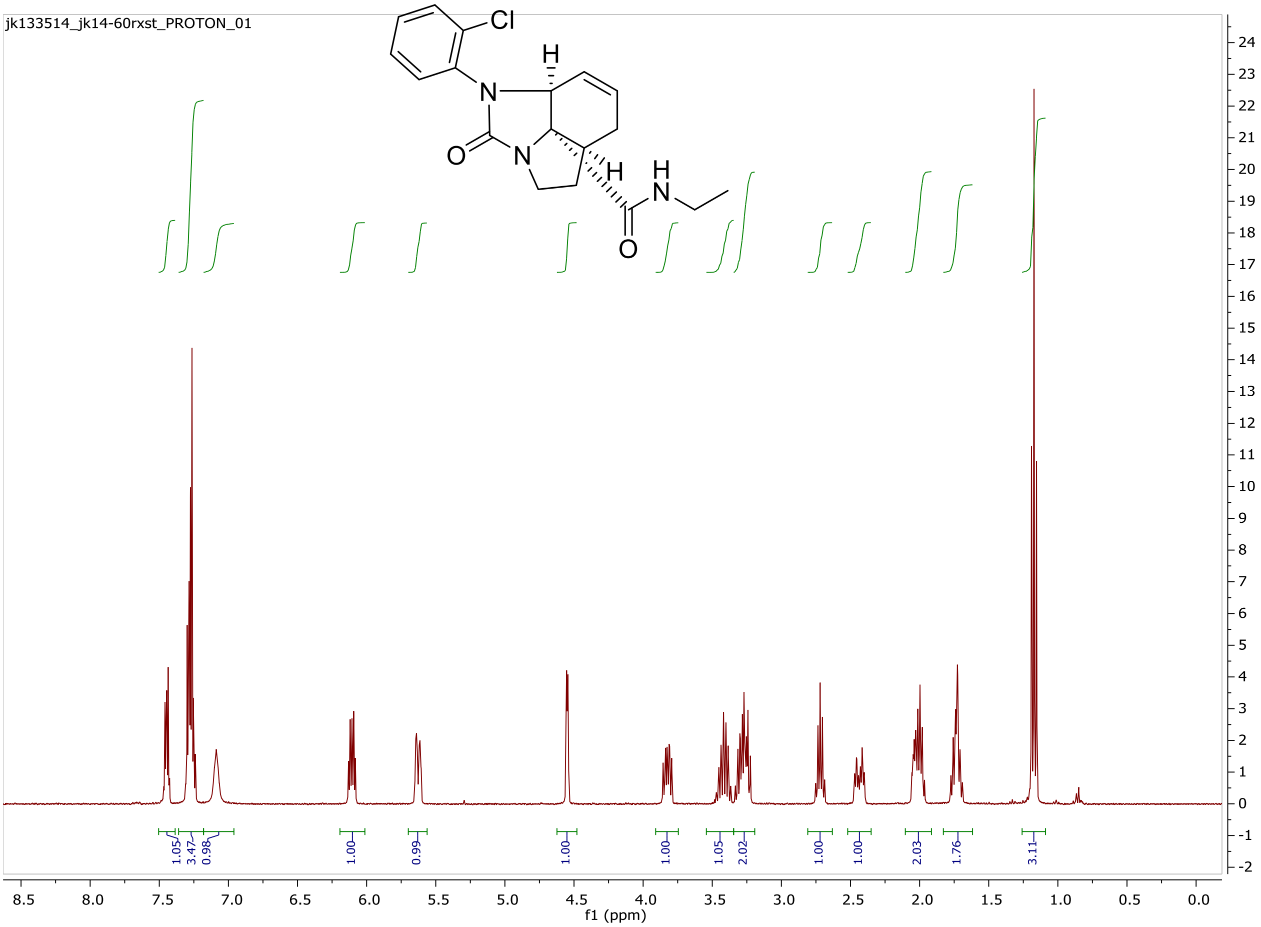

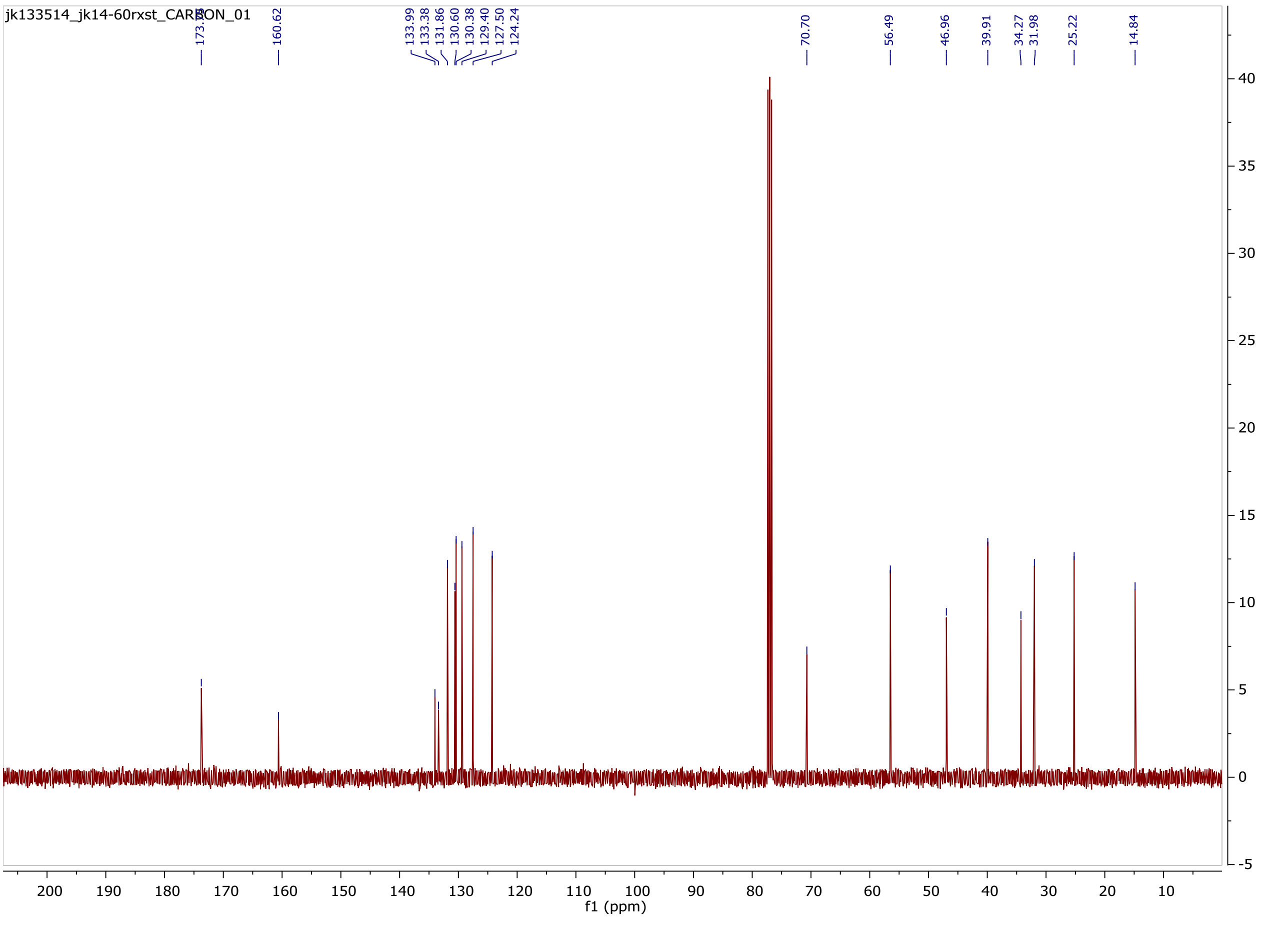

(±)-(31*R*,5a*S*,8a*S*)-1-(2-chlorophenyl)-2-oxo-1,4,5,5a,6,8a-hexahydroimidazo[4,5,1-hi]indole-31(2H)-carbonitrile

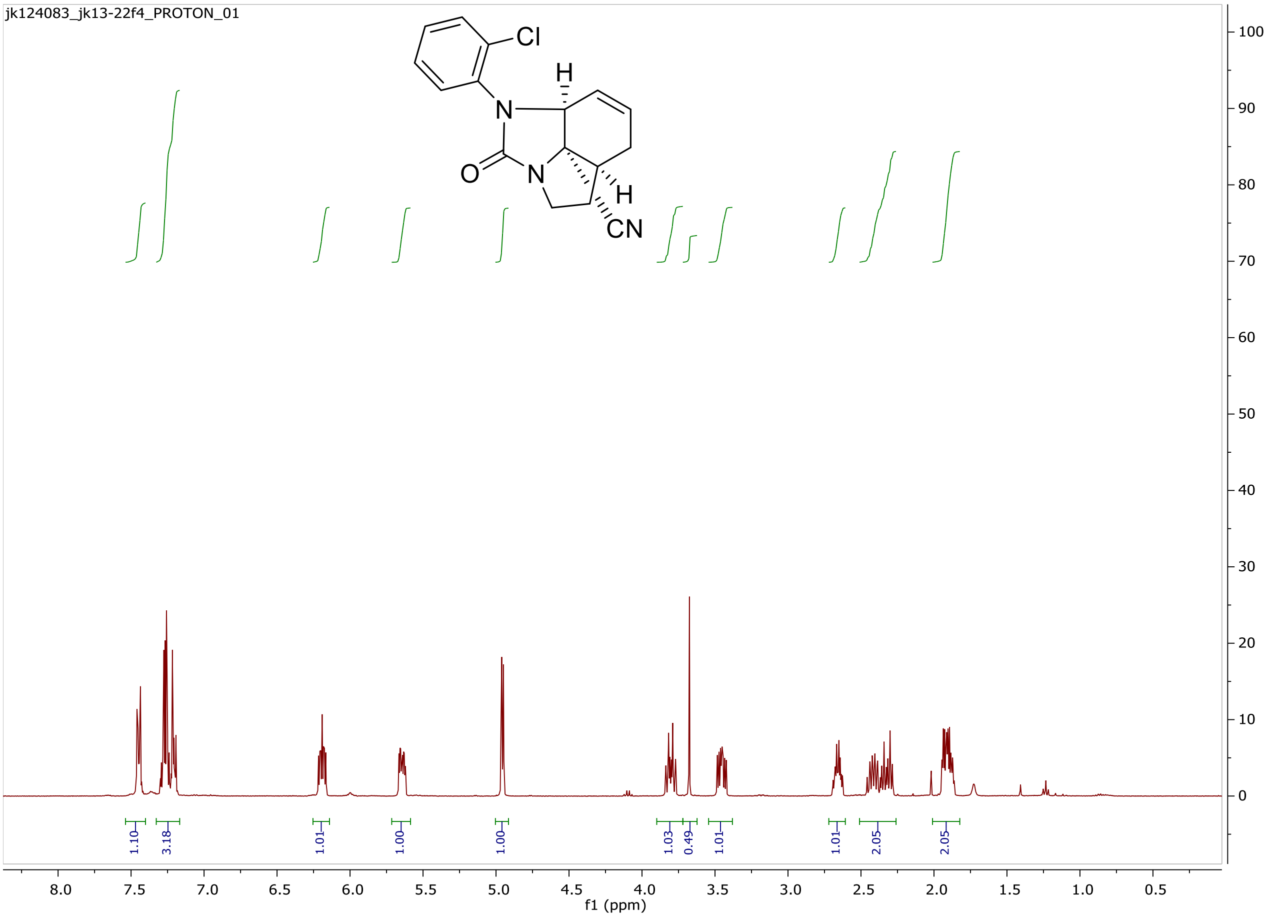

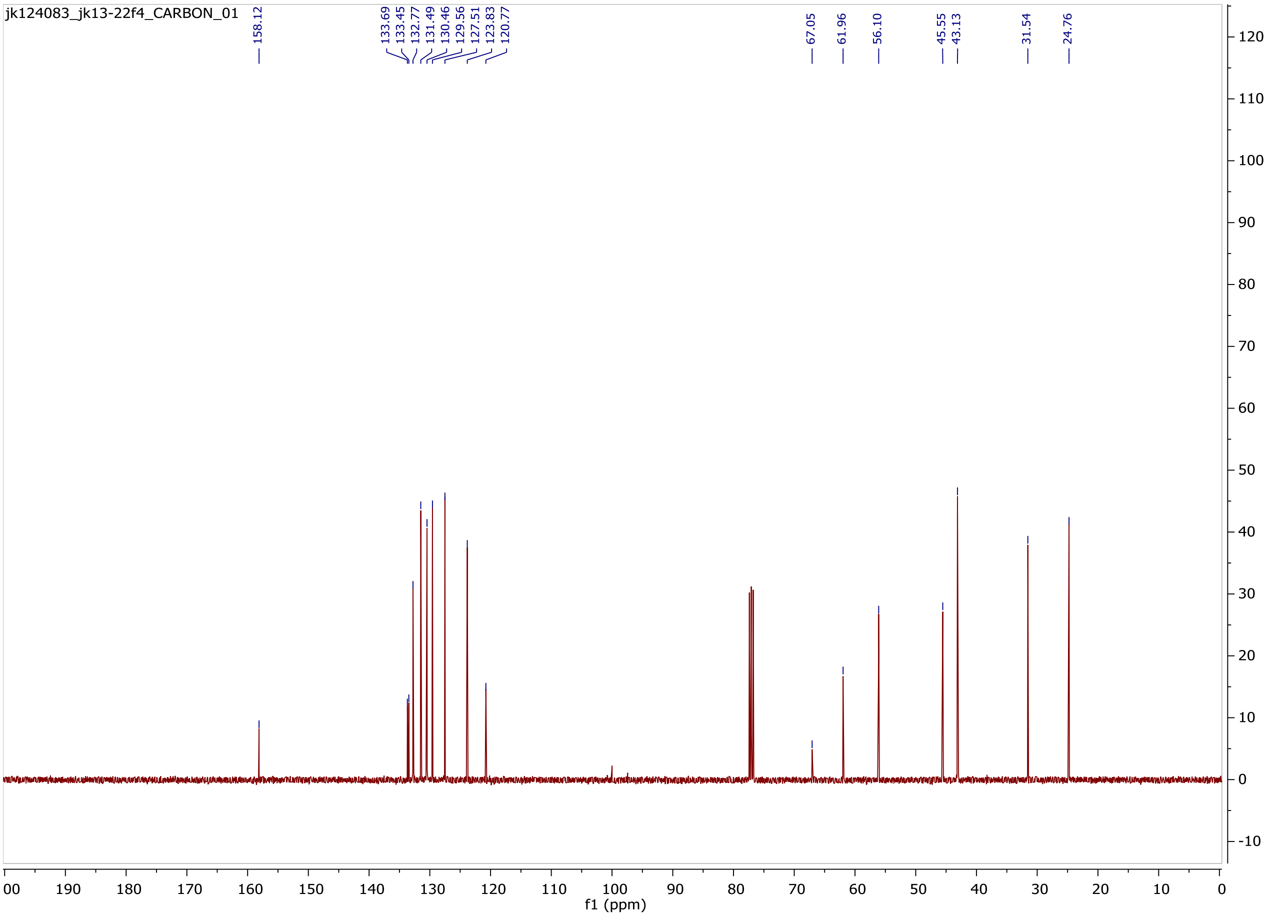

(±)-(31*R*,5a*S*,8a*S*)-N-ethyl-2-oxo-1-(2-(trifluoromethyl)phenyl)-1,4,5,5a,6,8a-hexahydroimidazo[4,5,1-hi]indole-31(2H)-carboxamide

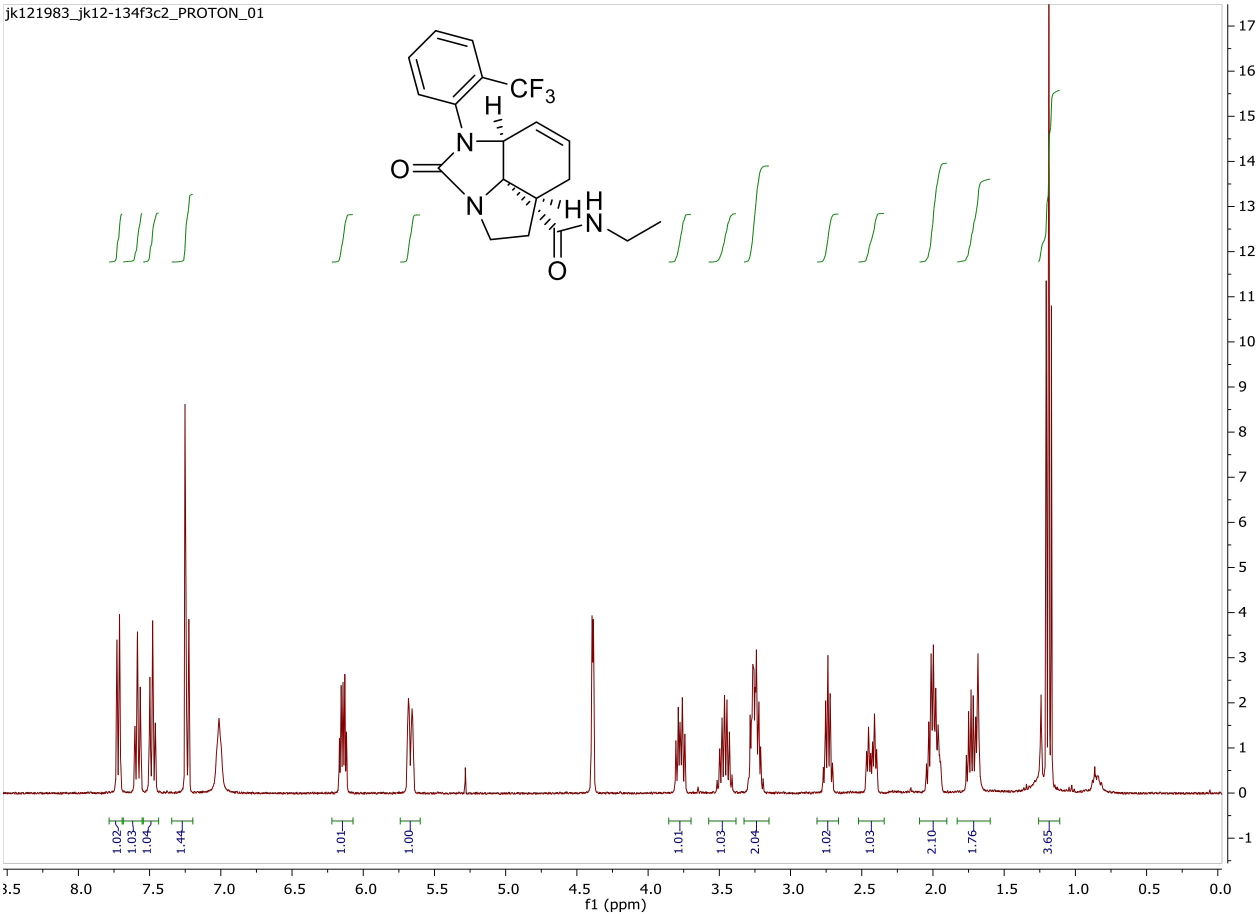

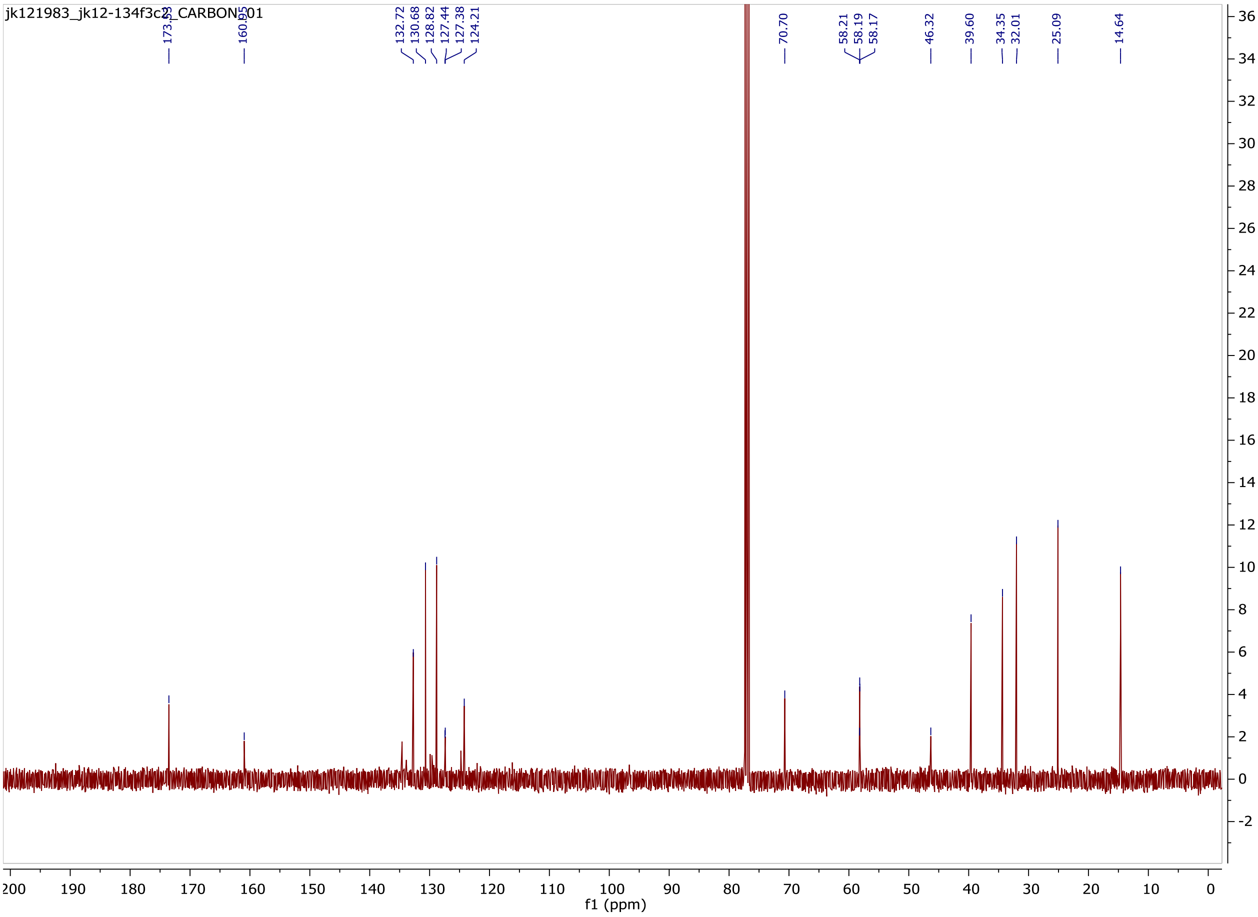

(±)-(31*R*,5a*S*,8a*S*)-N-ethyl-2-oxo-1-(3-(trifluoromethyl)phenyl)-1,4,5,5a,6,8a-hexahydroimidazo[4,5,1-hi]indole-31(2H)-carboxamide

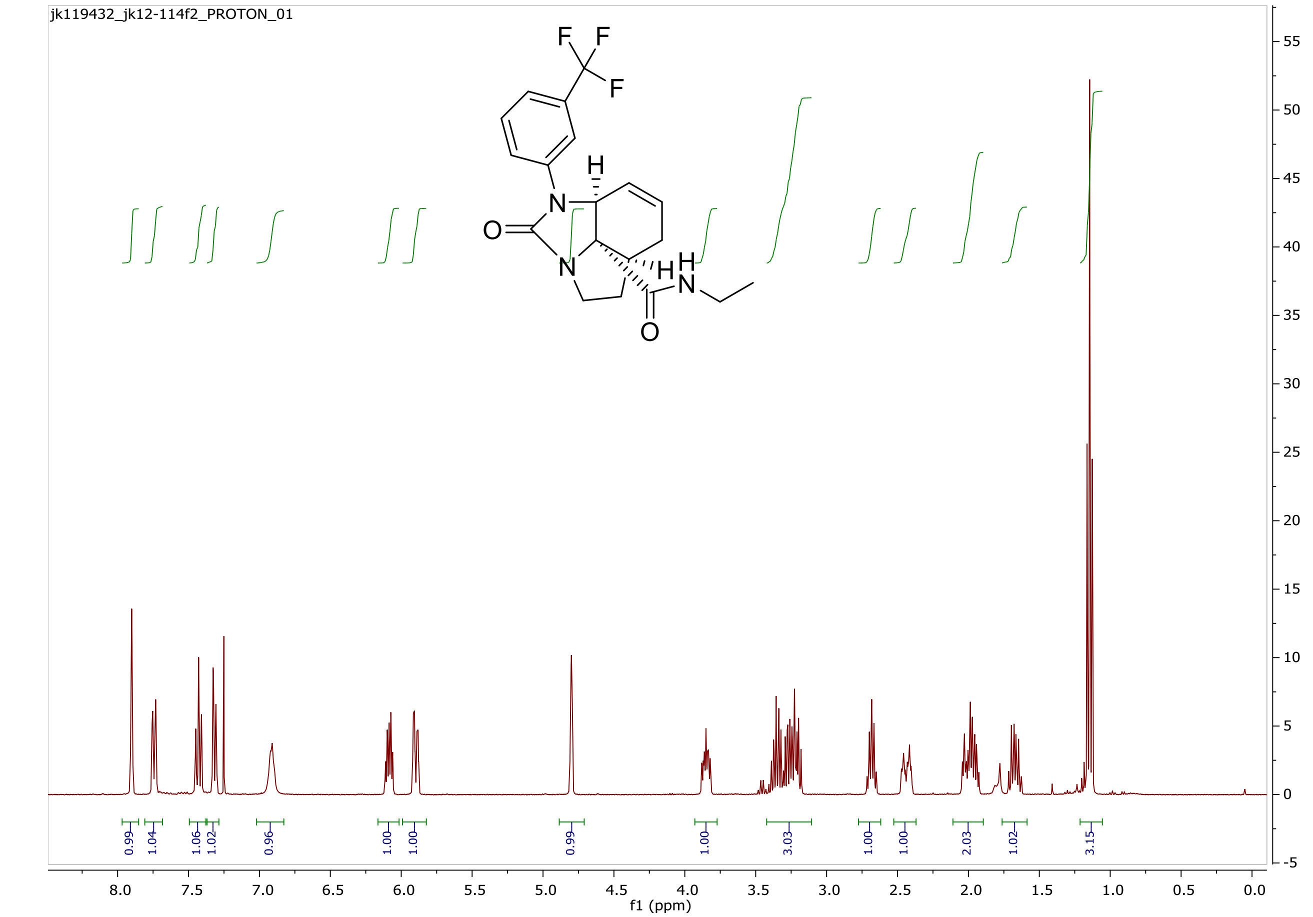

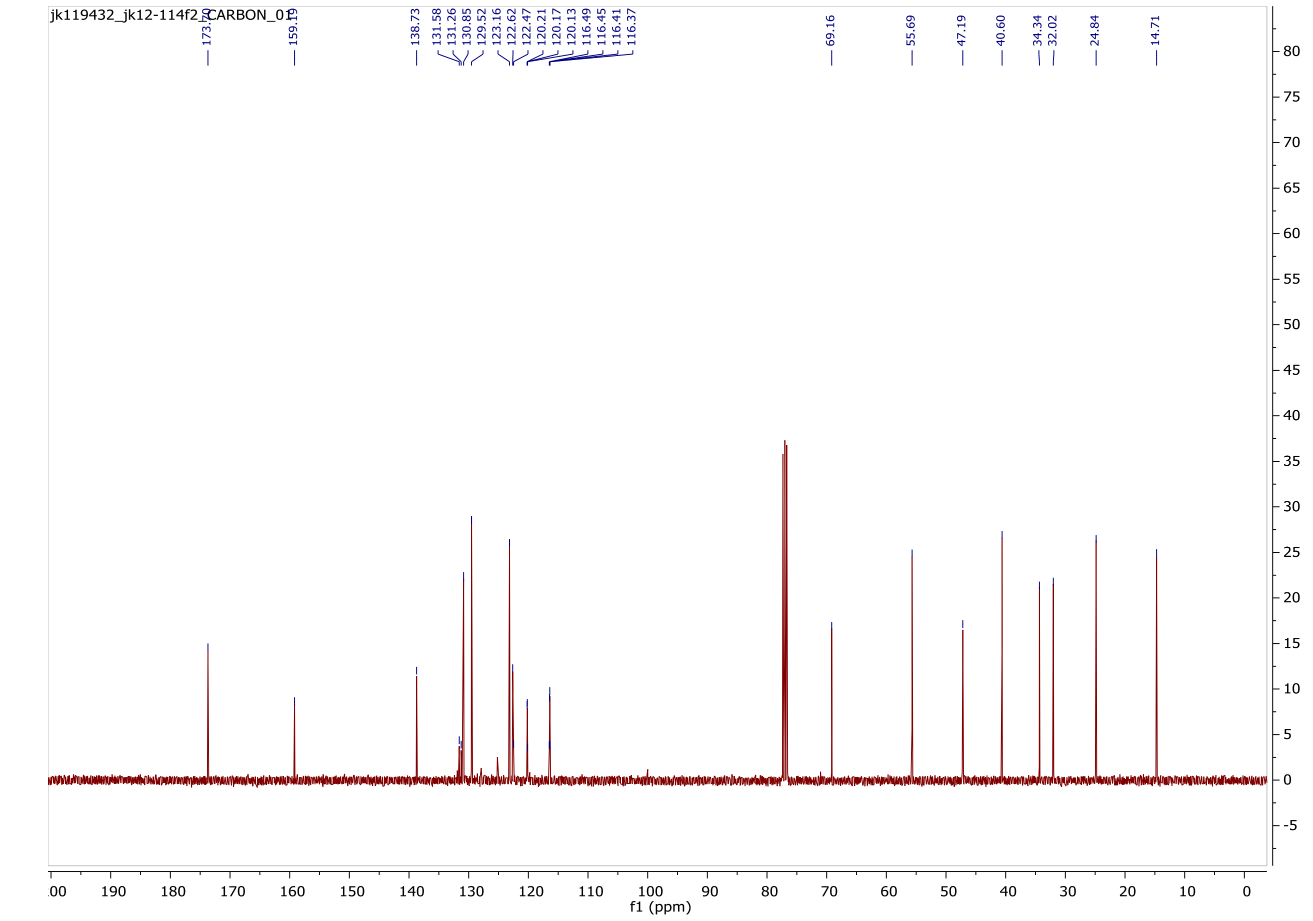

(±)-(31*R*,5a*S*,8a*S*)-N-ethyl-2-oxo-1-(4-(trifluoromethyl)phenyl)-1,4,5,5a,6,8a-hexahydroimidazo[4,5,1-hi]indole-31(2H)-carboxamide

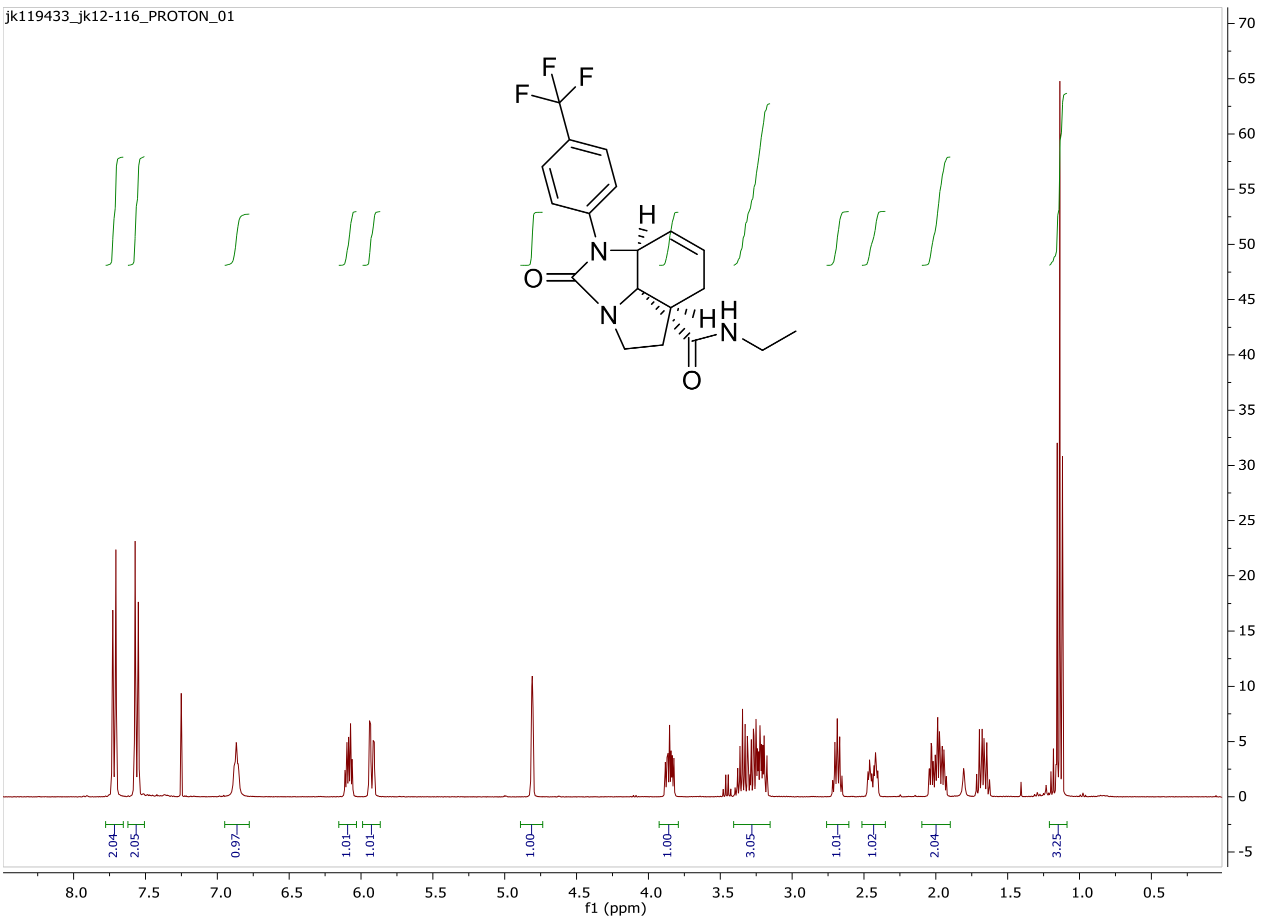

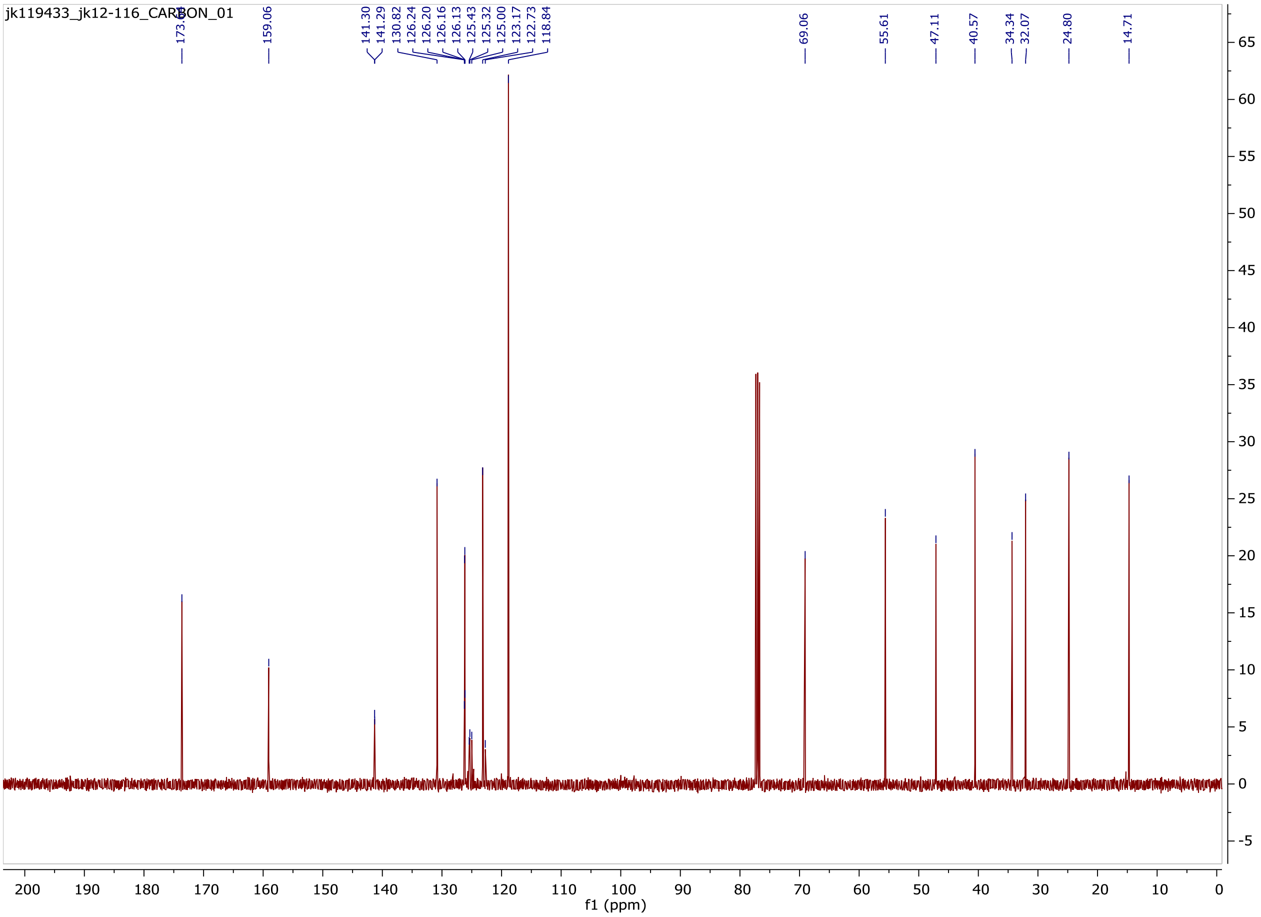

(±)-(31*R*,5a*S*,8a*S*)-31-acetyl-1-(4-(trifluoromethyl)phenyl)-31,4,5,5a,6,8a-hexahydroimidazo[4,5,1-hi]indol-2(1H)-one

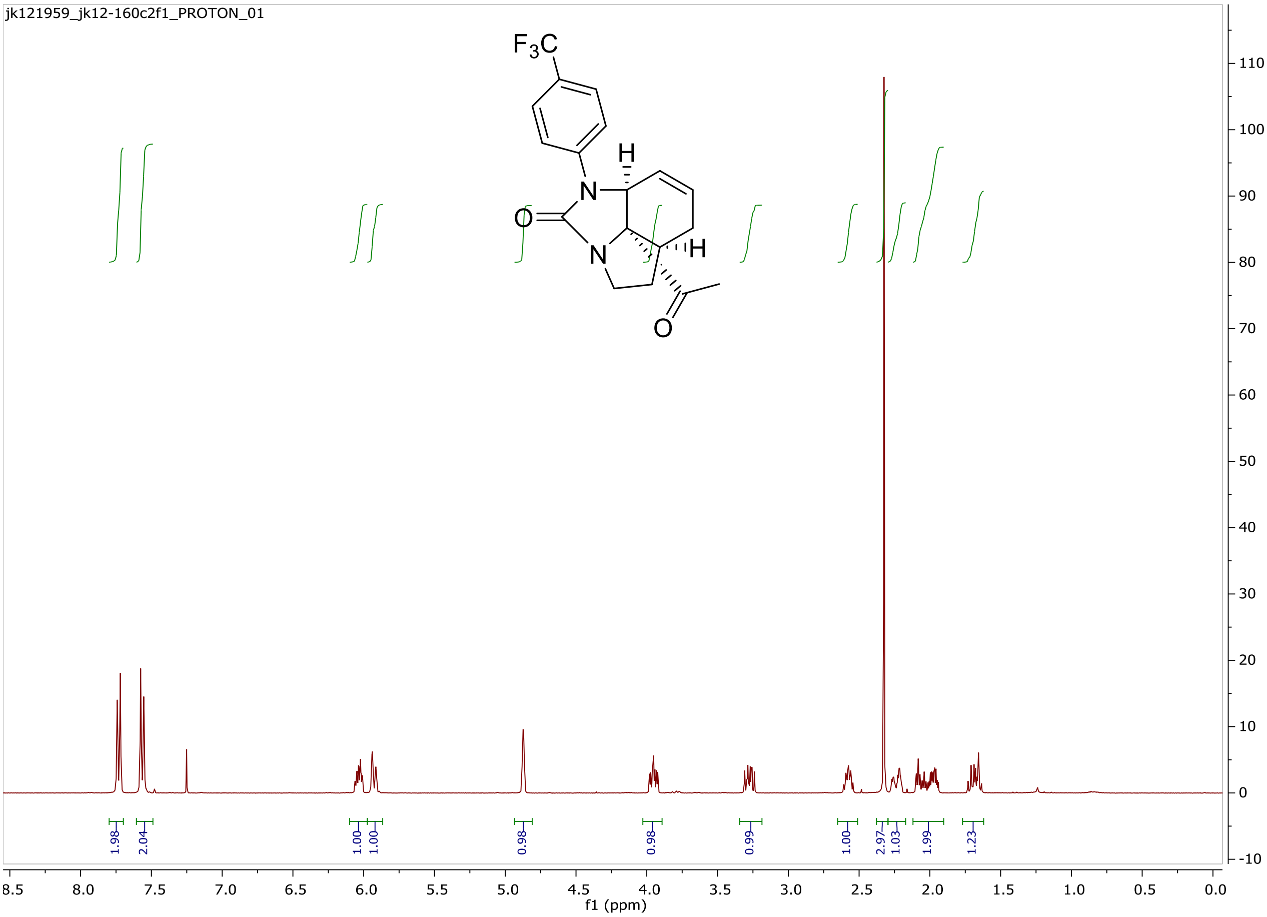

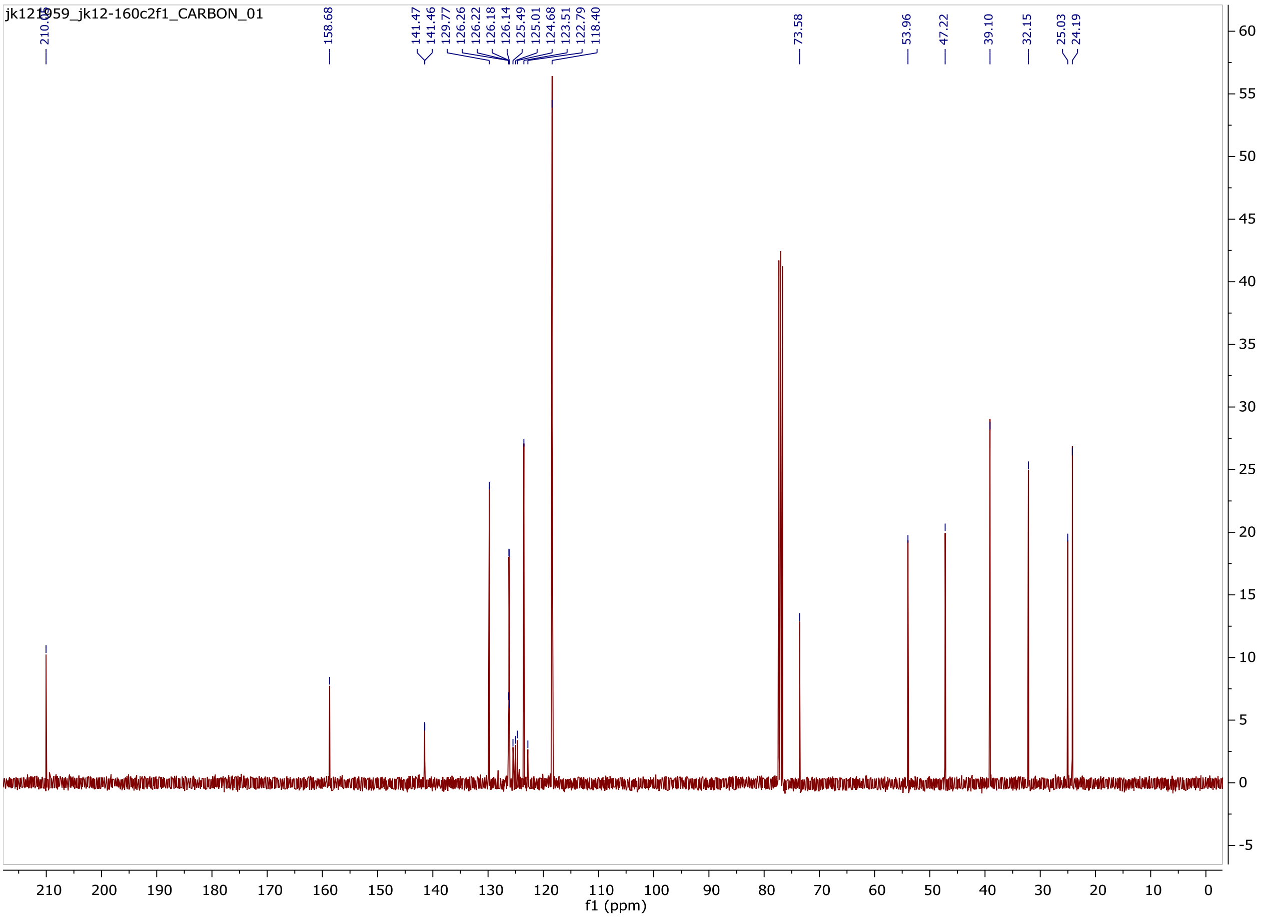

(±)-(31*R*,5a*S*,8a*S*)-N-ethyl-2-oxo-1-(2-(nitro)phenyl)-1,4,5,5a,6,8a-hexahydroimidazo[4,5,1-hi]indole-31(2H)-carboxamide

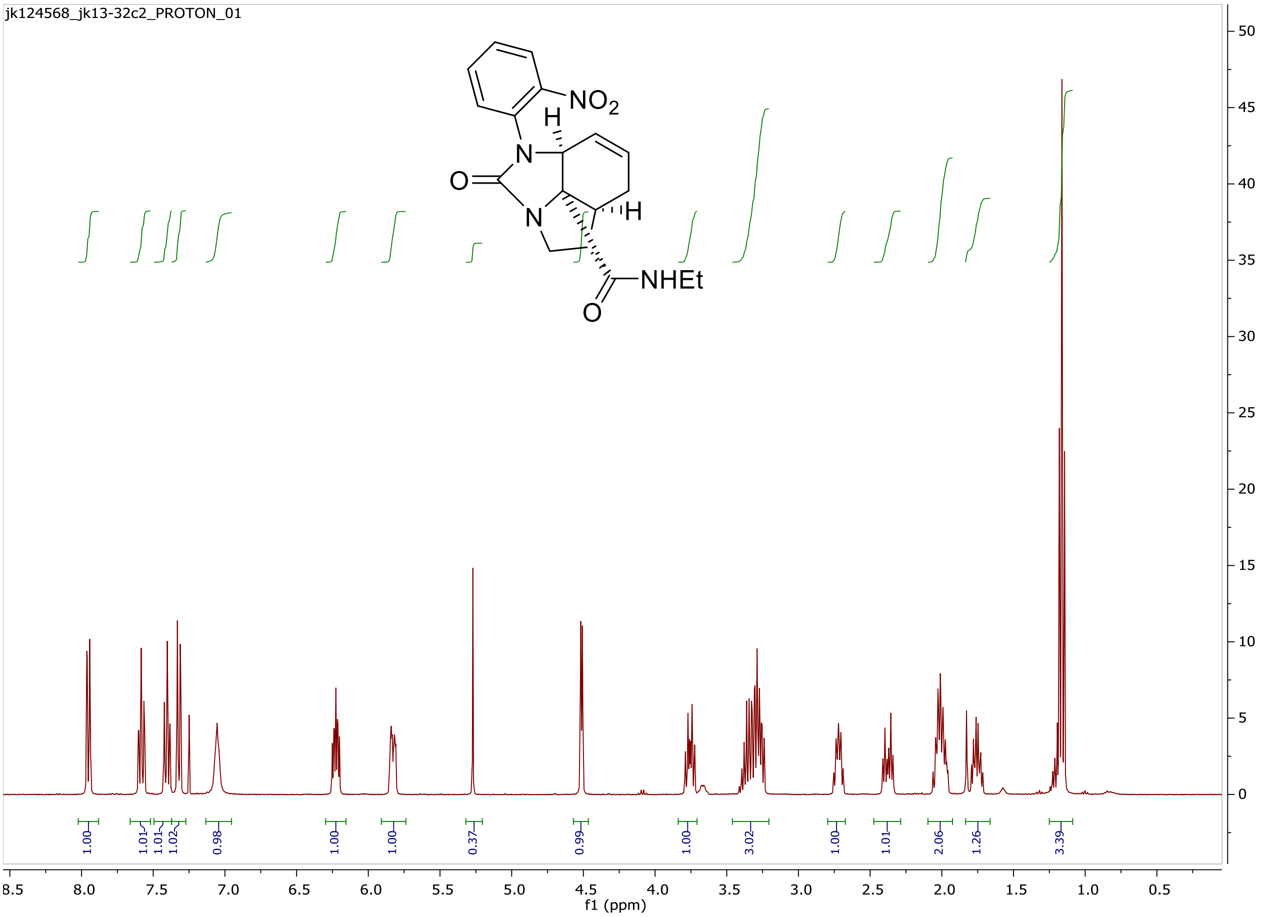

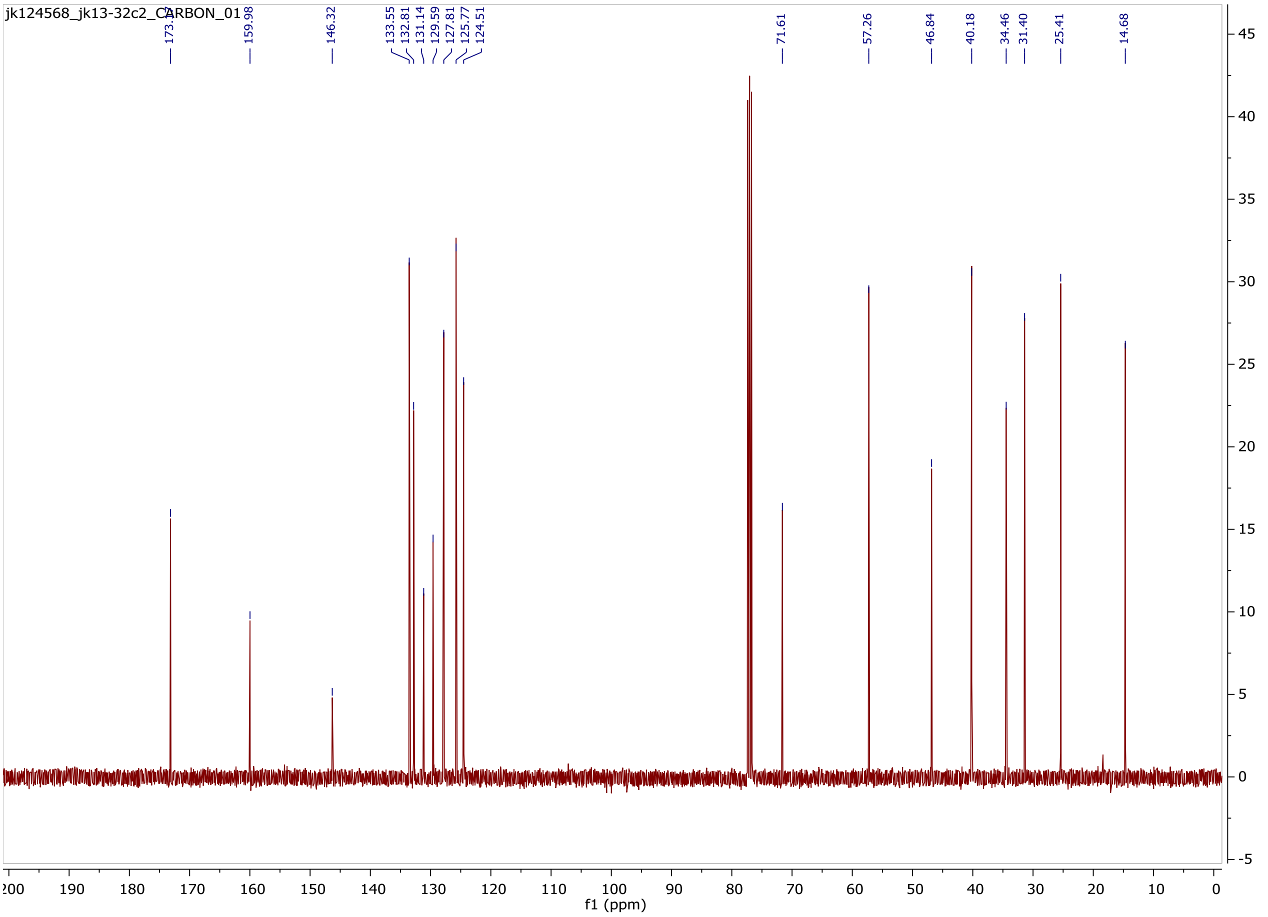

(±)-(31*R*,5a*S*,8a*S*)-1-(2-nitrophenyl)-2-oxo-1,4,5,5a,6,8a-hexahydroimidazo[4,5,1-hi]indole-31(2H)-carbonitrile

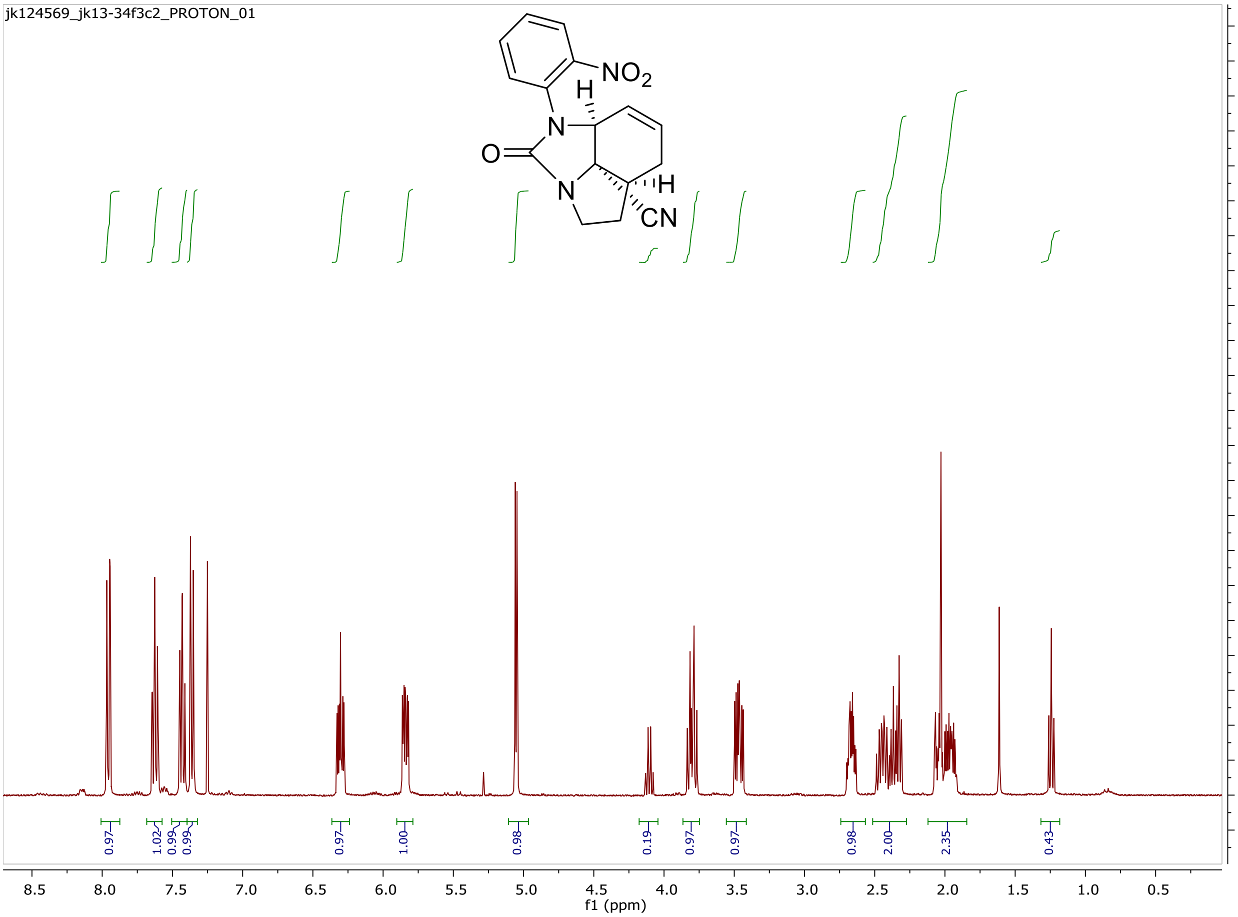

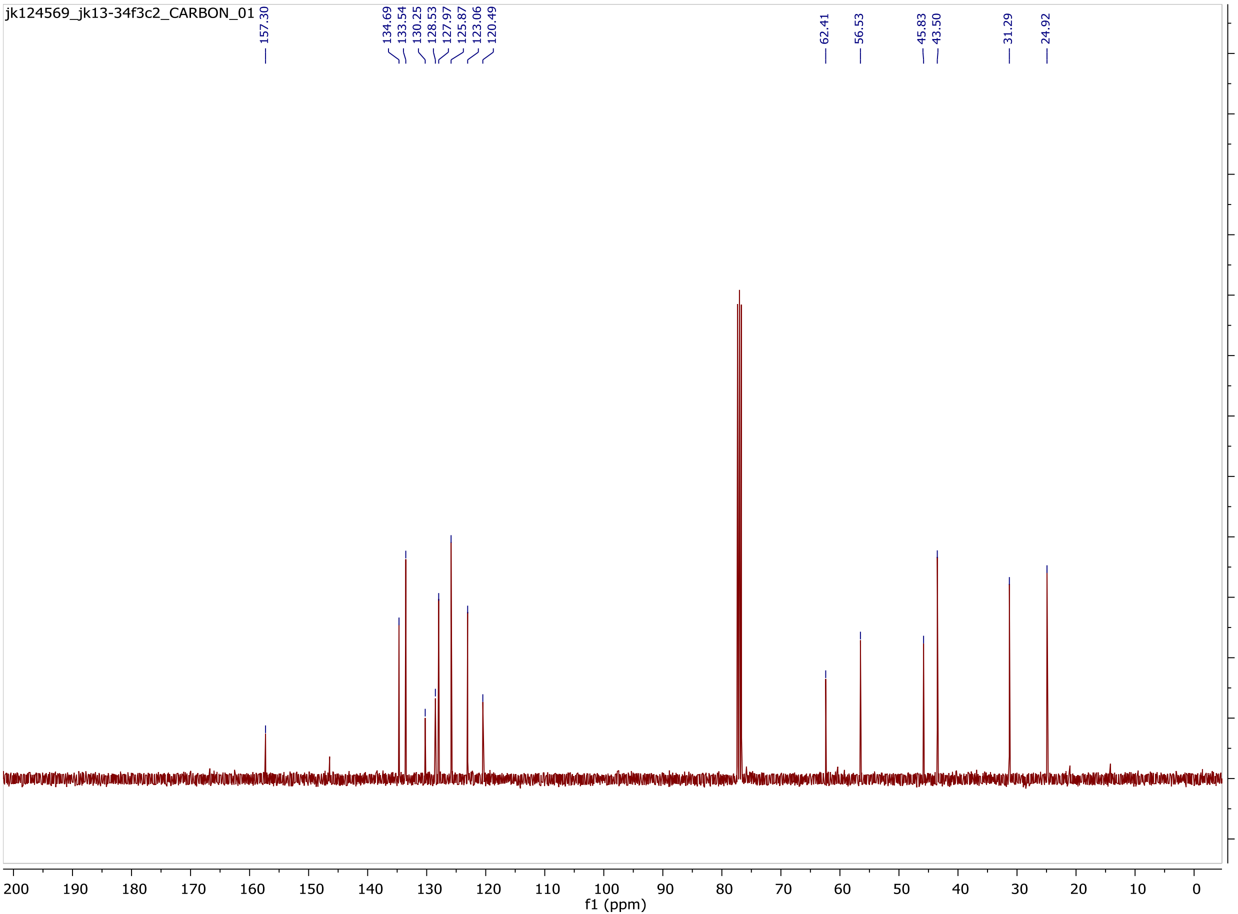

(±)-(31*R*,5a*S*,8a*S*)-1-(4-acetylphenyl)-N-ethyl-2-oxo-1,4,5,5a,6,8a-hexahydroimidazo[4,5,1-hi]indole-31(2H)-carboxamide

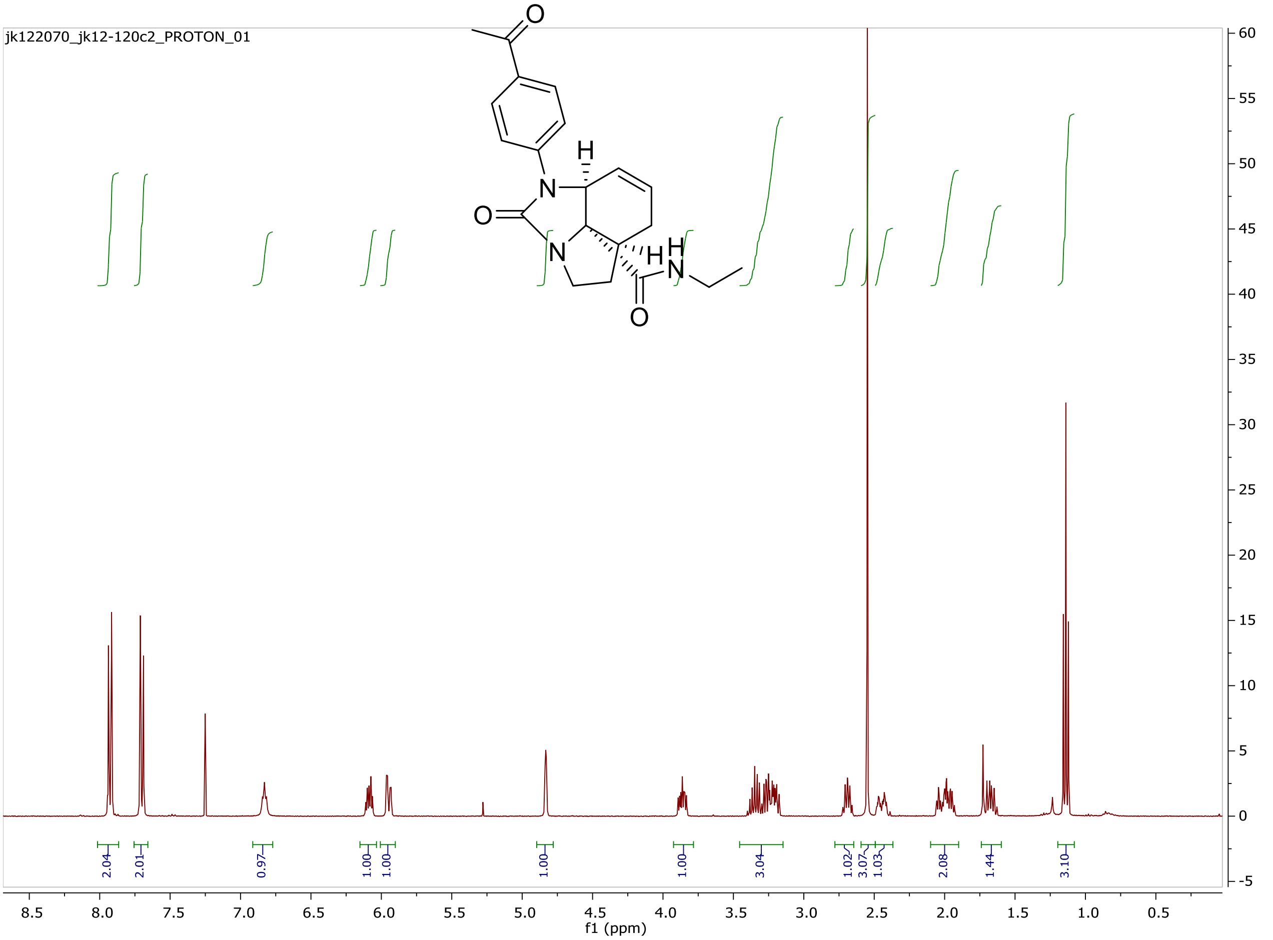

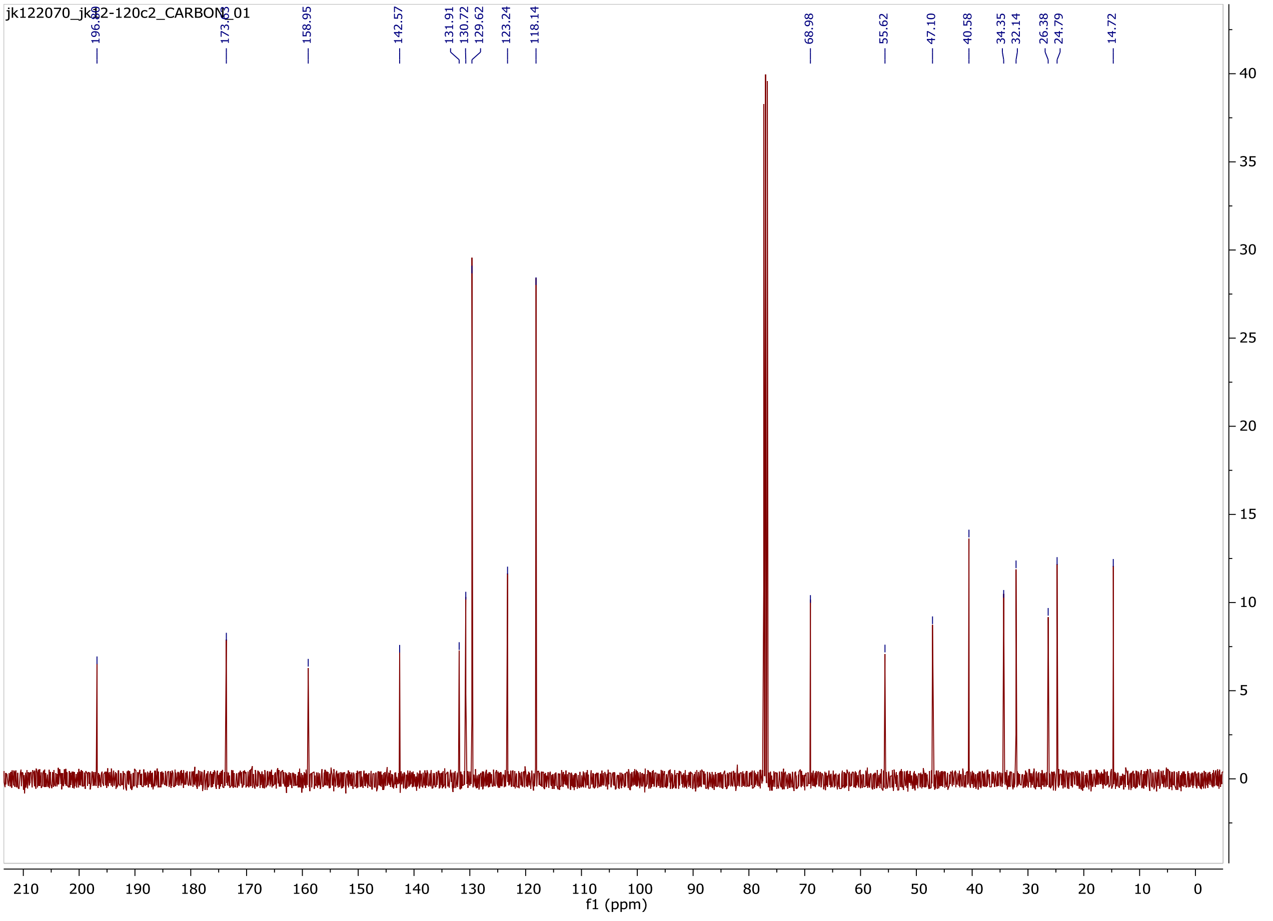

(±)-(31*R*,5a*S*,8a*S*)-N-ethyl-1-(4-methoxyphenyl)-2-oxo-1,4,5,5a,6,8a-hexahydroimidazo[4,5,1-hi]indole-31(2H)-carboxamide

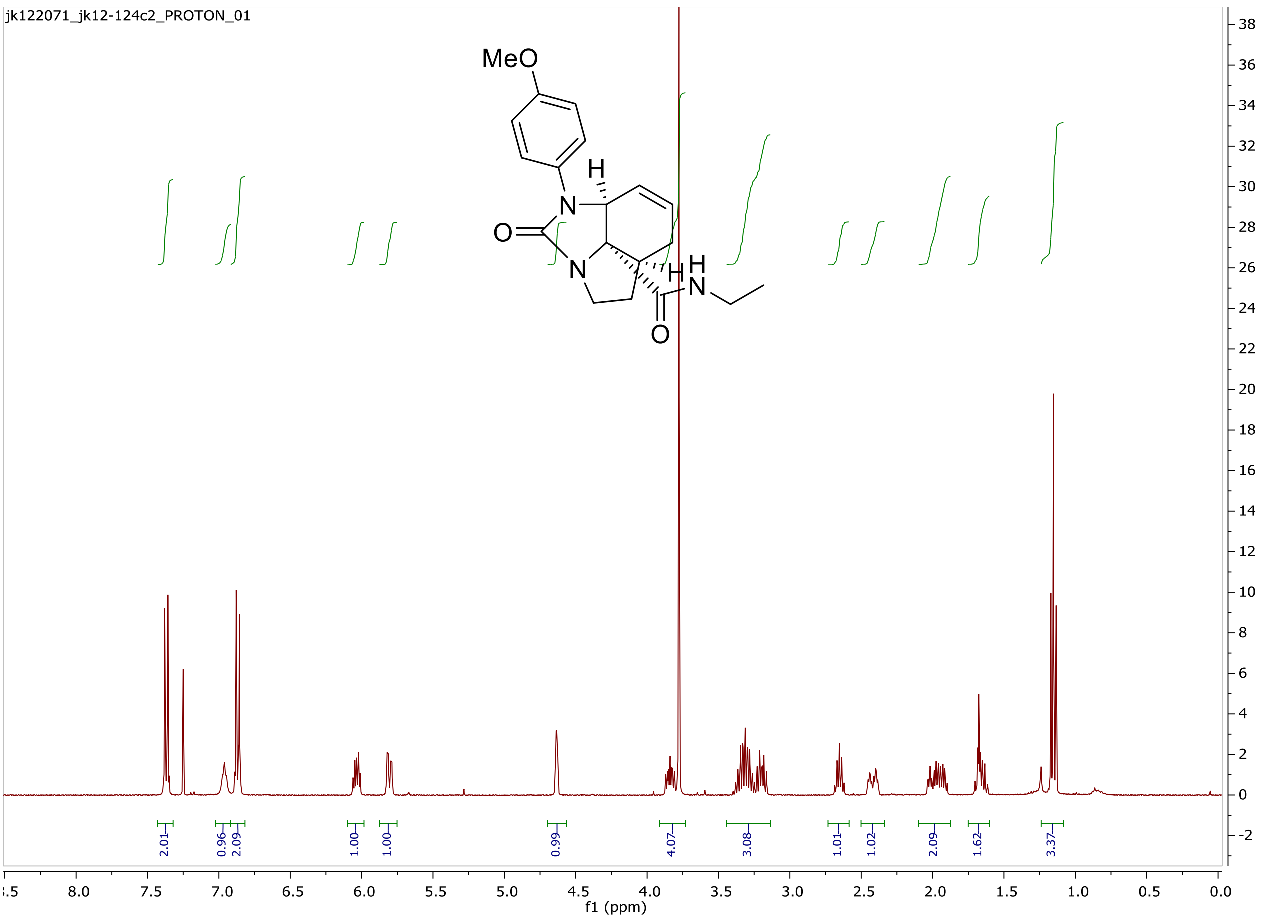

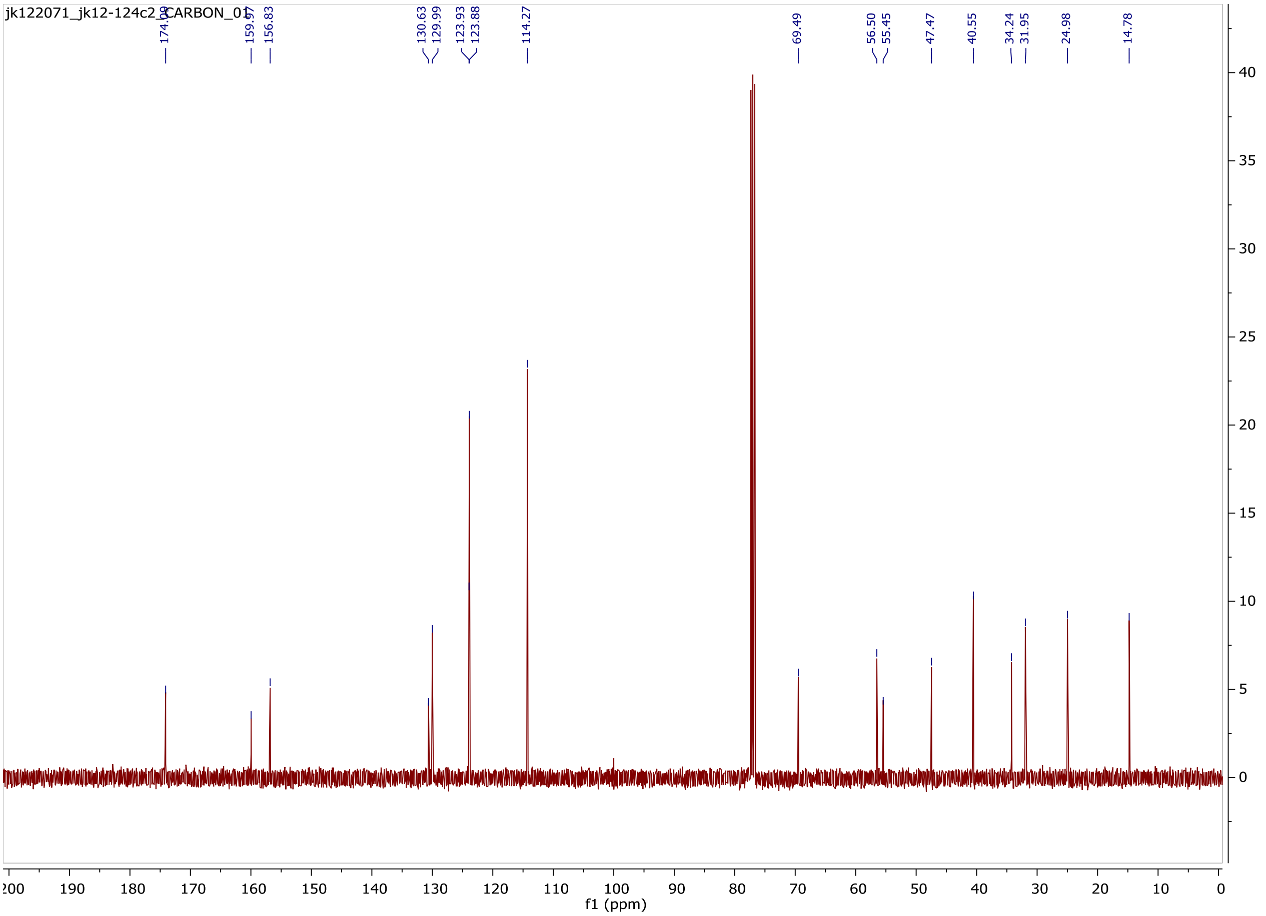

(±)-(31*R*,5a*S*,8a*S*)-N-ethyl-1-(2-methoxyphenyl)-2-oxo-1,4,5,5a,6,8a-hexahydroimidazo[4,5,1-hi]indole-31(2H)-carboxamide

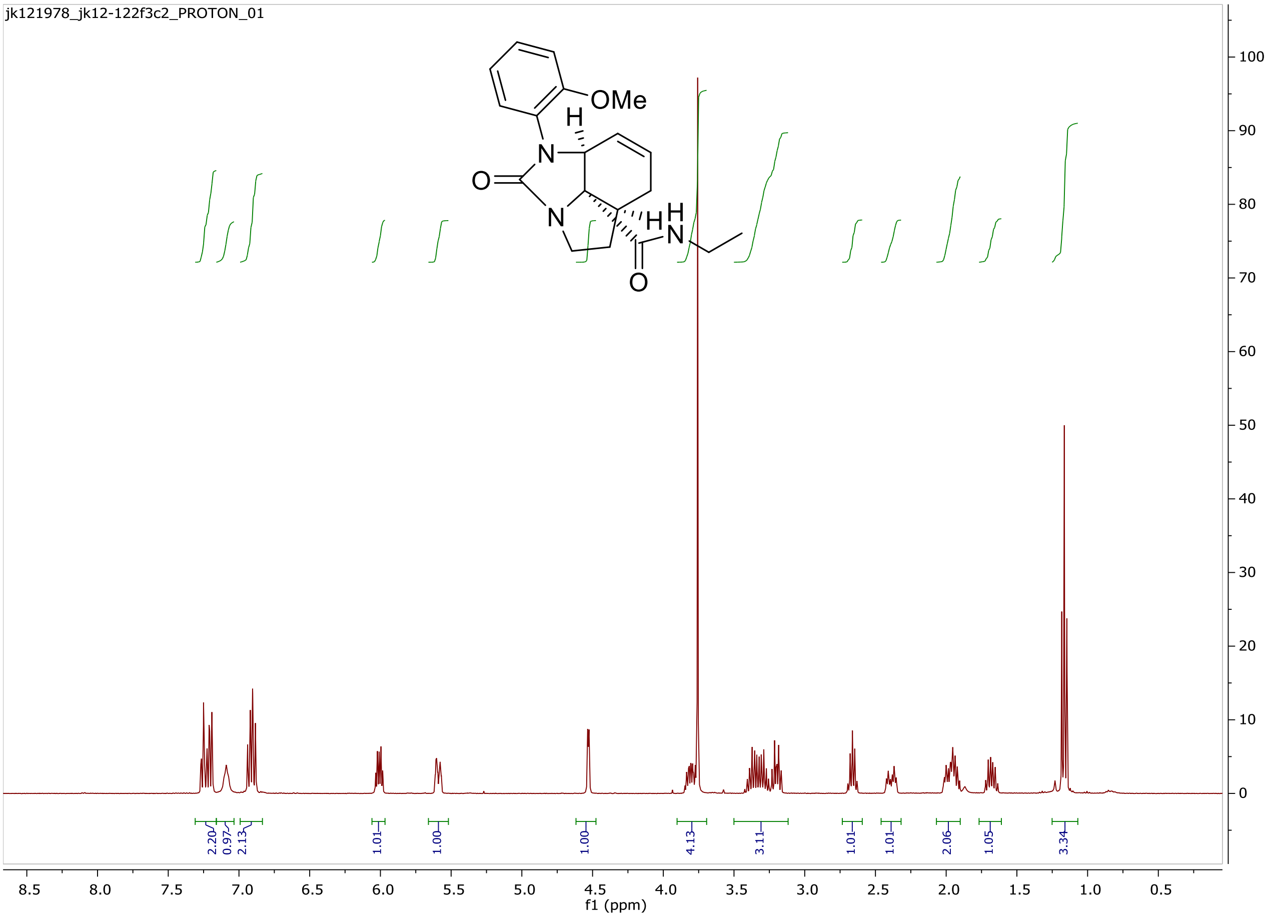

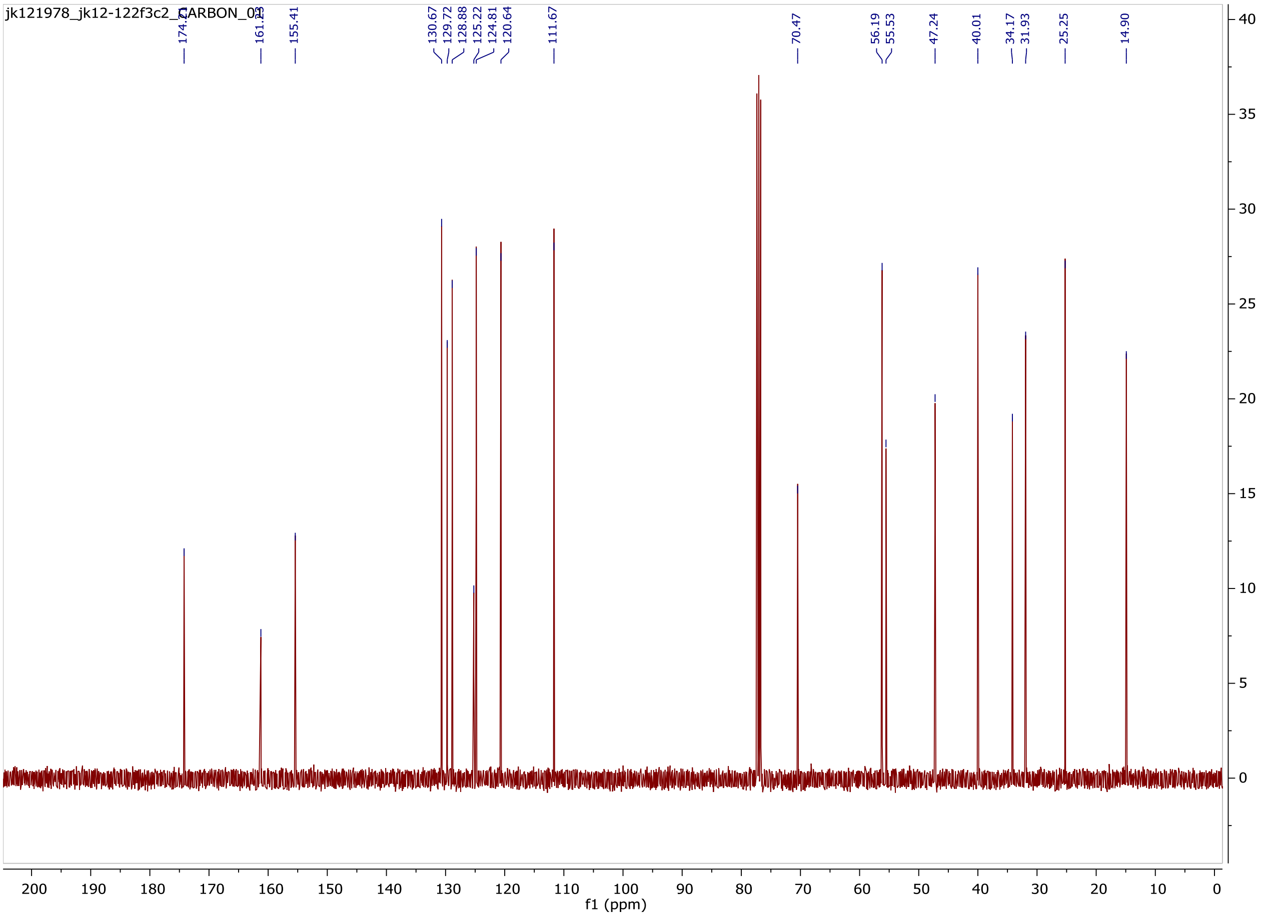

(±)-(31*R*,5a*S*,8a*S*)-1-(2,6-dichlorophenyl)-*N*-ethyl-2-oxo-1,4,5,5a,6,8a-hexahydroimidazo[4,5,1-hi]indole-31(2H)-carboxamide

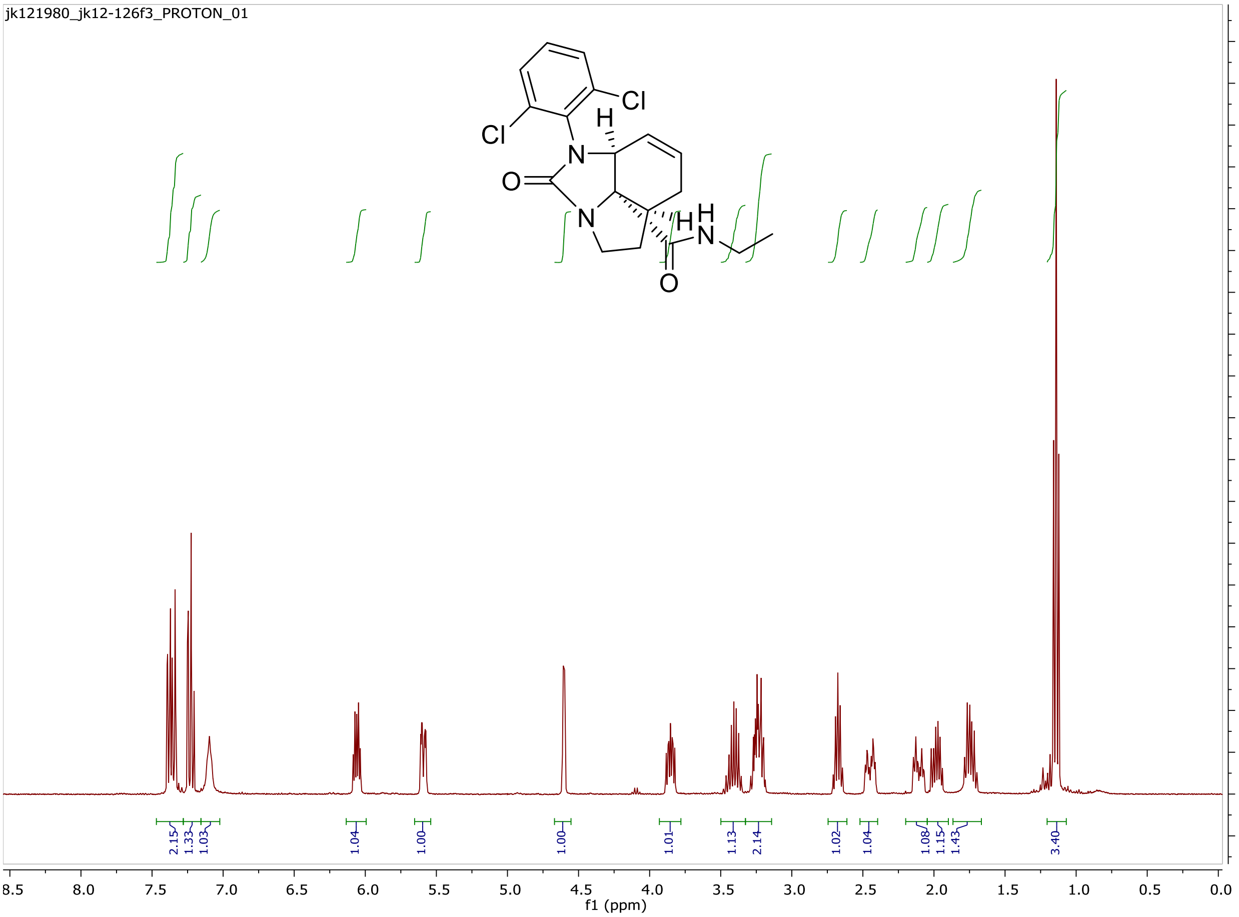

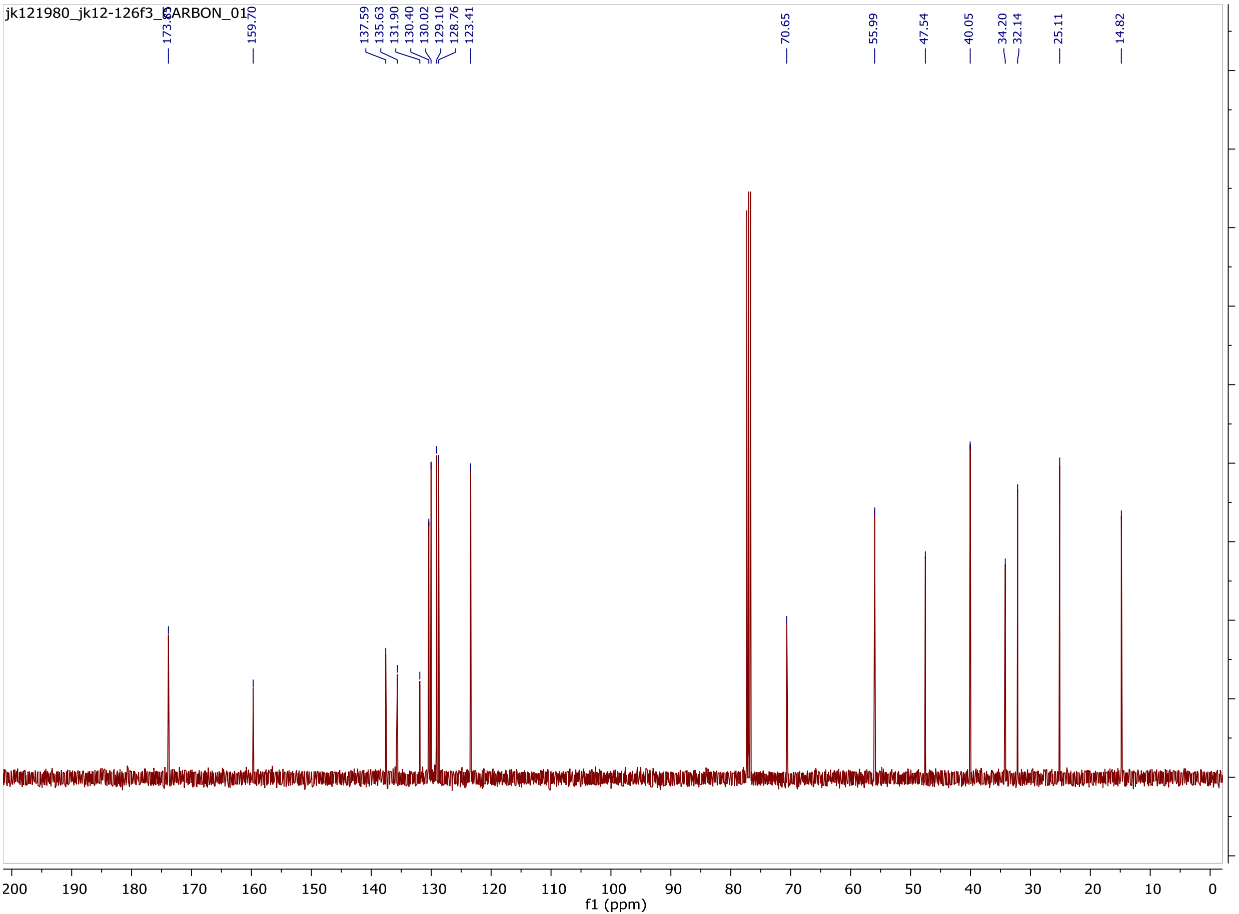

(±)-(31*R*,5a*S*,8a*S*,*Z*)-2-((2,6-dichlorophenyl)imino)-N-ethyl-8a-methyl-5,5a,6,8a-tetrahydro-2H-oxazolo[5,4,3-hi]indole-31(4H)-carboxamide

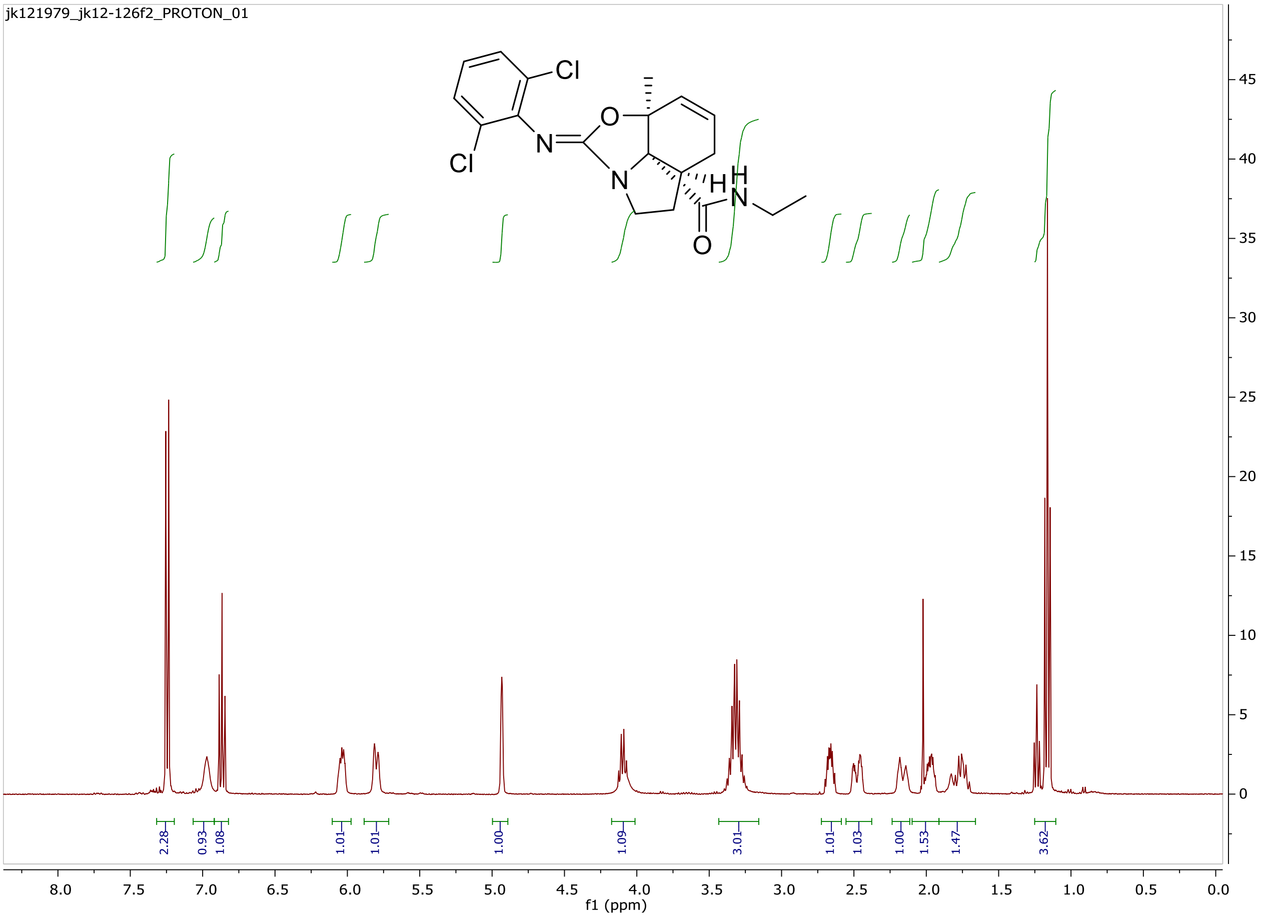

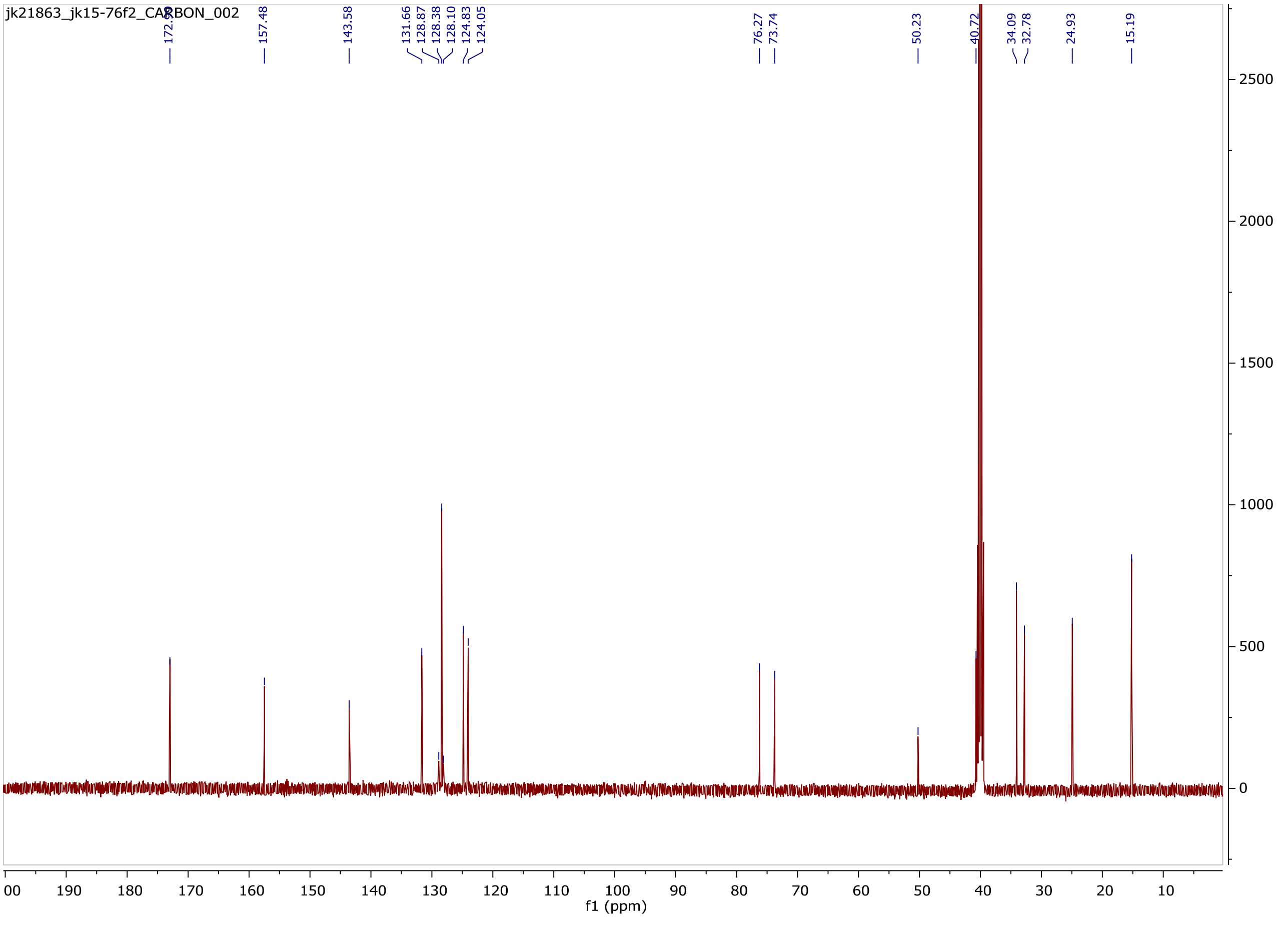

(±)-(31*R*,5a*S*,8a*S*,*Z*)-N-ethyl-2-(tosylimino)-5,5a,6,8a-tetrahydro-2H-oxazolo[5,4,3-hi]indole-31(4H)-carboxamide

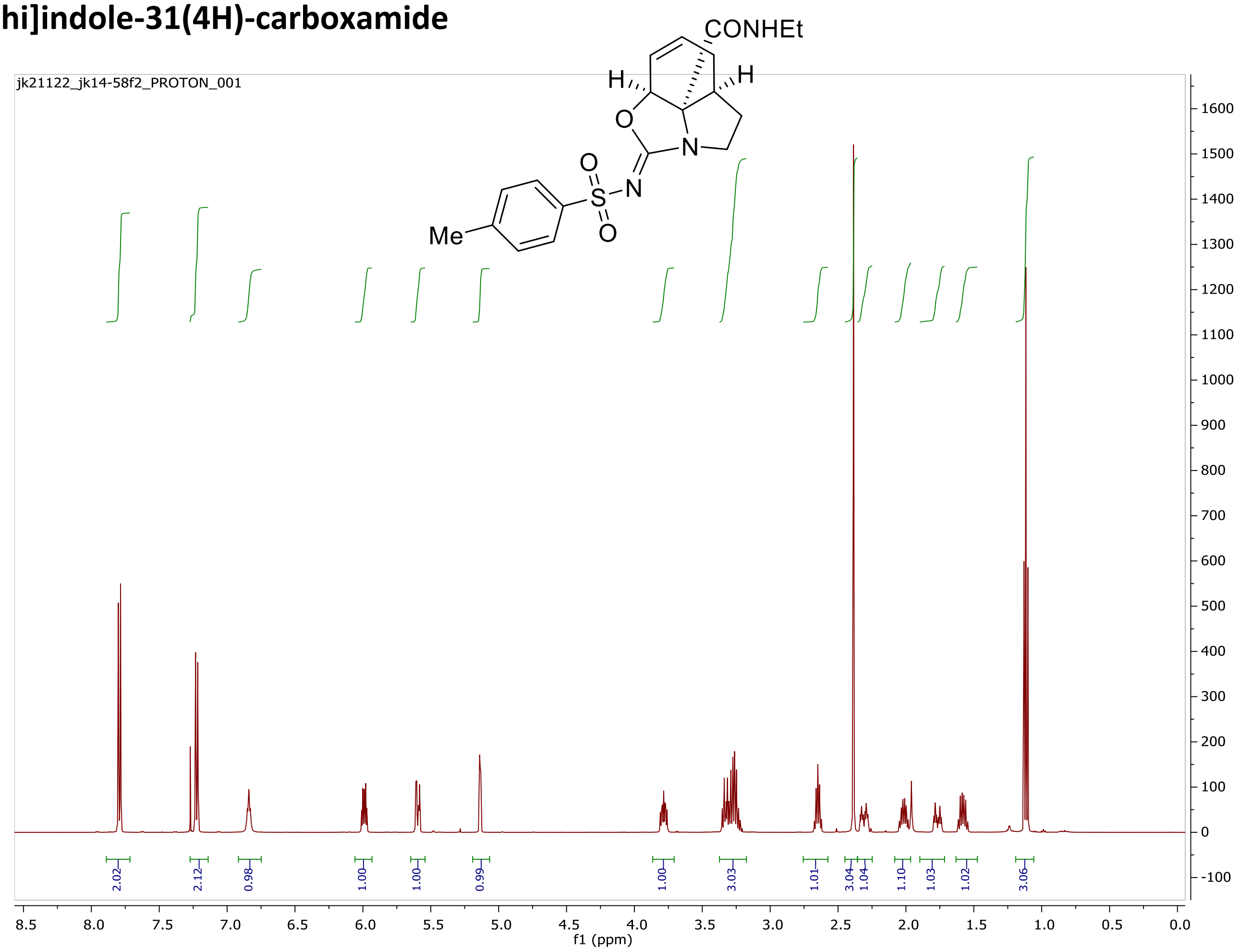

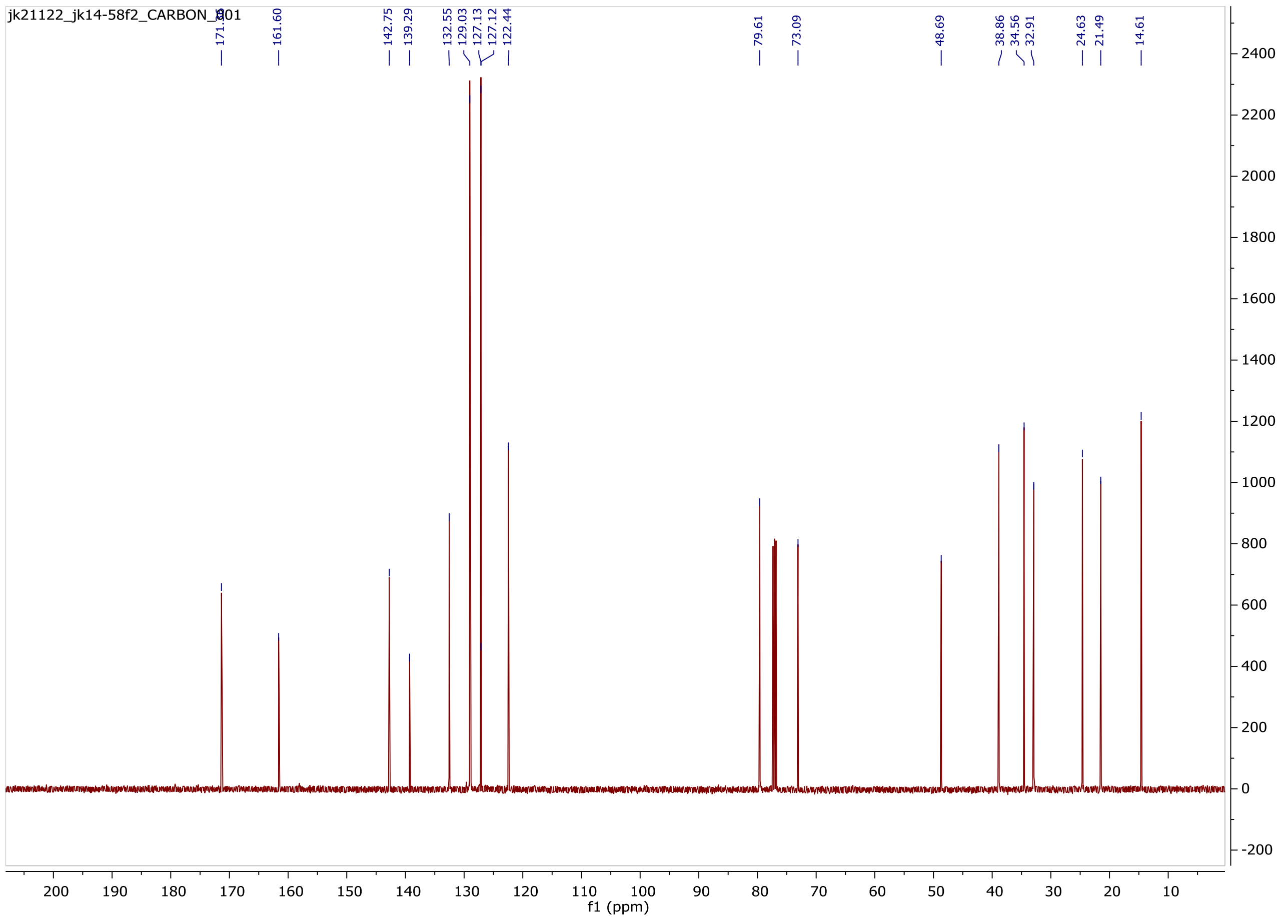

(±)-(31*R*,5a*S*,8a*S*,*Z*)-2-((2-chlorophenyl)imino)-N-ethyl-5,5a,6,8a-tetrahydro-2H-oxazolo[5,4,3-hi]indole-31(4H)-carboxamide

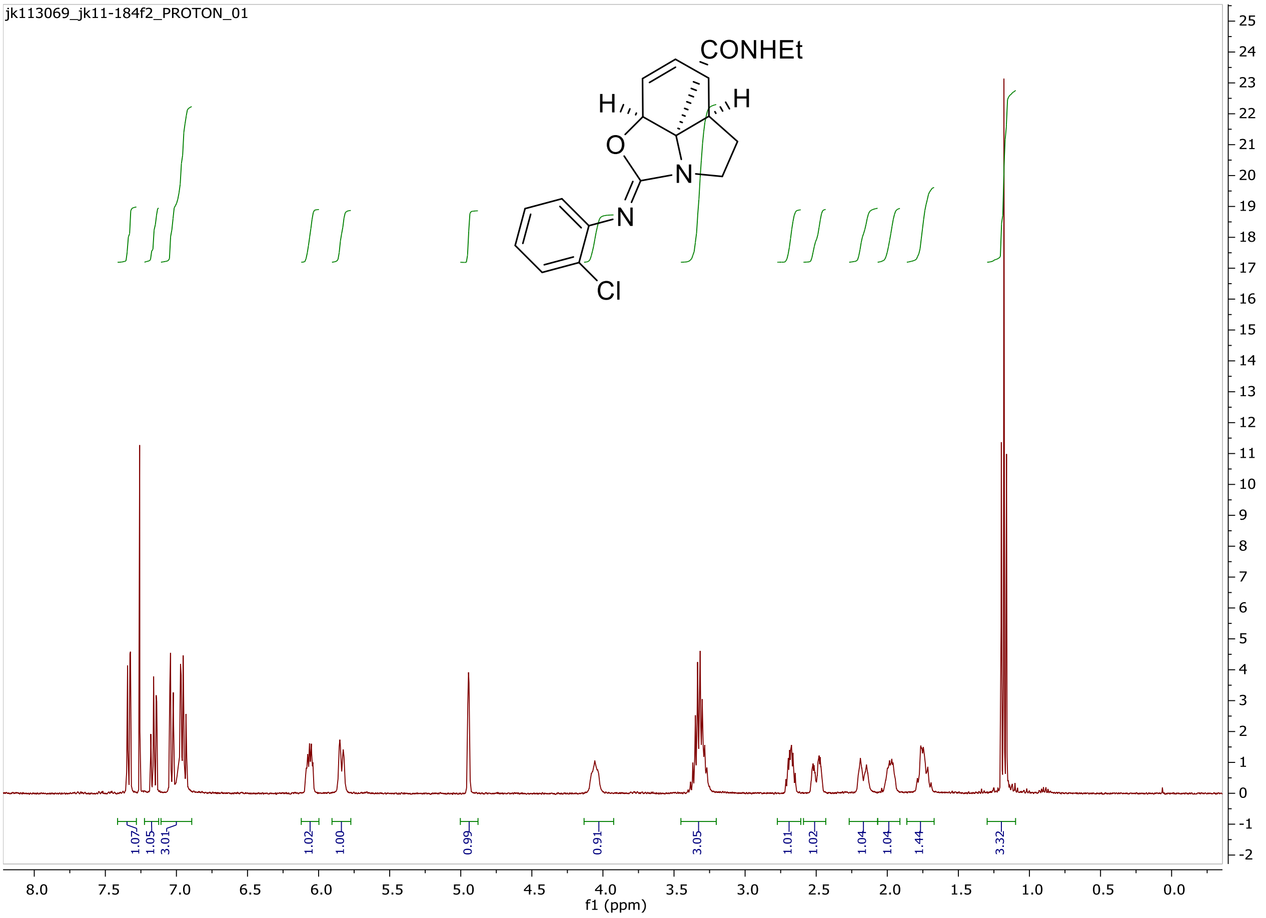

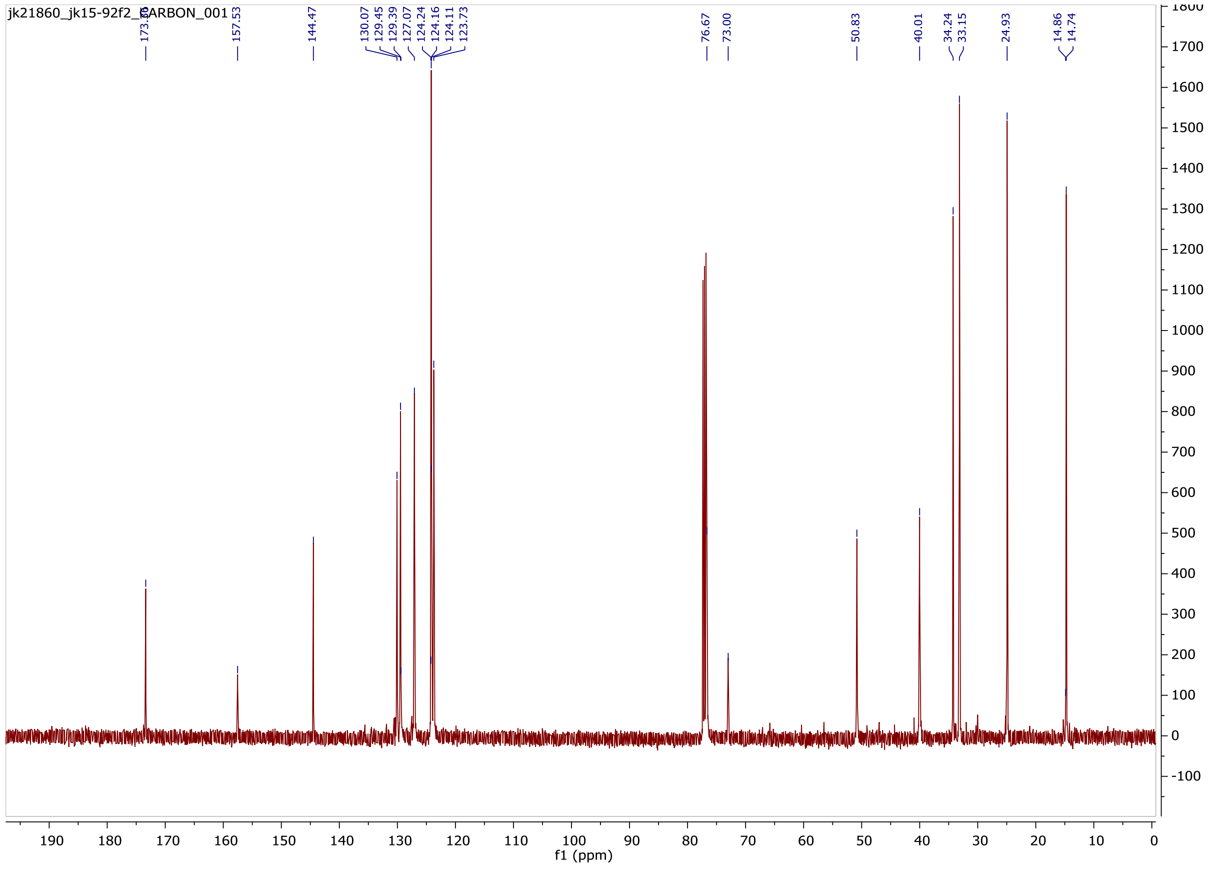

(±)-(31*R*,5a*S*,8a*S*)-N-ethyl-1-(4-nitrophenyl)-2-oxo-1,4,5,5a,6,8a-hexahydroimidazo[4,5,1-hi]indole-31(2H)-carboxamide

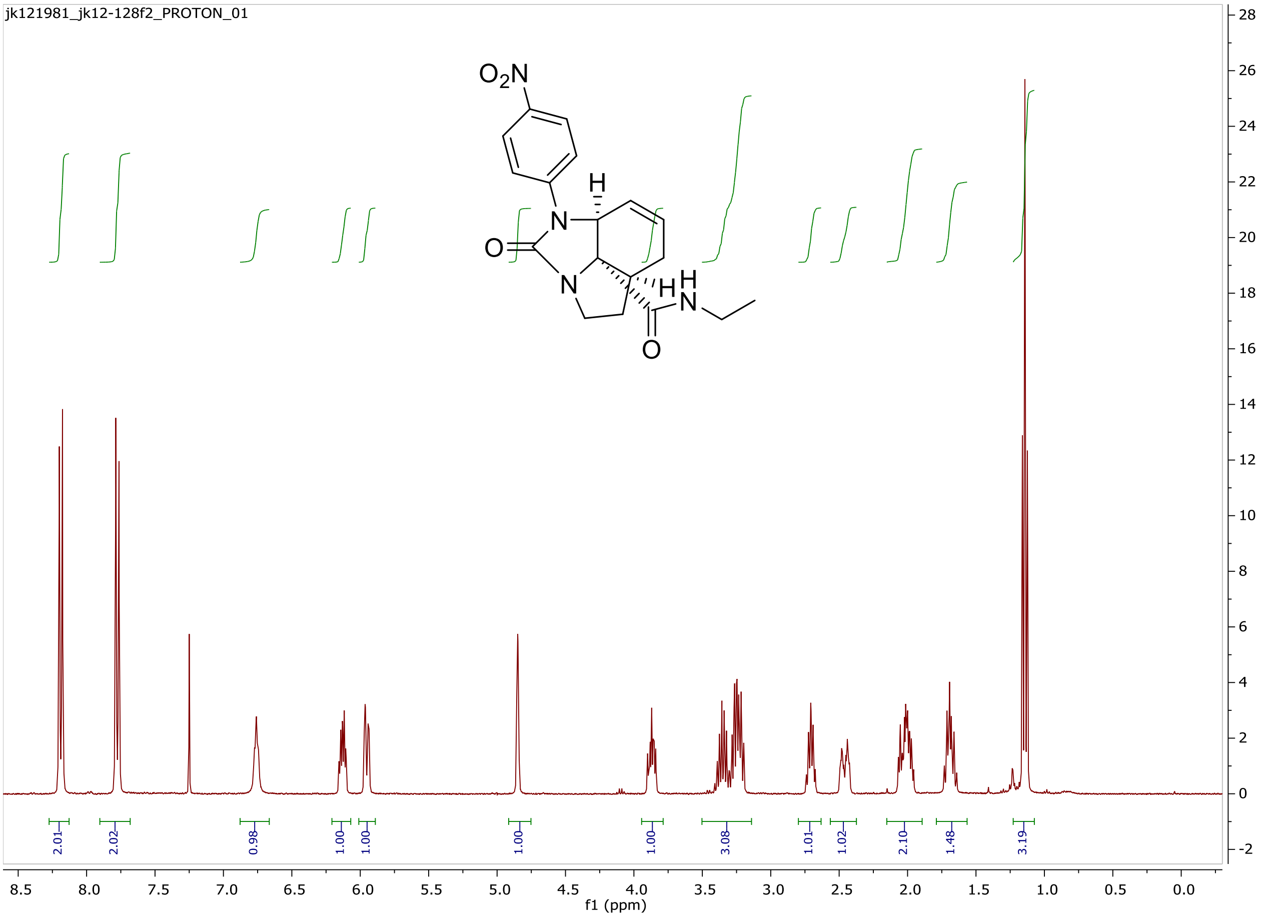

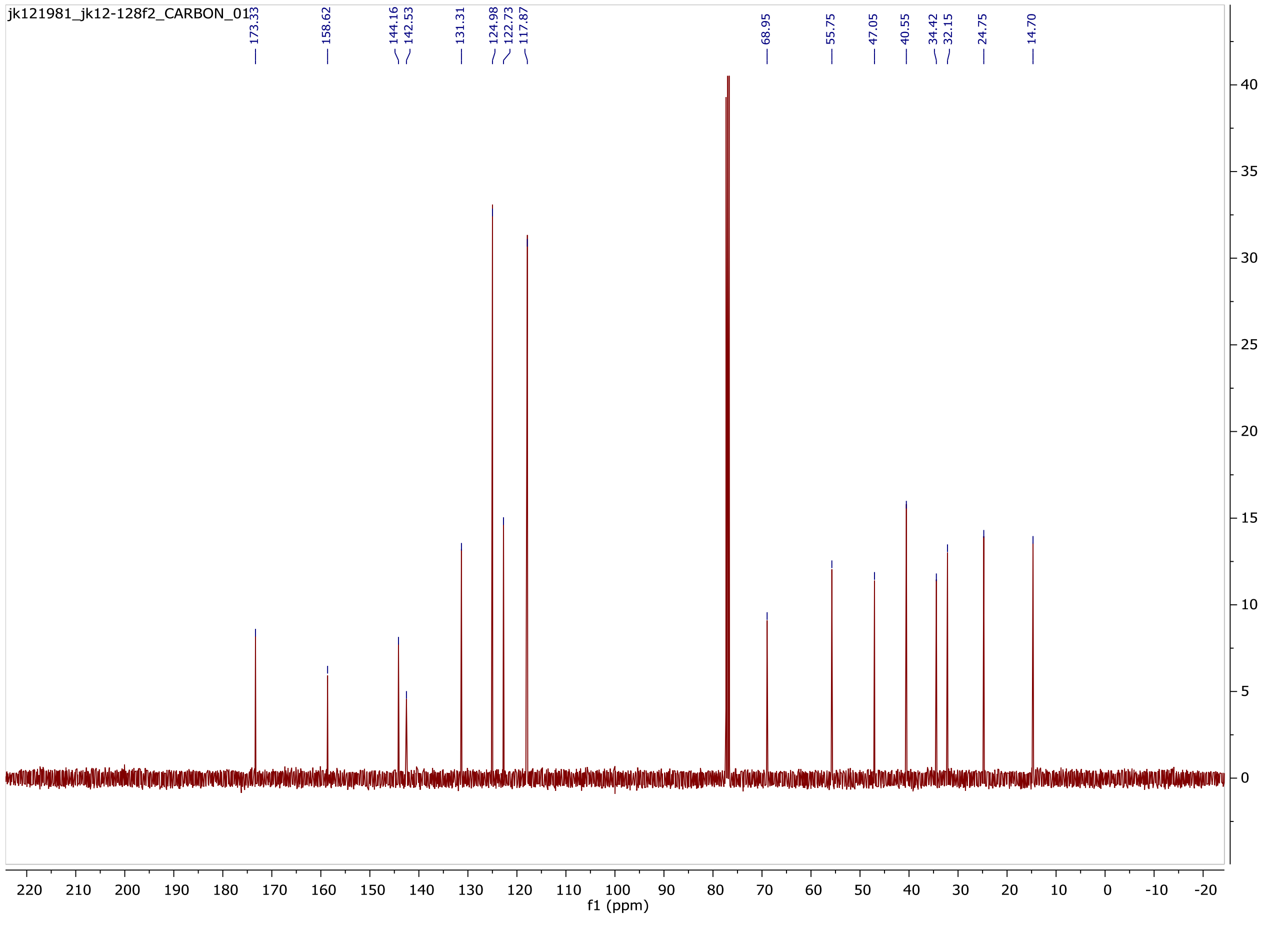

(±)-(3*R*,5a*S*)-1,1-Dicyano-*N*-ethyl-2-phenyl-1,4,5,5a,6,8a-hexahydropyrrolo [3,2,1-hi]indole-3(2*H*)-carboxamide 10a

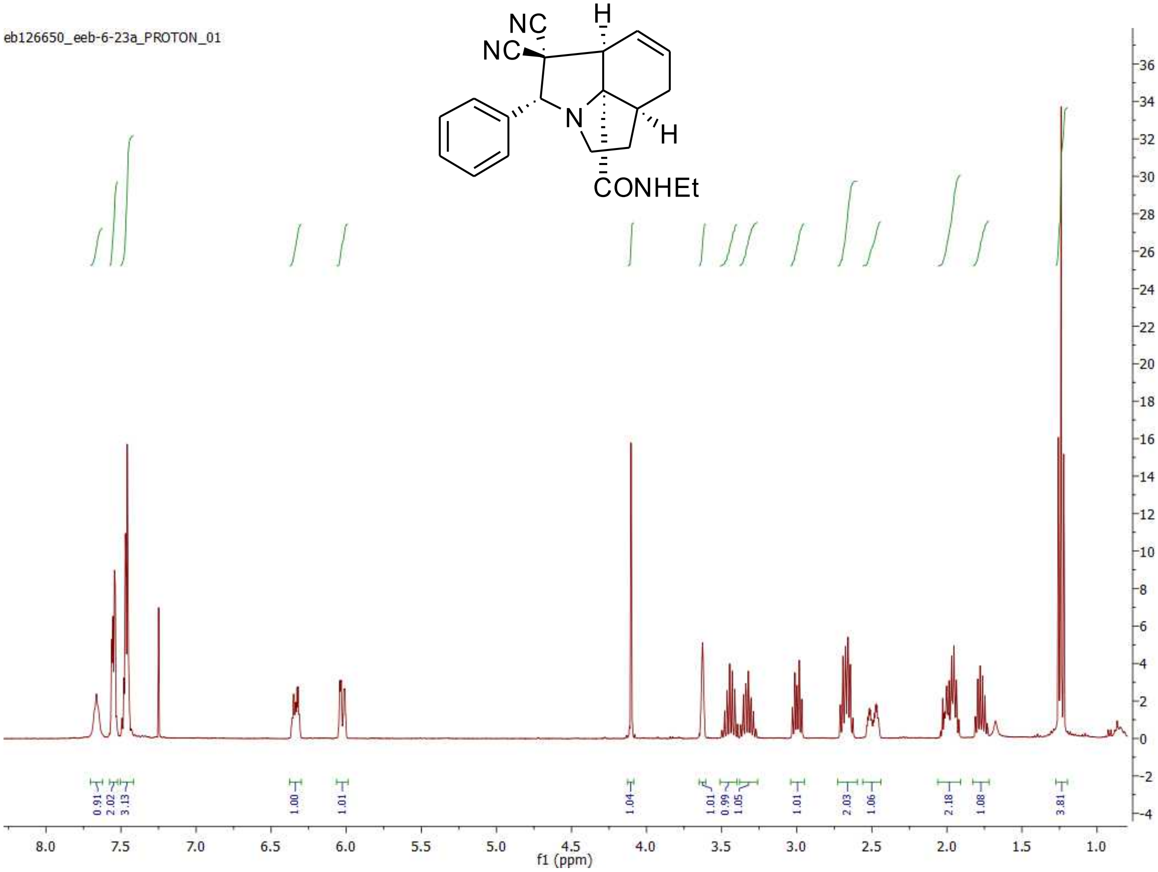

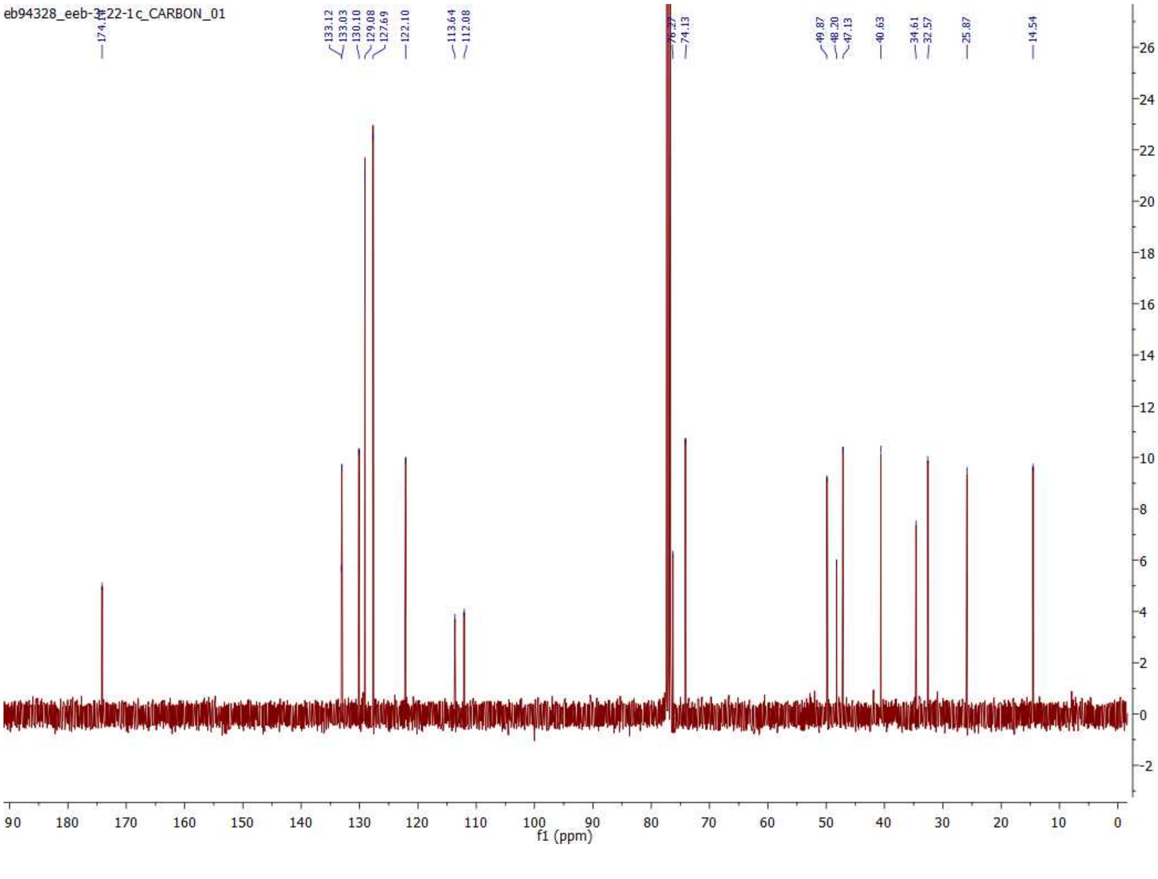

Di-*tert*-butyl 2-methylenemalonate

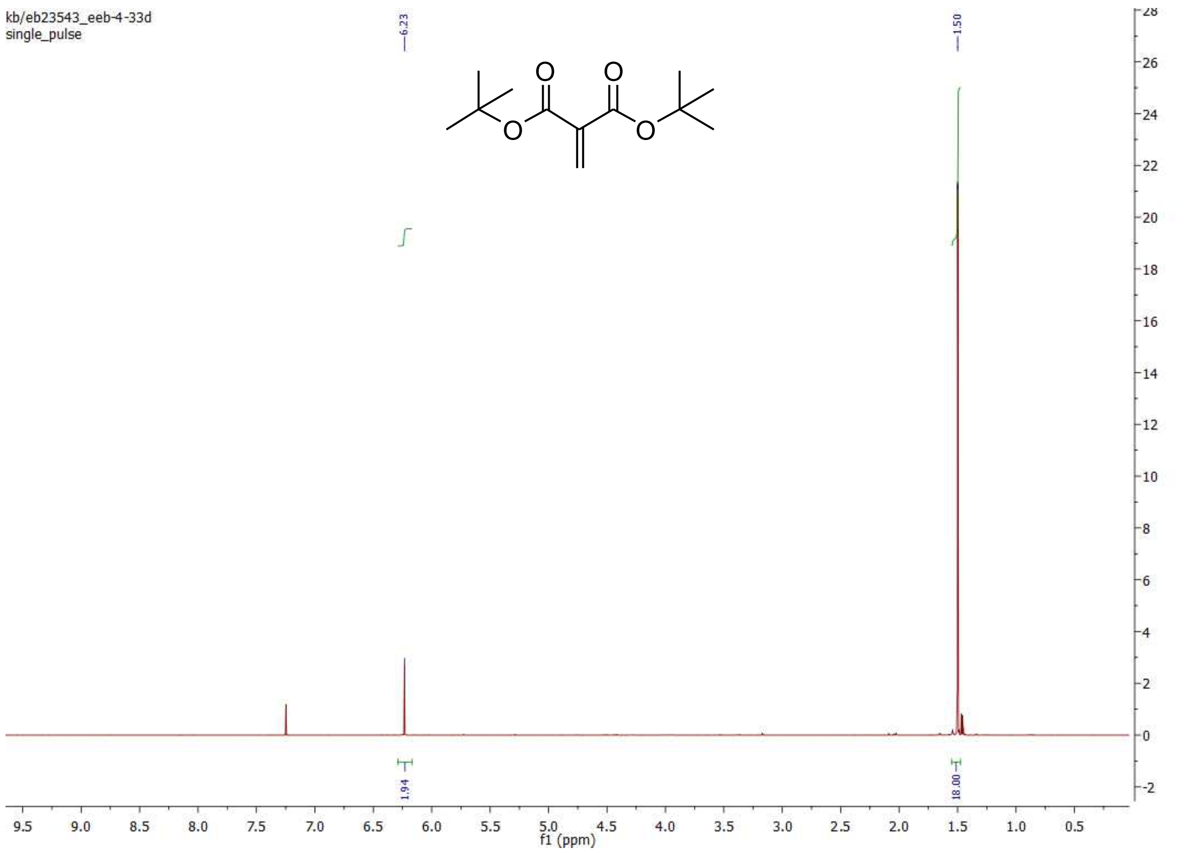

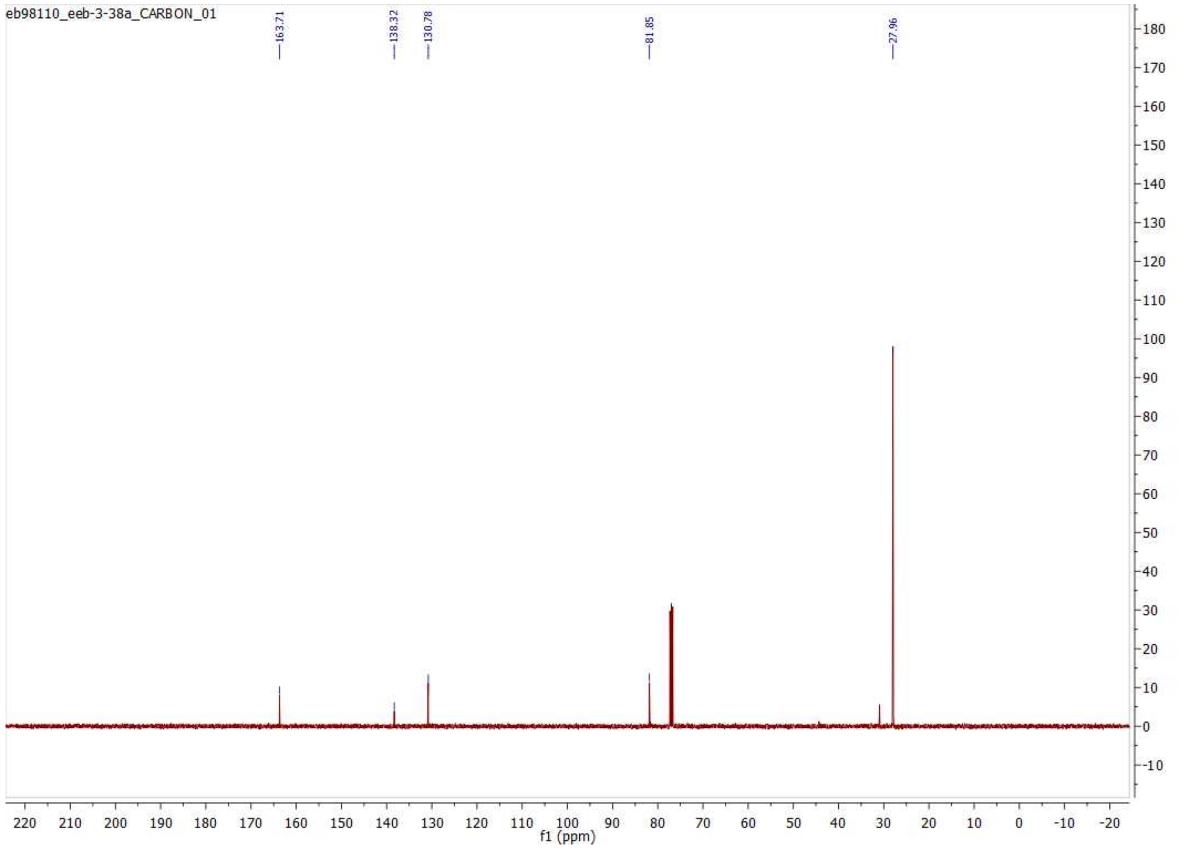

(±)-1-(*Tert*-butyl) 1-methyl (3*R*,8a*R*)-3-(ethylcarbamoyl)-3,4,5,5a,6,8a-hexahydropyrrolo[3,2,1-hi]indole-1,1(2*H*)-dicarboxylate 11a

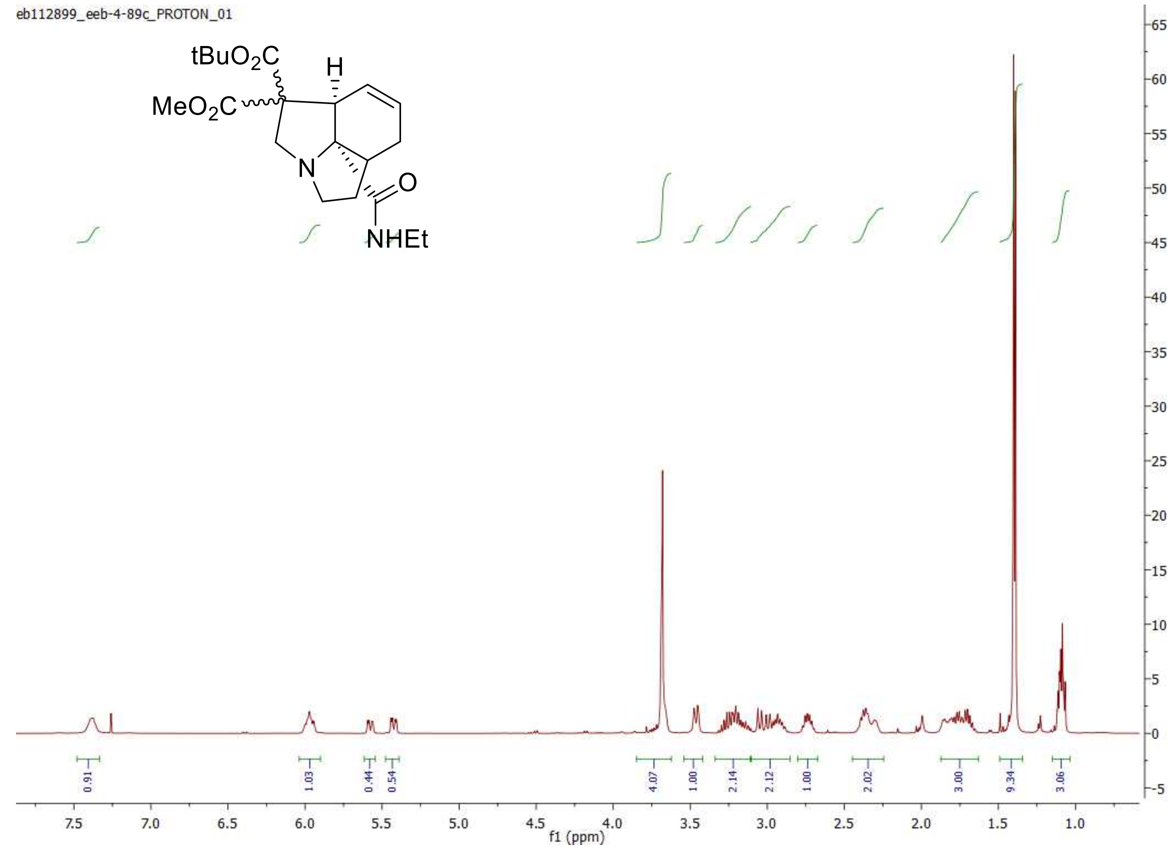

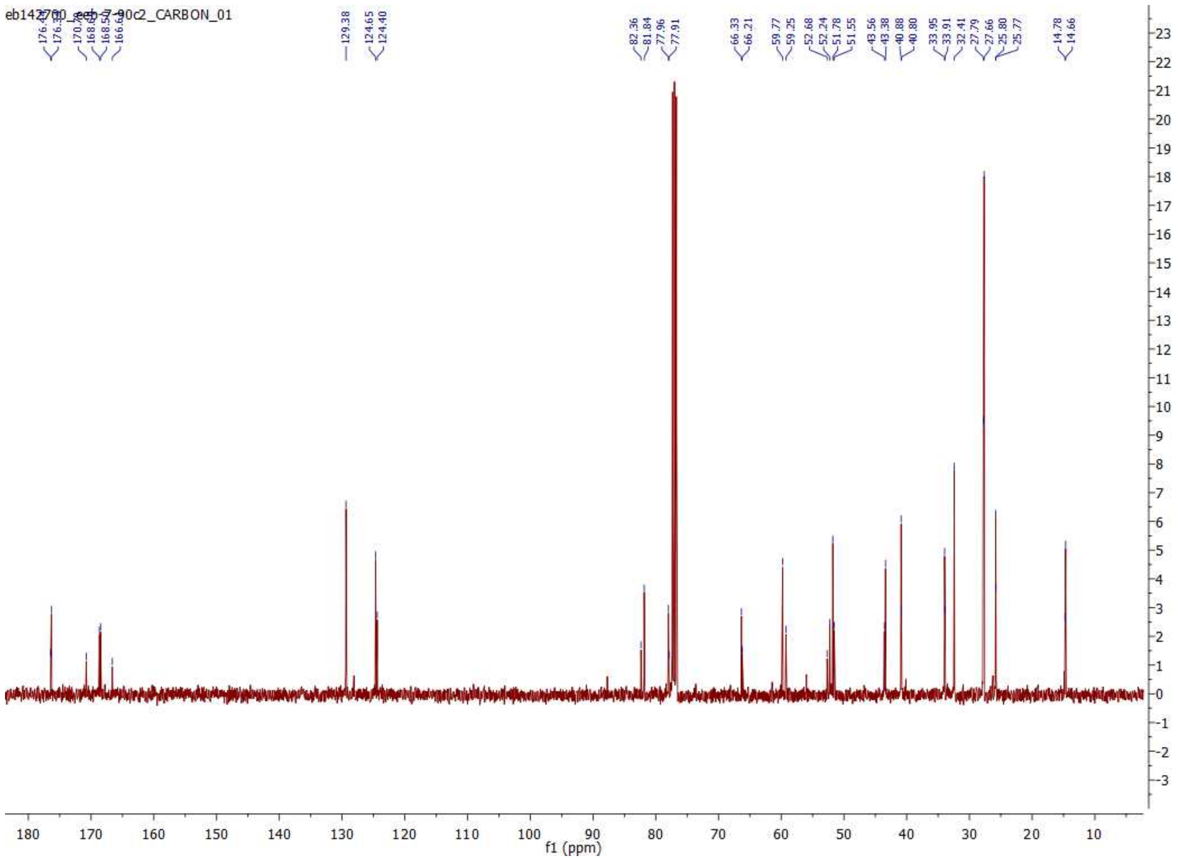

(±)-Di-*tert*-butyl (3*R*,8a*R*)-3-(ethylcarbamoyl)-3,4,5,5a,6,8a-hexahydropyrrolo[3,2,1-hi]indole-1,1(2*H*)-dicarboxylate 12a

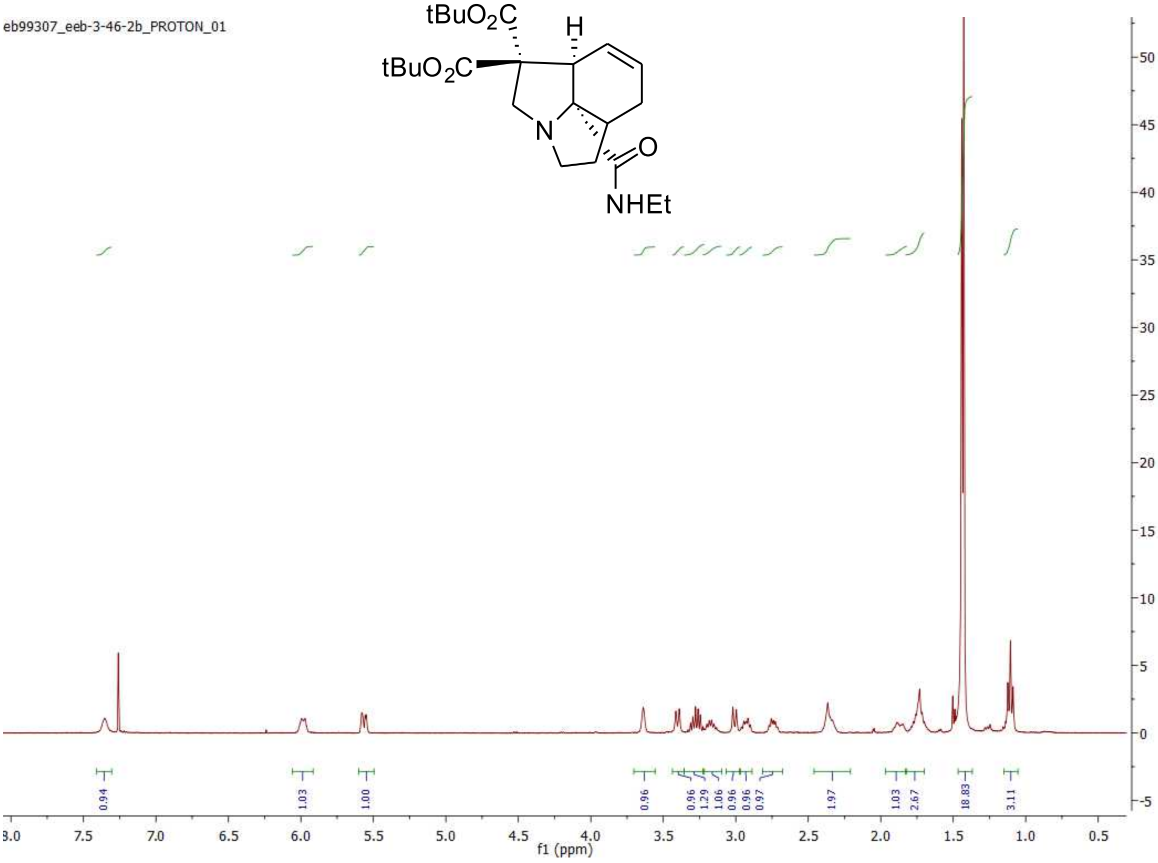

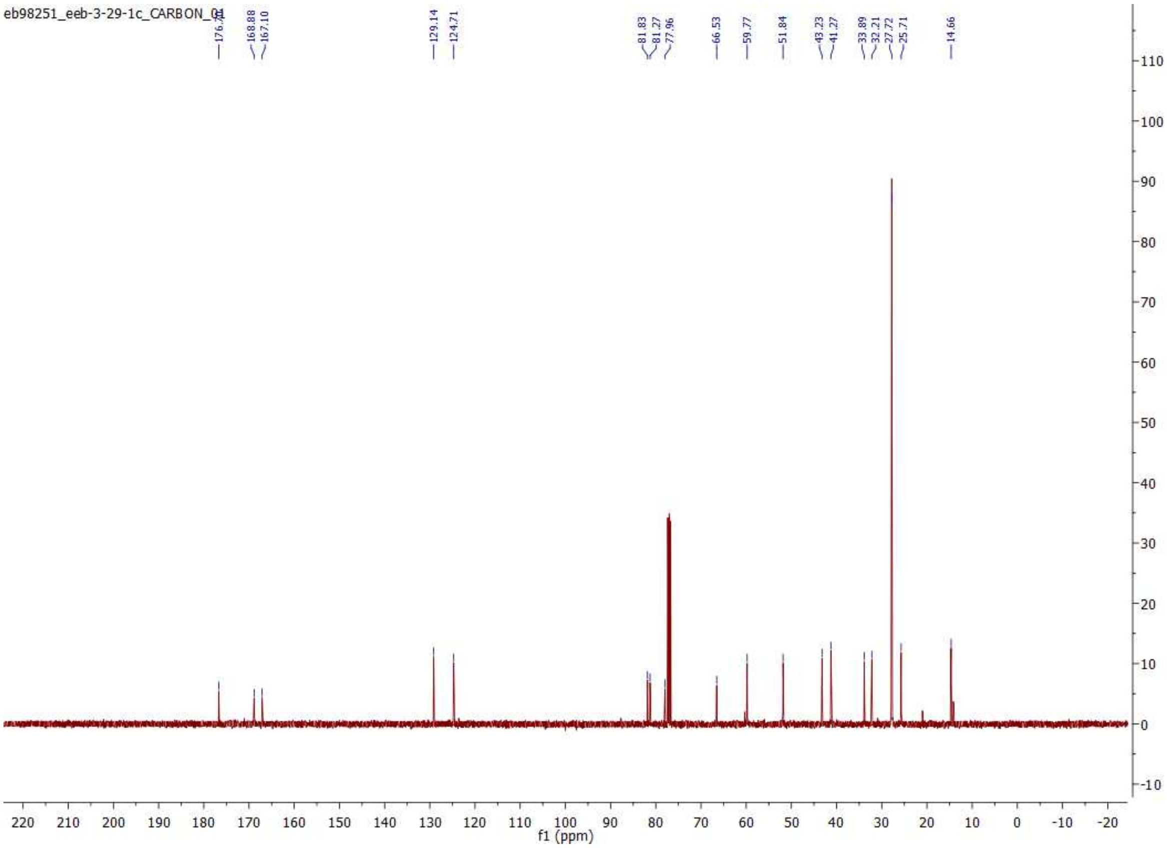

(±)-Di-*tert*-butyl (3*R*,8a*R*)-3-acetyl-3,4,5,5a,6,8a-hexahydropyrrolo[3,2,1-hi]indole-1,1(2*H*)-dicarboxylate 12b

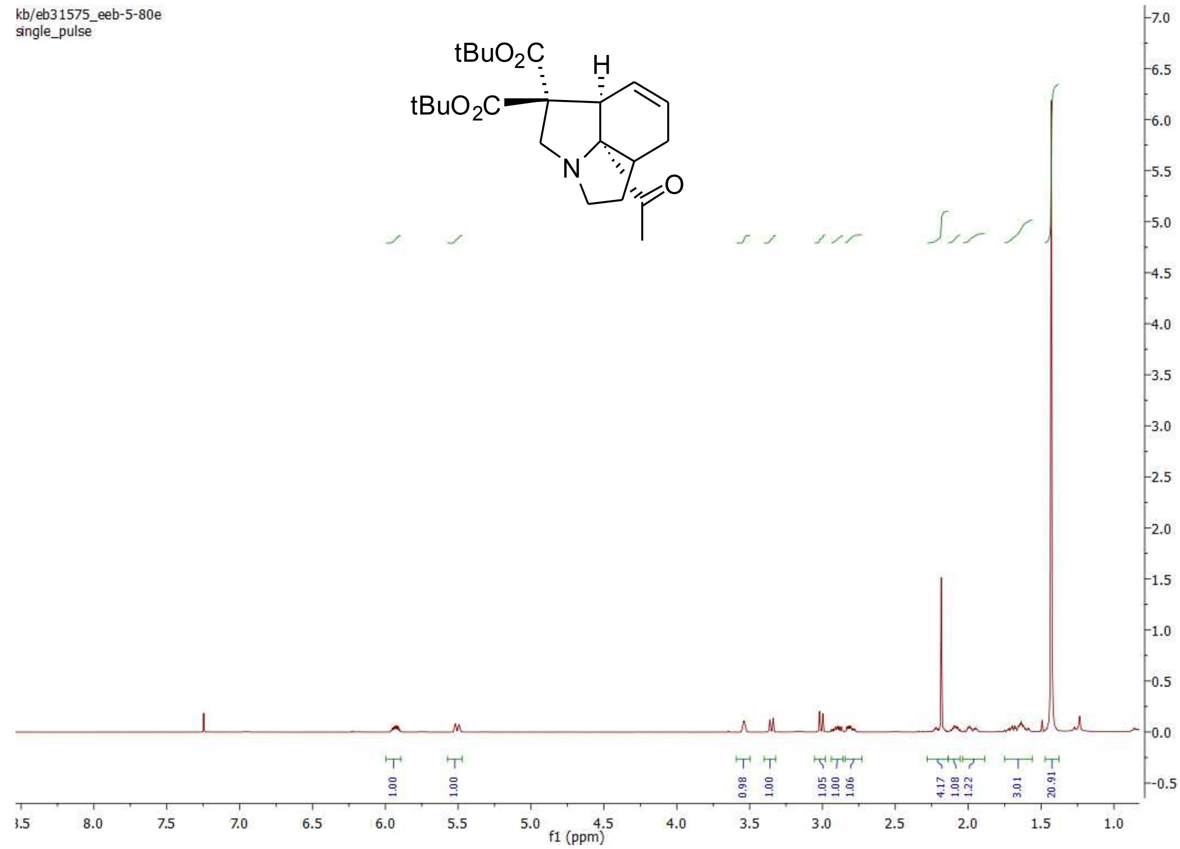

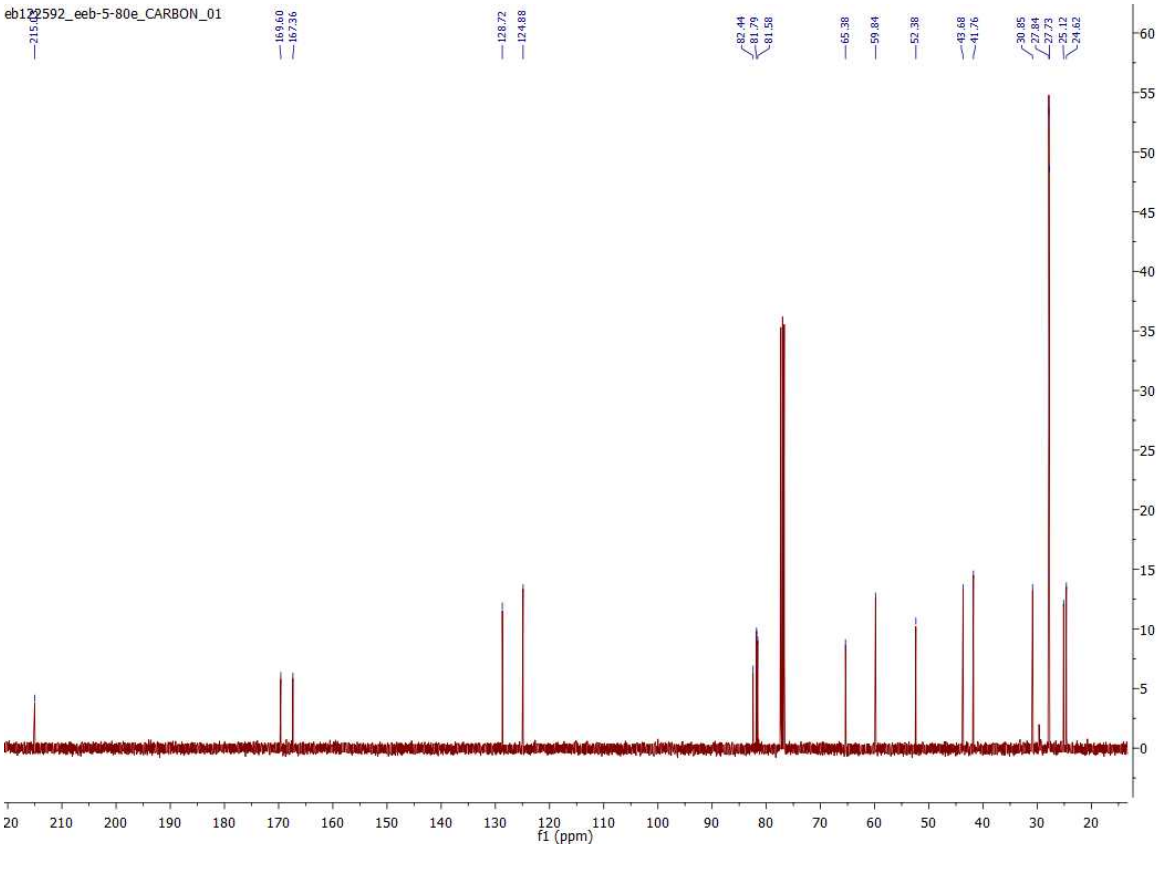

(±)-(3*R*,8a*S*)-*N*-ethyl-5,5a,6,8a-tetrahydro-2H-oxazolo[5,4,3-hi]indole-3(4*H*)-carboxamide 13a

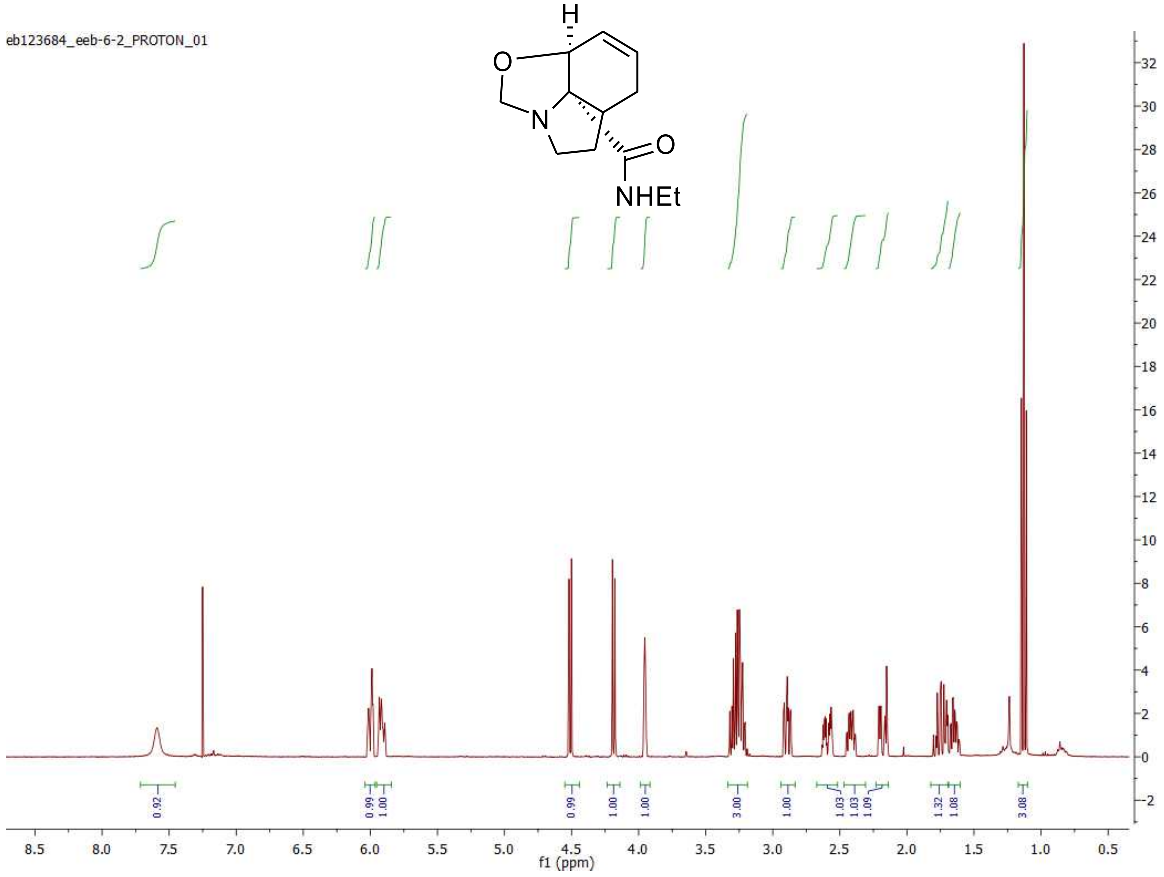

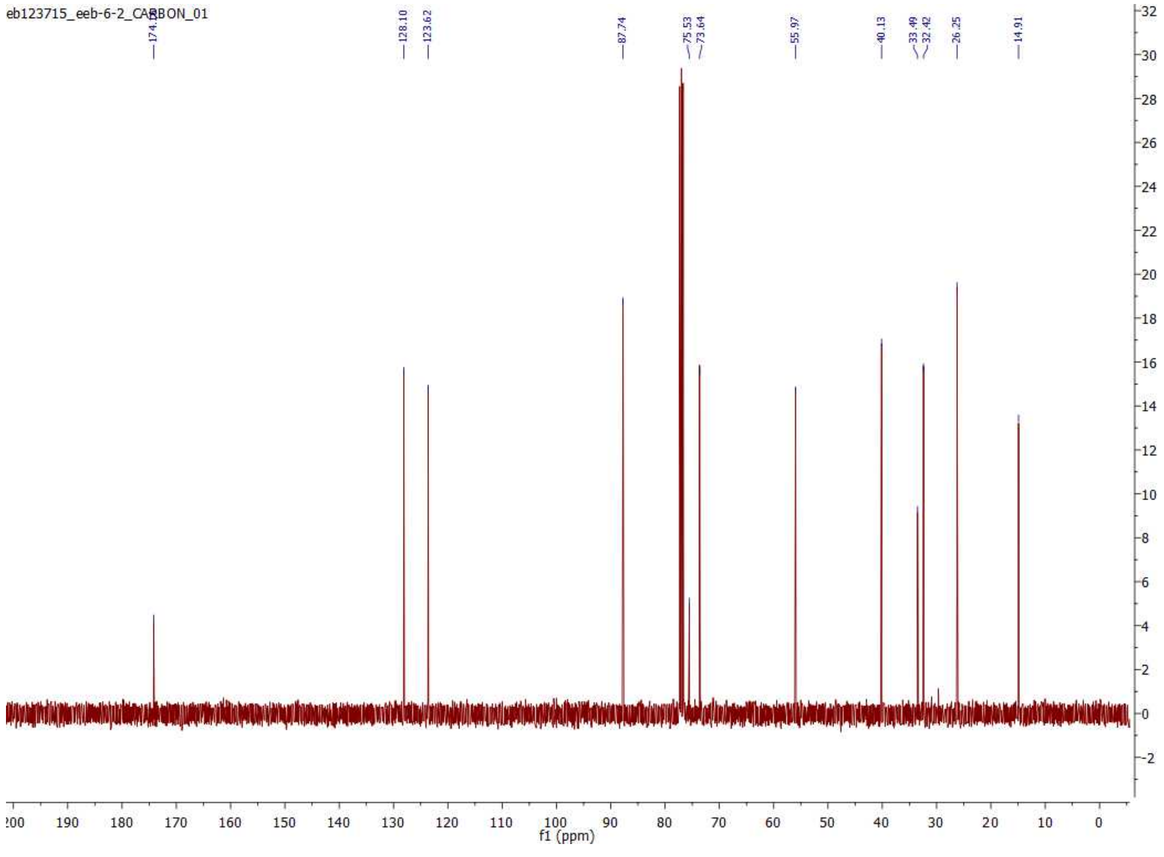

(±)-1-((3*R*,8a*S*)-5,5a,6,8a-Tetrahydro-2*H*-oxazolo[5,4,3-hi]indol-3(4*H*)-yl)ethan-1-one 13b

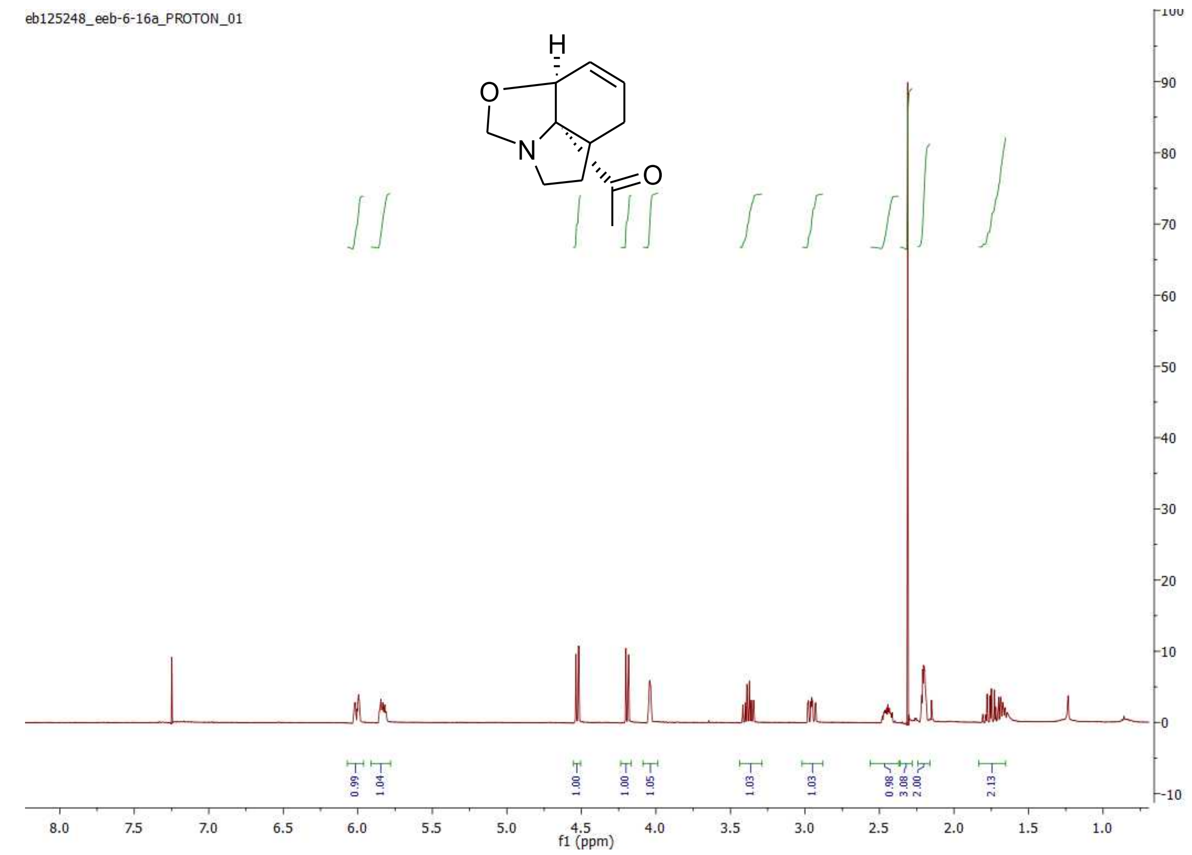

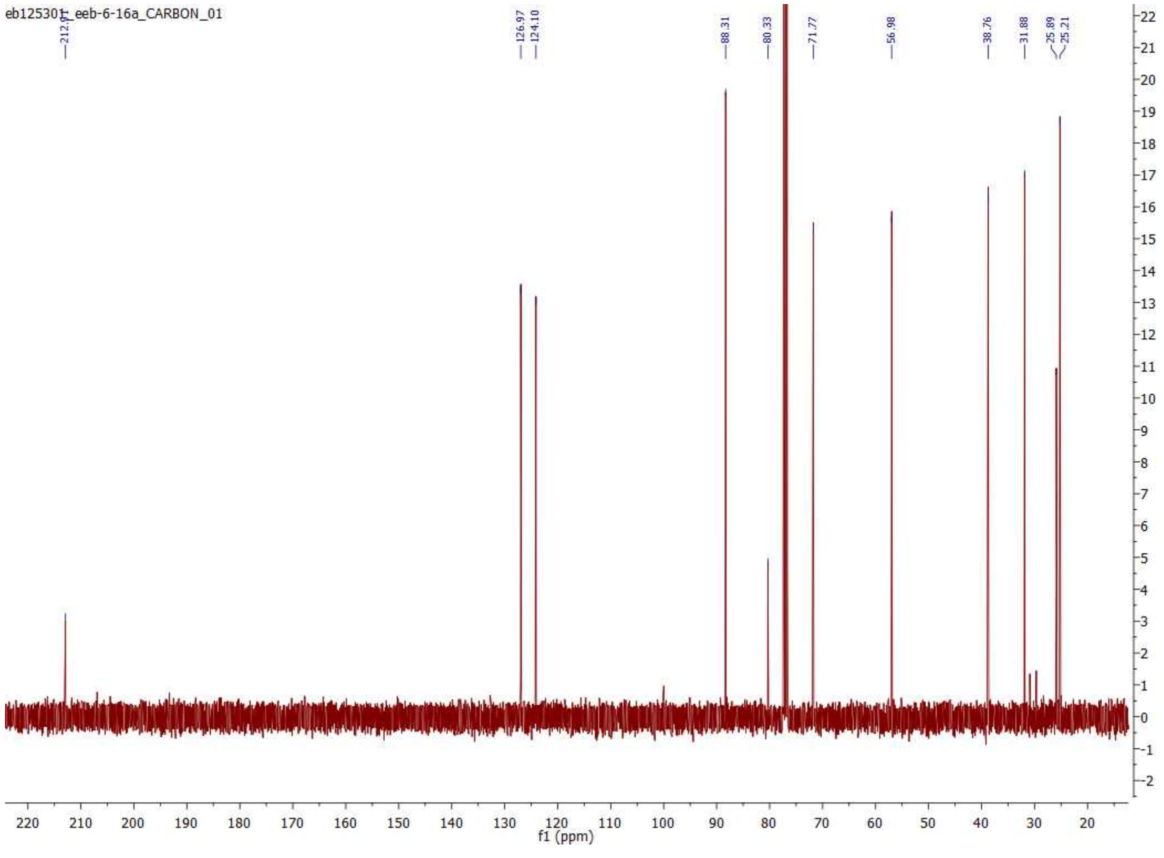

(±)-(2*S*,31*R*,8a*S*)-*N*-ethyl-2-methyl-5,5a,6,8a-tetrahydro-2*H*-oxazolo[5,4,3-hi]indole-3(4*H*)-carboxamide –14a

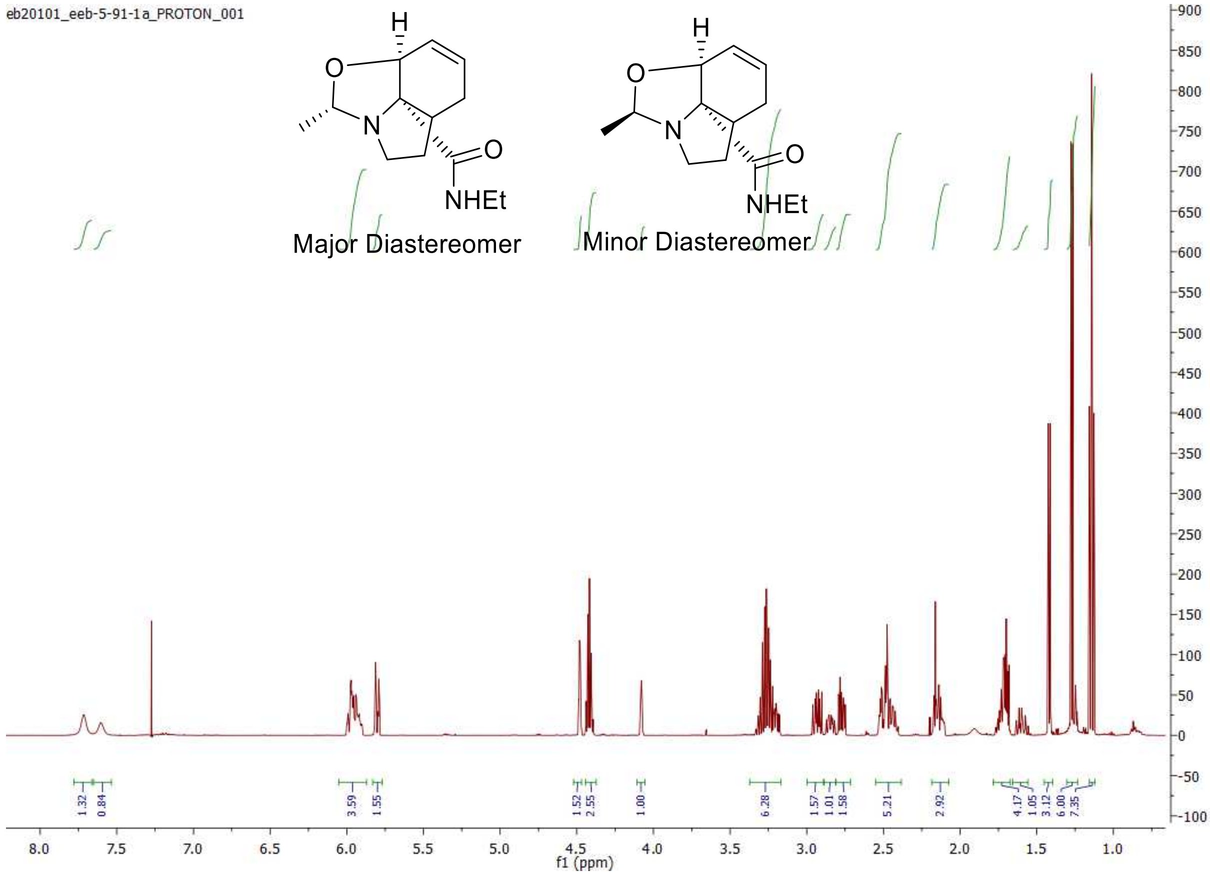

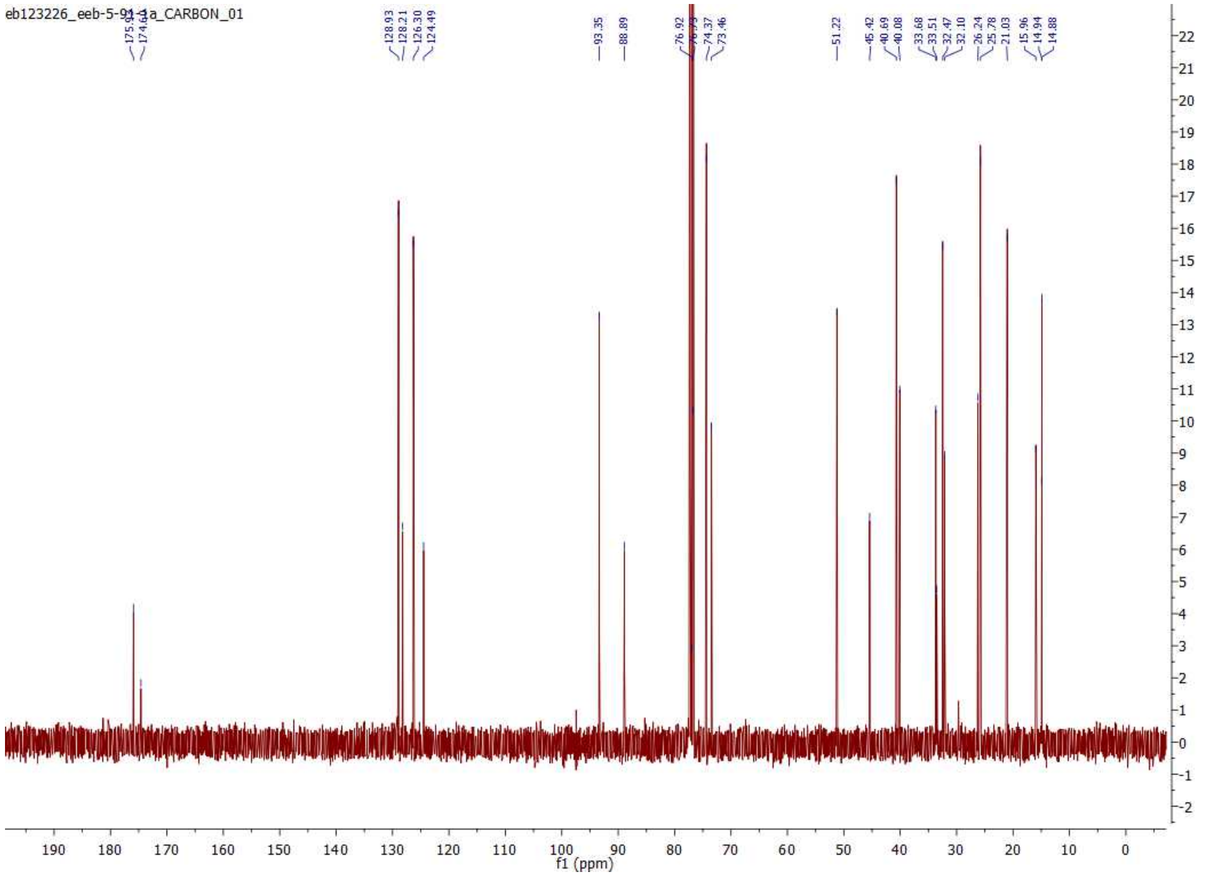

(±)-(2*S*,31*R*,8a*S*)-*N*-ethyl-2-methyl-5,5a,6,8a-tetrahydro-2*H*-oxazolo[5,4,3-hi]indole-3(4*H*)-carboxamide –14a

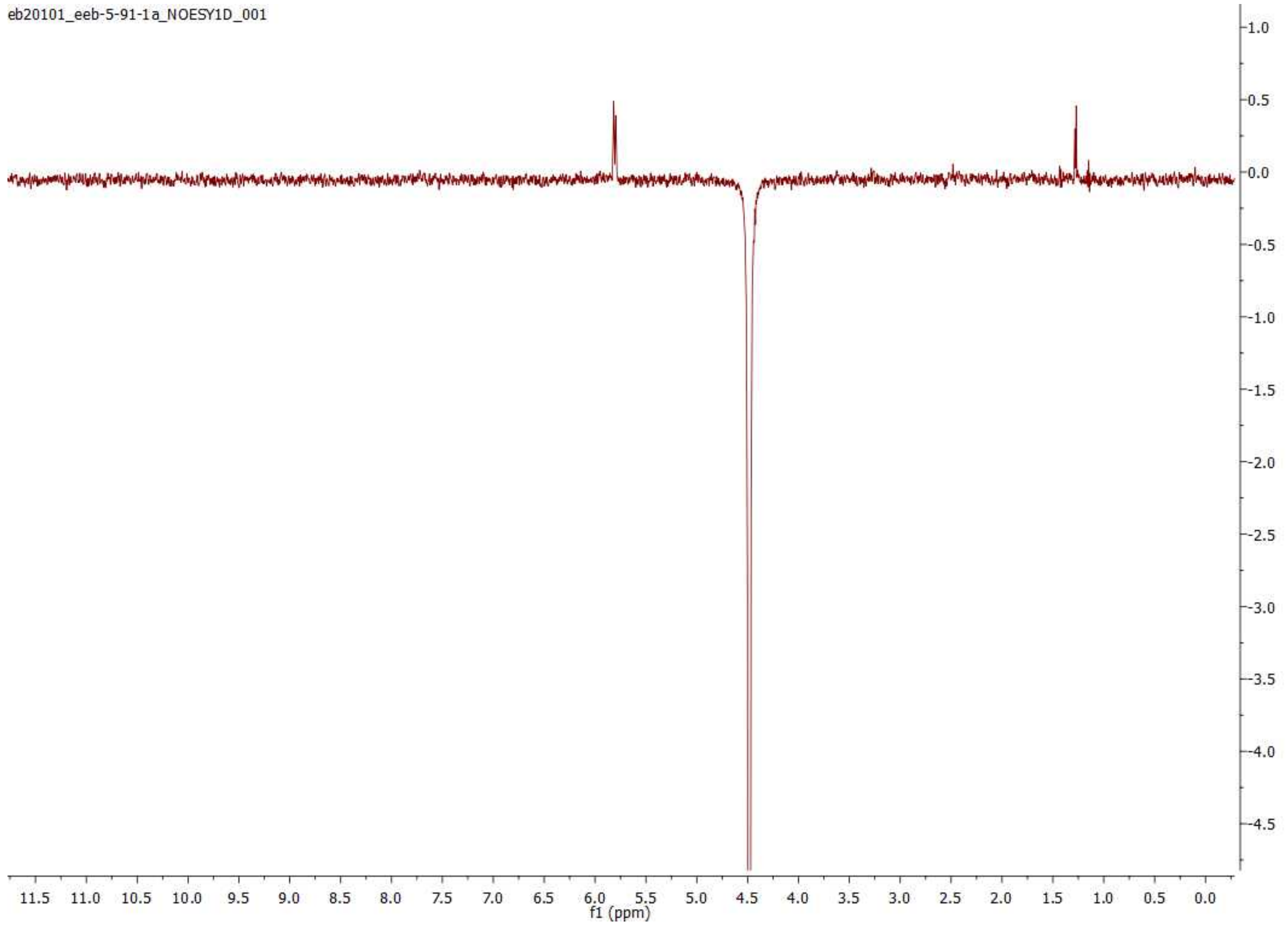

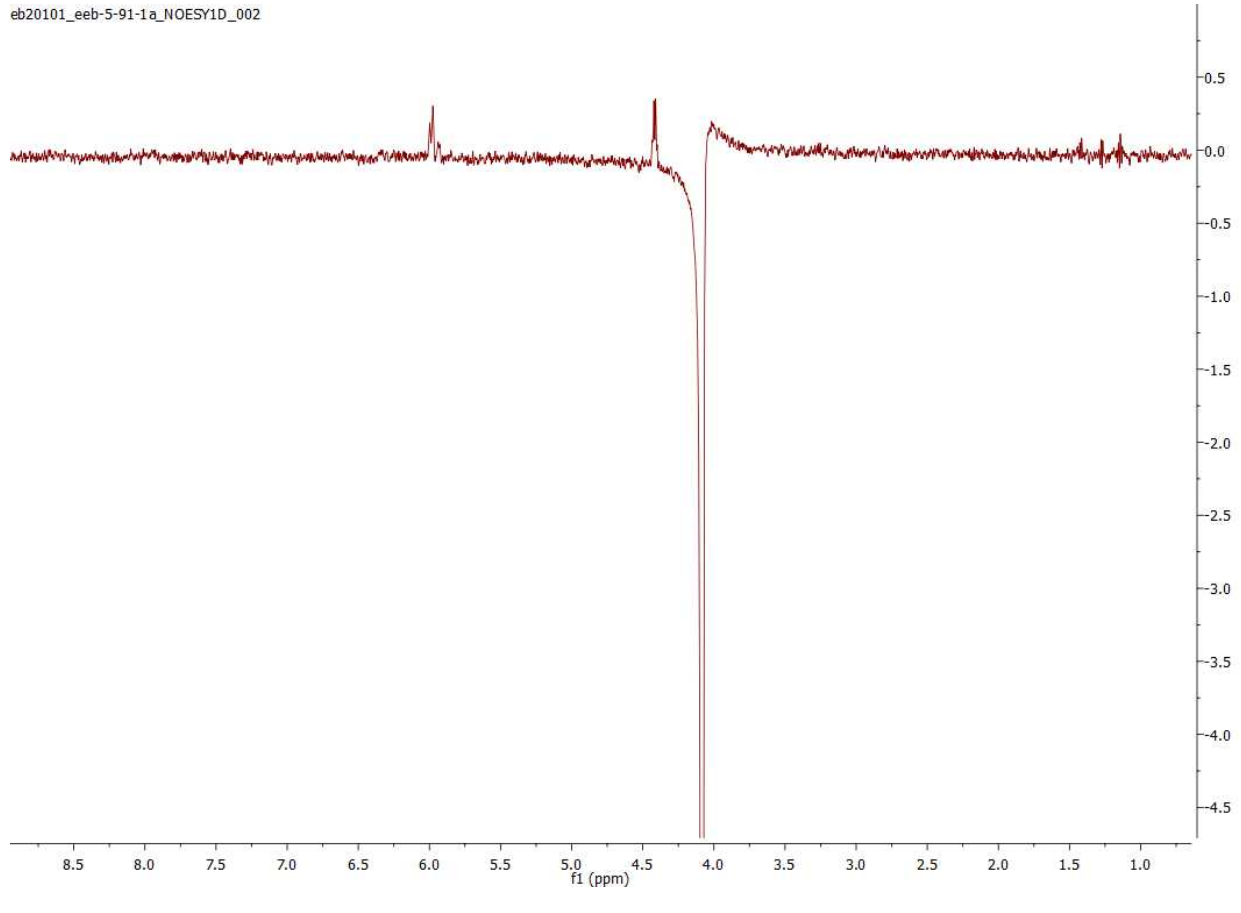

(±)-1-((3*R*,8a*S*)-2-Methyl-5,5a,6,8a-tetrahydro-2*H*-oxazolo[5,4,3-hi]indol-3(4*H*)-yl)ethan-1-one- Major Diastereomer 14b

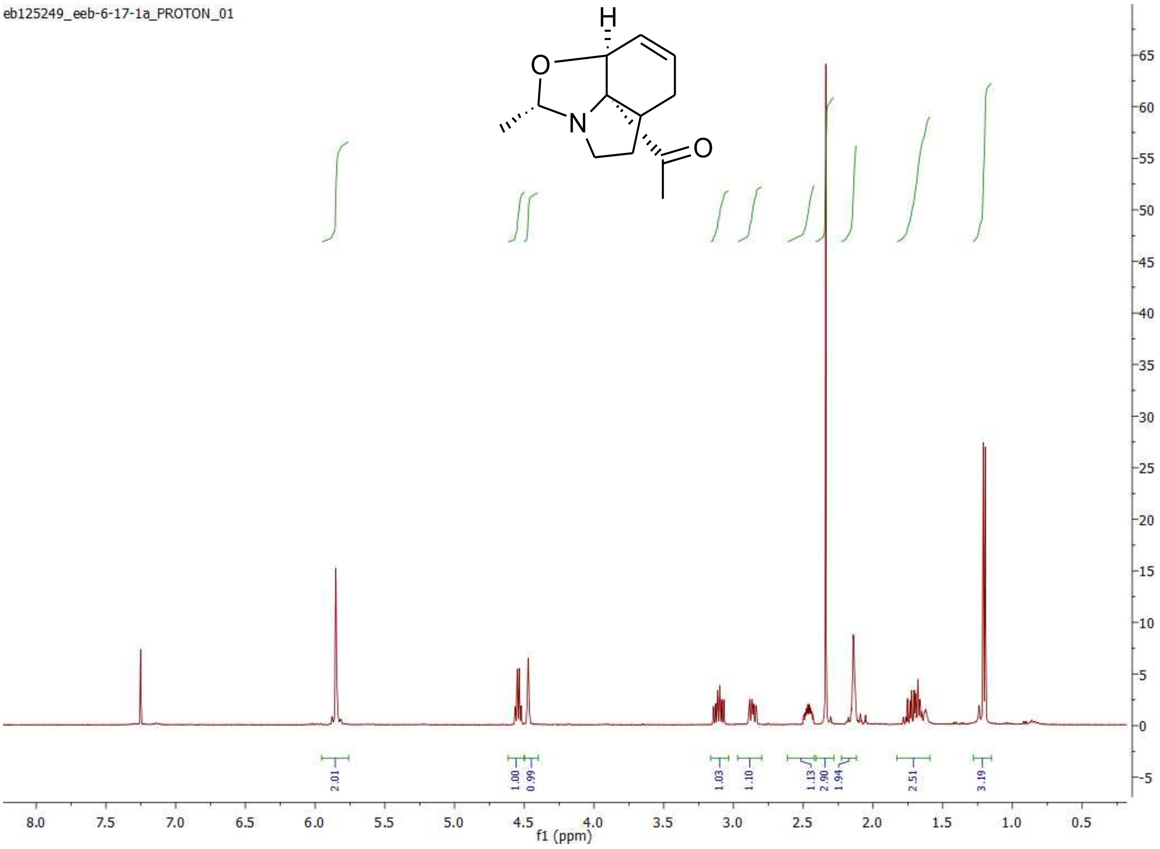

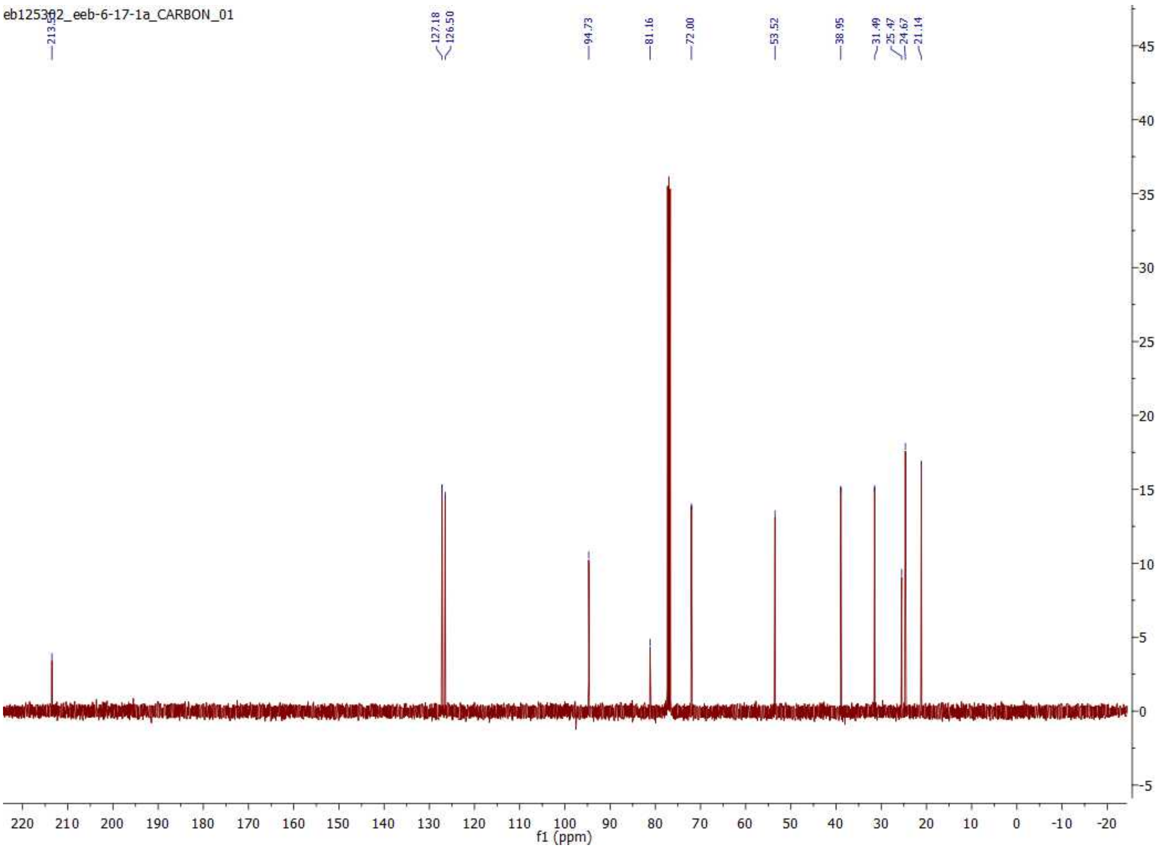

(±)-1-((3*R*,8a*S*)-2-Methyl-5,5a,6,8a-tetrahydro-2*H*-oxazolo[5,4,3-hi]indol-3(4*H*)-yl)ethan-1-one- Major Diastereomer 14b

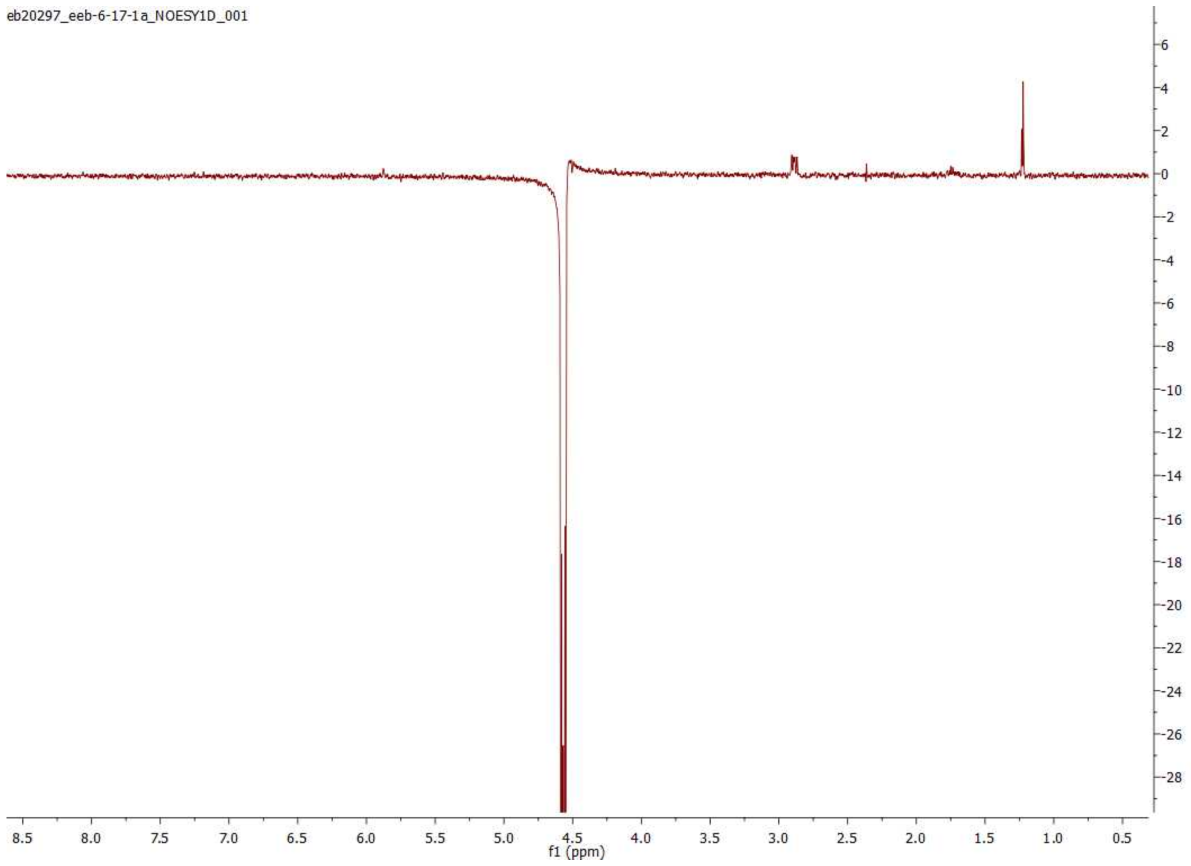

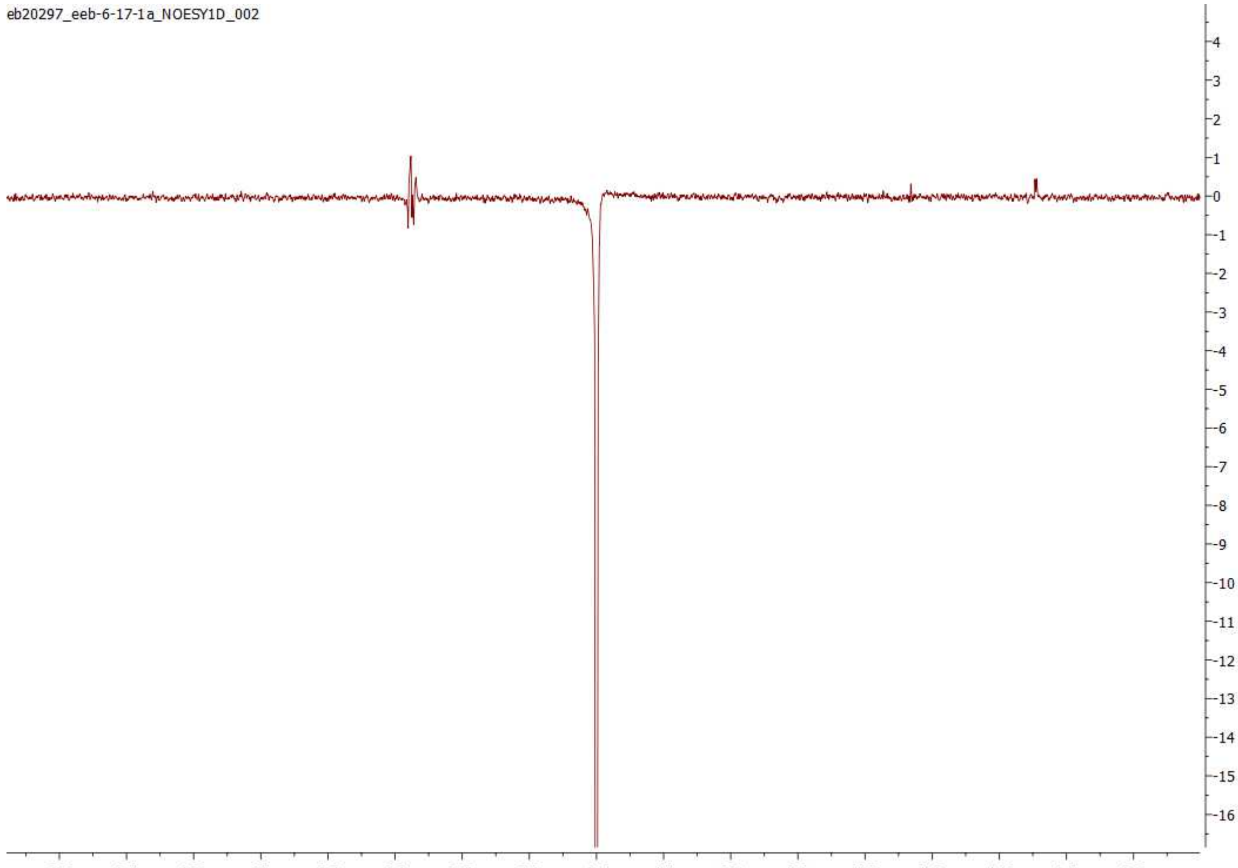

(±)-1-((2*R*,3*R*,8a*S*)-2-methyl-5,5a,6,8a-tetrahydro-2*H*-oxazolo[5,4,3-hi]indol-3(4*H*)-yl)ethan-1-one Minor diastereomer 14b

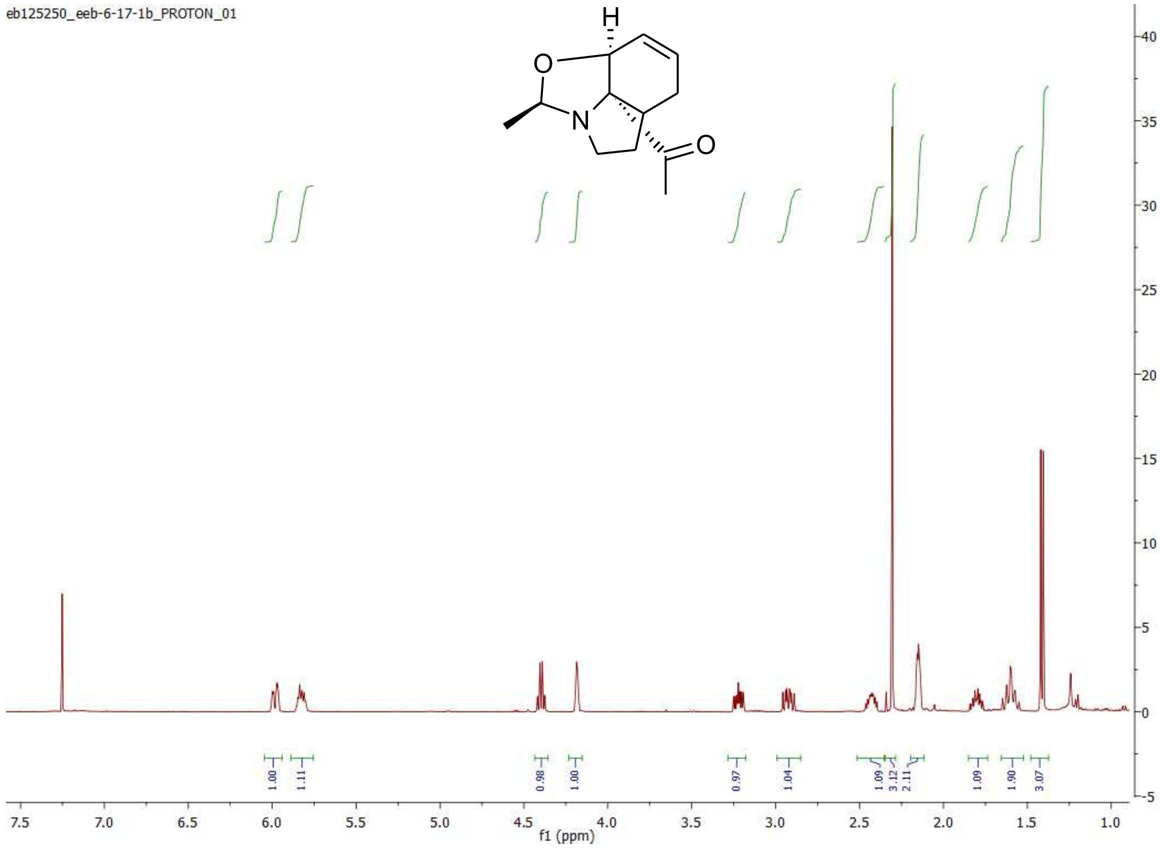

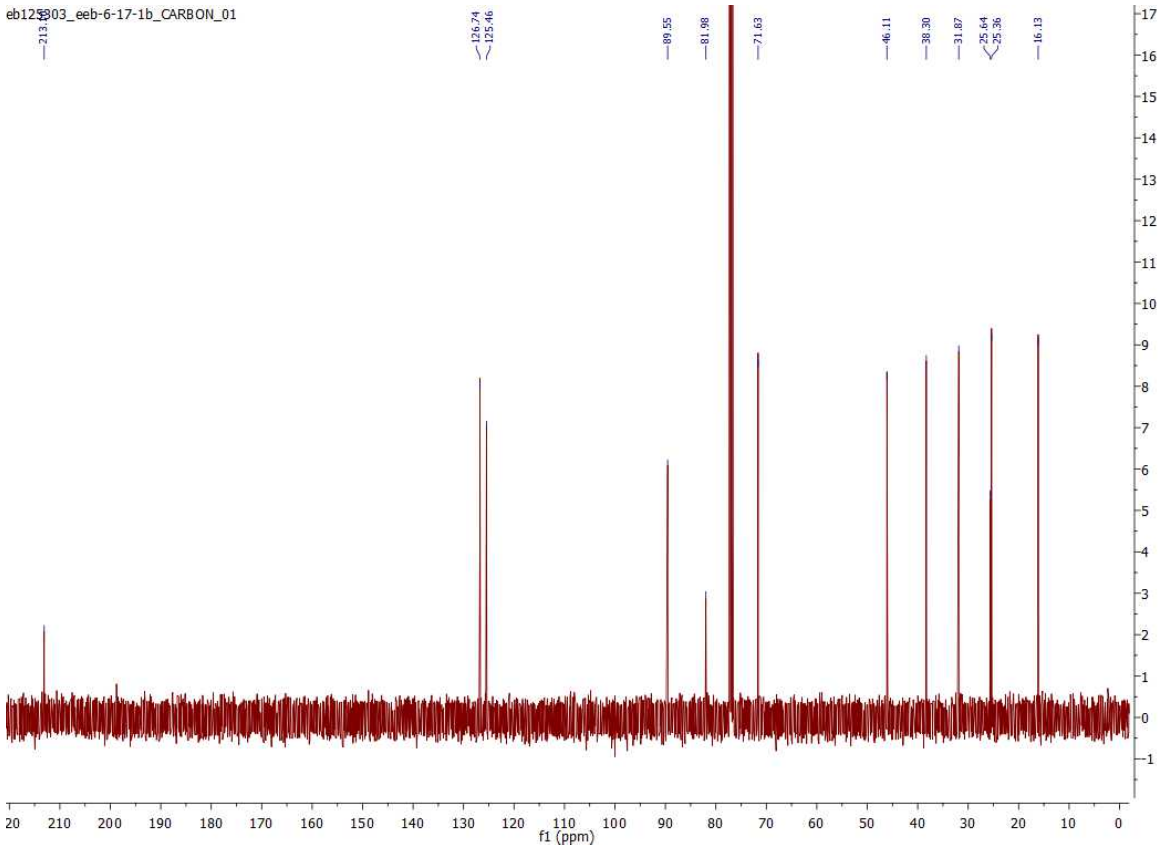

(±)-1-((2*R*,3*R*,8a*S*)-2-methyl-5,5a,6,8a-tetrahydro-2*H*-oxazolo[5,4,3-hi]indol-3(4*H*)-yl)ethan-1-one Minor diastereomer 14b

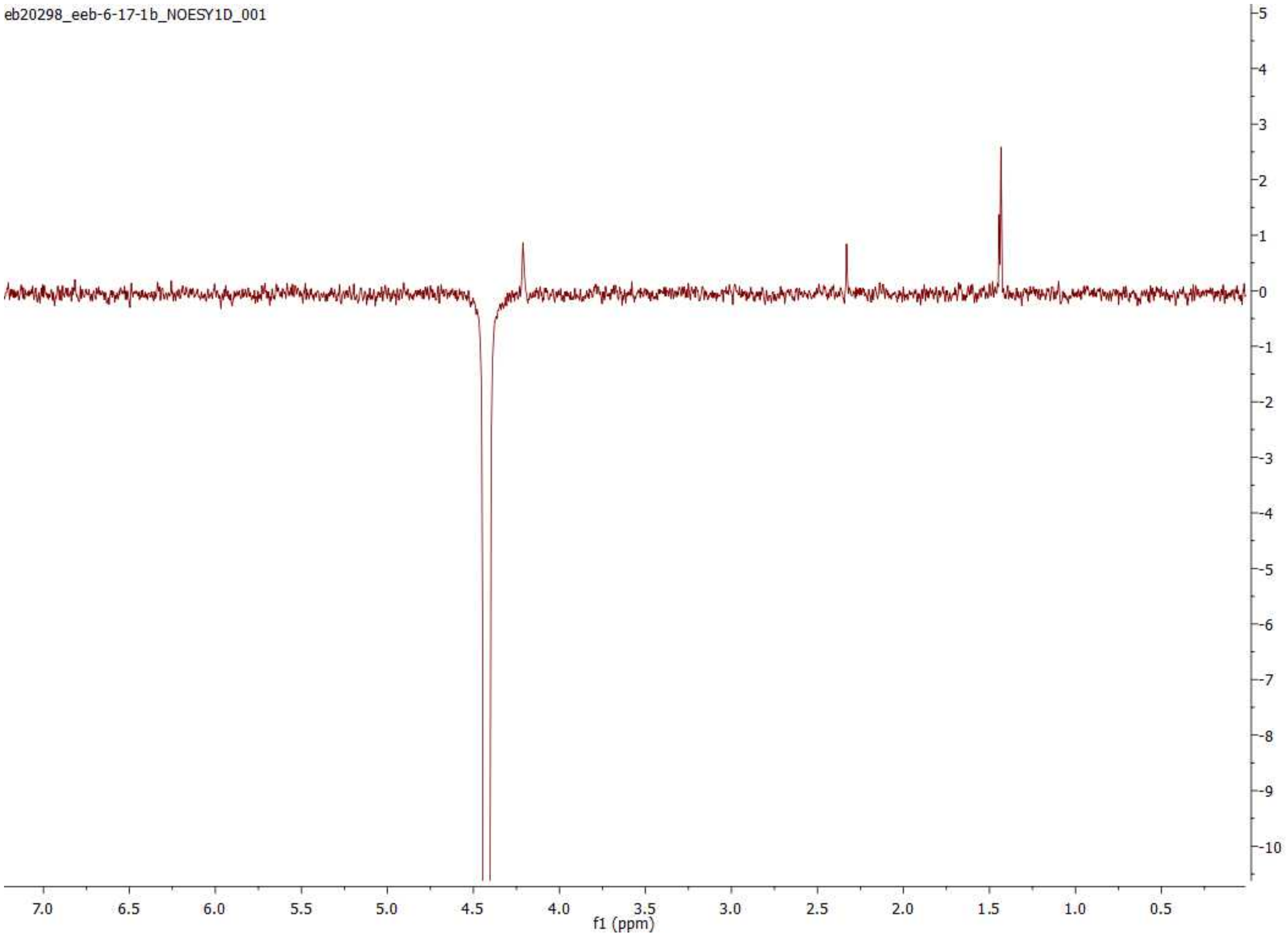

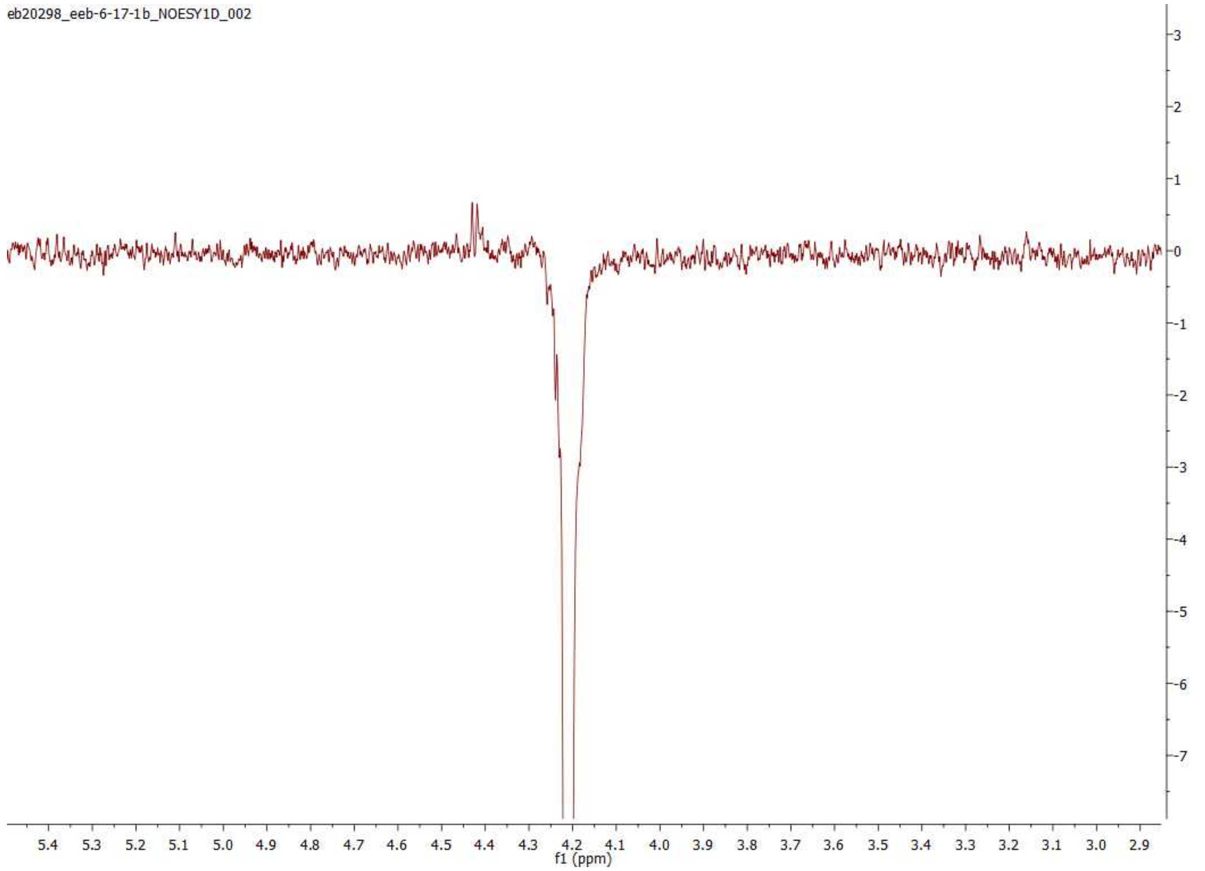

(±)-Ethyl (3*R*,8a*S*)-31-(ethylcarbamoyl)-3,4,5,5a,6,8a-hexahydro-2*H*-oxazolo[5,4,3-hi]indole-2-carboxylate- Major diastereomer 15a

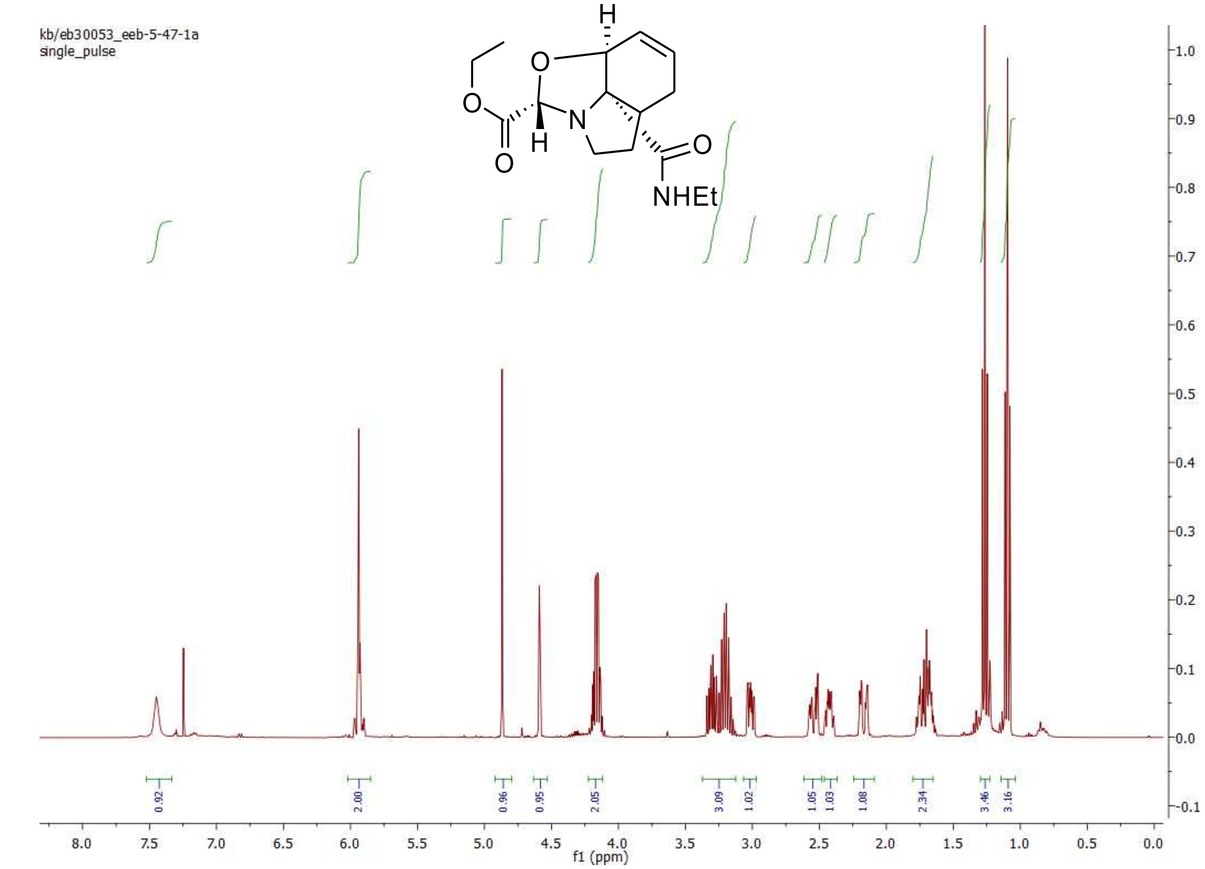

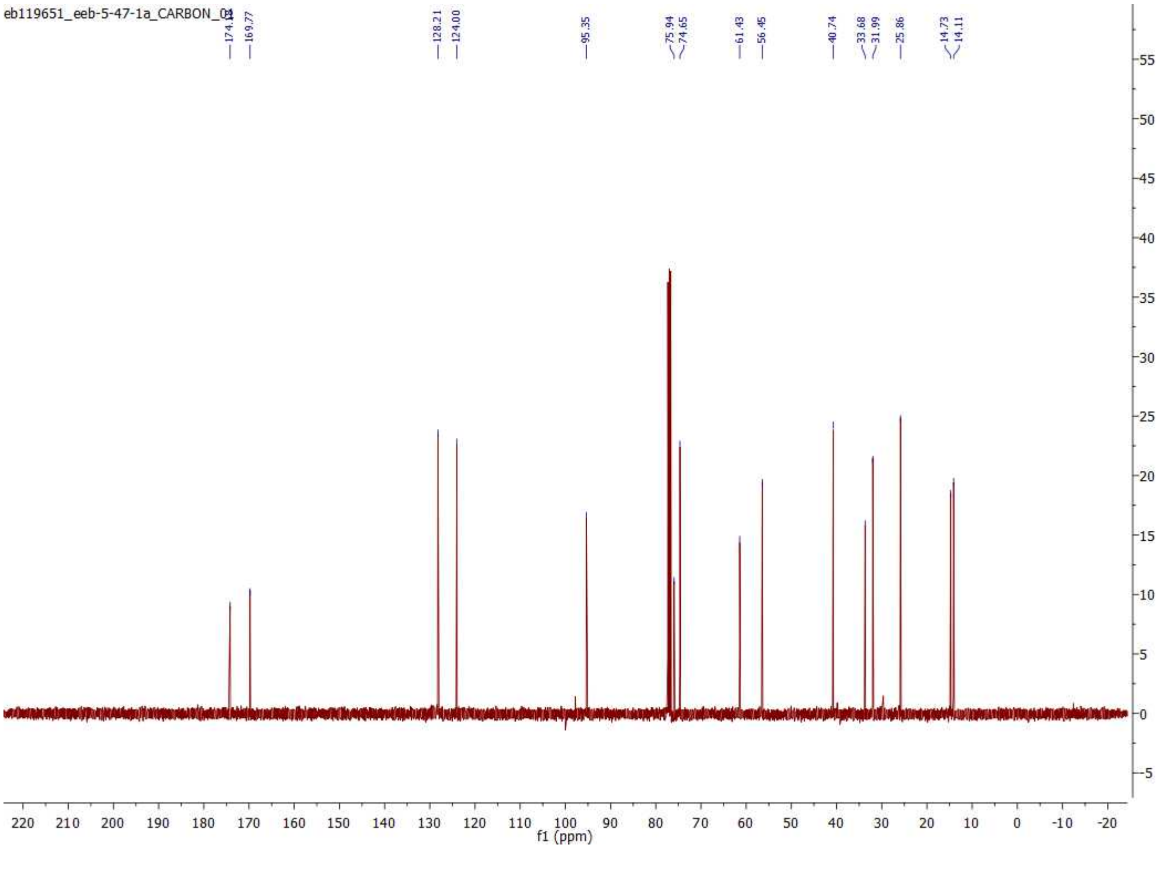

(±)-Ethyl (3*R*,8a*S*)-31-(ethylcarbamoyl)-3,4,5,5a,6,8a-hexahydro-2*H*-oxazolo[5,4,3-hi]indole-2-carboxylate- Major diastereomer 15a

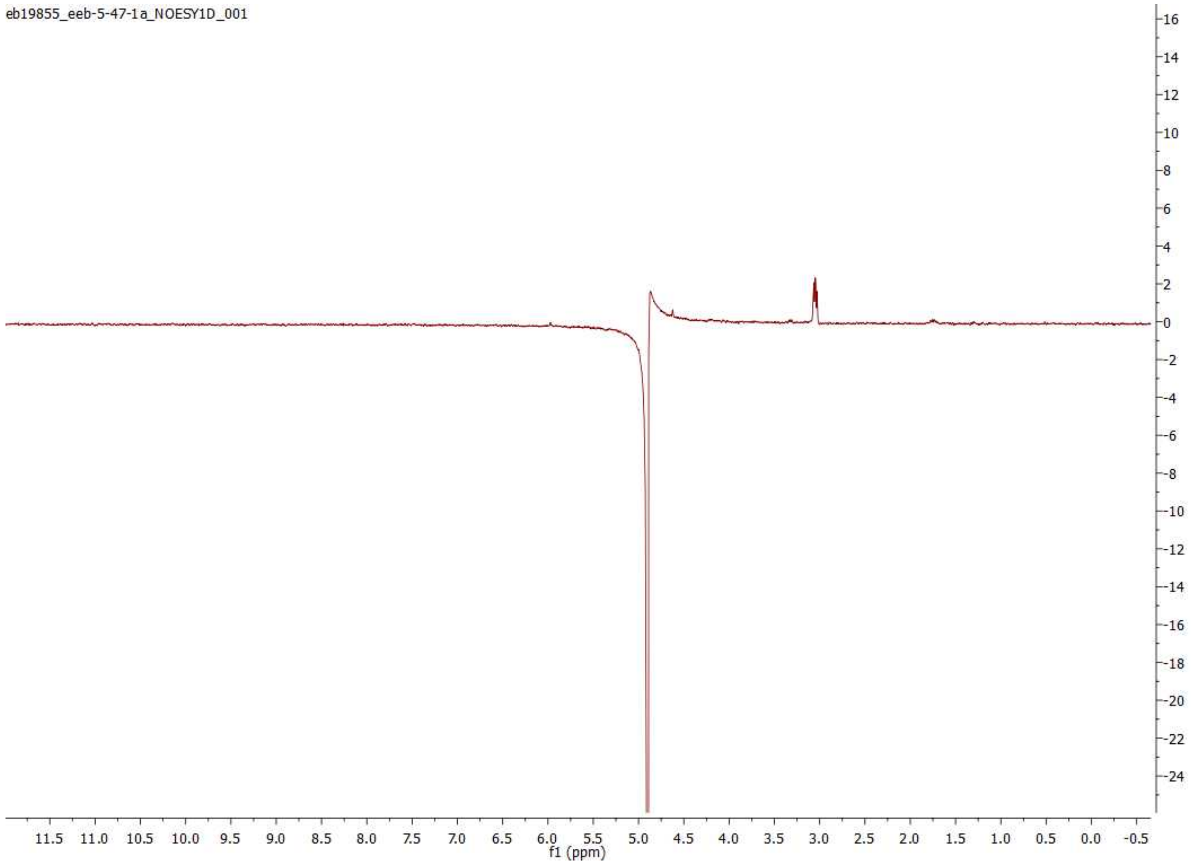

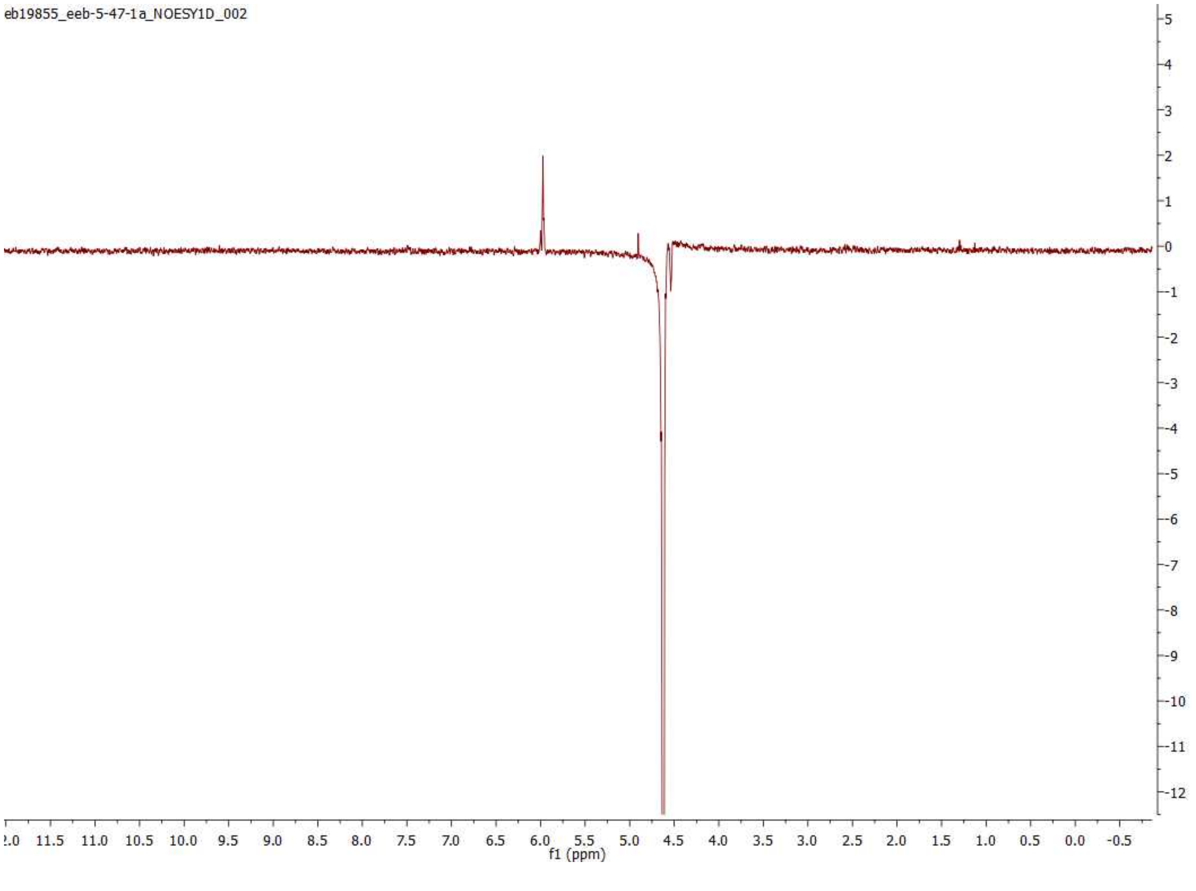

(±)-Ethyl (3*R*,8a*S*)-31-(ethylcarbamoyl)-3,4,5,5a,6,8a-hexahydro-2*H*-oxazolo[5,4,3-hi]indole-2-carboxylate- Major diastereomer 15a

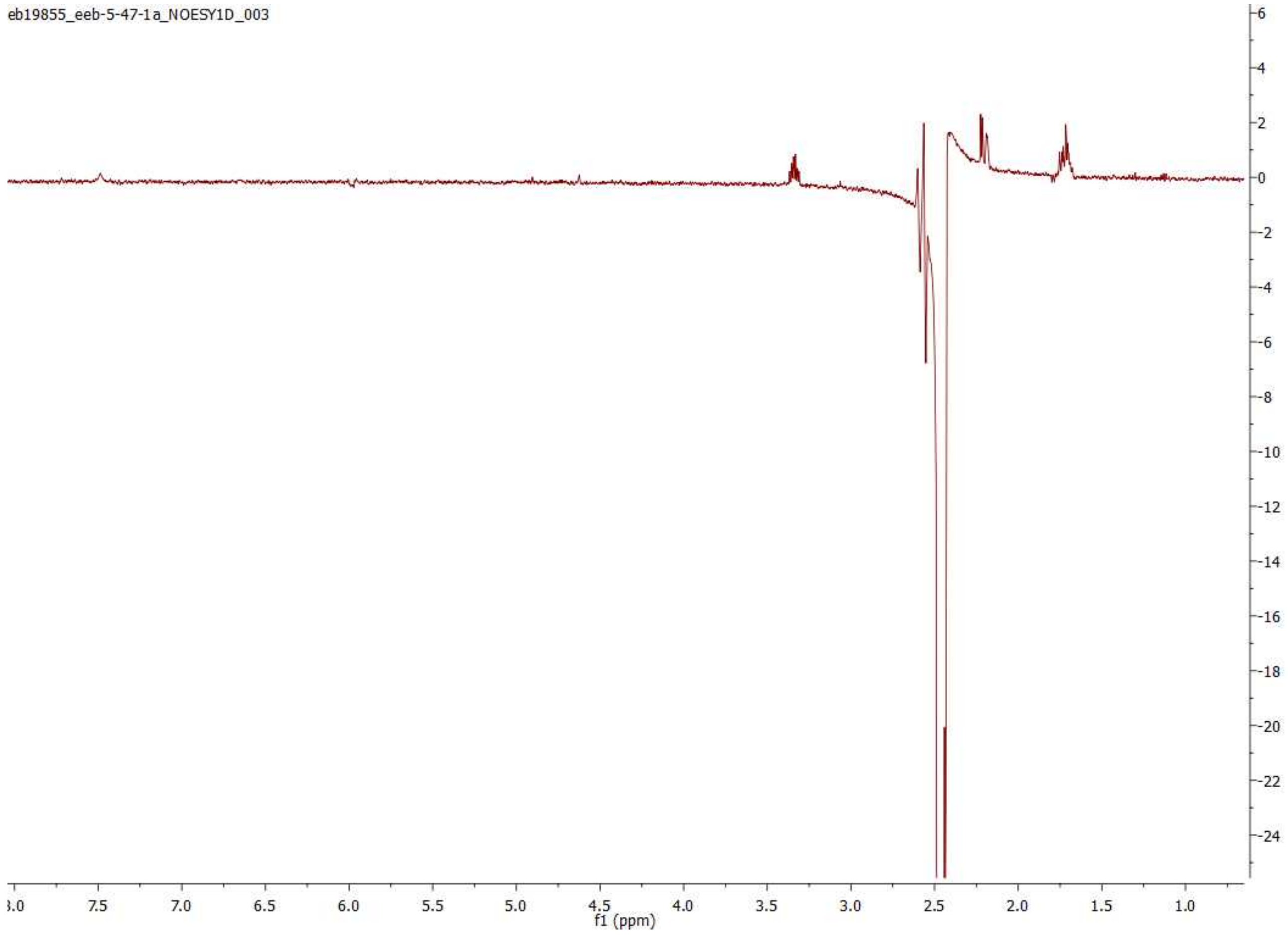

(±)-Ethyl (3*R*,8a*S*)-3-(ethylcarbamoyl)-3,4,5,5a,6,8a-hexahydro-2H-oxazolo[5,4,3-hi]indole-2-carboxylate – mixture of diastereomers 15a

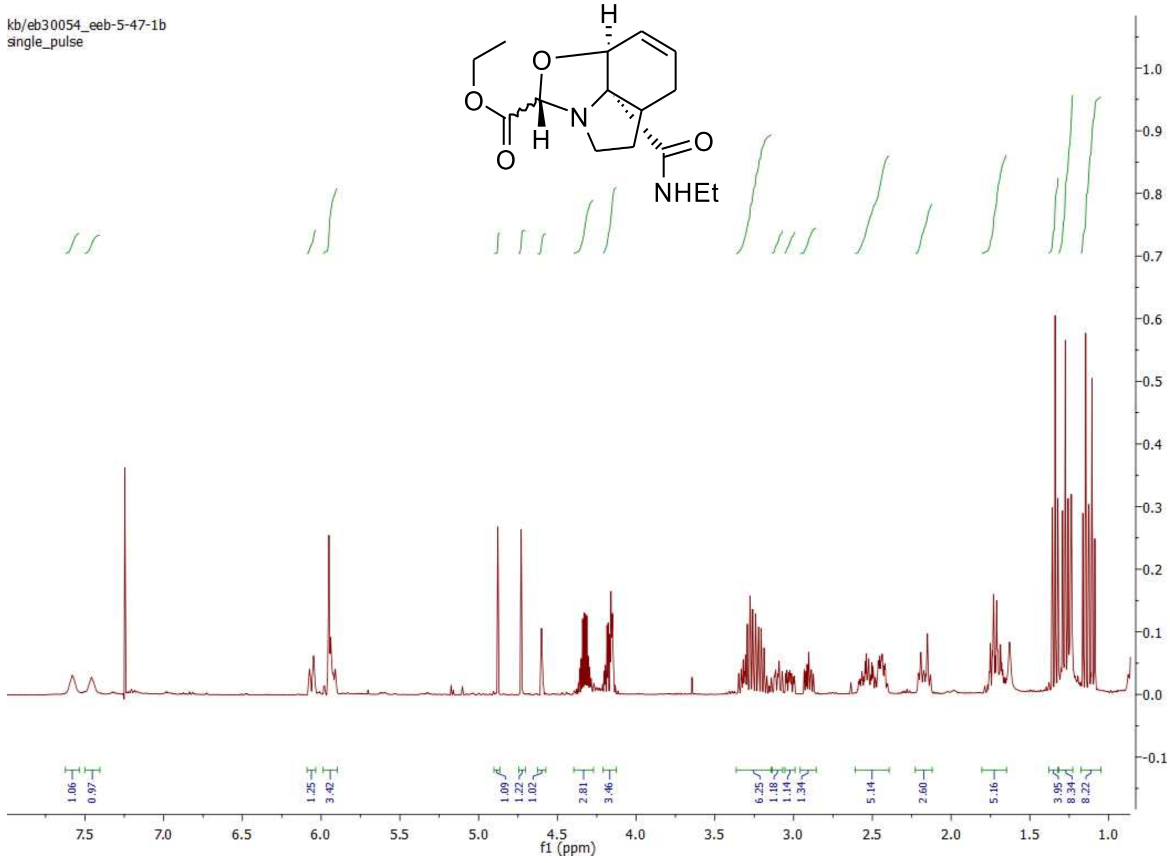

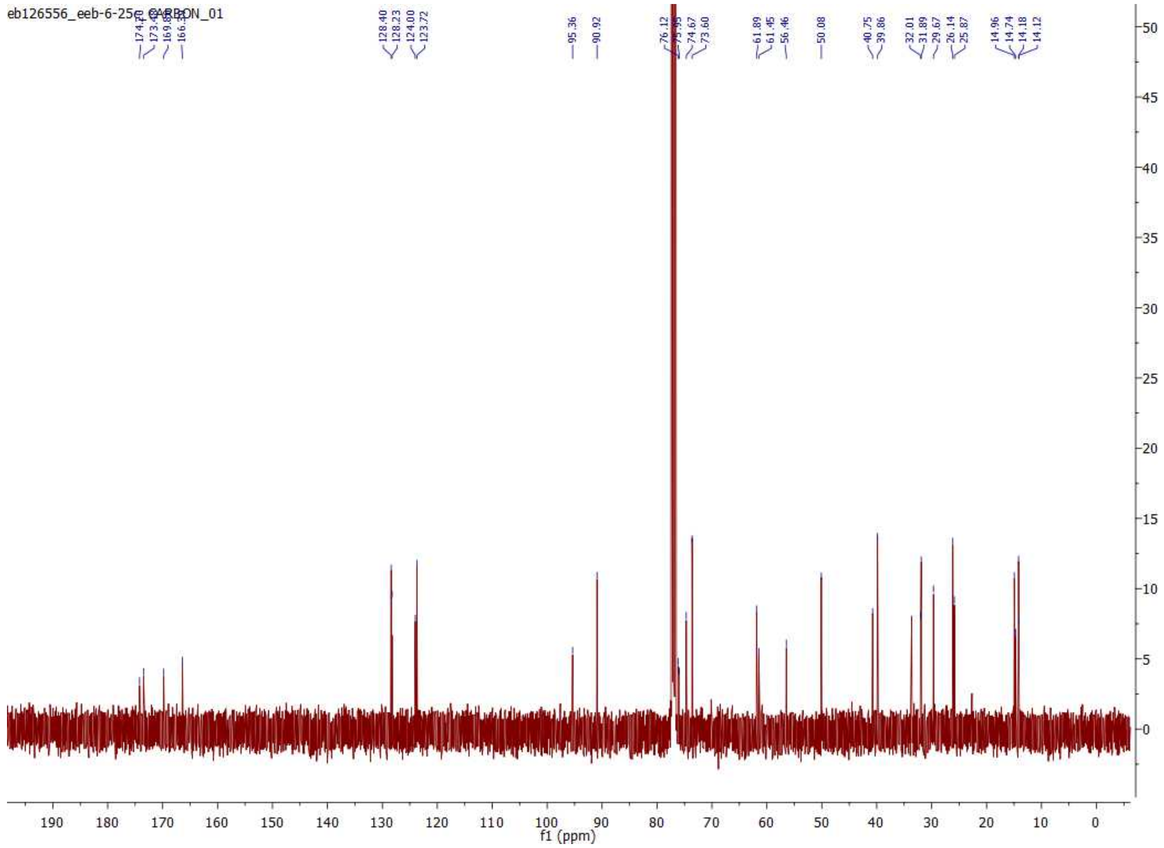

(±)-Ethyl (3*R*,8a*S*)-3-(ethylcarbamoyl)-3,4,5,5a,6,8a-hexahydro-2H-oxazolo[5,4,3-hi]indole-2-carboxylate – mixture of diastereomers 15a

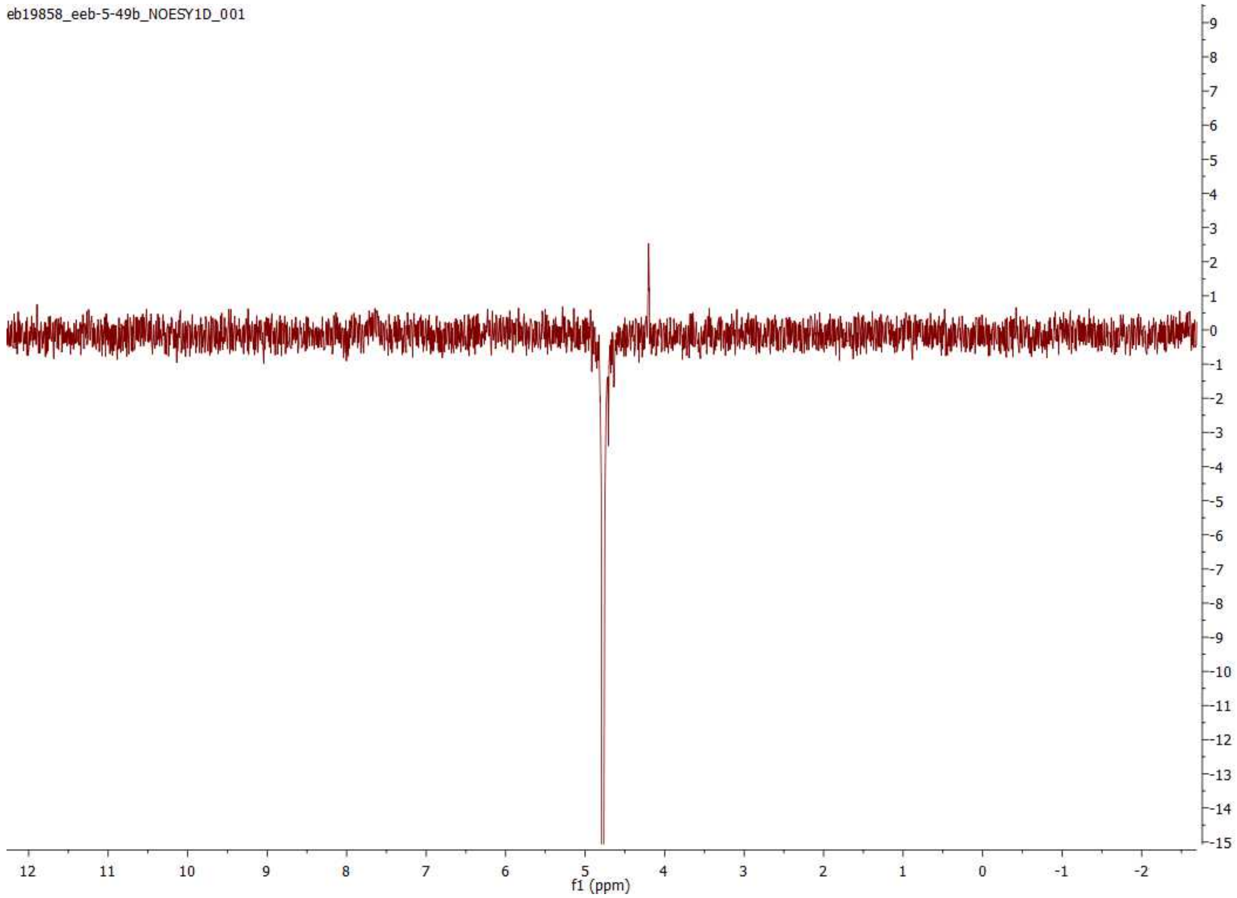

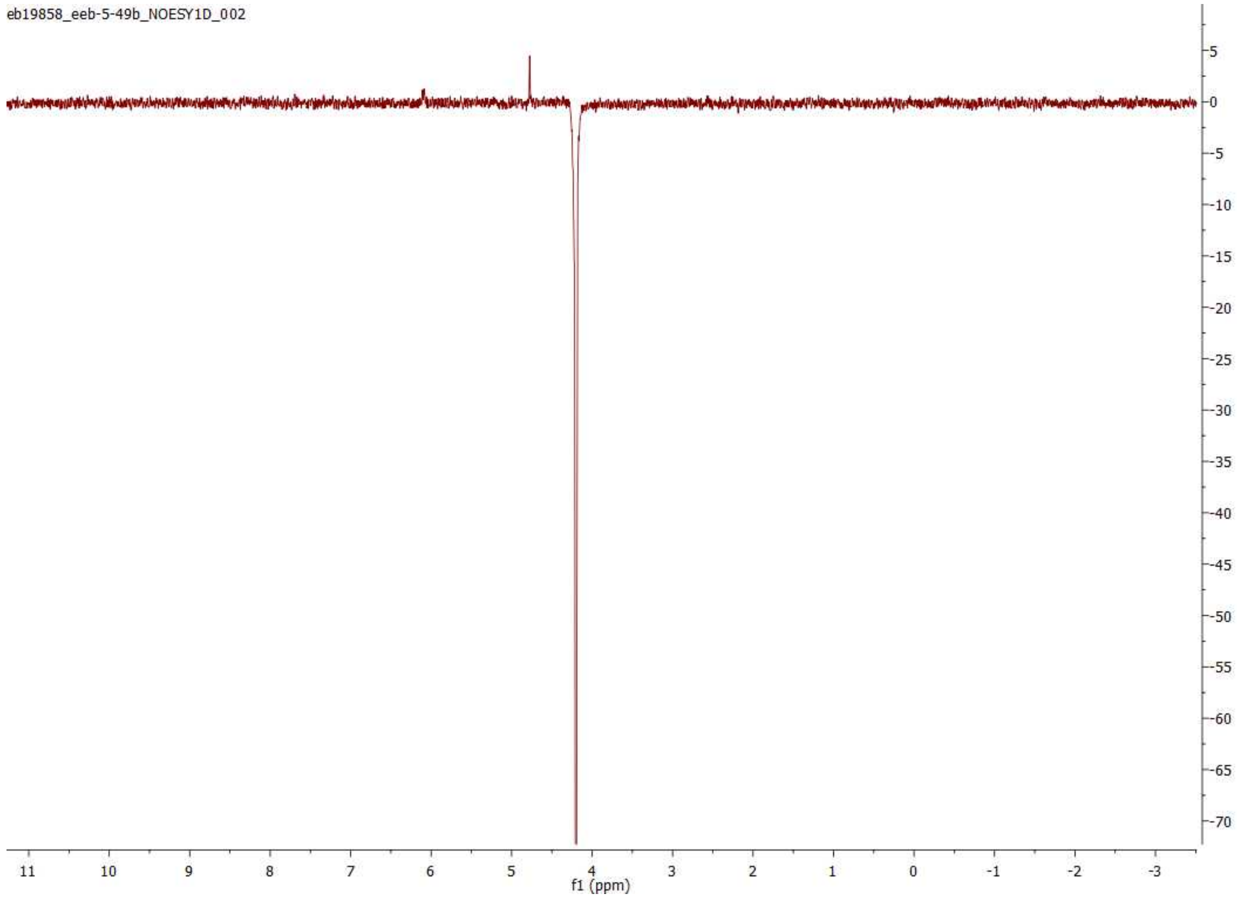

(±)-Ethyl (2*S*,3*R*,8a*S*)-3-acetyl-3,4,5,5a,6,8a-hexahydro-2*H*-oxazolo[5,4,3-hi]indole-2-carboxylate– Major Diastereomer 15b

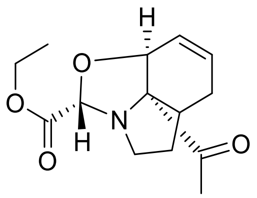

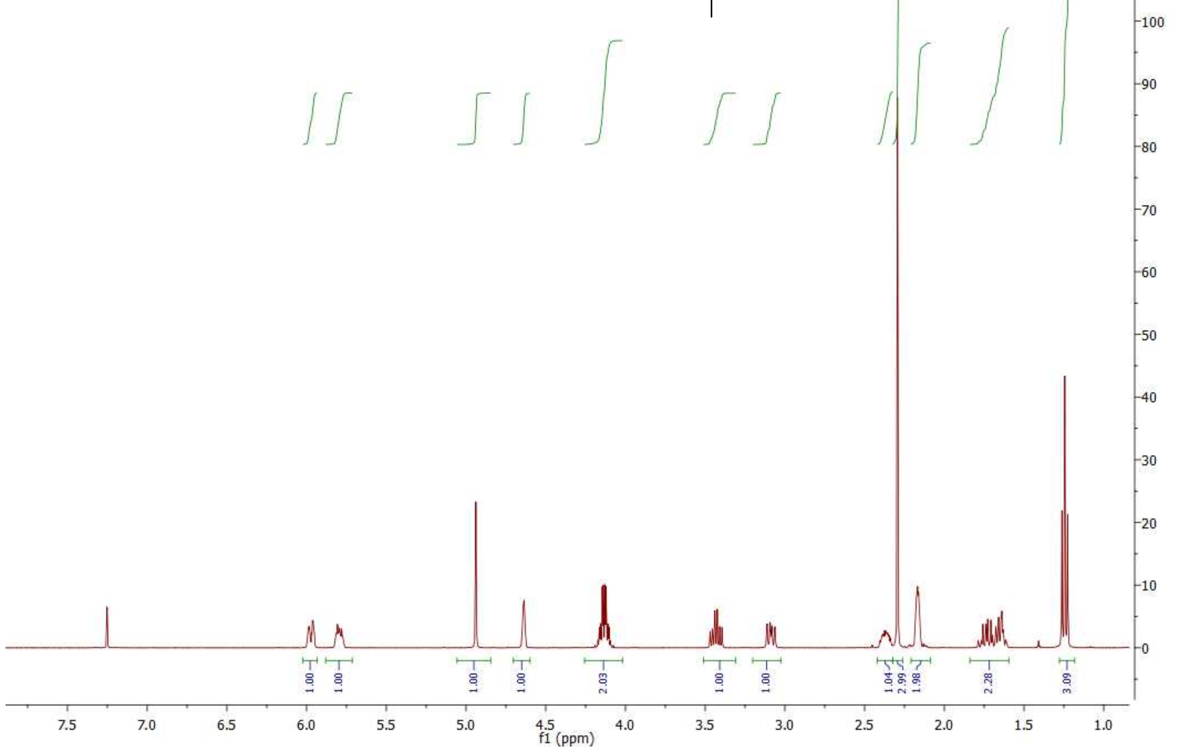

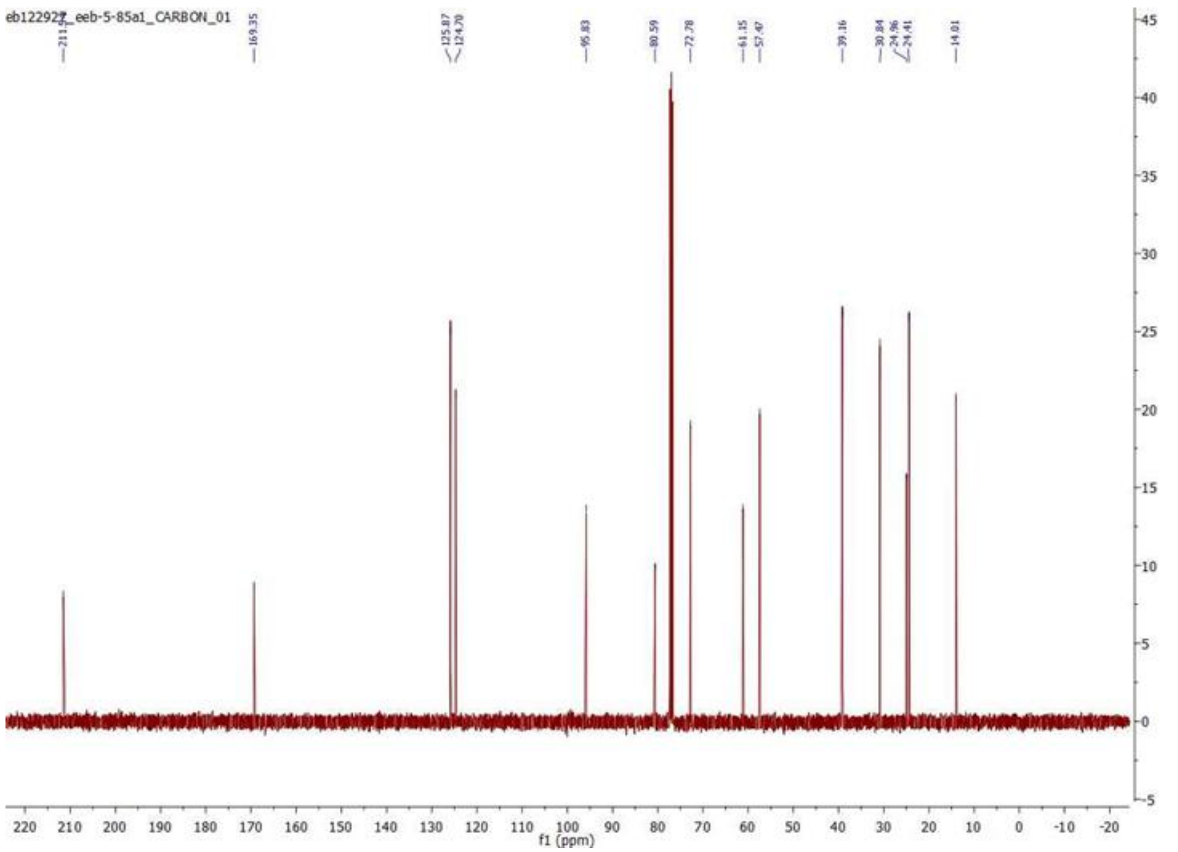

(±)-Ethyl (2*S*,3*R*,8a*S*)-3-acetyl-3,4,5,5a,6,8a-hexahydro-2*H*-oxazolo[5,4,3-hi]indole-2-carboxylate– Major Diastereomer 15b

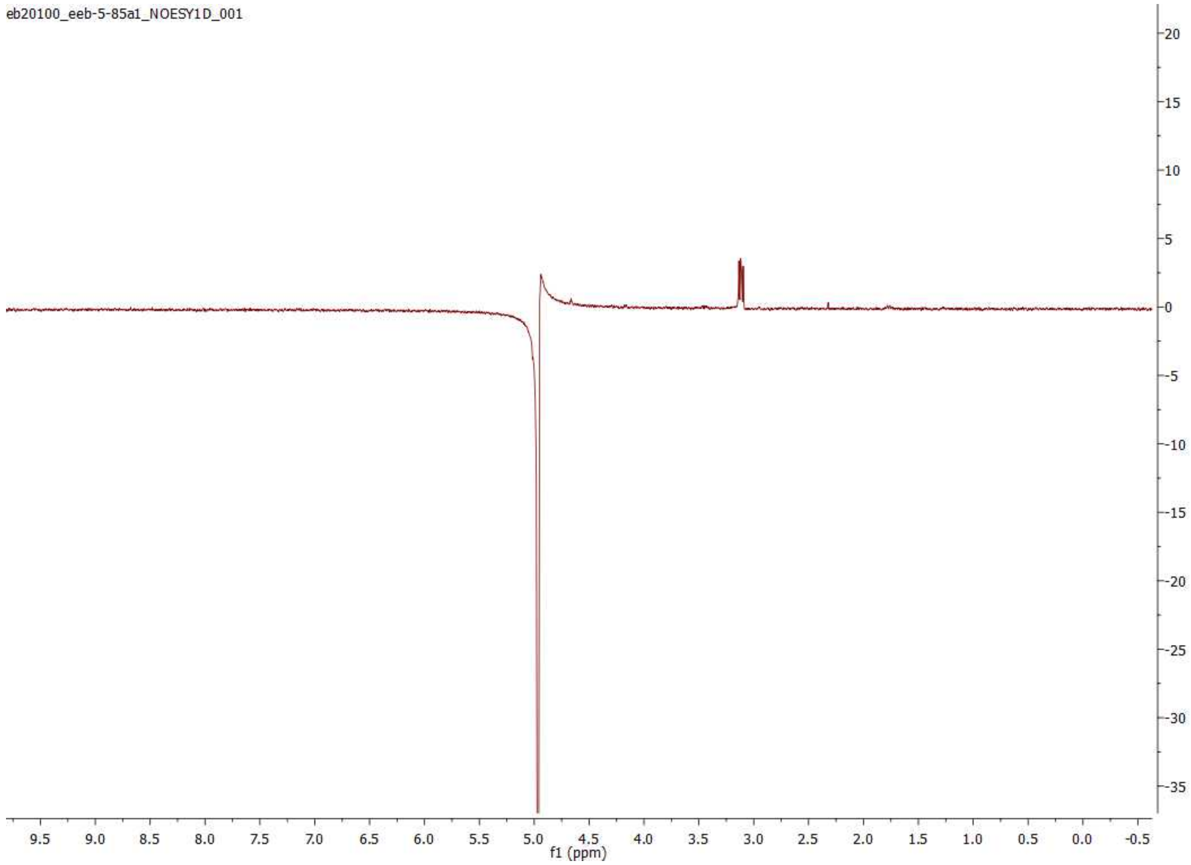

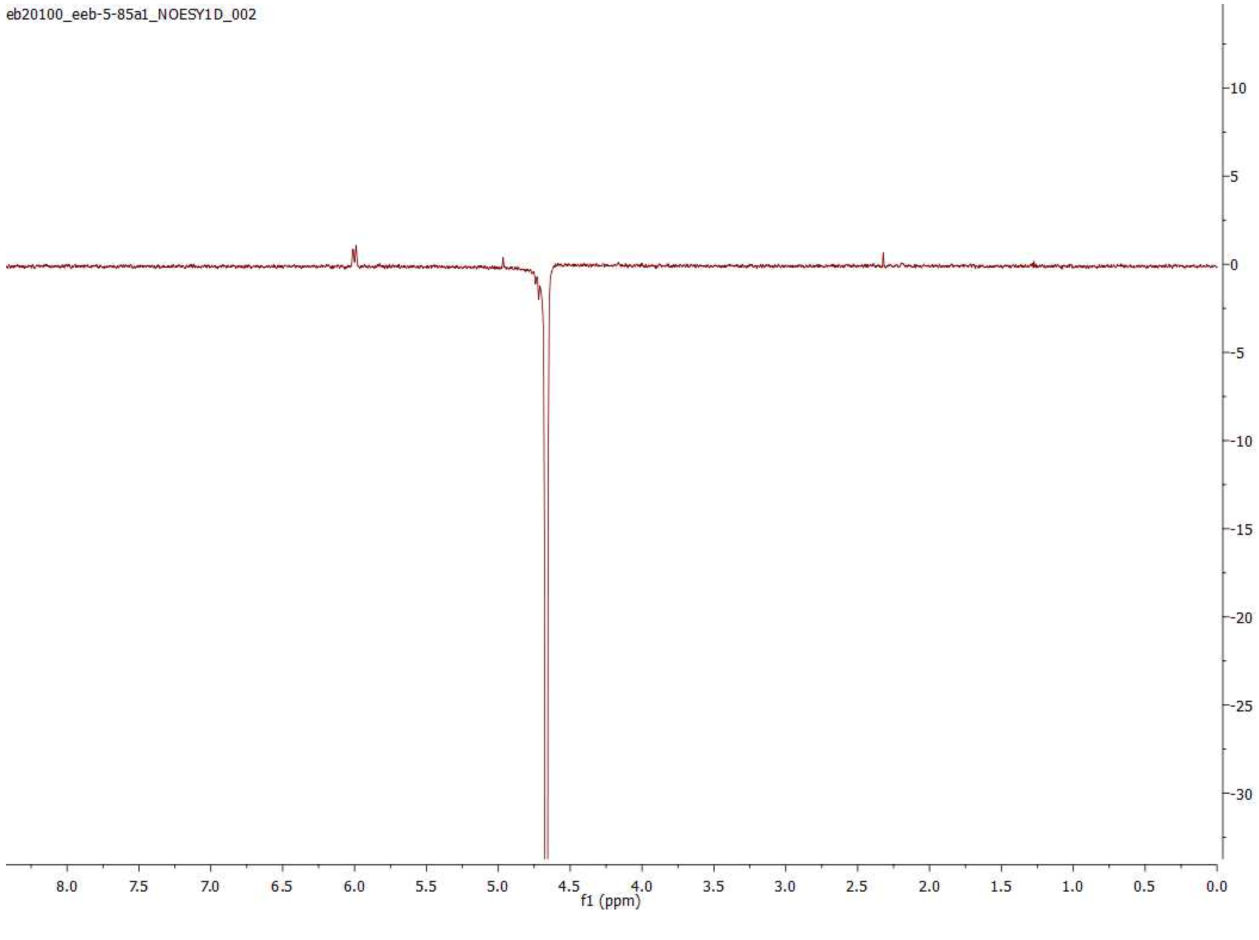

(±)-Ethyl (2*S*,3*R*,8a*S*)-3-acetyl-3,4,5,5a,6,8a-hexahydro-2*H*-oxazolo[5,4,3-hi]indole-2-carboxylate– Major Diastereomer 15b

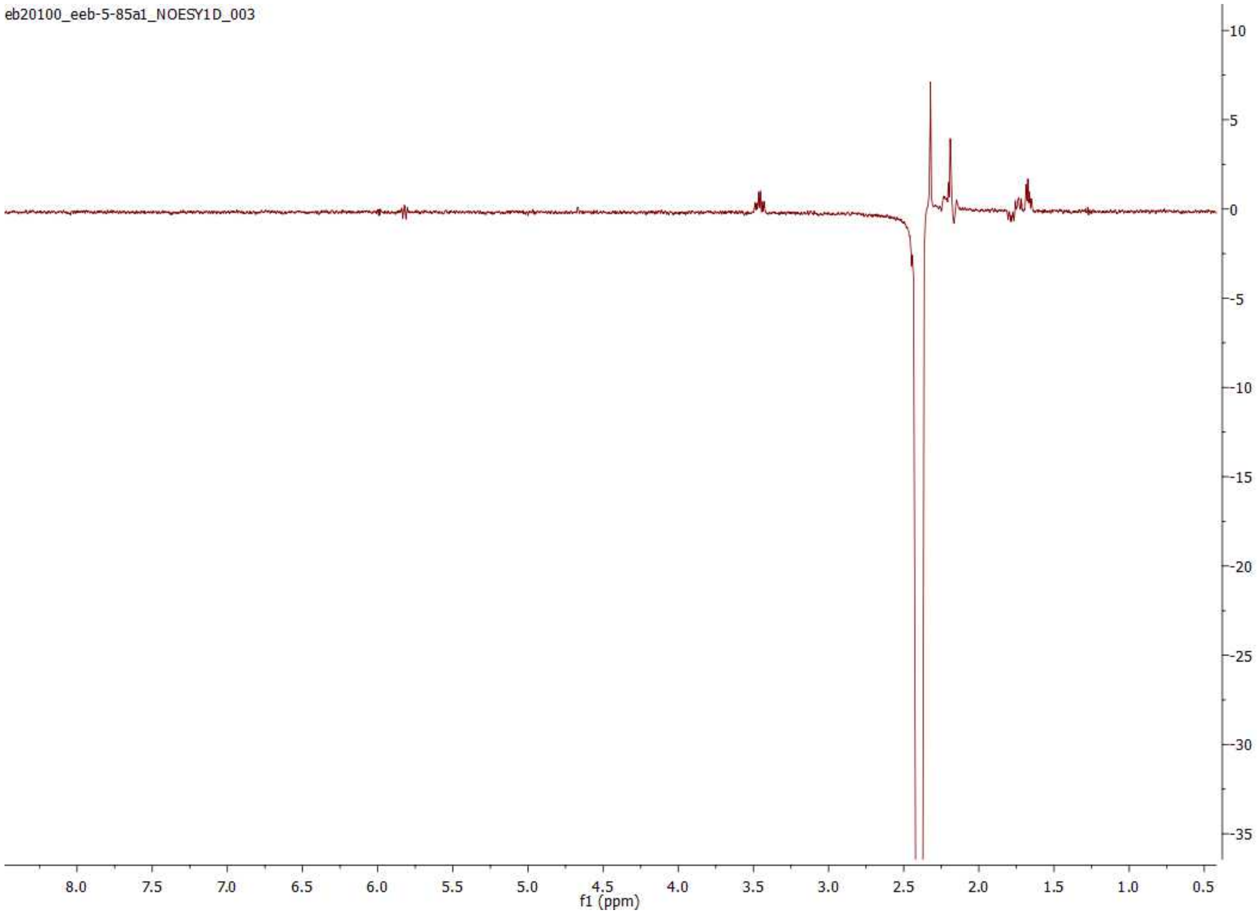

(±)-Ethyl (2*R*,3*R*,8a*S*)-3-acetyl-3,4,5,5a,6,8a-hexahydro-2H-oxazolo[5,4,3-hi]indole-2-carboxylate – minor diastereomer 15b

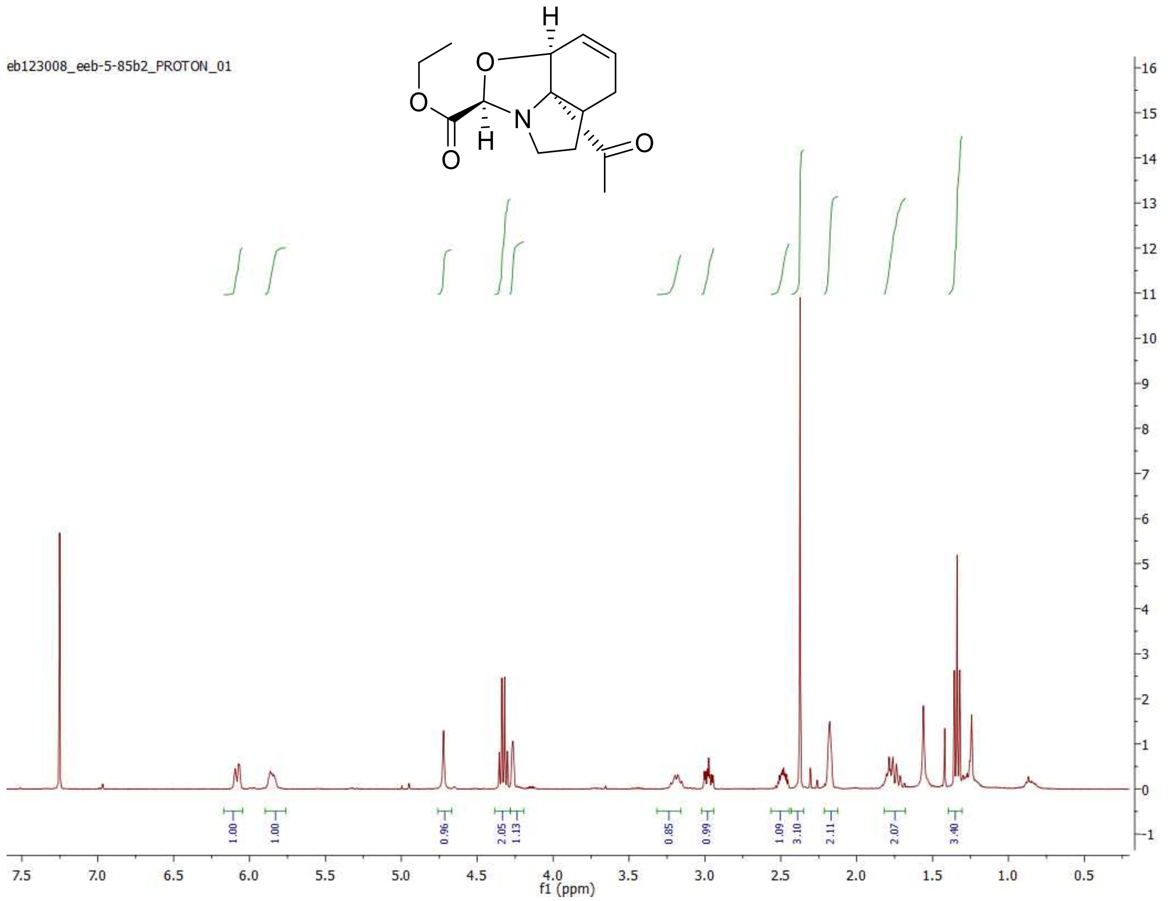

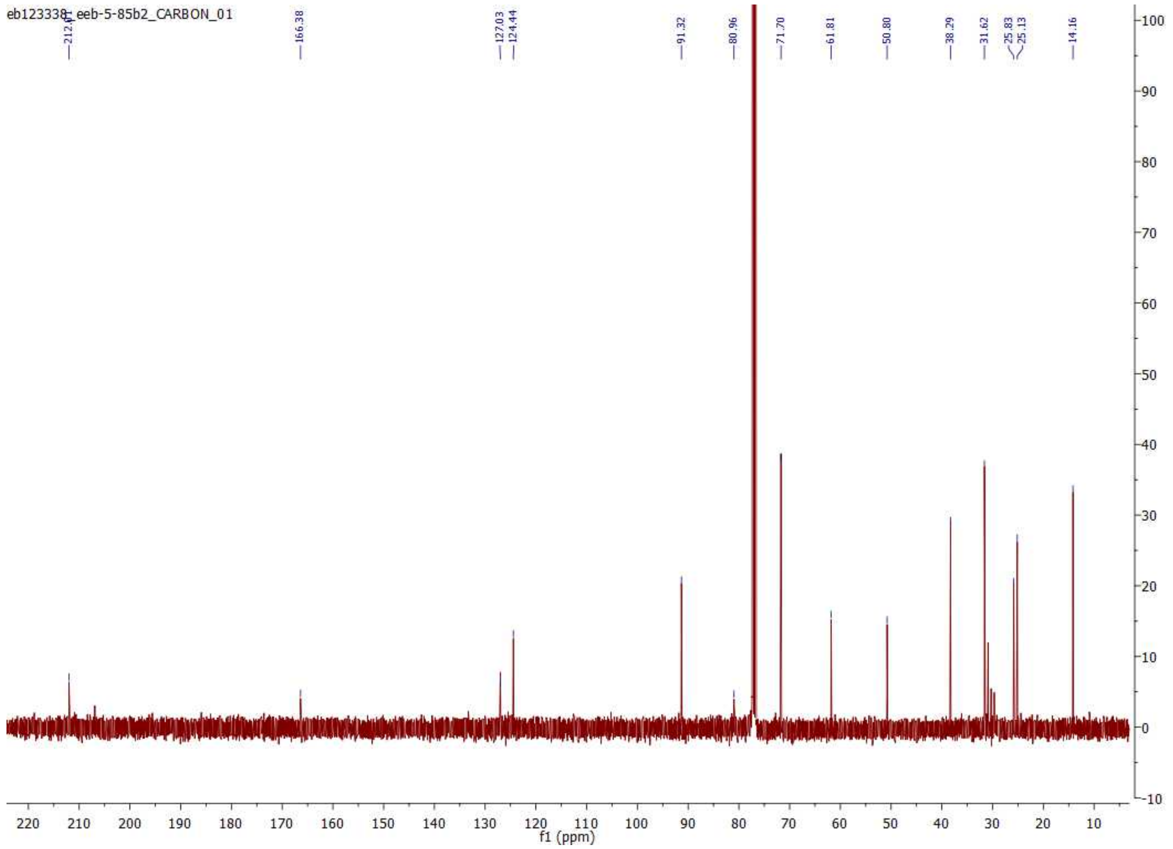

(±)-Ethyl (2*R*,3*R*,8a*S*)-3-acetyl-3,4,5,5a,6,8a-hexahydro-2H-oxazolo[5,4,3-hi]indole-2-carboxylate – minor diastereomer 15b

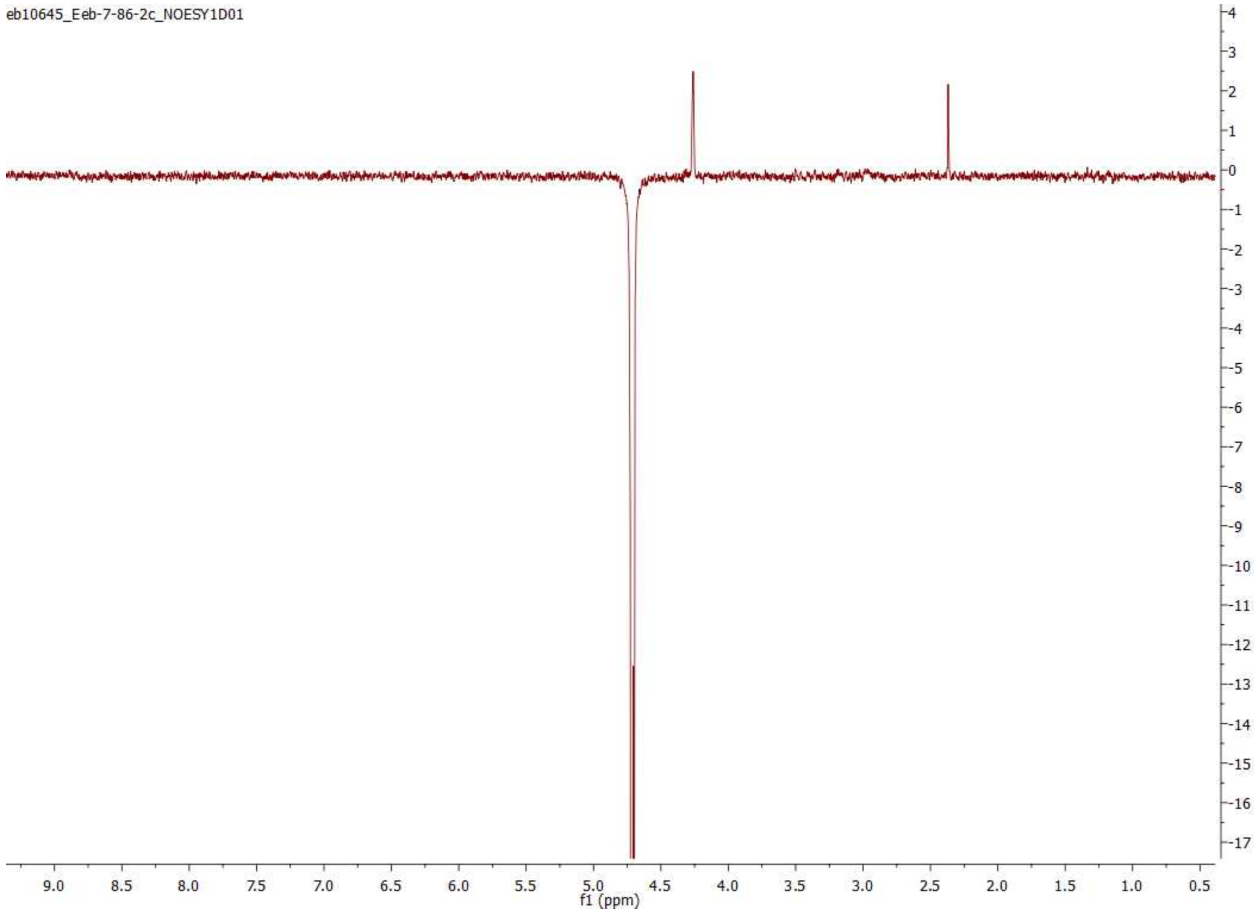

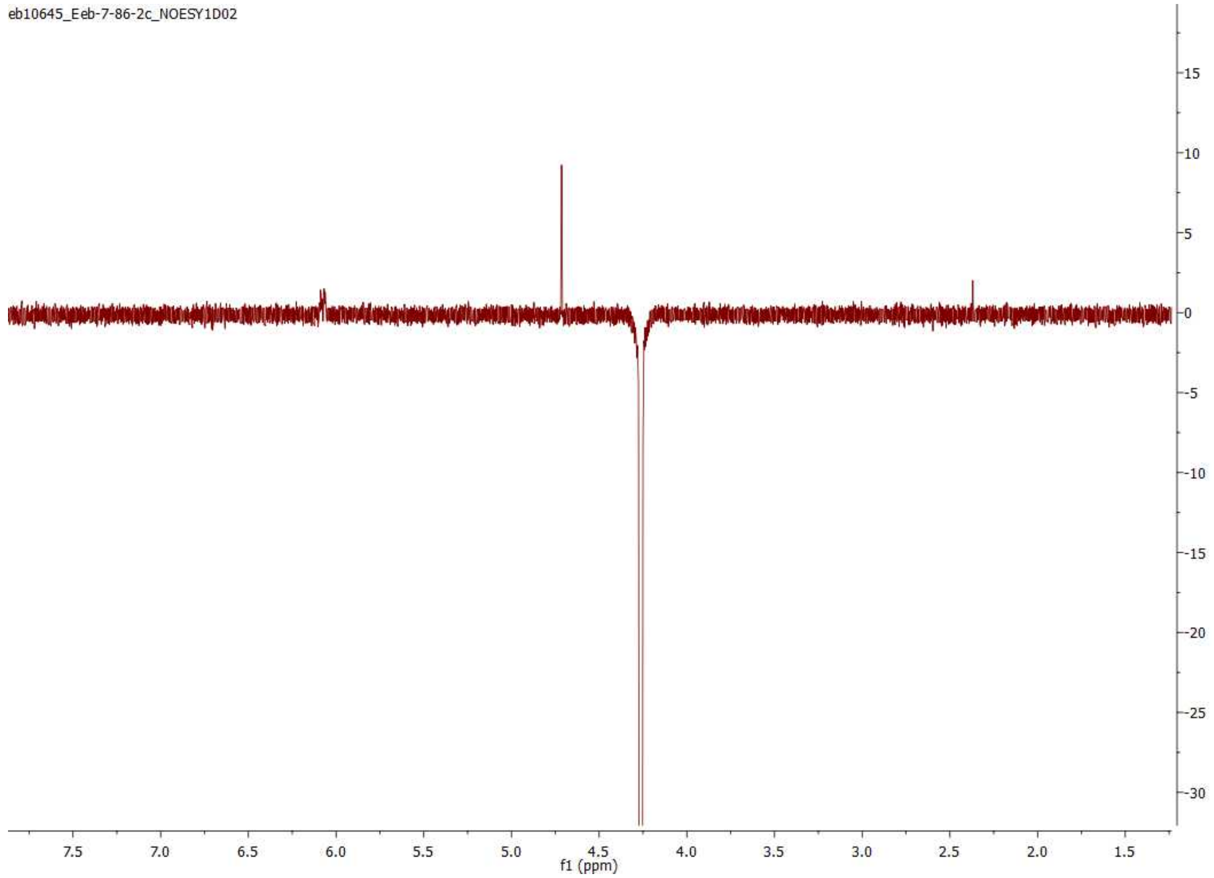

(±)-Ethyl (3*S*,8a*S*)-31-cyano-3,4,5,5a,6,8a-hexahydro-2*H*-oxazolo[5,4,3-hi]indole-2-carboxylate 15c

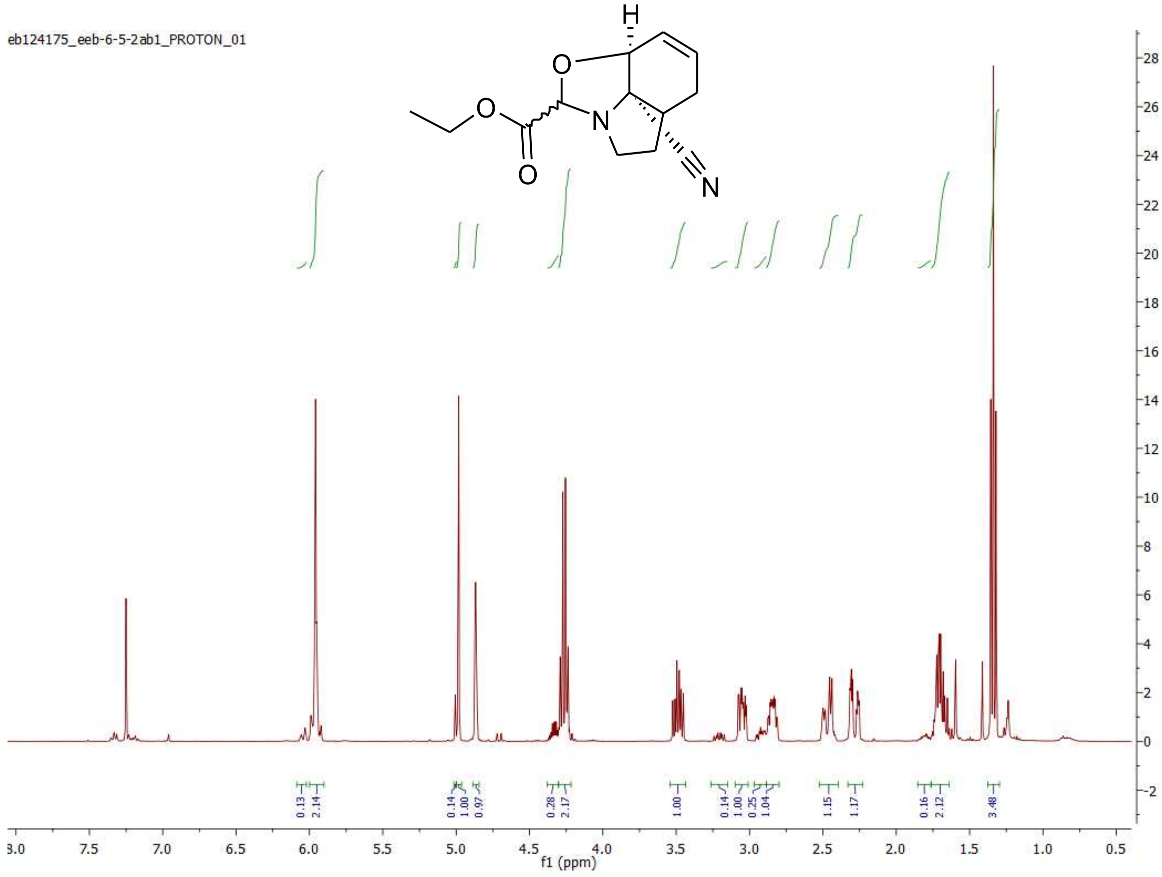

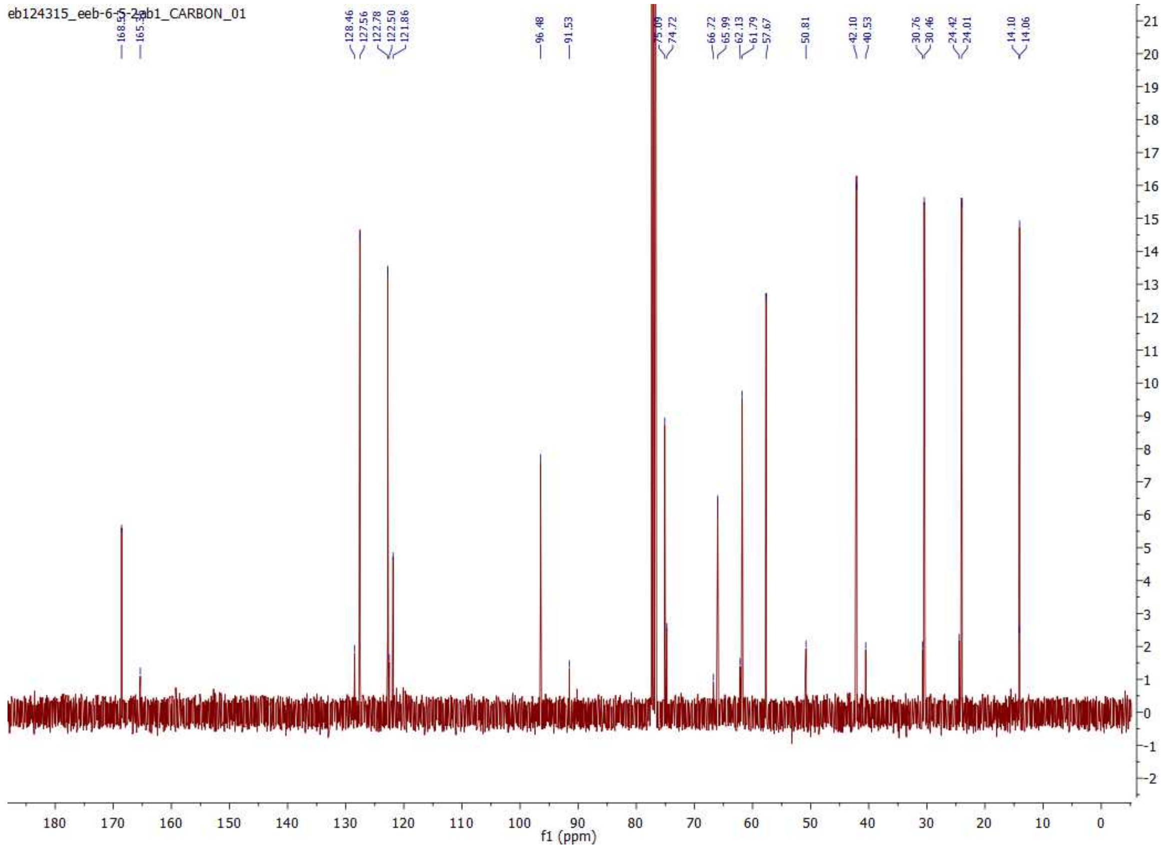

(±)-Ethyl (3*S*,8a*S*)-31-cyano-3,4,5,5a,6,8a-hexahydro-2*H*-oxazolo[5,4,3-hi]indole-2-carboxylate 15c

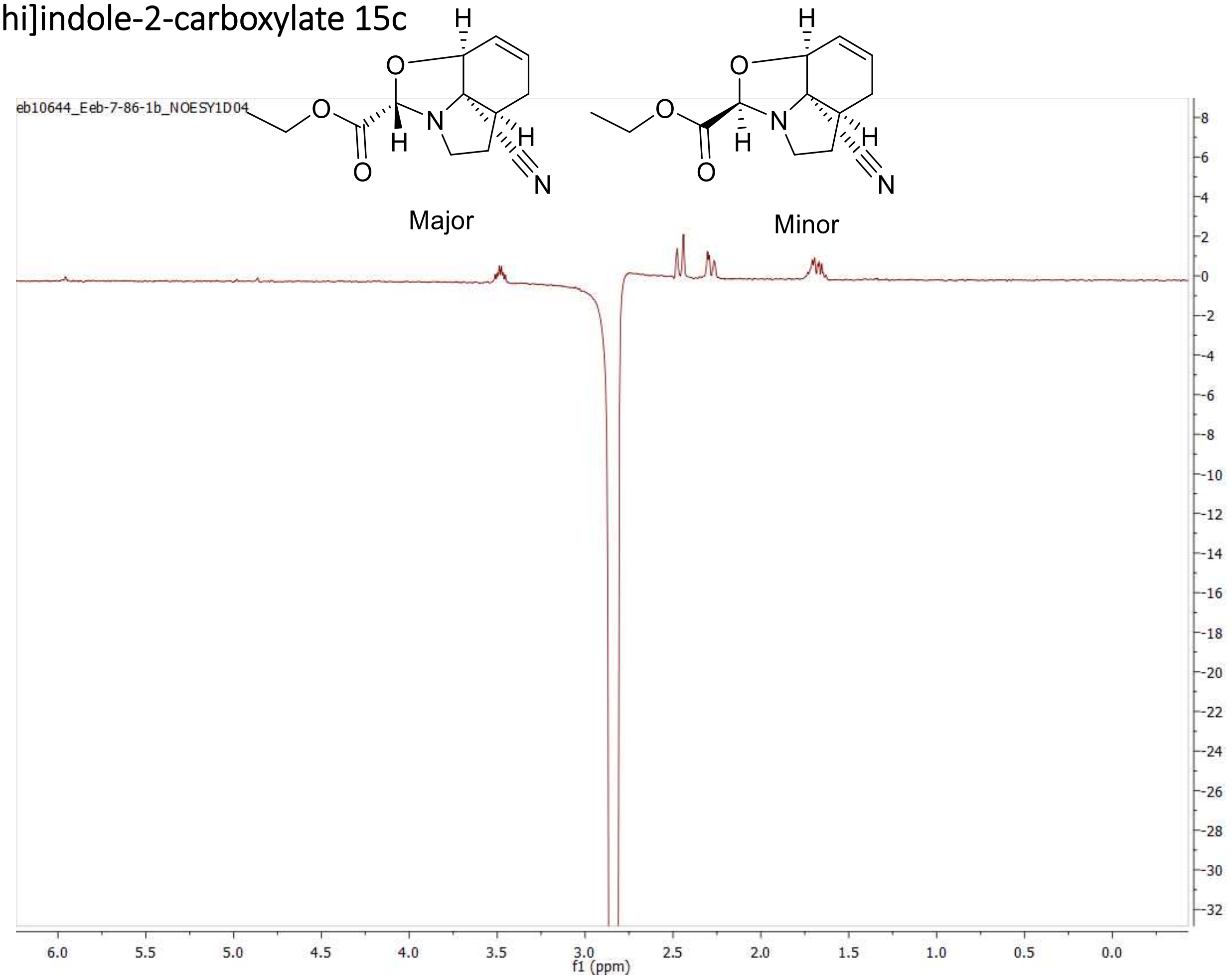

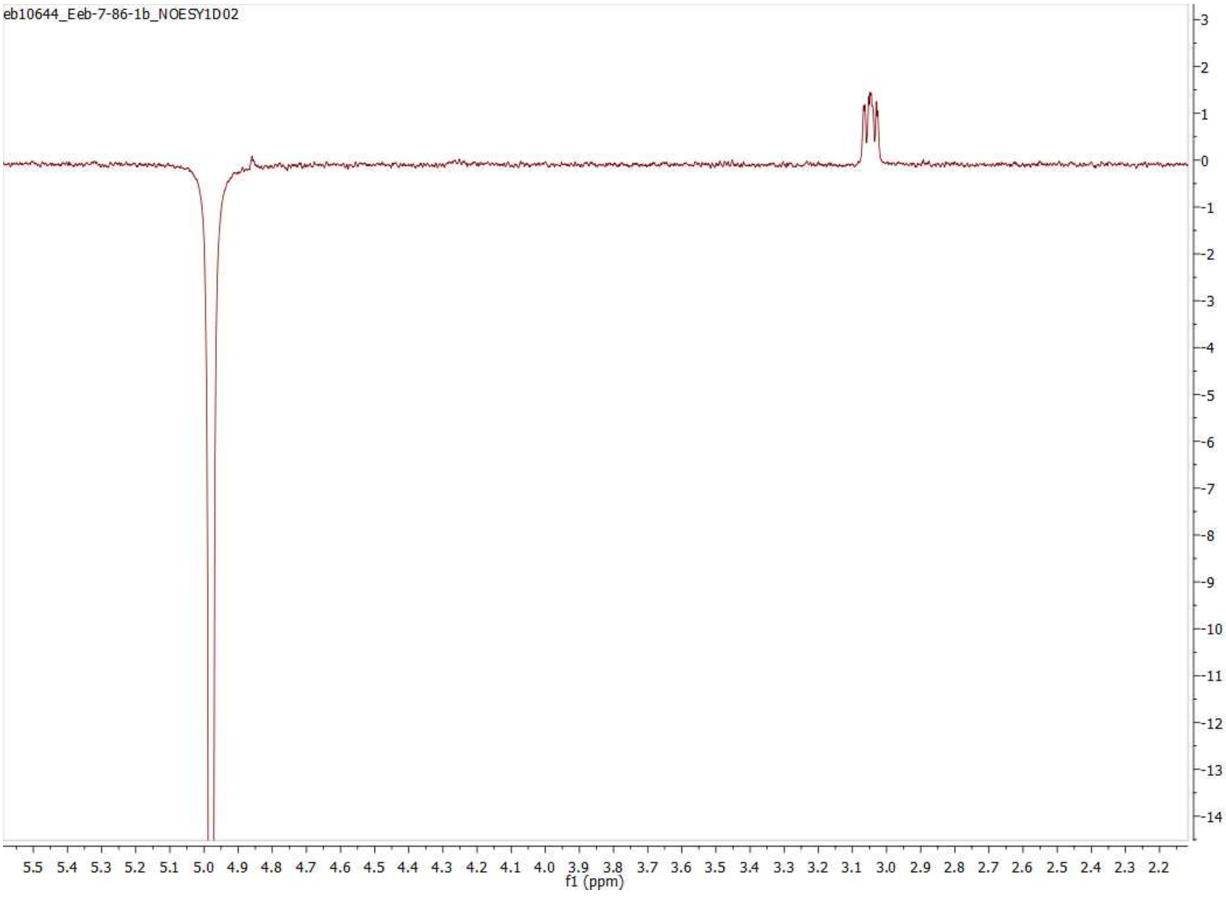

(±)-(3*R*,8a*S*)-2-(4-acetylphenyl)-*N*-ethyl-5,5a,6,8a-tetrahydro-2*H*-oxazolo[5,4,3-hi]indole-3(4*H*)-carboxamide 17a

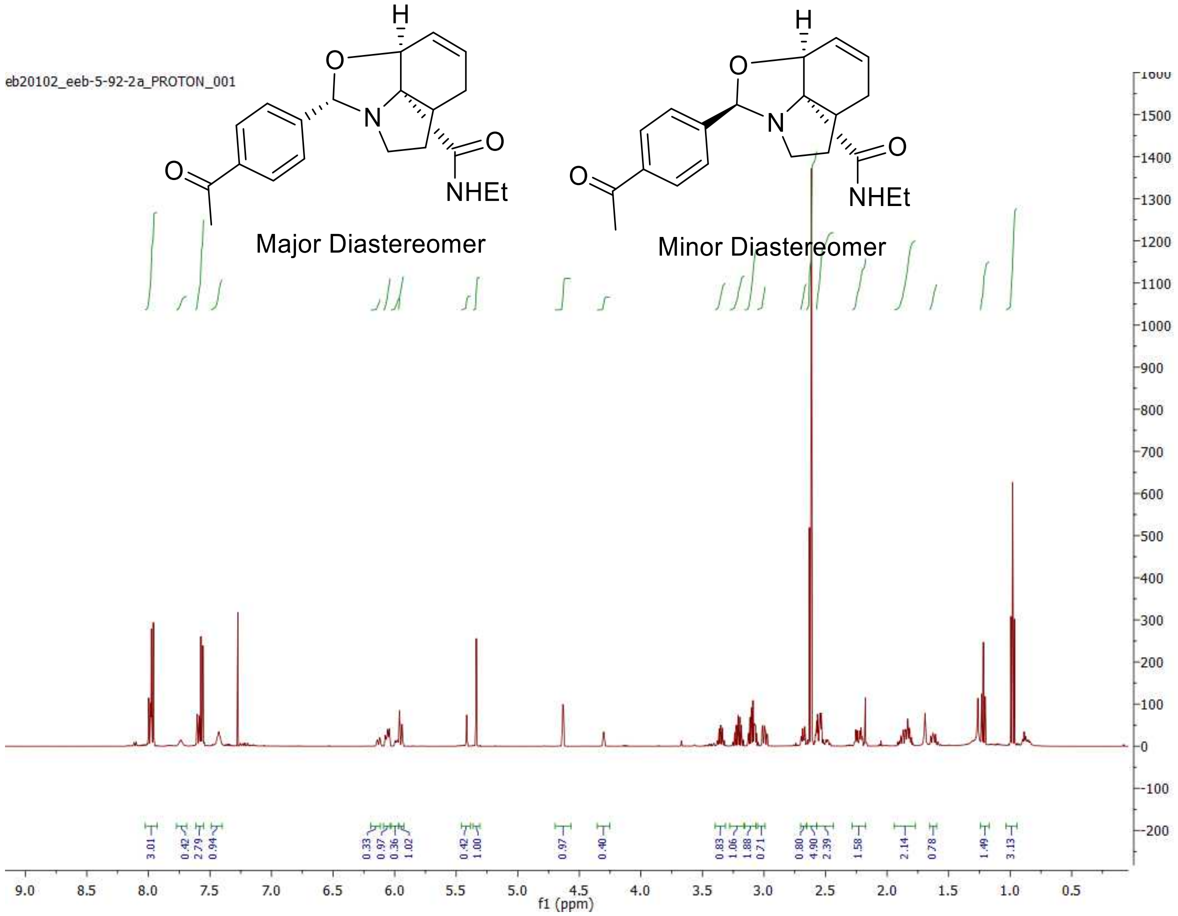

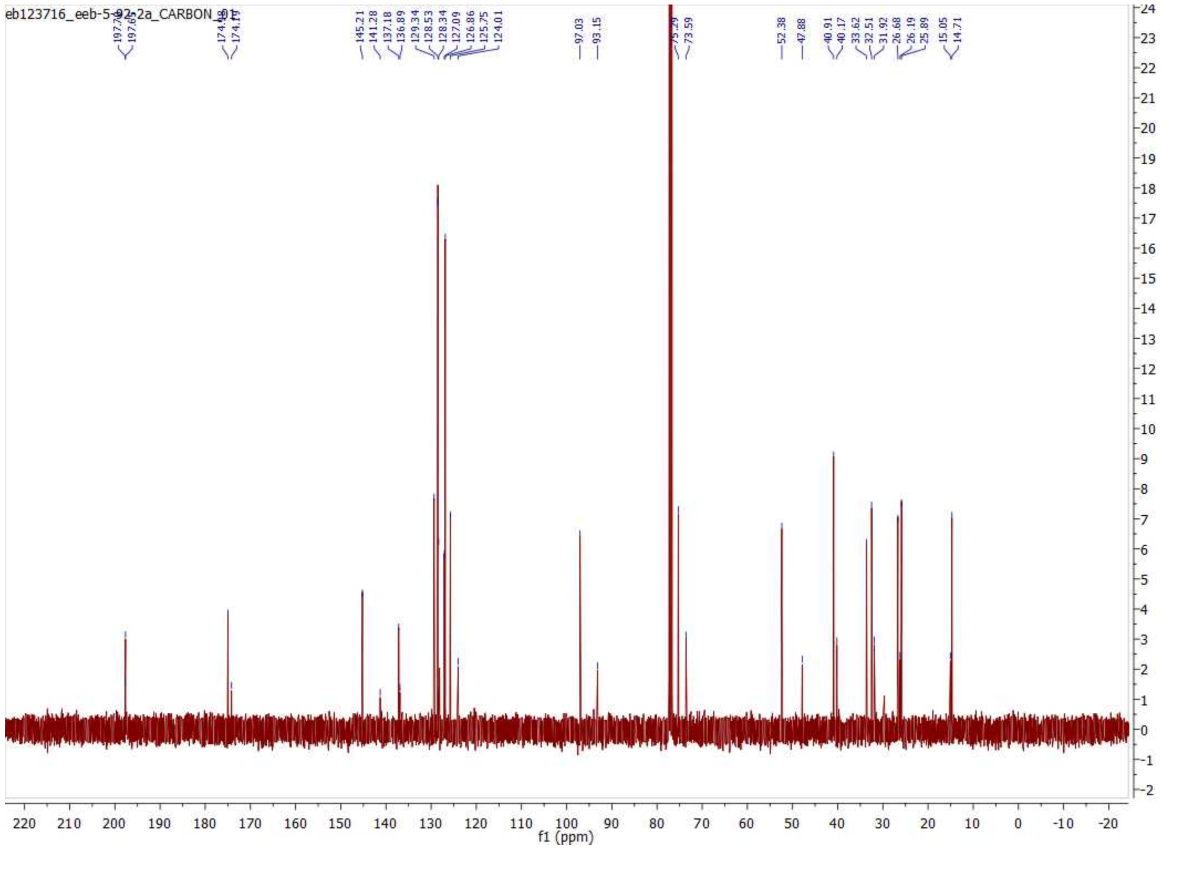

(±)-(3*R*,8a*S*)-2-(4-acetylphenyl)-*N*-ethyl-5,5a,6,8a-tetrahydro-2*H*-oxazolo[5,4,3-hi]indole-3(4*H*)-carboxamide 17a

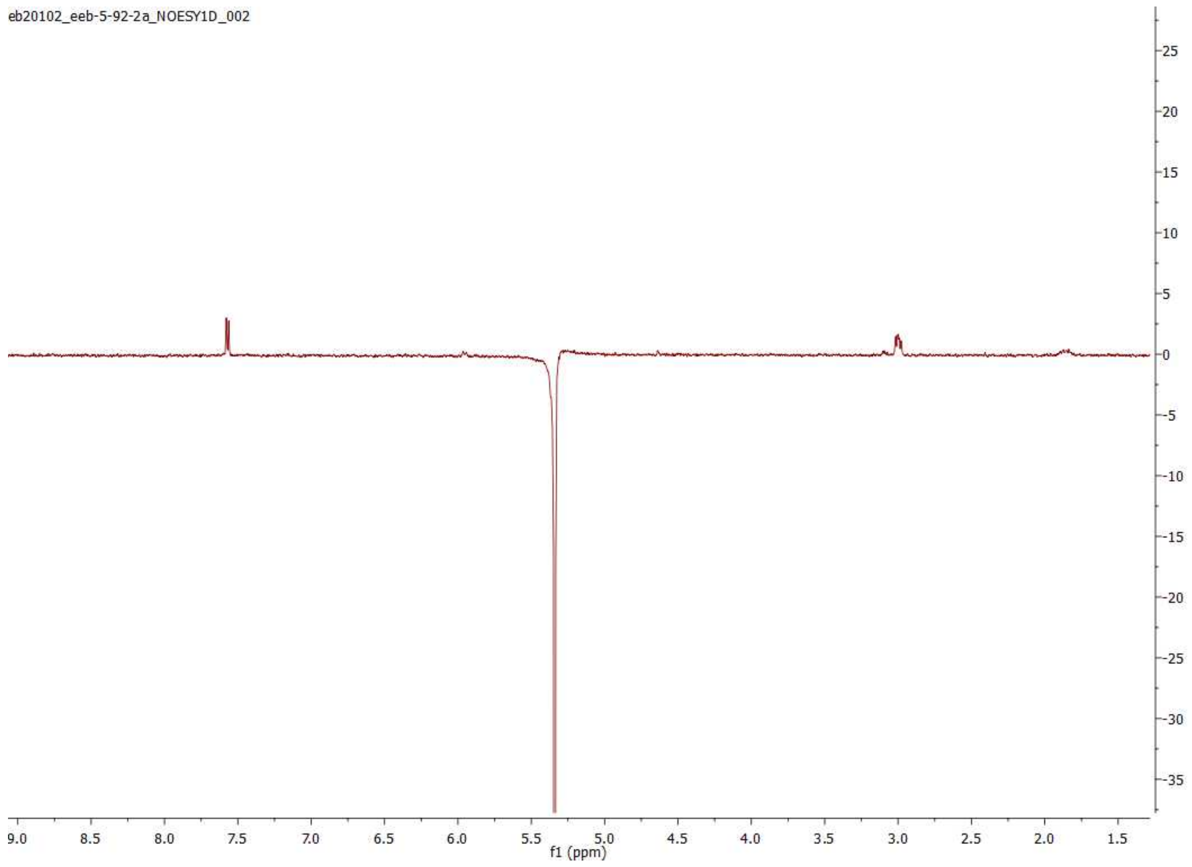

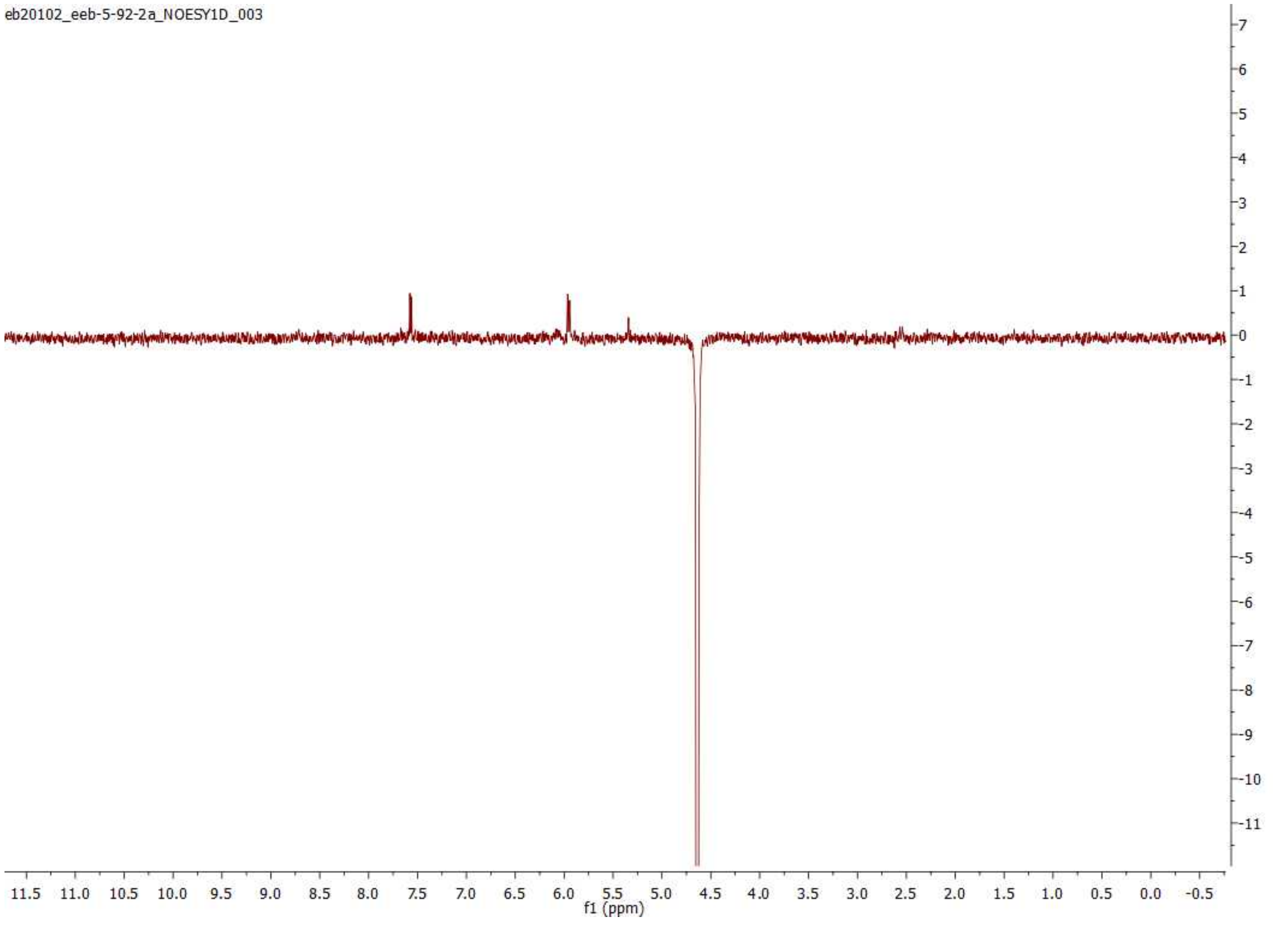

(±)-(3*R*,8a*S*)-2-(4-acetylphenyl)-*N*-ethyl-5,5a,6,8a-tetrahydro-2*H*-oxazolo[5,4,3-hi]indole-3(4*H*)-carboxamide 17a

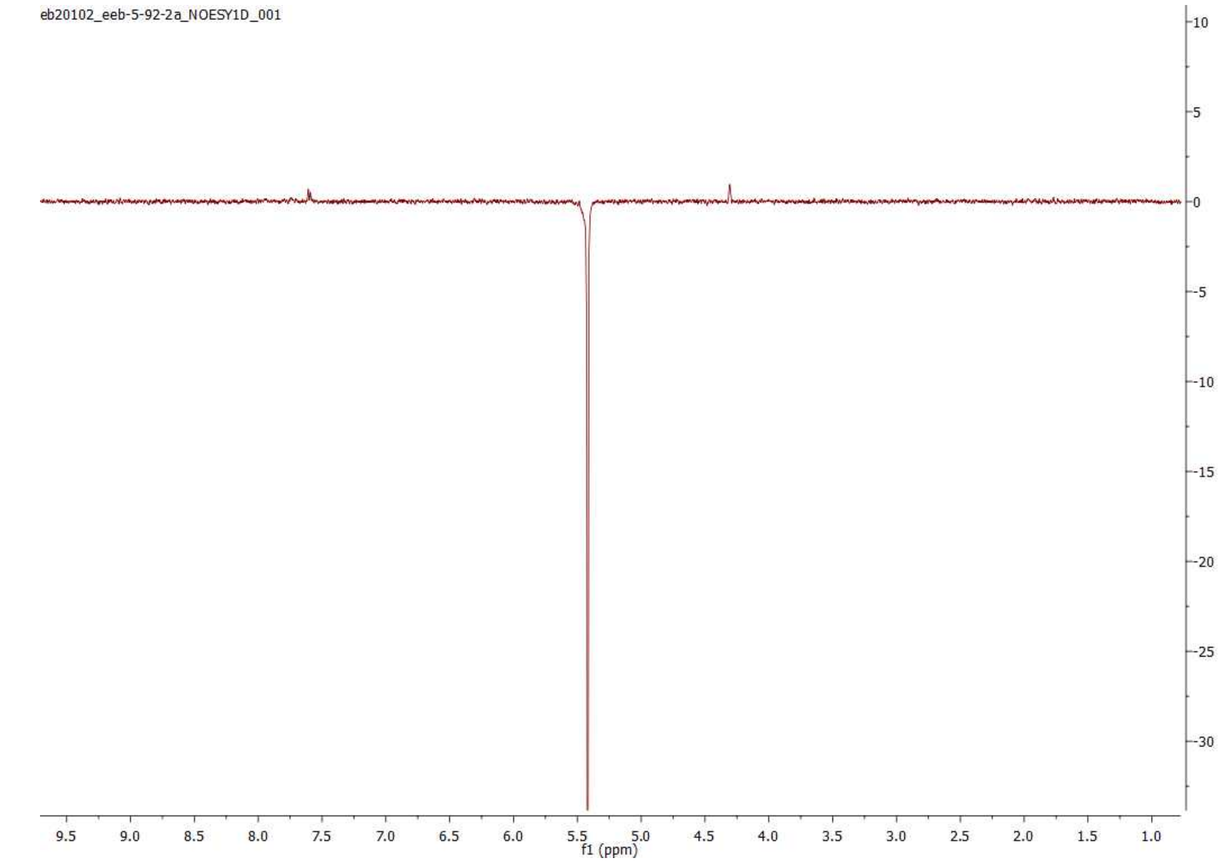

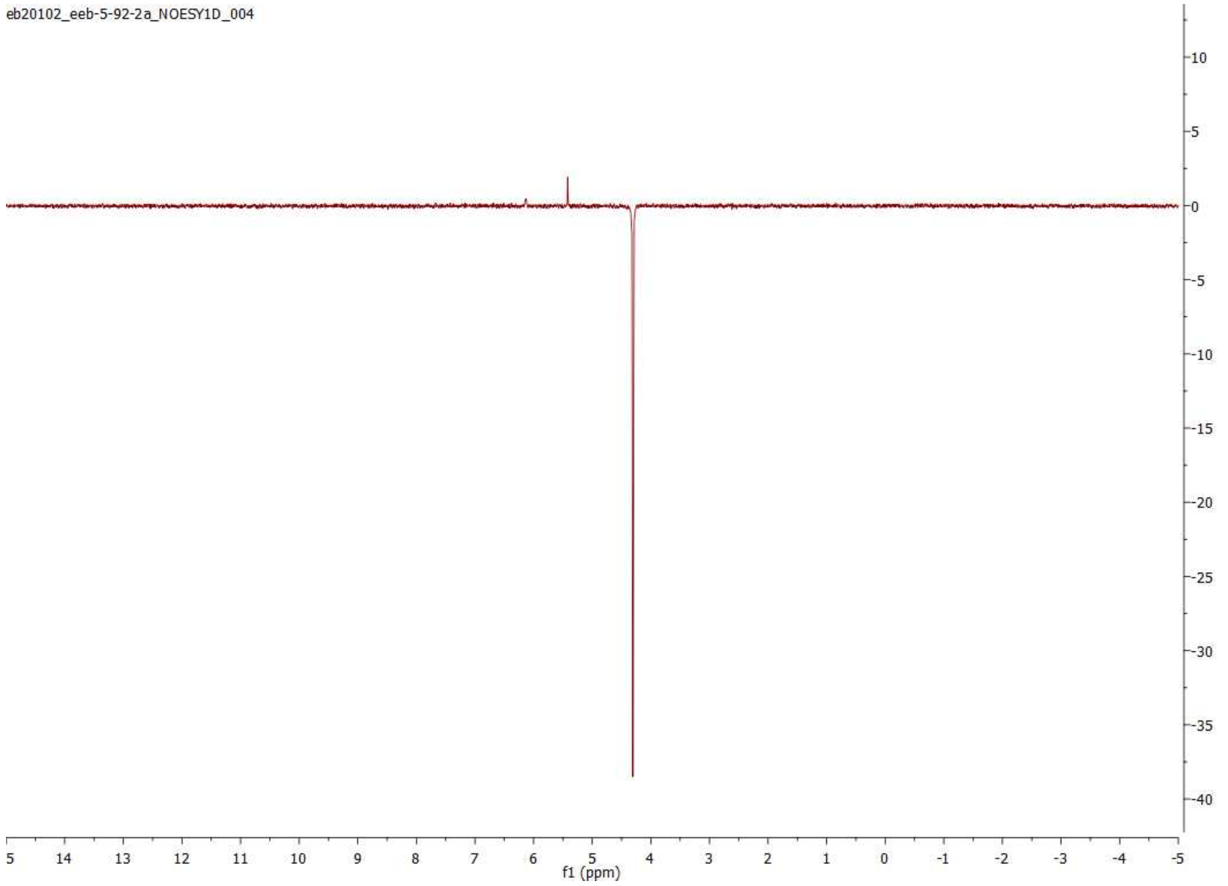

(±)-(3*R*,8a*S*)-*N*-Ethyl-2-(4-nitrophenyl)-5,5a,6,8a-tetrahydro-2*H*-oxazolo[5,4,3-hi]indole-3(4*H*)-carboxamide 18a

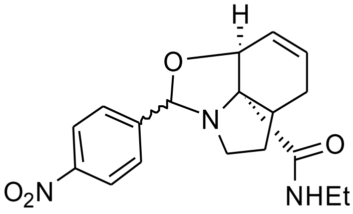

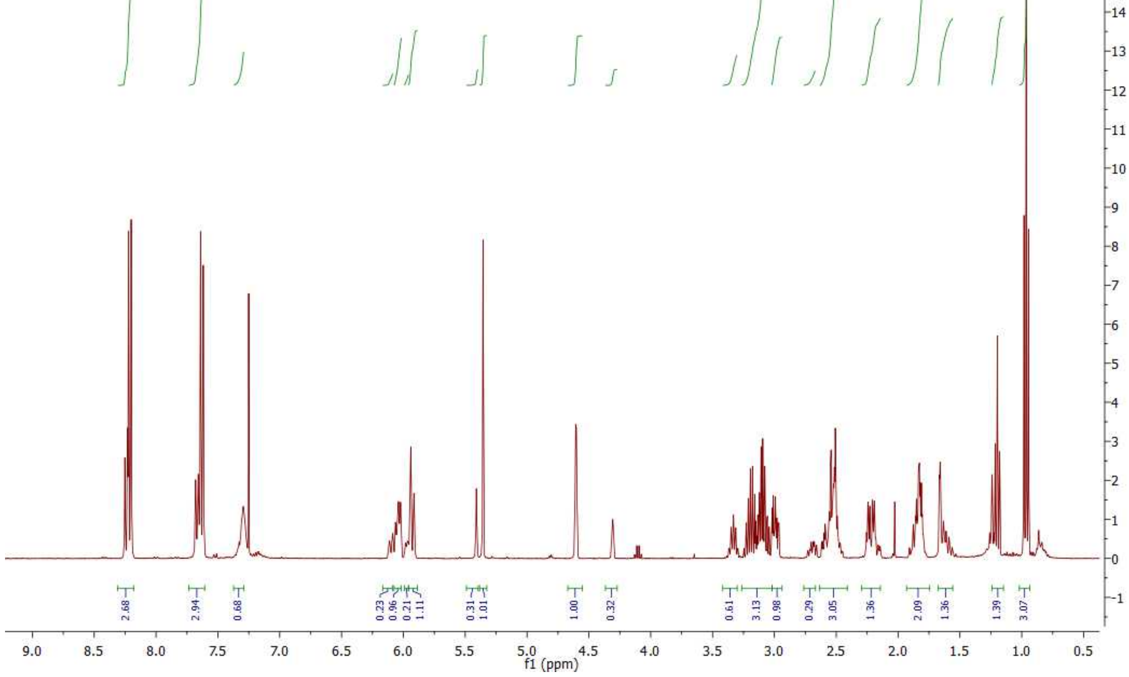

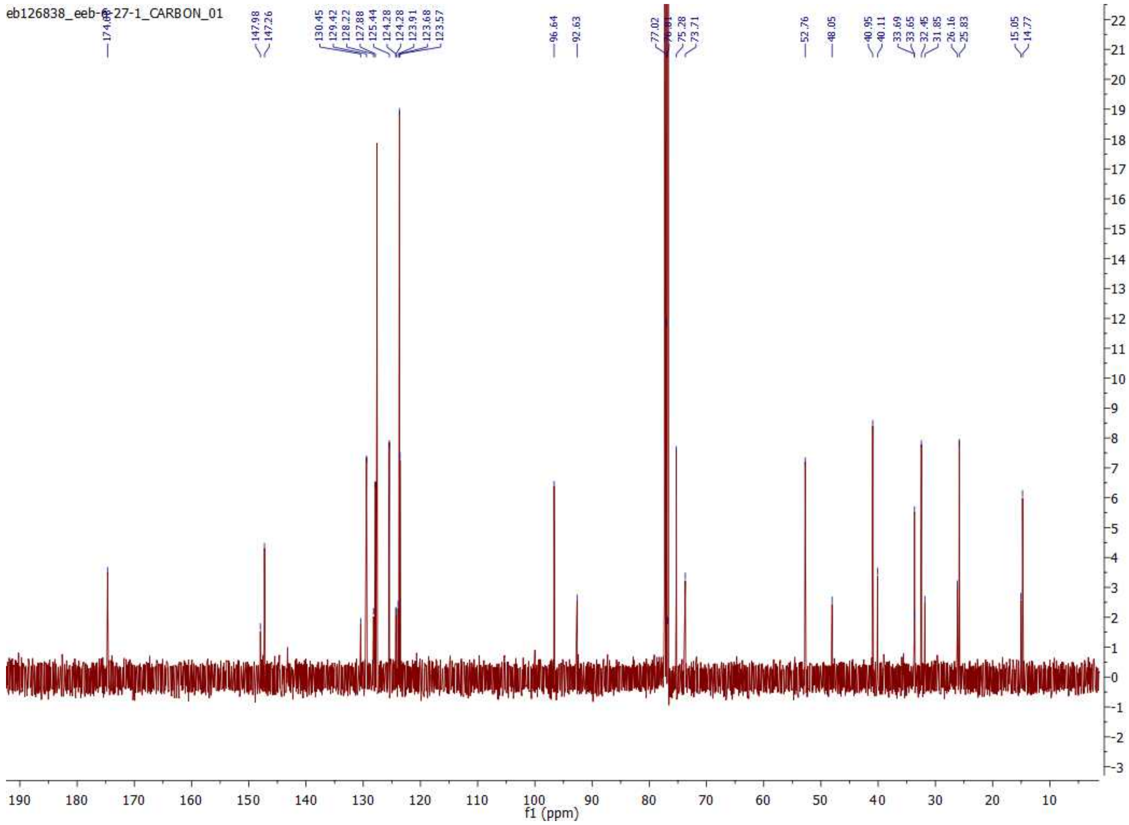

(±)-(3*R*,8a*S*)-*N*-Ethyl-2-(4-nitrophenyl)-5,5a,6,8a-tetrahydro-2*H*-oxazolo[5,4,3-hi]indole-3(4*H*)-carboxamide 18a

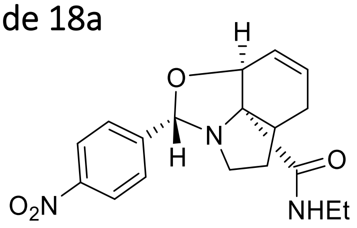

Major

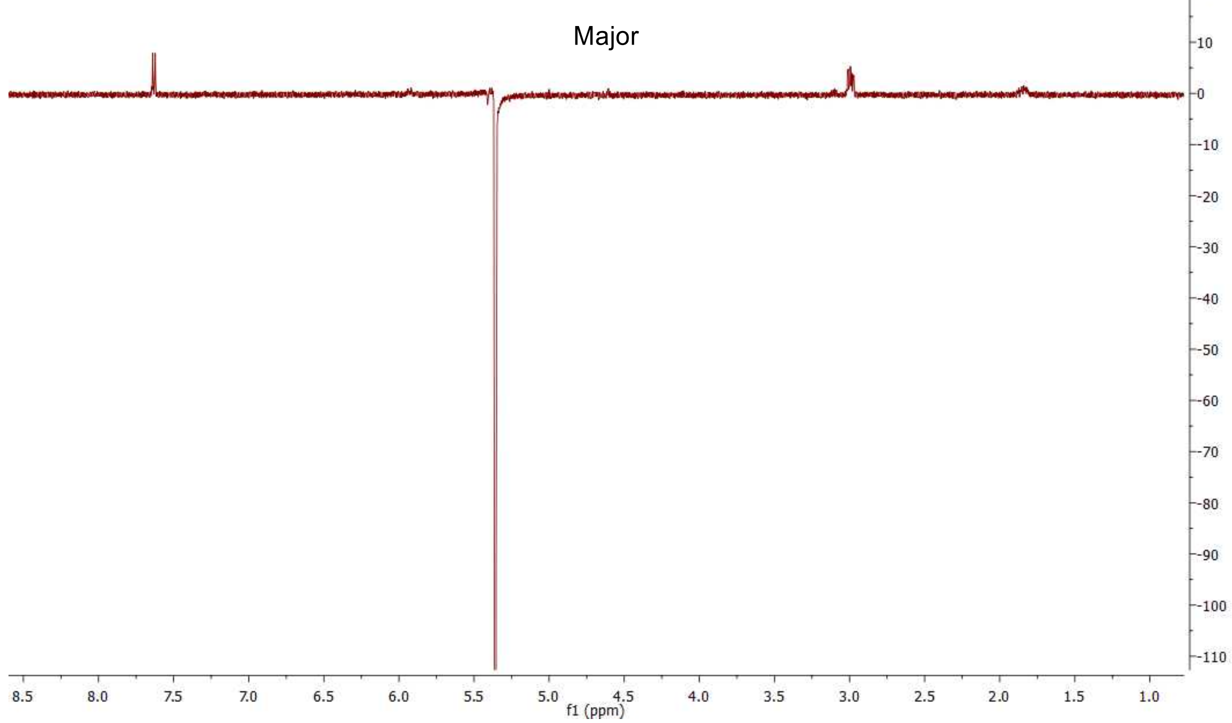

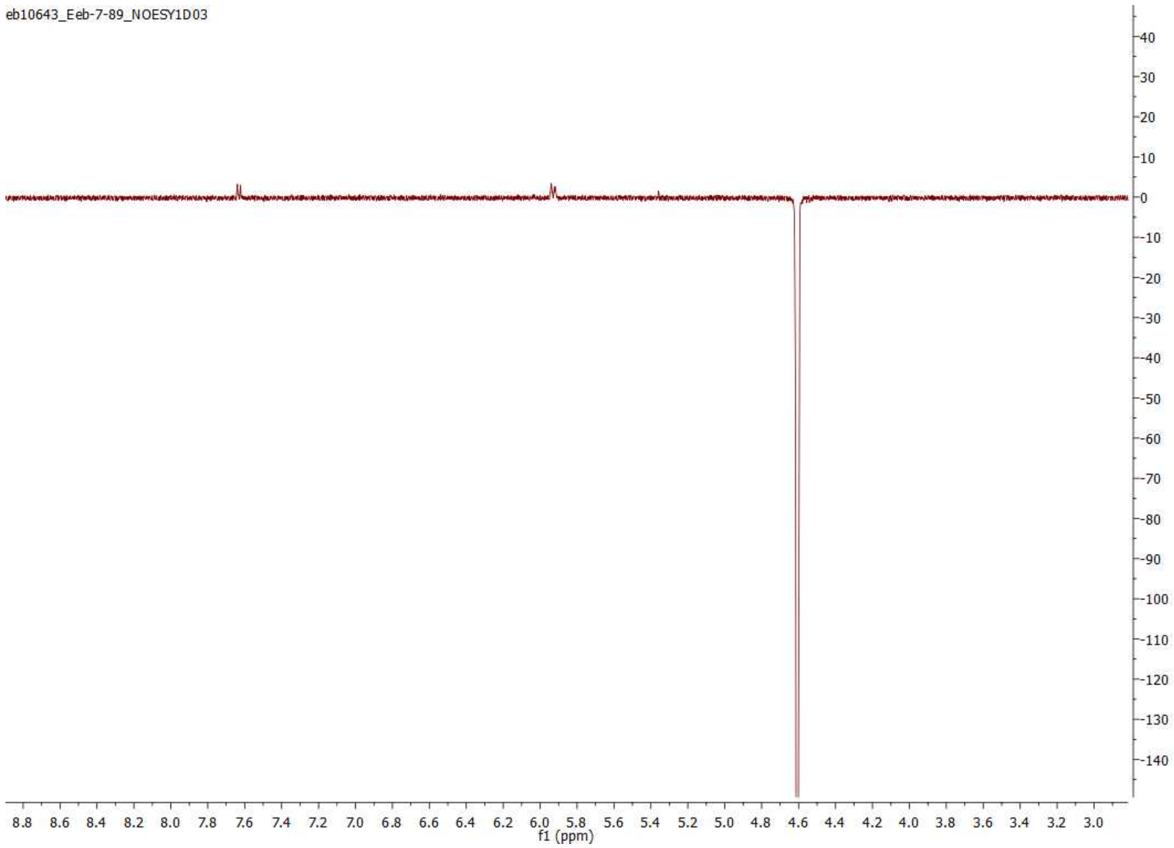

(±)-(3*R*,8a*S*)-*N*-Ethyl-2-(4-nitrophenyl)-5,5a,6,8a-tetrahydro-2*H*-oxazolo[5,4,3-hi]indole-3(4*H*)-carboxamide 18a

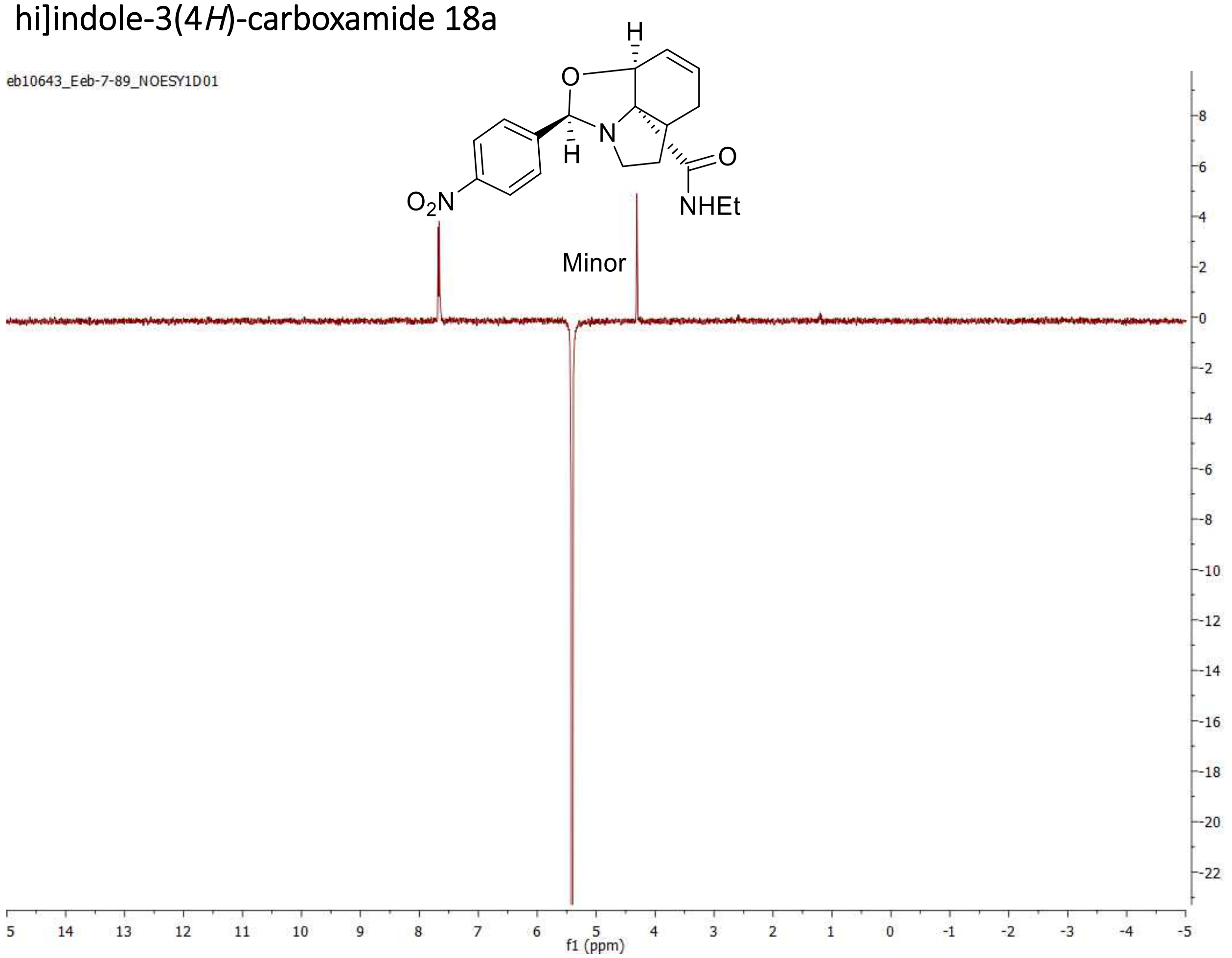

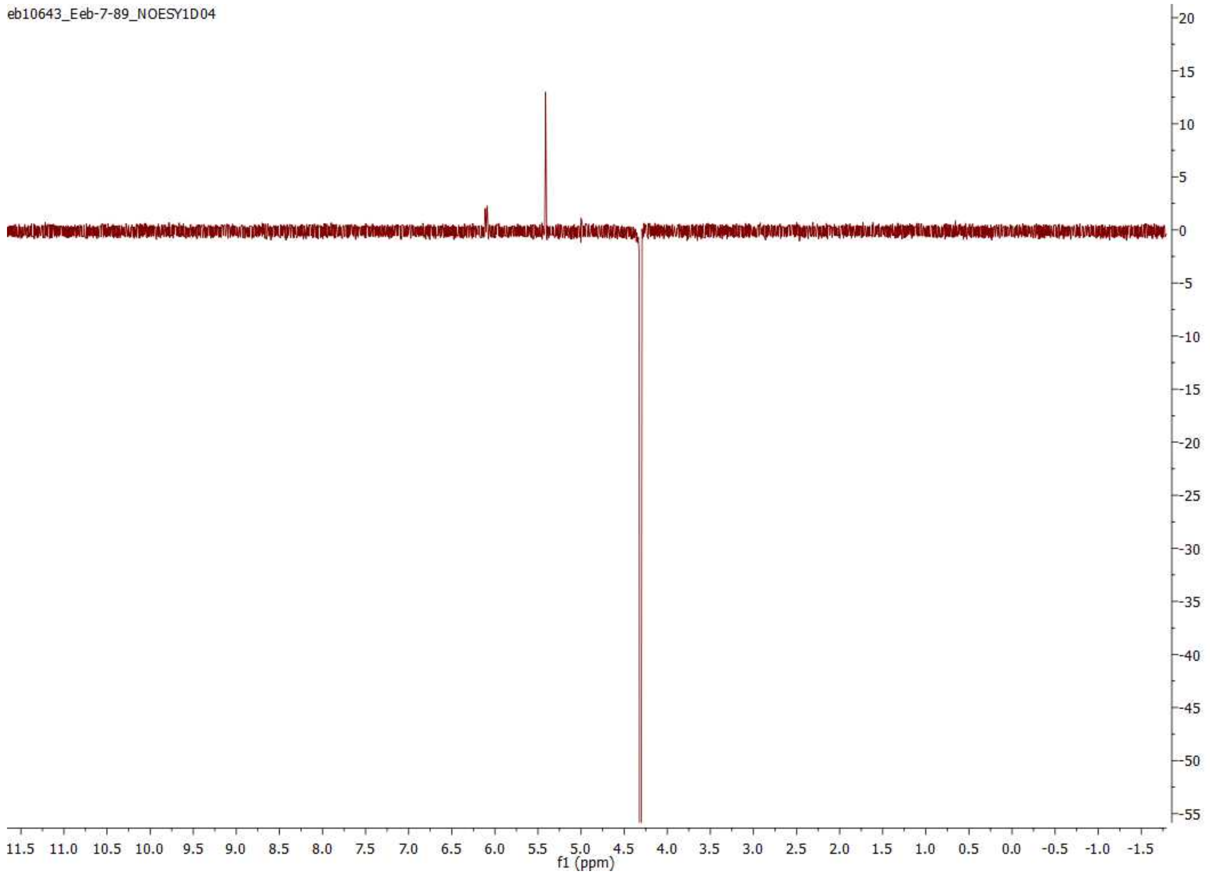

(±)-(2*S*,3*R*,8a*S*)-*N*-ethyl-2-methyl-1-tosyl-1,4,5,5a,6,8a-hexahydroimidazo[4,5,1-hi]indole-3(2*H*)-carboxamide – major diastereomer 19a

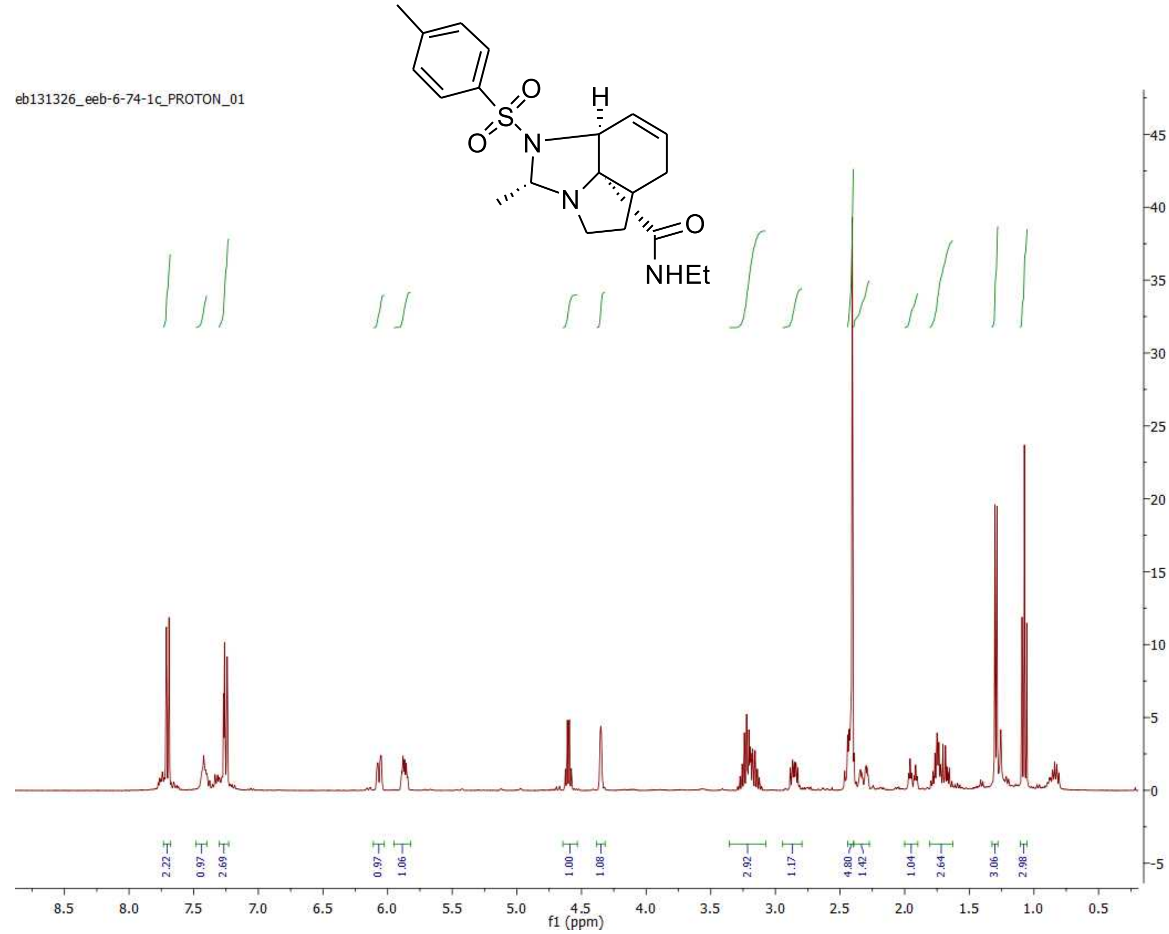

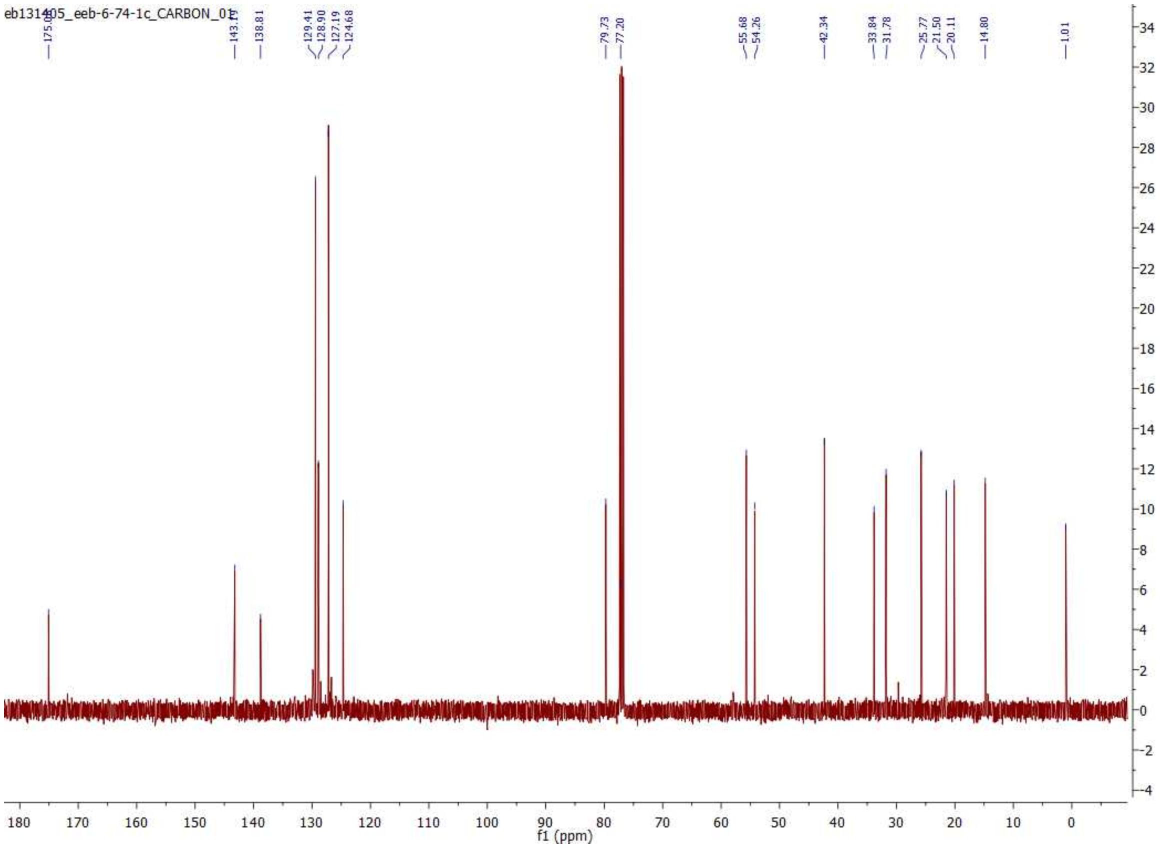

(±)-(2*S*,3*R*,8a*S*)-*N*-ethyl-2-methyl-1-tosyl-1,4,5,5a,6,8a-hexahydroimidazo[4,5,1-hi]indole-3(2*H*)-carboxamide – major diastereomer 19a

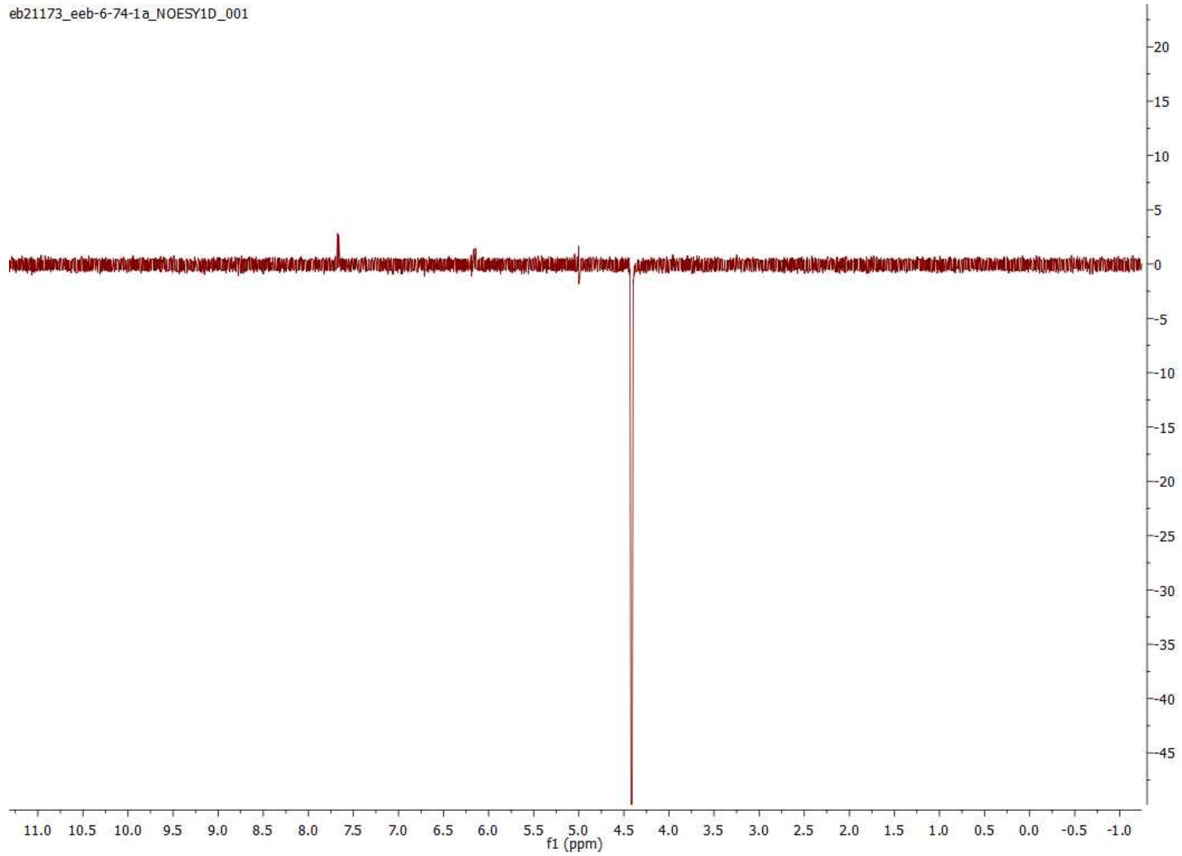

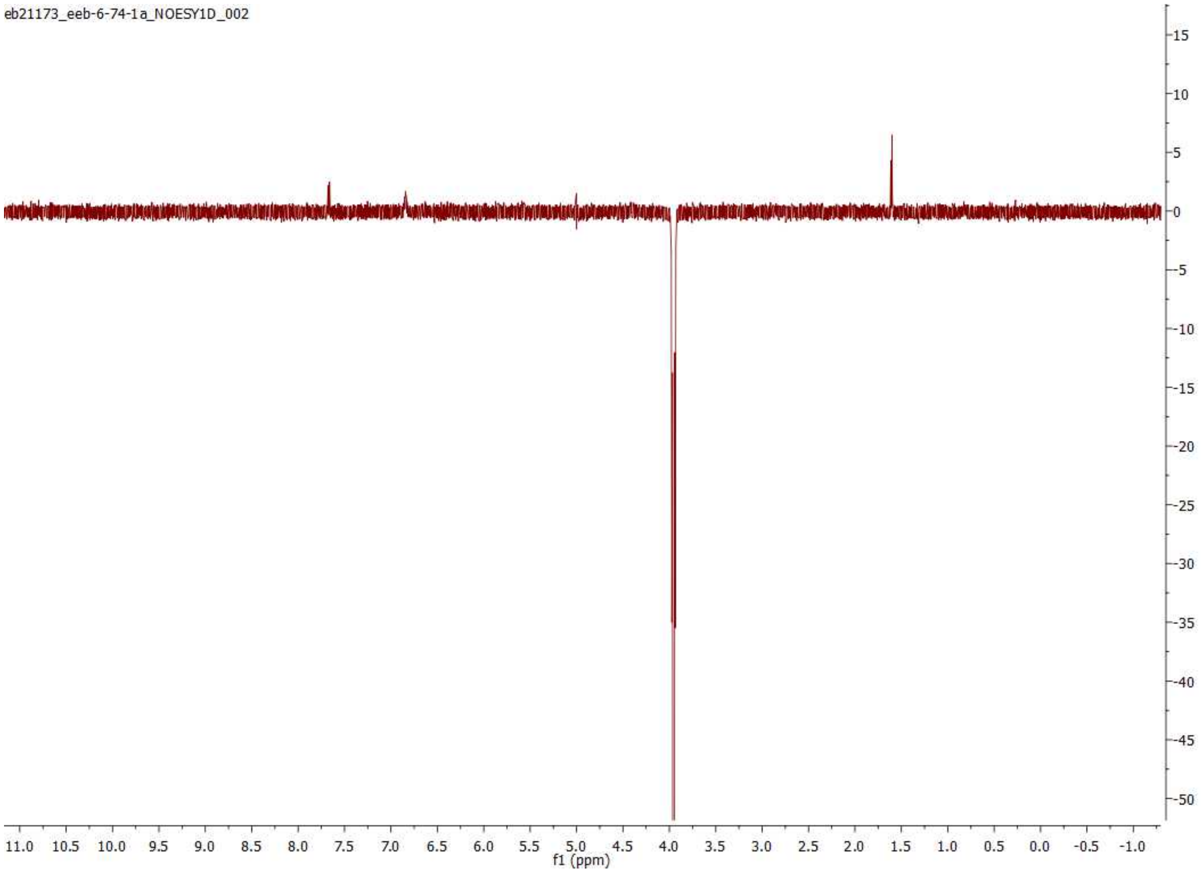

(±)-(2*R*,3*R*,8a*S*)-*N*-ethyl-2-methyl-1-tosyl-1,4,5,5a,6,8a-hexahydroimidazo[4,5,1-hi]indole-3(2*H*)-carboxamide Minor diastereomer19a

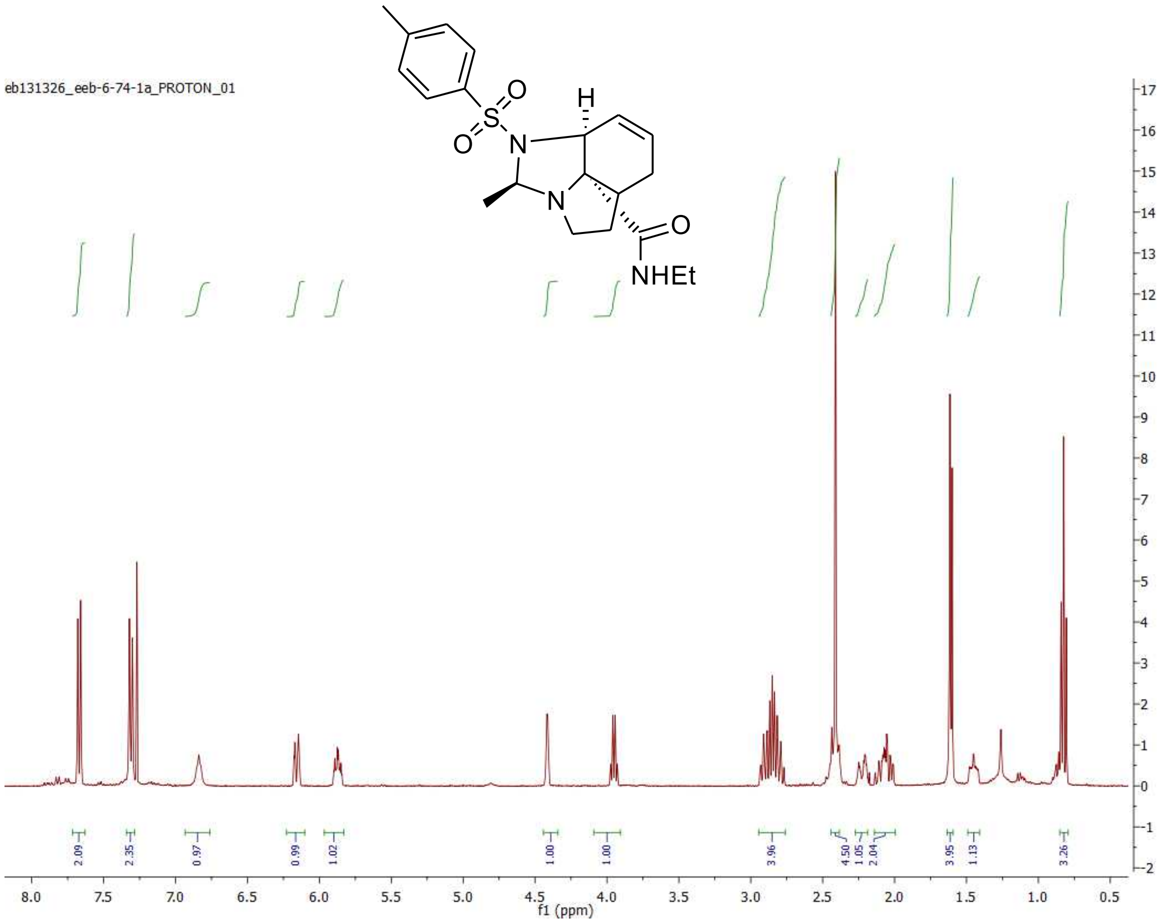

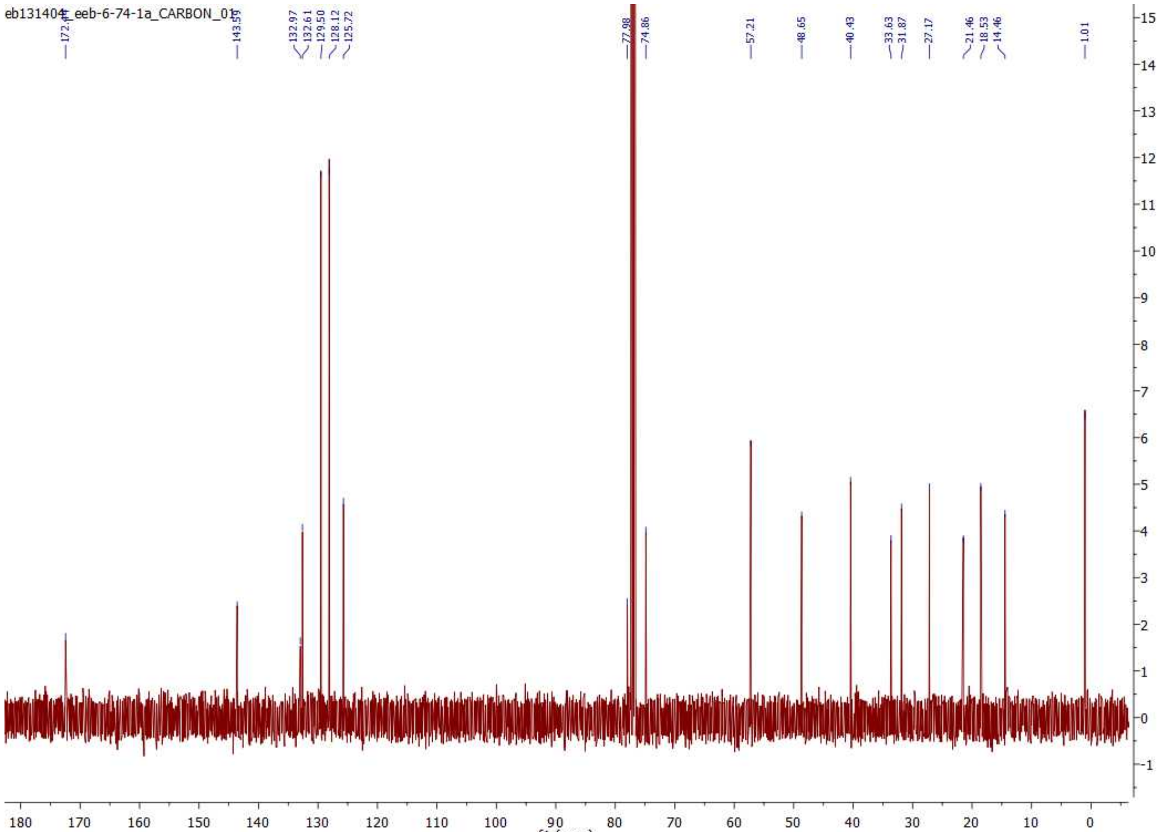

(±)-(2*R*,3*R*,8a*S*)-*N*-ethyl-2-methyl-1-tosyl-1,4,5,5a,6,8a-hexahydroimidazo[4,5,1-hi]indole-3(2*H*)-carboxamide Minor diastereomer19a

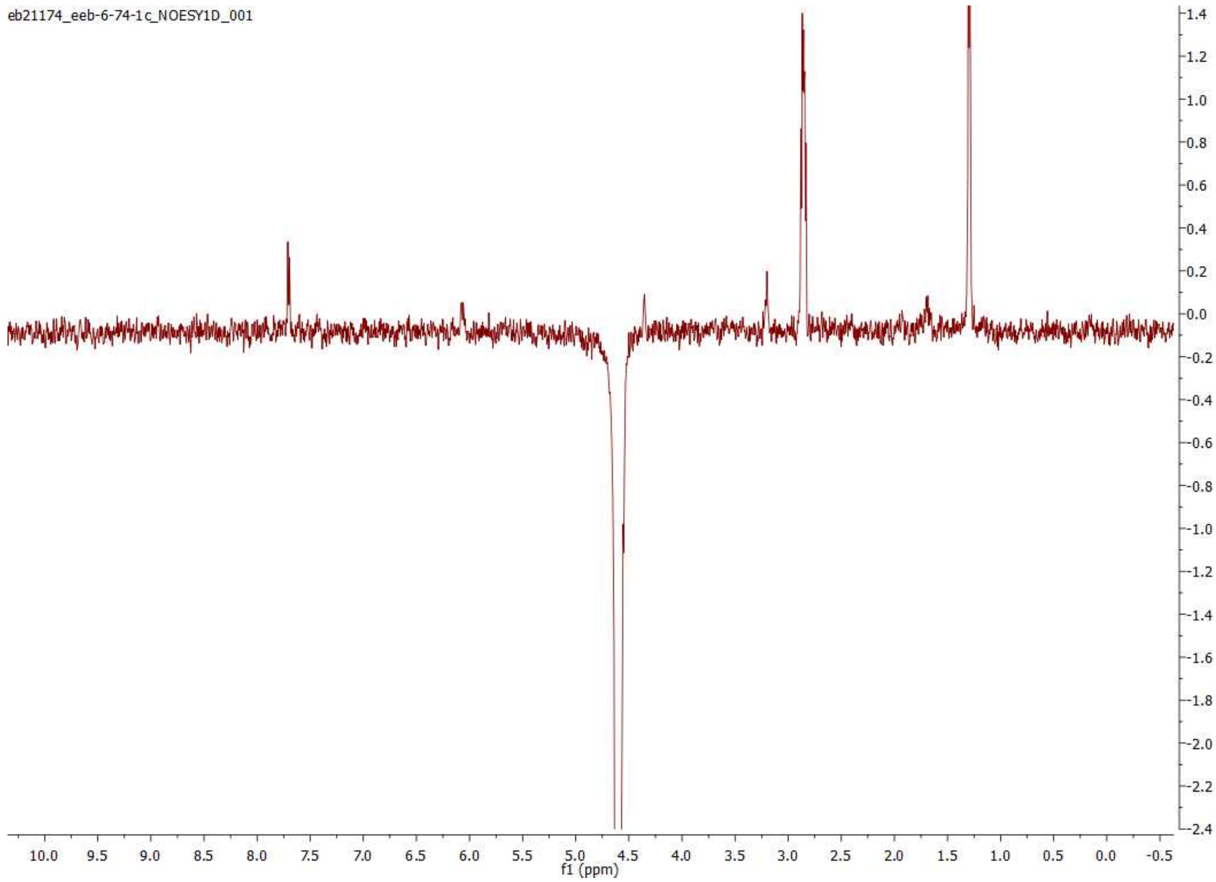

(±)-(3*R*,8a*S*)-*N*-ethyl-2-(4-nitrophenyl)-1-tosyl-1,4,5,5a,6,8a-hexahydroimidazo[4,5,1-hi]indole-3(2*H*)-carboxamide 20a

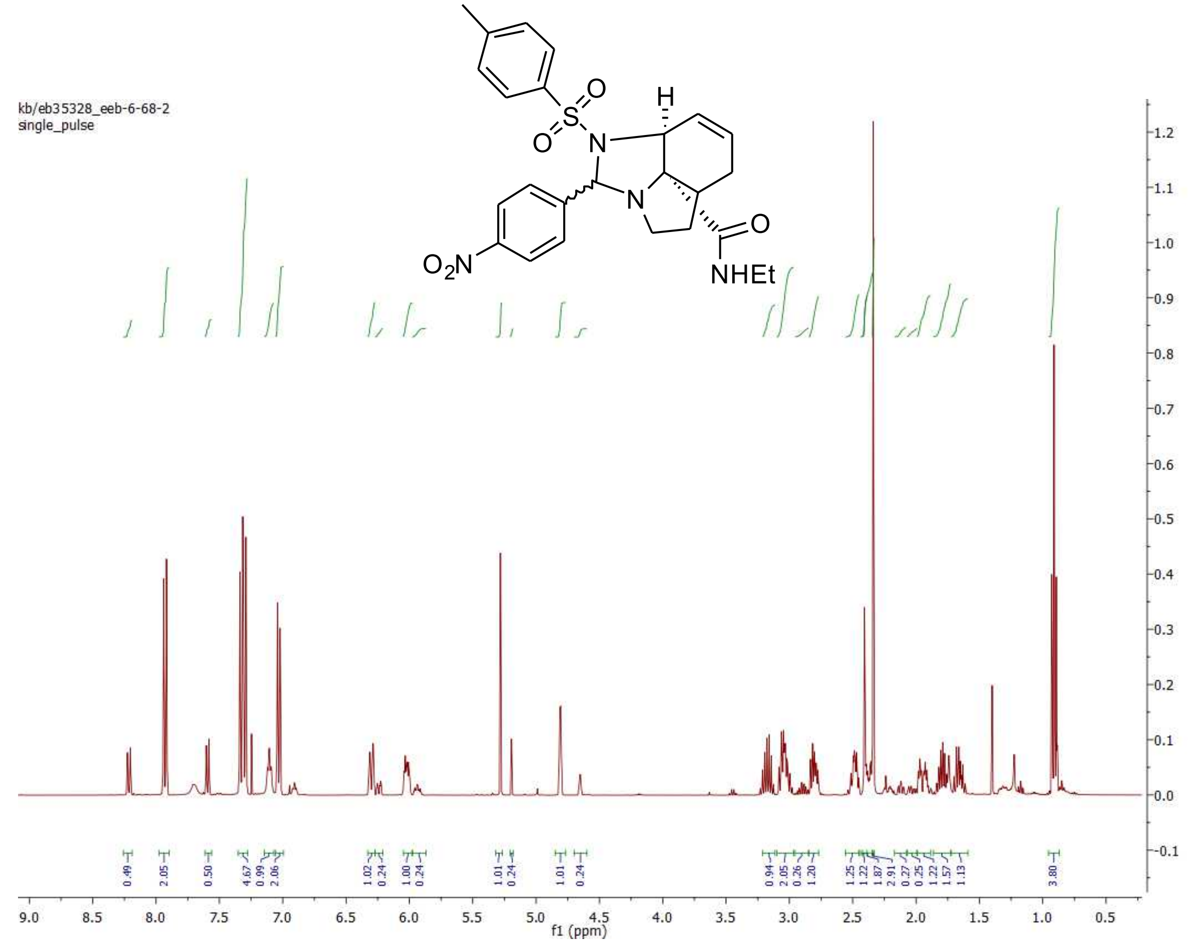

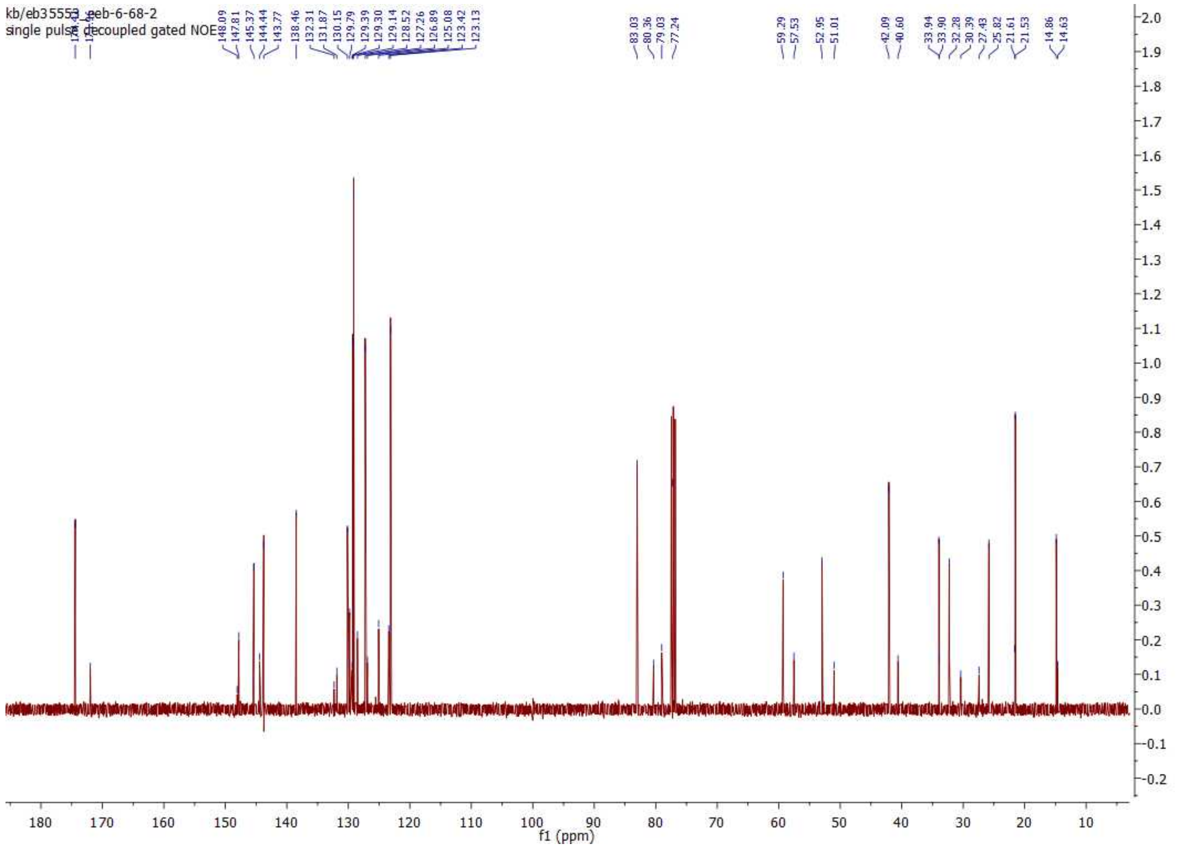

(±)-(3*R*,8a*S*)-*N*-ethyl-2-(4-nitrophenyl)-1-tosyl-1,4,5,5a,6,8a-hexahydroimidazo[4,5,1-hi]indole-3(2*H*)-carboxamide major diastereomer 20a

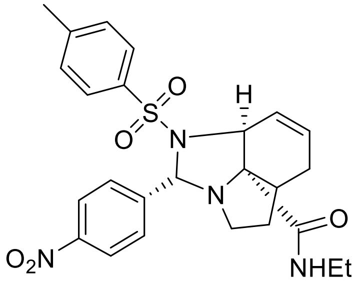

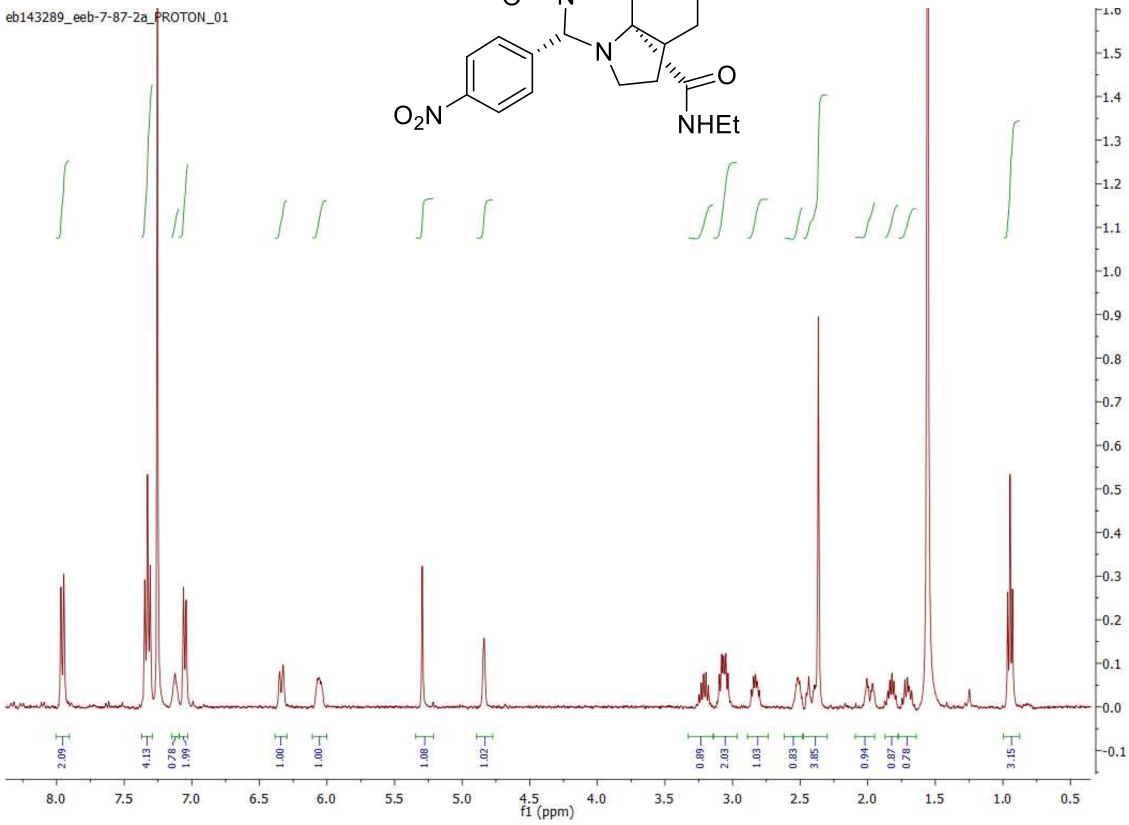

(±)-(2*S*,3*R*,8a*S*)-*N*,2-diethyl-1-tosyl-1,4,5,5a,6,8a-hexahydroimidazo[4,5,1-hi]indole-3(2*H*)-carboxamide Major diastereomer 21a

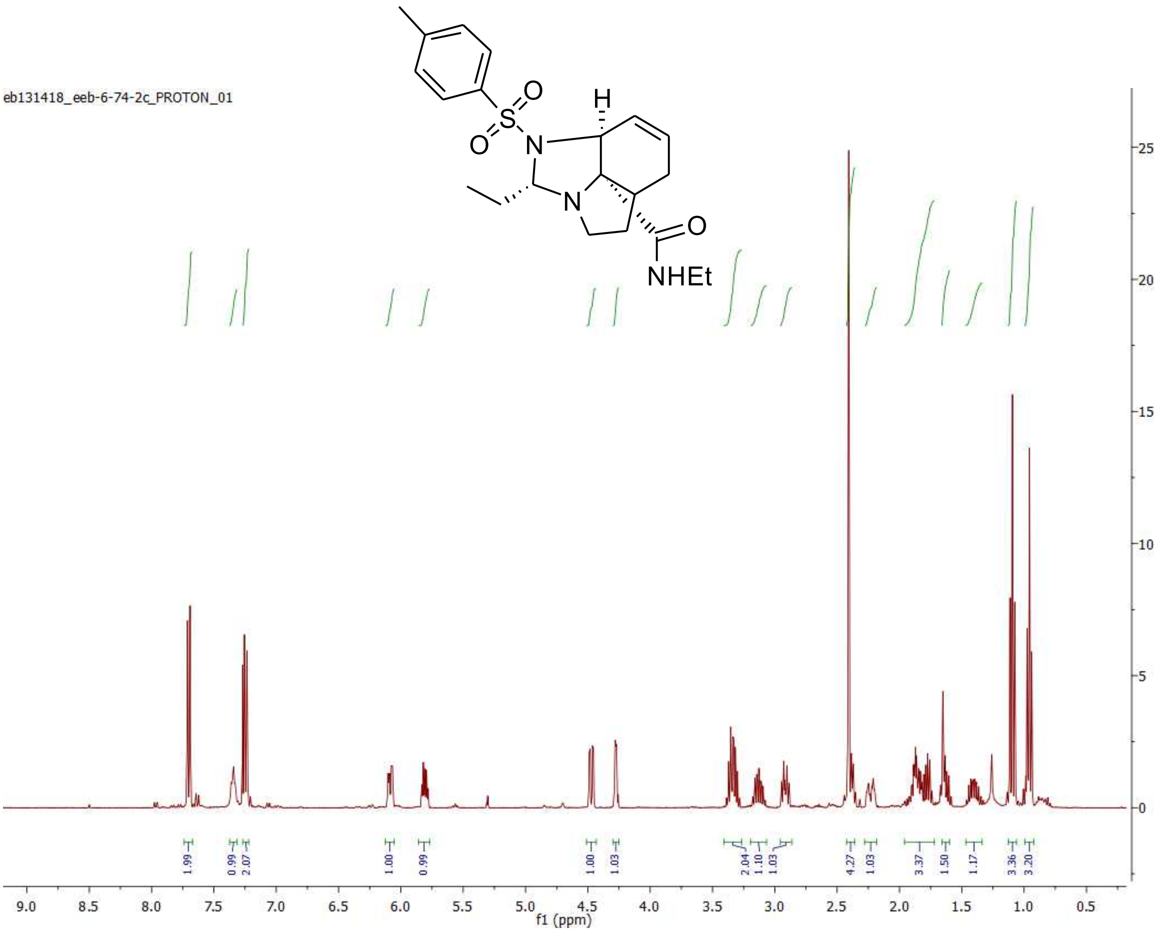

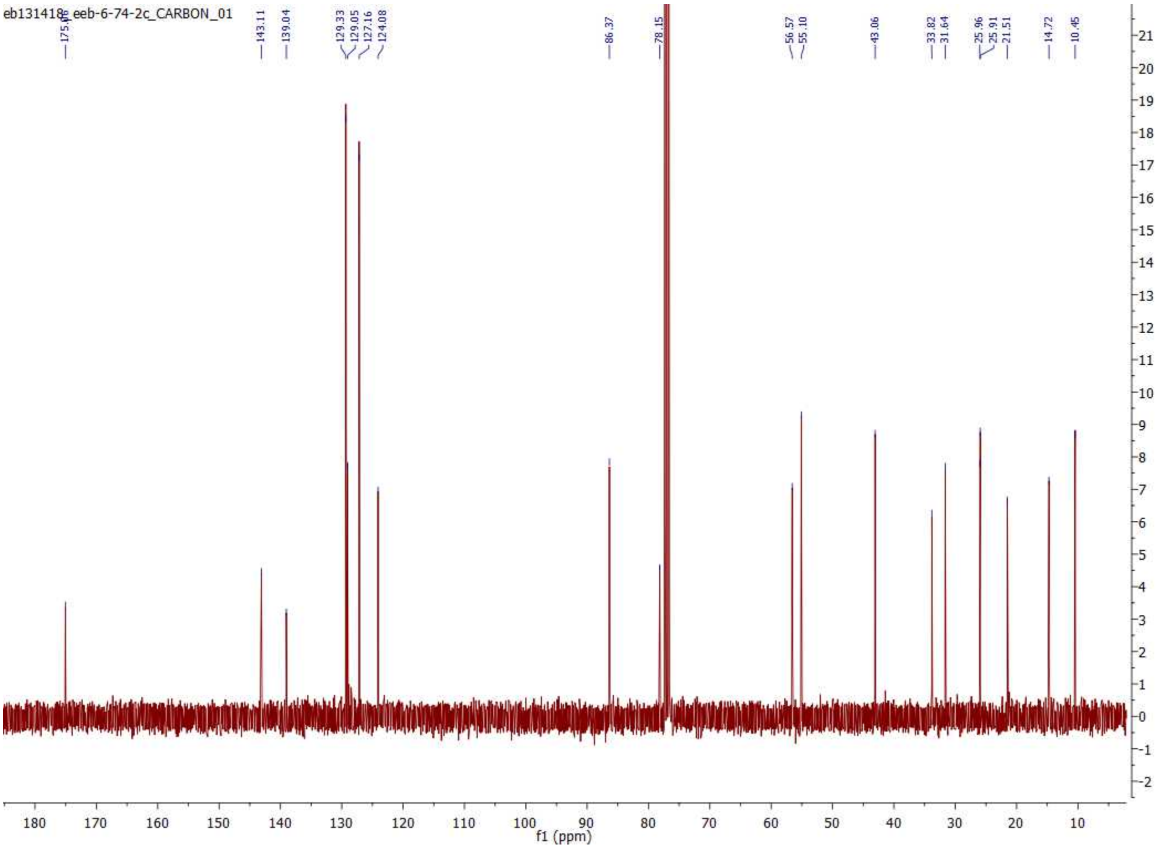

(±)-(2*S*,3*R*,8a*S*)-*N*,2-diethyl-1-tosyl-1,4,5,5a,6,8a-hexahydroimidazo[4,5,1-hi]indole-3(2*H*)-carboxamide Major diastereomer 21a

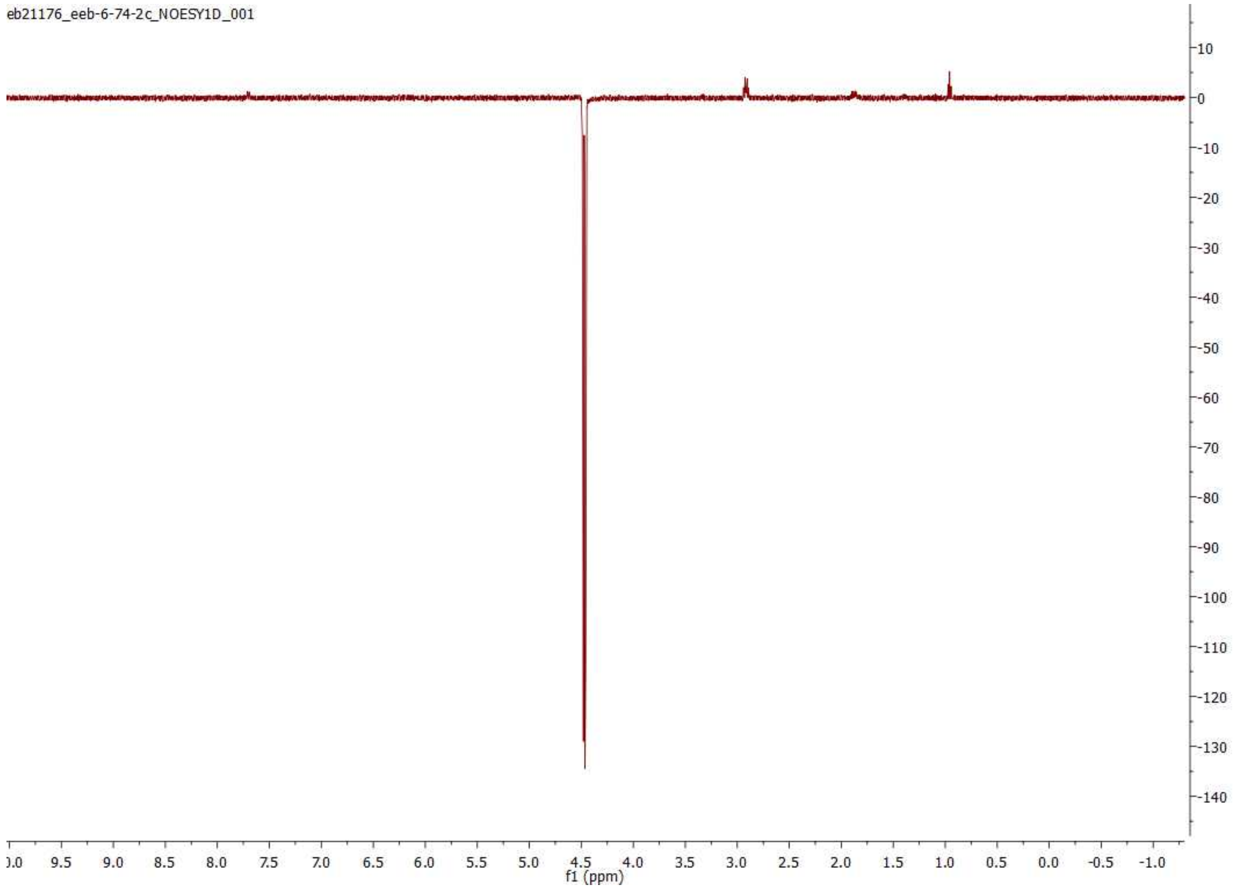

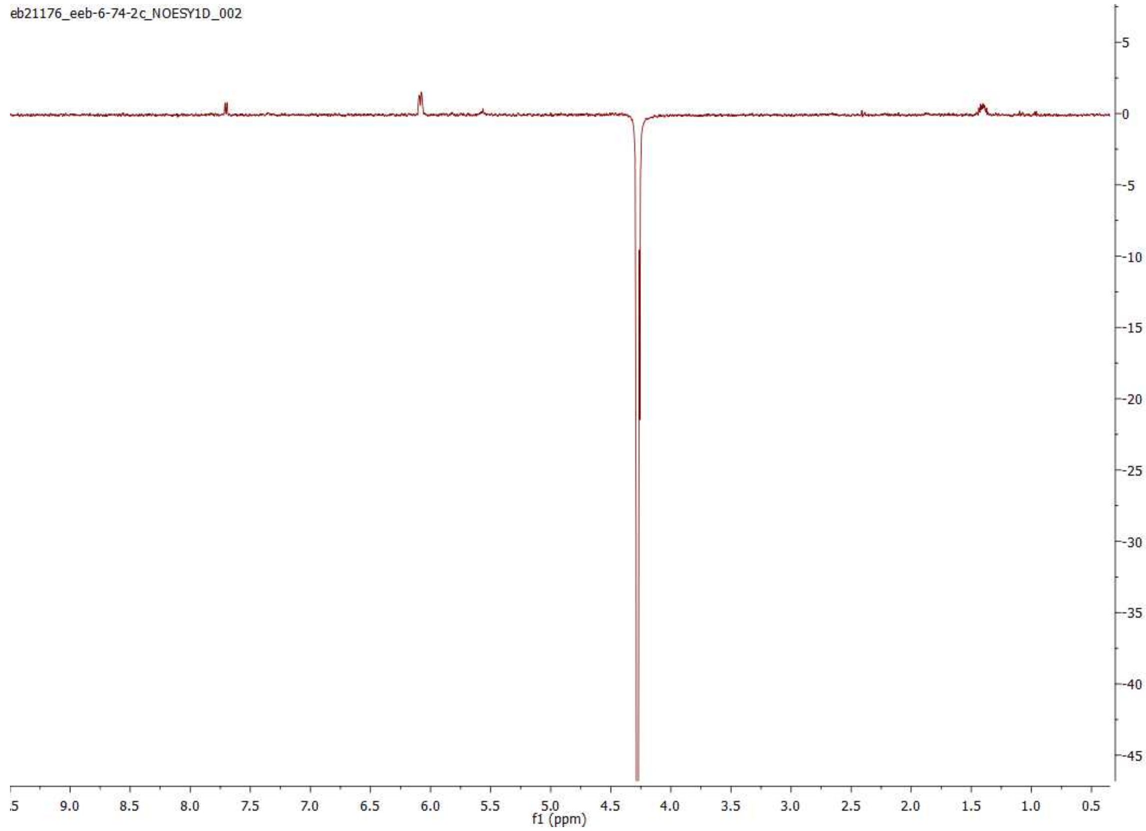

(±)- (2*R*,3*R*,8a*S*)-*N*,2-diethyl-1-tosyl-1,4,5,5a,6,8a-hexahydroimidazo[4,5,1-hi]indole-3(2*H*)-carboxamide minor diastereomer 21a

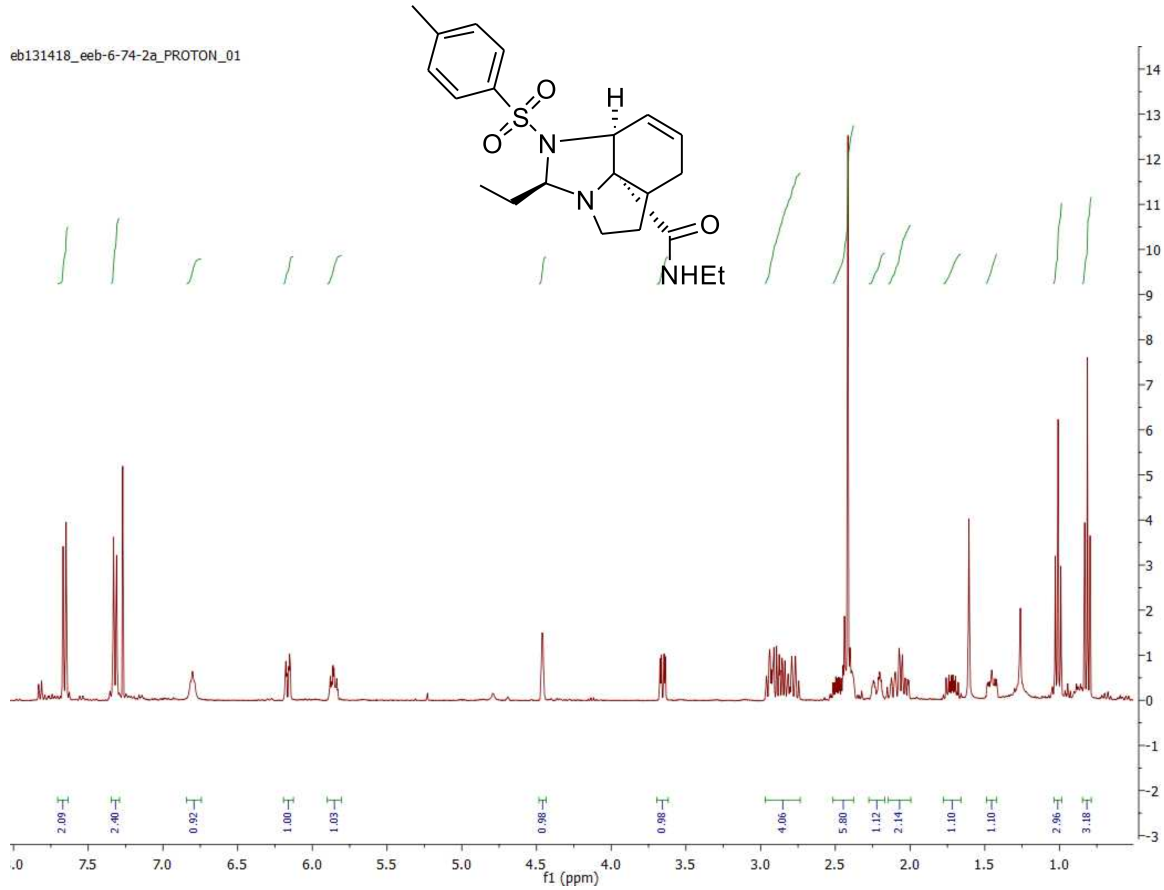

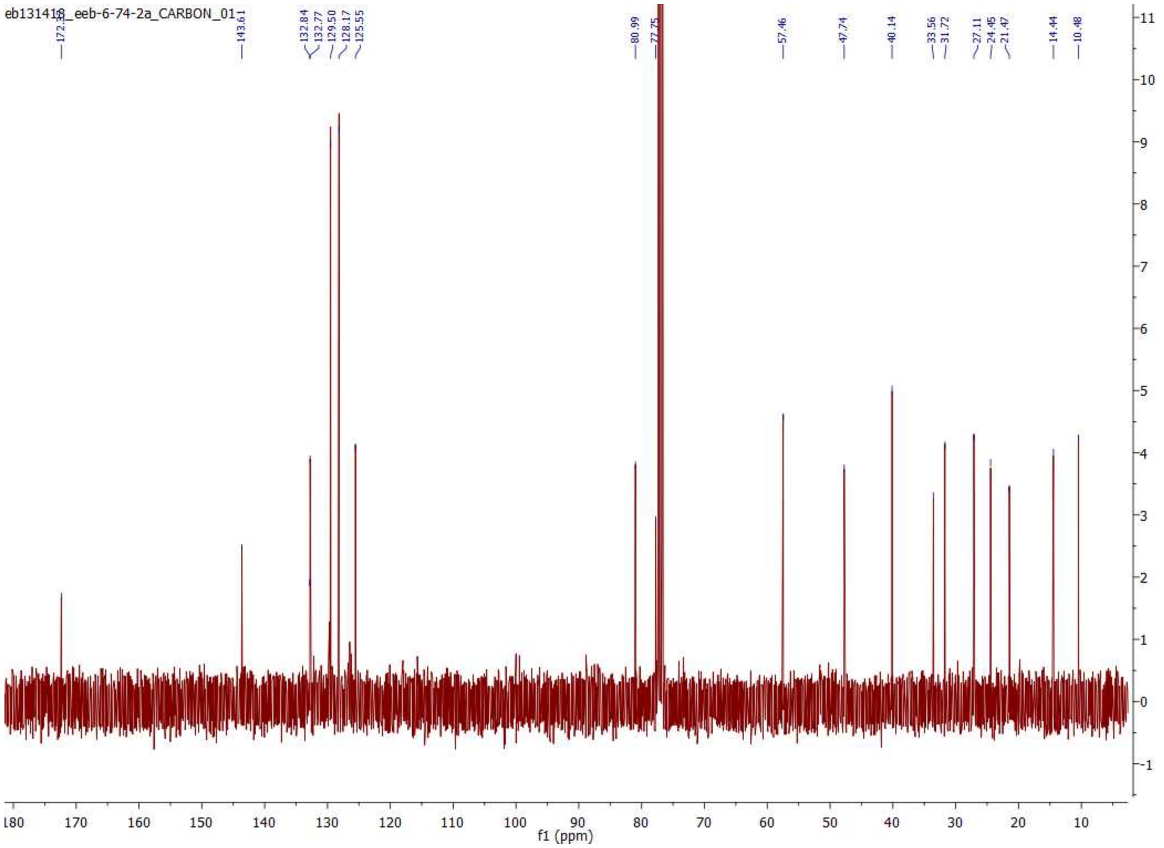

(±)- (2*R*,3*R*,8a*S*)-*N*,2-diethyl-1-tosyl-1,4,5,5a,6,8a-hexahydroimidazo[4,5,1-hi]indole-3(2*H*)-carboxamide minor diastereomer 21a

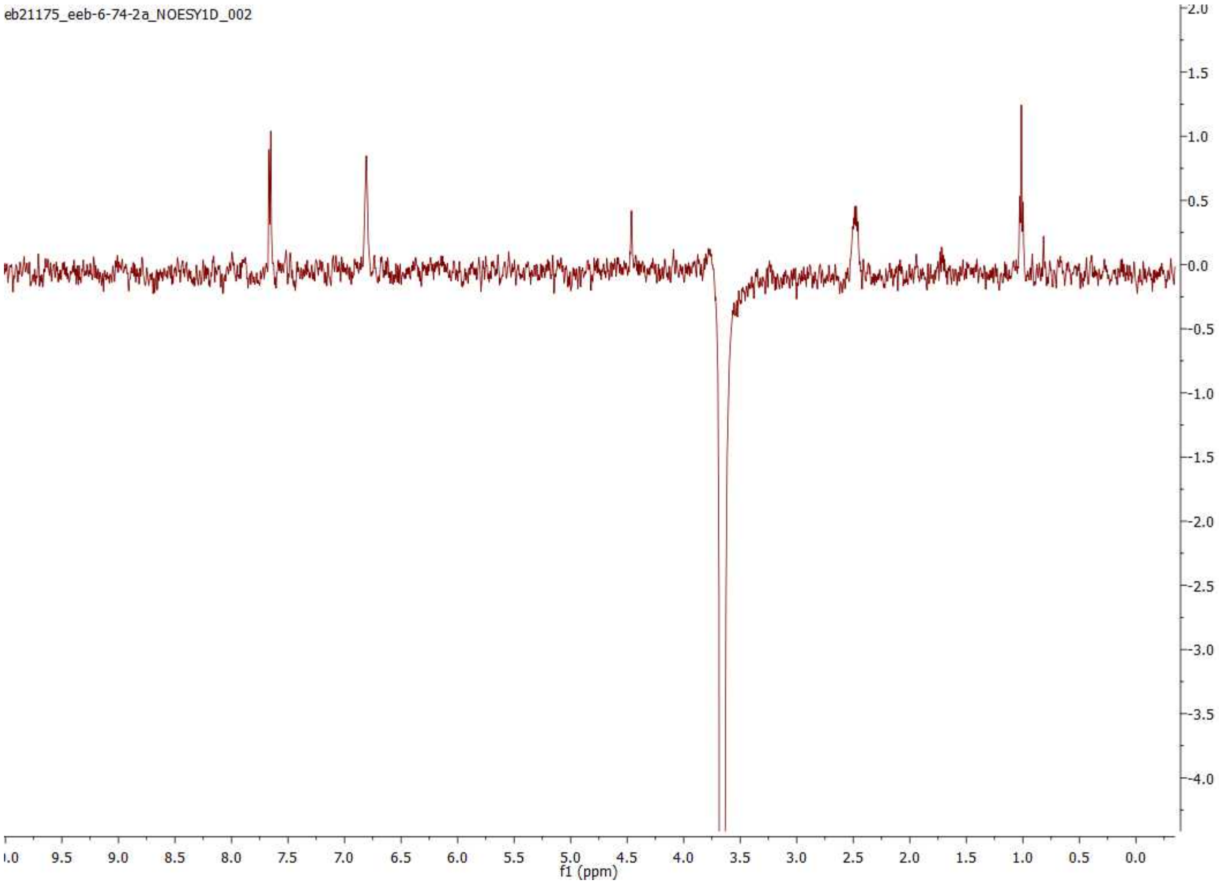

(±)-(3*R*,8a*S*)-*N*-ethyl-2-phenyl-1-tosyl-1,4,5,5a,6,8a-hexahydroimidazo[4,5,1-hi]indole-3(2*H*)-carboxamide 22a

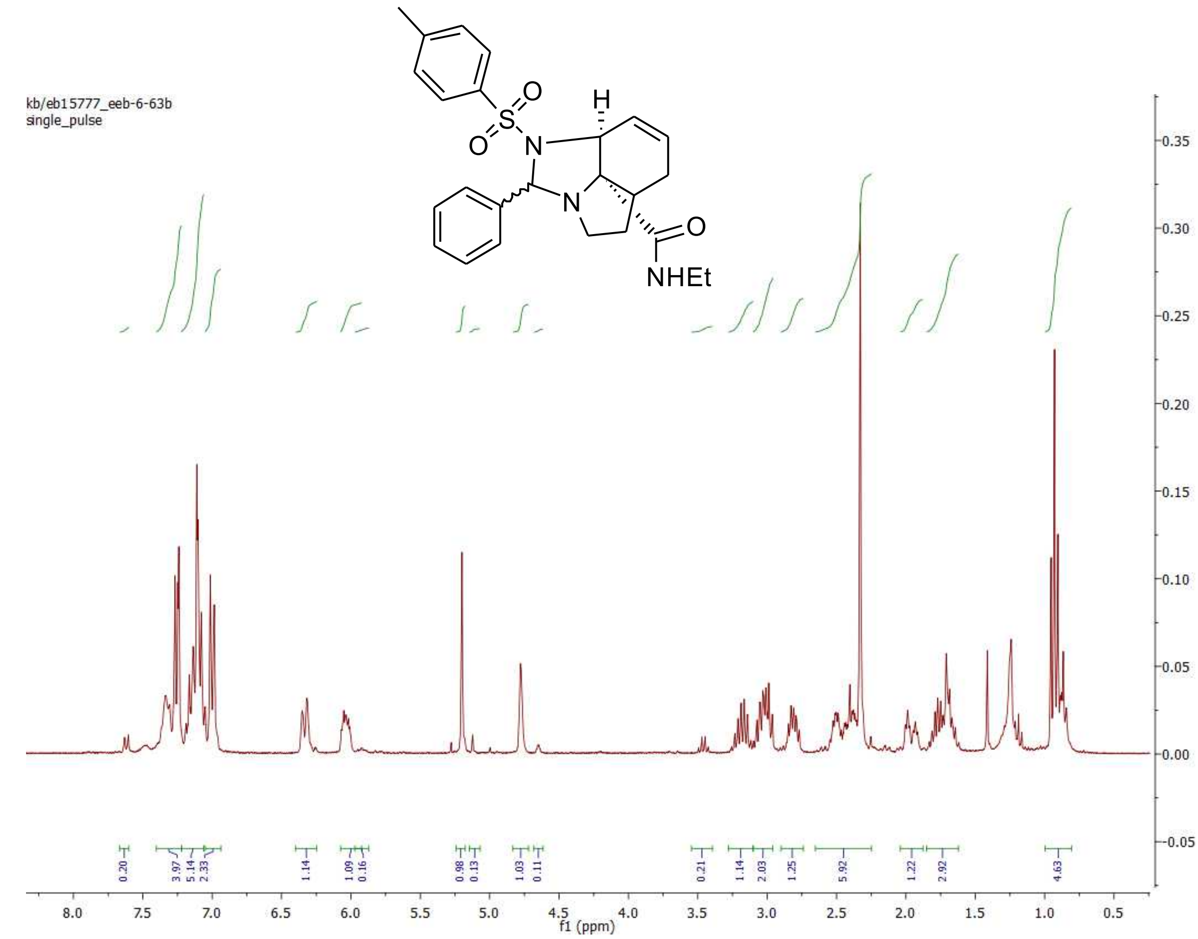

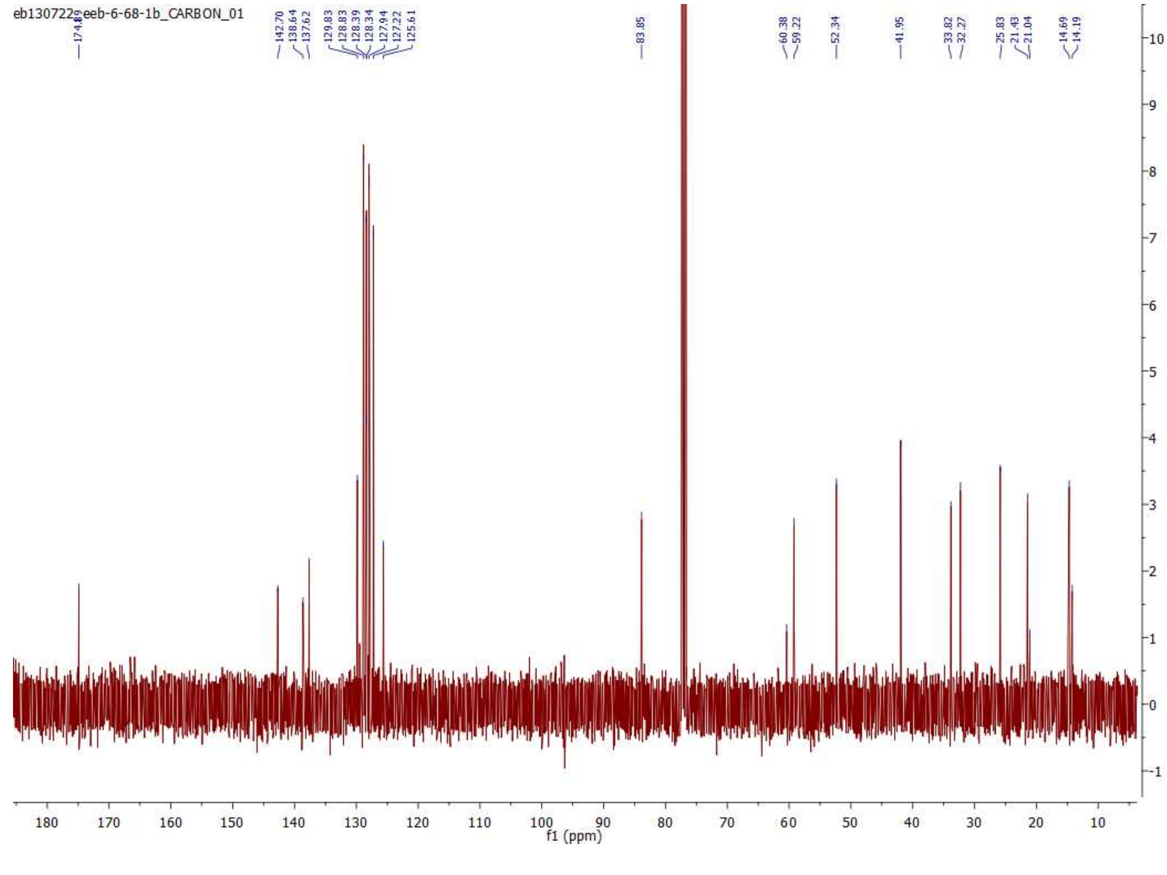

(±)-(3*R*,8a*S*)-*N*-ethyl-2-phenyl-1-tosyl-1,4,5,5a,6,8a-hexahydroimidazo[4,5,1-hi]indole-3(2*H*)-carboxamide – major diastereomer 22a

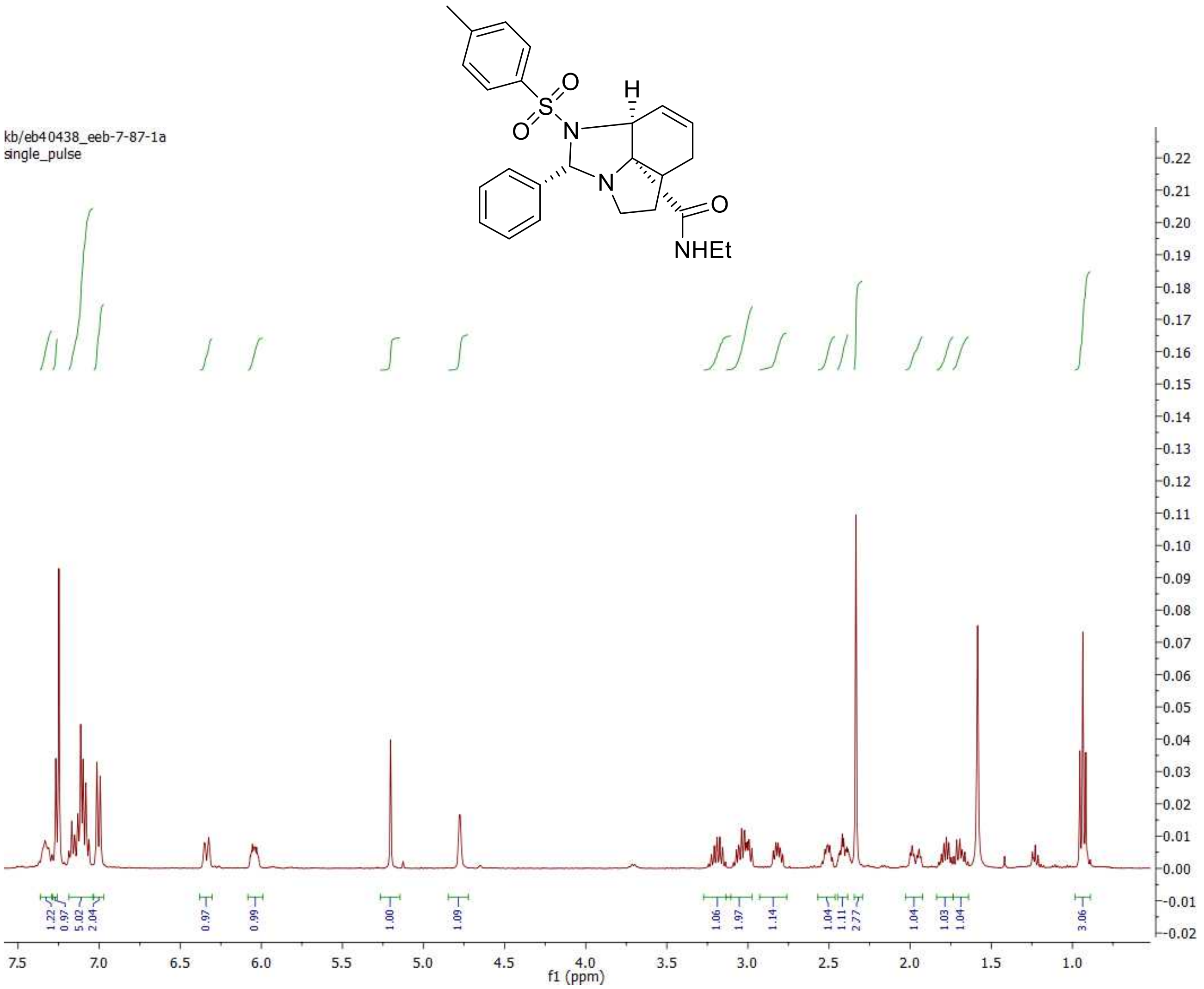

(±)-(6a*R*,8a*R*)-7-Ethyl-1-((*E*)-3-oxobut-1-en-1-yl)-2,3,3a,4,6a,7-hexahydroazeto[2,3-h]indol-8(1*H*)-one

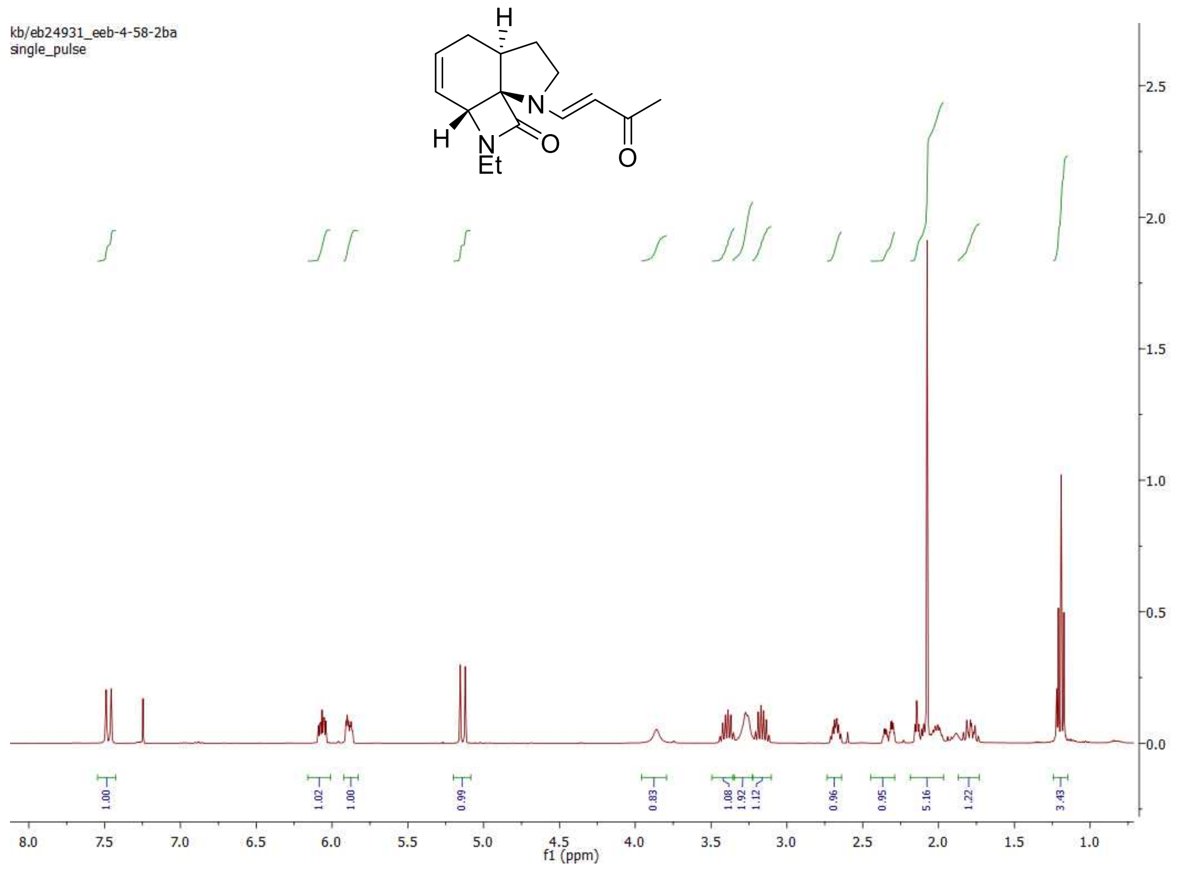

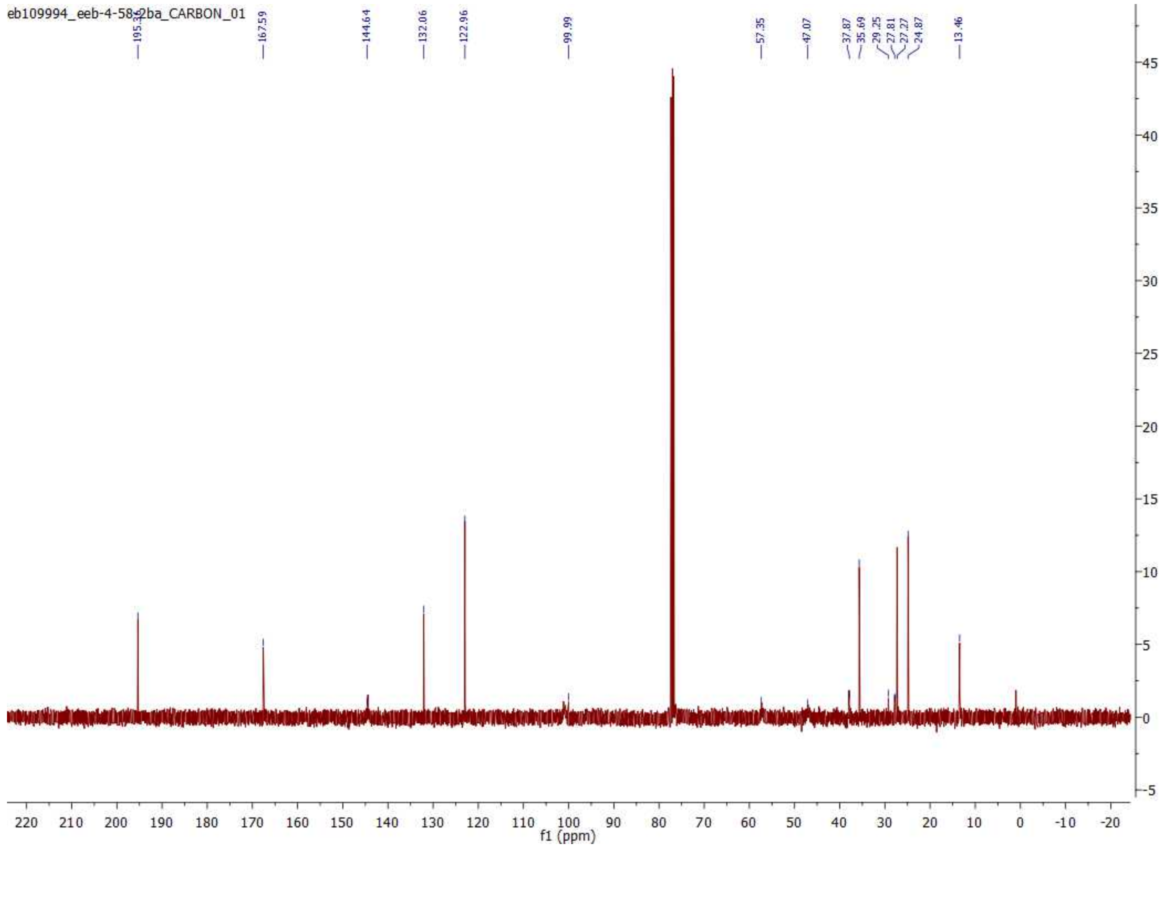

(±)-Methyl (*E*)-3-((6a*R*,8a*R*)-7-ethyl-8-oxo-3,3a,4,6a,7,8-hexahydroazeto[2,3-h]indol-1(2H)-yl)acrylate

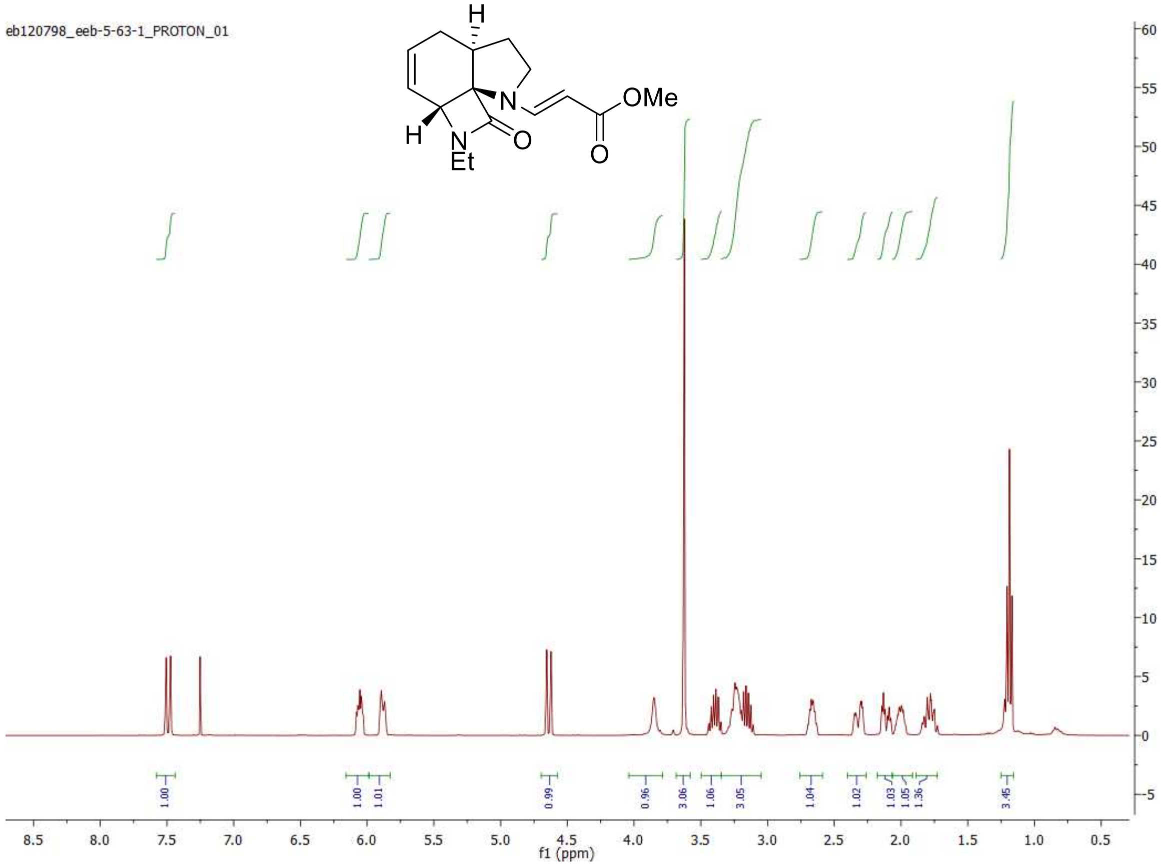

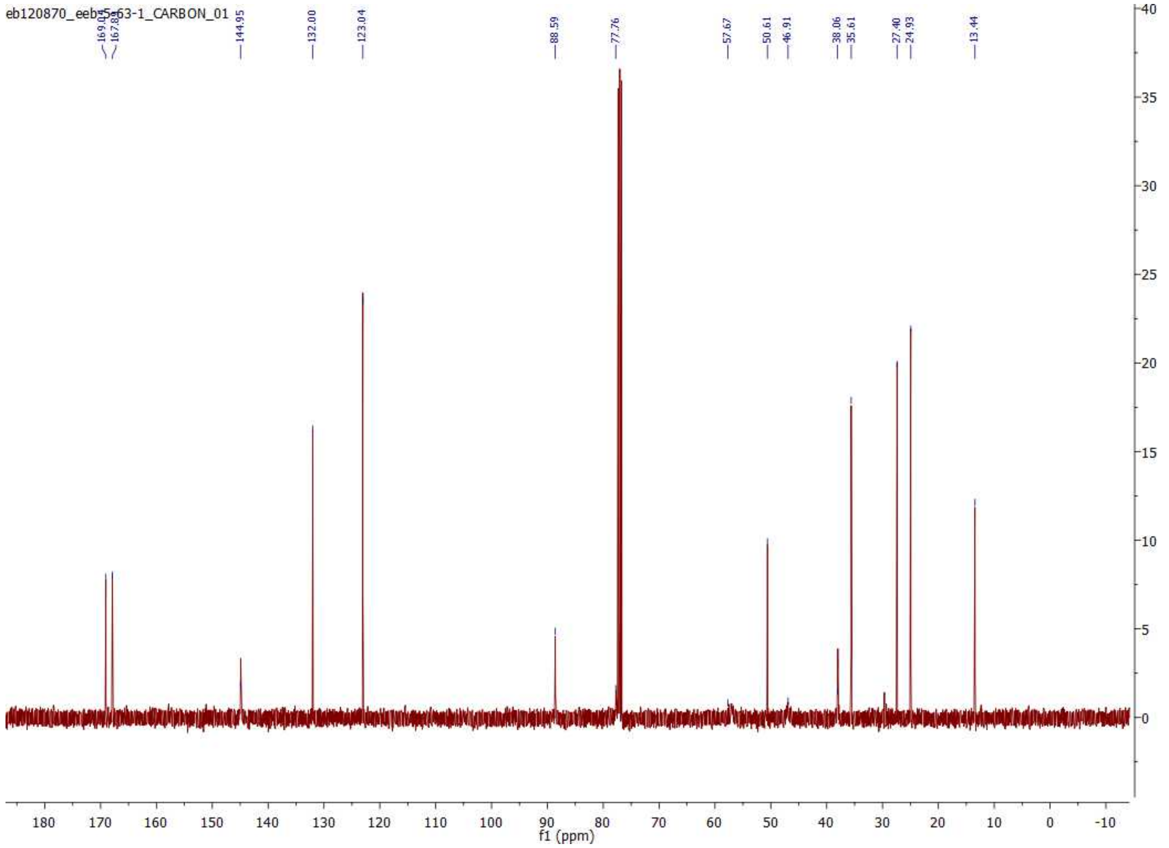

(±)-Ethyl (*E*)-3-((6a*R*,8a*R*)-7-ethyl-8-oxo-3,3a,4,6a,7,8-hexahydroazeto[2,3-h]indol-1(2*H*)-yl)acrylate

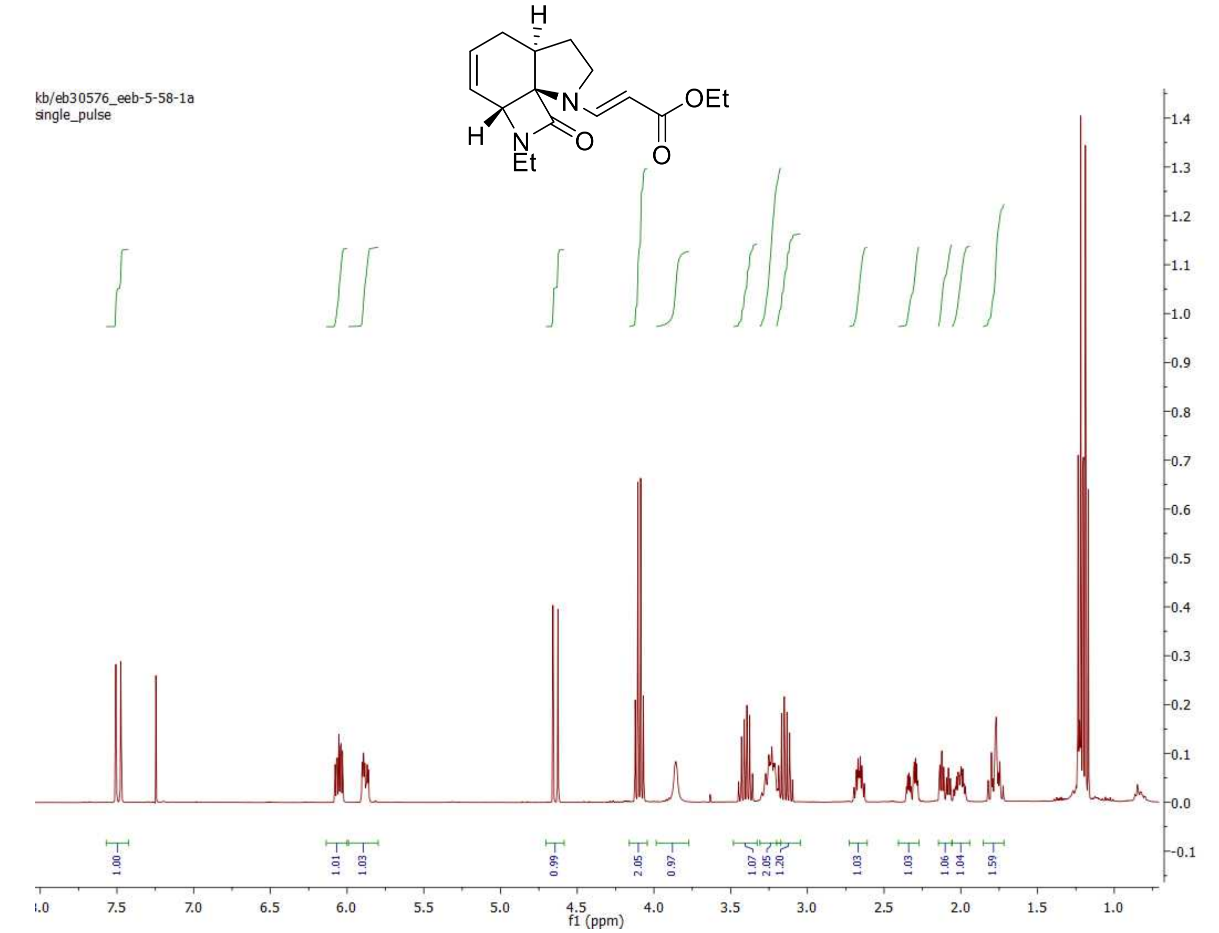

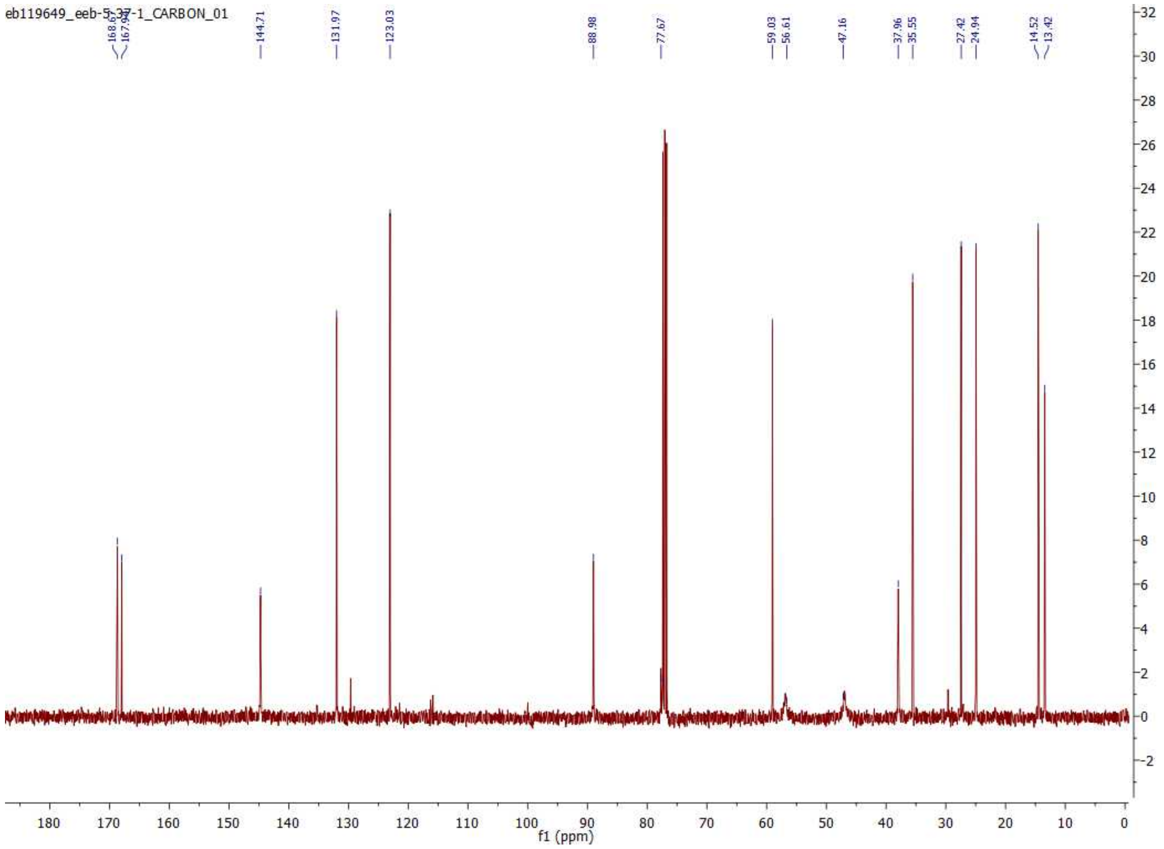

(±)-(*E*)-3-((6a*R*,8a*R*)-7-ethyl-8-oxo-3,3a,4,6a,7,8-hexahydroazeto[2,3-h]indol-1(2*H*)-yl)acrylaldehyde

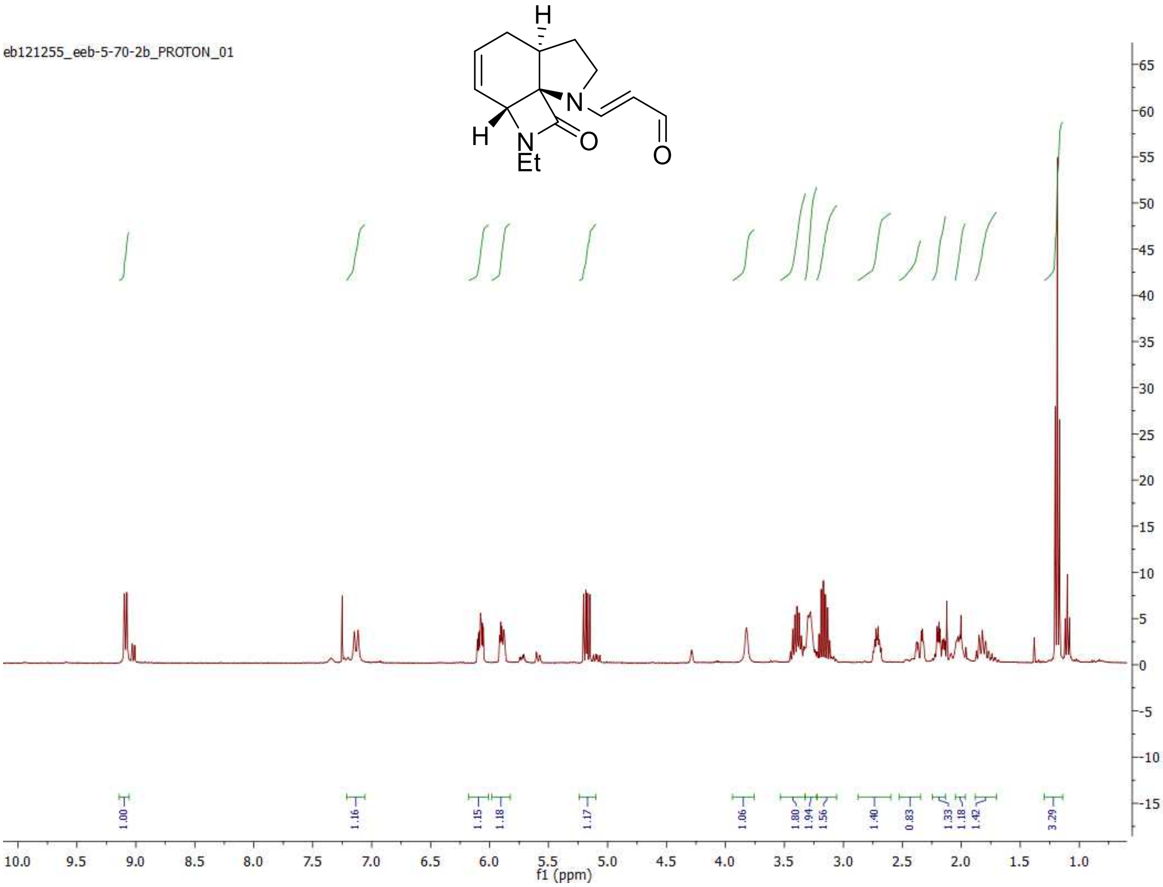

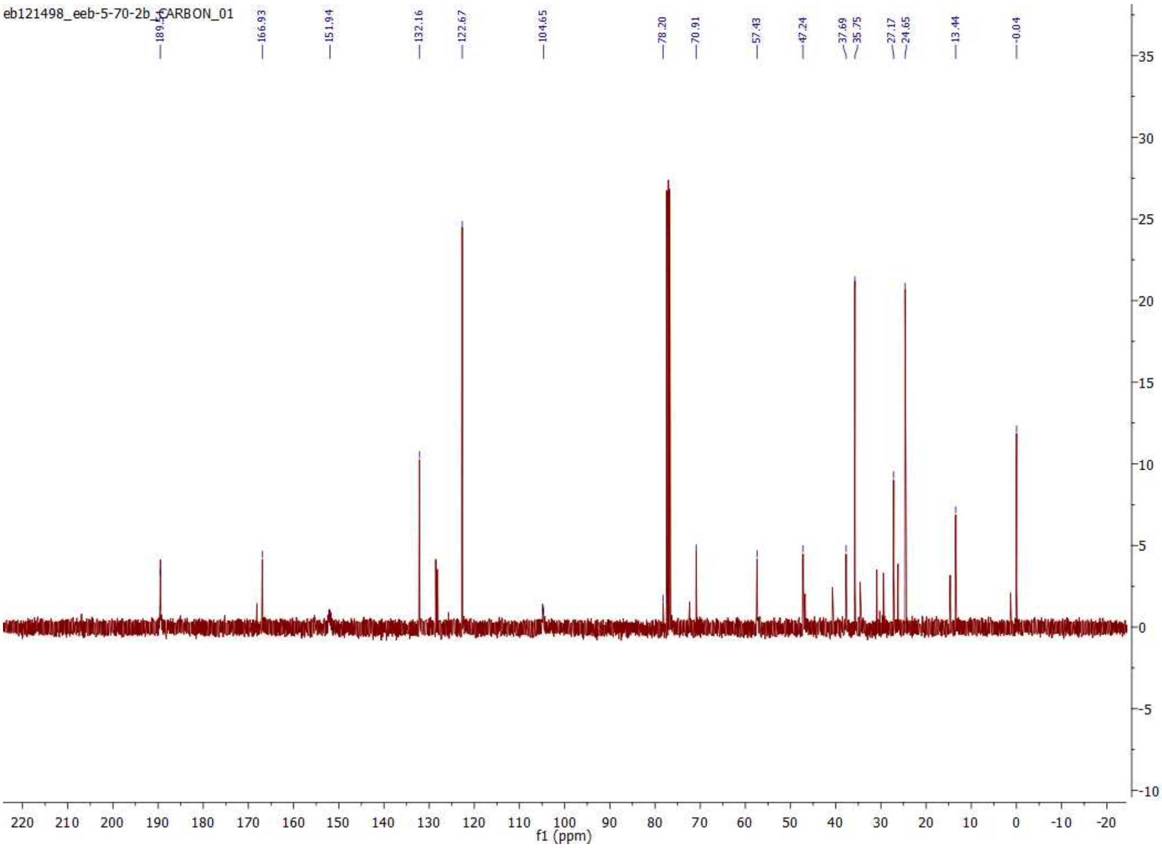

(±)-Dimethyl 2-((6a*R*,8a*R*)-7-ethyl-8-oxo-3,3a,4,6a,7,8-hexahydroazeto[2,3-h]indol-1(2*H*)-yl)maleate

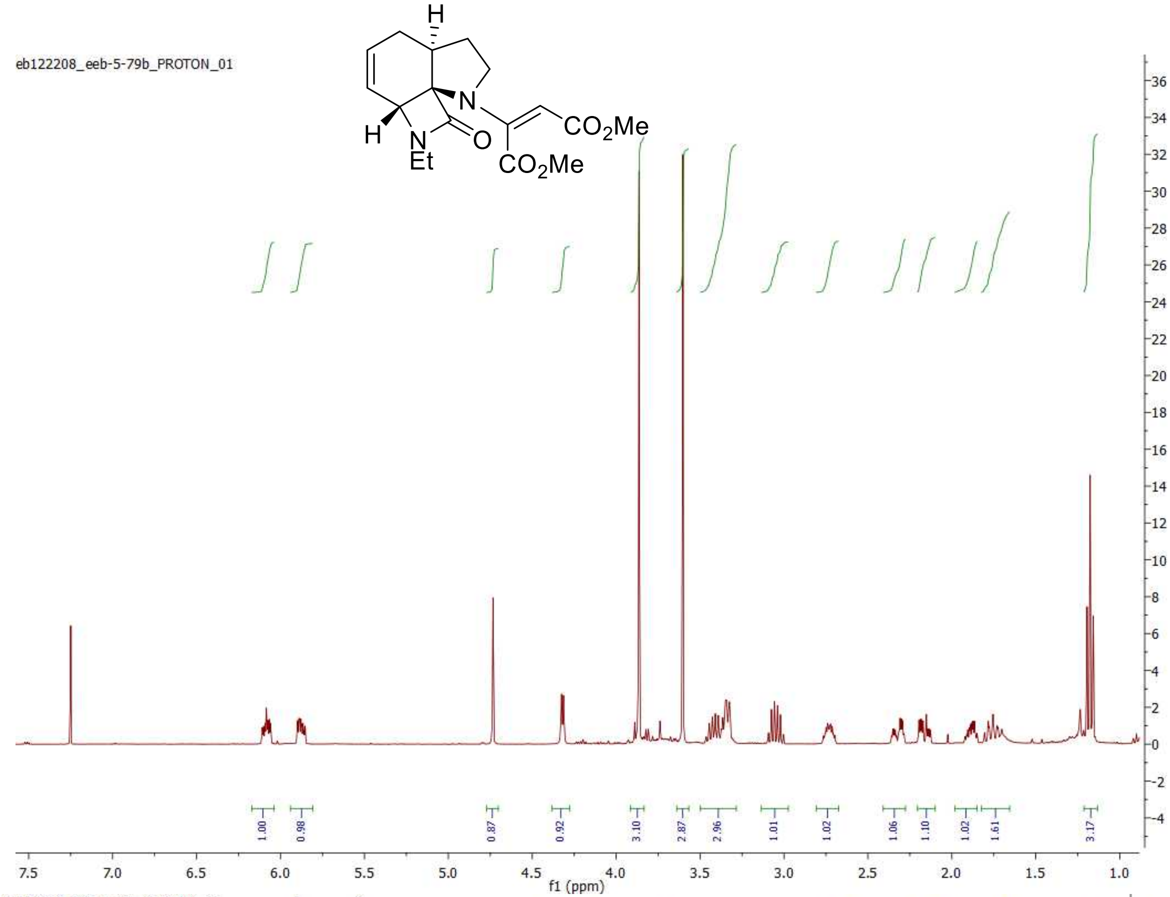

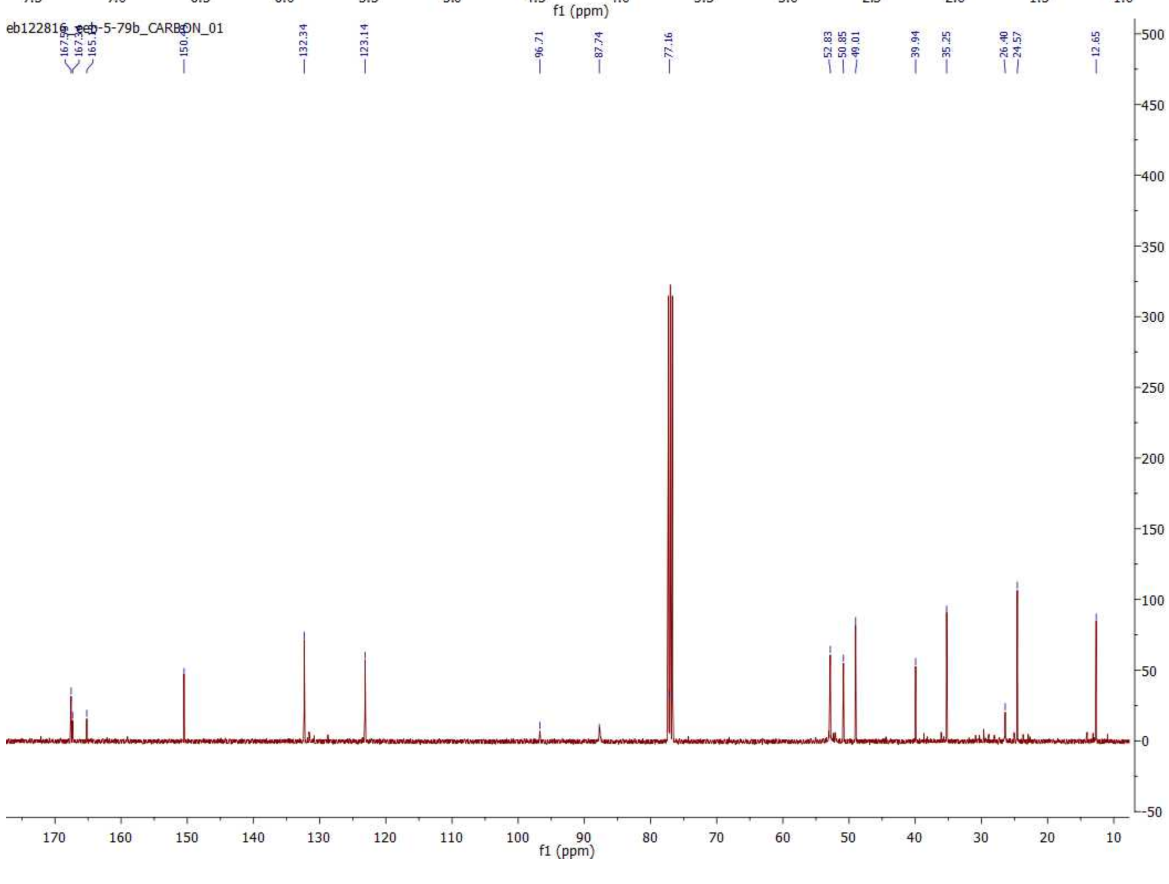

(±)-(3*R*,5a*R*)-5-acetyl-1,5a,8,8a-tetrahydropyrrolo[3,2,1-hi]indole-3(2*H*)-carbonitrile

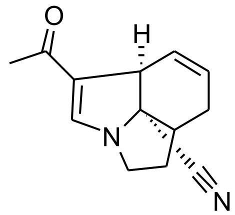

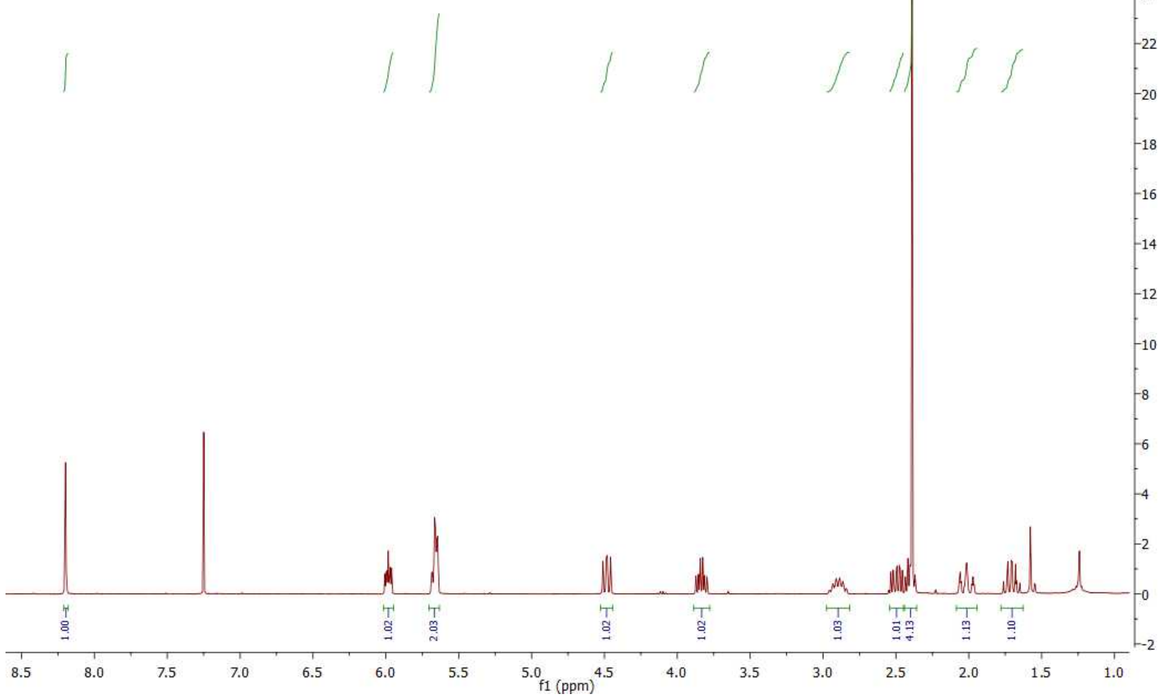

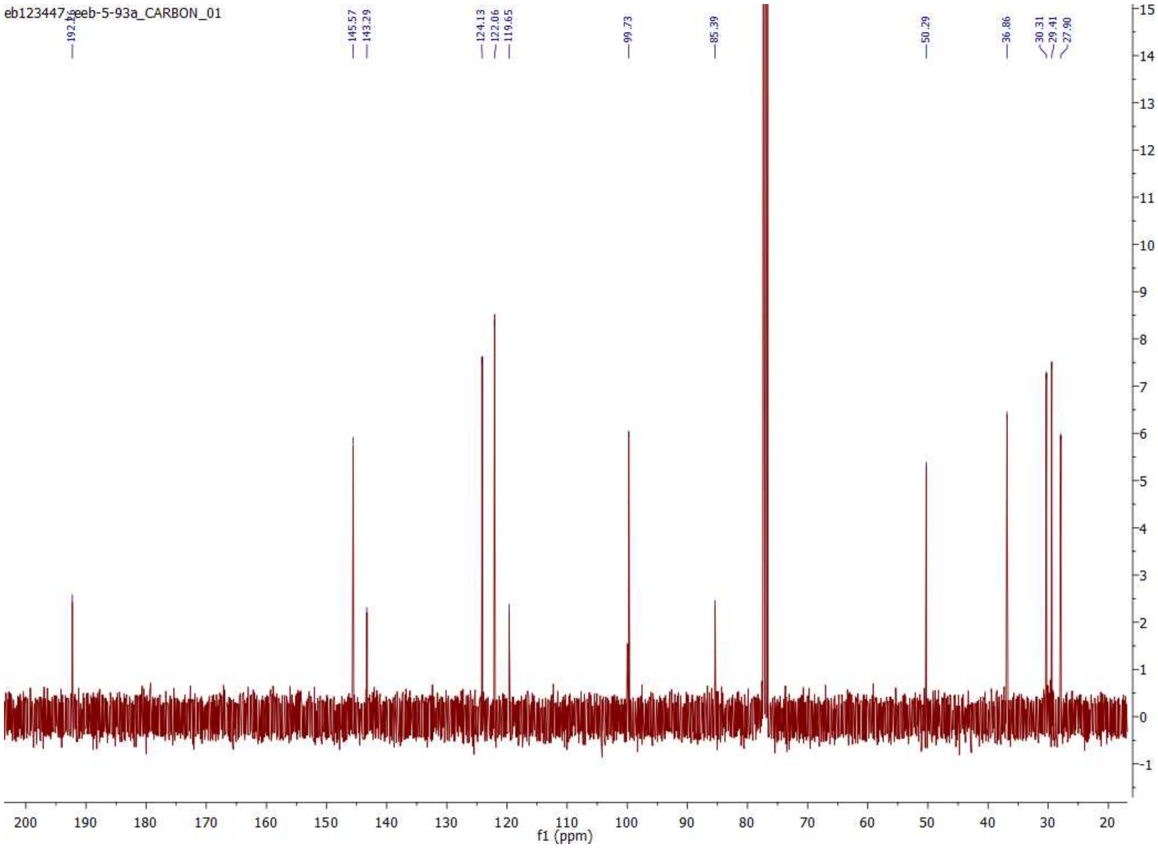

(±)-Methyl (3*R*,5a*R*)-31-cyano-1,2,31,5a,8,8a-hexahydropyrrolo[3,2,1-hi]indole-5-carboxylate

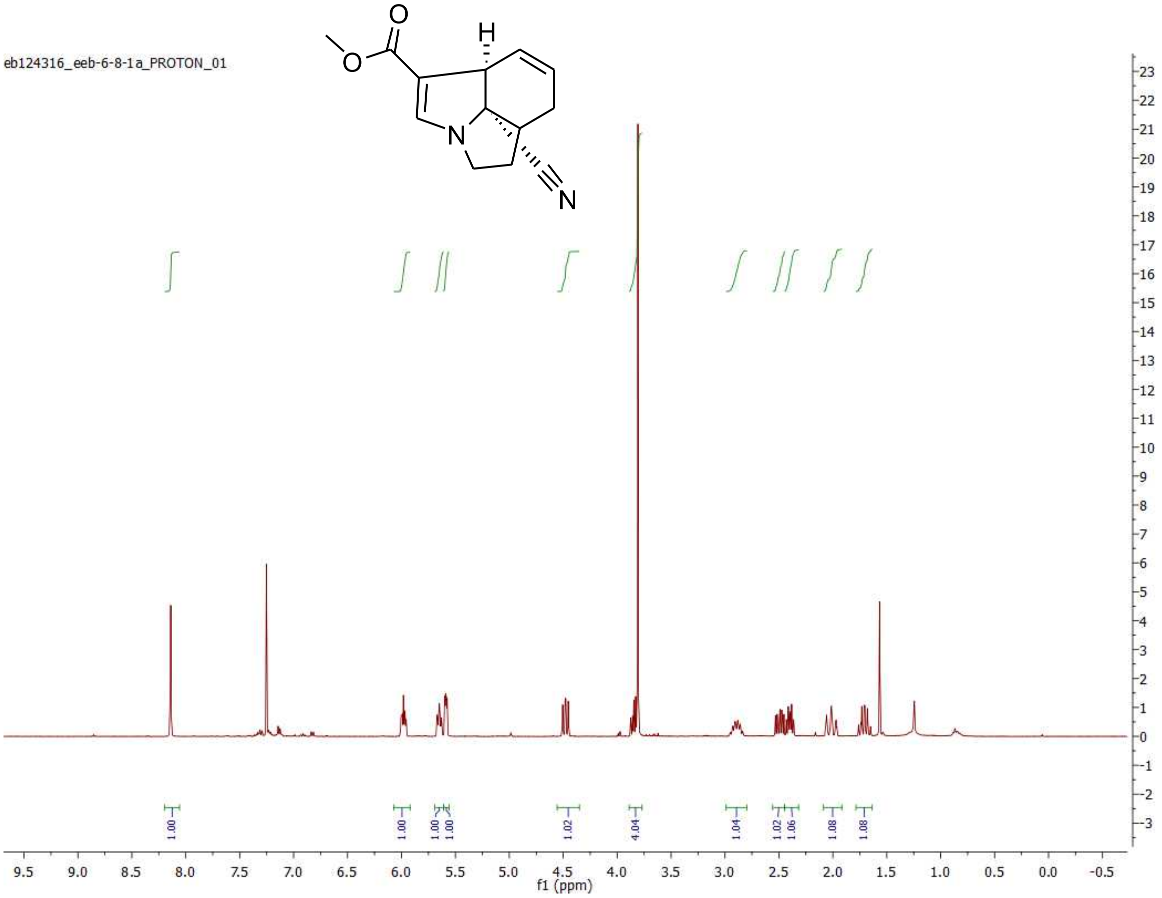

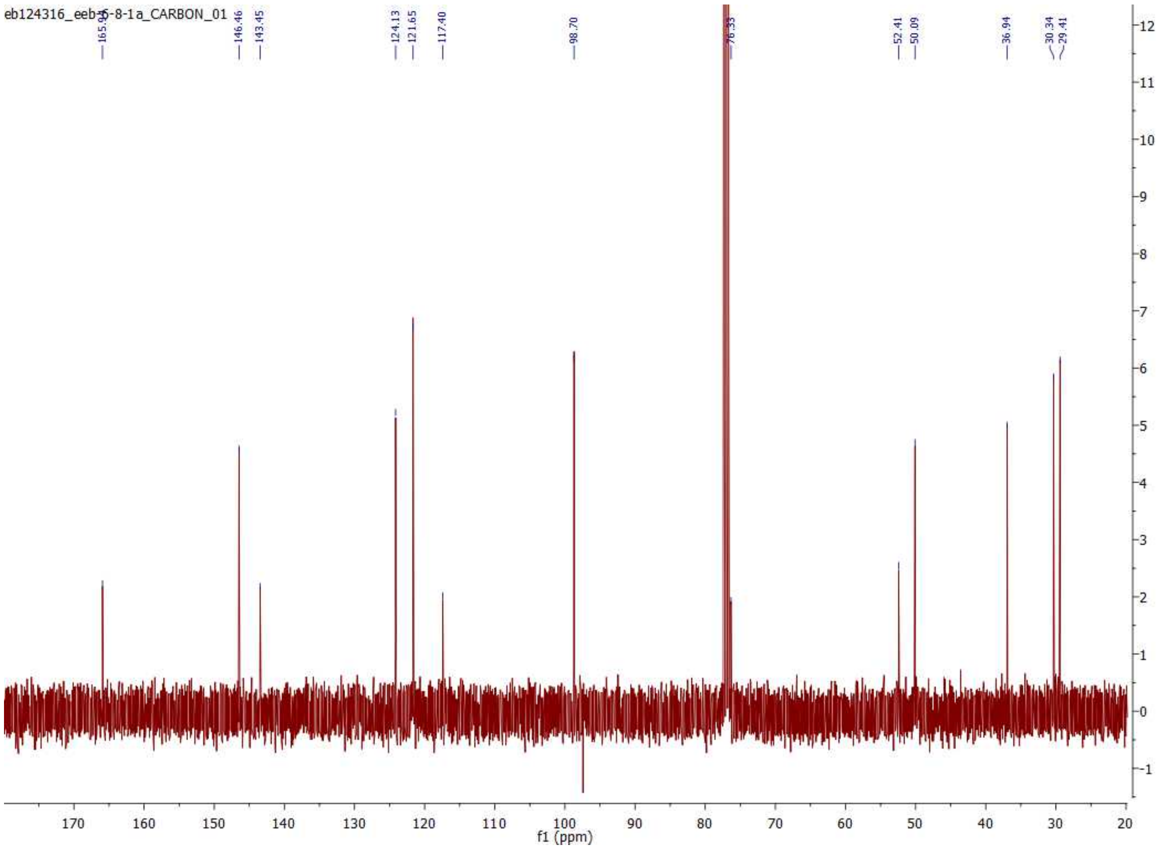

(±)-(3*R*,5a*R*)-5-formyl-1,5a,8,8a-tetrahydropyrrolo[3,2,1-hi]indole-3(2*H*)-carbonitrile

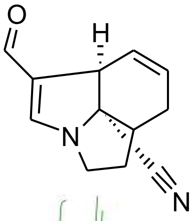

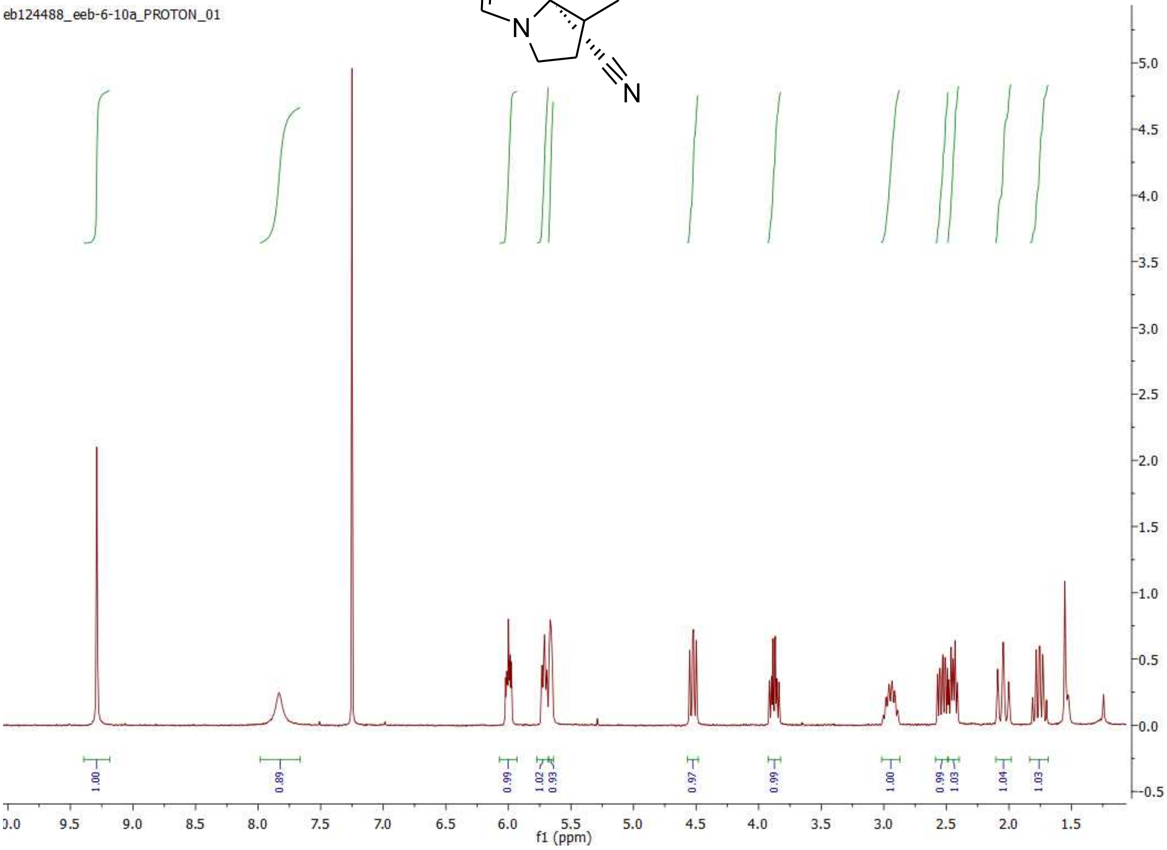

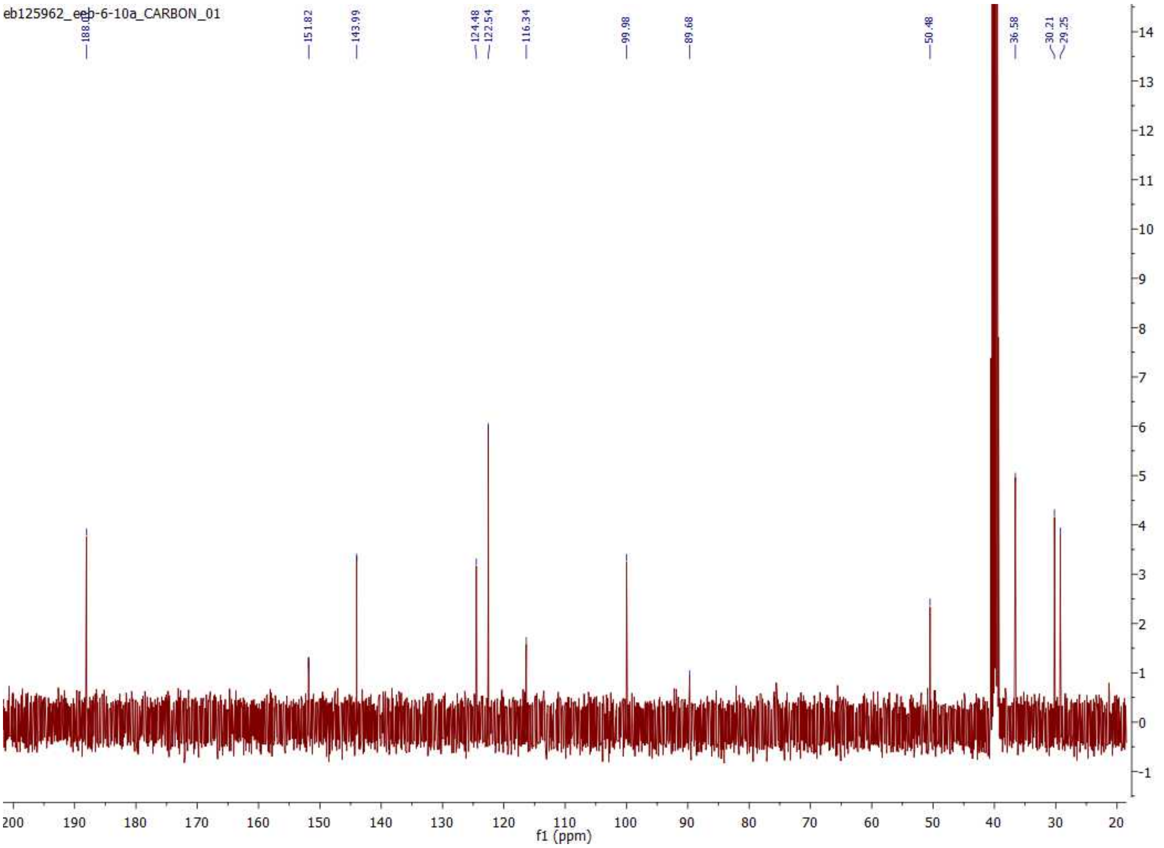

(±)-Dimethyl (3*R*,5a*R*)-31-cyano-1,2,3,5a,8,8a-hexahydropyrrolo[3,2,1-hi]indole-4,5-dicarboxylate

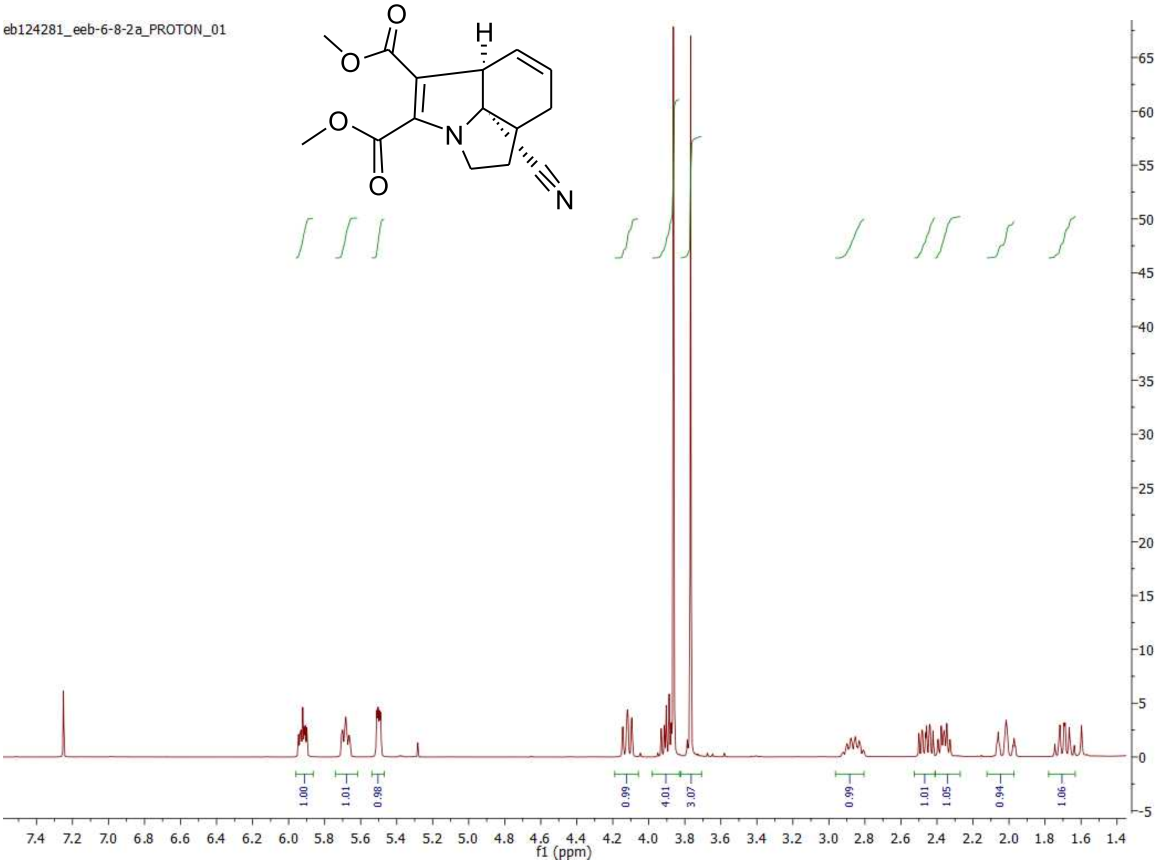

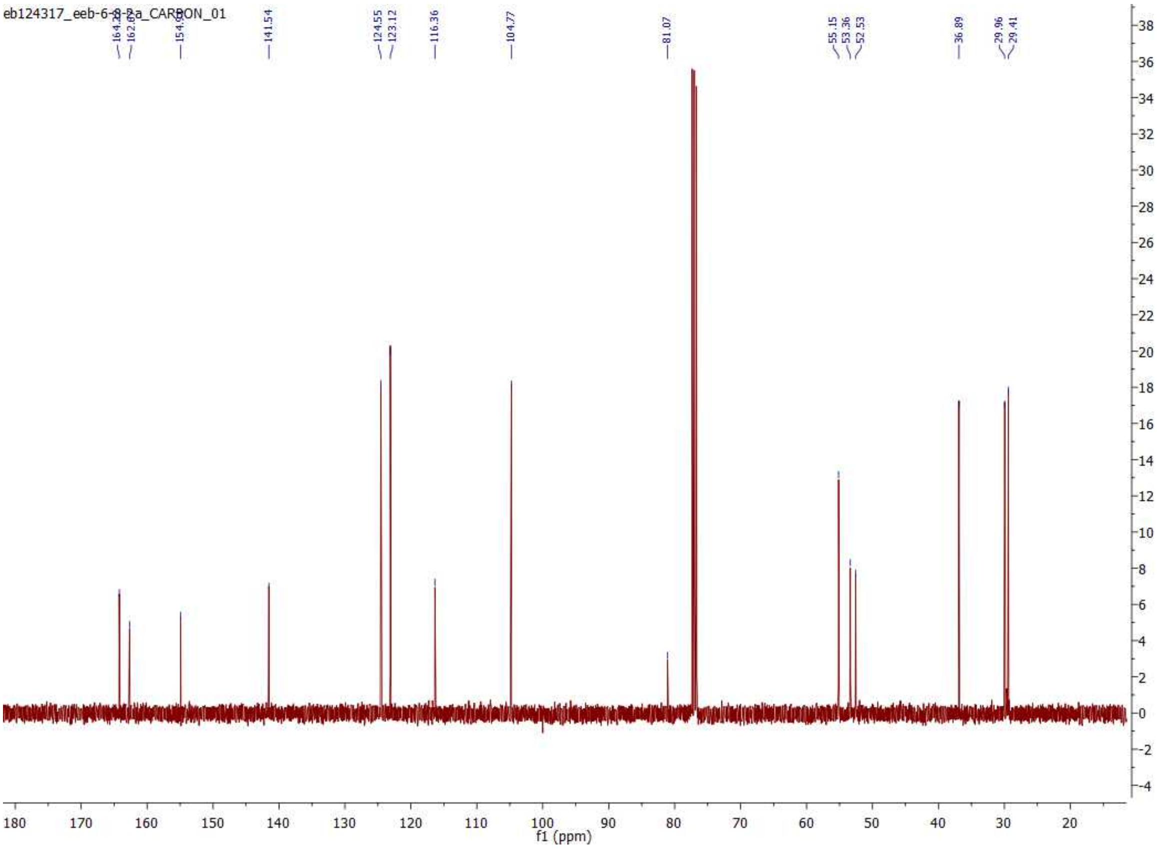

(±)-(3*R*,5a*R*)-1,5a,8,8a-tetrahydropyrrolo[3,2,1-*hi*]indole-3,5(2*H*)-dicarbonitrile

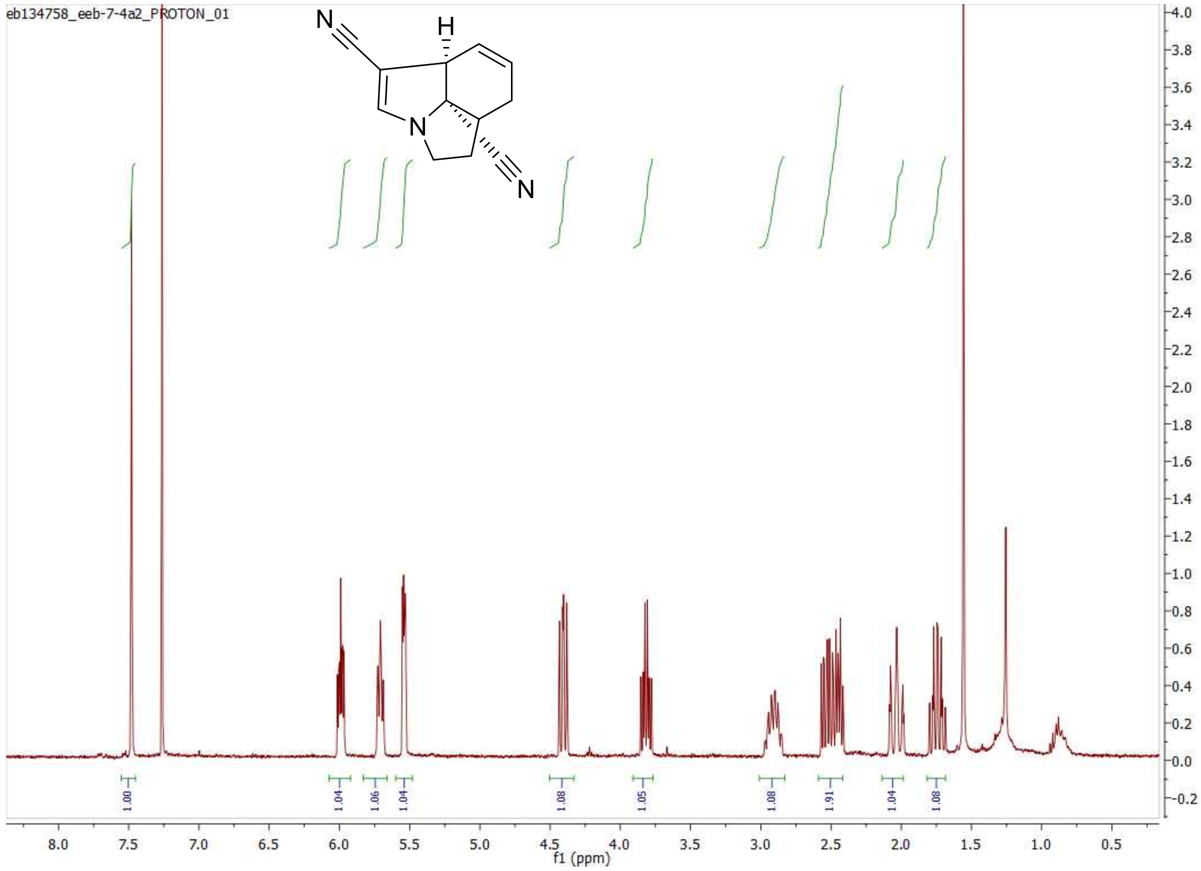

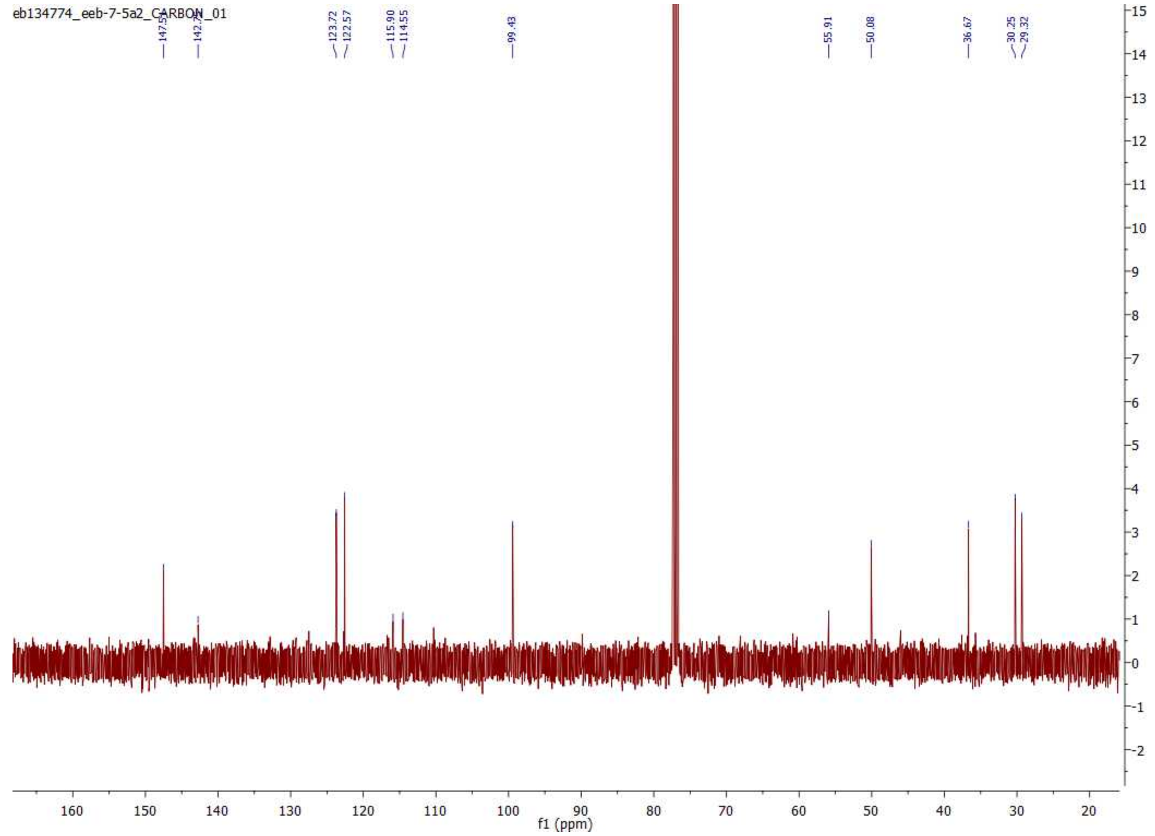

Supplement: Supplementary file 2 [file SC-007-C5SC04062K-s002.pdf]
